# The urine albumin-creatinine ratio is a predictor for incident long-term care in a general population

**DOI:** 10.1371/journal.pone.0195013

**Published:** 2018-03-28

**Authors:** Shuko Takahashi, Fumitaka Tanaka, Yuki Yonekura, Kozo Tanno, Masaki Ohsawa, Kiyomi Sakata, Makoto Koshiyama, Akira Okayama, Motoyuki Nakamura

**Affiliations:** 1 Division of Cardioangiology, Department of Internal Medicine, Iwate Medical University, Uchimaru, Morioka, Iwate, Japan; 2 St. Luke's International University, Akashi-cho, Chuo-ku, Tokyo, Japan; 3 Department of Hygiene and Preventive Medicine, School of Medicine, Iwate Medical University, Nishitokuta, Yahaba-cho, Shiwa-Gun, Iwate, Japan; 4 Morioka Tsunagi Onsen Hospital, Tsunagi, Morioka, Iwate, Japan; 5 Iwate Health Service Association, Kita-Iioka, Morioka, Iwate, Japan; 6 Research Institute of Strategy for Prevention, Arakawa, Chuo-ku, Tokyo, Japan; The University of Tokyo, JAPAN

## Abstract

**Background:**

Several types of cardiovascular diseases (CVDs) impair the physical and mental status. The purpose of this study was to assess the predictive ability of several cardiovascular biomarkers for identifying the incidence of disability as future recipients of public long-term care (LTC) service.

**Methods:**

The subjects of this study were community-dwelling elderly individuals ≥ 65 years of age without a history of CVD (n = 5,755; mean age, 71 years). The endpoint of this study was official certification as a recipient of LTC. The cohort was divided into quartiles (Qs) based on the levels of three CVD biomarkers: the urinary albumin-creatinine ratio (UACR), plasma B-type natriuretic peptide concentration (BNP), and serum high-sensitivity C-reactive protein concentration (hsCRP). A time-dependent Cox proportional hazard model was used to determine the multi-adjusted relative hazard ratios (HRs) for incident LTC among the quartiles of each biomarker.

**Results:**

During the follow-up (mean 5.6 years), 710 subjects were authorized as recipients of LTC. The HR was only significantly increased in the higher Qs of UACR (Q3, p < 0.01; Q4, p < 0.001). However, other biomarkers were not significantly associated with the endpoint. The risk predictive performance for the incidence of LTC as evaluated by an essential model (i.e. age- and sex-adjusted) was significantly improved by incorporating the UACR (net reclassification improvement = 0.084, p < 0.01; integrated discrimination improvement = 0.0018, p < 0.01).

**Conclusions:**

These results suggest that an increased UACR is useful for predicting physical and cognitive dysfunction in an elderly general population.

## Introduction

The high prevalence of disabled elderly individuals who require long-term care (LTC) for physical and cognitive functional impairments is a significant public health problem in aging societies. To help elderly individuals with disabilities successfully manage their daily life, the Japanese government has recently implemented an LTC support system based on several previous European programs [1]. This system is a form of social support for those ≥65 years of age who cannot manage their daily life due to physical and mental disability, and the type and degree of the support was determined by structural questionnaires and a medical interview to evaluate their physical and cognitive dysfunctions [2].

Because the elderly population is rapidly growing in several developed countries, the number of candidates for LTC may increase sharply in the near feature. In particular, the number of older adults with disabilities is estimated to increase from 10 million in 2000 to 21 million in 2040 in the United States [3]. Similarly, in Japan, the number certified for LTC was estimated to be 4.9 million in 2015 and is predicted to increase to 5.3 million in 2025. Therefore, effective preventive measures for physical and psychological dysfunctions in the elderly population may be urgently required [4]. However, the detailed characteristics of the people at risk for disability in the future have not yet been fully determined in the general population. As several types of cardiovascular diseases (CVDs), such as stroke, coronary artery disease, and heart failure, impair not only physical conditions but also the mental status [5–7], we hypothesized that certain CVD biomarkers might be useful for assessing the risk of disability in mass screening settings.

The present study, therefore, compared recently well-studied CVD risk biomarkers—namely the urine albumin-creatinine ratio (UACR), plasma B-type natriuretic peptide (BNP), and serum high-sensitive C-reactive protein (hsCRP)—for their ability to predict the incidence of LTC certification among the elderly general population.

## Materials and methods

The research plan was deliberated and approved by the Ethics Committee of Iwate Medical University Institute Review Board #1 (approval no. H13-33). The rights and welfare of the participants in this study were protected by the ethical guidelines outlined in the Declaration of Helsinki.

### Study population

**Fig 1:**
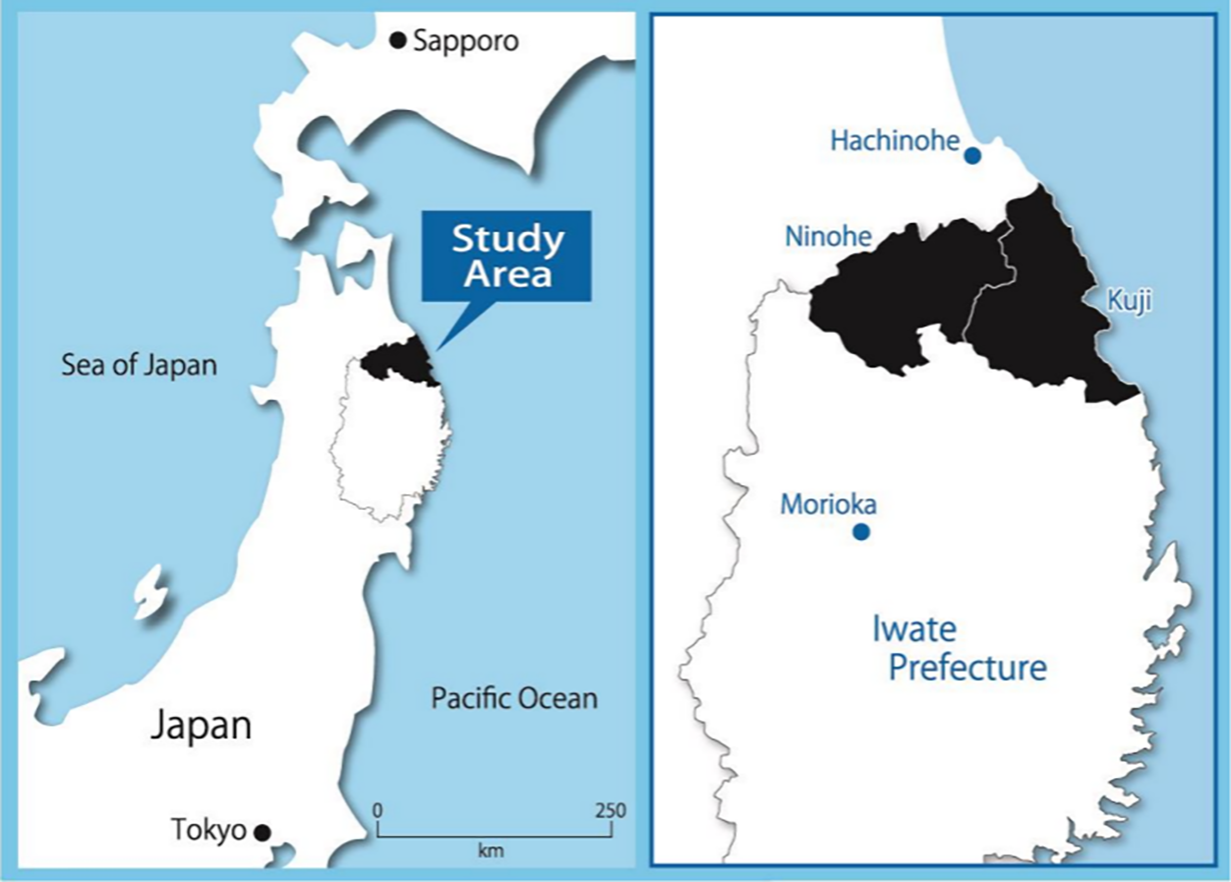
Map of the study area. The black zone indicates the study area, including Kuji and Ninohe in northern Iwate Prefecture, northeast of Honshu, Japan. The population was 131,341 in the study area at the baseline survey (2002).

**Fig 2:**
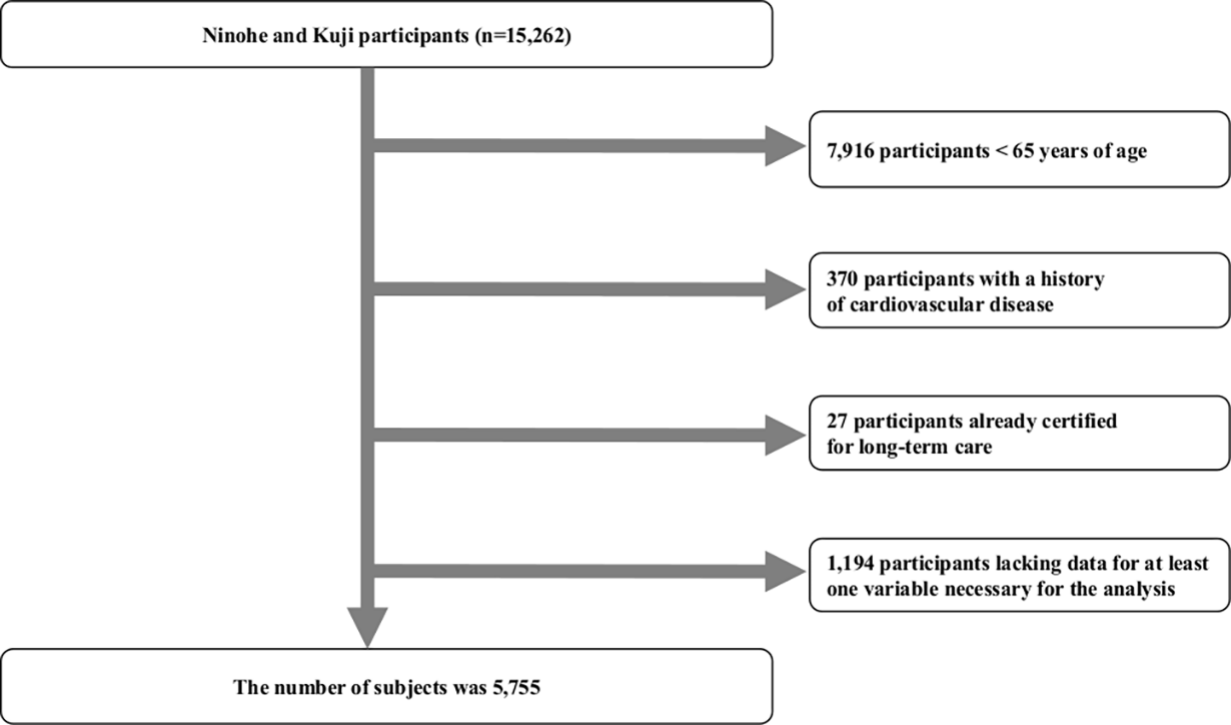
Flow chart of the procedure used to select participants for this study. The UACR, BNP, and hsCRP were measured in 15,262 participants living in the Ninohe and Kuji districts between 2002 and 2005. Among those participants, those < 65 years of age, who had a history of cardiovascular diseases (stroke, myocardial infarction or heart failure), who were already receiving LTC, or who lacked at least 1 variable for this study analysis were excluded. The final number of subjects in this study was 5,755.

The original cohort of the Iwate-KENCO study was recruited from a community-based population living in the Ninohe, Kuji, and Miyako districts of northern Iwate Prefecture, Japan. The cohort was recruited from among the subjects of a government-regulated multiphasic health checkup for the general population. Invitations to participate in the multiphasic health screening program were issued by government offices in each municipality. Of participants who took part in multiphasic health screening program, the total number of subjects who agreed to join the Iwate-KENCO study in the above 3 districts was 26,469 (original cohort, acceptance rate of 84.5%). Baseline examinations including CVD biomarker measurement and ECG recording were performed between 2002 and 2005. The details of recruitment and baseline measurements have been described in our previous reports [8]. In the original cohort, the UACR, BNP, and hsCRP were measured in 15,262 participants living in the Ninohe and Kuji districts (Fig 1). After excluding people <65 years of age (n = 7,916) or who had a history of stroke, myocardial infarction, or heart failure (n = 370); those who were already receiving LTC (n = 27); and those who lacked data for at least 1 variable that was necessary for the analysis (n = 1,194), we ultimately analyzed data for 5,755 participants (2,212 males and 3,543 females, mean age 71.3 years) (Fig 2).

### Baseline data and questionnaires

Systemic blood pressure was measured by experienced technicians in the sitting position using an automatic digital device (BP-103i IIModel 513000; Nippon Colin, Komaki, Japan). All subjects rested for at least 5 min before measurement. Measurements were performed twice, with the mean value used for statistical analyses. Anthropometrical examinations (body weight [kg] and height [cm]) were performed. Body weight was measured with an accuracy of ± 0.1 kg using a standard scale while dressed in very light clothing without shoes. The body mass index was calculated as the weight (kg) divided by the square of the height (m^2^).

Blood samples were drawn from a peripheral vein while the subject was seated. An enzymatic method was used to measure the serum levels of total cholesterol (TC; mg/dl). A direct quantitative assay was used to measure the high-density lipoprotein cholesterol (HDLC; mg/dl). The quality of TC and HDLC measurements was controlled by the program of the Centers for Disease Control in the United States. Non-high-density lipoprotein cholesterol (non-HDLC; mg/dl) was calculated by subtracting HDLC from TC. The serum creatinine level was determined by an enzymatic method using an auto-analyzer (Hitachi 7700 automatic analyzer; Hitachi, Tokyo, Japan). The estimated glomerular filtration rate (eGFR; mL/min/1.73 m^2^) was calculated using the formula devised by the Chronic Kidney Disease Epidemiology Collaboration (CKD-EPI). Glycosylated hemoglobin (HbA1c; %) was measured quantitatively with high-performance liquid chromatography (Tosoh, Tokyo, Japan). The value for HbA1c (Japan Diabetes Society; JDS) was estimated as an equivalent value of the National Glycohemoglobin Standardization Program (NGSP) calculated by the following formula: HbA1c (NGSP) = HbA1c (JDS) + 0.4.

All subjects used a self-reported questionnaire developed by the study committee to document their medical history, including the status (yes or no) of prescribed drugs for hypertension, diabetes mellitus, dyslipidemia, stroke, myocardial infarction, and heart failure. Smoking status (current or non-smoker) and alcohol status (drinker or non-drinker) were also assessed by a questionnaire (S1 Table). The level of education was classified into 2 categories according to the duration of education: low (< 7 years) and high (≥ 7 years).

### Biomarkers

At the baseline examination, a single-void urine sample was collected during the daytime and was used to measure the UACR (mg/g creatinine). We did not assess the presence of bacteria or white blood cells in the urine, but febrile patients were excluded. Urinary albumin was assessed quantitatively using an immunonephelometric method (N antiserum albumin, Dade Behring, Tokyo, Japan), and urinary creatinine was measured quantitatively using an enzymatic colorimetric test (Hitachi 7700 automatic analyzer; Hitachi). The sensitivity limit for albumin was 6 mg/l. The inter- and intraassay coefficients of variation were both within 5%. Subjects showing levels below the sensitivity limit were regarded as having ‘no microalbuminuria’, irrespective of their urine creatinine concentration. When blood samples for routine blood testing were being taken, an additional 2 ml was collected into a test tube containing EDTA-2Na for plasma BNP measurement. Plasma BNP levels were measured by direct radioimmunoassay using monoclonal antibodies specific for human BNP (Shiono RIA BNP kit; Shionogi, Osaka, Japan). The cross-reactivity of the antibody was 100% for human BNP and 0.001% for human arterial natriuretic peptide. The intra- and interassay coefficients of variation were 5% and 6%, respectively. The serum levels of hsCRP were measured using the Behring latex-enhanced CRP assay on a Behring nepherometer BN-100 (Germany). Both within- and between-assay quality control procedures were used, and the coefficient of variation of the method was less than 2%. The hsCRP values in the calibrator were assigned using CRM-470 (IRMM, Geel, Belgium), an international plasma protein reference material.

### Risk factors

Regarding risk factors, hypertension was defined as a systolic blood pressure ≥ 140 mmHg and/or diastolic blood pressure ≥ 90 mmHg and/or the use of antihypertensive medications. Diabetes mellitus was defined as a non-fasting glucose concentration ≥ 200 mg/dl and/or fasting blood glucose level ≥ 126 mg/dl and/or hemoglobin A1_c_ value ≥ 6.5% and/or the use of antidiabetic agents, including insulin. Dyslipidemia was defined as a serum TC ≥ 220 mg/dl, serum HDLC < 40 mg/dl, and/or the use of anti-lipidemic medications. Atrial fibrillation subjects were selected based on standard 12-lead ECG tracings. CVD was assessed by follow-up survey after the baseline study. We defined CVD as a composite of stroke, myocardial infarction, or heart failure. The details of this process have been shown in previous reports [9]. Interim CVD was defined as any types of CVD event (i.e. stroke, myocardial infarction, or heart failure) before being certified to receive LTC.

### Endpoint measurement

The endpoint of this study was official certification to receive LTC. The levels of LTC were objectively determined by qualified personnel using structural questionnaires and medical interviews to evaluate patients’ physical and cognitive abilities [1]. The present certification system for LTC was graded by two degrees comprising “Support Levels (persons who require daily assistance)” and “Care Need (persons who are bedridden and suffering from dementia and/or physical impairment)”. In the present study, LTC of any level in the above categories was defined as the endpoint. During the follow-up (mean 5.6 years), 710 subjects within the cohort (n = 5,755) were authorized as recipients of LTC (2.2% per year).

### Statistical analyses

Continuous variables are expressed as the mean ± standard deviation (SD). Group comparisons between the LTC and non-LTC groups were based on the chi-squared test, Student’s *t-*test, or the Kruskal-Wallis test.

We showed the hazard ratios of the risk of incidence of interim CVD among quartiles (Qs) of each biomarker (UACR, BNP, and hsCRP; see median, IQR in Table 1) using a multivariate Cox proportional hazard regression analysis (cofounders: age, sex, BMI, SBP, TC, HDLC, blood hemoglobin, HbA1c, eGFR, the duration of education, atrial fibrillation, smoking status, and drinking status). To determine the incident risk for LTC, time-dependent Cox regression analyses adjusted by multiple confounders, including interim CVD, were employed to calculate the hazard ratios (HRs) of the Qs for each biomarker.

**Table 1:** A comparison of the baseline characteristics between the LTC and non-LTC groups.

		LTC (n = 710)	non-LTC (n = 5,045)	*p*-values
**Sex (%)**	**Males**	34.8	38.9	0.033*
**Age (years)**		74.8 ± 5.0	70.8 ± 4.3	<0.001*
**Clinical data**	**BMI (kg/m**^**2**^**)**	23.9 ± 3.5	24.1 ± 3.2	0.225
	**SBP (mmHg)**	132.6 ± 18.8	131.2 ± 19.6	0.083
	**DBP (mmHg)**	74.8 ± 10.1	75.5 ± 10.5	0.055
	**TC (mg/dl)**	199.2 ± 32.1	200.7 ± 31.7	0.228
	**HDLC (mg/dl)**	58.8 ± 14.3	58.7 ± 14.9	0.950
	**Non-HDLC (mg/dl)**	140.4 ± 32.0	142.0 ± 31.3	0.212
	**Hb (g/dl)**	13.3 ± 1.4	13.5 ± 1.3	<0.001*
	**HbA1c (%)**	5.2 ± 0.8	5.6 ± 0.7	0.166
	**eGFR (mL/min/1.73 m**^**2**^**)**	68.3 ± 10.7	71.9 ± 8.5	<0.001*
**Educational years (%)**	**< 7 years**	36.6	21.4	<0.001*
**Current smoker (%)**		10.8	9.8	0.379
**Current drinker (%)**		25.4	31.3	0.001*
**Hypertension (%)**		60.7	52.6	<0.001*
**Diabetes mellitus (%)**		18.5	12.4	<0.001*
**Dyslipidemia (%)**		34.1	37.5	0.074
**Atrial fibrillation (%)**		3.9	1.8	<0.001*
**Interim CVD**	**Overall (%)**	17.9	3.2	<0.001*
	**Stroke (%)**	14.9	2.3	
	**MI (%)**	0.5	0.4	
	**HF (%)**	1.6	0.6	
	**Stroke and MI (%)**	0.0	0.1	
	**Stroke and HF (%)**	0.3	0.0	
	**HF and MI (%)**	0.3	0.1	
**Biomarkers (median, IQR)**				
	**UACR (mg/g Cr)**	21.8 (11.1–49.7)	15.8 (6.1–32.9)	<0.001*
	**BNP (pg/ml)**	28.4 (15.3–54.4)	22.8 (12.3–39.9)	<0.001*
	**hsCRP (mg/l)**	0.5 (0.3–1.1)	0.5 (0.3–1.0)	0.403

LTC, long-term care; BMI, body mass index; SBP, systolic blood pressure; DBP, diastolic blood pressure; TC, total cholesterol; HDLC, high-density lipoprotein cholesterol; non-HDLC, non-high-density lipoprotein cholesterol; Hb, blood hemoglobin; HbA1c, glycosylated hemoglobin; eGFR, estimated glomerular filtration rate; CVD, cardiovascular disease; Cr, creatinine; MI, myocardial infarction; HF, heart failure; UACR, urinary albumin-creatinine ratio; BNP, B-type natriuretic peptide; hsCRP, high-sensitivity C-reactive protein.

*Statistically significant

To determine the additive effect of a biomarker in conjunction with essential risk factors for LTC (i.e. by age and sex) in their overall predictive accuracy on tests, we used net reclassification improvement (NRI) and integrated discrimination improvement (IDI). We calculated the changes in the risk reclassification for the probability of LTC in the base model and the base + UACR model. Tertiles of risk categories with cut-off points of 0.33 and 0.67 were derived from the results of incident risk for LTC adjusted by age and sex.

All-cause deaths and migration was confirmed by the official resident registration data issued by the local government offices (December 2009). All p-values were based on two-sided tests, and p-values < 0.05 were considered statistically significant. The Statistical Package for Social Sciences (SPSS) software program, version 19.0 (IBM, Chicago, IL, USA), or R (version 3.3.1) was used for all analyses.

## Results

The baseline characteristics are compared between the LTC and non-LTC groups in Table 1. The mean age of the LTC group was older than that of the non-LTC group. The percentage of women was significantly higher in the LTC group than in the non-LTC group. Blood hemoglobin and eGFR in the LTC group were significantly lower than in the non-LTC group (p < 0.05). The proportion of few educational years was also significantly higher in the LTC group than in the non-LTC group (p <0.001). There were no significant differences in the smoking status proportions between the two groups. The prevalence of hypertension and diabetes mellitus was significantly higher in the LTC group than in the non-LTC group (both p < 0.001). Furthermore, the prevalence of atrial fibrillation in the LTC group was significantly higher than in the non-LTC group (p < 0.001). The prevalence of interim CVD in the LTC group was significantly higher than in the non-LTC group (17.9% vs. 3.2%, p < 0.001). The median period from the baseline study to the interim CVD was 2.9 years (inter-quartile range, 1.7–4.3 years), and the median period from interim CVD to LTC certification was 0.7 years (inter-quartile range, 0.2–3.2 years). The median UACR and BNP in the LTC group were significantly higher than in the non-LTC group (UACR, p < 0.001; BNP, p < 0.001).

Table 2 shows the hazard ratios for the risk of incidence of interim CVD for each biomarker. In the age-/sex-adjusted model, the hazard ratios increased significantly according to the Qs of the UACR and of BNP (both p for trend < 0.001). These associations remained significant even in the multivariable adjusted model. However, the association was not significant among any Qs of hsCRP.

**Table 2:** Cox regression analyses for the risk of interim CVD for each biomarker.

				Age-/sex-adjusted			Multiple factor-adjusted		
		Number of participants	Number of incidents	HR	95% CI	*p*-values	HR	95% CI	*p*-values
**UACR**	**Q1**	1439	46	1.00			1.00		
	**Q2**	1439	53	1.07	(0.72–1.59)	0.726	1.04	(0.70–1.55)	0.842
	**Q3**	1439	82	1.85	(1.29–2.67)	0.001*	1.70	(1.18–2.45)	0.005*
	**Q4**	1438	106	2.28	(1.61–3.24)	<0.001*	1.91	(1.33–2.74)	<0.001*
					*p* for trend	<0.001*		*p* for trend	<0.001*
**BNP**	**Q1**	1442	54	1.00			1.00		
	**Q2**	1441	42	0.82	(0.55–1.23)	0.332	0.82	(0.55–1.23)	0.327
	**Q3**	1441	63	1.22	(0.85–1.76)	0.285	1.20	(0.83–1.73)	0.327
	**Q4**	1431	128	2.47	(1.78–3.42)	<0.001*	2.16	(1.53–3.06)	<0.001*
					*p* for trend	<0.001*		*p* for trend	<0.001*
**hsCRP**	**Q1**	1384	66	1.00			1.00		
	**Q2**	1330	61	0.93	(0.66–1.32)	0.702	0.90	(0.63–1.28)	0.544
	**Q3**	1654	67	0.81	(0.57–1.13)	0.215	0.73	(0.51–1.04)	0.080
	**Q4**	1387	93	1.33	(0.97–1.83)	0.076	1.11	(0.79–1.55)	0.551
					*p* for trend	0.013*		*p* for trend	0.068

HR, hazard ratio; CI, confidence interval; CVD, cardiovascular disease; UACR, urinary albumin-creatinine ratio; BNP, B-type natriuretic peptide; hsCRP, high-sensitivity C-reactive protein.

Adjusted by age, sex, body mass index, systolic blood pressure, total cholesterol, high-density lipoprotein cholesterol, blood hemoglobin, HabA1c, estimated glomerular filtration rate, duration of education, atrial fibrillation, smoking status and drinking status.

* Statistically significant

**Fig 3:**
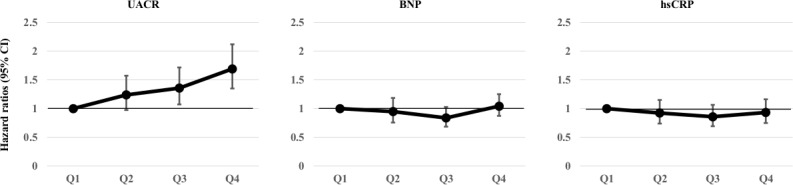
A time-dependent Cox regression analysis for the risk of LTC for each biomarker. Hazard ratios (95% confidence intervals) for the risk of LTC with each biomarker adjusted by age, sex, body mass index, systolic blood pressure, total cholesterol, high-density lipoprotein cholesterol, blood hemoglobin, HbA1c, eGFR, the duration of education, atrial fibrillation, smoking status, drinking status, and interim CVD. The solid line is the hazard ratio, the bar is the 95% confidence interval. The levels of quartiles for each biomarker are shown in Table 1 (see median and IQR).

For LTC, we performed a time-dependent Cox regression analysis to determine the risk of disability with each biomarker. In multivariate-adjusted models including interim CVD, there was a linear relationship between the Qs of the UACR and LTC certification (*p* for trend <0.001). Furthermore, the HR was significantly increased in Q3 and Q4 of the UACR (Q1 = 1.0 [reference], Q2 = 1.24, Q3 = 1.36, Q4 = 1.69) (Fig 3 and S2 Table). However, the hazard ratios of BNP and hsCRP for LTC were not significant for any Qs in either model (Fig 3 and S2 Table). To eliminate possible effects of interim CVD on LTC, we also analyzed a multivariate Cox proportional hazards model to determine the hazard risk of incident LTC among these biomarkers in a model excluding subjects with interim CVD. The association remained statistically significant for the highest Q of UACR (Q1 = 1.0 [reference], Q2 = 1.10, Q3 = 1.26, Q4 = 1.61; p for trend < 0.001) (S3 Table).

Table 3 shows the changes in the risk reclassification for the probability of LTC in the base model (i.e. age- and sex-adjusted) and the base + the UACR model. Adding the UACR to the base model led to a significant improvement in reclassification (NRI = 0.084, p < 0.01; IDI = 0.0018, p < 0.01).

**Table 3:** Change in the risk classification for the probability of needing long-term care using the base model compared with the base + UACR model.

			Base model and UACR			% Net correctly reclassified*
			Low risk (<6%)	Intermediate risk (7%-13%)	High risk (>13%)	
**Non-LTC (n = 5,045)**	**Base model**	**Low risk**	1699	8	0	0.1
		**Intermediate risk**	326	1507	38	
		**High risk**	0	112	1355	
**LTC (n = 710)**	**Base model**	**Low risk**	64	0	0	1.0
		**Intermediate risk**	17	163	8	
		**High risk**	0	18	440	

NRI = 0.084 (0.022–0.147), *p*-value < 0.01

IDI = 0.0018 (0.0001–0.0035), *p*-value < 0.01

Base model: age- and sex-adjusted

*The proportion of net correctly reclassified was shown using the probabilities from the model based on each tertile and the model based on each score adjusted by the UACR.

UACR, urinary albumin-creatinine ratio; NRI, net reclassification improvement; IDI, integrated discrimination improvement; LTC, long-term care.

## Discussion

The present study demonstrated that an increased UACR was associated with the incidence of the certified need for LTC longitudinally in the general population, even after adjusting for established CVD risk factors and interim CVD. There was a linear relationship between the UACR and incident LTC. Furthermore, the additive effect of the UACR in conjunction with essential risk factors significantly improved its predictive ability.

Few studies have shown the relationship between any biomarkers and LTC development. The main reasons for needing LTC among elderly individuals include physical disability and deterioration of the cognitive function [10]. A major cause of physical disability may be CVD, including stroke, heart failure, and myocardial infarction. We therefore initially hypothesized that interim CVD was strongly linked to the incidence of LTC and therefore analyzed the association between three established CVD biomarkers and the incidence of LTC. Accordingly, we found that only the UACR had a significant association with the incidence of LTC.

Although the exact mechanisms underlying the association between the UACR and incident LTC have not been clarified in this study, several previous studies have suggested that an increased UACR is an early marker of diffuse endothelial dysfunction, including glomerular endothelial dysfunction [11–18]. In addition, an elevated UACR is a useful marker for predicting the incidence of CVD [15]. Our previous study found an association between low-grade albuminuria as measured by the UACR and the risk CVD incidence in a community-based sample [9]. These findings suggest that interim CVD has a significant impact on the certification for LTC due to physical disabilities, making it reasonable to assume that subjects with a higher UACR are at risk for incident LTC.

However, interim CVD (stroke, heart failure, and myocardial infarction) was only present in 18% of the LTC group (Table 1), and the observed association between the UACR and LTC was robust, even after the exclusion of subjects with interim CVD (S3 Table). In addition, although the hazard ratio derived from the Cox models for the interim CVD incidence of elevated plasma BNP was comparable to that of the UACR (Table 2), the predictive ability of BNP for incidence of LTC was not significant (Fig 3). These results suggest that additional mechanisms other than the interim CVD hypothesis (interim CVD is the main cause of incident LTC) might be involved in the association between the UACR and LTC.

We assumed that incident LTC in the present study was caused by not only physical impairment but also cognitive dysfunction [19]. Several previous studies have suggested that an elevated UACR is reflected by generalized vascular endothelial dysfunction and microvascular diseases [14–16, 18, 20, 21]. In the brain, microvascular diseases can increase the permeability of the blood-brain barrier, which leads to extravasation of blood substances and leakage of serum proteins [14]. These abnormalities have been suggested to induce subsequent neuronal damage with gray matter atrophy and cortical thinning [11, 14, 16, 18]. Indeed, Sajjad et al. reported that subjects with a higher UACR showed a faster cognitive decline than those with a lower UACR [11]. The UACR may therefore be a common biomarker for predicting CVD events and cognitive decline, possibly due to a systemic disorder of the microvasculature, including the cerebral arteries.

**Fig 4:**
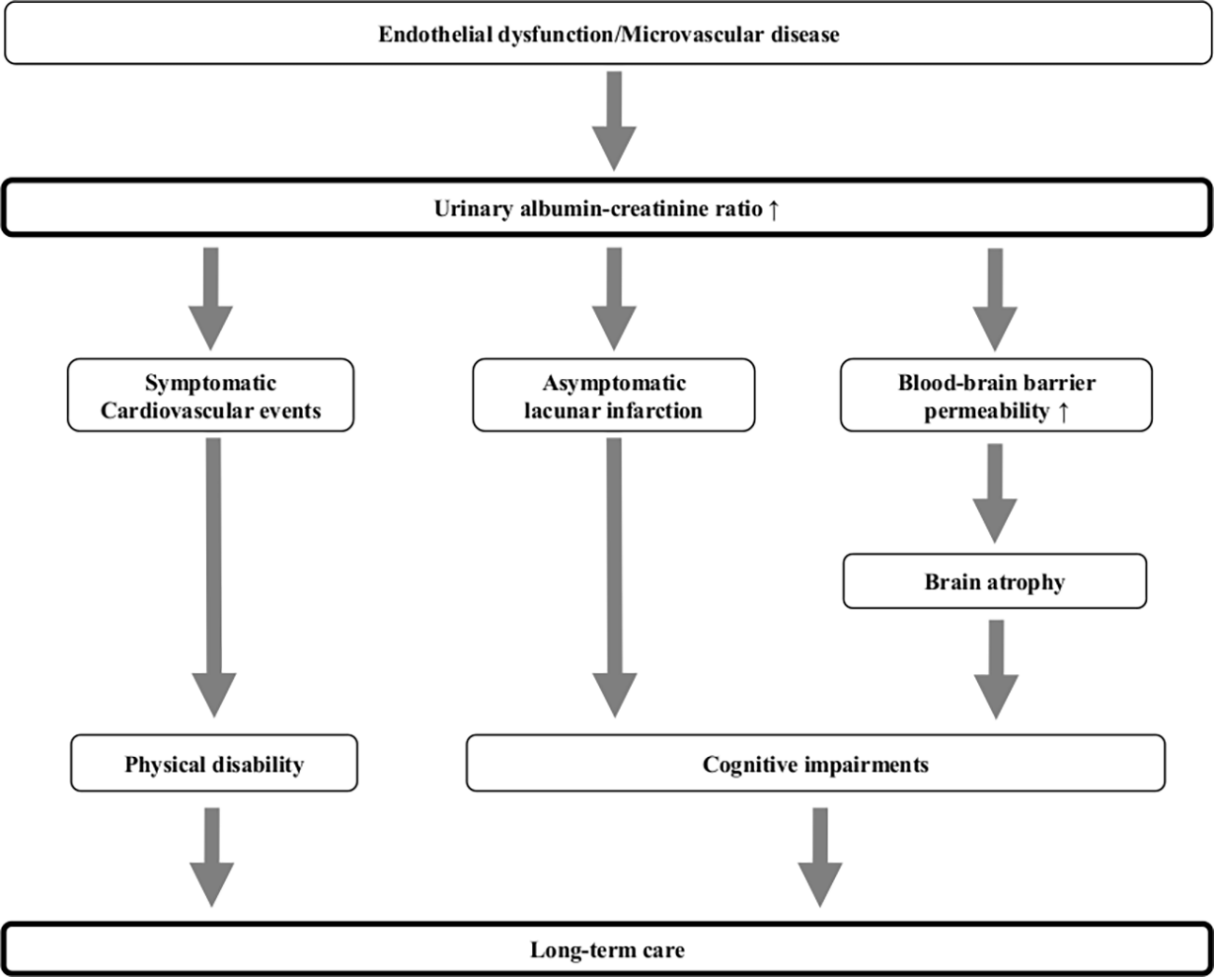
Possible pathophysiologic links between the urinary albumin-creatinine ratio and incident long-term care.

Moreover, previous reports have shown an increased UACR to be a risk factor for lacunar infarction in the general population [22, 23] and suggested that patients with lacunar infarction show a stepwise decline in their cognitive function over long-term follow-up [24, 25]. Given these previous findings, the present LTC subjects with elevated UACR levels might show mild dementia due to lacunar infarction without any apparent focal symptoms or physical disabilities. These mechanisms might explain the association observed between incident LTC and UACR (Fig 4).

Several studies have shown that a higher BNP concentration is significantly associated with the incidence of stroke and heart failure. In addition, the BNP concentration is affected by the renal function, as reflected by parameters such as creatinine clearance, the eGFR and the serum creatinine concentration. On the other hand, patients who suffer from stroke and heart failure tend to have physical impairment, such as hemiparesis and difficulty working with dyspnea on exertion. Thus, we initially hypothesized that BNP would be significantly associated with LTC. However, the results showed that the BNP concentration had no relationship with LTC, even after adjustment for major confounding factors, including renal function parameters (S4A, S4B and S4C Table). The reasons for this negative association between the BNP concentration and LTC are unclear at present. However, we hypothesize that LTC might have a weak relationship with the physical disability caused by CVD, but a strong relationship with cognitive decline and lacunar infarction without any apparent focal symptoms. Another reason is that the prevalence of interim CVD among the subjects in LTC was only 18%. This small parentage of patients with stroke and heart failure might explain the lack of a significant relationship between the BNP concentration and LTC. Although an elevated blood level of hsCRP is a well-known factor for predicting CVD, this biomarker did not have a significant predictive value for future LTC in this study. Some studies have reported that hsCRP is a sensitive marker of physical disability with a good prognostic value for future cognitive dysfunction [26–28]. However, our study did not show any association between the serum hsCRP levels and incident LTC. Although the precise reasons for this negligible relationship are unclear based on our present findings, one of the reasons may be due to the lower levels of serum hsCRP in the Japanese population than in other ethnicities [29]. Indeed, the present study did not show the hsCRP to be an efficient predictor of the incidence of interim CVD (Table 2).

### Limitations

The present study is associated with several limitations. First, we were unable to evaluate the causes of disability (e.g. physical impairment and cognitive dysfunction). However, as the present study has shown that an elevated UACR might be related to both types of causes of disability (physical and cognitive; S2 Table and S3 Table), our main results might be valid. Second, BNP did not have any relationship with LTC after adjustment for several confounding factors. The prevalence of interim CVD might have been low during the study period. In addition to CVD, LTC might have a relationship with cognitive decline and lacunar infarction without focal symptoms. Third, the urinary albumin test was conducted only once, which might have led to an underestimation of the true albuminuria-related risk. Fourth, although the present study excluded obviously febrile subjects, some urine samples might have been contaminated by bacteria, which may have led to misclassification by UACR Qs. Fifth, the generalizability of these results is unclear, as the percentage of LTC was lower in our study than in other epidemiological studies [30, 31]. This might be due to the relatively short follow-up duration of this study. Finally, our subjects proactively underwent a health checkup, suggesting they may be healthier or more health-conscious than non-participants. This possibility might have led to underestimation in the present results.

## Conclusion

To prevent LTC within a large elderly population, it may be important to establish simple and objective means of identifying subjects at risk of needing LTC. The present results suggest that an increased UACR might be useful for assessing such a risk and helpful for predicting the incidence of disabilities in the elderly general population. Further studies to explore types of interventions and their cost-effectiveness are needed to reduce the incidence of LTC.

## Supporting information

S1 Table Questionnaire used in the present study (Japanese and English). (DOCX)

S2 Table A time-dependent Cox regression analyses for the risk of incidence of LTC for each biomarker (including interim CVD: n = 5,755). (DOCX)

S3 Table Cox regression analyses for the risk of incidence of LTC for each biomarker (excluding interim CVD: n = 5,468). (DOCX)

S4 Table A. A Cox regression analysis of the risk of interim CVD according to the BNP concentration adjusted for the kidney function (n = 5,755).
B. A time-dependent Cox regression analysis of the risk of LTC according to BNP concentration adjusted for the kidney function (including interim CVD: n = 5,755).
C. A Cox regression analysis of the risk of LTC according to BNP concentration adjusted for the kidney function (excluding interim CVD: n = 5,468).
(ZIP)

S5 Table A time-dependent Cox regression analysis for the risk of incidence of LTC for each biomarker, including subjects who had a history of stroke, myocardial infarction or heart failure (n = 6,055). (DOCX)

S6 Table Data in the present study. (ZIP)
