# Supplementary material for: The urine albumin-creatinine ratio is a predictor for incident long-term care in a general population
Source: PLoS One. 2018 Mar 28;13(3):e0195013. doi: 10.1371/journal.pone.0195013 (PMC5874057; doi:10.1371/journal.pone.0195013)
Supplement: S1 Table — (DOCX) [file pone.0195013.s001.docx]

**S1 Table. Questionnaire used in the present study (Japanese and English)**

This table shows the questionnaire, “The urine albumin-creatinine ratio is a predictor for incident long-term care in a general population”. The original questionnaire was drafted in Japanese.

**質問紙 （日本語, Japanese）**

1. 既往歴

下記の病名の中で、今までにかかった病気の有無について左の「あり」「なし」内に✔印を記入してください。「あり」の場合は、既往歴記入欄に病名番号と現在の状況を記入してください。

| 循環器 | 1 | 高血圧症 | 呼吸器 | 43 | 肺結核 |
| --- | --- | --- | --- | --- | --- |
|  | 2 | 狭心症 |  | 44 | 胸膜炎 |
|  | 3 | 心筋梗塞 |  | 45 | 肺炎 |
|  | 4 | 無痛性虚血性心疾患 |  | 46 | 気管支喘息 |
|  | 5 | 心筋症 |  | 47 | 慢性気管支炎 |
|  | 6 | 弁膜症 |  | 48 | 気管支拡張症 |
|  | 7 | 先天性心疾患 |  | 49 | 慢性肺気腫 |
|  | 8 | 不整脈 |  | 50 | 肺線維症 |
|  | 9 | 脳内出血 |  | 51 | じん肺 |
|  | 10 | くも膜下出血 |  | 52 | 肺梗塞 |
|  | 11 | 脳梗塞 |  | 53 | 肺のう胞 |
|  | 12 | 一過性脳虚血発作 |  | 54 | 自然気胸 |
|  | 13 | 大動脈瘤 | その他 | 55 | 緑内障 |
|  | 14 | 脳動脈瘤 |  | 56 | 聴力障害 |
| 消化管 | 15 | 食道疾患 |  | 57 | 貧血 |
|  | 16 | 胃潰瘍 |  | 58 | 膠原病 |
|  | 17 | 十二指腸潰瘍 |  | 59 | 慢性関節リウマチ |
|  | 18 | 慢性胃炎 |  | 60 | 骨粗鬆症 |
|  | 19 | 胃ポリープ |  | 61 | 神経・筋疾患 |
|  | 20 | 大腸ポリープ |  | 62 | 脳外科疾患 |
|  | 21 | 潰瘍性大腸炎 |  | 63 | 精神疾患 |
|  | 22 | クローン病 |  | 64 | 甲状腺疾患 |
|  | 23 | 腸閉塞 |  | 65 | 乳房疾患 |
|  | 24 | 痔疾患 |  | 66 | 婦人科疾患 |
| 泌尿器 | 25 | 尿路結石 |  | 67 | 肋骨・鎖骨骨折 |
|  | 26 | 腎嚢胞 | 1-67以外の疾患 | 71 | がん疾患（64~66は重複しないように） |
|  | 27 | 慢性腎炎（慢性腎不全を含む） |  | 72 | 内科疾患 |
|  | 28 | ネフローゼ症候群 |  | 73 | 心療内科疾患 |
|  | 29 | 前立腺肥大症 |  | 74 | 外科疾患 |
| 代謝 | 30 | 糖尿病 |  | 75 | 整形外科疾患 |
|  | 31 | 高脂血症 |  | 76 | 泌尿器科疾患 |
|  | 32 | 痛風（高尿酸血症を含む） |  | 77 | 眼科疾患 |
| 肝胆膵 | 33 | 肝のう胞 |  | 78 | 耳鼻科疾患 |
|  | 34 | 肝血管腫 |  | 79 | 皮膚科疾患 |
|  | 35 | 肝障害 |  | | |
|  | 36 | 脂肪肝 |  |  |  |
|  | 37 | 慢性肝炎 |  |  |  |
|  | 38 | 肝硬変 |  |  |  |
|  | 39 | 胆石症 |  |  |  |
|  | 40 | 胆のう炎 |  |  |  |
|  | 41 | 胆のうポリープ |  |  |  |
|  | 42 | 慢性膵炎 |  |  |  |

病名番号（現在の状況）

治療中・観察中・放置・手術・済

2. 嗜好 当てはまる項目の番号を◯で囲んでください。

①アルコールを飲みますか？

1. 以前から（ほとんど）飲まない
2. 以前は飲んでいたが今は（ほとんど）飲まない
3. 飲むことも有るが毎週1回未満
4. 飲む

②タバコを吸いますか？

1. もともと吸わない
2. 辞めた
3. 吸う

3. 小学校を含め何年間学校に通いましたか（各種学校を含みます）。

年

**Questionnaire (English)**

1. Medical history

Please respond yes or no regarding your medical history of the items below. If you answer yes, please describe the number of disease and indicate your current condition from the four items.

| Cardiology | 1 | Hypertension | Pulmonology | 43 | Pulmonary tuberculosis |
| --- | --- | --- | --- | --- | --- |
|  | 2 | Angina pectoris |  | 44 | Pleurisy |
|  | 3 | Myocardial infarction |  | 45 | Pneumonia |
|  | 4 | Silent myocardial ischemia |  | 46 | Asthma |
|  | 5 | Myocarditis |  | 47 | Chronic bronchitis |
|  | 6 | Valvular heart disease |  | 48 | Bronchiectasis |
|  | 7 | Congenital heart disease |  | 49 | Chronic obstructive pulmonary disease |
|  | 8 | Arrhythmia |  | 50 | Pulmonary fibrosis |
|  | 9 | Intracranial hemorrhage |  | 51 | Pneumoconiosis |
|  | 10 | Subarachnoid hemorrhage |  | 52 | Pulmonary infarction |
|  | 11 | Cerebral infarction |  | 53 | Pulmonary cyst |
|  | 12 | Transient ischemic attack |  | 54 | Spontaneous pneumothorax |
|  | 13 | Aortic aneurysm | Others | 55 | Glaucoma |
|  | 14 | Cerebral arterial aneurysm |  | 56 | Hearing disorder |
| Gastroenterology | 15 | Esophageal disease |  | 57 | Anemia |
|  | 16 | Gastric ulcer |  | 58 | Collagen disease |
|  | 17 | Duodenum ulcer |  | 59 | Rheumatoid arthritis |
|  | 18 | Chronic gastritis |  | 60 | Osteoporosis |
|  | 19 | Gastric polyp |  | 61 | Neuromuscular disorder |
|  | 20 | Colon polyp |  | 62 | Neurosurgical disease |
|  | 21 | Ulcerative colitis |  | 63 | Mental disease |
|  | 22 | Crohn disease |  | 64 | Thyroid disease |
|  | 23 | Intestinal obstruction |  | 65 | Breast disease |
|  | 24 | Hemorrhoid |  | 66 | Gynecologic disorder |
| Urology | 25 | Urolithiasis |  | 67 | Ribs or clavicle bone fracture |
|  | 26 | Renal cyst | Other diseases not mentioned in No. 1 to No. 67 | 71 | Cancer (without duplicating No. 64 to No. 66) |
|  | 27 | Chronic nephritis, including chronic kidney disease |  | 72 | Internal medical disorder |
|  | 28 | Nephrotic syndrome |  | 73 | Psychosomatic disease |
|  | 29 | Prostatic hyperplasia |  | 74 | Surgical disease |
| Metabolic disease | 30 | Diabetes mellitus |  | 75 | Orthopedics disease |
|  | 31 | Dyslipidemia |  | 76 | Urological disorder |
|  | 32 | Gout, including hyperuricemia |  | 77 | Ophthalmological disease |
| Hepato-pancreato-biliary | 33 | Liver cyst |  | 78 | Otolaryngological disease |
|  | 34 | Hemangioma |  | 79 | Dermatological disease |
|  | 35 | Liver disease |  | | |
|  | 36 | Fatty liver |  |  |  |
|  | 37 | Chronic hepatitis |  |  |  |
|  | 38 | Liver cirrhosis |  |  |  |
|  | 39 | Cholelithiasis |  |  |  |
|  | 40 | Cholecystitis |  |  |  |
|  | 41 | Gallbladder polyp |  |  |  |
|  | 42 | Chronic pancreatitis |  |  |  |

The number of current conditions

Treatment with prescribed drugs

Left neglected without treatment

Observation

Surgical operation

Finished treatment

2. Alcohol consumption and smoking.

Please circle the number of the item that best fits your situation in the questions below.

①Do you drink alcohol?

1. No.
2. Not now, but I drank daily in the past.
3. I drink occasionally.
4. I drink daily.

②Do you smoke?

1. No.
2. I have quit smoking.
3. Yes.

3. How many years have you gone to school, including elementary school and vocational school?

_____ years
