# Supplementary material for: The urine albumin-creatinine ratio is a predictor for incident long-term care in a general population
Source: PLoS One. 2018 Mar 28;13(3):e0195013. doi: 10.1371/journal.pone.0195013 (PMC5874057; doi:10.1371/journal.pone.0195013)
Supplement: S2 Table — (DOCX) [file pone.0195013.s002.docx]

| **S2 Table. A time-dependent Cox regression analyses for the risk of incidence of LTC for each biomarker (including interim CVD: n = 5,755).** | | | | | | | | | | |
| --- | --- | --- | --- | --- | --- | --- | --- | --- | --- | --- |
|  | |  |  |  | **Age/sex-adjusted** | | | **Multiple factor adjusted** | | |
|  | |  | **Number of participants** | **Number of incidents** | **HR** | **95% CI** | ***p*-values** | **HR** | **95% CI** | ***p*-values** |
| **UACR** | | **Q1** | 1439 | 120 | 1.00 |  |  | 1.00 |  |  |
|  | | **Q2** | 1439 | 155 | 1.21 | ( 0.95 - 1.54 ) | 0.116 | 1.24 | ( 0.98 - 1.57 ) | 0.08 |
|  | | **Q3** | 1439 | 187 | 1.35 | ( 1.07 - 1.71 ) | 0.010* | 1.36 | ( 1.07 - 1.72 ) | 0.010* |
|  | | **Q4** | 1438 | 248 | 1.66 | ( 1.33 - 2.06 ) | <0.001* | 1.69 | ( 1.35 - 2.12 ) | <0.001* |
|  | |  |  |  |  | *p* for trend | <0.001* |  | *p* for trend | <0.001* |
| **BNP** | | **Q1** | 1442 | 145 | 1.00 |  |  | 1.00 |  |  |
|  | | **Q2** | 1441 | 147 | 0.96 | ( 0.77 - 1.21 ) | 0.751 | 0.95 | ( 0.75 - 1.20 ) | 0.653 |
|  | | **Q3** | 1441 | 163 | 0.87 | ( 0.69 - 1.09 ) | 0.228 | 0.84 | ( 0.67 - 1.05 ) | 0.130 |
|  | | **Q4** | 1431 | 255 | 1.11 | ( 0.90 - 1.37 ) | 0.333 | 1.04 | ( 0.84 - 1.30 ) | 0.709 |
|  | |  |  |  |  | *p* for trend | 0.111 |  | *p* for trend | 0.183 |
| **hsCRP** | | **Q1** | 1384 | 175 | 1.00 |  |  | 1.00 |  |  |
|  | | **Q2** | 1330 | 152 | 0.91 | ( 0.73 - 1.13 ) | 0.375 | 0.92 | ( 0.74 - 1.15 ) | 0.477 |
|  | | **Q3** | 1654 | 193 | 0.86 | ( 0.70 - 1.06 ) | 0.151 | 0.86 | ( 0.69 - 1.07 ) | 0.165 |
|  | | **Q4** | 1387 | 190 | 0.94 | ( 0.77 - 1.16 ) | 0.581 | 0.93 | ( 0.75 - 1.16 ) | 0.537 |
|  |  | |  |  |  | *p* for trend | 0.531 |  | *p* for trend | 0.576 |
| HR, hazard ratio; CI, confidence interval; LTC, long-term care; CVD, cardiovascular disease; UACR, urinary albumin-creatinine ratio; BNP, B-type natriuretic peptide; hsCRP, high-sensitivity C-reactive protein. | | | | | | | | | | |
| Adjusted for the age, sex, body mass index, systolic blood pressure, total cholesterol, high-density lipoprotein cholesterol, blood hemoglobin, HabA1c, estimated glomerular filtration rate, duration of education, atrial fibrillation, smoking status, drinking status and interim CVD. | | | | | | | | | | |
| *** Statistically significant | | | | | | | | | | |
