# Supplementary material for: The urine albumin-creatinine ratio is a predictor for incident long-term care in a general population
Source: PLoS One. 2018 Mar 28;13(3):e0195013. doi: 10.1371/journal.pone.0195013 (PMC5874057; doi:10.1371/journal.pone.0195013)
Supplement: S3 Table — (DOCX) [file pone.0195013.s003.docx]

| **S3 Table. Cox regression analyses for the risk of incidence of LTC for each biomarker (excluding interim CVD: n=5,468).** | | | | | | |
| --- | --- | --- | --- | --- | --- | --- |
|  |  | **Number of participants** | **Number of incidents** | **HR** | **95% CI** | ***p*-values** |
| **UACR** | **Q1** | 1393 | 102 | 1.00 |  |  |
|  | **Q2** | 1386 | 129 | 1.10 | ( 0.85 - 1.43 ) | 0.480 |
|  | **Q3** | 1357 | 152 | 1.26 | ( 0.98 - 1.62 ) | 0.077 |
|  | **Q4** | 1332 | 200 | 1.61 | ( 1.25 - 2.06 ) | <0.001* |
|  |  |  |  |  | *p* for trend | <0.001* |
| **BNP** | **Q1** | 1388 | 118 | 1.00 |  |  |
|  | **Q2** | 1399 | 127 | 1.05 | ( 0.82 - 1.35 ) | 0.713 |
|  | **Q3** | 1378 | 140 | 0.97 | ( 0.76 - 1.25 ) | 0.813 |
|  | **Q4** | 1303 | 198 | 1.22 | ( 0.96 - 1.56 ) | 0.103 |
|  |  |  |  |  | *p* for trend | 0.177 |
| **hsCRP** | **Q1** | 1318 | 143 | 1.00 |  |  |
|  | **Q2** | 1269 | 120 | 0.85 | ( 0.67 - 1.09 ) | 0.207 |
|  | **Q3** | 1587 | 175 | 0.93 | ( 0.71 - 1.14 ) | 0.374 |
|  | **Q4** | 1294 | 145 | 0.87 | ( 0.69 - 1.13 ) | 0.310 |
|  |  |  |  |  | *p* for trend | 0.613 |
| HR, hazard ratio; CI, confidence interval; CVD, cardiovascular disease; UACR, urinary albumin-creatinine ratio; BNP, B-type natriuretic peptide; hsCRP, high-sensitivity C-reactive protein.  Adjusted for the age, sex, body mass index, systolic blood pressure, total cholesterol, high-density lipoprotein cholesterol, blood hemoglobin, HabA1c, estimated glomerular filtration rate, duration of education, atrial fibrillation, smoking status and drinking status. | | | | | | |
| * Statistically significant | | | | | | |
