# Supplementary material for: The urine albumin-creatinine ratio is a predictor for incident long-term care in a general population
Source: PLoS One. 2018 Mar 28;13(3):e0195013. doi: 10.1371/journal.pone.0195013 (PMC5874057; doi:10.1371/journal.pone.0195013)
Supplement: S4 Table — A. A Cox regression analysis of the risk of interim CVD according to the BNP concentration adjusted for the kidney function (n = 5,755). B. A time-dependent Cox regression analysis of the risk of LTC according to BNP concentration adjusted for the kidney function (including interim CVD: n = 5,755). C. A Cox regression analysis of the risk of LTC according to BNP concentration adjusted for the kidney function (excluding interim CVD: n = 5,468). (ZIP) [file pone.0195013.s004.zip › S4_Table/20180321 S4A_Table.docx]

**S4A Table. A Cox regression analysis of the risk of interim CVD according to the BNP concentration adjusted for the kidney function (n = 5,755).**

|  | **BNP** | **HR** | **95% CI** | ***p*-values** |
| --- | --- | --- | --- | --- |
| **Model 1** | **Q1** | 1.00 |  |  |
|  | **Q2** | 0.82 | (0.55 - 1.23) | 0.330 |
|  | **Q3** | 1.20 | (0.83 - 1.74) | 0.325 |
|  | **Q4** | 2.20 | (1.56 - 3.11) | <0.001* |
|  |  |  | *p for trend* | <0.001* |
| **Model 2** | **Q1** | 1.00 |  |  |
|  | **Q2** | 0.82 | (0.55 - 1.23) | 0.332 |
|  | **Q3** | 1.22 | (0.85 - 1.76) | 0.285 |
|  | **Q4** | 2.47 | (1.78 - 3.42) | <0.001* |
|  |  |  | *p for trend* | <0.001* |
| **Model 3** | **Q1** | 1.00 |  |  |
|  | **Q2** | 0.82 | (0.55- 1.23) | 0.33 |
|  | **Q3** | 1.21 | (0.83 - 1.74) | 0.322 |
|  | **Q4** | 2.20 | (1.55 - 3.11) | <0.001* |
|  |  |  | *p for trend* | <0.001 |

Basic model: adjusted by age, sex, body mass index, systolic blood pressure, total cholesterol, high-density lipoprotein cholesterol, blood hemoglobin, HabA1c, duration of education, atrial fibrillation, smoking status, drinking status.

Model 1: adjusted basic model + creatinine clearance (Cockcroft-Gault formula).

Model 2: adjusted basic model + estimated glomerular filtration rate.

Model 3: adjusted basic model + serum creatinine level.

Abbreviations: HR, hazard ratio; CI, confidence interval; BUN, B-type natriuretic peptide; CVD, cardiovascular disease.

Serum creatinine was logarithmically transformed to improve normality prior to analyses.

* Statistically significant
