# Supplementary material for: The urine albumin-creatinine ratio is a predictor for incident long-term care in a general population
Source: PLoS One. 2018 Mar 28;13(3):e0195013. doi: 10.1371/journal.pone.0195013 (PMC5874057; doi:10.1371/journal.pone.0195013)
Supplement: S4 Table — A. A Cox regression analysis of the risk of interim CVD according to the BNP concentration adjusted for the kidney function (n = 5,755). B. A time-dependent Cox regression analysis of the risk of LTC according to BNP concentration adjusted for the kidney function (including interim CVD: n = 5,755). C. A Cox regression analysis of the risk of LTC according to BNP concentration adjusted for the kidney function (excluding interim CVD: n = 5,468). (ZIP) [file pone.0195013.s004.zip › S4_Table/20180321 S4B_Table.docx]

**S4B Table. A time-dependent Cox regression analysis of the risk of LTC according to BNP concentration adjusted for the kidney function (including interim CVD: n = 5,755).**

|  | **BNP** | **HR** | **95% CI** | ***p*-values** |
| --- | --- | --- | --- | --- |
| **Model 1** | **Q1** | 1.00 |  |  |
|  | **Q2** | 0.95 | (0.75 - 1.19) | 0.638 |
|  | **Q3** | 0.83 | (0.66 - 1.05) | 0.121 |
|  | **Q4** | 1.04 | (0.84 - 1.30) | 0.705 |
|  |  |  | *p for trend* | 0.165 |
| **Model 2** | **Q1** | 1.00 |  |  |
|  | **Q2** | 0.95 | (0.75 - 1.20) | 0.653 |
|  | **Q3** | 0.84 | (0.67 - 1.05) | 0.130 |
|  | **Q4** | 1.04 | (0.84 - 1.30) | 0.709 |
|  |  |  | *p for trend* | 0.183 |
| **Model 3** | **Q1** | 1.00 |  |  |
|  | **Q2** | 0.95 | (0.76 - 1.20) | 0.677 |
|  | **Q3** | 0.84 | (0.67 - 1.06) | 0.134 |
|  | **Q4** | 1.05 | (0.85 - 1.31) | 0.645 |
|  |  |  | *p for trend* | 0.161 |

Basic model: adjusted by age, sex, body mass index, systolic blood pressure, total cholesterol, high-density lipoprotein cholesterol, blood hemoglobin, HabA1c, duration of education, atrial fibrillation, smoking status, drinking status.

Model 1: adjusted basic model + creatinine clearance (Cockcroft-Gault formula).

Model 2: adjusted basic model + estimated glomerular filtration rate.

Model 3: adjusted basic model + serum creatinine level.

Serum creatinine was logarithmically transformed to improve normality prior to analyses.

Abbreviations: HR, hazard ratio; CI, confidence interval; BUN, B-type natriuretic peptide; CVD, cardiovascular disease.

* Statistically significant
