# Supplementary material for: The urine albumin-creatinine ratio is a predictor for incident long-term care in a general population
Source: PLoS One. 2018 Mar 28;13(3):e0195013. doi: 10.1371/journal.pone.0195013 (PMC5874057; doi:10.1371/journal.pone.0195013)
Supplement: S5 Table — (DOCX) [file pone.0195013.s005.docx]

| **S5 Table. A time-dependent Cox regression analysis for the risk of incidence of LTC for each biomarker, including subjects who had a history of stroke, myocardial infarction or heart failure (n=6,055).** | | | | | | |
| --- | --- | --- | --- | --- | --- | --- |
|  |  | **Number of participants** | **Number of incidents** | **HR** | **95% CI** | ***p*-values** |
| **UACR** | **Q1** | 1506 | 132 | 1.00 |  |  |
|  | **Q2** | 1516 | 178 | 1.29 | (1.03-1.62) | 0.027* |
|  | **Q3** | 1502 | 203 | 1.36 | (1.09-1.70) | 0.007* |
|  | **Q4** | 1531 | 277 | 1.69 | (1.36-2.09) | <0.001* |
|  |  |  |  |  | *p* for trend | <0.001* |
| **BNP** | **Q1** | 1516 | 163 | 1.00 |  |  |
|  | **Q2** | 1496 | 158 | 0.91 | (0.73-1.14) | 0.415 |
|  | **Q3** | 1499 | 184 | 0.87 | (0.70-1.07) | 0.191 |
|  | **Q4** | 1544 | 285 | 1.01 | (0.82-1.24) | 0.932 |
|  |  |  |  |  | *p* for trend | 0.366 |
| **hsCRP** | **Q1** | 1446 | 185 | 1.00 |  |  |
|  | **Q2** | 1386 | 164 | 0.94 | (0.76-1.16) | 0.537 |
|  | **Q3** | 1751 | 229 | 0.95 | (0.77-1.17) | 0.62 |
|  | **Q4** | 1472 | 212 | 0.97 | (0.78-1.19) | 0.739 |
|  |  |  |  |  | *p* for trend | 0.934 |
| HR, hazard ratio; CI, confidence interval; CVD, cardiovascular disease; UACR, urinary albumin-creatinine ratio; BNP, B-type natriuretic peptide; hsCRP, high-sensitivity C-reactive protein.  Adjusted for the age, sex, body mass index, systolic blood pressure, total cholesterol, high-density lipoprotein cholesterol, blood hemoglobin, HabA1c, estimated glomerular filtration rate, duration of education, atrial fibrillation, smoking status and drinking status. | | | | | | |
| * Statistically significant | | | | | | |
