# Supplementary material for: The urine albumin-creatinine ratio is a predictor for incident long-term care in a general population
Source: PLoS One. 2018 Mar 28;13(3):e0195013. doi: 10.1371/journal.pone.0195013 (PMC5874057; doi:10.1371/journal.pone.0195013)
Supplement: S6 Table — (ZIP) [file pone.0195013.s006.zip › S6 Table/S6 Table.pdf]

filter_420_n2	flag_kai_s	mchkai_n	p_interum	p_interum_i	sex	age	bmi	sbp	dbp	tc	hdlc	nonHDLC	hb	Hba1c_N	egfr_epi	Q_school_t	TBC_SITU_c	ALC_SITU_c	t1htt_in	t1lipid_o	dm_ni	af	u_alb_ix	h_crp	bnp
1	0	0	100	4.68	1	70	19.5	124	76.5	128	21	107	15.6	4.9	70.1	2	0	0	1	0	1	1	72.3	0.16	24.2
1	0	0	100	7.23	1	65	31.3	118.5	71.5	170	23	147	10.8	6	11.0	2	0	1	0	0	1	1	3003.7	0.33	262
1	0	0	100	1.5	1	74	23.6	157	87.5	131	24	107	11.2	5.2	13.7	2	0	0	0	0	1	1	210.6	0.12	9.2
1	0	0	100	4.63	1	78	26.4	137.5	70	137	25	112	10.4	5.3	33.1	2	0	1	1	0	1	1	95.2	0.03	70.3
1	1	1	1.9	1.9	2	73	21.9	140	85.5	175	25	150	11.4	5.3	73.8	2	1	1	0	0	1	1	18.4	0.01	27.7
1	0	0	100	5.16	1	66	26	139	78.5	212	26	186	15.2	5.7	50.9	2	1	1	1	0	1	1	6.2	0.1	48.2
1	0	0	100	7.06	1	68	28.9	145	83	244	26	218	15.3	5.3	74.6	2	1	0	0	0	1	1	13.2	0.03	14.4
1	0	0	100	6.98	1	70	19.6	134.5	90.5	75	26	49	13.9	6.3	82.8	1	0	0	0	0	1	1	26.3	0.09	1.85
1	0	0	100	4.58	1	71	28	112.5	73	175	27	148	15.9	5.2	73.1	2	1	1	0	0	1	1	12.4	0.03	4
1	0	0	100	5.15	1	67	22.8	194	106	191	27	164	14.6	5.3	79.4	2	0	0	0	0	1	1	32	0.04	19.4
1	0	0	100	5.05	2	65	27.3	124	73	171	27	144	13.4	5.6	78.0	2	1	1	0	0	1	1	13.4	0.02	21.9
1	0	1	2.5	2.5	1	67	28.6	164.5	93.5	139	27	112	15.3	5.8	71.6	2	1	1	0	0	1	1	13	0.06	8.36
1	0	1	1.1	1.1	1	80	25.8	123.5	71.5	185	27	158	15	6.3	51.3	2	1	1	1	0	1	1	15.5	0.2	36.7
1	0	0	100	6.98	1	70	26.3	156	74.5	202	28	174	16.2	6.4	73.6	1	1	0	0	0	1	1	1818.2	3.68	21.1
1	0	0	100	6.86	1	67	24.8	110.5	63	150	28	122	13.9	7.1	79.4	2	0	1	1	0	0	1	9.4	0.02	32
1	0	0	100	4.64	1	69	25.6	105.5	66.5	155	28	127	15.3	5.1	70.6	2	1	1	0	0	1	1	4.5	0.05	5.22
1	1	0	100	6.87	1	79	26.3	168.5	83.5	197	28	169	15	5.2	46.5	2	1	1	0	0	1	1	42.3	0.31	43.7
1	0	0	100	4.63	2	79	23.7	124	81	172	28	144	11.4	5.5	57.2	2	1	1	0	0	1	1	39.5	0.13	44.4
1	0	0	100	5.37	2	74	22.8	130.5	81	193	28	165	12.5	5.4	69.6	2	1	0	0	0	1	1	8.1	0.04	6.81
1	0	0	100	5.37	1	78	23.8	102.5	61	161	28	133	15.2	6.5	78.3	2	0	1	1	0	0	1	7.3	0.02	13
1	0	0	100	5.37	1	83	24.9	111.5	67.5	146	28	118	15	6.4	50.2	2	1	1	1	0	1	1	122.3	0.13	19
1	1	1	3.3	3.3	1	71	21.9	147	81	187	28	159	14.5	5	54.6	2	0	1	0	0	1	1	19.5	0.12	29.3
1	0	0	100	6.86	1	72	18.8	153.5	90.5	141	29	112	15.4	5.3	54.2	2	0	0	0	0	1	1	19.6	0.14	31.8
1	0	0	100	5.05	1	75	26.5	121.5	83	159	29	130	10.2	5.4	43.4	2	1	1	1	0	1	1	25.2	1.51	46.6
1	0	0	100	6.8	1	69	27.5	121.5	80	166	29	137	16.9	5.4	70.6	2	0	1	1	0	1	1	6.9	0.03	1.86
1	0	0	100	5.07	1	74	26.6	111	67	132	29	103	12.9	5.4	71.5	2	1	0	1	0	1	1	23.5	0.09	78.9
1	0	0	100	6.45	1	77	26.1	156	86.5	185	29	156	16.2	5.1	66.7	2	1	1	0	0	1	1	39.6	0.03	18.2
1	1	0	100	5.58	1	65	24	98.5	60	197	29	168	16.2	5.1	72.6	2	0	1	1	0	1	1	5.2	0.05	21.9
1	0	0	100	4.58	2	72	21.4	120.5	63.5	153	29	124	13.4	5.2	78.9	2	1	1	1	0	1	1	29.3	0.05	14.2
1	0	0	100	5.06	2	69	25.3	138	80	145	29	116	12.5	5	75.9	1	1	1	0	0	1	1	9.1	0.06	8.2
1	1	0	100	6.87	1	66	28.5	130	76	266	29	237	15.6	6.3	75.7	2	0	1	1	0	0	1	12.5	0.7	33
1	0	0	100	6.87	2	76	27.5	137.5	81.5	242	29	213	13	5.3	68.6	2	1	1	1	0	1	1	20.9	0.3	11.5
1	0	1	6.1	6.1	1	70	23.4	107.5	62.5	154	29	125	12.8	6.1	44.9	2	1	1	0	0	1	1	298.7	0.65	0.05
1	0	1	5.4	5.4	1	75	25.2	159	79.5	182	29	153	14.8	5.6	53.1	2	1	1	0	0	1	1	11.5	0.03	19.8
1	1	1	4.1	4.1	2	66	23.8	125	78	166	29	137	12.7	6	77.5	2	1	1	1	0	1	1	20.2	1.82	66.1
1	0	0	100	6.88	1	68	27	107.5	66	223	30	193	15.1	5.7	71.1	2	0	1	1	0	1	1	10.7	0.5	26.7
1	0	0	100	6.96	1	67	26.5	163	92	130	30	100	11.3	4.4	79.4	2	1	0	0	0	1	1	83.4	8.58	138
1	0	0	100	4.58	1	79	24.7	120	78	153	30	123	13.2	5.4	51.6	2	1	1	1	0	1	1	3.4	0.05	71.1
1	0	0	100	5.15	1	66	23	121.5	81	179	30	149	14.8	5.7	79.9	2	1	0	1	0	1	1	22.9	0.01	6.95
1	0	0	100	6.84	1	68	25.2	147.5	83.5	231	30	201	14.4	5.8	74.6	2	1	1	0	0	1	1	8.2	0.07	3.33
1	0	0	100	4.58	2	73	26.9	161	81	221	30	191	13.5	5.4	70.1	2	1	1	0	0	1	1	18.2	0.17	11.4
1	0	0	100	4.64	2	66	28.6	121.5	70	149	30	119	13	5.3	77.5	2	1	1	0	0	1	1	13	0.3	31.9
1	0	0	100	5.37	2	67	24.9	134.5	77.5	146	30	116	11.3	6	73.1	2	1	1	1	0	1	1	11.9	0.48	163
1	0	0	100	6.87	1	84	23.5	131.5	76	141	30	111	14.2	5.5	70.5	2	1	1	1	0	1	1	8.2	0.06	5.24
1	1	0	100	6.84	1	83	20.5	103.5	59	155	30	125	13.2	5.2	56.3	1	1	0	1	0	1	1	6.7	0.05	71.3
1	0	0	100	5.16	2	78	25.6	133	87	198	30	168	15.3	6	39.2	1	1	1	0	0	1	0	161.8	0.35	106
1	0	0	100	6.95	2	74	28.7	123	81.5	149	30	119	15.6	5	73.2	1	1	1	0	0	1	1	63.7	0.1	12
1	1	0	100	4.64	2	74	26.6	124.5	73.5	148	30	118	11.4	5.8	69.6	1	1	1	0	0	1	1	58.6	0.03	70.1
1	1	1	3.3	3.3	1	70	21	148.5	82.5	199	30	169	9.4	5.1	12.7	2	1	1	0	0	1	1	840.5	0.67	24.7

1	0	0	100	6.98	1	74	23.6	129.5	68.5	191	31	160	11.5	6.5	71.5	1	1	1	0	0	0	1	15.5	0.25	5.68
1	0	0	100	4.59	1	73	24.4	126	68.5	162	31	131	15.2	5.3	72.0	2	1	0	0	0	1	1	4.5	0.26	23.9
1	0	0	100	6.88	1	68	21.6	109	66.5	181	31	150	16	5.6	71.1	2	0	1	1	0	1	1	13.1	0.08	17.1
1	0	0	100	6.84	1	75	23	134.5	74	229	31	198	13.3	5.9	75.1	2	1	0	1	0	1	1	13.2	0.02	7.93
1	0	0	100	5.08	1	69	23.6	138	69.5	156	31	125	13.1	6	70.6	2	1	0	0	0	0	1	50.5	0.06	8.95
1	0	0	100	1.22	1	74	19.1	129.5	71	163	31	132	14	5.5	60.0	2	0	1	0	0	1	1	50.4	0.01	41.1
1	0	0	100	6.96	1	74	23.2	154	79.5	96	31	65	14.6	5.4	71.5	2	1	0	0	0	1	1	9.3	0.07	0.05
1	0	0	100	6.8	2	74	23.6	103	61	184	31	153	13.6	5.5	73.2	2	1	1	1	0	1	1	10.8	0.46	45.2
1	0	0	100	4.58	2	66	25.2	147.5	80	231	31	200	13.2	5.9	73.6	2	1	1	0	0	1	1	12.9	0.04	42.2
1	0	0	100	4.59	2	67	22.3	122	80	147	31	116	12.8	6	87.9	2	1	1	1	0	1	1	18	0.06	12
1	0	0	100	5.04	2	76	25.9	160	89.5	189	31	158	13	5.8	50.7	2	1	1	0	0	1	1	37.3	0.03	40.4
1	0	0	100	6.95	1	78	28.4	136	86	187	31	156	14.7	5.1	52.0	2	1	1	0	0	1	1	1729.8	0.17	24.3
1	0	0	100	6.98	1	74	24.9	143	73.5	180	31	149	15.9	7	75.6	2	0	1	0	0	0	1	18.4	0.43	4.55
1	1	0	100	1.33	2	71	30.5	143.5	66	99	31	68	6	4.8	79.4	2	1	1	0	0	1	1	13.8	0.18	48.2
1	0	1	3.2	3.2	1	69	27.6	140	81.5	184	31	153	15.9	5.7	62.2	2	1	0	0	0	1	1	55.2	0.09	18.9
1	1	1	3.6	3.6	1	79	28.8	132	71	264	31	233	15.9	6.2	57.9	2	1	1	0	0	1	1	48.9	0.17	11.1
1	0	0	100	4.58	1	77	22.2	155.5	84.5	172	32	140	14.9	6.4	47.1	2	1	0	0	0	1	1	912.9	0.14	22.8
1	0	0	100	6.88	1	77	21.9	99	60.5	240	32	208	16.1	5.1	66.7	2	0	1	1	0	1	1	7.3	0.01	26.8
1	0	0	100	5.05	1	72	25.7	175	108.5	141	32	109	14.7	5.4	60.9	2	1	1	0	0	1	1	3.8	0.05	9.86
1	0	0	100	6.96	1	74	25.6	161	87.5	114	32	82	13.1	4.9	68.2	2	1	1	0	0	1	1	129.6	0.01	58.8
1	0	0	100	6.87	1	79	23.3	135	85.5	195	32	163	15.4	5.4	65.8	2	1	0	1	0	1	1	6.3	0.05	18.8
1	0	0	100	6.87	2	71	27.3	159.5	80	216	32	184	11.7	5.6	71.1	2	1	1	0	0	1	1	21	0.12	23
1	0	0	100	4.62	2	68	32	135	83	235	32	203	13.8	5.8	53.6	2	1	0	0	0	1	1	47.7	0.26	46.6
1	0	0	100	5.06	2	65	25.5	117	70	218	32	186	12.6	5.7	82.8	2	1	1	0	0	1	1	21.1	0.01	14.5
1	0	0	100	6.87	1	71	25.3	109.5	69.5	155	32	123	14.9	5.8	73.1	2	1	1	0	0	0	1	7.7	1.56	6.51
1	0	0	100	4.59	1	72	28.4	145	69	275	32	243	13.6	6.4	60.9	2	1	1	0	0	1	1	16.1	0.21	110
1	0	0	100	5.05	1	73	22.9	131	67.5	197	32	165	14.3	5.6	72.0	2	1	0	1	0	1	1	37	0.07	25.2
1	0	0	100	6.8	1	77	24.6	129	67.5	251	32	219	14.8	5.9	66.7	2	1	0	0	0	1	1	5.8	0.06	6.26
1	0	0	100	6.87	1	66	29.7	133	83.5	240	32	208	14.5	5.9	63.5	1	1	1	1	0	1	1	17.5	0.08	14
1	0	0	100	5.06	2	74	30.7	123.5	70	203	32	171	13.8	7	77.8	2	1	1	0	0	0	1	79.5	0.12	24.8
1	0	0	100	6.98	2	72	24.1	115.5	73	207	32	175	12.7	5.5	74.3	2	1	0	1	0	1	1	11.6	0.03	0.05
1	1	0	100	6.86	2	76	31	116.5	70	177	32	145	10.9	5.9	76.7	1	1	1	1	0	1	1	18.6	4.37	33.4
1	0	0	100	2.05	1	80	18	151	75	107	32	75	10.1	7	68.6	2	0	1	0	0	0	1	37.7	5.69	118
1	1	1	3.1	3.1	1	83	29.8	129.5	72	204	32	172	13.2	5.9	67.2	2	1	1	1	0	1	0	5.8	0.05	85.4
1	0	0	100	3.69	1	79	20	94.5	54	147	33	114	10.4	6.2	65.8	2	1	0	1	0	1	1	6.5	1.65	36.9
1	0	0	100	7.04	1	65	23.1	117.5	69	199	33	166	13.5	5.1	57.0	2	1	1	1	0	1	1	4.1	0.12	15.4
1	0	0	100	6.96	1	65	24.9	111	70.5	202	33	169	13.8	6.4	72.6	1	0	0	1	0	1	1	32.1	0.5	10.1
1	0	0	100	6.83	1	72	25.3	128	74.5	207	33	174	14.2	6.5	72.6	2	1	0	0	0	0	1	92.7	0.27	84.9
1	0	0	100	7.07	1	74	29.1	136.5	78.5	197	33	164	14.4	5.1	68.2	2	1	1	1	0	1	1	13.6	0.01	0.05
1	0	0	100	7.08	1	74	20.8	134.5	82.5	149	33	116	16.3	5.8	68.2	2	1	1	1	0	1	1	11.7	0.07	16.7
1	0	0	100	6.87	1	67	19.7	101.5	64.5	168	33	135	16.3	5.3	79.4	2	0	1	1	0	1	1	4	0.09	8.53
1	0	0	100	6.87	1	73	25.1	155.5	95.5	180	33	147	14	5.4	68.6	2	1	0	0	0	1	1	4.8	0.04	27.1
1	0	0	100	7.21	1	72	26.3	114.5	86	137	33	104	13.7	5.1	60.9	2	1	1	1	0	1	1	47	0.06	50.2
1	0	0	100	7	1	68	24.7	122.5	71	205	33	172	14.7	5.8	74.6	2	1	1	0	0	0	1	483.4	0.04	20.8
1	0	0	100	6.87	1	76	26.1	107.5	63	183	33	150	14.7	5	52.7	2	1	1	1	0	1	1	7.1	0.03	14.4
1	1	0	100	2.02	1	78	23.9	108	61.5	184	33	151	14	5.9	46.8	2	1	1	1	0	1	0	6	0.14	240
1	1	0	100	4.59	1	68	22.3	108	70	196	33	163	12.3	5.6	62.6	2	1	1	0	0	1	1	54.5	4.76	5.82
1	1	0	100	5.04	1	79	25.1	156.5	78	219	33	186	15.4	6.1	73.0	2	0	1	1	0	1	1	16.4	0.12	16.9
1	0	0	100	4.64	2	73	25.2	142	82.5	213	33	180	14	5.2	70.1	1	1	1	0	0	1	1	14.4	0.19	25.2
1	0	0	100	7	2	72	24.4	116	72	208	33	175	12.3	5.4	74.3	2	1	1	1	0	1	1	35.9	0.01	17
1	0	0	100	6.98	2	68	24.8	147	78.5	141	33	108	14.1	4.4	81.1	1	1	1	0	0	1	1	16.5	0.02	35.9

1	0	0	100	5.07	2	72	23.2	118	63	209	33	176	12.6	5.6	45.9	1	1	1	1	0	1	1	190	0.09	29.4
1	0	0	100	5.04	1	82	24.7	149.5	64	239	33	206	12.2	6	50.6	2	1	1	0	0	1	1	2334.1	0.21	89.9
1	0	0	100	4.58	1	69	31.8	177.5	87	176	33	143	13.2	6.2	70.6	2	1	1	0	0	1	1	27.3	0.39	11.7
1	0	0	100	7.01	1	70	27.5	114	77	195	33	162	13	5.5	70.1	1	1	1	1	0	1	1	8	0.06	5.11
1	0	0	100	6.87	1	74	27.4	122.5	75	219	33	186	14.6	5.2	68.2	2	0	1	1	0	1	1	41	0.02	10.9
1	0	0	100	7.06	2	70	29.3	140.5	73.5	193	33	160	13.8	5.2	71.6	2	1	1	0	0	1	1	7.2	0.1	33
1	0	0	100	6.86	2	74	25.9	128	53.5	211	33	178	11.7	5.3	40.3	2	1	1	0	0	0	1	4.5	0.45	117
1	0	0	100	7.02	2	72	23.6	136	73	200	33	167	13.1	6.6	78.9	1	1	1	0	0	0	1	9.6	0.32	7.91
1	0	0	100	4.6	2	76	25.2	133	63.5	299	33	266	11.5	5.7	50.7	1	1	1	0	0	1	1	9.2	0.2	32.4
1	0	0	100	4.61	2	66	24.1	118.5	74	212	33	179	12.5	5.5	73.6	2	1	1	0	0	1	1	12.7	0.4	6.32
1	1	0	100	7.01	2	76	26.4	95.5	65.5	184	33	151	14.3	5.4	44.6	1	1	1	1	0	1	1	51.2	0.07	26.1
1	0	1	1.1	1.1	1	76	22.9	150	83.5	119	33	86	13.9	6.9	59.2	2	1	1	0	0	0	1	262.5	0.01	37.7
1	0	0	100	4.58	1	75	25.4	142.5	79.5	174	34	140	16.5	5.2	71.0	2	0	1	0	0	1	1	8.8	0.35	24.9
1	0	0	100	6.86	1	77	25.9	158	84.5	217	34	183	13.9	5.1	66.7	2	1	1	0	0	1	1	20.8	0.33	11.7
1	0	0	100	6.96	1	66	27.3	114.5	77	213	34	179	14.6	5.8	75.7	2	0	1	0	0	1	1	9.6	0.13	4.93
1	0	0	100	7.31	1	70	25.1	131	75	153	34	119	13.1	5.3	55.0	1	1	1	1	0	1	1	23.3	0.22	26
1	0	0	100	6.8	1	73	25.4	148.5	81	146	34	112	15.4	5.2	68.6	2	1	0	0	0	1	1	109.5	0.18	15.1
1	0	0	100	5.06	1	75	26.1	126	69	114	34	80	12.8	5.7	71.0	2	1	1	1	0	1	1	5.6	0.31	18.9
1	0	0	100	5.08	1	76	26.1	141	78.5	189	34	155	17.4	5.4	74.5	1	1	0	0	0	1	1	14.8	0.31	4.71
1	0	0	100	7.06	1	73	29.9	107	65	158	34	124	16	7	68.6	1	1	1	1	0	0	1	37.3	0.05	13.4
1	0	0	100	6.86	1	70	23.8	161.5	83.5	185	34	151	14.6	5.4	73.6	2	1	1	0	0	1	1	26	0.01	4.01
1	0	0	100	1.75	1	72	23.2	136	64.5	149	34	115	13.6	6.4	76.7	2	1	1	1	0	0	1	17.2	0.01	49.4
1	0	0	100	7.03	1	71	28.3	118	66.5	149	34	115	14.9	6.6	77.2	2	0	0	1	0	0	1	14.1	0.05	21.7
1	0	0	100	5.06	1	66	28.7	134	77	158	34	124	15.3	5.4	79.9	2	0	0	1	0	1	1	15.3	0.05	22.6
1	0	0	100	7	1	72	21.9	140	76.5	128	34	94	14.6	5.1	76.7	2	1	1	0	0	1	1	9.7	0.02	28.5
1	0	0	100	4.59	1	69	24.3	117	61.5	116	34	82	14.9	4.9	78.3	2	0	1	0	0	1	1	35.2	0.03	10.7
1	0	0	100	6.45	1	76	19	130	71	157	34	123	13.8	5.5	70.5	1	0	1	1	0	1	1	35.7	0.04	24.3
1	0	0	100	6.87	1	65	20.7	107	66.5	162	34	128	15	5.5	76.2	2	0	1	1	0	1	1	7	0.05	0.05
1	1	0	100	5.32	1	72	20.6	109	63.5	196	34	162	13	5.1	76.7	2	1	1	1	0	1	1	3.7	0.07	16.7
1	0	0	100	4.59	2	75	22.4	132.5	77	153	34	119	12.1	5.8	58.8	2	1	1	0	0	1	1	9.6	2.71	48.6
1	0	0	100	7.03	2	72	25.8	100	51	152	34	118	12.6	5.6	74.3	1	1	1	1	0	1	1	11.3	1.93	186
1	0	0	100	5.06	2	76	26.9	151	73.5	201	34	167	13	5.5	58.4	1	1	1	0	0	1	1	28.6	0.56	33.3
1	0	0	100	6.97	2	69	26	119	71.5	188	34	154	13.3	5	86.7	1	1	1	1	0	1	1	19.7	0.02	7.24
1	1	0	100	4.12	2	71	23.1	126	63.5	191	34	157	13.1	5.4	60.5	2	1	1	0	0	1	1	12.2	0.03	16.9
1	0	0	100	6.83	1	82	25.4	117.5	69	168	34	134	14.8	5	71.5	1	0	1	1	0	1	1	7	0.04	18.2
1	0	0	100	6.88	1	65	28	103.5	62.5	244	34	210	14.7	5.2	76.2	2	0	0	1	0	1	1	6	0.32	1.48
1	0	0	100	7.07	1	69	25.6	141	78.5	224	34	190	14.2	6.3	62.2	2	0	1	0	0	1	1	10.3	0.03	0.05
1	0	0	100	4.58	1	79	22.3	125	65	164	34	130	13.5	6.1	65.8	2	1	0	1	0	0	1	82.1	0.07	62.3
1	0	0	100	5.15	1	71	22.8	132	80	170	34	136	15.1	5.4	73.1	2	1	1	0	0	1	1	10.6	0.05	23.9
1	0	0	100	5.05	1	73	24.8	151	82	187	34	153	14.1	5.6	60.4	2	1	1	0	0	1	1	14	0.08	61.6
1	0	0	100	5.07	1	65	25.8	101.5	67	171	34	137	14.7	5.4	72.6	2	0	0	1	0	1	1	5.4	0.04	20.4
1	0	0	100	1.06	1	70	25.2	155.5	80.5	166	34	132	12.8	4.5	55.0	2	1	1	0	0	1	1	21.7	0.04	14
1	0	0	100	5.04	1	67	27.5	139	87.5	150	34	116	14.6	5.4	42.0	2	1	1	0	0	1	1	310	0.04	26.5
1	1	0	100	7.08	1	76	24.5	134.5	74	187	34	153	14.5	5.2	74.5	2	1	0	0	0	1	1	34.9	0.1	303
1	0	0	100	7.01	2	67	28	142	86	195	34	161	14.5	5.6	81.7	1	1	1	0	0	1	1	14.6	0.12	18.1
1	0	0	100	6.86	2	77	26.1	165.5	91	166	34	132	13.1	5.5	71.7	1	1	1	0	0	1	1	16.9	0.14	29.8
1	0	0	100	4.63	2	69	26.6	131.5	71	155	34	121	12.9	5	72.1	2	1	1	0	0	1	1	3.5	0.08	205
1	1	0	100	5.36	2	73	27	130.5	85.5	212	34	178	13.3	5.5	73.8	1	1	1	1	0	1	1	34.3	0.03	12.9
1	0	0	100	4.64	1	80	27	113	53	183	34	149	12.3	6	51.3	1	1	1	0	0	1	1	57.2	0.18	234
1	1	1	2.3	2.3	2	72	29.1	132	82.5	202	34	168	13.9	5.4	70.6	1	1	1	1	0	1	1	18.6	0.02	19.3
1	0	0	100	4.59	1	67	23.1	110	65	202	35	167	15.9	5.2	75.2	2	0	1	1	0	1	1	8.6	0.1	3.27

1	0	0	100	4.59	1	74	25	146	82.5	205	35	170	15.1	5.2	48.1	2	1	0	0	0	1	1	40.9	0.1	29.1
1	0	0	100	6.86	1	71	21.6	158	95.5	172	35	137	13.7	4.9	32.3	1	1	0	0	0	1	1	508.8	0.21	14.8
1	0	0	100	6.71	1	65	22.9	134	71.5	166	35	131	14.6	5.6	63.9	2	0	0	0	0	1	1	10	0.25	9.85
1	0	0	100	5.04	1	66	28.1	113.5	79	143	35	108	14.5	5.2	75.7	2	1	0	1	0	1	1	9.3	0.2	14.9
1	0	0	100	5.13	1	72	23.9	119	75	152	35	117	10.6	5.7	69.1	2	1	1	1	0	1	1	6.2	0.08	9.97
1	0	0	100	7.08	1	66	24.1	176.5	89.5	212	35	177	15	5.2	75.7	2	1	0	0	0	1	1	33.6	0.08	48.1
1	0	0	100	4.58	1	71	22.9	143	90	237	35	202	14.8	6.1	61.3	2	1	0	0	0	1	1	18.2	0.05	15.3
1	0	0	100	7.04	1	68	27.3	138.5	82	189	35	154	14.7	5.3	78.8	2	1	1	1	0	1	1	7	0.03	0.05
1	0	0	100	6.95	1	79	24.1	107.5	63	187	35	152	16.1	5.2	73.0	2	0	1	1	0	1	1	4.4	0.06	4.88
1	0	0	100	4.6	1	69	27.3	143.5	72	216	35	181	14.8	6	74.1	2	1	0	0	0	1	1	97.7	0.04	43.5
1	0	0	100	5.05	1	72	28.8	109	63.5	178	35	143	14.7	5.9	54.2	2	1	1	0	0	1	1	3.1	0.07	7.16
1	1	0	100	6.85	1	74	26.4	117	81	168	35	133	16.8	7.9	71.5	2	1	0	1	0	0	1	35.3	0.22	7.74
1	1	0	100	4.82	1	74	26.8	149	82.5	213	35	178	10.4	5.8	18.9	2	1	1	0	0	1	1	1480.4	0.17	21.4
1	0	0	100	1.43	2	66	34.8	127	73	209	35	174	13.7	5.6	62.7	2	1	0	1	0	1	1	13.8	0.44	10.9
1	0	0	100	6.86	2	71	31	158	80	155	35	120	14.4	7.2	46.2	2	1	0	0	0	0	1	2192.6	0.11	65
1	0	0	100	6.86	2	68	27	164	76	210	35	175	13.3	5.6	81.1	2	1	1	0	0	1	1	18.3	0.25	53.2
1	0	0	100	5.07	2	66	30.9	156	78	198	35	163	14	6	82.3	2	1	1	0	0	1	0	41.5	0.52	66.8
1	0	0	100	7	2	75	29.3	123.5	70.5	199	35	164	13	5.5	69.1	1	1	1	1	0	1	1	3.4	0.02	12.5
1	0	0	100	7.03	2	69	23.6	156	90	204	35	169	13.6	5.2	80.5	1	1	0	0	0	1	1	13.9	0.05	38.6
1	0	0	100	3.99	2	68	21.1	140	76	123	35	88	11.9	5.2	76.4	1	1	1	0	0	1	1	21.1	0.03	105
1	0	0	100	4.59	2	68	31	121.5	72	206	35	171	12.9	5	72.6	2	1	1	0	0	1	1	8.7	0.07	27.2
1	0	0	100	4.6	2	73	28.2	150	83.5	176	35	141	12.7	5.5	70.1	1	1	0	0	0	1	1	8.7	0.05	63.7
1	0	0	100	7.01	2	80	30.2	134	75.5	175	35	140	13.9	5.5	74.6	1	1	1	0	0	1	1	24.3	0.03	54.6
1	0	0	100	4.58	1	75	24.4	143	82	154	35	119	14.2	5.9	71.0	2	1	1	0	0	1	0	50	0.16	64.6
1	0	0	100	7.08	1	67	22.8	100	60.5	184	35	149	11.7	4.9	84.6	2	1	1	1	0	0	1	12.4	0.05	3.34
1	0	0	100	7.21	1	72	23.8	121	66.5	192	35	157	15.1	5.1	72.6	2	0	0	1	0	1	1	24	0.04	17.2
1	0	0	100	7.02	1	72	26.4	128.5	67	136	35	101	14.7	5.1	69.1	1	1	1	1	0	1	1	3.3	0.05	25.5
1	0	0	100	6.95	1	68	27.7	155.5	80	214	35	179	14.6	9.5	78.8	2	1	1	0	0	0	1	95.5	0.03	16.1
1	1	0	100	3.72	1	68	20.8	114.5	63.5	136	35	101	12.6	8.4	74.6	1	1	0	1	0	0	1	380.6	0.08	24.7
1	0	0	100	7.03	2	71	27.7	142	79	181	35	146	13.7	5.9	71.1	2	1	1	0	0	1	1	14.7	0.53	12.5
1	0	0	100	5.06	2	79	25.3	130.5	76	167	35	132	14.1	6	70.7	2	1	1	0	0	1	1	47.6	0.59	50.4
1	0	0	100	5.07	2	69	32.3	108	69	179	35	144	12.6	5.6	53.2	2	1	1	0	0	1	1	37.9	0.26	33.7
1	0	0	100	5.37	2	74	29.7	144.5	80.5	147	35	112	14.2	6.1	59.2	1	1	1	0	0	1	1	11.6	0.12	20.7
1	0	0	100	6.87	2	70	27.9	143	90	264	35	229	14.3	5.9	75.3	2	1	1	0	0	1	1	20.3	0.15	45.9
1	0	0	100	6.88	2	69	24.9	134	80	232	35	197	13.9	5.4	61.4	2	1	1	0	0	1	1	80.7	0.09	24.9
1	0	0	100	5.15	2	71	27.3	138	77	166	35	131	12.6	6	79.4	1	1	1	1	0	1	1	13.3	0.04	17
1	0	0	100	5.16	2	66	29.1	163.5	88	209	35	174	15.2	10.6	62.7	2	1	1	0	0	0	1	117.4	0.04	29.3
1	0	0	100	6.85	2	70	24.9	139.5	77.5	230	35	195	12.1	5.7	75.3	1	1	1	0	0	1	1	5.8	0.03	85.4
1	0	0	100	4.63	2	69	24.7	132	67.5	198	35	163	13.8	5.5	72.1	1	1	1	0	0	1	1	23.6	0.04	14.6
1	0	0	100	5.04	2	70	28.7	148	87	182	35	147	13.8	6.4	71.6	2	1	1	0	0	0	1	29.3	0.06	27
1	1	0	100	6.84	2	70	18.2	122	67.5	134	35	99	11.4	5.1	71.6	1	1	1	1	0	1	1	6.1	0.03	59.5
1	0	0	100	5.06	1	81	30.4	136	94.5	132	35	97	14.2	5.4	76.7	1	1	1	0	0	1	1	56	1.34	44.4
1	0	0	100	4.6	1	83	29.3	149.5	74	172	35	137	11.7	5.6	56.3	2	1	1	0	0	1	1	11.8	0.17	15
1	1	0	100	4.24	1	84	32.8	150	80	186	35	151	14.4	5.8	55.9	1	0	0	0	0	1	1	180.8	0.37	119
1	0	1	0.7	0.7	1	72	21.6	144	78.5	201	35	166	15.1	3.9	60.9	2	1	0	0	0	1	1	35.3	0.04	0.05
1	0	0	100	4.58	1	65	24	121.5	71.5	194	36	158	15.8	6.4	80.5	2	0	0	1	0	0	1	52.2	0.53	1.61
1	0	0	100	6.87	1	70	20.8	107.5	66.5	208	36	172	13.8	5.4	61.7	1	0	1	1	0	1	1	23.1	0.2	64.9
1	0	0	100	5.06	1	68	26.8	100.5	63	128	36	92	15.1	5.6	84.0	2	0	0	1	0	1	1	9.9	2.7	42.6
1	0	0	100	6.87	1	65	23.6	117.5	80.5	136	36	100	14.6	6.2	72.6	2	1	0	1	0	1	1	6.7	0.13	0.05
1	0	0	100	6.82	1	73	28.1	165	91.5	175	36	139	16.3	5.4	81.1	1	1	1	0	0	1	1	64.1	0.1	63
1	0	0	100	4.69	1	77	29.9	137.5	76.5	182	36	146	16.5	9.7	47.1	2	0	0	1	0	0	1	11.2	0.21	22.3

1	0	0	100	7.06	1	77	27.4	99	67	167	36	131	15.5	5.2	66.7	1	1	1	1	0	1	1	7.3	0.03	15.9
1	0	0	100	4.58	1	65	24.4	112.5	72	210	36	174	14.3	5.3	80.5	2	1	0	1	0	1	1	9.9	0.03	5.36
1	0	0	100	4.58	1	66	22.7	110.5	63.5	163	36	127	14.6	4.7	75.7	2	1	0	0	0	1	1	8.2	0.07	5.94
1	0	0	100	6.87	1	70	25.1	146	86.5	153	36	117	14	5.2	73.6	2	1	1	0	0	1	1	5	0.02	61.8
1	0	0	100	6.85	1	69	24	133.5	76.5	217	36	181	13.9	5.1	62.2	2	1	0	1	0	1	1	5.9	0.09	6.85
1	0	0	100	7.01	1	70	25.6	134	82	166	36	130	16	5.1	73.6	2	1	1	0	0	1	1	9.6	0.02	27
1	0	0	100	2.93	1	76	23.9	101	59	180	36	144	16	12	79.4	1	0	1	1	0	0	1	16.2	0.01	6.02
1	0	0	100	2.99	1	77	28	131.5	69	232	36	196	15	5	66.7	2	1	1	0	0	1	1	7	0.02	18.5
1	0	0	100	4.61	1	71	22.8	129	86.5	171	36	135	14.6	6.1	73.1	2	1	0	1	0	1	1	4.7	0.04	9.41
1	0	0	100	6.86	1	70	25.8	106	71	195	36	159	14.9	5.1	73.6	2	1	0	1	0	1	1	5.5	0.08	9.18
1	0	0	100	4.62	2	72	31.6	158.5	70	197	36	161	13.9	6.4	70.6	2	1	1	0	0	1	1	6.5	0.12	12.4
1	0	0	100	5.37	2	65	21.9	105.5	62.5	153	36	117	12.3	5.3	78.0	2	1	1	1	0	1	1	6.2	0.58	11.5
1	0	0	100	4.59	2	66	25.1	112	69	137	36	101	12.8	5.2	77.5	2	1	1	0	0	1	1	10.3	0.07	22.6
1	0	0	100	5.16	2	74	21.6	119	70	181	36	145	13.2	5.5	73.2	1	1	1	1	0	1	1	15.5	0.01	26.5
1	0	0	100	5.16	2	67	27.6	106	68	245	36	209	13.7	5.4	73.1	2	1	1	1	0	1	1	47.4	0.09	38
1	0	0	100	6.84	2	69	24.1	113	69.5	194	36	158	12.4	5.4	72.1	1	1	1	1	0	1	1	18.7	0.03	11.2
1	0	0	100	6.85	2	79	21.6	144	78	180	36	144	11.6	4.8	67.2	1	1	1	0	0	1	1	11.3	0.05	7.1
1	0	0	100	4.64	2	69	25.4	113.5	66	198	36	162	12.1	5.1	72.1	2	1	1	1	0	1	1	3	0.06	12.9
1	0	0	100	6.8	2	70	29.5	149	85	207	36	171	12	5.4	60.9	2	1	1	0	0	1	1	51.8	0.06	26.9
1	0	0	100	6.85	2	77	28.2	139.5	72	188	36	152	15.1	5.2	68.2	1	1	1	0	0	1	1	23.5	0.03	14.5
1	1	0	100	6.86	2	72	24.9	162.5	84	251	36	215	14.6	5.7	74.3	1	1	1	0	0	1	1	56.2	0.11	9.98
1	0	0	100	7.06	2	83	21.5	133.5	68	164	36	128	12.6	5.6	65.3	1	1	1	0	0	1	1	38	0.29	50.4
1	0	0	100	6.45	1	75	21.3	169	85.5	150	36	114	15.7	5	67.7	1	0	1	0	0	1	1	337.7	0.42	91.4
1	0	0	100	7.03	1	71	21.8	108	62.5	183	36	147	13	5.5	77.2	2	1	1	1	0	1	1	30	0.04	225
1	0	0	100	6.96	2	75	28	162.5	90	249	36	213	13.8	5.7	77.2	2	1	1	0	0	1	1	1317.6	0.35	4.4
1	0	0	100	4.59	2	71	26.3	127.5	77	149	36	113	11.2	4.9	85.5	2	1	0	1	0	1	1	48.1	0.02	55.1
1	0	0	100	4.6	2	67	23.8	124	74	215	36	179	11.6	5.9	81.7	2	1	1	1	0	1	1	19.7	0.04	25.8
1	0	0	100	4.67	2	74	27.8	157	86	191	36	155	12.9	5.7	51.4	2	1	1	0	0	1	1	71.1	0.04	8.35
1	0	0	100	5.37	2	65	24.3	141.5	79	173	36	137	11.7	5.5	78.0	1	1	1	0	0	1	1	6	0.06	44.7
1	0	0	100	6.86	2	69	26.2	165	82.5	183	36	147	14.2	5.4	75.9	2	1	1	0	0	1	1	49.4	0.05	50.1
1	0	0	100	7	2	80	25.5	151	74	222	36	186	12.7	5.4	56.8	1	1	1	0	0	1	1	9.7	1.71	62.4
1	0	1	3.5	3.5	1	67	23.9	151.5	89	161	36	125	14.2	5.2	75.2	2	1	1	0	0	1	1	17.4	0.03	0.05
1	0	1	3.8	3.8	1	72	25.6	140	93.5	201	36	165	15.2	5.4	72.6	2	1	0	0	0	1	1	92.1	0.07	71.4
1	0	0	100	6.87	1	65	27.4	149	98	211	37	174	16.2	7	72.6	2	0	0	0	0	0	1	18.2	0.27	28.1
1	0	0	100	5.16	1	72	27.9	130	86.5	172	37	135	16.4	5.7	48.8	2	0	0	0	0	1	1	108.5	0.11	25.2
1	0	0	100	6.85	1	72	25	117	63.5	173	37	136	15.1	5.3	60.9	2	1	0	1	0	1	1	21.1	0.17	44.9
1	0	0	100	5.05	1	74	23.1	198.5	110	176	37	139	14.7	5.9	68.2	2	0	0	0	0	1	1	14.8	0.21	36.3
1	0	0	100	4.63	1	73	22.8	123	70.5	166	37	129	15.3	5.3	60.4	2	1	0	0	0	1	1	15.4	0.67	5.66
1	0	0	100	6.84	1	79	21.9	134	73	252	37	215	13.9	5.3	57.9	2	0	1	0	0	1	1	16.2	0.24	3.41
1	0	0	100	6.86	1	74	25.7	178.5	105.5	238	37	201	14.8	5.4	68.2	2	0	1	0	0	1	1	49.9	0.27	8.24
1	0	0	100	7	1	76	24.4	127.5	61.5	164	37	127	15.3	5.5	74.5	2	1	1	1	0	1	0	26.4	0.03	51.6
1	0	0	100	5.16	1	74	27.2	135.5	81.5	157	37	120	13.4	5.2	71.5	1	1	0	0	0	1	1	19	0.05	62.4
1	0	0	100	6.85	1	67	26.9	130.5	78.5	197	37	160	15.8	5.1	84.6	2	0	1	1	0	1	1	13.6	0.01	11.5
1	0	0	100	5.06	1	69	26.4	118	70.5	182	37	145	14.5	5.5	70.6	2	1	1	1	0	1	1	2.3	0.09	32
1	0	0	100	5.07	1	78	27.6	156.5	81.5	203	37	166	15.3	5.4	69.6	2	1	0	0	0	1	1	159.2	0.03	7.61
1	0	0	100	4.63	1	72	20.8	152	90.5	157	37	120	13.1	4.8	69.1	2	0	1	0	0	1	1	45.1	0.03	23.2
1	0	0	100	6.87	1	74	25	135	74	180	37	143	14.1	4.9	75.6	2	1	1	1	0	1	1	8.9	0.03	12.1
1	0	0	100	6.87	1	65	24	155	89.5	216	37	179	15.2	5.5	85.8	2	1	0	0	0	1	1	12.8	0.04	3.08
1	1	0	100	3.74	1	74	24.6	160	85.5	145	37	108	15.2	5.1	71.5	1	1	0	0	0	1	1	60.8	0.1	0.05
1	0	0	100	7.06	2	67	23.3	141.5	75.5	191	37	154	12.3	5.2	76.9	2	1	1	0	0	1	1	17	0.16	26.5
1	0	0	100	7.06	2	74	26	115.5	58.5	222	37	185	13.2	6.4	51.4	2	1	1	1	0	1	1	13.2	0.61	14.1

1	0	0	100	4.12	2	67	29.4	154.5	73	220	37	183	12.5	6	81.7	2	1	1	0	0	1	1	11.5	0.13	29.7
1	0	0	100	2.63	2	78	32	150.5	82.5	197	37	160	14.5	7.4	67.7	1	1	1	0	0	0	1	33.6	0.52	25.5
1	0	0	100	6.8	2	73	27	118	66	196	37	159	14.5	7.4	70.1	1	1	1	1	0	0	1	48.6	0.34	2.58
1	0	0	100	6.8	2	70	27.2	151	91	175	37	138	14.4	5.3	75.3	2	1	1	0	0	1	1	19.4	0.52	33.8
1	0	0	100	6.85	2	65	26.3	129.5	74	222	37	185	13.4	5.6	74.2	2	1	1	1	0	1	1	11.7	0.33	7.12
1	0	0	100	6.98	2	66	27	120	69	181	37	144	13.7	4.5	73.6	2	1	1	0	0	1	1	36.6	0.06	0.05
1	0	0	100	6.8	2	70	25.7	108.5	70	173	37	136	13.5	6.6	71.6	2	1	1	1	0	0	1	4.7	0.05	11.1
1	0	0	100	5.37	2	65	27.1	127.5	82.5	207	37	170	13.7	5.2	78.0	2	1	1	1	0	1	1	201.7	0.07	14
1	0	0	100	5.05	2	68	24.5	129.5	81	223	37	186	13.3	5.7	76.4	2	1	1	1	0	1	1	14.9	0.08	27.7
1	0	0	100	6.45	2	75	21.6	142	75	164	37	127	10	4.8	32.7	1	1	1	0	0	1	1	31.3	0.02	28.9
1	0	0	100	6.86	2	75	24.8	126.5	69.5	156	37	119	12.5	5.6	72.7	2	1	1	1	0	1	1	106.9	0.03	20.6
1	0	0	100	0.89	1	81	23.4	148	79	155	37	118	15.4	5.6	64.9	2	1	1	0	0	1	1	9.4	0.01	53.8
1	0	0	100	6.8	1	68	26.8	108.5	74.5	186	37	149	13.5	5.5	74.6	2	0	1	0	0	1	1	10.5	0.11	15
1	0	0	100	6.98	1	65	25.8	118.5	69.5	170	37	133	16	6.1	80.5	2	1	0	1	0	1	1	8.5	0.01	13.1
1	0	0	100	5.06	1	68	24.1	112	66.5	168	37	131	14	4.6	78.8	2	1	0	0	0	1	1	47.3	0.01	32.9
1	1	0	100	6.97	1	71	20.7	113	65	172	37	135	12.9	6.4	73.1	1	0	1	1	0	0	1	10.3	0.07	8.43
1	0	0	100	7.08	2	69	25.7	126	75.5	252	37	215	13.3	5.7	80.5	1	1	1	1	0	1	1	51.5	0.12	11.5
1	0	0	100	6.85	2	75	29.1	124	65.5	250	37	213	14.6	7.1	69.1	2	1	1	0	0	0	1	158	0.18	64.6
1	0	0	100	5.07	2	76	27.7	117	64.5	198	37	161	14.3	5.8	72.2	1	1	1	0	0	1	1	50.4	1.46	15.7
1	0	0	100	5.07	2	74	27.5	150.5	76	206	37	169	14.8	5.3	69.6	2	1	1	0	0	1	1	203.9	0.22	39
1	0	0	100	6.99	2	66	31.8	108	68.5	213	37	176	13.6	5.2	73.6	1	1	1	0	0	1	1	7.2	0.1	12.8
1	0	0	100	6.99	2	67	34.8	144.5	87	181	37	144	13.7	7.1	76.9	2	1	1	0	0	0	1	5.3	0.12	33.7
1	0	0	100	4.63	2	79	25.9	127.5	70.5	172	37	135	11.7	5.1	67.2	1	1	1	1	0	1	1	15.1	0.11	25.6
1	0	0	100	6.86	2	72	25.7	103.5	62	172	37	135	11.4	5.1	74.3	2	1	1	1	0	1	1	11.6	0.06	15.2
1	0	0	100	5.06	2	68	25.2	112.5	64.5	153	37	116	11.9	6	76.4	2	1	1	1	0	1	1	4	0.03	40.6
1	0	0	100	4.67	2	66	21.2	116.5	76	174	37	137	12.4	5.5	77.5	2	1	1	1	0	1	1	12.7	0.05	50.7
1	1	0	100	5.05	2	74	24.3	101.5	53	194	37	157	12.9	5.1	69.6	1	1	1	0	0	1	1	18.3	0.06	37.7
1	1	0	100	6.85	2	72	24.6	153	84	211	37	174	14.1	4.7	60.1	2	1	1	0	0	1	1	36.1	0.03	52.2
1	0	0	100	6.88	1	82	18.5	138	70.5	138	37	101	11.7	5	56.7	1	1	1	1	0	1	1	7.6	0.01	138
1	0	0	100	4.68	1	81	20.2	134.5	70.5	160	37	123	10.4	5.5	45.8	1	1	0	0	0	1	1	545.5	0.01	115
1	0	0	100	5.37	2	80	22.3	117.5	62	147	37	110	11.9	5.7	70.2	1	1	1	1	0	1	1	229.9	0.07	98.5
1	0	1	5.3	5.3	1	75	27.7	138	90	219	37	182	14.8	5.8	71.0	2	0	0	0	0	1	1	38.6	0.25	9.03
1	0	0	100	4.58	1	70	20.8	126	86	176	38	138	12.3	5.5	61.7	2	1	0	1	0	1	1	8.6	0.15	19.9
1	0	0	100	6.87	1	68	24.1	159	96	227	38	189	14.1	5.4	71.1	2	0	0	0	0	1	1	20.1	0.14	46
1	0	0	100	4.62	1	66	22.3	143	82	228	38	190	15.8	5.7	75.7	2	0	1	0	0	1	1	82	2.7	11
1	0	0	100	4.63	1	72	23.8	151	83	196	38	158	13.4	5.7	69.1	2	1	1	0	0	1	1	19.2	0.1	35.9
1	0	0	100	2.38	1	75	20	112.5	68	214	38	176	12.7	6.1	67.7	2	1	1	1	0	1	1	109.5	0.31	27.9
1	0	0	100	6.83	1	69	27.4	94	50	229	38	191	14.3	6	62.2	2	1	1	0	0	1	1	49.9	6.67	128
1	0	0	100	1.77	1	69	23.3	130	63	192	38	154	13.7	7	62.2	2	1	0	0	0	0	1	13.1	0.11	17.5
1	0	0	100	7.08	1	67	24.7	127.5	75.5	196	38	158	15.6	5.4	75.2	2	1	1	1	0	1	1	5.8	0.03	5.19
1	0	0	100	4.58	1	75	29	157	101.5	230	38	192	15.8	5.5	67.7	2	1	1	0	0	1	1	15.4	0.02	31.8
1	0	0	100	4.59	1	76	21.4	107	62	177	38	139	13.6	5.6	67.2	2	1	1	1	0	1	1	7.6	0.02	20.5
1	0	0	100	6.87	1	66	23.6	112	72.5	155	38	117	15.7	5.7	46.2	1	0	0	0	0	1	1	12.2	0.07	30.6
1	0	0	100	6.85	1	67	22	129	77.5	253	38	215	14.8	5.8	75.2	2	0	0	0	0	1	1	11.9	0.09	26.9
1	0	0	100	6.85	1	69	23.6	135.5	78	177	38	139	13.9	4.9	74.1	2	1	0	1	0	1	1	11.1	0.07	16
1	0	0	100	6.99	1	79	20.4	129.5	66.5	158	38	120	13.8	5.7	73.0	2	0	1	1	0	1	1	31.8	0.03	42
1	0	0	100	4.64	1	79	23.2	176.5	104.5	208	38	170	17	10.4	69.1	2	0	0	0	0	0	0	55.5	0.09	29.9
1	0	0	100	4.6	1	73	26	150	81.5	174	38	136	14.9	5.1	72.0	2	0	1	0	0	1	1	10.6	0.06	8.8
1	0	0	100	4.62	1	77	25.1	141	89	187	38	149	14.5	6.6	70.1	2	1	0	0	0	0	1	7.9	0.04	20.7
1	0	0	100	4.62	1	73	24.9	140.5	74	171	38	133	12.9	5.7	68.6	2	0	0	0	0	1	1	38.9	0.04	49.5
1	0	0	100	6.87	1	70	23.6	143.5	72	261	38	223	16.5	7	70.1	2	0	1	0	0	0	1	66.8	0.05	10.6

1	0	0	100	6.87	1	72	23.6	142.5	80.5	195	38	157	17.8	5.9	76.7	2	0	0	0	0	1	1	54	0.08	21.1
1	0	0	100	5.04	1	72	28.1	120	81.5	169	38	131	14.2	5.5	72.6	2	0	0	1	0	1	1	161.1	0.03	32.9
1	1	0	100	2.55	1	72	23.4	154.5	83	207	38	169	13.4	5.8	69.1	2	1	1	0	0	1	1	10.1	0.03	12.8
1	0	0	100	6.85	2	72	28.4	161.5	81	261	38	223	12.6	5.1	60.1	2	1	1	0	0	1	1	94.6	0.21	68.7
1	0	0	100	6.85	2	74	21.8	153.5	74	172	38	134	12.2	5.2	69.6	1	1	1	0	0	1	1	16.3	0.15	62.8
1	0	0	100	4.6	2	65	20.4	98.5	56.5	145	38	107	10.9	5.9	82.8	2	1	1	1	0	1	1	75.6	2.96	33.1
1	0	0	100	5.37	2	74	27.7	122	63	172	38	134	13.8	6	69.6	2	1	1	0	0	1	1	19.1	0.11	37.7
1	0	0	100	4.58	2	65	20.6	119.5	67.5	195	38	157	12.6	5.8	82.8	2	1	1	0	0	1	1	31.2	0.03	21.7
1	0	0	100	6.84	2	71	23.1	133	64.5	214	38	176	12.9	5.6	74.8	1	1	1	0	0	1	1	9.9	0.03	2.63
1	0	0	100	5.07	2	75	23.3	178	98.5	238	38	200	13.7	5.2	72.7	1	1	1	0	0	1	1	6.5	0.05	9.19
1	0	0	100	6.98	2	73	24.7	151.5	77	185	38	147	12.2	5.8	73.8	1	1	1	0	0	1	1	14.8	0.06	5.17
1	0	0	100	4.63	2	67	25.3	99.5	63.5	206	38	168	13.5	10.7	81.7	2	1	1	1	0	0	1	11.5	0.07	11.6
1	0	0	100	5.11	2	75	29.6	145.5	76	255	38	217	13.1	4.9	51.0	1	1	1	0	0	1	1	484.4	0.09	17.8
1	0	0	100	6.84	2	76	20.6	132	82	198	38	160	11.4	6.5	58.4	1	1	0	0	0	0	1	6.8	0.01	27.6
1	0	0	100	4.59	2	68	21	120	65	266	38	228	13.3	5.6	61.8	2	1	1	1	0	1	1	11.4	0.01	32.1
1	0	0	100	4.62	2	71	24.8	147.5	72.5	193	38	155	13.5	5.6	74.8	2	1	1	0	0	1	1	24.5	0.05	91.6
1	0	0	100	7.07	1	70	21.4	105.5	65.5	209	38	171	13.5	4.8	77.7	1	1	0	1	0	1	1	12.9	0.13	13
1	0	0	100	6.87	1	71	28.7	129.5	69.5	228	38	190	13.5	6.2	11.8	2	1	1	0	0	0	1	1804.3	0.12	17.2
1	0	0	100	1.7	1	76	22.7	150	87	211	38	173	16.5	5.5	47.5	2	1	1	0	0	1	1	560.3	0.1	24.8
1	0	0	100	6.85	1	73	24	131	76	168	38	130	14	5.4	76.1	2	1	0	0	0	1	1	7.2	0.6	8.15
1	0	0	100	5.05	1	68	36	116.5	71	195	38	157	15.6	8.5	71.1	2	1	0	1	0	0	1	62	0.42	3.36
1	0	0	100	6.96	1	67	18.1	139.5	81.5	146	38	108	12.2	5.5	84.6	2	1	1	0	0	1	1	9.1	0.51	9.26
1	0	0	100	5.37	1	69	25.9	120.5	68.5	226	38	188	16.5	5.8	74.1	2	0	0	1	0	1	1	99.8	0.35	4.43
1	0	0	100	7.06	1	78	20	150.5	88	145	38	107	14.8	4.9	66.3	2	0	0	0	0	1	1	34.7	0.04	29.1
1	0	0	100	7.08	1	76	23	134.5	72	152	38	114	13.4	4.9	70.5	2	0	1	0	0	1	1	24.1	0.06	25.4
1	0	0	100	4.58	1	73	21.4	122	83	167	38	129	13.5	5.8	72.0	2	0	0	1	0	1	1	4.7	0.01	22.7
1	0	0	100	6.85	1	79	29	134.5	76	174	38	136	13.3	5.8	65.8	2	1	1	0	0	1	1	55.2	0.08	57.6
1	0	0	100	5.08	1	68	26.7	140	79	206	38	168	14.9	5.2	74.6	2	1	0	0	0	1	1	4.8	0.06	13.2
1	0	0	100	6.83	1	72	24.8	116.5	70	240	38	202	15.2	6.1	60.9	2	0	1	1	0	1	1	7.7	0.06	18.9
1	0	0	100	1.81	1	67	24	138	82	218	38	180	17	5.9	71.6	2	0	0	1	0	0	1	6.1	0.02	1.11
1	0	0	100	1.55	1	73	20.2	118.5	61.5	137	38	99	15	4.9	53.9	2	1	1	1	0	1	1	11.3	0.07	13.9
1	1	0	100	4.58	1	72	22.8	119	71	202	38	164	14.7	6.3	76.7	2	0	1	1	0	1	1	15.1	0.01	19.7
1	0	0	100	4.53	2	68	30.2	102.5	72.5	183	38	145	11.1	5.8	72.6	2	1	1	1	0	1	1	9.5	0.12	36.3
1	0	0	100	6.86	2	74	25	128	67	209	38	171	12.7	4.9	59.2	2	1	1	1	0	1	1	7.9	0.1	50.4
1	0	0	100	6.86	2	72	24.8	142.5	75	211	38	173	11.3	6.1	70.6	1	1	1	0	0	1	1	6.8	0.12	54.1
1	0	0	100	6.87	2	76	25.8	145	86	154	38	116	11.4	5.9	76.7	2	1	1	0	0	1	1	5.6	0.15	81.3
1	0	0	100	6.87	2	67	21.5	159	75.5	219	38	181	14.4	7.7	76.9	2	1	1	0	0	0	1	239.9	0.1	5.88
1	0	0	100	6.83	2	67	28.2	122	72	219	38	181	13.9	5.3	62.2	2	1	1	0	0	1	1	13.8	0.19	25.6
1	0	0	100	6.96	2	67	25.8	128	79	168	38	130	13.9	5	81.7	2	1	0	1	0	1	1	25.9	0.63	1.44
1	0	0	100	5.04	2	68	30.9	132.5	71	200	38	162	13.7	7.3	87.3	2	1	0	1	0	0	1	244	0.29	5.34
1	0	0	100	6.86	2	68	25.1	126.5	85.5	203	38	165	13	5.1	72.6	1	1	1	0	0	1	1	11	0.03	28.5
1	0	0	100	6.86	2	72	25.5	139.5	88	207	38	169	13.6	5.7	60.1	2	1	1	0	0	1	1	18.3	0.05	59.7
1	0	0	100	6.87	2	74	22.3	148	82.5	226	38	188	11.8	5.1	73.2	1	1	1	0	0	1	1	3.7	0.04	7.83
1	0	0	100	5.16	2	69	31.6	127.5	71.5	179	38	141	12.6	5.9	61.4	1	1	1	0	0	1	1	23.4	0.06	139
1	0	0	100	5.07	2	71	31.4	158.5	83	192	38	154	15.1	6.3	79.4	2	1	1	0	0	1	1	102.9	0.09	31.7
1	0	0	100	6.8	2	74	24	118.5	79	130	38	92	13	4.8	73.2	2	1	1	1	0	0	1	23.3	0.02	21.6
1	0	0	100	5.37	2	75	25.7	127.5	67.5	214	38	176	12.7	5.4	69.1	1	1	1	0	0	1	1	992.6	0.06	64.6
1	1	0	100	5.37	2	79	23.6	122	62	230	38	192	12.7	6.2	67.2	1	1	1	0	0	1	1	36.3	0.11	49.6
1	0	0	100	4.63	2	80	27.4	131.5	72	146	38	108	12.7	5.6	66.7	1	1	1	0	0	1	1	24	0.24	42.2
1	0	1	3.1	3.1	2	69	24.7	127.5	60.5	207	38	169	12.2	5	75.9	2	1	1	1	0	1	0	22.3	0.09	64.9
1	1	1	4.2	4.2	2	76	21.3	132	73.5	180	38	142	14.5	5.5	76.7	2	1	1	0	0	1	1	20.4	0.28	12.8

1	0	1	1.6	1.6	1	81	25.2	137	77.5	188	38	150	14.8	5.6	64.9	2	1	1	1	0	1	1	4.6	0.04	193
1	0	0	100	4.58	1	70	25.5	128.5	65.5	213	39	174	15.1	5.7	73.6	2	0	1	0	0	1	1	17	0.16	6.47
1	0	0	100	4.58	1	67	24.7	100.5	60.5	166	39	127	14.8	6	84.6	2	0	0	1	0	1	1	3.9	0.16	26
1	0	0	100	7.01	1	75	20.9	91	55	160	39	121	13.2	5.3	75.1	1	1	1	1	0	1	1	20.4	0.13	24.7
1	0	0	100	6.8	1	65	25.7	114	66	261	39	222	14.9	5.5	80.5	2	0	0	1	0	1	1	15.5	0.3	1.98
1	0	0	100	4.59	1	70	25	138.5	71.5	225	39	186	14.1	5.6	61.7	2	1	0	0	0	0	1	12.6	0.1	109
1	0	0	100	4.59	1	69	27.3	157	77	225	39	186	14.9	5.5	78.3	2	1	0	0	0	1	1	20.9	0.11	5.78
1	0	0	100	4.6	1	68	25.5	141.5	91	202	39	163	16.4	5	71.1	2	1	0	0	0	1	1	8.8	0.1	11.5
1	0	0	100	5.37	1	66	20.8	106	72	134	39	95	15.1	5.2	72.1	2	0	1	1	0	1	1	17.3	0.12	6.01
1	0	0	100	6.8	1	78	25.5	126.5	63.5	165	39	126	15.5	5.1	58.3	2	0	0	1	0	1	1	6.6	0.31	62.1
1	0	0	100	7.05	1	69	26.1	123.5	77.5	217	39	178	15.9	5.1	62.2	2	1	1	1	0	1	1	10.5	0.08	0.05
1	0	0	100	4.58	1	72	22.5	145	75.5	217	39	178	15.3	5.7	69.1	2	0	1	0	0	1	1	27.1	0.04	20.4
1	0	0	100	6.86	1	65	25.1	126.5	89.5	218	39	179	14.9	5.7	72.6	2	1	1	0	0	1	1	6.6	0.06	7.94
1	0	0	100	6.86	1	78	25.8	137.5	73.5	182	39	143	13.8	5	46.8	1	1	0	0	0	1	1	11.6	0.08	23
1	0	0	100	6.87	1	71	21.3	110.5	72	178	39	139	13.9	5.2	61.3	2	1	1	1	0	1	1	6	0.05	63.3
1	0	0	100	5.15	1	70	28	152.5	87.5	143	39	104	15.1	5.3	55.0	2	0	0	0	0	1	1	47.2	0.05	335
1	0	0	100	7.01	1	66	23	111	66	181	39	142	12.8	5.5	79.9	2	1	1	1	0	1	1	6.7	0.07	11.7
1	0	0	100	5.05	1	73	29.1	129.5	74.5	150	39	111	16.2	7.4	68.6	2	1	1	1	0	0	1	29	0.07	75.9
1	0	0	100	6.98	1	77	26.5	141.5	76.5	192	39	153	12.3	5	70.1	2	1	1	0	0	1	1	15	0.05	0.05
1	0	0	100	7	1	79	24.3	125	66	226	39	187	14.1	5	65.8	1	1	1	1	0	1	1	8.9	0.01	36.8
1	0	0	100	4.63	1	71	23.7	132.5	76	171	39	132	15.9	4.7	69.6	2	1	0	0	0	1	1	13.4	0.04	9.36
1	0	0	100	4.64	1	69	28	134.5	72	164	39	125	17	5.6	74.1	2	1	0	1	0	1	1	40.3	0.04	22
1	0	0	100	6.83	1	65	18.8	102	65	181	39	142	15.1	4.8	80.5	2	0	1	1	0	1	1	8.2	0.03	12.6
1	0	0	100	5.36	1	67	25.8	156.5	87	182	39	143	14.9	5.8	75.2	2	1	0	0	0	1	1	11.6	0.02	87.6
1	0	0	100	5.36	1	76	23.4	142	75.5	151	39	112	14.8	6.8	52.7	2	1	1	0	0	0	1	19	0.04	17.5
1	0	0	100	5.04	1	65	22.4	149.5	92.5	180	39	141	13.2	5.3	80.5	2	1	0	0	0	1	1	38.7	0.05	35.4
1	1	0	100	7.03	1	69	27.9	121.5	76.5	151	39	112	15.5	4.8	83.4	2	1	0	1	0	1	1	19.4	0.02	13.2
1	0	0	100	6.86	2	69	22.5	142	80.5	198	39	159	13.4	5.3	80.5	2	1	1	0	0	1	1	12.2	0.27	11.9
1	0	0	100	7.01	2	65	35.7	192.5	107.5	216	39	177	13.7	6.2	82.8	2	1	1	0	0	1	1	33.3	0.5	13.1
1	0	0	100	5.06	2	73	22.2	137	69.5	168	39	129	14	5.4	70.1	2	1	1	1	0	1	1	19.7	0.13	9.68
1	0	0	100	6.98	2	71	26.7	154.5	84	164	39	125	11.9	5.1	79.4	2	1	1	0	0	1	1	48.1	0.13	1.32
1	0	0	100	4.64	2	67	32.2	145.5	72.5	202	39	163	13.9	6.3	81.7	2	1	0	0	0	1	1	33.8	0.48	20.2
1	0	0	100	6.87	2	75	23.1	124.5	82.5	198	39	159	13.5	6	72.7	2	1	1	1	0	1	1	13.3	0.34	10.9
1	0	0	100	5.16	2	68	24	102	55	228	39	189	11.7	5.5	76.4	2	1	1	1	0	1	1	34.2	0.01	44.9
1	0	0	100	6.98	2	66	23.1	174.5	102.5	211	39	172	13.3	5.9	62.7	1	1	1	0	0	1	1	42.9	0.06	9.06
1	0	0	100	6.8	2	77	22.7	120.5	65.5	238	39	199	12.9	5.4	68.2	1	1	1	1	0	1	1	7.7	0.07	8.48
1	0	0	100	4.62	2	67	34.9	179	83.5	167	39	128	13.7	5.2	73.1	2	1	1	0	0	1	1	19.8	0.08	39.4
1	0	0	100	4.62	2	72	22.6	125.5	74.5	164	39	125	14.5	5.9	70.6	2	0	1	1	0	1	1	8.7	0.02	30.9
1	0	0	100	1.66	2	65	25	97	63	288	39	249	13	5.7	74.2	2	1	1	1	0	1	1	18.5	0.03	8.62
1	0	0	100	5.37	2	68	25.9	123.5	70	181	39	142	13.7	6.1	72.6	2	1	1	0	0	0	1	12.6	0.05	11.8
1	0	0	100	5.37	2	65	21.9	142	72	157	39	118	12.7	4.9	78.0	2	1	1	0	0	1	1	34.9	0.01	19.9
1	0	0	100	6.86	2	78	27.3	120	66	183	39	144	13.6	5.7	57.6	1	1	1	0	0	1	1	26.8	0.06	102
1	0	0	100	6.87	2	69	23	121.5	69.5	205	39	166	13.7	5.1	75.9	2	1	1	1	0	1	1	14.1	0.02	24.8
1	0	0	100	6.87	2	66	24	127.5	75	224	39	185	12.2	5.5	73.6	2	1	1	1	0	1	1	11.8	0.02	2.58
1	1	0	100	4.63	2	76	33.5	138	72.5	218	39	179	13.2	6.5	76.7	1	1	1	0	0	0	1	35.9	0.11	46.5
1	1	0	100	6.98	2	71	25.3	110	67	280	39	241	13.7	10.4	41.2	2	1	1	1	0	0	1	75.7	0.04	0.05
1	0	0	100	1.9	1	80	21	153	78	226	39	187	15.4	5.2	65.4	2	0	1	0	0	1	1	28.3	0.02	27.4
1	0	0	100	4.34	1	80	19.3	146	80	167	39	128	13.8	7.2	68.6	1	1	1	0	0	0	1	51.5	0.01	12
1	1	0	100	5.02	1	93	21	121.5	72.5	219	39	180	13.7	5.7	59.6	1	0	1	1	0	1	1	10.5	0.05	8.75
1	0	0	100	0.39	2	81	25.8	130	70	201	39	162	12.9	5.5	48.9	1	1	1	0	0	1	1	55	0.03	51.7
1	0	0	100	3.48	1	71	22.8	133	67.5	197	39	158	14.9	7.8	77.2	2	0	0	0	0	0	1	19.5	0.06	14.6

1	0	0	100	6.87	1	76	22.9	167.5	94	170	39	131	11.3	5.2	43.1	1	1	1	0	0	1	1	55.8	0.02	30.5
1	0	0	100	5.08	1	68	26	109	72	189	39	150	14.8	5.5	74.6	2	1	1	1	0	1	1	8	0.05	3.21
1	0	0	100	6.83	1	73	26.8	135.5	86	221	39	182	15.1	5.7	68.6	2	1	1	1	0	1	1	576.1	0.05	16.7
1	0	0	100	5.06	1	79	21.1	126.5	72	117	39	78	12.2	6.8	57.9	2	1	1	1	0	0	1	98.9	0.04	15.3
1	0	0	100	4.59	2	75	36.4	144	76	232	39	193	14	5.6	58.8	1	0	1	0	0	1	1	590.1	0.14	40.6
1	0	0	100	5.16	2	67	26.5	128	77	168	39	129	11.4	5.7	87.9	2	1	1	0	0	1	1	19.5	6.9	40.7
1	0	0	100	6.85	2	67	28	122	69	168	39	129	15.5	6.4	76.9	2	1	1	1	0	1	0	16.8	0.29	119
1	0	0	100	4.62	2	68	26.2	143.5	80	212	39	173	13.7	5.6	76.4	2	1	1	0	0	1	1	11	0.21	27.8
1	0	0	100	4.58	2	76	25.7	125.5	68.5	206	39	167	13.1	5.6	82.5	2	1	0	1	0	1	1	19	0.01	17.2
1	0	0	100	5.16	2	73	20.3	146.5	78.5	190	39	151	13.3	5.9	70.1	2	1	1	0	0	1	1	29	0.05	34.6
1	0	0	100	5.36	2	72	27.5	111	60	163	39	124	12.1	5.4	74.3	1	1	1	1	0	1	1	5.3	0.08	106
1	1	0	100	7.08	2	71	28.3	147.5	79	192	39	153	15	7.8	79.4	2	1	1	0	0	0	1	37.7	0.24	4.52
1	1	0	100	6.84	2	74	23.4	143	74.5	203	39	164	11.8	5.1	69.6	2	1	1	0	0	0	1	24.8	0.22	41
1	1	0	100	6.97	2	76	28.7	152	90	220	39	181	14.7	5.9	68.6	1	1	1	0	0	1	1	3.8	0.08	33.4
1	1	0	100	5.81	2	78	22.3	111	60	182	39	143	12.4	5.2	71.2	1	1	1	1	0	1	1	18.2	0.02	4.44
1	1	0	100	4.59	2	77	26	172	97.5	233	39	194	13.1	5.4	44.3	1	1	1	0	0	1	1	61.7	0.07	36.7
1	1	0	100	7.04	2	82	26.9	132	82.5	225	39	186	12.2	5.2	69.2	1	1	1	0	0	1	1	53.3	0.38	51.4
1	0	1	0.3	0.3	1	74	27.6	115	74	189	39	150	15.2	6.3	48.1	2	1	0	1	0	1	1	5.1	0.61	17.8
1	0	1	0	0	1	66	25.2	149	83.5	219	39	180	13.4	4.7	75.7	2	1	1	0	0	1	1	53.1	0.5	394
1	0	1	3.7	3.7	1	79	25.9	120	74	151	39	112	13.2	5	69.1	2	1	0	0	0	1	1	51.8	0.03	38.7
1	0	0	100	7.08	1	70	25.4	163.5	104.5	155	40	115	14.9	5.9	82.8	2	0	1	0	1	1	1	20.2	0.45	2.83
1	0	0	100	4.64	1	67	25.4	153	91.5	199	40	159	16.1	7.5	75.2	2	1	0	0	1	0	1	32.2	0.11	3.7
1	0	0	100	6.96	1	67	29.9	93.5	59.5	163	40	123	15.1	6	75.2	2	0	0	1	1	1	1	8.3	0.15	16.9
1	0	0	100	2.67	1	68	25.7	129.5	90.5	174	40	134	13.5	5.7	78.8	2	0	0	0	1	1	1	11.1	0.13	19.7
1	0	0	100	5.37	1	70	27.6	159.5	103	154	40	114	14.5	7.2	70.1	2	1	0	0	1	0	1	109.4	0.16	40.3
1	0	0	100	6.88	1	70	22.7	147	78	167	40	127	14.3	4.8	70.1	2	1	1	0	1	1	1	5.4	0.03	41
1	0	0	100	5.16	1	72	23	111	76	183	40	143	14.5	5.7	69.1	2	0	1	1	1	1	1	6.4	0.01	26.4
1	0	0	100	5.16	1	66	24.4	129	71.5	143	40	103	14.3	5.2	75.7	2	1	0	0	1	1	1	5.1	0.08	57.7
1	0	0	100	6.85	1	68	23.2	123	80	173	40	133	14.4	5.2	71.1	2	1	0	1	1	1	1	8.9	0.09	0.05
1	0	0	100	7.21	1	78	21.4	99.5	58	148	40	108	13.6	5.2	69.6	1	1	1	0	1	1	1	24.3	0.07	29.3
1	0	0	100	7.01	1	75	21.5	120.5	71	145	40	105	14.5	4.4	80.0	2	1	1	1	1	1	1	19.5	0.03	29.2
1	0	0	100	5.06	1	74	20.1	101	68	147	40	107	12.7	5.6	68.2	2	0	0	1	1	1	1	7	0.07	28.8
1	0	0	100	5.08	1	70	24.9	127.5	80.5	189	40	149	15.8	5.4	73.6	2	1	1	1	1	1	1	8.1	0.05	5.95
1	0	0	100	6.97	1	75	20.4	145	82	175	40	135	12	5.5	67.7	2	1	1	0	1	1	1	18.7	0.01	38.2
1	0	0	100	4.61	1	66	25.4	130	75	146	40	106	15.6	5.2	75.7	2	1	0	0	1	1	1	13.4	0.04	45.3
1	0	0	100	4.62	1	79	23.3	155	85.5	201	40	161	14.4	5.4	65.8	2	1	1	0	1	1	1	24.2	0.05	50.8
1	0	0	100	6.44	1	65	21.3	143	78	218	40	178	15.7	5.4	80.5	2	0	1	0	1	1	1	19.3	0.06	16.2
1	0	0	100	5.36	1	70	23.1	153	77.5	182	40	142	13.8	5.4	77.7	2	1	0	0	1	1	1	31.5	0.01	116
1	0	0	100	3.15	1	67	27	147	85	172	40	132	14.4	5.6	84.6	2	1	0	0	1	1	1	38.9	0.02	20.3
1	1	0	100	6.87	1	68	26	147.5	82.5	151	40	111	13.5	5.6	78.8	2	0	0	0	1	1	1	19.4	0.59	202
1	1	0	100	6.86	1	78	26.4	138	78.5	182	40	142	14.4	5.3	66.3	2	1	1	1	1	1	1	9	0.07	0.05
1	0	0	100	7	2	69	27.3	153.5	81.5	232	40	192	13.1	5.5	75.9	1	1	1	0	0	1	1	2063.3	0.14	51.7
1	0	0	100	5.05	2	71	24.3	106	65.5	162	40	122	13.1	5.8	71.1	1	1	1	1	1	1	1	31.4	0.1	16.8
1	0	0	100	5.07	2	67	22.6	136.5	82	191	40	151	11.9	5.4	81.7	2	1	1	1	1	1	1	49.6	0.1	15.8
1	0	0	100	4.63	2	68	29.5	145.5	91	190	40	150	14	5.4	76.4	2	1	1	0	1	1	1	40.5	0.15	21.6
1	0	0	100	6.8	2	69	22.8	95.5	66.5	275	40	235	14.1	5.8	61.4	2	0	1	1	0	1	1	8.9	0.17	5.17
1	0	0	100	6.8	2	72	27	150	77.5	302	40	262	13.5	5.9	70.6	2	1	1	0	0	1	1	378.6	0.29	16.9
1	0	0	100	4.62	2	69	26.7	150.5	86	224	40	184	14.5	6.2	75.9	2	1	1	0	0	1	1	210	0.17	21.7
1	0	0	100	4.69	2	67	22.9	150.5	77	191	40	151	13.7	5.7	76.9	2	1	0	0	1	1	1	11.7	0.2	29.1
1	0	0	100	7	2	67	30.5	144.5	91	189	40	149	14.5	5	81.7	2	1	0	0	1	1	1	15	0.05	16.2
1	0	0	100	0.44	2	69	24.2	132	86	253	40	213	13.7	5	80.5	2	1	1	1	0	1	1	41.7	0.09	12.9

1	0	0	100	6.86	2	67	17.9	131.5	81.5	132	40	92	12.1	5.1	76.9	2	1	1	1	1	1	1	20.1	0.01	9.67
1	0	0	100	6.84	2	70	25.8	123.5	65.5	188	40	148	13.2	5.2	80.0	1	1	1	0	1	1	1	33	0.05	24.8
1	0	0	100	7.21	2	68	27.2	126	84.5	223	40	183	12.6	5.4	72.6	2	1	1	1	0	1	1	13.1	0.03	19.1
1	0	0	100	7.02	2	67	24.5	128	77	293	40	253	12	6	76.9	2	1	1	1	0	1	1	23.3	0.02	19.2
1	0	0	100	5.06	2	66	26.6	119.5	72	192	40	152	13.7	5.4	82.3	2	1	1	0	1	1	1	22.7	0.03	27.9
1	0	0	100	5.06	2	68	27	142	85.5	204	40	164	14.1	6	81.1	2	1	1	0	1	1	1	34.9	0.02	16.7
1	0	0	100	5.07	2	67	25.2	130.5	79	177	40	137	12.9	5.7	76.9	2	1	1	1	1	1	1	9.6	0.05	57.3
1	0	0	100	5.08	2	69	27.1	163	93.5	192	40	152	13.5	5.2	72.1	2	1	1	0	1	1	1	7.6	0.03	22.4
1	0	0	100	4.64	2	66	27.9	125	72	212	40	172	12.1	8.7	77.5	2	1	1	0	1	0	1	31	0.03	15.8
1	0	0	100	4.64	2	77	22.8	112.5	59	223	40	183	12.2	5.5	71.7	1	1	1	0	0	1	1	411.8	0.05	65.9
1	0	0	100	6.8	2	68	24.9	131	71	214	40	174	13.2	5.7	81.1	2	1	0	1	1	1	1	42.7	0.08	11.3
1	0	0	100	5.37	2	70	27.9	113	62.5	266	40	226	12.1	5.2	75.3	1	1	1	1	0	1	1	23	0.03	27.9
1	0	0	100	6.87	2	76	23.1	130	67.5	130	40	90	10.7	5.3	72.2	2	1	1	1	1	1	1	513.9	0.03	56.5
1	1	0	100	4.53	2	73	29.5	162.5	82.5	172	40	132	10.6	5	45.5	1	1	1	0	1	1	1	28.1	0.35	16.9
1	1	0	100	6.87	2	70	18.9	95.5	58.5	166	40	126	12.2	5.3	86.1	1	1	1	1	0	1	1	82.3	0.13	42.1
1	1	0	100	6.39	2	79	30	127.5	74	173	40	133	12.9	5.8	70.7	1	1	1	1	0	1	1	16.5	0.08	43.6
1	0	0	100	6.8	1	80	20.9	123.5	72.5	204	40	164	15.2	5.7	65.4	2	1	1	1	1	1	1	6.4	0.42	21.6
1	0	0	100	3.2	2	81	20.4	153	81	244	40	204	13	5.4	69.7	2	1	1	0	0	1	1	266.3	0.05	22.2
1	0	0	100	5.16	1	67	26.2	124	89	150	40	110	14.4	5.8	75.2	2	1	1	0	1	1	1	16.3	2.35	15.9
1	0	0	100	6.98	1	74	21.4	165.5	95.5	277	40	237	13.5	5.5	68.2	2	0	0	0	0	1	1	3.8	0.35	4.83
1	0	0	100	6.87	1	74	23.4	138.5	72.5	217	40	177	13.6	5.5	71.5	2	0	1	1	1	1	1	21.7	0.25	14.2
1	0	0	100	1.69	1	74	25.7	134.5	88	203	40	163	14.5	5.7	75.6	2	1	1	0	1	1	1	37.1	0.16	11.9
1	0	0	100	6.86	1	72	22.3	108	61	227	40	187	14.2	6.4	76.7	2	1	0	1	0	1	1	10.8	0.05	7.63
1	0	0	100	6.84	1	73	17.4	120.5	77	158	40	118	13.3	5.7	72.0	2	1	1	1	1	1	1	12.5	0.01	39.6
1	0	0	100	4.59	1	78	24.5	118	74	217	40	177	14.8	5.1	52.0	2	0	1	1	1	1	1	4.2	0.05	33.1
1	0	0	100	6.87	2	65	26	160.5	98.5	242	40	202	14.1	6	78.0	2	1	1	0	0	1	1	47.8	0.11	8.88
1	0	0	100	4.6	2	73	24.4	110	63	170	40	130	13	5.8	73.8	2	1	1	0	1	1	1	7.9	0.1	9.57
1	0	0	100	5.06	2	74	26.8	125	71	237	40	197	14.1	5.8	69.6	2	1	1	1	0	1	1	10.6	0.17	117
1	0	0	100	5.06	2	71	25.7	119.5	75.5	213	40	173	13.5	6.1	60.5	2	1	0	0	0	1	1	10.6	0.23	10.6
1	0	0	100	7.07	2	66	28	150	83	201	40	161	14.2	5.2	62.7	1	1	1	0	0	1	1	47.1	0.07	10.2
1	0	0	100	7	2	72	24	117	68.5	165	40	125	12.4	4.9	70.6	2	1	1	0	1	1	1	30.3	0.02	54.1
1	0	0	100	5.08	2	65	25.2	119.5	69.5	182	40	142	12.8	5.4	78.0	2	1	1	0	1	1	1	17.2	0.08	27.8
1	0	0	100	7	2	68	20.8	98	64	192	40	152	12.7	5.1	72.6	2	1	1	1	1	1	1	6.7	0.06	2.58
1	0	0	100	0.94	2	66	22.1	140.5	71	152	40	112	12.3	5	82.3	2	1	1	0	1	1	1	191.8	0.05	68.1
1	1	0	100	4.64	2	76	28.9	126	84	210	40	170	13.1	5	58.4	2	1	1	0	1	1	1	29	0.1	36.3
1	1	0	100	6.8	2	73	24.1	123.5	85.5	218	40	178	12.4	5.3	78.3	2	1	1	0	1	1	1	17.8	0.06	21.7
1	1	0	100	6.86	2	81	24.4	151	68	222	40	182	11.5	5.7	66.3	1	1	1	0	0	1	1	22.3	0.13	83.6
1	1	0	100	6.87	2	80	18.3	94.5	64.5	152	40	112	10.6	4.7	74.6	2	1	1	1	1	1	1	16.7	0.73	121
1	0	1	4.7	4.7	1	74	22	121.5	71.5	196	40	156	15	5.6	68.2	1	1	1	1	1	1	1	13.5	0.11	248
1	0	1	2.3	2.3	1	83	28.6	125.5	62.5	185	40	145	14.7	7.2	64.0	2	1	0	0	1	0	1	53.9	0.07	28.1
1	1	1	1.4	1.4	2	76	20.9	144	84.5	167	40	127	13.1	5.2	76.7	1	1	1	0	1	1	1	68.9	0.05	21.1
1	0	0	100	7	1	67	25.1	123	65	194	41	153	15.4	5.1	63.0	2	0	1	1	1	1	1	22.2	0.4	176
1	0	0	100	4.58	1	68	24.8	148.5	99	174	41	133	15.1	6.2	74.6	2	1	0	0	1	1	1	16.7	0.1	18.2
1	0	0	100	4.58	1	72	24.6	143.5	87.5	211	41	170	15.3	5.7	69.1	2	1	0	0	1	1	1	21.3	0.14	38.7
1	0	0	100	4.59	1	72	25.8	161	86.5	179	41	138	14.7	6.6	69.1	2	0	0	0	1	0	1	21.3	0.38	2.1
1	0	0	100	6.87	1	74	24.8	144	91.5	232	41	191	13	5.7	60.0	2	1	1	0	0	1	1	3	0.1	22.8
1	0	0	100	7.01	1	65	20.8	156.5	101	140	41	99	15.2	5.3	80.5	2	0	0	0	1	1	1	7.1	0.35	16.9
1	0	0	100	6.81	1	65	23.9	120.5	69	180	41	139	14.8	5.9	80.5	2	1	0	1	1	1	1	18.8	0.19	37.8
1	0	0	100	7.02	1	69	22.3	131	73.5	192	41	151	13.1	5.8	74.1	2	1	1	1	1	1	1	10.7	0.66	16.8
1	0	0	100	6.8	1	72	23.8	238.5	127.5	222	41	181	16.1	5.4	72.6	2	1	1	0	0	1	1	41.8	0.17	11.3
1	0	0	100	1.32	1	72	27.2	162	89	258	41	217	16.3	5.6	72.6	2	1	0	0	0	1	1	14.8	0.32	5.93

1	0	0	100	4.59	1	79	22.2	111.5	63	205	41	164	13.2	5	57.9	2	1	1	1	1	1	1	2.6	0.24	44.1
1	0	0	100	6.38	1	70	24.1	156	79.5	226	41	185	13.5	9.6	73.6	2	1	0	0	0	0	1	2053.4	0.26	18.3
1	0	0	100	4.69	1	65	26.8	113	77	217	41	176	13.6	5.4	85.8	2	1	1	1	1	1	1	12.4	0.13	19.6
1	0	0	100	1.71	1	71	21.1	110.5	67	179	41	138	13.8	6	69.6	2	0	1	1	1	1	1	11.5	0.12	17.6
1	0	0	100	5.05	1	71	24.1	119	75.5	165	41	124	13.8	5.6	82.2	2	1	1	1	1	1	1	14	0.31	47.5
1	0	0	100	7	1	71	26.2	125	67.5	192	41	151	14.7	5.8	73.1	1	0	1	1	1	1	1	5.5	0.02	17
1	0	0	100	7.06	1	74	23.6	122.5	68	191	41	150	13.6	5.7	75.6	2	1	1	1	1	0	1	6.7	0.06	74.3
1	0	0	100	7.08	1	66	25.9	118.5	65	241	41	200	15	4.9	79.9	2	1	1	1	0	1	1	17.1	0.07	5.02
1	0	0	100	7.08	1	70	23.1	121.5	82.5	197	41	156	16	5.9	77.7	2	1	0	1	1	1	1	7.7	0.06	20.1
1	0	0	100	4.58	1	76	27.4	143	75	181	41	140	15	5.7	67.2	2	0	0	0	1	1	1	7.4	0.07	31.5
1	0	0	100	6	1	73	22.3	146.5	87	175	41	134	16.5	6.2	68.6	2	0	0	0	1	1	1	43.8	0.03	10
1	0	0	100	6.85	1	71	24.3	117.5	74.5	222	41	181	14.8	6	69.6	2	1	0	0	0	1	0	15.1	0.07	72.4
1	0	0	100	7.01	1	65	24.7	159	92.5	142	41	101	13.3	5.2	76.2	2	1	0	0	1	1	1	5.1	0.01	22.3
1	0	0	100	7.01	1	66	22.2	124	76	246	41	205	14.3	5.9	79.9	2	1	1	1	0	1	1	21.4	0.01	18
1	0	0	100	7.04	1	72	20.8	98.5	59	150	41	109	13.1	5.6	69.1	2	0	1	1	1	1	1	9.2	0.01	8.57
1	0	0	100	5.06	1	78	21.7	127	62.5	168	41	127	15.4	5.8	73.5	2	0	1	1	1	1	1	13.7	0.07	8.28
1	0	0	100	5.07	1	73	27.5	147	84	186	41	145	13.4	5.9	76.1	1	1	1	0	0	1	1	58.4	0.03	37.5
1	0	0	100	5.07	1	68	24.8	159.5	88.5	153	41	112	13.7	7.4	84.0	2	0	0	0	1	0	1	32.5	0.07	20.6
1	0	0	100	5.08	1	73	24.1	129	73.5	176	41	135	14.4	5.7	60.4	2	0	1	1	1	1	1	39.6	0.08	5.9
1	0	0	100	6.98	1	73	21.2	130	76	252	41	211	16.9	5.5	60.4	2	1	1	1	0	1	0	9.5	0.06	39.4
1	0	0	100	6.98	1	68	26.1	153.5	84.5	186	41	145	14.5	5.4	78.8	2	0	1	0	1	1	1	40.2	0.06	19.8
1	0	0	100	6.87	1	71	25.1	133	81	150	41	109	16.5	6	77.2	2	0	1	1	0	1	1	105.5	0.04	26
1	0	0	100	6.98	1	69	24.9	132	77	184	41	143	14.3	5.3	70.6	2	1	0	1	1	1	1	7.6	0.05	35.2
1	0	0	100	6.83	1	66	21.9	123.5	81	157	41	116	14.9	5.9	75.7	2	0	0	1	1	1	1	13.3	0.04	12.1
1	0	0	100	4.68	1	66	25.3	128.5	85.5	167	41	126	12.8	5.5	72.1	2	1	0	1	1	1	1	9	0.02	4.39
1	0	0	100	6.96	1	65	22.7	137	84	184	41	143	14.8	5.5	63.9	2	1	0	1	1	1	1	10.4	0.09	10.9
1	0	0	100	5.05	1	75	22.1	175	100.5	144	41	103	13.9	5.1	67.7	2	0	1	0	1	1	1	15.5	0.03	55.2
1	0	0	100	5.06	1	74	25.7	117	62	186	41	145	13.6	6	60.0	2	1	0	0	1	1	1	3.9	0.02	4.79
1	0	0	100	6.85	1	66	25.1	122.5	81.5	170	41	129	15	5	63.5	2	1	0	1	1	1	1	25.5	0.01	5.32
1	0	0	100	6.86	1	73	25.8	138.5	72	150	41	109	14.7	5.2	76.1	2	0	0	0	1	1	1	32.1	0.09	9.18
1	1	0	100	4.6	1	73	23	190	86.5	161	41	120	11.9	6.2	68.6	2	0	0	0	1	1	1	46	0.35	22.1
1	1	0	100	7.04	1	66	18.5	129	84.5	110	41	69	12.8	5.1	79.9	1	1	1	1	1	1	1	7.6	0.05	11.8
1	0	0	100	6.87	2	67	31.4	117.5	69.5	257	41	216	15.1	11.6	81.7	2	1	1	1	0	0	1	105.1	0.48	28.8
1	0	0	100	7.21	2	65	21	137	75	180	41	139	12.4	7.4	63.1	2	1	1	0	1	0	1	9	0.66	40.2
1	0	0	100	6.8	2	72	25	106	73.5	159	41	118	12.6	5.6	60.1	2	1	1	0	1	1	1	16.9	0.11	22.6
1	0	0	100	4.68	2	72	24.5	124	72.5	189	41	148	13.7	5.1	74.3	1	1	1	1	1	1	1	56.3	0.3	40.9
1	0	0	100	6.86	2	73	24.9	97	59.5	208	41	167	10.6	5.5	78.3	1	1	1	1	1	1	1	28.5	0.25	21.1
1	0	0	100	6.86	2	69	25	127.5	64	212	41	171	12.9	5.7	80.5	2	1	1	1	1	1	1	28	0.4	36
1	0	0	100	7.08	2	69	26.3	164	86.5	174	41	133	13.6	5.5	75.9	2	1	1	0	1	1	1	10.2	0.04	40.3
1	0	0	100	5.16	2	74	27.6	149	80	194	41	153	11.8	5.3	69.6	2	1	1	0	1	1	1	18.7	0.04	12.9
1	0	0	100	6.86	2	65	29.8	133.5	76.5	259	41	218	13.1	5.7	78.0	2	1	1	1	0	1	1	9	0.04	37
1	0	0	100	4.35	2	70	24.3	120	69.5	160	41	119	11.8	5.5	80.0	1	1	1	1	1	1	1	13.4	0.02	31.4
1	0	0	100	5.06	2	66	27.4	117	67	144	41	103	13.4	5.6	62.7	2	1	1	0	1	1	1	5.5	0.04	22.7
1	0	0	100	4.63	2	75	20.4	98	60	213	41	172	10.6	5.6	51.0	2	1	1	1	1	1	1	15.1	0.01	25.7
1	0	0	100	4.64	2	68	24.6	120.5	62	226	41	185	13.4	5.7	76.4	2	1	1	1	0	1	1	19	0.02	6.6
1	0	0	100	6.96	2	65	17.7	109.5	76	165	41	124	12.1	4.5	78.0	2	1	1	1	1	1	1	12.3	0.02	8.53
1	0	0	100	6.79	2	78	27.6	135	69.5	239	41	198	13.4	6	67.7	1	1	1	1	0	1	1	8.8	0.09	4.14
1	0	0	100	6.84	2	70	22.7	118.5	62.5	289	41	248	11.4	5.8	37.3	1	1	1	1	0	1	1	11.3	0.06	22.7
1	0	0	100	4.62	2	72	26.7	158.5	90.5	200	41	159	13.1	4.9	70.6	1	1	1	0	1	1	1	11	0.03	41.1
1	0	0	100	5.05	2	68	17.2	126	68.5	203	41	162	13.8	5.2	61.8	2	1	1	1	1	1	1	28.3	0.06	17.7
1	0	0	100	6.85	2	70	20.1	160.5	86	151	41	110	13.7	5.2	75.3	2	1	1	0	1	1	1	22.7	0.07	50.8

1	0	0	100	6.85	2	66	20.9	147	93	241	41	200	12.9	5.3	62.7	2	1	1	0	0	1	1	1508.5	0.02	44.5
1	0	0	100	6.86	2	71	22.7	105	57.5	196	41	155	13.1	5.8	79.4	2	1	1	1	1	1	1	16.2	0.08	12
1	0	0	100	6.86	2	69	23.3	143	76.5	195	41	154	14.2	4.9	75.9	1	1	1	0	1	1	1	55.2	0.07	25
1	0	0	100	1.77	2	68	25.6	159	100	200	41	159	13	5.1	72.6	2	1	1	0	1	1	1	28.5	0.05	18.7
1	0	0	100	6.87	2	70	27.3	132.5	85.5	197	41	156	12.9	5.5	80.0	2	1	1	0	1	1	1	40.3	0.05	22.1
1	1	0	100	4.93	2	70	22.9	145.5	88	195	41	154	13.9	5.6	75.3	2	1	1	0	1	1	1	5.7	0.04	28.4
1	1	0	100	6.8	2	68	23.7	116	57.5	162	41	121	11.4	5.8	61.8	1	1	1	0	1	1	1	66.9	0.05	87.6
1	0	0	100	0.42	1	83	23.9	161	85	181	41	140	13.5	5.4	56.3	2	0	1	0	1	1	1	6.1	0.7	61.2
1	0	0	100	0.7	1	85	20.9	128.5	73	230	41	189	13.2	5.5	63.1	2	1	1	1	0	1	1	37.8	0.02	35.5
1	0	0	100	4.63	2	80	26.9	150	73	221	41	180	11.1	5.3	66.7	2	1	1	0	0	1	1	25.9	0.04	31.5
1	0	0	100	6.85	1	76	22.1	154.5	83.5	182	41	141	14.4	5	59.2	2	1	1	0	1	1	1	119	0.12	30.1
1	0	0	100	6.97	1	74	25.7	120	80.5	185	41	144	14.2	5.2	71.5	2	1	0	0	1	1	1	8.1	0.2	12.9
1	0	0	100	5.07	1	72	25.4	125.5	72	143	41	102	14.4	5.4	72.6	2	1	1	1	1	1	1	7.2	0.6	58.1
1	0	0	100	6.86	1	71	18.3	117	65.5	182	41	141	14.1	5	77.2	2	0	0	1	1	1	1	16.7	0.06	23.8
1	0	0	100	5.16	1	71	28.8	107.5	66.5	170	41	129	14.9	5.5	69.6	2	1	1	0	1	1	1	18.7	0.02	20.5
1	0	0	100	6.84	1	69	24.1	102.5	64	219	41	178	15.4	5	70.6	2	1	1	1	1	1	1	5.8	0.02	43.4
1	0	0	100	4.62	1	72	21.7	136	78.5	235	41	194	15.6	5.2	72.6	2	1	1	0	0	1	0	92.9	0.07	184
1	0	0	100	6.82	1	79	20.9	138.5	76.5	199	41	158	12.7	5.3	69.1	2	1	1	1	1	1	1	10.2	0.04	28.7
1	0	0	100	6.85	1	78	22.5	129.5	68.5	147	41	106	13.8	6.4	69.6	2	1	1	1	1	0	1	11.5	0.02	52.9
1	1	0	100	6.42	1	75	23.2	137.5	73	159	41	118	12.5	5.9	75.1	1	1	1	1	1	1	1	14.7	0.01	13.5
1	1	0	100	6.85	1	74	19.6	135	77	154	41	113	13.8	5.8	60.0	2	1	1	1	1	1	1	194.8	0.03	14.4
1	0	0	100	7.04	2	72	29.3	150	79	261	41	220	14.9	5.8	70.6	1	1	1	0	0	1	1	5.9	0.11	0.86
1	0	0	100	5.06	2	72	30	160	83	255	41	214	13.2	5.8	70.6	1	1	1	0	0	1	1	12.1	0.11	18
1	0	0	100	6.98	2	75	31.5	128	72	175	41	134	13.8	5.5	72.7	2	1	1	0	1	1	1	9.9	0.41	30
1	0	0	100	7.07	2	77	25.1	121.5	70.5	174	41	133	12	5.2	76.1	1	1	1	0	1	1	1	25.8	0.03	19.5
1	0	0	100	7.07	2	70	29.1	144	89.5	224	41	183	14.2	5	75.3	2	1	1	0	0	1	1	22.2	0.04	0.05
1	0	0	100	6.86	2	77	22.6	162	81	166	41	125	13.2	5.1	68.2	1	1	1	0	1	0	1	11.3	0.02	22.8
1	0	0	100	5.16	2	67	27.8	108.5	71.5	206	41	165	13.2	5.3	62.2	2	1	1	0	1	1	1	18.5	0.02	6.79
1	0	0	100	6.85	2	73	27.6	122.5	66	214	41	173	13.3	6	78.3	2	1	1	1	0	1	1	13.3	0.04	77.5
1	0	0	100	6.99	2	70	28.7	124.5	74.5	206	41	165	11.3	5.1	80.0	1	1	0	0	1	1	1	219.2	0.09	19
1	0	0	100	5.06	2	68	24.3	148	89.5	197	41	156	11.5	5.8	81.1	2	1	0	0	1	1	1	15.5	0.01	28.9
1	0	0	100	4.63	2	66	21	136.5	76.5	148	41	107	12.6	5.9	77.5	2	1	1	0	1	1	1	7.2	0.01	12.1
1	0	0	100	6.8	2	65	28.9	105	63.5	181	41	140	14	6.1	74.2	2	1	1	0	1	1	1	29.6	0.07	9.28
1	0	0	100	4.62	2	75	25.4	152	84	231	41	190	12.7	6.3	58.8	1	1	1	0	0	1	1	13.5	0.02	16.2
1	1	0	100	7.06	2	68	26.9	145.5	82.5	194	41	153	14.6	5.5	76.4	2	1	1	0	1	1	1	75.1	0.12	25.4
1	1	0	100	6.96	2	78	29.2	117.5	66.5	185	41	144	12.3	5.7	75.6	1	1	1	1	1	1	1	15	0.02	10.2
1	1	0	100	5.37	2	74	28.5	140	82	176	41	135	12.9	5.3	73.2	1	1	1	0	1	1	1	9.5	0.05	46.5
1	1	0	100	5.06	2	76	25.4	149	77	252	41	211	13.1	5.6	39.7	2	1	1	0	0	1	1	27.4	0.01	23.2
1	1	0	100	6.85	2	82	27.1	151	78	244	41	203	13.1	6.7	69.2	1	1	1	0	0	0	1	44.8	0.06	78.1
1	0	1	1.4	1.4	1	66	19.3	94.5	62.5	142	41	101	13.4	5	75.7	2	1	1	1	1	1	1	12	0.48	82.5
1	0	1	2.9	2.9	1	67	24.4	121.5	68.5	156	41	115	14	6	63.0	2	1	0	0	1	1	0	68.9	0.2	80.5
1	0	1	2.6	2.6	1	70	25.8	121.5	72	199	41	158	15.1	5.5	82.8	2	1	1	1	1	1	1	6.6	0.03	34.9
1	0	1	3.1	3.1	1	69	27.1	150	86.5	181	41	140	15.3	5.4	74.1	2	1	0	0	1	1	1	5.9	0.09	18.4
1	0	1	3.3	3.3	2	71	20.7	113	67.5	161	41	120	11	6	52.5	2	1	1	0	1	1	1	4.7	0.29	43.7
1	1	1	0.8	0.8	2	77	29	136	66	224	41	183	13.1	5.7	68.2	2	1	1	0	0	1	1	32.9	0.11	18.8
1	1	1	2	2	2	78	23.8	161	86.5	237	41	196	13.6	6.5	67.7	1	1	1	0	0	0	1	14.2	0.21	43.8
1	0	0	100	4.02	1	69	23.4	161.5	94.5	177	42	135	12.3	5.4	70.6	2	1	1	0	1	1	1	29.8	0.28	8.04
1	0	0	100	6.85	1	74	23.9	120.5	81.5	216	42	174	14.7	5.6	60.0	2	1	0	1	1	1	1	7.9	0.13	9.83
1	0	0	100	7.02	1	67	26.2	126.5	80.5	199	42	157	14.7	5.9	50.6	2	0	1	1	1	1	1	10.5	0.76	8.61
1	0	0	100	5.08	1	77	25	218.5	109.5	192	42	150	13.3	5.3	66.7	2	1	0	0	1	1	1	81.5	0.11	39.4
1	0	0	100	6.98	1	68	18.2	152	79.5	186	42	144	16.1	6.3	74.6	2	0	0	0	1	1	1	53.8	0.25	25

1	0	0	100	6.8	1	75	23.9	98	67	167	42	125	13.7	5.8	67.7	2	1	0	1	1	1	1	4.3	0.42	127
1	0	0	100	4.62	1	71	22.7	162	83	132	42	90	11.9	6.2	73.1	2	1	0	0	1	1	1	57.6	0.18	17.2
1	0	0	100	4.69	1	74	25.4	147.5	86.5	135	42	93	15.6	5	68.2	2	1	0	0	1	1	1	3	0.12	34.7
1	0	0	100	5.36	1	69	24.9	137	94	193	42	151	15.7	5.3	55.4	2	1	0	0	1	1	1	158	0.12	39
1	0	0	100	5.06	1	65	24.8	132.5	90.5	198	42	156	15.4	5.4	76.2	2	0	0	0	1	1	1	9.6	0.26	32.7
1	0	0	100	6.44	1	68	22.9	85	49.5	187	42	145	14.2	5.9	90.5	2	1	1	1	1	1	1	8.7	0.66	23.8
1	0	0	100	1.93	1	74	26.4	158	89.5	216	42	174	16.9	5.5	75.6	2	1	0	0	1	1	1	981.5	0.11	78.2
1	0	0	100	7.06	1	72	26.3	122	78	205	42	163	14.4	5	76.7	1	1	0	0	1	1	1	22.8	0.05	25.4
1	0	0	100	7.42	1	71	21.7	95.5	61.5	174	42	132	12	5.3	73.1	2	1	0	1	1	1	1	10.8	0.01	82.3
1	0	0	100	4.58	1	71	25.5	166.5	87	145	42	103	14.7	5.3	69.6	2	1	0	0	1	1	1	11.5	0.03	41.5
1	0	0	100	6.88	1	66	21.9	100.5	67	173	42	131	14.6	5.1	79.9	2	1	0	1	1	1	1	6.7	0.01	27.1
1	0	0	100	5.16	1	68	24	154.5	72.5	163	42	121	13.2	5.1	74.6	2	1	0	0	1	1	1	22.7	0.02	21.2
1	0	0	100	6.84	1	70	24.7	111	63.5	147	42	105	13.9	5.1	70.1	2	1	0	0	1	1	1	14.3	0.04	36.7
1	0	0	100	6.84	1	72	24.1	117	73	170	42	128	15.8	4.9	72.6	2	1	1	1	1	1	1	8.2	0.03	7.67
1	0	0	100	6.85	1	66	22.7	113	72	157	42	115	14.8	4.8	79.9	2	0	0	1	1	1	1	37.6	0.03	27.1
1	0	0	100	6.85	1	65	22.8	131.5	81.5	162	42	120	14.8	5.2	76.2	2	1	0	1	1	1	1	6.1	0.02	10.4
1	0	0	100	6.86	1	67	19.1	101	60.5	195	42	153	13.5	5.6	84.6	2	1	0	1	1	1	1	5.2	0.06	20.7
1	0	0	100	7.01	1	65	29.8	153	96	155	42	113	16.8	5.5	80.5	2	1	0	0	1	1	1	10	0.05	1.34
1	0	0	100	7.01	1	72	22	148.5	79.5	171	42	129	13.3	5.1	81.7	1	1	1	0	1	1	1	4.9	0.05	22.9
1	0	0	100	5.05	1	65	30.5	133.5	85	192	42	150	14.6	5.7	76.2	2	0	1	1	1	1	1	16.2	0.04	15.8
1	0	0	100	5.07	1	79	25	95	64.5	181	42	139	14.2	5.5	69.1	2	1	1	1	1	1	1	6.6	0.06	15.5
1	0	0	100	6.97	1	65	23.5	106	73.5	123	42	81	10.4	4.6	80.5	2	1	1	1	1	1	1	10.1	0.02	21.9
1	0	0	100	7	1	65	25.1	118	78.5	190	42	148	13.3	5.2	76.2	2	0	0	1	1	1	1	5.4	0.08	21.5
1	0	0	100	4.63	1	74	23.6	130	74.5	167	42	125	15.1	5.2	68.2	2	0	0	1	1	1	1	10.7	0.01	21.6
1	0	0	100	4.64	1	65	25.7	159.5	110	133	42	91	16.2	5.3	76.2	2	1	0	0	1	1	1	7.3	0.04	16.6
1	0	0	100	6.8	1	79	21.2	112	69.5	160	42	118	11.8	5.7	69.1	2	1	0	1	1	1	1	6.7	0.05	25.4
1	0	0	100	6.82	1	70	25.1	128	80.5	182	42	140	16.1	5.5	77.7	2	1	0	1	1	1	1	7.9	0.03	16.3
1	0	0	100	6.44	1	75	22.2	136.5	87.5	150	42	108	14.9	5.8	71.0	2	1	1	1	1	1	1	17.7	0.02	8.61
1	0	0	100	5.37	1	73	24.7	130.5	74	156	42	114	14.6	5.3	72.0	1	1	0	1	1	1	1	13	0.01	6.34
1	0	0	100	6.86	1	72	21.8	125.5	66.5	165	42	123	12.1	5.6	88.0	2	0	1	1	1	1	1	15.3	0.07	52.3
1	0	0	100	2.86	1	65	23.3	161	88.5	223	42	181	15	5.1	72.6	2	1	0	0	0	1	1	4.1	0.06	12.6
1	0	0	100	6.87	1	77	21.1	179	86.5	171	42	129	14.2	5.2	74.0	2	0	1	0	1	1	1	28	0.07	25.7
1	1	0	100	5.3	1	76	23	160	81	195	42	153	14.4	4.9	52.7	1	0	1	0	1	1	1	286.2	0.45	54.5
1	1	0	100	5.37	1	78	23.3	157	88	160	42	118	14.2	5.2	58.3	2	1	1	0	1	1	1	13.5	0.01	89.8
1	0	0	100	6.86	2	67	26.3	128.5	68	224	42	182	11.8	5.6	73.1	1	1	1	0	0	1	1	20.1	0.12	31.9
1	0	0	100	7.01	2	69	27.3	112	65.5	190	42	148	13.7	5.2	72.1	2	1	1	1	1	1	1	8.4	0.13	7.83
1	0	0	100	6.8	2	74	25.8	134	82.5	207	42	165	13.2	5.8	69.6	2	1	1	0	1	1	1	25.7	0.15	12.7
1	0	0	100	2.8	2	78	30.5	135	75	206	42	164	13.7	5.3	67.7	1	1	1	0	1	1	1	16.7	0.42	2.36
1	0	0	100	4.59	2	73	26.7	156	74	247	42	205	12.7	5.7	51.7	1	1	1	0	0	1	1	20.8	0.13	75.4
1	0	0	100	4.6	2	69	23.9	128.5	68.5	285	42	243	13.4	6.2	72.1	2	1	1	1	0	1	1	25.3	0.13	18.6
1	0	0	100	4.69	2	69	27.6	138.5	78	191	42	149	13.3	5.6	75.9	2	1	0	1	1	1	1	59.4	0.11	16.3
1	0	0	100	6.87	2	66	29.2	131.5	83.5	167	42	125	13.7	5.7	73.6	2	1	0	1	1	1	1	1863.4	0.15	13.9
1	0	0	100	6.87	2	72	26.3	162	85	172	42	130	12.1	5	78.9	1	1	1	0	1	1	1	22	0.18	79.2
1	0	0	100	7.06	2	77	25.3	171.5	92	220	42	178	12.5	5.4	68.2	1	1	1	0	0	1	1	74.1	0.06	45.2
1	0	0	100	4.58	2	69	22.2	101.5	60	220	42	178	14.7	5.7	80.5	2	1	1	0	0	1	1	36.7	0.03	3.54
1	0	0	100	4.59	2	67	27.6	143	70	196	42	154	13.5	5.8	76.9	2	1	1	0	1	1	1	226.1	0.07	26.3
1	0	0	100	6.87	2	71	26.1	138	84	245	42	203	12	6	74.8	2	1	1	0	0	1	1	34.1	0.01	11.9
1	0	0	100	6.87	2	75	27.5	149	94	254	42	212	12.6	5.6	69.1	2	1	1	0	0	1	1	39.9	0.02	39.9
1	0	0	100	6.84	2	68	22.7	135	90	184	42	142	12.6	5.5	72.6	2	1	1	0	1	1	1	5.4	0.08	26.5
1	0	0	100	6.85	2	65	32.8	113.5	67.5	172	42	130	12.2	5.2	78.0	2	1	1	1	1	1	1	11.3	0.06	40.5
1	0	0	100	6.99	2	69	30.3	148.5	86	163	42	121	12.4	4.9	80.5	1	1	0	0	1	1	1	12.2	0.08	39.3

1	0	0	100	4.36	2	71	25.1	132.5	70	178	42	136	13.8	5.4	74.8	2	1	1	1	1	1	1	25.2	0.03	42.5
1	0	0	100	4.63	2	69	21.6	161.5	89.5	176	42	134	14.1	5	72.1	2	1	1	0	1	1	1	27.3	0.04	25.1
1	0	0	100	5.01	2	71	20.8	117.5	68	243	42	201	12.8	5.3	79.4	2	1	1	1	0	1	1	18.4	0.08	17
1	0	0	100	6.8	2	65	26.2	141	83	233	42	191	13.2	5.2	54.7	2	1	0	0	0	1	1	157.7	0.06	5.77
1	0	0	100	6.8	2	69	25.8	141.5	70.5	227	42	185	14.8	5.6	75.9	2	1	1	0	0	1	1	22.1	0.08	25.6
1	0	0	100	4.59	2	66	24.5	118.5	76.5	158	42	116	12.8	5.7	77.5	2	1	0	1	1	1	1	4.9	0.04	45.6
1	0	0	100	5.05	2	70	22.4	134	73.5	181	42	139	12.5	5	71.6	1	1	1	1	1	1	1	32.3	0.02	57.8
1	0	0	100	5.06	2	66	24.2	120	76	200	42	158	11.1	5.3	73.6	2	1	1	1	1	1	1	9.2	0.04	41.1
1	0	0	100	6.85	2	74	19.8	164.5	71.5	193	42	151	12.6	7.6	73.2	2	1	1	0	1	0	1	33.2	0.06	70.5
1	0	0	100	6.86	2	70	28.1	132.5	74.5	179	42	137	13.8	5.4	71.6	2	1	1	1	1	1	1	11.6	0.07	9.66
1	0	0	100	6.86	2	67	24.3	133.5	81.5	220	42	178	12.8	5.3	76.9	2	1	1	1	0	1	1	6.9	0.08	28.8
1	1	0	100	6.8	2	70	29.8	152	82	235	42	193	13.2	5.7	75.3	2	1	0	0	0	1	1	24.5	0.26	2.88
1	1	0	100	7.07	2	69	28	134.5	78	202	42	160	12.1	5.4	75.9	1	1	1	1	1	1	1	10.9	0.06	0.05
1	1	0	100	4.62	2	72	19.5	91.5	57	197	42	155	12.1	5.5	70.6	2	1	1	1	1	1	1	11.2	0.05	37.2
1	1	0	100	4.41	2	65	21.6	113.5	64.5	166	42	124	12.2	4.6	74.2	2	1	1	0	1	1	1	219.7	0.03	20.3
1	1	0	100	6.95	2	83	25.4	113.5	60.5	166	42	124	12.7	5.8	42.5	2	1	1	1	1	1	1	38.2	0.32	89.4
1	0	0	100	3.21	1	71	20.4	108.5	75.5	206	42	164	13.9	5.2	69.6	2	0	0	1	1	1	1	25.1	0.33	4.96
1	0	0	100	4.59	1	76	29.2	162.5	81.5	177	42	135	15.3	5.5	67.2	2	1	0	0	1	1	1	18.1	0.03	19.2
1	0	0	100	6.85	1	75	26.1	141	96	175	42	133	16.3	4.9	75.1	2	1	0	0	1	1	1	28.8	0.03	36.5
1	0	0	100	6.97	1	66	20.4	129.5	75.5	138	42	96	10	5.9	72.1	2	0	0	1	1	1	1	7.7	0.07	0.18
1	1	0	100	6.86	1	71	24.4	152	88	182	42	140	17.8	4.9	61.3	2	0	0	0	1	1	1	14.6	0.02	2.85
1	1	0	100	7.03	1	69	22.1	132.5	74.5	187	42	145	14.3	6.1	74.1	2	0	0	1	1	1	1	68.4	0.01	22.3
1	0	0	100	6.87	2	65	22.8	140.5	75	231	42	189	11	5.7	63.1	2	1	1	0	0	1	1	349.6	3.98	14.6
1	0	0	100	7.2	2	67	22.5	129	76.5	224	42	182	12.4	5.2	76.9	2	1	1	1	0	1	1	6	0.2	23.8
1	0	0	100	6.85	2	73	25.5	105.5	58	169	42	127	13.3	6.6	78.3	2	1	1	1	1	0	1	23	0.19	73.5
1	0	0	100	3.24	2	66	29.6	134	79	204	42	162	12.4	5.5	73.6	2	1	1	1	0	1	1	17.6	1.1	4.61
1	0	0	100	4.7	2	66	27.4	124.5	78	249	42	207	13.8	5.5	62.7	2	1	1	1	0	1	1	39.4	0.17	32
1	0	0	100	6.85	2	69	17.7	144	72	140	42	98	12.1	8.1	80.5	1	1	1	0	1	0	1	195.6	3.4	74.3
1	0	0	100	7.07	2	73	27.3	196.5	96	242	42	200	13	5.1	78.3	1	1	1	0	0	1	1	136.5	0.08	53.3
1	0	0	100	4.59	2	66	21.5	139	75.5	204	42	162	12.7	5	77.5	2	1	1	0	1	1	1	24.3	0.03	12.2
1	0	0	100	6.88	2	71	24.7	113	67.5	202	42	160	12.3	5.7	71.1	2	1	0	0	1	1	1	5.1	0.04	22.6
1	0	0	100	5.15	2	77	22.4	110.5	61	186	42	144	12.1	6.8	58.0	1	1	1	0	1	0	1	41.5	0.03	31.5
1	0	0	100	5.15	2	72	15.2	97.5	54	156	42	114	12.2	5.6	70.6	1	0	1	1	1	1	1	12.9	0.02	38.3
1	0	0	100	6.86	2	66	30.2	131	78.5	224	42	182	12.4	5.4	73.6	2	1	1	1	0	1	1	8.2	0.02	32
1	0	0	100	5.08	2	66	26.8	116.5	66	179	42	137	12.9	5.5	88.5	2	1	1	1	1	1	1	17.5	0.02	12.8
1	0	0	100	6.8	2	67	26.5	125.5	71.5	170	42	128	14.1	5.1	76.9	2	1	1	0	1	1	1	9.3	0.05	11.4
1	0	0	100	5.36	2	74	31.7	146	83.5	180	42	138	15.6	5.9	69.6	2	1	1	0	0	1	1	28.8	0.06	17.7
1	0	0	100	1.2	2	76	22.8	123.5	75.5	156	42	114	13.5	5.4	68.6	1	1	1	1	1	1	1	14.6	0.02	40.7
1	0	0	100	6.95	1	82	28.3	142.5	67.5	204	42	162	13.6	5.7	64.4	2	1	0	0	1	1	1	55.8	0.15	118
1	1	0	100	5.37	2	85	24.2	121	81	198	42	156	13.5	5.7	64.4	2	1	1	1	1	1	1	113.3	0.03	79.5
1	0	1	2.5	2.5	1	67	26.3	132	88.5	175	42	133	13	5.6	71.6	2	1	0	1	1	1	0	12.7	0.1	224
1	0	1	5.8	5.8	1	76	25.9	139	76.5	206	42	164	14.9	5.8	67.2	2	1	0	1	1	1	1	11.5	0.17	15.2
1	0	1	3	3	1	73	25.3	115.5	72	186	42	144	14.9	6.5	72.0	2	1	0	1	1	0	1	9.5	0.05	14.4
1	0	1	2	2	1	72	27.6	129	68.5	208	42	166	14.8	4.7	54.2	2	1	1	1	1	1	1	5.3	0.04	82.4
1	0	1	3.7	3.7	1	76	26.6	120.5	59.5	191	42	149	13.7	6.4	79.4	2	0	1	1	1	1	1	538.6	0.07	44.9
1	1	1	4.3	4.3	1	80	23.1	128	68	182	42	140	13.5	5.5	72.5	2	0	1	1	1	1	1	7.6	0.13	7.92
1	0	1	2.7	2.7	1	79	25.9	162	84.5	228	42	186	15.4	5.2	51.6	1	1	0	0	0	1	0	27.1	0.79	76.7
1	0	0	100	6.86	1	65	23.6	134.5	81.5	129	43	86	16.1	4.2	72.6	2	0	0	0	1	0	1	7.5	0.1	25.1
1	0	0	100	7.02	1	78	18	109.5	65.5	177	43	134	13.4	5	52.0	1	1	1	1	1	1	1	24.5	0.86	31.2
1	0	0	100	5.06	1	74	22.8	125	71	223	43	180	14.3	5.7	71.5	2	1	1	1	0	1	1	2.7	0.19	68
1	0	0	100	5.07	1	75	27.3	117.5	73.5	160	43	117	14.1	7	71.0	2	1	1	0	1	0	1	7.7	0.45	14.1

1	0	0	100	5.08	1	73	23.1	147	77.5	167	43	124	13.9	5.7	76.1	2	1	0	0	1	1	1	18.7	0.15	92.3
1	0	0	100	6.98	1	72	21.7	152	80	213	43	170	13.4	5.5	76.7	2	1	0	0	1	1	1	8.5	0.12	0.05
1	0	0	100	2.97	1	75	24.3	168.5	83	180	43	137	15.9	5	71.0	2	0	0	0	1	1	1	38.4	0.45	53.8
1	0	0	100	6.79	1	77	23.1	127	73	180	43	137	13.1	5.8	58.8	2	1	0	0	1	1	1	63.7	0.22	22.9
1	0	0	100	4.59	1	72	23.9	147.5	82.5	181	43	138	15.1	6.2	76.7	2	0	1	0	1	1	1	29.8	0.19	17
1	0	0	100	4.68	1	66	29.2	133	97.5	186	43	143	14.5	5.4	56.6	2	1	1	0	1	1	1	145.4	0.15	28.1
1	0	0	100	4.7	1	75	21.4	129	58	204	43	161	13.3	5.6	71.0	1	0	1	0	0	1	1	10.9	0.13	66.8
1	0	0	100	5.37	1	70	22.3	104	53.5	148	43	105	13.3	5.3	73.6	2	1	0	1	1	1	1	2.6	1.2	13.1
1	0	0	100	6.86	1	67	26.1	142	78	192	43	149	15.6	5.2	75.2	2	1	0	0	1	1	1	22.7	0.16	6.83
1	0	0	100	7.07	1	66	25.6	137.5	89	256	43	213	15.8	5	75.7	2	0	1	1	0	1	1	407.3	0.05	0.05
1	0	0	100	4.58	1	70	27.2	104	69	184	43	141	15.3	5.5	82.8	2	1	0	1	1	1	1	5.1	0.04	18.9
1	0	0	100	6.88	1	73	23.4	127	72.5	229	43	186	13.1	5.2	60.4	2	1	1	1	0	1	1	17	0.02	46.6
1	0	0	100	4.81	1	71	24.1	114	75.5	196	43	153	14.9	7.7	69.6	2	1	1	1	1	0	1	20	0.02	10.6
1	0	0	100	6.84	1	66	22	140	83	191	43	148	15.9	5.4	56.6	2	1	1	0	1	1	1	136.7	0.08	16.8
1	0	0	100	5.08	1	66	28.2	130.5	73.5	145	43	102	14.1	6.6	79.9	2	1	1	1	1	0	1	4.4	0.04	35.5
1	0	0	100	6.97	1	72	22.3	106	74.5	211	43	168	13.8	5	76.7	1	1	0	1	1	1	1	13.9	0.01	0.05
1	0	0	100	4.63	1	77	23.7	130.5	59	171	43	128	14.8	6.6	74.0	2	1	1	0	1	0	1	62.2	0.01	27.1
1	0	0	100	4.64	1	66	34.4	119.5	80	166	43	123	14.8	6.2	75.7	2	1	0	0	1	1	1	12.3	0.03	9.29
1	0	0	100	6.8	1	67	22.8	101	68	215	43	172	15	5.8	71.6	2	0	0	1	1	0	1	10	0.04	13.8
1	0	0	100	4.59	1	70	23.9	102.5	62	165	43	122	15	5.3	70.1	2	1	0	1	1	1	1	9.8	0.05	12.2
1	0	0	100	4.6	1	70	22.8	117	61	171	43	128	14.4	6.9	70.1	2	1	0	0	0	0	1	4.2	0.04	18.1
1	0	0	100	4.62	1	78	22.4	135.5	88.5	175	43	132	14.7	5.9	69.6	2	1	1	1	1	1	1	14	0.02	18.5
1	0	0	100	4.62	1	76	19.9	120	61	220	43	177	13.8	5.2	67.2	2	0	1	1	0	1	1	5.3	0.03	6.88
1	0	0	100	4.62	1	65	25.7	118.5	61	189	43	146	13.2	5.9	76.2	2	1	1	1	1	1	1	6.3	0.07	11.4
1	0	0	100	6.79	1	74	26	152.5	84	145	43	102	15.2	6	68.2	2	1	0	0	1	1	1	9.9	0.06	26.9
1	0	0	100	6.79	1	71	20.2	149.5	82.5	178	43	135	14.4	5.3	69.6	2	0	0	0	1	1	1	24.5	0.05	11
1	0	0	100	4.67	1	65	29.3	145.5	77	168	43	125	15.3	5.9	76.2	2	1	0	0	1	1	1	53.1	0.04	37.8
1	0	0	100	4.69	1	70	22.1	115.5	70.5	148	43	105	15	4.3	73.6	1	1	0	1	1	1	1	6	0.01	14.7
1	0	0	100	5.36	1	65	22.5	120.5	77	143	43	100	14.3	5.4	80.5	2	1	1	0	1	1	1	36.7	0.04	15.4
1	0	0	100	5.37	1	73	29.3	153	76.5	199	43	156	15.1	5.9	68.6	2	1	1	0	1	0	1	24.5	0.01	18.1
1	0	0	100	5.06	1	73	20.1	152.5	101	164	43	121	14.5	5.4	72.0	2	1	1	0	1	1	1	9.7	0.02	14.7
1	0	0	100	6.87	1	78	24.2	107	64	176	43	133	14.4	7.5	66.3	2	1	0	1	1	0	1	32.5	0.09	52.1
1	0	0	100	6.87	1	75	20.9	132.5	72	96	43	53	12.5	5.6	59.6	2	0	0	1	1	1	1	15.6	0.01	26
1	0	0	100	0.36	1	68	22.1	129.5	85	138	43	95	13.6	5.5	71.1	2	1	0	0	1	1	1	14.7	0.04	44
1	0	0	100	5.04	1	65	27.4	147.5	92	153	43	110	14.7	6	72.6	2	1	1	0	1	1	1	11.4	0.06	10.3
1	0	0	100	5.04	1	66	24.8	116	65.5	177	43	134	15.5	5.9	56.6	2	1	1	1	1	1	1	5.1	0.03	7.17
1	0	0	100	7.23	2	66	26.1	104	59	206	43	163	14.2	5.7	77.5	2	1	1	1	1	1	1	12.1	0.24	20
1	0	0	100	6.88	2	65	30.6	126	71.5	200	43	157	13.1	5.6	63.1	2	1	1	1	1	1	1	23.7	0.74	15.8
1	0	0	100	6.86	2	74	21.4	106	57.5	229	43	186	13.2	5.6	69.6	2	1	1	1	0	1	1	3.5	0.12	18.4
1	0	0	100	7.01	2	68	27.8	150	89.5	209	43	166	12.5	5.2	53.6	1	1	1	0	1	1	1	75.7	0.23	18.7
1	0	0	100	7.01	2	73	27.1	193.5	103	224	43	181	12.6	5.4	73.8	1	1	1	0	0	1	1	8.6	0.16	38.1
1	0	0	100	7.03	2	67	21.1	112	66	225	43	182	12.9	5.1	76.9	2	1	1	1	0	1	1	10.4	0.15	6.37
1	0	0	100	6.83	2	66	24.4	161	81.5	248	43	205	12	5.3	82.3	2	1	1	0	0	1	1	15.5	0.13	40.6
1	0	0	100	6.84	2	70	30.8	134.5	84	214	43	171	15	5.8	52.8	2	1	1	1	0	1	1	6.8	0.1	12.9
1	0	0	100	4.59	2	73	23.2	95	53.5	163	43	120	12.4	6.2	70.1	2	1	1	1	1	0	1	6.1	0.13	14.9
1	0	0	100	4.58	2	68	29.3	115.5	60	183	43	140	13.7	5.7	76.4	2	1	1	1	1	1	1	21.5	0.05	14.9
1	0	0	100	6.86	2	70	21.9	136.5	76	173	43	130	11.7	5.2	75.3	2	1	1	1	1	1	1	17	0.02	16.2
1	0	0	100	5.16	2	78	28.6	136.5	70.5	252	43	209	12.7	5.8	71.2	1	1	1	0	0	1	1	33	0.05	10.3
1	0	0	100	6.83	2	74	24.2	129	78	173	43	130	10.8	5.6	59.2	2	1	1	1	1	1	1	27.4	0.01	17.4
1	0	0	100	5.05	2	76	23.9	156	80	180	43	137	13	6.1	72.2	1	1	1	0	1	1	1	17.2	0.03	26.8
1	0	0	100	4.63	2	77	29.3	137.5	86.5	209	43	166	14	5.5	68.2	2	1	1	1	1	1	1	3.6	0.07	27.7

1	0	0	100	4.64	2	70	26.2	138.5	78	174	43	131	13.4	5.3	75.3	2	1	1	1	1	1	1	5	0.02	23.4
1	0	0	100	6.87	2	66	24	118.5	69.5	229	43	186	13.4	5.4	82.3	2	1	1	1	0	1	1	31.1	0.06	32.4
1	0	0	100	6.79	2	71	21	151	84.5	171	43	128	13.9	5.4	74.8	2	1	1	0	1	1	1	23.8	0.01	21.9
1	0	0	100	6.84	2	67	24.9	127	75.5	215	43	172	12.1	5.3	76.9	2	1	1	1	1	1	1	25.4	0.03	7.48
1	0	0	100	1.32	2	67	31.3	91.5	58.5	176	43	133	13	5.1	73.1	2	1	1	1	1	1	1	9.2	0.06	11.8
1	0	0	100	4.62	2	70	30.6	131	78.5	205	43	162	14.4	5.3	75.3	2	1	1	0	1	1	1	23.6	0.02	20.7
1	0	0	100	6.44	2	65	25.3	128	68.5	186	43	143	12	5	48.2	2	1	1	0	1	1	1	9.7	0.06	7.51
1	0	0	100	5.36	2	66	23	132.5	75	205	43	162	12.7	5.6	77.5	2	1	1	0	1	1	1	40.1	0.05	26.2
1	0	0	100	5.04	2	65	28.8	127.5	71.5	227	43	184	12.6	5.9	78.0	2	1	1	0	0	1	1	11	0.04	17.8
1	1	0	100	5.37	2	70	29.5	124.5	62	172	43	129	13.8	5.6	80.0	2	1	1	0	1	1	1	207.4	0.1	36.8
1	1	0	100	6.52	1	85	26.9	123	56.5	166	43	123	13.5	6.1	70.0	1	1	1	1	1	1	1	3	0.03	40.5
1	1	0	100	5.08	2	81	30.4	171	88.5	171	43	128	14.3	5.7	74.0	1	1	1	0	1	1	1	392.3	0.04	20.4
1	0	0	100	2.32	1	69	25.5	113	67	208	43	165	14.4	5.2	78.3	2	1	1	1	1	1	1	8.1	0.18	9.18
1	0	0	100	6.85	1	75	23	109.5	82	231	43	188	15.4	9.8	75.1	1	1	0	1	0	0	1	128.5	0.28	2.82
1	0	0	100	7.02	1	68	27.8	114.5	74	229	43	186	14.2	5.7	84.0	2	1	1	1	0	1	1	5.8	0.3	6.14
1	0	0	100	7	1	72	23.5	120	80.5	196	43	153	10.8	5.4	48.8	2	1	1	0	1	1	1	390.2	0.13	11.7
1	0	0	100	4.63	1	71	22.5	105	63	240	43	197	14.9	5.8	73.1	2	0	0	1	0	1	1	7.3	0.12	31.5
1	0	0	100	4.59	1	77	18.3	104	69	155	43	112	13.9	5.2	42.8	2	1	1	1	1	1	1	22.9	0.02	14
1	0	0	100	6.95	1	66	23	171	103.5	189	43	146	16.1	4.8	85.2	2	1	0	0	1	1	1	25.2	0.02	10.4
1	0	0	100	6.98	1	70	25.6	141	79.5	180	43	137	15.7	5.5	77.7	2	0	0	0	1	1	1	37.2	0.05	36.6
1	0	0	100	6.8	1	72	22.7	134.5	77.5	207	43	164	13.4	5	69.1	2	1	1	1	1	1	1	7.4	0.05	21.1
1	0	0	100	5.37	1	68	23.5	133.5	80.5	151	43	108	14.9	5.3	78.8	2	1	0	1	1	1	1	13.9	0.07	24.5
1	0	0	100	5.04	1	74	26.9	127.5	81	180	43	137	15.3	5.7	68.2	2	1	1	1	1	1	1	4.9	0.09	12.7
1	0	0	100	6.85	2	78	23.7	123.5	65.5	226	43	183	13.5	5.7	71.2	2	1	1	1	0	1	1	5.5	0.16	41.3
1	0	0	100	7.03	2	70	29.5	119	71	222	43	179	12.4	5.6	60.9	2	1	1	0	0	1	1	33.4	0.26	16.5
1	0	0	100	4.63	2	77	23	152.5	78	205	43	162	13	5.7	58.0	1	1	1	0	1	1	1	5.4	0.14	160
1	0	0	100	4.64	2	71	25	139	71.5	168	43	125	12.4	6.6	71.1	1	1	1	0	0	0	1	10	0.34	23.2
1	0	0	100	6.8	2	70	24.4	125	74.5	182	43	139	15	5.4	75.3	2	1	1	1	1	1	1	9.6	0.11	21.1
1	0	0	100	6.44	2	76	25.2	174	80	183	43	140	12.1	6.1	58.4	2	1	1	0	1	0	1	26.5	0.51	55
1	0	0	100	6.87	2	79	27.2	132.5	79.5	162	43	119	12.4	5.3	57.2	1	1	1	0	1	1	1	41.2	0.29	6.76
1	0	0	100	6.87	2	73	26.4	143	67.5	199	43	156	14.3	5.6	73.8	2	1	1	0	1	1	1	12.3	0.09	26
1	0	0	100	7.04	2	78	28.3	137.5	79.5	229	43	186	13.4	5.3	71.2	2	1	1	0	0	1	1	7.7	0.03	2.32
1	0	0	100	5.06	2	71	22.5	125.5	71	196	43	153	15.1	5.3	79.4	1	0	1	0	1	1	1	46.1	0.02	13.3
1	0	0	100	5.08	2	74	24.4	139.5	86.5	229	43	186	13.5	5.5	59.2	1	1	0	0	0	1	1	34.1	0.04	62.7
1	0	0	100	6.99	2	67	24.5	129.5	85.5	189	43	146	14.2	4.8	73.1	2	1	1	0	1	1	1	89.9	0.03	11.4
1	0	0	100	4.62	2	71	23.7	147	77	171	43	128	13	5.5	74.8	2	1	1	0	1	1	1	36.2	0.02	29
1	0	0	100	5.02	2	65	28.3	120	69	206	43	163	12.4	5.1	74.2	2	1	1	1	1	1	1	10	0.09	41.4
1	0	0	100	4.6	2	68	29.1	118	67	177	43	134	12	5.6	76.4	2	1	1	0	1	1	1	24.1	0.09	9.97
1	0	0	100	5.37	2	74	19.1	98	53.5	183	43	140	11	6	77.8	2	1	1	1	1	1	1	9.8	0.06	13.2
1	0	0	100	5.05	2	66	28.6	100	45	147	43	104	13.1	5.8	73.6	2	1	1	1	1	1	1	9.8	0.02	15.1
1	1	0	100	6.98	2	71	24.4	122	74	158	43	115	13	5.5	74.8	1	1	1	0	1	1	1	9.3	0.04	15.5
1	1	0	100	6.97	2	75	22.9	140	88	210	43	167	12	5.7	72.7	2	1	1	0	1	1	1	46.1	0.01	8.63
1	1	0	100	6.98	2	69	20.9	115.5	74	146	43	103	10.8	5.1	72.1	2	1	1	1	1	1	1	23.5	0.01	19.4
1	1	0	100	6.87	1	85	22.6	141	71	221	43	178	12.3	5.4	63.1	2	1	1	0	0	1	1	10.9	0.09	70.4
1	1	1	6	6	1	74	26.3	137.5	80	249	43	206	16.4	5.9	48.1	2	1	0	0	0	1	1	11.1	0.09	7.32
1	0	1	6.6	6.6	2	68	28.5	136.5	95.5	219	43	176	12.6	5.6	76.4	2	1	1	0	1	1	1	17.5	0.03	20.9
1	1	1	3.3	3.3	2	79	24.7	135.5	82.5	204	43	161	13.7	5.4	67.2	2	1	1	1	1	1	1	14.7	0.04	72.5
1	0	0	100	4.58	1	68	24.9	133.5	78	185	44	141	15.9	5.4	74.6	2	1	1	1	1	1	1	26.9	0.11	28
1	0	0	100	6.75	1	68	29.1	111	72.5	234	44	190	17.9	6.2	55.8	2	0	0	1	0	0	0	170.5	0.28	29
1	0	0	100	6.84	1	73	28.5	138	82.5	134	44	90	18.8	5.6	72.0	2	1	0	0	1	1	1	324.9	0.3	17.8
1	0	0	100	6.85	1	67	21.6	113.5	70	148	44	104	14.1	4.8	75.2	2	0	0	1	1	1	1	14	0.11	41.3

1	0	0	100	6.85	1	66	19.8	130.5	62.5	153	44	109	15.8	5.1	75.7	2	0	1	1	1	1	1	22.6	0.16	16.8
1	0	0	100	7.01	1	73	24.4	125	80	202	44	158	14.5	5.4	68.6	2	0	0	1	1	1	1	13.5	0.15	17.9
1	0	0	100	7.04	1	71	25.3	142.5	79	229	44	185	13.8	5.5	37.5	2	0	0	0	0	1	1	1091.8	0.18	0.05
1	0	0	100	5.05	1	75	25	118.5	69.5	157	44	113	15.8	6	71.0	2	1	1	1	1	1	1	20.7	0.19	26.8
1	0	0	100	4.63	1	75	27.3	142.5	88.5	156	44	112	14.7	5.4	71.0	2	1	1	0	1	1	1	13.8	0.1	23.8
1	0	0	100	4.64	1	72	23.7	147	93.5	221	44	177	14.8	5.7	76.7	2	1	0	0	0	1	1	29.6	0.25	19.9
1	0	0	100	4.64	1	71	26	129	77	175	44	131	15.6	5.8	73.1	2	0	0	1	1	1	1	89.7	0.39	16.1
1	0	0	100	6.8	1	67	23.6	135	78.5	129	44	85	12.2	5.9	75.2	2	1	1	1	1	1	1	10.3	0.11	26.9
1	0	0	100	4.69	1	67	23	143.5	92.5	182	44	138	15.5	5.4	79.4	2	1	0	0	1	1	1	11.1	0.17	14.1
1	0	0	100	5.04	1	67	27.9	162	99.5	204	44	160	14.6	6.3	75.2	2	1	1	0	1	1	1	62.8	0.11	9.71
1	0	0	100	7.08	1	76	25.9	116.5	69	165	44	121	15.1	5.2	67.2	2	1	1	0	0	1	1	13.6	0.04	12.7
1	0	0	100	4.58	1	71	23.1	130	72.5	165	44	121	14.9	6.3	77.2	2	1	0	0	1	1	1	11.9	0.03	0.77
1	0	0	100	4.58	1	65	23.1	177.5	101	156	44	112	15.1	5.9	76.2	2	1	1	0	1	1	1	18	0.02	54.7
1	0	0	100	4.59	1	67	25.4	116.5	78.5	182	44	138	16.9	5	71.6	2	1	0	0	1	1	1	13.7	0.04	14.6
1	0	0	100	6.86	1	67	24.6	91.5	64.5	180	44	136	14.5	5.3	75.2	2	0	1	1	1	1	1	3.7	0.06	20.4
1	0	0	100	5.16	1	73	28.7	149.5	85	192	44	148	14.9	5.6	72.0	2	1	1	0	1	1	1	10.2	0.02	54
1	0	0	100	5.16	1	73	28.6	143.5	89.5	196	44	152	13.4	5.2	53.9	1	1	1	0	1	1	1	6.2	0.07	26.4
1	0	0	100	6.85	1	75	17.7	123.5	70	224	44	180	14.6	5.5	67.7	2	0	1	1	0	1	1	4.5	0.04	15.3
1	0	0	100	7.01	1	78	24.5	121.5	69.5	163	44	119	15.7	5.9	73.5	2	1	0	1	1	1	1	17.8	0.02	30.8
1	0	0	100	7.01	1	72	23.1	142.5	85.5	161	44	117	13.4	5.3	48.8	2	1	1	0	1	1	1	61.7	0.01	18.8
1	0	0	100	7.03	1	69	22.5	119	73	160	44	116	15.5	5.4	74.1	2	1	1	1	1	1	1	5.9	0.01	8.26
1	0	0	100	7.04	1	71	26	126	72.5	255	44	211	15.2	5.4	73.1	2	1	1	0	0	1	1	7.4	0.05	2.99
1	0	0	100	6.38	1	74	19.6	141.5	69.5	153	44	109	5.9	4.9	80.5	2	1	1	0	1	1	1	6.5	0.01	47.3
1	0	0	100	4.63	1	69	26.3	144	73	218	44	174	15.4	7.3	74.1	2	1	1	0	1	0	1	10	0.06	16.1
1	0	0	100	4.63	1	68	27.1	125	81.5	181	44	137	15.1	5.7	74.6	2	1	0	1	1	1	1	5.4	0.05	9.59
1	0	0	100	2.36	1	74	27.3	150.5	88.5	215	44	171	16.8	6.4	80.5	2	1	0	0	1	0	1	271.6	0.05	27.7
1	0	0	100	6.95	1	68	23.2	143.5	79.5	176	44	132	16.4	4.8	71.1	2	1	0	0	1	1	1	35	0.07	10.2
1	0	0	100	6.97	1	67	22.7	188.5	95.5	200	44	156	16.6	4.7	84.6	2	1	0	0	1	1	1	148.7	0.02	16.2
1	0	0	100	1.55	1	71	22.2	128.5	80.5	200	44	156	15.5	5.5	73.1	2	0	0	1	1	1	1	13	0.05	27.5
1	0	0	100	1.62	1	66	25.7	158.5	87.5	218	44	174	16.3	5.2	75.7	2	1	0	0	1	1	1	8.6	0.08	30.8
1	0	0	100	6.83	1	77	21.9	134	82	137	44	93	13.9	5.3	74.0	2	1	0	1	1	1	1	4.1	0.04	37.5
1	0	0	100	4.6	1	74	25	110.5	66	117	44	73	14.1	5.6	53.5	2	1	0	0	1	1	1	37.8	0.05	165
1	0	0	100	2.98	1	65	23.7	172.5	89	199	44	155	15.8	5	80.5	2	0	0	0	1	1	1	20.1	0.03	5
1	0	0	100	5.37	1	66	25.9	129.5	76.5	157	44	113	14.9	5.1	72.1	2	1	0	1	1	1	1	10.6	0.02	33.3
1	0	0	100	6.45	1	74	26.6	131.5	77	193	44	149	15.4	5.9	68.2	2	1	0	0	1	1	1	15.3	0.05	40.5
1	0	0	100	6.87	1	70	23.5	150	73	152	44	108	14.4	5.3	77.7	2	1	1	0	1	1	1	18.4	0.02	30.1
1	1	0	100	7.08	1	74	25.9	144.5	94.5	124	44	80	16	7.8	75.6	2	0	0	0	1	0	1	44.8	0.3	33.2
1	1	0	100	6.97	1	66	25	136.5	84.5	178	44	134	14.6	5.2	75.7	1	0	0	1	1	1	1	15.2	0.36	2.68
1	1	0	100	6.45	1	74	23.2	121	81.5	148	44	104	15.3	5.1	71.5	2	1	0	0	1	1	1	39.4	0.02	23.6
1	0	0	100	7.05	2	65	26	135.5	85.5	173	44	129	13	5.3	63.1	2	1	1	1	1	1	1	8.3	0.16	8.96
1	0	0	100	7.08	2	71	25.3	168.5	75.5	246	44	202	15.3	5.8	71.1	1	1	1	0	0	1	1	340.3	0.12	0.05
1	0	0	100	6.88	2	76	20.9	102.5	58	196	44	152	12.2	5.3	58.4	2	1	1	1	1	1	1	7.1	0.15	31.4
1	0	0	100	5.08	2	65	24.4	126	74	212	44	168	13.1	5.7	74.2	2	1	1	1	1	1	1	19.2	0.35	13.8
1	0	0	100	4.63	2	66	26.3	127	71.5	246	44	202	14.2	5.9	73.6	2	1	1	1	0	1	1	16.5	0.14	38.1
1	0	0	100	0.46	2	78	19.6	155.5	81	174	44	130	12	5.8	71.2	2	1	1	0	1	1	1	26.7	0.37	48.1
1	0	0	100	6.8	2	65	28	146.5	82	187	44	143	14.1	6.1	63.1	2	1	1	0	1	1	1	974.2	0.44	21.9
1	0	0	100	6.8	2	71	21.1	147.5	83.5	218	44	174	14.1	5.8	74.8	2	1	1	0	1	1	1	6.3	0.58	7.34
1	0	0	100	6.85	2	72	25.6	124.5	69	202	44	158	13.2	5.5	74.3	2	1	1	1	1	1	1	64.1	0.15	32.4
1	0	0	100	6.85	2	76	22.8	160.5	89	194	44	150	11.7	5.4	68.6	1	1	1	0	1	1	1	48.6	0.78	55.4
1	0	0	100	7	2	67	27.5	105	63.5	236	44	192	14.2	5.6	73.1	1	1	1	1	0	1	1	12.4	0.04	30.7
1	0	0	100	7.07	2	76	27.7	121.5	69.5	207	44	163	14.1	5.2	68.6	1	1	1	0	0	1	1	19.7	0.03	8.52

1	0	0	100	4.58	2	67	21.1	113.5	70	225	44	181	13.5	5.5	62.2	2	1	1	1	0	1	1	6.6	0.05	67.2
1	0	0	100	6.87	2	65	28	141	78.5	205	44	161	12.2	5.2	63.1	2	1	1	0	1	1	1	19.9	0.02	40
1	0	0	100	6.84	2	66	27.4	144	77	182	44	138	11	6.9	82.3	2	1	1	0	1	0	1	21.6	0.08	14.2
1	0	0	100	6.84	2	75	24.6	101.5	65	196	44	152	12.7	6.2	72.7	2	1	1	1	1	1	1	5.2	0.08	7.99
1	0	0	100	6.85	2	69	27.1	111.5	72	185	44	141	12.9	5.2	80.5	2	1	1	1	1	1	1	14.9	0.02	21.4
1	0	0	100	5.07	2	71	24.6	127	85.5	186	44	142	14.4	5.7	74.8	2	1	1	1	0	1	1	6.4	0.02	25.3
1	0	0	100	6.97	2	73	27	125	64.5	252	44	208	12.8	5.4	59.7	2	1	1	0	0	1	1	11.1	0.08	28.8
1	0	0	100	6.98	2	74	22.6	129	68	193	44	149	13.7	5.2	69.6	2	1	1	1	1	1	1	10.4	0.06	0.05
1	0	0	100	6.99	2	65	24.6	135.5	81	167	44	123	12.5	5.2	74.2	2	1	1	1	1	1	1	7	0.03	27.1
1	0	0	100	4.62	2	69	22.6	128.5	73.5	209	44	165	13.8	7.5	72.1	2	1	1	0	1	0	1	13.6	0.02	48.9
1	0	0	100	4.62	2	69	26	164	87	201	44	157	14.4	4.8	75.9	2	1	1	0	1	1	1	24.8	0.04	22.3
1	0	0	100	4.63	2	68	33.3	118.5	81	206	44	162	13.7	5.9	72.6	2	1	1	0	1	1	1	20.2	0.05	9.68
1	0	0	100	7.15	2	68	32.6	185	114.5	198	44	154	14.6	5.3	76.4	2	1	1	0	1	1	1	19.9	0.06	30.6
1	0	0	100	6.8	2	67	23.9	95.5	55	190	44	146	13	5.3	76.9	2	1	1	1	1	1	1	21.5	0.03	16.2
1	0	0	100	4.6	2	67	27.1	121	73	171	44	127	14.1	5.5	76.9	2	1	0	1	1	1	1	6.1	0.02	6.12
1	0	0	100	4.62	2	69	24.4	118.5	68.5	205	44	161	12.1	5.8	75.9	2	1	1	1	1	1	1	10.8	0.02	33.2
1	0	0	100	4.62	2	65	25.1	116	77	227	44	183	12.5	4.9	74.2	2	1	0	0	0	1	1	32.7	0.03	18.6
1	0	0	100	6.96	2	70	25.3	138.5	66	196	44	152	12	5.8	80.0	2	1	1	1	1	1	1	7.2	0.07	11.2
1	0	0	100	6.96	2	75	18.7	134.5	73	236	44	192	10.9	5	72.7	2	1	1	1	0	1	1	8.6	0.01	0.05
1	0	0	100	5.37	2	65	21.8	112	72.5	132	44	88	11.3	5.4	74.2	2	1	1	1	1	1	1	11.1	0.08	55.1
1	0	0	100	5.37	2	65	29	99.5	53.5	204	44	160	12.9	5.9	78.0	2	1	1	1	1	1	1	16.4	0.02	19.4
1	0	0	100	5.06	2	74	23.5	140.5	76	204	44	160	13.1	5.8	69.6	2	1	1	0	1	1	1	3	0.04	15.5
1	0	0	100	5.06	2	70	23.2	150.5	90	242	44	198	13.8	6.2	71.6	1	1	1	0	0	1	1	81	0.05	37.6
1	0	0	100	6.86	2	69	28	149	74.5	225	44	181	13.9	4.9	80.5	1	1	1	0	0	1	1	57.8	0.07	30.5
1	0	0	100	5.04	2	67	28	144.5	60	202	44	158	11.6	5.6	76.9	2	1	1	0	1	1	1	12	0.07	58.2
1	1	0	100	6.87	2	76	29.5	125.5	73.5	230	44	186	14.9	7	72.2	1	1	1	0	0	0	1	24	0.28	17.1
1	1	0	100	4.64	2	71	23.4	117.5	55.5	211	44	167	13.2	5.4	71.1	2	1	1	1	0	1	1	23.3	0.05	64.5
1	1	0	100	6.98	2	78	24.2	130.5	69	238	44	194	13.7	5.5	67.7	1	1	1	0	0	1	1	49.9	0.09	33.6
1	1	0	100	6.8	2	76	28.4	150.5	90	204	44	160	14.2	5.3	72.2	1	1	1	0	1	1	1	919.8	0.08	46.9
1	1	0	100	5.19	2	79	27.3	121.5	65	215	44	171	13.8	5.2	67.2	1	1	1	0	0	1	1	20.9	0.07	71.1
1	0	0	100	5.16	1	83	21.5	133	77	213	44	169	17	5.6	67.2	1	1	1	1	1	1	1	15.1	0.02	64.6
1	0	0	100	6.98	2	81	24.3	127	67	162	44	118	12.1	5.3	74.0	1	1	1	1	1	1	1	17	0.01	9.35
1	0	0	100	6.84	1	66	24.9	114	72.5	170	44	126	14.2	5.2	63.5	2	1	0	0	0	1	1	210.1	6.45	16.2
1	0	0	100	6.8	1	74	27.6	159	97.5	211	44	167	16	5.6	68.2	2	1	0	0	1	1	1	31.5	0.1	9.53
1	0	0	100	4.81	1	69	25.4	122	69	204	44	160	15.7	5.6	70.6	2	1	1	1	1	1	1	4.5	0.01	11.5
1	0	0	100	7.21	1	74	17.6	115	71	180	44	136	14.6	5.4	68.2	2	0	1	0	1	1	1	26.1	0.05	34.8
1	0	0	100	7.21	1	71	29.9	142.5	81.5	244	44	200	14.8	6.5	77.2	2	0	0	0	0	0	1	69.3	0.02	77.7
1	0	0	100	4.64	1	66	21.1	164	113	158	44	114	13.7	5	75.7	2	0	1	0	1	1	1	17.9	0.02	16
1	0	0	100	4.18	1	77	24.3	143	89	191	44	147	14.7	5.8	66.7	2	1	1	0	1	1	1	195	0.07	11.9
1	1	0	100	7	1	79	24	102.5	60	151	44	107	13.5	5.5	73.0	2	1	1	1	1	1	1	20	0.31	27.8
1	0	0	100	7.02	2	72	20	142	74	183	44	139	12.9	5.6	70.6	2	1	1	0	1	1	1	7.3	0.14	87.8
1	0	0	100	5.07	2	65	22.5	106.5	60	177	44	133	13.2	5.6	82.8	2	1	1	1	1	1	1	7.4	0.21	26.6
1	0	0	100	7	2	71	24.8	122.5	68.5	229	44	185	11.4	5	71.1	1	1	1	1	0	1	1	46	0.31	15.5
1	0	0	100	4.7	2	67	23.9	136.5	73.5	133	44	89	13	5.6	87.9	2	1	0	1	1	1	1	88.7	0.1	5.54
1	0	0	100	6.86	2	70	26.3	144.5	78	229	44	185	13.2	5.4	71.6	2	1	1	0	0	1	1	61.7	0.1	16.3
1	0	0	100	7	2	73	23.2	122.5	70	273	44	229	13.8	5.2	73.8	1	1	1	1	0	1	1	37.7	0.04	14.7
1	0	0	100	7	2	73	26.6	140	72	278	44	234	14.2	9.3	45.5	2	1	1	0	0	0	1	12	0.05	31.3
1	0	0	100	4.59	2	66	25.2	104.5	76.5	228	44	184	13	5.3	77.5	2	1	1	0	0	1	1	19.4	0.07	7.29
1	0	0	100	4.62	2	72	20.8	119	62	174	44	130	12.8	5.9	74.3	2	1	1	1	1	1	1	11.2	0.04	28.4
1	0	0	100	4.63	2	76	24.5	139	83	216	44	172	13.7	5	68.6	2	1	1	0	1	1	1	23.1	0.07	36
1	0	0	100	4.64	2	73	27.3	160.5	82	227	44	183	13.9	5.5	59.7	2	1	1	0	0	1	1	17.5	0.07	11

1	0	0	100	6.82	2	72	27.9	130.5	83	249	44	205	12.8	5.8	78.9	1	1	1	1	0	1	1	15.6	0.04	25
1	0	0	100	6.83	2	66	28.4	111	71.5	236	44	192	13	5.5	77.5	2	1	1	1	0	1	1	9.3	0.04	0.05
1	0	0	100	6.84	2	70	21.9	130.5	76	221	44	177	11.7	5.5	52.8	2	1	1	1	0	1	1	6.8	0.04	5.46
1	0	0	100	4.62	2	75	27.8	150.5	69.5	154	44	110	11.1	5.6	72.7	1	1	1	0	1	1	1	73	0.03	85.4
1	0	0	100	4.7	2	73	27	151.5	82	118	44	74	12.3	6.4	78.3	2	1	1	0	1	0	1	30.9	0.07	35
1	0	0	100	5.06	2	79	27	123.5	65	267	44	223	13.1	5.4	43.7	2	1	1	1	0	1	1	4.8	0.04	46.7
1	0	0	100	5.06	2	72	24.6	102.5	66	221	44	177	13.2	8.5	60.1	2	1	1	0	0	0	1	16.9	0.05	29.3
1	0	0	100	6.45	2	69	20.5	162	81.5	212	44	168	14.1	5.8	80.5	2	1	1	0	0	1	1	25.4	0.02	26.5
1	0	0	100	6.87	2	67	25.7	128	85.5	176	44	132	15.6	5.4	76.9	2	1	1	0	1	1	1	129	0.08	13.3
1	1	0	100	6.84	2	69	23.1	128	75	212	44	168	12.3	5.9	72.1	1	1	1	1	1	1	1	15.5	0.4	35.3
1	1	0	100	7.03	2	72	22.8	124.5	80.5	226	44	182	12	5.3	74.3	2	1	1	1	0	1	1	7.7	0.03	19.6
1	0	0	100	7.04	1	80	24.1	135.5	83.5	189	44	145	15.4	5.4	68.6	2	1	1	1	1	1	1	10.9	0.23	0.05
1	0	0	100	5.06	1	82	24.7	112	65	140	44	96	11.5	5.7	50.6	2	1	0	1	1	1	1	122.6	0.3	97.8
1	0	0	100	7	2	85	24.2	102.5	56	164	44	120	11.8	5.5	64.4	1	1	1	1	1	1	1	11.8	1.09	54.9
1	0	0	100	5.04	2	80	27.6	121	71	196	44	152	12.6	5.3	70.2	1	1	1	0	1	1	1	12.4	0.68	45.3
1	0	1	0.9	0.9	1	71	23.8	129	75.5	231	44	187	15.4	7.5	73.1	1	0	0	1	0	0	1	244.9	0.12	4.26
1	0	1	2.6	2.6	1	74	27.6	144	90.5	155	44	111	15.5	6.7	71.5	2	1	0	0	1	0	1	20.7	0.03	11.4
1	1	1	1.7	1.7	1	76	22.6	153	82	189	44	145	14	5.5	74.5	1	1	0	0	1	1	1	11.2	0.03	4.89
1	1	1	5.2	5.2	2	71	32.9	120	69.5	181	44	137	13.7	6	74.8	2	1	1	0	1	1	1	48.4	0.3	11.4
1	1	1	2.4	2.4	1	74	27.9	154.5	83.5	180	44	136	11.9	5.5	75.6	1	1	1	0	1	1	1	21.8	0.16	12.8
1	1	1	2.6	2.6	1	72	31.5	135	87	192	44	148	13.9	5.5	72.6	2	0	1	0	1	1	1	12.8	0.38	1.23
1	1	1	3.5	3.5	2	78	29.4	145	76	202	44	158	13.5	7	71.2	1	1	1	0	1	0	1	61	0.33	210
1	0	0	100	6.88	1	71	22.5	155.5	81.5	146	45	101	14.6	5.4	73.1	2	0	0	0	1	1	1	6	0.27	16
1	0	0	100	7.2	1	73	21.9	142	81	167	45	122	13.7	5.4	60.4	2	1	1	0	1	1	0	39.4	0.7	125
1	0	0	100	7.21	1	79	23.5	128.5	67.5	210	45	165	15.3	5.1	51.6	2	1	1	1	1	1	1	6	0.1	23.8
1	0	0	100	7.03	1	73	25.6	157	95	237	45	192	14.3	5.5	68.6	2	1	1	0	0	1	1	204.9	0.12	15.7
1	0	0	100	7	1	77	28	121.5	68	165	45	120	14.5	5.8	66.7	2	0	0	1	1	1	1	6	3.44	13.9
1	0	0	100	6.42	1	73	26.3	121.5	74	211	45	166	14.7	9.7	81.1	2	1	1	1	1	0	1	211	0.11	7.83
1	0	0	100	4.64	1	66	29.3	135	80.5	193	45	148	15.5	5.9	79.9	2	0	0	1	1	1	1	5.3	0.12	18.8
1	0	0	100	4.54	1	71	23.9	121.5	77	151	45	106	13.4	5.5	73.1	2	1	0	1	1	1	1	10.8	1.55	14.8
1	0	0	100	6.44	1	68	25.3	145.5	80.5	174	45	129	16.2	5	78.8	2	1	0	0	1	1	1	8.5	0.14	20.7
1	0	0	100	4.58	1	68	25.5	170	86.5	192	45	147	15	5.7	78.8	2	1	1	0	1	1	1	21.2	0.06	70.1
1	0	0	100	6.86	1	67	20.7	115	81.5	160	45	115	11.4	4.9	38.6	2	0	0	0	1	1	1	12	0.03	20.6
1	0	0	100	6.86	1	71	20.3	94.5	60	182	45	137	13.9	4.9	49.2	2	1	0	1	1	1	1	15.4	0.03	0.05
1	0	0	100	6.86	1	79	20.9	110	63.5	142	45	97	13.4	5	65.8	2	1	1	1	1	1	1	9.4	0.07	41.1
1	0	0	100	4.81	1	67	25.6	133	83	187	45	142	16.1	5.3	79.4	2	1	0	0	1	1	1	9.6	0.07	11.7
1	0	0	100	6.85	1	68	22.2	130	95	129	45	84	16.1	5.6	78.8	2	1	0	0	1	1	1	14.2	0.08	0.05
1	0	0	100	6.86	1	68	20.3	105	76	178	45	133	16.1	5	71.1	2	1	0	1	1	1	1	6	0.02	1.32
1	0	0	100	5.31	1	68	16.8	129.5	74.5	138	45	93	12.9	5.7	99.2	1	0	0	1	1	1	1	7.3	0.06	19
1	0	0	100	7.03	1	78	23.6	134.5	84.5	178	45	133	15.7	5.1	58.3	2	1	0	1	0	1	0	12.2	0.07	5.86
1	0	0	100	7.04	1	73	23.9	105	69.5	191	45	146	12.8	5.3	76.1	2	1	0	1	1	1	1	2.8	0.07	43.4
1	0	0	100	5.06	1	72	27	141.5	79.5	179	45	134	15.3	6.2	76.7	2	0	0	0	1	0	1	11.9	0.06	40
1	0	0	100	5.06	1	72	23.4	142.5	85.5	211	45	166	15.8	5.7	81.7	1	0	1	0	1	1	1	13.1	0.08	15.2
1	0	0	100	5.06	1	69	24.5	130	84	136	45	91	13.7	5.5	70.6	2	1	0	1	1	1	1	3.6	0.04	21
1	0	0	100	5.06	1	73	22	116	76	162	45	117	13.6	5.7	68.6	2	1	0	1	1	1	1	3	0.02	11.2
1	0	0	100	7	1	69	21.2	128	66.5	184	45	139	14.8	5.4	70.6	2	0	1	1	1	1	1	6	0.06	8.4
1	0	0	100	4.63	1	76	28.3	113	70.5	143	45	98	13.6	5.3	74.5	1	1	1	0	1	1	1	4.9	0.04	26.5
1	0	0	100	4.63	1	70	24.3	152.5	84	187	45	142	15.3	5.9	70.1	2	1	1	0	1	1	1	63.7	0.04	23.3
1	0	0	100	4.64	1	68	22.3	143	85	186	45	141	14	5.2	71.1	2	1	1	0	1	1	1	4.4	0.02	11.7
1	0	0	100	6.96	1	66	21	119	62	196	45	151	12.7	4.9	72.1	2	1	0	1	1	1	1	9.5	0.04	34.3
1	0	0	100	6.8	1	71	25.6	137	66	181	45	136	15.1	5.2	61.3	1	1	0	1	1	1	1	34.8	0.05	348

1	0	0	100	6.8	1	78	20.3	136	76.5	220	45	175	11.7	6.2	66.3	2	0	1	1	0	1	1	64.1	0.05	3.78
1	0	0	100	6.84	1	72	25.8	128	75	206	45	161	13.7	4.9	69.1	2	1	0	0	1	1	1	50.7	0.01	21.9
1	0	0	100	4.6	1	67	20.4	144.5	81	199	45	154	14.9	5.6	84.6	2	0	0	0	1	1	1	4.7	0.01	13.9
1	0	0	100	4.62	1	72	23.3	169.5	97.5	253	45	208	15	4.8	69.1	2	1	0	0	0	1	1	11.2	0.04	39.3
1	0	0	100	6.44	1	67	21.5	137	78	161	45	116	14.1	5.3	75.2	2	1	1	0	1	1	1	48.4	0.01	29.1
1	0	0	100	5.36	1	78	27.3	116	68.5	160	45	115	14.3	5.8	46.8	2	0	1	1	0	1	1	12.9	0.02	12.6
1	0	0	100	5.37	1	70	23.6	126	77.5	170	45	125	14.8	4.8	73.6	1	1	0	1	1	1	1	8	0.01	12.3
1	0	0	100	5.37	1	67	25.8	120	77.5	184	45	139	13.6	5.5	79.4	2	1	0	1	1	1	1	13.7	0.01	23.3
1	0	0	100	5.05	1	66	23.9	129	82	205	45	160	16.4	6.2	79.9	2	1	0	1	1	1	1	6.6	0.03	8.63
1	0	0	100	5.06	1	79	24.8	110	65.5	212	45	167	16.2	5.9	65.8	2	1	0	1	1	1	1	15.2	0.06	9.7
1	0	0	100	6.85	1	71	24	166.5	98.5	202	45	157	15.4	5.5	73.1	2	1	0	0	1	1	1	14.7	0.03	21.2
1	0	0	100	4.79	1	74	22.4	147	81	118	45	73	13.8	5	60.0	2	1	1	0	1	1	1	39.4	0.05	62.9
1	1	0	100	4.58	1	74	24.5	165	82	268	45	223	14.3	5.4	43.7	2	1	0	0	0	1	1	2046.1	0.02	15.4
1	1	0	100	2.35	1	66	22.9	123	74	214	45	169	15.1	5.5	72.1	2	1	0	1	1	1	1	4.4	0.01	12.2
1	1	0	100	1.29	1	71	23.1	129	77	213	45	168	15.9	4.8	82.2	2	1	0	0	1	1	1	10.6	0.05	5.14
1	1	0	100	1.97	1	77	22	123	78.5	161	45	116	12.7	5.5	58.8	2	1	0	1	1	1	1	13.2	0.01	17.7
1	0	0	100	7.23	2	65	34.7	142	85	219	45	174	14.1	5.3	78.0	2	1	1	0	1	1	1	14.8	0.21	45.2
1	0	0	100	7.21	2	67	21.9	107.5	66.5	179	45	134	14.7	5.6	73.1	2	1	1	1	1	1	1	12.4	0.23	13.4
1	0	0	100	7.02	2	76	22.3	102	59	173	45	128	12.1	5.7	58.4	1	1	1	0	1	1	1	15.5	1.82	78.7
1	0	0	100	7.02	2	71	23.3	124	72.5	235	45	190	10.8	5.4	71.1	2	1	1	1	0	1	1	14	0.1	15.3
1	0	0	100	5.07	2	77	23.8	154.5	90.5	204	45	159	12.5	5.2	71.7	2	1	1	0	1	1	1	7.2	0.38	19.3
1	0	0	100	5.08	2	77	23.3	117	62.5	186	45	141	12	5.6	71.7	1	1	1	0	1	1	1	27.3	0.2	22.6
1	0	0	100	4.63	2	70	21.1	135	66.5	165	45	120	12.6	5.2	71.6	2	1	0	1	1	1	1	4.5	0.28	25.9
1	0	0	100	6.95	2	69	25.5	133.5	77	208	45	163	12.7	5.3	80.5	2	1	1	1	1	1	1	119.9	0.14	1.29
1	0	0	100	6.97	2	79	23.8	102	53.5	192	45	147	12.5	5.2	70.7	1	1	1	1	1	1	1	24.8	0.17	23
1	0	0	100	6.84	2	69	27	145	69	208	45	163	13.3	5.9	80.5	2	1	1	0	1	1	1	42	0.13	16.3
1	0	0	100	4.6	2	71	25.2	162.5	84.5	196	45	151	11.7	5.5	74.8	2	1	1	0	1	1	1	386.3	0.23	53.8
1	0	0	100	4.61	2	76	25.5	120.5	69.5	241	45	196	15.2	5.9	58.4	1	0	1	0	0	1	0	41.5	0.13	71.9
1	0	0	100	7	2	66	26.7	109.5	74	218	45	173	12.4	5.4	82.3	2	1	1	1	1	1	1	21.6	0.07	9.34
1	0	0	100	7.07	2	68	25.8	132.5	86.5	223	45	178	14.4	5.1	76.4	2	1	1	1	0	1	1	29.8	0.03	3.46
1	0	0	100	6.87	2	69	21.7	115.5	74.5	243	45	198	14.7	5.4	75.9	2	1	1	1	0	1	1	9.1	0.02	41.9
1	0	0	100	6.84	2	70	27.6	160.5	77	207	45	162	13.6	5.3	71.6	2	1	0	0	1	1	1	5.4	0.04	40.4
1	0	0	100	6.85	2	76	22.2	116.5	61.5	202	45	157	12.9	5.5	68.6	1	1	1	1	1	1	1	6.9	0.06	77
1	0	0	100	6.85	2	70	36.3	160.5	81.5	239	45	194	12.8	6.8	60.9	2	1	1	0	0	0	1	269.4	0.04	38.5
1	0	0	100	6.85	2	67	21.3	128.5	85.5	260	45	215	13.6	5.8	76.9	2	1	1	0	0	1	1	13.4	0.06	3.92
1	0	0	100	6.86	2	67	20.9	141	73	276	45	231	12.2	5.4	76.9	2	1	1	0	0	1	1	14	0.01	6.99
1	0	0	100	2.88	2	70	23.7	156.5	79.5	175	45	130	12.5	5.7	71.6	1	1	1	0	1	1	1	20.5	0.03	18.3
1	0	0	100	2.95	2	66	24.1	138	69	201	45	156	13.1	5.7	73.6	2	1	1	0	1	1	1	18.4	0.02	48.7
1	0	0	100	6.98	2	75	22.6	146	75.5	228	45	183	12.9	7.8	51.0	2	1	1	0	0	0	1	5.6	0.02	20.7
1	0	0	100	4.63	2	69	23.5	175.5	93.5	187	45	142	14	5.4	75.9	2	1	1	0	1	1	1	24	0.01	52.3
1	0	0	100	6.97	2	67	19.3	98	58	208	45	163	11	5.5	76.9	2	1	1	1	1	1	1	11.2	0.01	36.9
1	0	0	100	6.83	2	72	30.4	124	64.5	194	45	149	13.2	5.7	74.3	1	1	1	0	0	1	0	116	0.08	224
1	0	0	100	4.59	2	68	27.6	127.5	70	224	45	179	14.3	5.9	81.1	2	1	1	1	0	1	1	5.7	0.06	9.57
1	0	0	100	4.61	2	71	23.6	156.5	77.5	192	45	147	12.5	5.1	71.1	1	1	1	0	1	1	1	17.9	0.01	186
1	0	0	100	4.22	2	66	27.7	164.5	74	184	45	139	14.1	5.7	77.5	2	1	1	0	1	0	1	23.3	0.04	17.1
1	0	0	100	6.44	2	71	25.9	103.5	66.5	147	45	102	11.1	5.2	74.8	2	1	1	1	1	1	1	17.2	0.01	1.02
1	0	0	100	5.37	2	73	21.3	129.5	68	192	45	147	13.3	4.7	73.8	2	1	0	0	1	1	1	9.6	0.02	81.7
1	0	0	100	5.37	2	68	23.3	117.5	57.5	204	45	159	12.4	5.2	72.6	2	1	1	1	1	1	1	15.3	0.01	9.41
1	0	0	100	5.05	2	67	28.5	191	103	254	45	209	13.7	5.2	73.1	2	1	0	0	0	1	1	55	0.02	21.6
1	0	0	100	5.06	2	69	22.9	140	82.5	259	45	214	14.7	5.2	72.1	2	1	1	0	0	1	1	7.4	0.06	20.5
1	0	0	100	6.85	2	69	21.5	168.5	85	216	45	171	13.3	5.3	61.4	2	1	1	0	1	1	1	13.3	0.03	60.2

1	0	0	100	6.86	2	66	24	99.5	63	182	45	137	13.1	4.9	77.5	2	1	0	1	1	1	1	23.8	0.04	164
1	1	0	100	4.64	2	75	24.3	114.5	64.5	168	45	123	11.9	5.9	72.7	1	1	1	0	1	1	1	6	0.48	15.5
1	1	0	100	7.05	2	77	25.5	154.5	81	198	45	153	14.5	5.3	58.0	1	1	1	0	1	1	1	35.3	0.05	14.8
1	1	0	100	6.87	2	68	25.9	119	79	182	45	137	12.8	5.8	76.4	2	1	1	1	1	1	1	12.7	0.03	11.5
1	1	0	100	6.88	2	74	18.9	89	43	171	45	126	11.4	5.7	59.2	2	1	1	1	1	1	1	6.2	0.07	47.4
1	1	0	100	6.98	1	80	22.5	143	76	190	45	145	14.7	5.7	72.5	1	1	0	0	1	1	1	11.9	0.47	26
1	1	0	100	6.84	1	81	20.8	138	70.5	157	45	112	12.7	5.3	68.1	2	1	1	0	1	1	1	2.4	0.16	42
1	0	0	100	0.68	2	80	18.7	116	61	178	45	133	12.3	5.6	70.2	2	1	1	1	1	1	1	15.1	1.03	29.9
1	1	0	100	4.59	2	80	27.8	124	66	194	45	149	12.5	5	43.4	1	1	1	0	1	1	1	11.6	0.05	156
1	0	0	100	6.44	1	66	24.4	116	75.5	131	45	86	15	5.5	75.7	2	1	1	1	1	1	1	9.1	0.14	10.8
1	0	0	100	6.86	1	74	22.4	146.5	91	192	45	147	14.9	5.6	75.6	2	1	1	0	1	1	1	11.6	0.1	24.3
1	0	0	100	5	1	68	27.1	168.5	86.5	195	45	150	15.8	4.9	71.1	2	0	0	0	1	1	1	96.6	0.05	12.7
1	0	0	100	5.85	1	75	23.4	107	54.5	180	45	135	12.6	7.8	67.7	2	1	1	1	1	0	1	26.3	0.07	121
1	0	0	100	4.62	1	68	24.5	117.5	74.5	144	45	99	15.5	5.2	71.1	2	1	1	1	1	1	1	5.3	0.02	13.6
1	0	0	100	5.37	1	73	21.7	122.5	73	183	45	138	14.5	5.4	37.0	2	1	0	0	1	1	1	3.2	0.07	22.9
1	0	0	100	6.87	2	70	24.2	160.5	85.5	199	45	154	14.1	5.7	71.6	2	1	1	0	1	1	1	24.7	0.11	15.4
1	0	0	100	5.16	2	67	23.3	122	68	206	45	161	10.6	5.5	76.9	2	1	1	0	1	1	1	11.8	2.32	43
1	0	0	100	5.16	2	74	24	135	74.5	218	45	173	13.3	6.2	69.6	1	1	1	0	1	1	1	24.9	0.57	52.3
1	0	0	100	5.08	2	79	26.1	146.5	71.5	137	45	92	11.9	5.6	67.2	1	1	1	0	1	1	1	20.7	0.14	16.7
1	0	0	100	6.99	2	70	29.5	110.5	66	165	45	120	9.6	5.8	80.0	1	1	1	0	1	1	1	13.6	0.1	12.5
1	0	0	100	0.33	2	75	21.9	137	70	131	45	86	8.1	4.9	69.1	2	1	1	0	1	1	1	68.7	0.35	41.8
1	0	0	100	6.84	2	72	27	134	77	228	45	183	15.1	5.5	74.3	2	1	1	1	0	1	1	52.4	1.07	0.41
1	0	0	100	4.6	2	67	27.1	162.5	95	185	45	140	14.8	5.9	76.9	2	1	1	0	1	1	1	15.8	0.14	28.8
1	0	0	100	4.68	2	72	30.5	165.5	82	164	45	119	13.6	7.3	70.6	1	1	1	0	1	0	1	49.9	0.1	19
1	0	0	100	4.69	2	71	28.2	168.5	89	146	45	101	13.2	5.9	74.8	2	1	1	0	0	1	1	13.6	0.1	45.6
1	0	0	100	6.97	2	67	26.3	143.5	83.5	164	45	119	11.9	5.6	73.1	2	1	1	0	1	1	1	71.4	0.11	14.6
1	0	0	100	6.87	2	72	33.3	145.5	80.5	191	45	146	14.7	6.3	70.6	2	1	1	0	1	1	1	23.1	0.24	83.1
1	0	0	100	7.08	2	73	19.1	112.5	63.5	207	45	162	12	5.4	73.8	1	1	1	1	1	1	1	27.7	0.02	18.1
1	0	0	100	6.86	2	70	20.8	137	74.5	187	45	142	14.1	5.3	71.6	2	1	1	1	1	1	1	19.3	0.05	0.05
1	0	0	100	5.15	2	77	26.7	128.5	85	204	45	159	14.4	6.7	71.7	1	1	1	0	1	0	1	16.8	0.07	33.6
1	0	0	100	6.86	2	71	25.1	110	66.5	241	45	196	14	5.6	79.4	1	1	1	0	0	1	1	38.3	0.05	54.7
1	0	0	100	6.98	2	68	27.4	129.5	76	229	45	184	13.3	5.8	76.4	2	1	1	1	0	1	1	58.9	0.03	2.14
1	0	0	100	6.98	2	69	29.7	166.5	89.5	175	45	130	12.1	5.3	72.1	1	1	1	0	0	1	1	14.9	0.08	64.8
1	0	0	100	7	2	70	24.9	110	73	161	45	116	10.4	4.9	75.3	2	1	1	1	1	1	1	17.4	0.03	18.4
1	0	0	100	6.8	2	71	27.4	108.5	68	204	45	159	13	5.6	71.1	2	1	1	0	1	1	1	6.9	0.02	22.5
1	0	0	100	6.87	2	67	24.4	119	69	237	45	192	13.6	5.1	76.9	2	1	1	1	0	1	1	10.7	0.04	14.4
1	1	0	100	6.86	2	76	31.8	140	73	187	45	142	12.3	5.6	68.6	2	1	1	0	1	1	1	13.6	0.03	128
1	1	0	100	6.82	2	78	30.1	114	63.5	164	45	119	11.8	5.2	71.2	1	1	1	0	1	1	1	29.3	0.03	25.1
1	1	0	100	5.37	2	70	22.9	101	58	131	45	86	10.9	5	60.9	2	1	1	1	1	1	1	13.1	0.02	10.1
1	1	0	100	6.95	1	80	25.7	138	86	191	45	146	16	5.6	72.5	2	1	0	1	1	1	0	20.9	0.33	119
1	1	0	100	6.87	1	80	21.4	127	56	171	45	126	13	5.8	72.5	2	1	1	0	1	1	1	34.7	0.02	192
1	1	0	100	6.98	2	86	25.3	143.5	82	201	45	156	10.5	6.4	47.2	1	1	1	0	1	1	1	31.6	0.91	0.14
1	0	1	2.7	2.7	1	75	20.2	152.5	89	183	45	138	12.5	5.3	80.0	1	0	0	0	1	1	1	67.1	0.37	15.1
1	0	1	2.7	2.7	1	69	28	127	75.5	253	45	208	13.4	6.2	62.2	2	1	1	0	0	1	1	24.7	0.46	7.29
1	0	1	2.9	2.9	1	69	22.6	151.5	80	220	45	175	14.2	5.9	74.1	2	0	1	0	0	1	1	403.9	0.08	26.1
1	1	1	2	2	1	74	20.7	141.5	72	207	45	162	13.6	5.3	71.5	2	0	1	0	1	1	1	9	0.15	7.66
1	1	1	0.7	0.7	2	72	25.7	165.5	84.5	191	45	146	13.2	5.6	74.3	2	1	1	0	1	1	1	14.3	0.04	66
1	1	1	2.3	2.3	1	79	25.4	145.5	85	190	45	145	15.6	5.3	73.0	2	1	1	0	1	1	1	186.1	0.56	37.8
1	0	1	3.1	3.1	2	69	28.2	136	80.5	250	45	205	13.1	5.5	75.9	1	1	0	0	0	1	1	271	0.06	20.9
1	0	0	100	7.05	1	73	31	123.5	83	159	46	113	15	3.6	60.4	2	1	0	0	1	1	0	156.5	0.15	18.1
1	0	0	100	7.06	1	77	24.1	157.5	84.5	213	46	167	12.7	5.7	66.7	2	1	0	0	1	1	1	22.2	0.27	13.4

1	0	0	100	7.07	1	71	23.8	90.5	59.5	254	46	208	14.5	5.1	49.2	2	0	1	1	0	1	1	3	0.12	34.3
1	0	0	100	0.72	1	68	18.6	112	70	166	46	120	12.3	6.2	78.8	2	0	1	1	1	1	1	12.4	2.34	15.4
1	0	0	100	6.84	1	65	23.9	116.5	63	206	46	160	14.1	6	80.5	2	1	0	1	1	1	1	19.2	0.11	28
1	0	0	100	7.2	1	65	29	140	95.5	205	46	159	14.5	5.8	80.5	2	1	0	0	1	1	1	13.3	0.18	26.3
1	0	0	100	7.04	1	65	33.3	159	82.5	216	46	170	16	5.1	76.2	2	1	1	0	1	1	1	5.1	0.17	12.5
1	0	0	100	5.06	1	72	22.5	155.5	83	224	46	178	15.4	6.2	72.6	1	1	0	0	0	1	1	27.8	0.21	27.5
1	0	0	100	5.07	1	67	26	112	68	165	46	119	16.4	5.6	71.6	2	1	0	0	1	1	1	4.1	0.32	97.7
1	0	0	100	6.84	1	68	18.5	93.5	50.5	139	46	93	12.8	5.7	74.6	2	1	0	1	1	1	1	83	0.25	27.8
1	0	0	100	2.21	1	77	20.8	136	82	139	46	93	14.5	6.7	70.1	2	0	0	0	1	0	1	113	0.12	10.3
1	0	0	100	7.05	1	73	23.9	136	71	195	46	149	14	5.1	68.6	1	1	1	1	1	1	1	6.7	0.02	24.4
1	0	0	100	4.58	1	71	26.9	135.5	84	211	46	165	15.5	5.4	61.3	2	1	0	0	1	1	1	72.2	0.01	14.5
1	0	0	100	4.59	1	70	23.1	125	80.5	196	46	150	13.5	5.7	70.1	2	1	0	1	1	1	1	8.8	0.02	7.69
1	0	0	100	4.59	1	72	26.2	121	68	193	46	147	14.8	5	72.6	2	1	0	0	1	1	1	44.2	0.01	19.8
1	0	0	100	6.87	1	67	23.5	142	70.5	205	46	159	15.2	5.4	79.4	2	0	1	0	1	1	1	6.4	0.08	27.7
1	0	0	100	5.16	1	67	26.8	157	84.5	219	46	173	15.3	5.1	63.0	2	1	0	0	1	1	1	17.3	0.07	13.6
1	0	0	100	6.84	1	65	26.5	129	90	255	46	209	15.9	6.8	57.0	2	1	0	0	0	0	1	9.4	0.05	7.23
1	0	0	100	6.85	1	68	25.5	127	88.5	227	46	181	14.8	5.1	78.8	2	1	0	0	0	1	1	5.2	0.08	1.54
1	0	0	100	7.01	1	66	22.3	141.5	74.5	167	46	121	13.8	7.3	85.2	2	1	0	0	1	0	1	8.1	0.02	35.3
1	0	0	100	1.83	1	66	26.6	115.5	72	196	46	150	12.7	6.4	75.7	2	1	0	1	1	0	1	4.2	0.06	41.5
1	0	0	100	5.05	1	71	26.9	157.5	89.5	145	46	99	16.2	5.3	69.6	1	1	1	0	1	1	1	11.3	0.07	17.6
1	0	0	100	5.05	1	67	22.1	179	92	145	46	99	14.3	7	71.6	2	1	1	0	1	0	1	29.4	0.01	38.1
1	0	0	100	5.05	1	72	24.3	117	72.5	197	46	151	14.9	5.6	69.1	2	1	0	1	1	1	1	6.6	0.03	20.4
1	0	0	100	5.08	1	67	26.4	121	75.5	166	46	120	15.7	5.1	75.2	2	1	0	1	1	1	1	2.6	0.03	27.6
1	0	0	100	4.62	1	77	27	148.5	90	198	46	152	15.7	5.6	66.7	2	1	0	0	1	1	1	74.8	0.01	21.6
1	0	0	100	4.64	1	78	20.6	146.5	78.5	193	46	147	11.9	5.8	69.6	2	1	1	0	1	1	1	8.2	0.01	19.4
1	0	0	100	4.64	1	71	24.1	139	82	158	46	112	14.1	5.2	73.1	1	1	1	1	1	1	1	42.4	0.02	19.4
1	0	0	100	6.96	1	75	20.6	154.5	78	237	46	191	13.9	5	67.7	2	0	1	0	0	1	1	3.9	0.02	26.4
1	0	0	100	6.83	1	70	22.5	106	66.5	141	46	95	12.6	5.2	73.6	2	0	1	1	1	1	1	5.1	0.02	16.6
1	0	0	100	4.6	1	73	20.9	136.5	65	233	46	187	13.8	5.7	60.4	2	0	1	1	0	1	1	151.3	0.03	42.5
1	0	0	100	4.6	1	75	21.2	119.5	66	171	46	125	15.2	5.5	71.0	2	1	0	0	1	1	1	7	0.06	25
1	0	0	100	4.62	1	71	26.3	121	74	231	46	185	15.9	5.8	69.6	2	0	1	1	0	1	1	18.6	0.06	25.3
1	0	0	100	4.62	1	78	21.7	113.5	69	175	46	129	14.3	5.7	69.6	1	1	1	1	1	1	1	15.4	0.04	25.5
1	0	0	100	4.69	1	68	23.8	111	75	194	46	148	15.2	5.1	78.8	2	1	1	1	1	1	1	38.9	0.03	11
1	0	0	100	6.85	1	65	21	153	84.5	175	46	129	14.2	4.9	72.6	2	1	1	0	1	1	1	13.3	0.03	12
1	0	0	100	6.86	1	73	24.7	128	79	200	46	154	13.9	5.1	72.0	2	0	0	1	1	1	1	9.7	0.07	33
1	0	0	100	5.04	1	70	31.4	119.5	73.5	217	46	171	15.2	7.3	73.6	2	1	1	0	1	0	1	15.8	0.04	18.6
1	0	0	100	5.04	1	71	22.7	145.5	81	204	46	158	15.8	6.3	49.2	1	0	0	0	1	1	1	6.3	0.05	20.4
1	1	0	100	6.87	1	74	27.7	138	78.5	195	46	149	15.7	6.8	71.5	1	1	1	1	1	0	1	69.9	0.1	7.34
1	1	0	100	6.86	1	77	25.4	115	69	189	46	143	13.4	5.4	58.8	2	1	0	1	1	1	1	6	0.18	12.5
1	1	0	100	7	1	75	26.2	137	76	232	46	186	15.2	6	75.1	2	1	0	1	0	0	1	47.2	0.05	15.7
1	0	0	100	4.58	2	68	22.8	112	62.5	184	46	138	12.4	5.4	76.4	2	1	1	1	1	1	1	6.7	0.51	88.2
1	0	0	100	6.87	2	67	28.4	148	86.5	257	46	211	15.4	6	73.1	2	1	1	0	0	1	1	316	0.13	41.2
1	0	0	100	7.04	2	65	27.8	120.5	76	237	46	191	13.7	6	74.2	2	1	1	0	0	1	1	5.4	0.14	0.98
1	0	0	100	6.98	2	67	25.3	128	72.5	222	46	176	13.3	5.4	73.1	1	1	1	0	0	1	1	3	0.11	4.96
1	0	0	100	4.63	2	77	24.6	147	90	162	46	116	11.4	5.5	68.2	1	1	1	0	1	1	1	33.2	0.29	30.1
1	0	0	100	6.98	2	72	19.5	142.5	78	265	46	219	13.3	5.3	52.1	2	1	1	0	0	1	1	18.6	0.38	48.4
1	0	0	100	4.6	2	74	27.7	141.5	90	220	46	174	14.2	5.3	73.2	2	1	1	0	0	1	1	15.2	0.13	4.88
1	0	0	100	5.06	2	65	20.8	86.5	52	210	46	164	12.5	5.5	78.0	2	1	0	1	0	1	1	39	0.18	13.9
1	0	0	100	7.05	2	71	24.6	144.5	87.5	251	46	205	14.8	6.6	71.1	2	1	1	0	0	0	1	21	0.08	15.1
1	0	0	100	4.58	2	77	21.1	144	80	193	46	147	13.4	4.8	76.1	2	1	1	0	1	1	1	16.6	0.07	14.7
1	0	0	100	0.27	2	73	23.3	143.5	87	268	46	222	13	5.3	59.7	2	1	1	0	0	1	1	12.3	0.08	10.8

1	0	0	100	4.59	2	65	22.4	114	66.5	179	46	133	12.5	5.1	63.1	2	1	1	1	1	1	1	11.2	0.03	16.1
1	0	0	100	4.59	2	65	23.9	140.5	95.5	237	46	191	13.6	6	74.2	2	1	1	0	0	1	1	189.7	0.05	9.93
1	0	0	100	4.59	2	65	26.3	96.5	64.5	213	46	167	14.5	5.4	74.2	2	1	1	1	1	1	1	9.8	0.05	6.01
1	0	0	100	6.86	2	71	17.6	90.5	51.5	193	46	147	11.3	5.2	60.5	1	1	1	1	1	1	1	6.8	0.01	66.8
1	0	0	100	6.87	2	68	25.1	104	63	184	46	138	13.1	4.9	76.4	2	1	1	1	1	1	1	8.4	0.09	37.3
1	0	0	100	6.85	2	76	23.5	141	69.5	216	46	170	13.7	5	68.6	2	1	1	0	1	1	1	51.5	0.05	17.2
1	0	0	100	7	2	73	28.1	110.5	65.5	163	46	117	13.6	6.5	73.8	1	1	0	1	1	0	1	7.9	0.09	11
1	0	0	100	5.07	2	66	30.4	120	70.5	159	46	113	13.3	5.7	77.5	2	1	1	0	1	1	1	4	0.07	12.1
1	0	0	100	7	2	73	20.5	87	53.5	184	46	138	12.4	5.5	78.3	2	1	1	1	1	1	1	13	0.03	41.4
1	0	0	100	7	2	70	25.2	134	77	238	46	192	12.5	5.3	80.0	2	1	1	1	0	1	1	6.8	0.02	44.7
1	0	0	100	7	2	68	31.2	162	88	183	46	137	11.8	5.4	76.4	2	1	1	0	1	1	1	24.7	0.01	51.2
1	0	0	100	4.63	2	68	26.8	132	78.5	202	46	156	14.1	5.9	61.8	2	1	1	0	0	1	1	7.2	0.09	18.2
1	0	0	100	4.59	2	73	22.9	112	65	209	46	163	14.4	5.3	73.8	1	1	1	0	1	1	1	11.8	0.02	33.5
1	0	0	100	4.62	2	74	25.4	135.5	84.5	219	46	173	12.8	5.4	59.2	2	1	1	1	1	1	1	39	0.03	13.1
1	0	0	100	4.62	2	68	24.5	120	71.5	185	46	139	12.5	5.1	76.4	2	1	1	1	1	1	1	10.3	0.02	27.9
1	0	0	100	4.62	2	76	21.8	128.5	63.5	190	46	144	11.4	5.5	76.7	2	1	1	1	1	1	1	7.4	0.04	63
1	0	0	100	5.05	2	70	29.5	169.5	87	185	46	139	14.6	5.2	71.6	2	1	1	0	1	1	1	178.8	0.06	39
1	0	0	100	5.06	2	68	21.6	151	80.5	195	46	149	12.8	5.7	76.4	2	1	0	0	1	1	1	13.1	0.03	14.1
1	0	0	100	6.85	2	66	27.2	135	84.5	211	46	165	14.3	5.4	73.6	2	1	1	1	1	1	1	26.7	0.03	12.9
1	0	0	100	6.87	2	78	25.6	120	61.5	230	46	184	14.1	5.9	67.7	1	1	1	0	0	1	1	137.3	0.06	15.4
1	0	0	100	5.04	2	67	22.1	126.5	71.5	197	46	151	11.5	5.7	62.2	2	1	0	1	1	1	1	60.4	0.01	26
1	1	0	100	7	2	74	30.5	134.5	71.5	172	46	126	11	5.6	73.2	1	1	1	0	0	1	1	6.1	0.14	12.3
1	1	0	100	7.02	2	75	24.1	138.5	69	209	46	163	11.1	6.1	72.7	2	1	1	0	1	1	1	243.9	0.06	148
1	1	0	100	6.8	2	72	24.1	124	71.5	182	46	136	13.8	6.5	74.3	2	0	1	0	1	0	1	17.8	0.08	28
1	0	0	100	7.06	1	80	19.5	158.5	88	234	46	188	12.9	5.4	57.5	1	1	0	0	0	1	1	12.1	0.16	42.2
1	0	0	100	6.85	1	81	29.4	134.5	72	195	46	149	15.3	5.1	57.1	2	1	1	0	1	1	1	20.8	0.29	66.1
1	0	0	100	6.86	1	81	23.8	156	82	208	46	162	13.8	4.7	64.9	1	1	1	0	1	1	1	22.2	0.11	30.2
1	0	0	100	4.58	1	88	25.5	181.5	80	155	46	109	15.1	7.3	61.8	2	1	1	0	1	0	1	191.5	0.03	76.9
1	0	0	100	5.16	1	80	21.7	113	50.5	157	46	111	13.6	5.8	51.3	2	1	0	0	1	1	1	52.8	0.01	40.3
1	0	0	100	1.89	1	82	23.1	138.5	73.5	172	46	126	12.1	6.5	67.6	2	1	0	0	1	0	0	32.6	0.04	109
1	0	0	100	6.96	1	80	22.4	110.5	60.5	159	46	113	14.2	4.9	65.4	2	0	0	1	1	1	1	11.8	0.09	13.5
1	1	0	100	6.86	1	83	20.9	161.5	75.5	246	46	200	14.1	5.1	71.0	1	1	1	0	0	1	1	15.9	0.04	56.2
1	0	0	100	6.12	2	84	20.5	146.5	81	157	46	111	11.5	6.8	64.9	1	1	1	0	1	0	1	17.4	0.17	111
1	0	0	100	7.07	2	83	26	121.5	71.5	199	46	153	14.7	5.7	68.7	2	1	1	0	1	1	1	127.8	0.09	16.6
1	0	0	100	6.8	2	80	27.8	155	80	196	46	150	12.9	4.9	49.3	2	1	1	0	1	1	1	17.7	0.08	34
1	0	0	100	6.87	2	80	28	135	71.5	174	46	128	12.1	8.7	74.6	1	1	1	0	1	0	1	67.3	0.05	26.8
1	0	0	100	7.06	1	69	23.5	138.5	82.5	217	46	171	14.7	5.7	74.1	2	0	1	1	1	1	1	9.1	0.11	11.8
1	0	0	100	4.63	1	78	25.6	111.5	62.5	139	46	93	16.6	5.1	58.3	2	1	1	1	1	1	1	11.4	0.15	86.1
1	0	0	100	4.69	1	70	22.5	123.5	80	175	46	129	13.7	5.8	77.7	2	1	0	1	1	1	1	7.2	0.28	53.6
1	0	0	100	5.16	1	71	23.6	139	81	190	46	144	9.7	5.8	88.6	2	1	1	1	1	1	1	58.6	0.04	19.3
1	0	0	100	6.85	1	71	23	133.5	66	177	46	131	13.4	5.7	73.1	2	0	1	1	1	1	1	17.7	0.04	38.8
1	0	0	100	6.98	1	76	25.6	123.5	67	230	46	184	13.3	6.1	74.5	2	1	1	1	0	1	1	11.2	0.01	40.2
1	0	0	100	4.69	1	71	24.9	156	96	197	46	151	15.6	5.1	61.3	1	1	0	0	1	1	1	9.8	0.06	9.45
1	0	0	100	6.86	1	69	21.9	198.5	92.5	141	46	95	15.1	5.8	74.1	2	0	0	0	1	1	1	15.1	0.05	139
1	1	0	100	2.22	1	78	22.9	159.5	78.5	131	46	85	13.9	6.2	69.6	2	1	0	0	1	1	1	16.9	0.47	38.9
1	1	0	100	6.44	1	76	18.9	125.5	77	177	46	131	14	5.8	70.5	1	0	1	1	1	1	1	130.2	0.39	18.1
1	1	0	100	7.01	1	67	30.8	124	69	192	46	146	14.6	5.3	75.2	1	0	0	1	1	1	1	7.1	0.04	13.1
1	0	0	100	7	2	77	21.6	131.5	79	129	46	83	12.1	4.4	68.2	1	1	1	1	1	1	1	46.3	0.11	60.2
1	0	0	100	5.06	2	73	21.7	142.5	70.5	190	46	144	12.5	5.9	78.3	2	1	1	0	1	1	1	50.2	0.5	32
1	0	0	100	5.08	2	73	28.6	123.5	74.5	243	46	197	12.6	5.6	70.1	1	1	1	0	0	1	1	26	0.11	14.2
1	0	0	100	6.95	2	74	21.2	137.5	69.5	202	46	156	13.6	6	73.2	2	1	1	0	1	1	1	223.5	0.23	56.4

1	0	0	100	6.8	2	65	22.8	132	70.5	155	46	109	12.7	6.1	78.0	2	1	1	1	1	0	1	9.8	0.34	18.5
1	0	0	100	6.8	2	73	27.5	133	76	192	46	146	13.4	5.8	78.3	2	1	1	0	1	1	1	13	0.15	10.3
1	0	0	100	4.6	2	69	27.1	156.5	83.5	157	46	111	11.6	5.4	80.5	2	1	1	0	1	1	1	21.4	0.15	98
1	0	0	100	6.86	2	71	31	129	73	216	46	170	14.3	5.3	71.1	2	1	1	0	1	0	1	19.2	0.25	21.2
1	0	0	100	6.86	2	72	28.1	144.5	75	237	46	191	11.6	5.1	74.3	1	1	1	0	0	1	1	26	0.07	22.3
1	0	0	100	1.08	2	71	22	153	88.5	199	46	153	13.3	5.6	52.5	1	1	1	0	1	1	1	30.7	0.02	57.3
1	0	0	100	6.84	2	76	23.3	124	61	236	46	190	9.1	5.4	50.7	1	1	1	0	0	1	1	9.3	0.05	38.9
1	0	0	100	6.86	2	68	26.8	122.5	76	220	46	174	11.7	5.2	81.1	2	1	1	1	0	1	1	118.4	0.05	20
1	0	0	100	7.01	2	67	30.1	125	77	198	46	152	13.8	5.4	73.1	2	1	1	1	1	1	1	77.1	0.03	5.29
1	0	0	100	7.03	2	73	15.9	100	58	204	46	158	11.6	5.4	73.8	2	1	1	1	1	1	1	13.7	0.01	6.45
1	0	0	100	6.98	2	66	19.6	156	83.5	180	46	134	13.5	6.2	73.6	2	1	1	0	1	0	1	11.1	0.04	27.2
1	0	0	100	7	2	75	24	91.5	46.5	162	46	116	12.7	5.1	83.1	1	1	1	1	1	1	1	9	0.04	17.8
1	0	0	100	4.63	2	75	31.2	124.5	63.5	185	46	139	12.4	5.8	72.7	1	1	1	1	1	1	1	29.9	0.06	17.8
1	0	0	100	4.64	2	78	19.1	144.5	76.5	156	46	110	12.5	5.4	75.6	1	1	1	0	1	1	1	29.1	0.03	28.8
1	0	0	100	6.87	2	68	20.4	110.5	68	187	46	141	11.5	5.3	76.4	2	1	1	1	1	1	1	8.6	0.04	32.5
1	0	0	100	5.06	2	74	25.6	139	70	161	46	115	13.8	5.8	83.7	1	1	1	1	1	1	1	19.2	0.01	64
1	0	0	100	5.06	2	74	23.8	118	70.5	221	46	175	12.9	5.5	73.2	2	1	1	1	0	1	1	52.3	0.05	41.5
1	1	0	100	6.85	2	77	23.1	136	69.5	185	46	139	8.7	5.8	50.3	1	1	1	0	1	1	1	9.8	0.24	53.5
1	1	0	100	4.58	2	76	22.4	120.5	68.5	190	46	144	11.2	5.1	58.4	2	1	1	1	1	1	1	16.1	0.03	70
1	1	0	100	6.8	2	74	23.3	132.5	77	183	46	137	13.9	5.6	77.8	2	1	1	0	1	1	1	39.1	0.05	3.48
1	1	0	100	6.79	2	77	22.6	128.5	72	173	46	127	13.6	6	44.3	2	1	1	1	1	1	1	24.9	0.02	27.3
1	0	0	100	7.08	1	81	21.3	110	63	157	46	111	13.1	5.5	64.9	2	0	1	1	1	1	1	6.2	0.08	22.4
1	0	0	100	5.16	1	82	21.8	111.5	57.5	172	46	126	14.1	5.7	64.4	2	1	0	1	1	1	1	7.8	0.06	77.4
1	1	0	100	5.68	1	89	24.8	128	61	225	46	179	12	5.4	54.0	2	1	0	1	0	1	1	21.7	0.06	7.51
1	0	0	100	6.83	2	81	26.1	145	83	202	46	156	14.2	5.7	48.9	1	1	1	0	1	1	1	11	0.09	1.83
1	1	0	100	4.58	2	83	24.3	114	65	221	46	175	11.6	6.4	68.7	2	1	1	1	0	1	1	48.4	0.04	99
1	0	1	7	7	1	70	27.4	127	70.5	166	46	120	15.4	6.3	70.1	2	1	1	0	1	1	0	44.6	0.14	176
1	0	1	1.5	1.5	1	78	27.5	128.5	69.5	188	46	142	12.3	5.5	73.5	2	1	1	0	1	1	1	83.4	0.02	64.9
1	0	1	0.5	0.5	1	67	23	154	94.5	142	46	96	14	5	63.0	2	1	0	0	1	1	1	13.8	0.06	38.5
1	1	1	3	3	1	71	19.5	110.5	60.5	123	46	77	11.9	5.1	69.6	2	1	1	1	1	1	1	22.8	0.01	41.5
1	0	1	3.8	3.8	2	76	27.7	155.5	81.5	201	46	155	13.8	6.2	68.6	2	1	1	0	1	1	1	55.1	0.01	44.5
1	0	1	5.8	5.8	2	80	22.7	91	54	181	46	135	11.3	5.8	70.2	1	1	1	1	1	1	1	20.4	0.28	59.7
1	1	1	1.2	1.2	1	74	23.1	129	65.5	237	46	191	13.7	7	75.6	2	1	1	1	0	0	1	22.8	0.13	50.1
1	0	1	5.2	5.2	2	76	17.9	114.5	56.5	193	46	147	10.1	5.3	23.5	2	1	1	1	1	1	1	31	0.04	87.5
1	0	0	100	7.07	1	66	24.7	131	69	227	47	180	13.7	5	72.1	2	1	0	1	0	1	1	306.4	0.12	12.2
1	0	0	100	7.08	1	70	22.3	118.5	69.5	177	47	130	13.6	5.2	73.6	2	1	1	0	1	1	1	6.9	0.16	27.3
1	0	0	100	5.16	1	76	23.9	117	75	233	47	186	14.1	5.9	70.5	2	1	1	1	0	1	1	40.4	0.12	12.4
1	0	0	100	1.09	1	77	25	137	74	220	47	173	13.1	5.7	70.1	1	1	0	0	0	1	1	60.2	3.1	17.2
1	0	0	100	4.59	1	68	22.2	141	87.5	219	47	172	16	6.1	74.6	2	1	1	0	1	1	1	25.7	0.26	20.5
1	0	0	100	4.59	1	67	21.9	114	73	161	47	114	12.9	5.2	75.2	2	1	1	0	1	1	1	12.4	0.1	63.6
1	0	0	100	4.58	1	71	25.9	137	81	197	47	150	14.1	5.8	73.1	2	0	0	1	1	1	1	7.2	0.27	26
1	0	0	100	6.44	1	68	23.5	124	69.5	163	47	116	16.1	6	84.0	2	0	0	1	1	1	1	5.6	0.17	6.73
1	0	0	100	4.58	1	71	20	179	93.5	150	47	103	14.7	5.2	73.1	2	0	0	0	1	1	1	5.1	0.03	46.8
1	0	0	100	4.58	1	66	27.2	136	80	221	47	174	15.9	5.2	79.9	2	1	1	1	0	1	1	6.8	0.03	16.1
1	0	0	100	1.87	1	67	20.9	135	94.5	178	47	131	16.4	5.4	79.4	2	1	0	0	1	1	1	10.6	0.05	2.35
1	0	0	100	4.59	1	68	24.9	147.5	99.5	196	47	149	12.8	5.9	50.2	2	1	0	0	1	1	0	10.5	0.05	76
1	0	0	100	6.87	1	74	26.9	145.5	87	205	47	158	14.5	5.6	80.5	2	0	1	0	1	1	1	22.7	0.02	35.3
1	0	0	100	6.88	1	70	24.2	144	82.5	189	47	142	14.7	5.6	70.1	1	1	0	0	1	0	1	11.3	0.03	62.3
1	0	0	100	6.88	1	65	24.2	140	81.5	201	47	154	15.3	5.3	76.2	2	1	0	0	1	1	1	6.6	0.01	12.5
1	0	0	100	6.86	1	75	25.2	121	73	151	47	104	14.2	6.3	59.6	2	1	0	1	1	0	1	19	0.03	9.98
1	0	0	100	7.01	1	76	26.5	132.5	82.5	212	47	165	14.5	5.6	74.5	1	1	1	1	1	1	1	8.2	0.04	22.5

1	0	0	100	4.62	1	73	21	150	81.5	198	47	151	14.6	5	76.1	2	0	1	0	1	1	1	37.1	0.06	13.5
1	0	0	100	4.63	1	74	22.7	156.5	94	195	47	148	14.5	5.7	71.5	2	1	1	0	1	1	1	200	0.04	28.2
1	0	0	100	3.37	1	67	23	125	81.5	171	47	124	15.2	5.1	71.6	2	0	0	0	1	1	1	5.4	0.04	47.1
1	0	0	100	6.87	1	70	21.8	131.5	77.5	189	47	142	14.2	5.5	73.6	2	1	0	1	1	1	1	4	0.02	2.47
1	0	0	100	6.98	1	68	24.7	151	74	185	47	138	15	5.9	78.8	2	1	0	0	0	0	1	44.8	0.08	69.9
1	0	0	100	6.8	1	68	26.6	113.5	70	204	47	157	17.1	5.8	74.6	2	1	1	1	1	1	1	21.3	0.04	2.04
1	0	0	100	6.8	1	72	28.4	116.5	73	258	47	211	15.7	6.2	72.6	2	0	0	0	0	1	1	27.7	0.05	12.1
1	0	0	100	6.83	1	71	23.5	92.5	57.5	140	47	93	12.9	5.5	69.6	2	1	1	1	1	1	1	12.9	0.01	19.8
1	0	0	100	4.61	1	70	28.8	116	70	185	47	138	13.5	7.3	73.6	2	1	0	1	1	0	1	14.3	0.08	22
1	0	0	100	4.69	1	70	24.3	145.5	86	156	47	109	15.6	5.6	73.6	2	1	0	0	1	1	0	33.4	0.01	40.8
1	0	0	100	5.37	1	67	24.3	122	77.5	173	47	126	15	5.1	71.6	2	1	0	1	1	1	1	72.6	0.04	3.72
1	0	0	100	5.05	1	65	25.4	121.5	75.5	184	47	137	14.4	5.6	72.6	2	1	0	1	1	1	1	15.3	0.07	8.73
1	0	0	100	6.86	1	66	23.2	127.5	73.5	158	47	111	15.3	4.9	75.7	2	0	0	1	1	1	1	4.5	0.08	28.1
1	0	0	100	6.87	1	79	25.6	118	65.5	195	47	148	12.8	5.6	69.1	2	1	1	1	1	1	1	8.1	0.03	15.2
1	1	0	100	4.69	1	69	21.8	167	86.5	178	47	131	12.3	5.7	78.3	2	0	1	0	1	1	1	143.6	0.19	28.9
1	1	0	100	6.87	1	77	25.7	144.5	81	175	47	128	14.6	6.1	70.1	2	0	0	0	1	1	1	18.2	0.56	25.8
1	1	0	100	7.06	1	78	19.4	142.5	76.5	141	47	94	13.6	4.8	69.6	2	0	0	0	1	1	1	349.3	0.08	77.6
1	1	0	100	4.63	1	71	24.1	125.5	69.5	190	47	143	14.2	6.1	82.2	2	1	1	1	1	1	1	13.5	0.03	17.3
1	0	0	100	7.06	2	75	18.6	114	65.5	207	47	160	12.8	7.8	72.7	1	1	1	1	1	0	1	49.2	3.29	144
1	0	0	100	5.16	2	69	35.2	115.5	70.5	197	47	150	12.3	6.2	72.1	2	1	1	0	1	1	1	12.8	0.13	35
1	0	0	100	6.85	2	67	30.6	129.5	71	235	47	188	14.8	5.8	62.2	2	0	0	1	0	1	1	14	0.22	11
1	0	0	100	6.86	2	74	26	124.5	67	185	47	138	12.2	8.2	59.2	2	1	1	1	1	0	1	10.8	0.11	31.8
1	0	0	100	5.06	2	65	25	124	83	188	47	141	11.5	5	82.8	2	1	1	1	1	1	1	40	0.1	22
1	0	0	100	5.08	2	67	31.5	147.5	88	206	47	159	13.8	6	73.1	2	1	1	0	1	1	1	21.2	0.19	9.36
1	0	0	100	2.22	2	73	25.7	145	76	203	47	156	12.9	5.3	78.3	1	1	1	0	1	1	1	43.2	0.19	5.43
1	0	0	100	4.63	2	73	27.4	136.5	73	215	47	168	13.8	6.6	51.7	1	1	1	0	0	0	1	24.5	0.1	101
1	0	0	100	4.63	2	75	25.2	133.5	71	168	47	121	13.9	5.2	69.1	2	1	1	1	1	1	1	9.3	1.49	30
1	0	0	100	4.64	2	68	30.7	129	70	223	47	176	12.3	6.3	72.6	2	1	1	0	0	1	1	37.6	0.13	91.7
1	0	0	100	4.6	2	65	29.6	114	73	193	47	146	14	5.7	74.2	2	1	1	1	1	1	1	18.5	0.43	17.6
1	0	0	100	4.62	2	76	28.1	138.5	81	168	47	121	14.2	6	68.6	1	1	1	1	0	1	1	9.5	0.2	50.1
1	0	0	100	3.4	2	78	22.7	117	70.5	177	47	130	12.5	5.2	57.6	2	1	1	1	1	1	1	16.2	0.23	30.7
1	0	0	100	6.86	2	66	25.9	120.5	72.5	198	47	151	12.3	5.2	62.7	2	1	1	1	1	1	1	20.7	0.03	34.1
1	0	0	100	6.86	2	70	29.7	181.5	95	258	47	211	13.5	5.1	71.6	1	1	1	0	0	1	1	10.5	0.07	30.1
1	0	0	100	6.87	2	70	24.9	105	62	221	47	174	12.6	5.7	80.0	1	1	1	1	0	1	1	101.7	0.01	16.9
1	0	0	100	6.87	2	67	25.9	126.5	85.5	132	47	85	13.2	4.9	73.1	2	1	1	1	1	1	1	8.1	0.02	29.9
1	0	0	100	6.87	2	69	25.4	122	57.5	197	47	150	11.8	6	75.9	2	1	1	1	1	1	1	7.9	0.04	70.8
1	0	0	100	5.15	2	67	30.5	134	90	244	47	197	12.9	5.3	76.9	2	1	1	0	0	1	1	31.6	0.05	12.1
1	0	0	100	5.16	2	70	29	117	70	180	47	133	13.2	5.4	60.9	2	1	1	0	1	1	1	7.8	0.02	19.2
1	0	0	100	7.03	2	73	26.3	98.5	64	262	47	215	13.4	5.9	51.7	2	1	1	1	0	1	1	3.8	0.04	20.5
1	0	0	100	5.07	2	67	26.3	134	74	210	47	163	13.1	6	76.9	2	1	1	1	1	1	1	16.6	0.07	17.5
1	0	0	100	4.62	2	67	25.4	119.5	70	173	47	126	13.2	6.1	62.2	2	1	0	0	1	1	1	20.8	0.03	52
1	0	0	100	4.62	2	73	26.9	108	67	248	47	201	13.1	5.3	73.8	2	1	1	1	0	1	1	15.3	0.04	10.1
1	0	0	100	4.62	2	76	24.7	117	72.5	256	47	209	13.7	5.5	68.6	1	1	1	1	0	1	1	12	0.03	13.1
1	0	0	100	4.63	2	66	23	108	60	227	47	180	12.5	5.6	73.6	2	1	0	1	0	1	1	20.4	0.04	19.7
1	0	0	100	4.63	2	70	22.8	124.5	79	231	47	184	12	5.5	52.8	2	1	1	1	0	1	1	6.9	0.07	39.6
1	0	0	100	6.96	2	71	25.4	130.5	67	221	47	174	13.2	5.7	74.8	2	1	0	1	0	1	1	112.1	0.04	26.5
1	0	0	100	6.84	2	66	24.7	95	56.5	217	47	170	12.6	5.1	77.5	2	1	1	1	1	1	1	7	0.09	5.01
1	0	0	100	4.59	2	71	30.3	116	66.5	166	47	119	12.8	5.2	60.5	1	1	1	1	1	1	1	12.5	0.07	29.7
1	0	0	100	4.59	2	66	22.9	148	80.5	223	47	176	13	5.5	88.5	2	1	1	0	0	1	1	22.6	0.03	19.3
1	0	0	100	4.6	2	67	24	198	98	205	47	158	14.5	5.4	76.9	2	1	1	0	1	1	1	38.6	0.01	19.6
1	0	0	100	4.68	2	68	23.6	113.5	62	230	47	183	12.8	5.5	76.4	2	1	1	1	0	1	1	6.2	0.02	9.79

1	0	0	100	4.69	2	70	25.9	114	60.5	183	47	136	13.4	5.5	75.3	2	1	1	1	1	1	1	11.9	0.04	14.5
1	0	0	100	5.37	2	69	23.8	148.5	77.5	184	47	137	13.3	5.4	75.9	2	1	1	0	1	1	1	8.3	0.05	39.6
1	0	0	100	6.85	2	65	19	94	58	159	47	112	12.2	5.2	82.8	2	1	1	1	1	1	1	31.1	0.01	145
1	0	0	100	6.86	2	74	15.8	119	56	173	47	126	12.6	5.2	77.8	1	1	1	0	1	1	1	28.7	0.02	35.3
1	0	0	100	3.99	1	81	24.8	123	69.5	159	47	112	11.9	5.8	50.9	2	0	0	0	1	1	1	64.7	0.19	33.2
1	0	0	100	4.62	1	81	22.8	117.5	68.5	149	47	102	13.3	6	64.9	2	1	1	0	1	1	1	11.5	1.03	46.5
1	0	0	100	3.2	1	87	22.7	91.5	53.5	198	47	151	13.8	5.8	62.2	2	1	1	1	1	1	1	10.8	0.09	15.1
1	1	0	100	3.83	1	90	26.4	156	73.5	141	47	94	13.7	6.8	63.9	2	1	0	0	0	0	1	91.4	0.34	32.8
1	0	0	100	6.86	2	83	22.2	138.5	72.5	188	47	141	12.8	4.9	65.3	1	1	1	1	1	1	1	8	0.02	13.3
1	1	0	100	4.57	2	86	21	114.5	58.5	166	47	119	10.7	7.1	23.6	2	1	1	0	1	0	1	276.1	0.14	47.1
1	1	0	100	7.23	2	86	20.5	105	64.5	215	47	168	13.9	5.5	37.0	1	1	1	1	1	1	1	34.8	0.04	42.2
1	0	0	100	6.86	1	76	28.9	117	71	188	47	141	15.5	5.1	59.2	2	1	1	0	1	1	1	48.4	0.19	47.1
1	0	0	100	5.08	1	78	24.4	128	63	197	47	150	14.6	5.6	73.5	2	1	0	0	1	1	1	30.3	0.18	65.7
1	0	0	100	7	1	70	25.8	148	90	186	47	139	13.2	4.8	77.7	2	1	0	0	1	1	1	4.8	0.05	10.3
1	0	0	100	7.04	1	75	26.1	178.5	88.5	203	47	156	15.6	5.4	75.1	2	1	1	0	1	1	1	9.8	0.03	34.7
1	0	0	100	4.59	1	74	30.7	135	75	216	47	169	14.6	5.7	71.5	2	1	0	0	1	1	1	8.7	0.09	20.9
1	1	0	100	6.2	1	77	18.2	135	76.5	163	47	116	13.3	5.7	85.0	1	1	1	1	1	1	1	10.6	0.01	6.24
1	1	0	100	5.88	1	79	22.2	107	72.5	145	47	98	15	5.3	57.9	2	1	1	1	1	1	1	6	0.01	24
1	0	0	100	6.86	2	67	23.5	133.5	71.5	218	47	171	13.1	4.9	81.7	2	1	1	1	1	1	1	11.4	0.1	70.6
1	0	0	100	5.15	2	75	25.6	137	78	228	47	181	13.9	5.9	72.7	2	1	1	1	0	1	1	88.6	0.11	29.5
1	0	0	100	7.03	2	76	28.6	153.5	86.5	237	47	190	12	5.7	72.2	1	1	1	0	0	1	1	33.9	0.12	9.48
1	0	0	100	5.05	2	74	28.7	152	74	154	47	107	13.2	6.8	59.2	2	1	1	0	1	0	1	5	0.15	44
1	0	0	100	6.95	2	69	22.6	132	77	210	47	163	11.9	5.4	86.7	2	1	1	1	1	1	1	33.8	0.16	7.74
1	0	0	100	6.8	2	78	29	181.5	110.5	217	47	170	15.2	7	67.7	1	1	1	0	1	0	1	3669.4	0.74	65.6
1	0	0	100	6.87	2	73	27.7	127.5	73.5	229	47	182	12.7	5.8	59.7	2	1	1	0	0	1	1	36.9	0.23	19.2
1	0	0	100	7.06	2	75	20.2	162	81	136	47	89	12.5	5.3	77.2	2	1	1	0	1	1	1	30.5	0.02	10.5
1	0	0	100	2.39	2	70	29	128.5	88.5	236	47	189	13.9	5.5	71.6	1	1	1	1	0	1	1	18.9	0.07	10.7
1	0	0	100	4.58	2	76	26	122	75	193	47	146	12.2	5.7	68.6	1	1	1	1	1	1	1	19.8	0.07	10.4
1	0	0	100	4.59	2	75	35.8	159	97.5	168	47	121	14.9	5.7	72.7	2	1	1	0	1	1	1	141	0.09	36.9
1	0	0	100	5.56	2	77	26.8	164.5	83.5	186	47	139	13	5.2	71.7	2	1	1	0	1	1	1	86.3	0.01	102
1	0	0	100	6.88	2	65	22.8	132	68	203	47	156	13	5.1	74.2	2	1	0	1	1	1	1	15.5	0.03	46.3
1	0	0	100	5.16	2	70	26.3	103.5	61.5	211	47	164	14.4	5.7	86.1	1	1	1	0	0	1	1	229.1	0.03	16.5
1	0	0	100	6.84	2	70	20.6	114.5	59.5	227	47	180	13.8	5.2	71.6	2	1	1	1	0	1	1	7.5	0.03	52
1	0	0	100	5.07	2	65	25.4	113.5	76	202	47	155	13.6	5.4	74.2	2	1	0	1	1	1	1	63.8	0.03	16.5
1	0	0	100	7	2	73	24.5	148	85	216	47	169	13.4	5.2	59.7	1	1	1	0	0	1	1	10.3	0.05	5.88
1	0	0	100	4.63	2	74	33.3	141.5	78.5	194	47	147	13.3	5.5	69.6	1	1	1	0	0	1	1	54.4	0.05	162
1	0	0	100	4.6	2	67	26.6	161	91	200	47	153	12.4	5.6	73.1	2	1	1	0	1	1	1	19	0.08	50.9
1	0	0	100	4.68	2	67	25.1	100.5	62	182	47	135	11.7	5.5	76.9	2	1	1	1	1	1	1	22.6	0.07	16.6
1	0	0	100	6.96	2	65	24.2	157	87	180	47	133	12.7	5.1	82.8	2	1	0	0	1	1	1	18.1	0.06	0.05
1	0	0	100	5.37	2	67	23.6	145.5	84	207	47	160	9.9	8.2	54.0	1	1	1	0	1	0	1	10.3	0.02	81.3
1	0	0	100	5.05	2	72	23.9	180.5	88.5	169	47	122	12.1	5	78.9	2	1	1	0	1	1	1	24.8	0.02	20.6
1	0	0	100	6.85	2	66	22.6	131	79	209	47	162	13.3	5	73.6	2	1	1	0	1	1	1	50.9	0.03	38.3
1	1	0	100	4.58	2	69	30.8	170	92.5	165	47	118	14.1	5.5	75.9	1	1	1	0	1	1	1	314.1	0.45	61.5
1	1	0	100	5.07	2	73	26.8	133.5	78.5	222	47	175	13.3	5.2	73.8	2	1	1	1	0	1	1	8.6	0.26	9.6
1	1	0	100	5.06	2	65	17.8	98	65.5	181	47	134	12.4	5.3	82.8	2	1	1	0	1	1	1	20	0.21	28.5
1	1	0	100	7.2	2	71	24.9	158.5	92.5	189	47	142	12.8	5.5	60.5	2	1	1	0	1	1	1	35.5	0.05	15.7
1	1	0	100	5.37	2	79	29.5	130	75.5	190	47	143	13.1	5.9	75.1	1	1	1	0	1	1	1	9.8	0.06	48.9
1	1	0	100	6.97	2	68	28.2	134.5	83.5	176	47	129	11.8	6.1	76.4	2	1	1	1	1	1	1	18.6	0.04	17.3
1	0	0	100	5.06	2	80	23.9	99.5	50.5	178	47	131	12.3	5.7	34.8	2	1	1	0	1	1	1	118.2	0.22	80.5
1	1	0	100	5.37	2	82	27.5	137.5	75	214	47	167	13.2	5.6	56.0	1	1	1	0	1	1	1	13.3	0.16	53.7
1	1	0	100	6.86	2	80	24.2	128.5	60	232	47	185	12.4	5.1	70.2	1	1	1	1	0	1	1	11.8	0.01	22

1	1	0	100	6.87	2	81	27	136	78.5	238	47	191	12.8	5.5	56.4	1	1	1	0	0	1	1	12.7	0.02	5.84
1	0	1	6.8	6.8	1	69	27.7	115.5	78	203	47	156	15.3	5.6	62.2	2	1	0	0	1	1	1	25.5	0.51	15.6
1	0	1	1.9	1.9	1	70	29.1	174.5	86.5	206	47	159	14.3	5.6	73.6	2	0	1	0	1	1	1	58.8	0.12	56.7
1	1	1	6.4	6.4	1	70	18.7	121.5	67	196	47	149	14.4	5.2	73.6	2	1	0	1	0	1	1	10.8	0.01	18
1	1	1	0.5	0.5	1	70	26.4	142	78.5	211	47	164	15.8	5.6	70.1	2	1	0	0	1	1	1	3.6	0.04	42.4
1	0	1	3.3	3.3	2	78	23	152	86.5	166	47	119	13.2	5.1	57.6	1	1	0	0	1	1	1	50	0.52	510
1	0	1	1.5	1.5	2	76	21.4	164.5	101.5	219	47	172	11.7	4.9	68.6	1	1	1	0	1	1	1	34.7	0.03	34.9
1	0	1	1.3	1.3	2	79	17.8	112	60	231	47	184	11.3	4.8	67.2	1	1	1	1	0	1	1	44.1	0.03	20.4
1	0	1	4.1	4.1	2	70	26.8	158.5	83.5	196	47	149	14.4	6.7	60.9	2	1	1	0	1	0	1	20.8	0.05	11.6
1	1	1	2.4	2.4	2	71	24.7	149.5	90.5	221	47	174	13	5.3	79.4	2	1	1	0	0	1	1	58.6	0.04	11.3
1	0	0	100	7	1	71	21.8	157	79.5	153	48	105	13.8	6.7	77.2	2	1	0	0	1	0	1	109	1.23	4.27
1	0	0	100	6.87	1	72	24	129	88	232	48	184	15.2	5.4	40.5	2	1	0	1	0	1	1	21.5	0.1	3.19
1	0	0	100	6.88	1	72	25.1	132.5	72	206	48	158	15.1	5	72.6	2	1	0	0	1	1	1	17.5	0.1	48.8
1	0	0	100	5.16	1	65	30.9	149	83.5	231	48	183	14.9	5.4	80.5	2	0	0	0	0	1	1	19.4	0.1	29.5
1	0	0	100	0.73	1	74	23.3	113	76.5	175	48	127	15.3	6	71.5	2	0	0	0	1	1	1	28.7	0.25	23.1
1	0	0	100	6.86	1	67	23.8	140.5	80.5	240	48	192	16.2	6.9	63.0	2	0	0	0	0	0	0	39	0.17	139
1	0	0	100	4.64	1	75	24.8	153.5	81.5	182	48	134	15.2	5.8	75.1	1	0	0	0	1	1	0	110.6	7.1	352
1	0	0	100	6.96	1	69	27.1	120	79	216	48	168	14	5.3	74.1	1	0	1	0	1	1	1	112.8	0.2	15.8
1	0	0	100	6.97	1	70	23.4	134.5	77	183	48	135	13.1	5.4	55.0	1	0	1	1	0	1	1	6.6	0.12	25
1	0	0	100	6.84	1	68	23.9	140	86.5	201	48	153	14.4	5.6	71.1	2	1	0	0	1	1	0	26.3	0.18	313
1	0	0	100	4.62	1	65	25.5	150	100	199	48	151	15.8	5.1	76.2	2	1	0	0	1	1	1	21.1	0.15	19
1	0	0	100	6.97	1	72	23.3	146	74	174	48	126	15.8	7.2	69.1	2	1	0	0	1	0	1	66.6	0.41	39.4
1	0	0	100	5.06	1	69	22.9	124.5	79	192	48	144	14.5	5.6	78.3	2	1	0	1	1	1	1	3	0.35	8.17
1	0	0	100	5.07	1	69	23.2	123	78.5	214	48	166	16.2	5.3	62.2	2	1	0	1	1	1	1	5.7	0.47	23.5
1	0	0	100	7.07	1	68	21.7	118	82	202	48	154	15.8	5.1	78.8	2	1	1	1	1	1	1	34.9	0.06	24.8
1	0	0	100	7.08	1	71	25.6	97	58	149	48	101	14.6	5.1	73.1	2	1	1	1	1	1	1	19	0.01	36.9
1	0	0	100	7.08	1	68	25	117.5	73	202	48	154	16	5.1	71.1	2	0	0	1	1	1	1	7.4	0.05	7.92
1	0	0	100	6.87	1	71	23.7	112.5	70	247	48	199	14.8	5.3	69.6	2	1	0	1	0	1	1	3.2	0.07	5.36
1	0	0	100	5.75	1	71	20	113.5	71	121	48	73	13	5.2	73.1	2	0	0	1	1	1	1	2.7	0.07	67.2
1	0	0	100	5.16	1	72	21.4	98.5	66.5	129	48	81	16.6	5.2	54.2	1	0	0	0	1	1	0	21.2	0.09	202
1	0	0	100	7.2	1	66	23.4	116.5	79	164	48	116	16	5.4	75.7	2	0	0	1	1	1	1	7.4	0.05	30.1
1	0	0	100	7.01	1	65	20.4	104	64	129	48	81	12.6	5.9	80.5	2	0	0	0	1	1	1	4.3	0.05	46.4
1	0	0	100	7.01	1	68	26.1	116.5	68.5	163	48	115	14.4	5.1	78.8	2	1	1	1	1	1	1	14.4	0.04	4.23
1	0	0	100	7.01	1	68	27.9	143	72.5	177	48	129	14.2	6.8	74.6	2	1	0	0	1	0	1	8.8	0.04	26.8
1	0	0	100	7.01	1	71	25.2	159.5	86.5	193	48	145	13.6	5.5	73.1	2	1	0	0	1	1	0	22.8	0.05	48.7
1	0	0	100	6.98	1	71	22.8	150	84.5	226	48	178	14.6	5.6	77.2	2	1	0	0	0	1	1	17.8	0.05	0.05
1	0	0	100	4.62	1	68	26.6	117	64.5	220	48	172	15.6	6.5	78.8	2	1	1	1	0	0	1	20.2	0.05	5.4
1	0	0	100	5.05	1	69	24.8	128.5	84.5	213	48	165	13.3	5.6	70.6	2	1	1	0	1	1	1	22.7	0.07	25.2
1	0	0	100	4.64	1	72	25.4	112	66	170	48	122	16	5.7	69.1	2	0	0	1	1	1	1	15.6	0.04	2.71
1	0	0	100	4.64	1	73	22.1	141	84	165	48	117	12.5	5.7	76.1	2	1	1	0	1	1	1	13.5	0.01	28.1
1	0	0	100	6.87	1	69	22.1	157.5	81.5	126	48	78	12.6	5.4	78.3	2	0	0	0	1	1	1	5.7	0.03	40.4
1	0	0	100	6.87	1	67	28.6	116.5	87	213	48	165	18.5	5.6	71.6	2	0	0	1	1	1	0	56.4	0.07	16.1
1	0	0	100	6.98	1	69	23.8	143.5	81	177	48	129	16.4	4.6	74.1	2	1	0	0	1	1	1	3	0.02	19.3
1	0	0	100	6.8	1	76	24.1	139	71.5	186	48	138	14.7	5.9	67.2	2	0	0	0	1	1	1	20.4	0.05	22.1
1	0	0	100	6.83	1	65	22.3	111	75	183	48	135	13.4	4.8	80.5	2	1	0	1	1	1	1	92.9	0.02	35.8
1	0	0	100	6.84	1	71	24.3	130.5	64.5	162	48	114	14.3	5.1	37.5	2	1	0	1	1	1	1	6.3	0.02	51.1
1	0	0	100	4.61	1	73	25.6	106.5	63.5	179	48	131	15.1	4.9	68.6	2	1	1	1	1	1	0	70.8	0.05	88
1	0	0	100	4.62	1	71	26.2	182	103.5	220	48	172	13.8	7.4	69.6	2	1	1	0	0	0	1	1019.7	0.08	12
1	0	0	100	4.62	1	65	23.6	141.5	91.5	176	48	128	15.5	5.5	76.2	2	1	0	0	1	1	1	14.1	0.04	26
1	0	0	100	4.69	1	65	23.5	133.5	71	209	48	161	15.1	6.6	80.5	2	0	1	1	1	0	1	699.6	0.09	1.07
1	0	0	100	4.7	1	69	19.2	143.5	89	173	48	125	16.1	4.7	74.1	2	1	1	0	1	1	1	6.9	0.02	27.4

1	0	0	100	6.87	1	76	26.2	107	57.5	245	48	197	14	5.5	70.5	2	1	1	1	0	1	1	8.8	0.05	16.8
1	0	0	100	6.87	1	71	22.8	130	81.5	196	48	148	15.3	4.7	77.2	2	1	0	0	1	1	1	7.2	0.06	10.9
1	0	0	100	6.87	1	66	22.8	122	70.5	144	48	96	11.7	5.1	79.9	2	1	1	1	1	1	1	52	0.03	38.6
1	0	0	100	5.04	1	65	20.4	134	82	157	48	109	14.6	7.3	85.8	2	0	1	1	1	0	1	65.1	0.04	0.39
1	1	0	100	5.1	1	69	20.7	100.5	64	157	48	109	16	8.1	78.3	2	0	1	1	1	0	1	186.5	0.06	46.6
1	1	0	100	6.23	1	72	21.1	114.5	65.5	186	48	138	14.3	5.8	76.7	1	0	0	0	1	1	1	13.3	0.06	31.1
1	1	0	100	6.99	1	73	27.3	110	75.5	146	48	98	13.1	4.5	72.0	2	1	0	0	1	1	1	41.4	0.06	114
1	0	0	100	4.58	2	77	23.6	165	82	188	48	140	13.2	5.3	71.7	2	1	1	0	1	1	1	28.9	0.1	17.1
1	0	0	100	6.86	2	67	30.2	154	85	244	48	196	13.6	6.1	73.1	2	1	1	0	0	1	1	35.7	0.12	95.6
1	0	0	100	7.23	2	72	25.7	146.5	76.5	226	48	178	13.5	7.2	60.1	2	1	1	0	0	0	1	16.2	0.22	41.4
1	0	0	100	5.16	2	67	25.2	121	69.5	161	48	113	13.4	6.1	73.1	2	0	1	1	1	0	1	6.9	0.23	24.5
1	0	0	100	6.84	2	66	25.9	171	92.5	266	48	218	12.1	5.7	54.3	2	1	1	0	0	1	1	40	0.35	46.3
1	0	0	100	5.06	2	69	27.7	117.5	64.5	237	48	189	13.8	5.7	80.5	2	1	0	0	0	1	1	16.6	0.18	37.9
1	0	0	100	5.07	2	65	25.2	141.5	78	163	48	115	13.6	5.1	82.8	1	1	1	0	1	1	1	4	0.16	40.4
1	0	0	100	4.63	2	67	22.4	125	72.5	200	48	152	13.4	5	76.9	2	1	1	0	1	1	1	25.5	0.56	18
1	0	0	100	4.64	2	70	26.6	121	76.5	195	48	147	14	5.4	60.9	1	1	1	0	1	1	1	277.9	0.11	53.6
1	0	0	100	4.59	2	65	22.3	115.5	70.5	167	48	119	12.6	5.3	74.2	2	1	1	1	1	1	1	13.2	0.1	35
1	0	0	100	4.6	2	67	25.6	152	79	226	48	178	12.7	5.5	76.9	2	1	1	0	0	1	1	36.9	0.1	19.5
1	0	0	100	5.04	2	69	31.4	135	77	201	48	153	13	5.8	53.2	1	1	1	0	1	1	1	35.3	0.1	24.9
1	0	0	100	7.08	2	73	26.2	144.5	81.5	187	48	139	12.7	5.5	70.1	2	1	1	0	1	1	1	20	0.05	30.7
1	0	0	100	4.58	2	75	24.2	155	88.5	204	48	156	14	6.2	77.2	2	1	1	0	1	1	1	65.3	0.02	15.9
1	0	0	100	4.59	2	66	22.6	114	72	210	48	162	12.3	5.1	73.6	2	1	1	1	1	1	1	26	0.01	46.9
1	0	0	100	7.21	2	70	27.1	150.5	80.5	178	48	130	13	5.1	80.0	2	1	0	0	1	1	1	43.6	0.05	27.5
1	0	0	100	6.86	2	79	23.7	118	62	215	48	167	10.7	5.7	67.2	1	1	1	0	1	1	1	17.3	0.05	18.7
1	0	0	100	6.87	2	66	28.4	134.5	85.5	225	48	177	15	6.2	77.5	2	1	1	1	0	1	1	5.9	0.05	10.8
1	0	0	100	6.85	2	66	27.8	134.5	75	221	48	173	14	6.1	77.5	2	1	0	1	0	1	1	10.3	0.07	16.3
1	0	0	100	7	2	72	31.4	124	81.5	191	48	143	12.8	5.2	74.3	1	1	1	0	1	1	1	8.5	0.04	34.4
1	0	0	100	7.03	2	70	28.9	197.5	109	206	48	158	13.1	5.4	80.0	1	1	1	0	1	1	1	170.2	0.06	2.65
1	0	0	100	7.04	2	67	19.8	110.5	72.5	161	48	113	12.4	5.4	81.7	2	1	1	1	1	1	1	28.4	0.01	4.71
1	0	0	100	7.04	2	69	30.1	125.5	77	190	48	142	14.6	5.3	80.5	2	1	0	1	1	1	1	17.6	0.01	62.2
1	0	0	100	7.04	2	68	27.1	127	70	203	48	155	13.7	5.2	72.6	1	1	1	1	1	1	1	4.2	0.03	30.2
1	0	0	100	5.07	2	69	28.8	124.5	80	175	48	127	12.4	5.2	61.4	2	1	1	1	1	1	1	25.9	0.06	27.4
1	0	0	100	6.98	2	72	25.5	137.5	84.5	215	48	167	12.8	5.8	78.9	1	1	1	0	1	1	1	21.4	0.03	10.7
1	0	0	100	4.64	2	67	25.7	118.5	61	188	48	140	14.6	5.1	76.9	2	1	1	0	1	1	1	24.7	0.05	30.4
1	0	0	100	6.97	2	67	25.3	126	69.5	198	48	150	13	5.3	76.9	2	1	0	1	1	1	1	16.9	0.03	20.1
1	0	0	100	6.79	2	73	23.6	136.5	76	241	48	193	15.1	6.3	70.1	2	1	1	1	0	1	1	15.2	0.03	34.8
1	0	0	100	6.82	2	69	20.3	104	70.5	189	48	141	12.2	5.3	75.9	1	1	1	1	1	1	1	26.5	0.01	7.27
1	0	0	100	4.62	2	68	24.2	126	68.5	213	48	165	12.4	5.2	61.8	2	1	1	0	0	1	1	7.7	0.08	26.1
1	0	0	100	5.05	2	75	22.2	153	86	155	48	107	12.3	5.9	72.7	2	1	0	0	1	1	1	208.9	0.08	60.5
1	0	0	100	4.67	2	77	20.9	144.5	67.5	210	48	162	12.9	6.1	71.7	1	1	1	0	1	1	1	65.7	0.02	87.5
1	0	0	100	4.69	2	72	25.7	109	73.5	237	48	189	14.6	5.9	70.6	1	1	1	1	0	1	1	35.1	0.03	6.43
1	0	0	100	5.36	2	68	29.4	143	80.5	199	48	151	13.4	5.2	76.4	2	1	1	0	1	1	1	12.4	0.02	23
1	0	0	100	5.37	2	71	28.8	217.5	107.5	238	48	190	13.9	5.9	71.1	2	1	0	0	0	1	1	186.5	0.09	40.6
1	0	0	100	5.06	2	66	27.3	140	78	197	48	149	13.9	10.6	77.5	2	1	1	0	1	0	1	130.2	0.06	21.9
1	0	0	100	5.06	2	68	22.8	109	64.5	213	48	165	13.4	5.2	61.8	2	1	0	1	1	1	1	6.5	0.05	39.7
1	0	0	100	5.04	2	67	26.4	142	92	223	48	175	13	5.7	73.1	2	1	0	0	0	1	1	14.9	0.04	31.4
1	1	0	100	7.05	2	70	23.7	136	83	158	48	110	12.5	5.1	75.3	1	1	1	1	1	1	1	25.6	0.01	31.1
1	1	0	100	6.87	2	79	20.8	138	67	173	48	125	11.3	5.6	57.2	2	1	1	1	1	1	1	18.5	0.03	56
1	1	0	100	4.61	2	77	21.7	131.5	71.5	216	48	168	12.7	5.7	58.0	1	1	1	0	1	1	1	33.8	0.02	41.6
1	0	0	100	5.07	1	80	24	133.5	72.5	152	48	104	12.9	5.2	72.5	2	1	1	1	1	1	1	15	0.05	31.4
1	0	0	100	7	1	80	20.3	145	86	175	48	127	13.7	5.3	57.5	2	1	1	0	1	1	1	18.9	0.01	89.2

1	0	0	100	4.59	1	80	21.4	87.5	53.5	170	48	122	14.5	5.5	51.3	2	1	1	0	1	1	1	18.2	0.02	43.4
1	0	0	100	7	2	83	24.9	164.5	82	165	48	117	12.8	5.2	73.0	1	1	1	0	1	1	1	27	0.06	25.1
1	0	0	100	4.62	2	81	22.3	135.5	67.5	250	48	202	11.7	5.2	69.7	2	1	1	1	0	1	1	29.4	0.04	47.3
1	0	0	100	6.96	2	81	26.7	136.5	71	248	48	200	13.1	4.9	69.7	1	1	1	0	0	1	1	422.4	0.07	25.9
1	0	0	100	5.16	1	73	28.6	121	65.5	171	48	123	14.5	7.7	29.7	1	1	0	0	1	0	1	19.2	0.36	16.6
1	0	0	100	4.63	1	70	24.2	158.5	94.5	205	48	157	14.2	8.2	73.6	2	1	1	0	1	0	1	6.6	0.18	53.6
1	0	0	100	6.8	1	67	22.8	117	87	239	48	191	15.1	5.2	71.6	2	1	1	1	0	1	1	322.8	0.11	0.05
1	0	0	100	2.31	1	79	21.7	132	73	234	48	186	13.2	5.1	38.6	2	1	1	1	0	1	1	4.8	0.19	0.14
1	0	0	100	5.37	1	67	32	121.5	86	182	48	134	15.2	6.1	71.6	2	1	0	0	1	1	1	7.3	0.11	7.62
1	0	0	100	6.87	1	67	21.8	137	73.5	176	48	128	9.7	5.4	84.6	2	1	1	1	1	1	1	11.4	0.2	53.1
1	0	0	100	5.16	1	69	27.1	178.5	94.5	157	48	109	14.4	5.3	78.3	1	1	1	0	1	1	1	62.1	0.09	15.3
1	0	0	100	7	1	77	25.2	160.5	82.5	174	48	126	12.9	5.7	52.4	1	1	1	0	1	1	1	87.1	0.02	77.1
1	0	0	100	5.37	1	65	26.5	123	83	168	48	120	14.7	5.1	85.8	2	1	0	1	1	1	1	11.3	0.02	30.4
1	1	0	100	4.6	1	76	22.1	136	81	162	48	114	13.8	5.2	59.2	2	1	0	1	1	1	1	31.9	0.03	38.6
1	0	0	100	6.86	2	67	32	155	86	232	48	184	14.2	5.3	76.9	2	1	1	0	0	1	1	13.9	0.11	11
1	0	0	100	6.8	2	70	29.6	192.5	104	212	48	164	13.2	5.8	71.6	2	1	1	0	1	1	1	34.7	0.44	61.1
1	0	0	100	4.61	2	70	34.1	144.5	85.5	212	48	164	13.4	5.8	60.9	1	1	1	0	1	1	1	60.4	0.15	39.9
1	0	0	100	4.62	2	75	24.8	150	79.5	178	48	130	14.9	5.9	72.7	2	1	1	0	1	1	1	25.1	0.16	38.2
1	0	0	100	6.79	2	70	22	131.5	86	186	48	138	12.9	5.6	75.3	2	1	1	1	1	1	1	10.7	0.39	26.6
1	0	0	100	4.69	2	71	24.4	120	68.5	195	48	147	13	5	71.1	1	1	1	1	1	1	1	25.3	0.11	2.3
1	0	0	100	6.86	2	77	27.2	118.5	65	243	48	195	14.4	5.4	71.7	1	1	1	0	0	1	1	23.3	0.21	50
1	0	0	100	6.87	2	76	29.6	133.5	70	211	48	163	13.2	5.3	72.2	2	1	1	1	0	1	1	83.2	0.12	6.38
1	0	0	100	7.08	2	70	28.6	131.5	77.5	212	48	164	12.4	5.8	75.3	1	1	1	0	1	1	1	161.6	0.04	20.1
1	0	0	100	6.86	2	69	26.7	150	83	193	48	145	11.8	5	80.5	2	1	1	0	1	1	1	16.3	0.06	58.5
1	0	0	100	6.87	2	67	22.7	127	65	192	48	144	10.3	6	54.0	2	1	1	1	0	1	1	4.8	0.02	15
1	0	0	100	6.88	2	75	26.8	147	83.5	206	48	158	10.8	5.4	72.7	1	1	1	0	1	1	1	44.2	0.07	117
1	0	0	100	5.15	2	70	23.1	115.5	65.5	207	48	159	13.2	5.4	71.6	2	1	1	0	1	1	1	43.8	0.02	36.3
1	0	0	100	6.84	2	66	28.1	118	73.5	216	48	168	13.5	6.5	77.5	2	1	1	0	1	0	1	12.6	0.04	55
1	0	0	100	7	2	69	20.5	133	75	119	48	71	10.8	3.4	86.7	1	1	1	1	1	1	1	43.7	0.01	68.7
1	0	0	100	7.01	2	76	20.6	120	66	234	48	186	12.2	6.4	72.2	2	1	0	1	0	1	1	9.2	0.01	16.2
1	0	0	100	7.02	2	77	23.6	136.5	79	204	48	156	12	5.7	81.9	1	1	0	1	1	1	1	41.2	0.01	11
1	0	0	100	5.06	2	67	28.2	98.5	57	220	48	172	13.6	5.7	76.9	2	1	1	1	0	1	1	32.9	0.02	10.8
1	0	0	100	5.06	2	70	22.6	126.5	65.5	175	48	127	12.4	6.9	80.0	2	1	1	1	1	0	1	11.9	0.06	42.7
1	0	0	100	7	2	75	22.7	148	81.5	176	48	128	12.8	5.8	69.1	1	1	1	0	1	1	1	3.6	0.01	10.2
1	0	0	100	2.09	2	65	41.6	131.5	72.5	214	48	166	13.1	5.7	74.2	2	1	1	0	1	1	1	18.2	0.05	17.7
1	0	0	100	6.97	2	73	21.1	169.5	90.5	162	48	114	12.6	5.5	78.3	1	1	1	0	1	1	1	50.4	0.04	20.4
1	0	0	100	6.98	2	73	30.7	149	83	198	48	150	14.9	5.6	73.8	2	1	1	0	1	1	1	189.3	0.04	0.05
1	0	0	100	6.8	2	76	26.6	142	91	242	48	194	14.7	5.5	58.4	2	1	1	0	0	1	1	21.8	0.07	21.8
1	0	0	100	4.61	2	68	30.2	117	76	251	48	203	13.2	6	72.6	2	1	1	0	0	1	1	11.9	0.05	19
1	0	0	100	4.61	2	65	31.8	158	86	164	48	116	14	5.1	74.2	2	1	1	0	1	1	1	17.3	0.04	13.9
1	0	0	100	4.61	2	72	23.8	88.5	54	233	48	185	13.1	5.6	70.6	2	1	1	1	0	1	1	50.2	0.06	105
1	0	0	100	6.97	2	65	25	114	70.5	197	48	149	12	5.3	78.0	2	1	1	1	1	1	1	19.4	0.02	13.1
1	0	0	100	5.37	2	70	19.3	125.5	72	170	48	122	11.3	4.7	71.6	1	1	1	1	1	1	1	22.4	0.01	42.2
1	0	0	100	5.37	2	72	25.1	117	67.5	217	48	169	12.2	5.3	78.9	1	1	1	1	1	1	1	14	0.01	16.8
1	0	0	100	5.06	2	77	22.3	115	69	224	48	176	13.8	5.8	68.2	2	1	1	1	0	1	1	10.2	0.05	39.5
1	1	0	100	7.03	2	75	26.7	122	77	202	48	154	13.8	4.9	72.7	2	1	1	0	1	1	1	30.2	0.07	20.7
1	1	0	100	6.44	2	78	27.4	103	75	229	48	181	13.5	5.4	57.6	2	1	1	0	0	1	1	55.7	0.03	34.8
1	1	0	100	6.87	2	71	28.7	143	90	154	48	106	13.9	5.2	74.8	2	1	1	0	1	1	1	25	0.06	9.06
1	0	0	100	5.08	1	80	27	149	90.5	276	48	228	13.6	5.7	68.6	2	1	1	0	0	1	1	9.4	0.02	64.3
1	1	0	100	6.87	2	81	25.9	146.5	78.5	192	48	144	13.9	7.1	66.3	2	1	1	0	1	0	1	41.6	0.03	25.3
1	0	1	4.4	4.4	1	65	25	146	94	168	48	120	13.9	6	57.0	2	1	1	0	1	1	1	117.2	0.04	28.9

1	0	1	2.3	2.3	1	67	29.4	124.5	66.5	166	48	118	14	5.7	75.2	2	1	1	1	1	1	1	81	0.02	35.1
1	1	1	4.2	4.2	1	66	19.7	111	69.5	143	48	95	12.1	5.6	72.1	2	1	0	0	1	0	1	5.5	0.1	43.8
1	0	1	2.8	2.8	2	77	16.1	187	101.5	212	48	164	13.6	5.9	50.3	1	1	1	0	1	1	1	17.7	0.02	150
1	1	1	1.4	1.4	2	78	33.6	128	82.5	230	48	182	12.7	6.6	57.6	2	1	1	0	0	0	1	44.4	0.17	24.3
1	1	1	4.1	4.1	2	65	33.8	159	102.5	169	48	121	13.6	5.7	74.2	2	1	1	0	1	1	1	17.9	0.4	58.6
1	0	1	6.6	6.6	1	73	26.8	123	80.5	170	48	122	16.1	5.2	72.0	2	1	1	0	1	1	1	32.1	0.04	149
1	0	1	5.5	5.5	2	77	26.4	156.5	81	173	48	125	14	4.7	68.2	1	1	0	0	1	1	1	29.5	0.42	34.9
1	0	1	3.8	3.8	2	72	26.3	110.5	65	170	48	122	14.8	7.4	74.3	2	1	1	0	0	0	1	22.8	0.01	148
1	0	1	1.9	1.9	2	65	19.2	109	63.5	146	48	98	13.1	7.3	48.2	2	1	1	1	1	0	0	27.6	0.09	82.8
1	1	1	0.9	0.9	2	73	20.4	154	82	184	48	136	11.6	5.1	70.1	2	1	1	0	1	1	1	51.7	0.01	37.9
1	1	1	2.8	2.8	2	69	18.3	92	54	158	48	110	11.6	5.5	72.1	1	1	1	1	1	1	1	4	0.01	0.05
1	1	1	0.4	0.4	2	78	30.4	157.5	83	213	48	165	12.2	5.9	71.2	1	1	1	0	1	1	1	8.2	0.07	25.7
1	0	0	100	6.88	1	68	25.4	131	81.5	224	49	175	14.8	5.2	41.7	2	1	0	0	0	1	1	97.1	0.13	12.9
1	0	0	100	6.86	1	75	25.3	124	65.5	148	49	99	14.9	5.2	75.1	2	1	0	1	1	1	1	9.3	0.45	30.3
1	0	0	100	7.01	1	70	19.5	107.5	62	207	49	158	13.3	5.9	82.8	2	0	1	1	1	1	1	14.5	0.16	7.22
1	0	0	100	7.04	1	70	30.1	120.5	70	153	49	104	13.6	5.2	77.7	2	1	1	0	1	1	0	11	0.13	1.06
1	0	0	100	4.19	1	74	31.3	128	65	225	49	176	12.2	7.1	60.0	2	1	1	0	0	0	1	113.3	0.12	15.3
1	0	0	100	4.64	1	68	27.8	155.5	83.5	249	49	200	14.3	5.5	71.1	2	1	1	0	0	1	1	15.1	0.13	11
1	0	0	100	4.64	1	72	26.9	138.5	76.5	213	49	164	12.3	6.9	69.1	2	1	1	1	1	0	1	19.4	0.13	8.4
1	0	0	100	4.64	1	71	27.9	125	88.5	213	49	164	15.6	5.4	61.3	2	1	1	0	1	1	1	7.4	1.19	15.1
1	0	0	100	6.79	1	72	28.8	141.5	83	205	49	156	14.7	6.5	69.1	2	1	0	0	1	0	1	19.4	0.17	17.6
1	0	0	100	6.8	1	72	23.4	123.5	73	194	49	145	15.1	6.9	76.7	2	0	0	0	1	0	1	36.1	0.14	23.2
1	0	0	100	4.62	1	75	22.6	153	96	221	49	172	18	5.5	67.7	2	1	0	0	0	1	1	146	0.36	21.7
1	0	0	100	4.67	1	73	23.8	117	75	207	49	158	13.7	5	68.6	2	1	1	0	1	1	1	74.9	0.59	8.44
1	0	0	100	4.69	1	67	20.3	116	75.5	147	49	98	13.4	5.3	84.6	2	1	1	0	1	1	1	23	0.21	11
1	0	0	100	6.87	1	70	27.9	127.5	80.5	180	49	131	14.6	5.1	77.7	2	0	0	1	1	1	1	11	0.1	8.97
1	0	0	100	5.04	1	65	27.5	173.5	103.5	192	49	143	15.5	5.8	76.2	2	0	0	0	1	1	1	253	0.17	9.32
1	0	0	100	7	1	65	28.5	124	77.5	247	49	198	15.3	5.7	76.2	2	1	0	1	0	1	1	80.4	0.08	9.39
1	0	0	100	7.07	1	73	26.5	130	80.5	222	49	173	15.2	5.6	72.0	2	1	1	1	0	0	1	64.1	0.06	29.8
1	0	0	100	7.08	1	71	23.4	131	76	203	49	154	13.5	5.3	61.3	2	1	0	1	1	1	1	14.7	0.04	17.3
1	0	0	100	7.08	1	75	25.3	123	77.5	284	49	235	15.2	5.5	71.0	2	0	1	1	0	1	1	8.6	0.07	5.63
1	0	0	100	4.58	1	69	21.2	114.5	73.5	177	49	128	12.6	5.1	83.4	2	1	1	1	1	1	1	17.6	0.04	43.1
1	0	0	100	5.16	1	67	26.7	132	75	181	49	132	14	5.8	75.2	2	1	0	1	1	1	1	2.8	0.01	20.1
1	0	0	100	5.16	1	72	23	146	71.5	163	49	114	13.6	5.1	76.7	2	1	0	0	1	1	1	112.4	0.06	64.8
1	0	0	100	6.84	1	68	27.7	109	62	185	49	136	13.4	6.4	71.1	2	1	1	1	1	1	1	29.8	0.04	13.2
1	0	0	100	6.85	1	73	25.2	143.5	85	194	49	145	13.4	5.5	48.5	2	1	1	0	1	1	1	368.2	0.08	15.8
1	0	0	100	6.86	1	65	25	118	63	174	49	125	15.2	5.9	80.5	2	0	1	1	1	1	1	6.3	0.07	29.3
1	0	0	100	6.86	1	70	23.6	118	78	174	49	125	14.2	5.1	61.7	2	0	0	0	1	1	1	6.8	0.01	13.3
1	0	0	100	7	1	75	21.9	127	75	142	49	93	13.8	5.1	59.6	1	1	1	0	1	1	1	11.3	0.03	16.1
1	0	0	100	2.39	1	65	22.5	154.5	85.5	129	49	80	15.3	5.9	85.8	2	1	0	0	1	1	1	12.7	0.01	38.3
1	0	0	100	7.03	1	74	19.4	132	81.5	215	49	166	13.7	5.1	75.6	1	1	1	1	1	1	1	7.5	0.02	18.4
1	0	0	100	4.63	1	75	26	130.5	73	192	49	143	13.6	5.9	75.1	2	1	0	0	1	1	1	14.4	0.03	22.6
1	0	0	100	4.63	1	68	28.2	107.5	63.5	192	49	143	15.4	5.5	50.2	2	1	0	1	1	1	1	16.4	0.05	41.5
1	0	0	100	4.63	1	67	24.7	108	70	189	49	140	14.7	5.3	75.2	2	1	0	1	0	1	1	10.9	0.01	6.69
1	0	0	100	4.64	1	67	21.8	120.5	75	232	49	183	15.5	5.4	79.4	2	0	0	1	0	1	1	11.9	0.02	8.91
1	0	0	100	6.96	1	74	21.2	157	79	194	49	145	12.9	5.1	68.2	1	1	0	0	1	1	1	8.5	0.07	34.6
1	0	0	100	6.96	1	65	28.9	146.5	96	223	49	174	16.1	6.1	80.5	1	1	0	0	0	1	1	10.2	0.07	5.18
1	0	0	100	6.98	1	67	19.9	111.5	69	177	49	128	12.9	5.2	79.4	2	1	0	0	1	1	1	8.5	0.03	27.1
1	0	0	100	3.76	1	73	22.9	120	78.5	207	49	158	14.4	5.2	68.6	2	1	1	1	1	1	1	4.1	0.04	61.9
1	0	0	100	4.62	1	65	22.6	103.5	68.5	159	49	110	14.7	5.1	76.2	2	1	1	1	1	1	1	4.8	0.06	6.28
1	0	0	100	4.62	1	75	25.2	159	81.5	160	49	111	15.1	4.9	21.8	2	1	0	0	1	1	1	1482.5	0.06	14.4

1	0	0	100	6.44	1	65	22.5	141.5	96	240	49	191	14.9	5.8	57.0	2	0	1	0	0	1	1	98.8	0.05	2.9
1	0	0	100	4.68	1	76	21.6	146	85	197	49	148	15.6	5.2	70.5	1	1	0	0	1	1	1	26.6	0.01	18.2
1	0	0	100	5.37	1	69	22.4	120.5	70.5	179	49	130	14.2	5.1	74.1	2	1	0	1	1	1	1	4.3	0.02	41.5
1	0	0	100	5.37	1	68	23.7	132.5	73.5	167	49	118	13.5	5.6	78.8	2	1	0	0	1	0	1	3.8	0.03	6.53
1	0	0	100	5.05	1	77	24.8	131	65.5	203	49	154	15	5.1	74.0	2	1	0	0	1	1	1	5.3	0.05	39.9
1	0	0	100	5.06	1	74	27.3	106	56.5	173	49	124	13.8	5.7	53.5	2	1	0	0	1	1	1	12.2	0.03	35.9
1	0	0	100	6.85	1	74	25.3	148.5	90.5	196	49	147	14.3	5.5	53.5	2	1	1	0	1	1	1	19.6	0.03	10.4
1	0	0	100	6.87	1	69	22.6	138	80.5	203	49	154	16.2	5.1	55.4	2	0	0	1	1	1	1	52.9	0.01	266
1	0	0	100	5.04	1	74	24	126.5	71.5	127	49	78	14.2	5.2	75.6	2	1	1	1	1	1	1	81.6	0.04	53.1
1	1	0	100	6.86	1	77	21.2	113.5	62	136	49	87	10.4	4.7	66.7	1	1	1	1	1	1	1	10.5	0.01	20.5
1	0	0	100	6.86	2	77	24.7	155.5	81.5	174	49	125	14.5	5.2	58.0	2	1	1	0	1	1	1	24.9	0.15	49.7
1	0	0	100	5.06	2	66	26	131	81.5	214	49	165	12.9	5.2	62.7	2	1	0	1	1	1	1	21.7	0.18	35.4
1	0	0	100	5.08	2	66	22.2	106	65	169	49	120	12.7	5.5	62.7	2	1	1	1	1	1	1	9.7	0.11	33.2
1	0	0	100	6.87	2	65	38.1	159	81	185	49	136	13.9	6.7	74.2	2	1	1	0	0	0	0	44.9	0.34	156
1	0	0	100	6.95	2	70	23	137.5	72	219	49	170	13.4	5.1	80.0	2	1	1	0	1	1	1	16.5	0.13	23.2
1	0	0	100	6.44	2	68	24.7	109	64	136	49	87	12.3	5.3	87.3	2	1	0	1	1	1	1	18.7	0.13	1.93
1	0	0	100	5.37	2	68	34.5	151	87.5	187	49	138	13.4	6	81.1	2	1	1	0	1	1	1	114.8	0.18	10.2
1	0	0	100	5.05	2	74	20.1	140	71.5	138	49	89	14.3	6	69.6	2	1	1	0	1	1	1	26.8	0.2	15.3
1	0	0	100	6.87	2	66	28.7	144	81	226	49	177	13.6	5.8	73.6	1	1	1	0	0	1	1	22.7	0.12	4.92
1	0	0	100	4.58	2	71	23.8	118	68	265	49	216	13.6	6.3	79.4	1	1	1	1	0	0	1	52.9	0.05	37.1
1	0	0	100	6.87	2	76	21.1	93	55.5	168	49	119	12	5	58.4	1	1	1	1	1	1	1	18.5	0.03	14.6
1	0	0	100	6.87	2	65	21.1	111	64.5	177	49	128	13	5.8	78.0	2	1	1	1	1	1	1	4.4	0.03	2.75
1	0	0	100	6.87	2	66	25.8	143.5	77	222	49	173	12.7	5.5	62.7	2	1	1	0	0	1	1	10.2	0.06	21.8
1	0	0	100	6.87	2	74	27.1	149.5	78.5	211	49	162	12	5.5	77.8	1	1	1	0	1	1	1	119.6	0.08	23.8
1	0	0	100	6.84	2	65	22.7	105	69.5	182	49	133	12.6	5.5	78.0	2	1	1	1	1	1	1	23.2	0.02	11.9
1	0	0	100	6.84	2	70	24.8	132	88.5	223	49	174	12.4	5.7	75.3	1	1	1	1	0	1	1	12.8	0.02	17.2
1	0	0	100	6.84	2	66	24.1	146	74.5	239	49	190	13.7	6	73.6	2	1	1	0	0	1	1	4.4	0.02	18.4
1	0	0	100	6.85	2	68	33.1	154.5	84	250	49	201	13.1	5.2	76.4	2	1	1	0	0	1	1	19	0.09	108
1	0	0	100	6.86	2	69	22.6	138.5	77.5	209	49	160	12	5.7	75.9	2	1	1	1	1	1	1	13.1	0.03	23.7
1	0	0	100	6.86	2	71	23	122	70.5	235	49	186	14	5.6	71.1	2	1	1	1	0	1	1	10.1	0.04	4.14
1	0	0	100	7.01	2	65	24.3	137	79	201	49	152	13.9	5.2	82.8	2	1	1	1	1	1	1	9.8	0.06	12.5
1	0	0	100	7.03	2	66	27.1	151.5	85.5	190	49	141	12.6	5.3	82.3	2	1	1	0	1	1	1	12.4	0.01	22
1	0	0	100	7.04	2	66	20.1	156.5	80.5	222	49	173	12	5.9	77.5	1	1	1	0	0	1	1	9.3	0.01	14.4
1	0	0	100	5.05	2	68	25.1	103	61	174	49	125	11.2	5.5	34.4	2	1	1	0	0	1	1	858.5	0.02	19.4
1	0	0	100	4.62	2	76	19.2	99	51.5	158	49	109	10	5.2	68.6	1	1	1	1	1	1	1	55.4	0.07	49.9
1	0	0	100	4.64	2	67	18.6	148.5	67	206	49	157	9.9	6.5	62.2	2	1	1	0	1	0	1	15.7	0.03	91.8
1	0	0	100	6.87	2	66	20	122.5	76.5	151	49	102	13	6.2	77.5	2	1	1	1	1	1	1	24.1	0.08	11.6
1	0	0	100	6.96	2	66	28.8	153.5	83.5	204	49	155	13.4	5.6	77.5	2	1	1	0	1	1	1	49	0.05	16.4
1	0	0	100	6.97	2	70	23.5	148.5	85.5	192	49	143	14.7	5.1	75.3	1	1	0	0	1	1	1	78.1	0.06	49.9
1	0	0	100	6.8	2	66	28.8	112	65.5	156	49	107	13.7	5.2	73.6	2	1	0	1	1	1	1	5.3	0.09	58.7
1	0	0	100	6.83	2	69	20.6	107	65.5	258	49	209	13	5.3	72.1	1	1	1	0	0	1	1	10.8	0.01	17.7
1	0	0	100	4.59	2	67	28	153	71.5	169	49	120	13.1	6	76.9	2	1	1	0	1	1	1	35.8	0.03	42.2
1	0	0	100	4.59	2	74	23.2	156	77	192	49	143	11.1	5.4	36.3	2	1	0	0	1	1	1	32.7	0.02	66.4
1	0	0	100	4.6	2	67	26.3	123.5	75	230	49	181	13.8	5.8	73.1	2	1	1	0	0	1	1	20.3	0.07	14.4
1	0	0	100	4.62	2	70	27.3	125	73.5	227	49	178	13.6	5.4	71.6	2	1	1	1	0	1	1	5.3	0.06	9.37
1	0	0	100	4.7	2	70	21.7	110.5	62	168	49	119	12.5	5.3	80.0	1	1	1	1	1	1	1	486.5	0.02	51.7
1	0	0	100	5.36	2	68	25.8	108.5	61	196	49	147	12.8	5.6	76.4	2	1	1	1	1	1	1	10.3	0.01	46.3
1	0	0	100	5.37	2	68	26.5	143	75	253	49	204	14	5.9	76.4	2	1	1	0	0	1	1	18.6	0.05	13.2
1	0	0	100	5.06	2	66	22.7	126	78	210	49	161	13.9	5.7	73.6	2	1	1	0	1	1	1	18.9	0.04	12.2
1	0	0	100	6.86	2	65	26.1	136	99	234	49	185	13.8	4.7	78.0	2	1	1	0	0	1	1	61.7	0.03	25.8
1	0	0	100	6.87	2	67	21.9	96.5	57.5	187	49	138	13.2	5.3	76.9	2	1	1	1	1	1	1	24	0.04	33.4

1	0	0	100	6.87	2	77	23.8	182.5	112.5	245	49	196	12.6	5.1	58.0	2	1	1	0	0	1	1	96.3	0.04	95.4
1	0	0	100	5.04	2	66	31.5	123.5	73	174	49	125	14.1	6.2	77.5	2	1	1	0	1	1	1	21.3	0.08	2.82
1	0	0	100	5.04	2	70	19.7	112.5	70.5	166	49	117	11.5	5.3	75.3	1	1	1	1	1	1	1	10.1	0.07	13.8
1	1	0	100	6.98	2	76	27.1	123	71	217	49	168	12.3	5.8	58.4	1	1	1	1	1	1	1	72.4	0.12	14.7
1	1	0	100	6.97	2	72	27.1	139.5	80.5	251	49	202	13.8	5.7	78.9	1	1	1	0	0	1	1	131.8	0.13	31.9
1	1	0	100	7.07	2	69	23.5	135.5	85	230	49	181	13.3	5.2	75.9	2	1	1	1	0	1	1	11.7	0.04	16.6
1	1	0	100	7.01	2	70	28.7	131.5	76	201	49	152	14.5	6	80.0	1	1	1	0	1	1	1	25.2	0.03	11.3
1	1	0	100	6.87	2	72	23.7	126	75	168	49	119	16	7.2	70.6	2	1	1	1	0	0	1	24.7	0.08	5.57
1	0	0	100	0.42	1	86	24.3	137	81	186	49	137	14.5	5	55.2	2	1	1	1	1	1	1	8.1	0.08	24.7
1	1	0	100	6.85	1	84	24.3	172.5	87.5	211	49	162	15	5.4	49.9	2	1	1	0	1	1	1	2977.9	0.09	124
1	0	0	100	1.99	1	66	21.5	126	68.5	238	49	189	13.1	6.3	79.9	2	0	1	1	0	1	1	7.5	0.15	7.25
1	0	0	100	6.8	1	75	24.8	117.5	73	210	49	161	12.5	8.3	80.0	2	1	1	0	1	0	1	41	0.14	17.3
1	0	0	100	5.16	1	66	27.3	111.5	77	175	49	126	15.3	5.7	72.1	2	1	0	1	1	1	1	13.6	0.02	145
1	0	0	100	6.86	1	76	25.5	115.5	65.5	185	49	136	16	5.2	52.7	2	1	0	1	1	1	1	5	0.04	10.5
1	0	0	100	6.99	1	68	21.6	123.5	75	165	49	116	12.1	5.3	78.8	2	1	0	1	1	1	1	8.7	0.01	15.1
1	0	0	100	6.98	1	69	30.6	165.5	80.5	304	49	255	15.8	6.1	62.2	1	1	1	0	0	1	1	65.3	0.05	78.3
1	0	0	100	6.79	1	65	22.3	125	84	227	49	178	13.3	5.1	80.5	2	1	0	1	0	1	1	9.6	0.03	7.1
1	1	0	100	4.65	1	68	23.8	150	75.5	200	49	151	15.9	4.9	78.8	2	0	0	0	1	1	1	448.4	0.08	1.37
1	0	0	100	7	2	74	24.3	148.5	80	166	49	117	12.4	5.7	73.2	1	1	1	0	1	1	1	13.7	0.8	113
1	0	0	100	4.63	2	72	30.4	165.5	93.5	249	49	200	13.9	5.9	70.6	1	1	1	0	0	1	1	64.8	0.34	44.1
1	0	0	100	6.8	2	66	28.4	116	77	216	49	167	14.1	5.7	82.3	2	1	1	1	1	1	1	8.5	0.1	24.5
1	0	0	100	4.62	2	75	25.5	132	68.5	172	49	123	13.4	5	58.8	2	1	1	1	1	1	1	13.9	0.12	26.6
1	0	0	100	4.67	2	68	29	141	81	251	49	202	14.8	5.7	72.6	2	1	1	0	0	1	1	14.1	0.12	10.6
1	0	0	100	6.87	2	65	25.1	130.5	92.5	221	49	172	13.3	5.1	82.8	2	1	1	0	0	1	1	30.2	0.19	22.9
1	0	0	100	6.92	2	70	27	116	74	125	49	76	13.7	8.4	75.3	1	1	1	0	1	0	1	15.3	0.01	93
1	0	0	100	7.08	2	74	25.3	147	84	188	49	139	13	5.2	59.2	1	1	1	0	1	1	1	7.2	0.06	31
1	0	0	100	5.15	2	77	22.9	129	78	163	49	114	14.2	6.5	68.2	1	1	1	0	0	0	1	31.5	0.02	24.7
1	0	0	100	5.16	2	77	27.2	136	64.5	164	49	115	15	5.3	71.7	1	1	1	0	1	1	1	38.9	0.05	48.7
1	0	0	100	5.16	2	65	28.9	118.5	68.5	203	49	154	13.8	5.4	82.8	2	1	1	1	1	1	1	12.8	0.06	21.3
1	0	0	100	7.21	2	70	23.5	139	69.5	197	49	148	13.7	5.2	75.3	2	1	1	1	1	1	1	29.5	0.02	31.6
1	0	0	100	7	2	73	27.9	160.5	84	247	49	198	13.3	5.2	78.3	1	1	1	0	0	1	1	16.3	0.07	58.2
1	0	0	100	7.01	2	67	22.5	136.5	63.5	143	49	94	11	5.7	81.7	2	1	1	1	1	1	1	20.7	0.01	24.2
1	0	0	100	7.03	2	79	24.2	106	61.5	230	49	181	13.1	5	70.7	2	1	1	1	0	1	1	9.6	0.04	3.78
1	0	0	100	5.05	2	66	25.8	117.5	72.5	205	49	156	13.8	5.2	82.3	2	1	1	1	1	1	1	23.6	0.04	10.8
1	0	0	100	5.05	2	70	24.4	115.5	71.5	174	49	125	13.1	5.8	75.3	2	1	1	0	1	1	1	52.6	0.07	16.5
1	0	0	100	5.06	2	67	22.5	126	82	200	49	151	12.5	6.1	76.9	2	1	1	0	1	1	1	53.2	0.07	20.4
1	0	0	100	4.62	2	74	24	116	75	192	49	143	13.2	5.6	51.4	1	1	1	1	1	1	1	9.5	0.03	26
1	0	0	100	4.64	2	68	31	147.5	76	195	49	146	14.9	5.7	81.1	2	1	1	0	1	1	1	8.9	0.06	32.5
1	0	0	100	6.79	2	66	26.5	125.5	66	239	49	190	11.6	5.7	62.7	2	1	1	0	0	1	1	14.8	0.04	18.2
1	0	0	100	6.8	2	76	23.7	148.5	76	178	49	129	12.3	5.5	76.7	1	1	1	0	1	1	1	77.1	0.02	41.4
1	0	0	100	6.82	2	65	24.2	114.5	67.5	248	49	199	12.6	5.7	74.2	2	1	1	0	0	1	1	3.2	0.05	8.27
1	0	0	100	6.83	2	69	22.9	168	87	206	49	157	12.8	5.3	80.5	2	1	1	0	1	1	1	151.6	0.09	4.66
1	0	0	100	6.79	2	73	23.7	105	60	203	49	154	14.9	7.4	59.7	1	1	0	1	1	0	1	7.6	0.04	22.2
1	0	0	100	5.05	2	70	26.4	153.5	87.5	272	49	223	13.3	5.4	75.3	2	1	1	0	0	1	1	32.5	0.08	9.96
1	0	0	100	5.06	2	71	30.5	130	74.5	191	49	142	12.3	5.7	60.5	1	1	0	0	1	1	1	27.3	0.07	40.1
1	0	0	100	5.07	2	77	20.2	102	66	132	49	83	13.9	5.6	76.1	2	1	1	1	1	1	1	26.1	0.01	31.2
1	1	0	100	7.08	2	78	21.1	146	91	143	49	94	11.6	5.5	71.2	2	1	1	0	1	1	1	46.1	0.42	7.27
1	1	0	100	4.62	2	78	24.7	109.5	57	140	49	91	14.6	7.1	71.2	2	1	1	0	1	0	1	16.5	1.27	19.1
1	1	0	100	4.64	2	70	25.8	135.5	77.5	197	49	148	13.6	5.7	71.6	2	1	1	1	1	1	1	10.3	0.03	11.1
1	1	0	100	6.96	2	71	24.9	135	90.5	209	49	160	12.8	5.1	71.1	1	1	1	0	1	1	1	74.3	0.02	23.1
1	0	0	100	6.87	2	80	31.6	128	73.5	219	49	170	14.9	5.9	70.2	2	1	1	0	1	1	1	68.8	0.24	15.9

1	1	0	100	7	2	80	27.2	122.5	69.5	174	49	125	13.8	5.3	66.7	1	1	1	0	1	1	1	22.6	0.18	45.1
1	1	0	100	6.44	2	86	23.1	100	55.5	200	49	151	14.4	5.2	71.5	2	1	1	1	0	1	0	41	0.04	28.9
1	1	0	100	6.86	2	80	23.9	130.5	71	262	49	213	12	5.5	56.8	1	1	1	0	0	1	1	31.5	0.08	11.7
1	0	1	0.3	0.3	1	67	21.6	144.5	92.5	220	49	171	14.9	6.1	79.4	1	1	0	0	0	1	1	11.2	0.14	52.3
1	1	1	1.6	1.6	1	77	24.2	141	72.5	223	49	174	16	5.1	70.1	1	1	1	0	0	1	1	39.1	0.26	22.3
1	1	1	0	0	1	72	21	137	82.5	179	49	130	16.7	5	72.6	2	1	0	1	1	1	0	276.5	0.06	221
1	1	1	0.5	0.5	1	80	25.5	139	80	206	49	157	13.8	6.4	68.6	2	1	0	0	1	1	0	414.8	0.23	236
1	1	1	4.3	4.3	2	73	26.5	132.5	90.5	212	49	163	14	5.3	73.8	2	1	1	0	1	1	1	17.2	0.03	26.3
1	0	1	1.6	1.6	1	81	30.3	151.5	73.5	224	49	175	14.1	5.1	68.1	1	1	1	0	0	1	1	5	0.17	53.3
1	0	0	100	6.86	1	71	21.9	108.5	65.5	235	50	185	13.7	5.2	61.3	2	1	1	1	0	1	1	5.2	0.19	28.4
1	0	0	100	6.87	1	68	21	112	81	204	50	154	14.4	5.8	74.6	2	1	1	1	1	1	1	16	0.41	34.2
1	0	0	100	6.84	1	71	24.6	125	72	191	50	141	14.1	5.5	73.1	2	1	0	1	1	1	1	38.6	0.18	42.7
1	0	0	100	6.85	1	67	21.5	151	87.5	230	50	180	14.4	5.4	50.6	2	1	0	0	0	1	1	8.3	0.49	2.24
1	0	0	100	4.64	1	70	28.2	166.5	97.5	237	50	187	15.2	5.6	70.1	2	1	0	0	0	1	1	1859.7	0.69	29.5
1	0	0	100	5.04	1	71	24.8	156.5	100.5	243	50	193	15.3	5.2	69.6	2	1	0	0	0	1	1	26.8	0.37	14.4
1	0	0	100	4.69	1	68	22.6	151.5	82	156	50	106	13.9	5.4	78.8	2	1	0	0	1	1	1	30.1	2.67	122
1	0	0	100	6.87	1	69	24.9	105	71	196	50	146	14.1	4.9	70.6	2	1	0	0	1	1	1	5.5	0.17	17.9
1	0	0	100	6.86	1	67	24.6	106	61	169	50	119	14.2	5	75.2	2	1	1	1	1	1	1	39.9	0.01	9.84
1	0	0	100	3.62	1	77	23.8	136.5	71.5	210	50	160	15.6	5.3	58.8	2	1	1	1	1	1	1	5	0.03	0.05
1	0	0	100	7.23	1	69	28.9	159	88	188	50	138	14.2	5.2	49.9	2	1	1	0	0	1	1	97.9	0.07	26
1	0	0	100	6.88	1	71	25.9	116.5	71.5	196	50	146	13.3	5.6	77.2	2	1	0	0	1	1	1	10.8	0.04	66.1
1	0	0	100	5.16	1	70	24.5	143.5	76.5	216	50	166	16.2	5.9	70.1	2	1	0	0	1	0	1	17.2	0.01	15.1
1	0	0	100	6.84	1	69	25.7	116	80	165	50	115	15.1	6.2	74.1	2	0	1	0	1	0	1	13.1	0.05	128
1	0	0	100	7.21	1	72	23	95	62.5	199	50	149	14.3	5.6	69.1	2	0	1	1	0	1	1	17.1	0.03	38.7
1	0	0	100	6.86	1	67	19.6	171.5	90.5	189	50	139	14.4	6.7	75.2	2	1	1	0	1	0	1	54.2	0.04	35.7
1	0	0	100	7.01	1	70	19.3	128.5	78	160	50	110	12.3	5.5	73.6	2	1	1	1	1	1	1	19.9	0.03	48.6
1	0	0	100	6.86	1	69	26	147	95	199	50	149	15.6	5.5	70.6	2	0	1	0	1	1	1	12.2	0.06	10.2
1	0	0	100	7.02	1	74	23.2	128	78	172	50	122	14.6	5.7	75.6	2	0	0	1	1	1	1	26	0.06	31.9
1	0	0	100	7.02	1	65	20.3	102	61.5	189	50	139	13.7	5.3	80.5	2	1	1	1	1	1	1	11.4	0.07	12.2
1	0	0	100	7.02	1	79	22.4	133	82.5	217	50	167	12	5.5	69.1	2	1	1	1	1	1	1	27.9	0.02	75.8
1	0	0	100	5.06	1	69	22.7	115	72.5	195	50	145	15.4	5.7	78.3	2	1	0	1	1	1	1	4.6	0.09	62.5
1	0	0	100	5.06	1	71	29.5	129	70.5	156	50	106	14	5.7	69.6	2	1	1	1	1	1	1	5.9	0.03	26.2
1	0	0	100	5.07	1	65	24.9	136	76.5	215	50	165	14.1	5.9	72.6	2	0	0	0	1	1	1	7	0.04	52.3
1	0	0	100	6.97	1	66	29.9	133.5	74.5	196	50	146	13.9	6.6	85.2	2	1	0	0	1	0	1	20.7	0.02	34.8
1	0	0	100	6.99	1	70	21.3	105	53.5	173	50	123	11.5	5.1	70.1	2	1	1	1	1	1	1	8.5	0.03	8.32
1	0	0	100	7	1	68	26.9	115	74	139	50	89	14.6	4.6	74.6	2	1	1	1	1	1	1	7.9	0.02	14.3
1	0	0	100	7	1	75	23.7	93.5	58.5	111	50	61	13.2	5.1	71.0	2	0	1	1	1	1	1	15.6	0.09	17.7
1	0	0	100	4.63	1	73	27.1	118	76	159	50	109	14.9	5.6	53.9	2	1	1	1	1	1	1	2.7	0.03	12.9
1	0	0	100	4.63	1	68	24.6	147	89	287	50	237	13.6	5	71.1	2	1	1	0	0	1	1	11.9	0.04	19.2
1	0	0	100	4.63	1	74	26.2	147	79.5	152	50	102	13.6	5.4	71.5	1	1	0	0	1	1	1	18	0.03	14.4
1	0	0	100	4.64	1	69	22.7	129	81	241	50	191	15.5	5.9	70.6	2	0	0	0	0	1	1	88.5	0.07	7.33
1	0	0	100	4.64	1	68	22.2	119	79	179	50	129	15.1	5.4	62.6	2	1	0	1	1	1	1	10.2	0.08	6.97
1	0	0	100	4.64	1	70	26.4	162.5	96	181	50	131	13.8	5.6	77.7	2	1	0	0	1	1	1	12.6	0.05	199
1	0	0	100	4.64	1	76	18.5	151	88.5	194	50	144	15.7	5.7	74.5	2	0	0	0	1	1	1	197.9	0.08	185
1	0	0	100	6.87	1	73	22	130.5	80.5	217	50	167	15.2	5.5	76.1	2	0	1	1	1	1	1	16.4	0.05	0.82
1	0	0	100	6.95	1	70	24.5	146	71	222	50	172	14.3	4.8	61.7	2	1	1	0	0	1	1	15.9	0.08	38.7
1	0	0	100	6.96	1	70	26.6	141.5	86.5	200	50	150	15.9	5.5	70.1	1	1	0	0	1	1	1	16.2	0.02	19.9
1	0	0	100	6.8	1	68	26.7	147	89.5	175	50	125	14.9	5.7	74.6	2	1	0	0	1	1	1	14.9	0.08	15.9
1	0	0	100	6.82	1	69	20.4	129.5	78	138	50	88	13.7	5.4	78.3	2	1	0	1	1	1	1	27.4	0.07	67.2
1	0	0	100	6.84	1	70	22.9	138.5	90.5	186	50	136	15	5.3	61.7	2	1	0	0	1	1	1	6	0.04	23.2
1	0	0	100	6.84	1	72	23.7	142	86	180	50	130	14.8	5.3	72.6	2	1	0	0	1	1	1	5.6	0.03	42.9

1	0	0	100	4.62	1	67	27.3	113.5	65	253	50	203	15.4	5.6	75.2	2	1	0	1	0	1	1	23.4	0.03	8.02
1	0	0	100	4.62	1	70	22.9	141	78	174	50	124	14.3	5.8	77.7	2	1	0	0	1	1	1	3.4	0.05	30
1	0	0	100	4.62	1	65	25.2	189	105	131	50	81	15.9	5	76.2	2	0	0	0	1	1	1	7.6	0.09	39
1	0	0	100	4.62	1	73	25.6	140	78.5	159	50	109	13.2	5.1	76.1	2	0	0	0	1	1	1	13	0.01	38.4
1	0	0	100	6.44	1	71	29.9	128	81	259	50	209	16.6	6.8	82.2	2	1	1	1	0	0	1	19.5	0.09	2.04
1	0	0	100	6.96	1	66	28.4	130.5	75	173	50	123	16.4	5.3	75.7	2	1	1	0	1	1	1	3	0.05	0.52
1	0	0	100	5.36	1	66	22.8	156	83.5	220	50	170	15.3	5.8	72.1	2	1	0	0	0	1	1	20.2	0.04	56
1	0	0	100	5.06	1	69	21.3	113	76.5	186	50	136	12.7	5.5	78.3	2	0	0	0	1	1	1	23.6	0.08	19.6
1	0	0	100	6.85	1	65	21.5	122.5	65.5	193	50	143	13.9	6.7	76.2	2	1	1	1	1	0	1	6.6	0.02	26.3
1	0	0	100	5.04	1	76	22.4	138	75.5	196	50	146	13.5	6.7	59.2	2	1	0	0	1	0	1	91.9	0.03	12.3
1	0	0	100	5.04	1	72	24.7	108	65	151	50	101	13.1	5.3	60.9	1	1	1	1	1	1	1	4.9	0.02	60.6
1	0	0	100	5.04	1	74	26.5	129.5	66.5	186	50	136	13.6	5.5	75.6	2	1	0	0	1	1	1	51.9	0.05	73.1
1	1	0	100	6.33	1	79	22	112	67.5	191	50	141	14.1	5	65.8	2	0	1	1	1	1	1	37	0.1	18.9
1	1	0	100	4.59	1	76	27.1	151.5	79	205	50	155	13.5	6	67.2	2	1	1	0	1	1	1	10.4	0.09	46
1	1	0	100	4.59	1	75	20.9	146.5	86.5	153	50	103	15.4	4.7	71.0	1	1	0	0	1	1	1	6.2	0.02	17
1	0	0	100	6.84	2	74	25.1	168.5	88.5	228	50	178	14.5	5.7	69.6	1	1	1	0	0	1	1	59.8	0.13	29.9
1	0	0	100	6.85	2	76	22.9	125	67.5	294	50	244	14.1	5.8	58.4	1	1	1	1	0	1	1	12.9	0.23	36.4
1	0	0	100	5.07	2	69	22.4	106	63	164	50	114	13	5.4	80.5	2	1	1	1	1	1	1	86.2	0.48	24.3
1	0	0	100	5.08	2	73	22.1	126	71	194	50	144	12.1	6.4	73.8	2	1	1	1	1	0	1	7.4	0.18	38.8
1	0	0	100	6.87	2	77	29.1	166	93	187	50	137	13.4	5.6	71.7	2	1	1	0	0	1	1	119.3	0.13	25.7
1	0	0	100	6.96	2	74	25.8	135	80	173	50	123	13	5.5	73.2	1	1	1	1	1	1	1	28.5	0.68	13.4
1	0	0	100	6.8	2	73	25.4	129	76	193	50	143	12.7	7	70.1	2	1	1	1	1	0	1	13.1	0.13	9.76
1	0	0	100	6.83	2	66	22	102.5	57	190	50	140	12.4	5.3	73.6	2	1	1	0	1	1	1	3.5	0.41	11.1
1	0	0	100	4.6	2	78	28.1	123	69	212	50	162	12.9	5.5	57.6	1	1	1	1	1	1	1	7.3	0.16	26.4
1	0	0	100	7.08	2	66	25	116.5	80	179	50	129	12.7	5	73.6	2	1	1	1	1	1	1	11.9	0.01	75.2
1	0	0	100	7.08	2	66	28	128	81.5	208	50	158	12.8	5.1	82.3	2	1	1	1	1	1	1	27.1	0.04	6.89
1	0	0	100	4.58	2	67	23.3	149.5	84	217	50	167	13.9	5.2	73.1	2	1	1	0	1	1	1	19.6	0.03	3.32
1	0	0	100	4.58	2	72	21.1	127.5	70.5	207	50	157	13.1	6.1	70.6	2	1	1	1	1	0	1	15.4	0.01	24.6
1	0	0	100	4.58	2	73	19.9	182	105.5	251	50	201	12.4	5	78.3	2	1	1	0	0	1	1	32.2	0.01	32.6
1	0	0	100	4.59	2	75	20.8	124.5	77	164	50	114	10.7	6.2	69.1	2	1	1	1	1	1	1	33.4	0.02	100
1	0	0	100	6.86	2	75	28.5	121.5	72	183	50	133	13.5	5.4	69.1	2	1	1	1	1	1	1	20.9	0.01	81
1	0	0	100	6.87	2	76	21.1	123.5	73.5	208	50	158	13.1	5.5	68.6	1	1	1	1	1	1	1	36.8	0.05	15.7
1	0	0	100	6.87	2	65	25.4	127.5	82.5	194	50	144	15.6	5.6	78.0	2	1	1	1	1	1	1	5.1	0.06	29
1	0	0	100	6.88	2	70	23.1	108	61.5	212	50	162	13.2	5.3	60.9	2	1	0	1	1	1	1	12.7	0.05	22.2
1	0	0	100	6.88	2	79	20.4	118	64.5	242	50	192	13.6	5.3	49.6	2	1	1	1	0	1	1	23.9	0.06	81.4
1	0	0	100	5.16	2	78	21.4	161	88.5	212	50	162	13	5.5	57.6	1	1	1	0	1	1	1	18	0.01	25.4
1	0	0	100	6.84	2	65	25.1	151.5	95.5	203	50	153	13	5.4	74.2	2	1	0	0	1	1	1	12.2	0.06	47
1	0	0	100	6.84	2	70	22.6	151	77	196	50	146	13.4	5.9	75.3	2	1	1	0	1	1	1	17	0.05	24.9
1	0	0	100	7.02	2	73	26.2	167.5	86	201	50	151	12.3	6	73.8	1	1	0	0	1	1	1	96.6	0.02	56.5
1	0	0	100	7.04	2	66	25.4	139	80	201	50	151	12.3	5.5	82.3	2	1	1	0	1	1	1	14.8	0.01	1.18
1	0	0	100	7.04	2	69	27	157	75.5	241	50	191	15.8	5.5	75.9	1	1	1	0	0	1	1	67.7	0.07	0.05
1	0	0	100	5.05	2	65	28.4	139.5	89.5	163	50	113	12.4	5.5	78.0	2	1	1	0	1	1	1	11.3	0.02	12.6
1	0	0	100	5.06	2	66	27.7	136.5	84	228	50	178	12.9	5.5	82.3	2	1	1	0	0	1	1	46.6	0.06	16.5
1	0	0	100	5.06	2	65	19.7	130	68	156	50	106	12.4	5.8	74.2	2	1	1	0	1	1	1	2.8	0.08	30
1	0	0	100	6.98	2	75	24.2	124.5	68.5	194	50	144	12	5.7	69.1	1	1	0	1	1	1	1	19.5	0.08	12.1
1	0	0	100	6.98	2	67	24.9	124	77	197	50	147	11	6	73.1	2	1	1	1	1	1	1	23.5	0.05	14.2
1	0	0	100	6.99	2	65	28.5	124	85	186	50	136	11.5	5	78.0	2	1	1	1	1	1	1	44.7	0.02	14.2
1	0	0	100	7	2	67	25.1	140	81.5	158	50	108	11.5	5.7	76.9	2	1	1	0	1	1	1	25.2	0.01	33.1
1	0	0	100	4.63	2	74	21.7	121.5	74.5	168	50	118	11.8	5.5	51.4	2	1	1	1	1	1	1	11.6	0.05	110
1	0	0	100	4.63	2	76	19.9	104	61	208	50	158	13	5.3	68.6	2	1	1	1	1	1	1	14.8	0.06	15.1
1	0	0	100	4.63	2	71	22.7	150.5	86.5	191	50	141	12.9	5.9	74.8	2	1	1	0	1	1	1	36.7	0.05	30.5

1	0	0	100	6.95	2	65	27	157	89	216	50	166	13.9	5.8	74.2	2	1	1	0	1	1	1	26.7	0.05	18.6
1	0	0	100	6.96	2	66	29.2	140	72	213	50	163	15.4	5.2	82.3	2	1	1	0	1	1	1	407.9	0.06	0.05
1	0	0	100	6.98	2	69	23.5	136	81	217	50	167	13.6	5.3	75.9	2	1	1	1	1	1	1	24.3	0.05	12
1	0	0	100	6.8	2	75	20.9	128.5	70	206	50	156	11.3	5.9	58.8	2	1	1	0	0	1	1	125.2	0.04	17.1
1	0	0	100	6.83	2	68	29.5	139.5	90	143	50	93	13.7	4.9	72.6	1	1	1	0	1	1	1	7.2	0.02	13.5
1	0	0	100	6.84	2	71	21.7	152	80	212	50	162	11	5.3	71.1	2	1	1	0	1	1	1	18.2	0.01	47
1	0	0	100	4.59	2	68	24.1	116	73	230	50	180	12.4	5.4	61.8	2	1	1	1	0	1	1	36	0.03	9.45
1	0	0	100	4.59	2	76	30.2	118	61.5	207	50	157	11.3	6.4	82.5	1	1	1	0	0	1	1	31.8	0.03	30.7
1	0	0	100	4.69	2	71	23.5	160	94.5	202	50	152	13.1	5.5	74.8	1	1	1	0	1	1	1	223.8	0.02	25.1
1	0	0	100	6.96	2	65	23.6	111.5	61	205	50	155	14	6.3	74.2	2	1	1	0	1	1	1	6.4	0.04	11.8
1	0	0	100	5.37	2	70	21.2	109	57.5	179	50	129	11.9	5.6	75.3	2	1	1	1	0	1	1	10.7	0.02	14.1
1	0	0	100	5.06	2	70	25.9	125.5	74	195	50	145	13.9	5.8	75.3	2	1	1	0	1	1	1	7.8	0.07	19.4
1	0	0	100	6.86	2	68	23.4	105	71	141	50	91	11.7	5.6	76.4	2	1	1	1	1	1	1	27.2	0.09	11.4
1	0	0	100	5.04	2	69	27.3	175.5	98	179	50	129	13.3	6.4	80.5	2	1	1	0	1	1	1	39.2	0.03	87.7
1	1	0	100	7.2	2	72	27.3	127.5	64	207	50	157	8.2	6.3	52.1	1	1	0	1	1	1	1	28.3	9.37	56.3
1	1	0	100	5.04	2	77	28	144.5	68.5	178	50	128	13.6	5.6	50.3	2	1	1	0	1	1	1	166.5	0.17	23.6
1	1	0	100	7.06	2	72	21.4	142.5	84	180	50	130	11.5	5.1	74.3	2	1	1	0	1	1	1	14.1	0.01	38.2
1	1	0	100	7.07	2	71	22.5	119	71	216	50	166	12.3	5.1	60.5	2	1	1	1	1	1	1	41.8	0.02	27.6
1	1	0	100	4.58	2	73	24.7	115.5	75	218	50	168	13.1	5.9	70.1	1	1	1	1	0	1	1	214.2	0.02	63.2
1	1	0	100	6.56	2	74	21.9	159	93	152	50	102	14.4	5.7	73.2	2	1	1	0	1	1	1	170.2	0.01	16.2
1	0	0	100	7	1	81	27.4	156.5	80	205	50	155	13.6	5.3	64.9	2	1	0	0	1	1	1	37.5	0.05	79.8
1	0	0	100	7.08	1	80	23	138	72	170	50	120	13.6	5	65.4	2	1	1	1	1	1	1	6.5	0.03	28
1	0	0	100	5.08	1	81	26	126	75	200	50	150	15.9	5.7	76.7	2	1	0	1	1	1	1	20.2	0.05	50.8
1	0	0	100	5.37	1	80	21.6	105	68.5	156	50	106	14.5	5.3	65.4	1	1	0	1	1	1	1	6.9	0.01	15.7
1	0	0	100	6.85	1	86	27.1	160.5	71	203	50	153	14.3	5.4	62.7	1	1	0	0	1	1	1	880.9	0.05	178
1	0	0	100	3.87	1	73	20.5	94	60	184	50	134	15	5.3	72.0	2	1	1	1	1	1	1	26.2	0.53	10.4
1	0	0	100	7.06	1	68	27.6	114	66.5	128	50	78	13.1	5.3	74.6	1	1	1	1	1	1	1	10.2	0.02	39
1	0	0	100	4.58	1	74	24	140	82	199	50	149	14.5	5.4	75.6	2	1	0	0	1	1	1	7.2	0.03	45.5
1	0	0	100	6.86	1	70	25.6	159.5	85.5	231	50	181	13.9	7	61.7	2	1	1	0	0	0	1	12.3	0.02	47
1	0	0	100	6.98	1	68	27.6	129.5	84.5	205	50	155	16	5.6	71.1	2	1	0	0	1	1	1	33.1	0.08	42.7
1	0	0	100	3.03	1	69	31.3	141.5	86.5	178	50	128	15	5.2	62.2	2	0	0	0	1	1	1	5.2	0.09	20
1	0	0	100	6.83	1	79	19.7	132.5	73.5	268	50	218	14.4	5.7	65.8	1	1	1	1	0	1	1	15.8	0.07	8.91
1	0	0	100	5.05	1	74	23	105.5	65.5	152	50	102	14	5.6	75.6	2	1	0	0	1	1	1	9.4	0.07	74.5
1	0	0	100	1	1	78	17.7	101	66.5	130	50	80	12.3	5.5	69.6	2	1	1	0	1	1	1	23.5	0.02	121
1	1	0	100	4.05	1	76	21	128.5	71.5	183	50	133	13.5	5.3	70.5	2	0	1	0	1	1	1	76.7	0.17	29.2
1	1	0	100	4.61	1	78	23	149.5	66.5	158	50	108	8.4	4.6	52.0	2	1	1	0	1	1	0	17.5	0.03	103
1	0	0	100	7.05	2	69	26.7	164.5	97.5	235	50	185	13	5.2	72.1	2	1	1	0	0	1	1	41.4	0.32	31
1	0	0	100	6.85	2	73	20.7	114	66	189	50	139	11.4	5.3	73.8	2	1	1	1	1	1	1	17.9	0.42	42.2
1	0	0	100	6.98	2	72	26.5	144	72.5	248	50	198	12	5.7	74.3	1	1	1	0	0	1	1	13.6	0.21	24.7
1	0	0	100	6.96	2	66	28.2	135.5	76.5	208	50	158	14.4	5	62.7	2	1	1	0	1	1	1	19.3	0.11	14.9
1	0	0	100	6.98	2	73	28.1	178	83	228	50	178	14	5.5	73.8	1	1	1	0	0	1	1	22.2	0.45	13.6
1	0	0	100	6.8	2	71	26.1	130.5	73	250	50	200	12	4.9	52.5	2	1	1	0	0	1	1	9.9	0.13	41.2
1	0	0	100	6.83	2	65	24.9	131	76.5	333	50	283	12.9	4.8	78.0	2	1	1	1	0	1	1	10.3	0.25	20.2
1	0	0	100	4.59	2	68	28.5	136.5	64.5	214	50	164	12.2	5.3	81.1	2	1	1	1	0	1	1	27.1	0.13	28.2
1	0	0	100	6.87	2	72	28.6	123	68	238	50	188	14.4	5.4	70.6	1	1	1	0	0	1	1	15.2	0.17	41.1
1	0	0	100	7.06	2	76	27.5	113	66	235	50	185	12.7	5.4	50.7	1	1	1	1	0	1	1	11.4	0.03	37.8
1	0	0	100	7.06	2	66	28.4	116.5	67.5	232	50	182	14	5.4	73.6	2	1	1	1	0	1	1	137.7	0.07	0.05
1	0	0	100	6.86	2	66	29.9	131.5	74	220	50	170	14.1	5.6	77.5	2	1	1	0	0	1	1	14.6	0.03	7.94
1	0	0	100	6.87	2	76	21.7	160.5	87	141	50	91	12.9	5.2	58.4	1	1	1	0	1	1	1	5	0.07	75.6
1	0	0	100	6.98	2	66	31.9	165	89.5	200	50	150	11.9	5.2	73.6	2	1	1	0	0	1	1	55.6	0.08	35.1
1	0	0	100	7	2	69	26.2	130	71.5	196	50	146	11.6	5.3	61.4	1	1	1	1	1	1	1	7.2	0.04	41.9

1	0	0	100	7	2	71	22.4	144	83	206	50	156	11.9	4.9	74.8	1	1	1	0	1	1	1	26.3	0.04	10.7
1	0	0	100	4.62	2	65	24.9	154	80.5	217	50	167	14.4	7.6	78.0	2	1	1	0	1	0	1	24.6	0.06	7.86
1	0	0	100	4.69	2	67	20.8	125	71	176	50	126	14	5.4	62.2	2	1	1	1	1	1	1	9.1	0.06	16.6
1	0	0	100	6.86	2	67	23.1	152	78.5	200	50	150	13.2	5.5	76.9	2	1	1	0	0	1	1	33.3	0.08	4.17
1	0	0	100	5.04	2	75	21.3	118	66	141	50	91	11.5	5.9	72.7	2	1	1	1	1	1	1	26.9	0.06	39.5
1	0	0	100	5.04	2	65	23.8	118	78.5	152	50	102	12.8	5.6	78.0	2	1	1	1	1	1	1	5.2	0.02	8.86
1	1	0	100	4.63	2	78	30.8	126.5	74	251	50	201	11.9	5.4	57.6	1	1	1	0	0	1	1	60.1	0.41	83.8
1	1	0	100	6.96	2	69	24	146.5	76.5	180	50	130	11.4	4.8	86.7	2	1	1	0	1	1	1	14.7	0.77	58.7
1	1	0	100	5.37	2	77	34	130.5	69.5	191	50	141	15.2	6.4	44.3	1	1	1	0	1	1	1	31.6	0.13	22.4
1	1	0	100	7.08	2	75	22.9	119	67	192	50	142	14.5	5.2	72.7	1	1	1	1	1	1	1	40.7	0.04	29.1
1	1	0	100	5.16	2	69	24.7	123.5	69	182	50	132	11.6	5.4	72.1	2	1	1	0	1	1	1	4.4	0.01	32.7
1	0	0	100	3.65	1	87	22	152	79	172	50	122	11.5	6	54.8	2	1	0	0	1	1	1	16	0.16	25.6
1	0	0	100	7.06	1	83	21.9	148.5	82	189	50	139	13.1	5.8	71.0	2	1	0	0	1	1	1	23.3	0.01	24.2
1	1	0	100	5.23	1	85	29.9	156.5	95.5	180	50	130	16	7.4	40.4	2	1	0	0	1	0	1	2117.6	0.57	44
1	1	0	100	7.08	2	84	24.3	152.5	83	278	50	228	12.1	5.5	64.9	1	1	1	0	0	1	1	56.6	0.13	73.4
1	1	1	0.9	0.9	1	77	25.3	167.5	84	139	50	89	15.5	4.9	74.0	2	1	0	0	1	1	0	12.7	0.17	221
1	1	1	3.2	3.2	1	69	24.7	150	88.5	205	50	155	16	6.5	78.3	2	1	0	0	1	0	1	291.4	1.43	5.66
1	1	1	0.6	0.6	1	67	23.1	144	83	150	50	100	15.3	8.1	79.4	2	1	0	0	1	0	1	92	0.03	24.6
1	0	1	3.5	3.5	2	71	32.3	124	101.5	252	50	202	14.7	5.4	74.8	2	1	1	0	0	1	1	204.9	0.12	11.7
1	0	1	2.1	2.1	1	80	25.2	107.5	61.5	210	50	160	13.4	5.7	46.2	1	0	1	1	1	1	1	3.9	0.1	78.4
1	1	1	1.6	1.6	1	75	25.8	140	84.5	255	50	205	14.1	5.8	71.0	1	0	0	0	0	1	1	113.8	0.28	345
1	1	1	4.4	4.4	2	76	23.2	102.5	68	233	50	183	11.3	5.2	39.7	2	1	1	0	0	1	1	12.8	0.03	40.4
1	0	0	100	5.07	1	77	25.2	136	65	192	51	141	12.2	5.8	66.7	2	1	0	1	1	1	0	25.7	0.15	112
1	0	0	100	7.07	1	79	26.6	107	63	212	51	161	11.8	5.5	65.8	2	1	1	0	1	1	1	54.2	0.12	13.1
1	0	0	100	4.59	1	66	25.6	138.5	78	231	51	180	16.6	5.3	72.1	2	0	0	1	0	1	1	15.9	0.14	4.92
1	0	0	100	6.84	1	69	24.4	148.5	80.5	191	51	140	13.5	5.5	70.6	2	0	1	0	1	1	1	26.5	0.14	20.5
1	0	0	100	7.02	1	77	23.1	140	83	177	51	126	14.7	5.3	70.1	2	0	1	0	1	1	1	6	0.68	62.8
1	0	0	100	1.91	1	70	19.5	173.5	104.5	187	51	136	14.1	10.8	73.6	1	1	0	0	1	0	1	758.6	0.25	9.13
1	0	0	100	6.83	1	72	21.5	128.5	82	176	51	125	13.6	5.2	60.9	2	1	0	1	1	1	1	7.3	0.12	18.2
1	0	0	100	6.44	1	73	22.9	137	83	185	51	134	13.7	5.9	76.1	2	1	1	0	1	1	1	22.6	0.15	25.4
1	0	0	100	4.67	1	66	22.5	151	94.5	164	51	113	16.5	4.9	75.7	2	1	0	0	1	1	1	11.9	1.55	14.1
1	0	0	100	5.05	1	76	23	128	73.5	165	51	114	13.2	8.2	70.5	2	1	1	1	1	0	1	10	0.28	15.7
1	0	0	100	6.85	1	76	22.4	171.5	88.5	253	51	202	16.5	5.8	59.2	1	1	1	0	0	1	1	99.1	0.1	19.2
1	0	0	100	2.41	1	73	22.8	120	73.5	219	51	168	13.5	4.9	53.9	2	1	1	0	1	1	1	12.7	0.04	44.4
1	0	0	100	4.58	1	71	24.5	125.5	72	181	51	130	13.6	5.8	73.1	2	1	0	0	1	1	1	3.8	0.06	39.1
1	0	0	100	6.87	1	68	24.7	119	70.5	243	51	192	14.4	5.8	71.1	2	1	1	1	0	1	1	3.1	0.03	30.5
1	0	0	100	3.6	1	70	29.3	112	60	177	51	126	14.1	5.8	73.6	2	1	1	1	1	1	1	20.3	0.05	23.4
1	0	0	100	5.16	1	66	29.2	120	73.5	212	51	161	15.4	5.2	75.7	2	1	0	1	1	1	1	6.3	0.07	19.4
1	0	0	100	5.16	1	68	23.3	129.5	87.5	235	51	184	15.8	5.3	71.1	2	1	1	0	0	1	1	5.8	0.08	5.24
1	0	0	100	6.85	1	67	26.1	136.5	84.5	221	51	170	16.2	5.4	79.4	2	1	0	1	0	1	1	10	0.02	1.99
1	0	0	100	6.85	1	73	30.4	122.5	71.5	199	51	148	14.2	5.3	72.0	2	0	0	1	1	1	1	59.9	0.09	2.5
1	0	0	100	6.31	1	65	21.1	107.5	70	247	51	196	15	5.5	76.2	2	0	0	1	0	1	1	4.5	0.03	6.54
1	0	0	100	5.08	1	67	23.2	166.5	103	169	51	118	13.5	6.1	75.2	2	1	0	0	1	1	1	62.8	0.09	43.3
1	0	0	100	6.97	1	69	29.3	143.5	87	215	51	164	14.7	5	70.6	2	1	0	0	1	1	1	8.2	0.05	7.6
1	0	0	100	6.98	1	72	17.8	116.5	75	180	51	129	13	5.4	69.1	2	1	1	1	1	1	1	7.2	0.01	0.05
1	0	0	100	5.46	1	78	22.7	119	63.5	140	51	89	13.3	5.5	73.5	1	0	0	1	1	1	1	5.2	0.02	40.1
1	0	0	100	4.62	1	75	23.1	95.5	59	149	51	98	15.3	5.5	75.1	2	1	1	1	1	0	1	9.3	0.03	135
1	0	0	100	4.63	1	73	30.9	147.5	74	160	51	109	13	5.7	60.4	1	1	0	0	1	1	1	7	0.06	26.9
1	0	0	100	6.95	1	68	24	181.5	101.5	198	51	147	16.6	5.6	71.1	2	1	0	0	1	1	1	228.3	0.04	4.83
1	0	0	100	6.98	1	66	23.3	131.5	76	212	51	161	14.3	5.3	79.9	2	1	0	1	1	1	1	16.2	0.03	26.9
1	0	0	100	6.98	1	74	25.5	172.5	81	189	51	138	15.6	5.9	68.2	2	0	1	0	1	0	1	56.7	0.06	19.1

1	0	0	100	6.98	1	79	19.8	125	75.5	166	51	115	14.3	4.7	69.1	1	1	1	1	1	1	1	7.9	0.03	44.5
1	0	0	100	6.83	1	65	24.2	106	70	182	51	131	13.3	5.5	72.6	2	1	0	0	1	1	1	10.7	0.07	49.3
1	0	0	100	4.62	1	73	26.3	114	69	198	51	147	14.7	5.5	72.0	2	1	1	0	1	1	1	8	0.05	22.4
1	0	0	100	4.62	1	74	18.5	101	61.5	154	51	103	14.7	5.4	71.5	2	0	1	1	1	1	1	5.5	0.07	6.62
1	0	0	100	3.99	1	74	25.4	146	85.5	227	51	176	17.5	5.1	75.6	2	1	0	0	0	1	0	9.2	0.03	138
1	0	0	100	6.44	1	74	24.8	114	60	172	51	121	14.2	5.4	71.5	2	0	1	1	1	1	1	13.3	0.07	1.35
1	0	0	100	4.67	1	68	27.8	122	79	235	51	184	15.9	5.2	74.6	2	1	0	1	0	1	1	8	0.06	10.8
1	0	0	100	4.69	1	68	24.1	99.5	63.5	212	51	161	15.2	6.1	78.8	2	1	1	1	1	1	1	29.1	0.06	22.5
1	0	0	100	4.7	1	73	21.6	102	65.5	183	51	132	15.1	5.4	76.1	2	0	1	1	1	1	1	5.7	0.02	11.7
1	0	0	100	6.96	1	68	25.2	157.5	82.5	196	51	145	14.2	5.2	74.6	2	1	0	0	1	1	1	3.7	0.03	0.05
1	0	0	100	5.36	1	72	19.5	107.5	70	158	51	107	15	5.1	81.7	2	1	1	1	1	1	1	11.9	0.02	34.7
1	0	0	100	5.37	1	66	19.9	111.5	67.5	173	51	122	13.9	5.6	79.9	2	1	0	1	1	1	1	10.2	0.05	21.8
1	0	0	100	6.85	1	74	24.9	189	101.5	137	51	86	14.9	5.6	71.5	2	0	1	0	1	1	1	14.2	0.04	39.9
1	0	0	100	6.86	1	73	25.1	133.5	76	183	51	132	14	5.4	68.6	2	0	0	1	1	1	1	15.4	0.07	17.9
1	0	0	100	5.04	1	71	22.5	131	71	163	51	112	14.4	5.4	73.1	2	1	1	0	1	1	1	22.1	0.04	4.05
1	1	0	100	4.63	1	69	21.9	123.5	66.5	185	51	134	14.5	6.6	74.1	2	0	0	0	1	0	1	38.7	0.15	32.3
1	0	0	100	7.07	2	77	28.5	148	86.5	209	51	158	12.6	5.6	71.7	1	1	1	0	0	1	1	29.5	0.35	29.7
1	0	0	100	4.59	2	79	21	153.5	84	223	51	172	13.8	5.5	67.2	2	1	1	0	0	1	1	15.7	0.54	18.6
1	0	0	100	5.08	2	70	21.7	128.5	80.5	153	51	102	14.5	5.1	71.6	1	1	1	1	1	1	1	11.5	0.12	26.2
1	0	0	100	4.64	2	68	23.5	125	70	176	51	125	11.9	5.2	76.4	2	1	1	1	1	1	1	23.4	0.22	10.3
1	0	0	100	4.6	2	78	25.9	159.5	81.5	244	51	193	13.3	5.8	67.7	2	1	1	0	0	1	1	32.9	0.13	35.6
1	0	0	100	4.62	2	76	29.1	135.5	70.5	169	51	118	14.5	5.6	68.6	1	1	1	0	1	1	1	13.9	0.12	14.7
1	0	0	100	4.62	2	75	27.7	136	69.5	189	51	138	12.7	5.3	58.8	2	1	1	0	1	1	1	10.8	0.1	88.3
1	0	0	100	4.62	2	71	28.1	185	88	185	51	134	14.3	5.1	79.4	1	1	0	0	0	1	0	78.2	0.64	73.3
1	0	0	100	5.36	2	73	21.6	108	65.5	235	51	184	14	5.6	59.7	2	1	0	1	0	1	1	6.1	0.39	27.7
1	0	0	100	6.85	2	74	26.6	164.5	96	238	51	187	13	5.5	59.2	2	1	1	0	0	1	1	56.4	0.16	42
1	0	0	100	5.04	2	66	22	122.5	75	148	51	97	12.2	5.3	73.6	2	1	1	1	1	1	1	39.6	0.62	18.3
1	0	0	100	7	2	67	22.8	142.5	74	192	51	141	13	5.2	76.9	2	1	1	0	1	1	1	76	0.06	12.3
1	0	0	100	7.06	2	69	24.5	124	64	206	51	155	13.2	5.6	80.5	1	1	1	0	1	1	1	43.1	0.01	49.1
1	0	0	100	4.58	2	73	23.4	124	59	178	51	127	13.1	5.6	70.1	1	1	1	0	1	1	1	19	0.05	114
1	0	0	100	4.59	2	71	25.1	117.5	69.5	202	51	151	13.4	6	74.8	2	1	1	0	1	1	1	7.4	0.07	16.8
1	0	0	100	6.86	2	71	23.3	123.5	66.5	234	51	183	13	5.5	60.5	2	1	1	0	0	1	1	132.3	0.05	37.1
1	0	0	100	6.86	2	71	22.3	118	72	187	51	136	12.9	5.7	74.8	2	1	1	1	1	1	1	20.2	0.05	18.2
1	0	0	100	6.88	2	69	21	99.5	56.5	199	51	148	12.6	5.2	72.1	2	1	1	1	1	1	1	12.9	0.01	34.9
1	0	0	100	6.88	2	67	24.3	109.5	67.5	205	51	154	11.5	4.6	81.7	2	1	1	1	1	1	1	81.1	0.02	22.6
1	0	0	100	5.15	2	65	25.6	126	73.5	222	51	171	12.5	5.8	78.0	2	1	1	1	0	1	1	30	0.04	40.3
1	0	0	100	5.16	2	70	26.3	131.5	68.5	222	51	171	13	6.1	52.8	2	1	1	0	0	1	1	15.1	0.03	84.8
1	0	0	100	5.16	2	65	23.7	128	82	215	51	164	12.4	5.7	38.6	2	1	0	1	1	1	1	589.7	0.04	33.7
1	0	0	100	6.84	2	72	22.1	118	69	196	51	145	12.4	5.7	74.3	2	1	1	1	1	1	1	25.1	0.01	17.5
1	0	0	100	6.84	2	68	20.4	130.5	64	212	51	161	12.8	7.2	76.4	2	1	1	1	1	0	1	5.6	0.03	3.79
1	0	0	100	7.2	2	65	29.9	142.5	81.5	236	51	185	14.3	5.7	74.2	2	1	0	0	0	1	1	82.2	0.05	24.8
1	0	0	100	6.84	2	67	24.9	128	73.5	218	51	167	12.1	5.8	73.1	2	1	1	0	1	1	1	11.4	0.03	14.4
1	0	0	100	6.84	2	69	20.1	110.5	63	183	51	132	12.6	4.8	75.9	2	1	1	0	1	1	1	11.8	0.05	55
1	0	0	100	6.85	2	66	24.8	125.5	72	246	51	195	14.3	6.5	73.6	2	1	1	0	0	0	1	19.8	0.03	27.6
1	0	0	100	7	2	70	23.2	121.5	79.5	175	51	124	12.8	5.5	75.3	1	1	1	0	1	1	1	14.8	0.02	31
1	0	0	100	7.02	2	75	22.5	111.5	63.5	226	51	175	12.7	5.5	72.7	1	1	1	1	0	1	1	12.5	0.05	45.4
1	0	0	100	7.02	2	72	23.8	132	81	184	51	133	14.1	5.1	78.9	1	1	1	0	1	1	1	19.7	0.04	25.4
1	0	0	100	7.04	2	65	23.1	146	76	249	51	198	11	5.4	78.0	2	1	1	0	0	1	1	10	0.04	11.9
1	0	0	100	5.18	2	68	21.8	133.5	78	171	51	120	14.3	4.9	76.4	2	1	1	1	1	1	1	10.7	0.01	24.3
1	0	0	100	4.64	2	66	21.8	143.5	78	210	51	159	13.7	5.1	62.7	2	1	1	0	1	1	1	10.1	0.05	30.9
1	0	0	100	6.87	2	67	24.2	112	64.5	194	51	143	14.8	5.7	76.9	2	1	1	1	1	1	1	10.9	0.05	2.21

1	0	0	100	6.95	2	71	22.3	131.5	79.5	168	51	117	13.3	5.1	46.2	2	1	1	0	0	1	1	249.7	0.01	20.2
1	0	0	100	6.98	2	74	21.1	156	74.5	195	51	144	10.9	5.1	77.8	1	1	1	0	1	1	1	12.5	0.01	33
1	0	0	100	6.8	2	70	27.5	118	69	197	51	146	14.4	12.1	71.6	2	1	1	1	1	0	1	7.2	0.04	5.05
1	0	0	100	6.8	2	67	22.4	135.5	76.5	246	51	195	14	5.7	73.1	2	1	1	1	0	1	1	5.6	0.06	4.66
1	0	0	100	6.82	2	68	21.2	153.5	77	147	51	96	13.2	4.9	81.1	2	1	0	0	1	1	1	13.2	0.02	24.1
1	0	0	100	4.6	2	74	22.5	118	66.5	187	51	136	12.9	5.4	73.2	2	1	1	1	1	1	1	21.5	0.02	12.1
1	0	0	100	4.6	2	72	32.3	117.5	63	198	51	147	14.1	6.1	74.3	2	1	1	1	1	1	1	14.6	0.04	25.7
1	0	0	100	4.62	2	66	21.8	108.5	65.5	171	51	120	12	5.5	77.5	2	1	0	1	1	1	1	27.5	0.01	54.4
1	0	0	100	4.67	2	67	26.3	119	72.5	172	51	121	12.4	5.7	76.9	2	1	1	0	0	1	1	20.4	0.04	16.6
1	0	0	100	4.69	2	66	30.4	156.5	92	191	51	140	14.1	5.4	77.5	1	1	1	0	1	1	1	18.4	0.06	27
1	0	0	100	6.86	2	70	24.3	110	63	214	51	163	12.9	5.6	71.6	1	1	1	1	1	1	1	28.5	0.04	19.1
1	0	0	100	6.87	2	71	24.6	109.5	72	185	51	134	14.2	6.7	74.8	2	1	0	1	1	0	1	18.3	0.06	10.9
1	0	0	100	5.04	2	67	27.7	150.5	84	217	51	166	13.3	6.4	73.1	2	1	1	0	0	1	1	6	0.08	34.4
1	0	0	100	5.04	2	68	27.4	103	55.5	163	51	112	13.9	5.6	76.4	2	1	1	1	1	1	1	26.3	0.05	20.7
1	0	0	100	5.05	2	68	23.1	122.5	71.5	154	51	103	13.3	5.3	81.1	2	1	1	1	1	1	1	12	0.03	41.2
1	1	0	100	4.59	2	76	22.8	112.5	65.5	295	51	244	13.5	5.4	58.4	2	1	1	1	0	1	1	11	0.02	7.33
1	1	0	100	7.04	2	65	24.3	116.5	70	178	51	127	12.8	5.6	74.2	2	1	1	1	1	1	1	6	0.03	10.4
1	1	0	100	6.96	2	71	22.5	130	64.5	180	51	129	12.4	5.9	71.1	2	1	1	1	1	1	1	6.6	0.06	0.05
1	1	0	100	6.99	2	82	25.5	129	65	179	51	128	12.3	5.1	69.2	1	1	1	0	1	1	1	11.6	0.02	28.7
1	0	0	100	4.58	1	71	27.6	151	86.5	196	51	145	15.4	6.9	73.1	2	1	1	0	1	0	1	153.9	0.1	84.4
1	0	0	100	5.15	1	66	23.7	124	73	198	51	147	13.9	6.3	75.7	2	1	1	0	1	0	1	18.5	0.2	31.5
1	0	0	100	6.84	1	79	20.5	96.5	63	174	51	123	14	6	69.1	2	0	1	1	1	1	1	5.1	0.1	25.8
1	0	0	100	5.06	1	77	30.7	155	70.5	159	51	108	12.7	6.5	52.4	1	1	1	0	1	0	1	43.4	0.11	118
1	0	0	100	5.07	1	69	26.1	152.5	79.5	181	51	130	13.2	5.8	70.6	2	1	0	0	1	1	0	161.2	0.17	294
1	0	0	100	4.59	1	74	25.8	114	64	182	51	131	14.7	5.7	71.5	1	1	0	1	1	1	1	107.1	0.54	19.1
1	0	0	100	4.61	1	70	28.5	137.5	77.5	219	51	168	13.7	5.5	73.6	1	1	0	0	0	1	1	674.9	0.18	42.2
1	0	0	100	6.87	1	65	27.5	183	99.5	152	51	101	16.7	4.8	63.9	2	1	1	0	1	1	1	94.2	0.21	125
1	0	0	100	6.86	1	72	23.5	120	72	200	51	149	15.9	5.3	69.1	2	0	0	1	0	1	1	22.9	0.06	7.57
1	0	0	100	7.21	1	76	23.6	128	75	164	51	113	15	6.4	67.2	2	1	1	1	1	1	1	10.9	0.05	41.8
1	0	0	100	6.86	1	71	28.5	147.5	96.5	206	51	155	14.9	6.9	69.6	2	0	0	0	1	0	1	12.9	0.07	53.7
1	0	0	100	4.63	1	73	19.8	136.5	80	185	51	134	13.6	5.4	76.1	2	1	1	1	1	1	1	23	0.01	14.6
1	0	0	100	4.54	1	72	24.3	133	70	163	51	112	15	5.4	76.7	2	1	0	1	1	1	1	4.9	0.04	35.9
1	0	0	100	5.37	1	76	22.5	117.5	58.5	163	51	112	13.2	5.3	74.5	2	0	0	1	1	1	1	9.7	0.02	32.6
1	0	0	100	6.86	1	74	17.2	138	74	154	51	103	14.1	5.6	75.6	2	1	1	1	1	1	1	15.1	0.01	33.1
1	1	0	100	6.45	1	70	21.3	120	65	199	51	148	16	6.4	82.8	2	0	0	1	1	0	1	11.9	0.13	24
1	1	0	100	7	1	70	26.6	145	90.5	157	51	106	15.3	5.4	77.7	2	1	1	0	1	1	1	13	0.04	8.98
1	0	0	100	7.08	2	67	32.4	121	64.5	197	51	146	13	5.9	81.7	2	1	1	0	1	1	1	44	0.19	5.8
1	0	0	100	7.04	2	65	23.1	112	71	190	51	139	12.9	6.1	78.0	2	1	1	1	1	1	1	5.1	0.1	7.36
1	0	0	100	4.63	2	72	28.6	133	77.5	236	51	185	11.8	5.4	60.1	2	1	1	0	0	1	1	17.7	0.38	51.5
1	0	0	100	1.23	2	79	33.4	146	88	239	51	188	10.7	5.6	49.6	2	1	1	0	0	1	0	56.7	0.19	125
1	0	0	100	4.61	2	74	21.5	125.5	73	165	51	114	12.9	5.3	73.2	2	1	1	0	1	1	1	37.3	0.39	33.4
1	0	0	100	4.61	2	70	27.5	149.5	77	259	51	208	13.1	6.3	60.9	2	1	1	0	0	1	1	52.7	0.12	7.14
1	0	0	100	4.62	2	74	29	105	57.5	220	51	169	12.6	5.4	59.2	2	1	1	0	0	1	1	53.8	0.15	8.02
1	0	0	100	4.69	2	70	29.9	99	57	191	51	140	13.7	5.4	75.3	2	1	1	1	1	1	1	7.4	0.25	4.05
1	0	0	100	6.86	2	70	23.2	107.5	61	227	51	176	11.3	5.3	75.3	2	1	1	1	0	1	1	10.3	0.25	30.9
1	0	0	100	6.88	2	65	26.9	117	64.5	163	51	112	12	5.3	74.2	2	1	1	1	1	1	1	12.5	0.02	42.1
1	0	0	100	7	2	68	20.4	117.5	66.5	132	51	81	13.7	4.6	81.1	1	1	0	1	1	1	1	26.6	0.02	29.6
1	0	0	100	7.02	2	68	24.8	112.5	67.5	168	51	117	12.2	5.1	76.4	1	1	0	1	1	1	1	14.3	0.05	15.6
1	0	0	100	5.08	2	69	32.1	164	92.5	226	51	175	14.4	5.5	72.1	2	1	1	0	0	1	1	10.6	0.07	13.1
1	0	0	100	6.97	2	68	23.6	119	56	165	51	114	13.4	8.7	76.4	2	1	1	0	1	0	1	14.9	0.01	20.6
1	0	0	100	4.62	2	74	27.1	143.5	75.5	190	51	139	13.8	5.9	73.2	2	1	1	0	1	1	1	46.9	0.05	115

1	0	0	100	4.62	2	78	30.5	119	73.5	218	51	167	14	5.7	71.2	1	1	1	1	1	1	1	13.6	0.03	49.7
1	0	0	100	4.64	2	65	25.8	149	81.5	201	51	150	13.5	5.5	74.2	2	1	1	0	1	1	1	6.6	0.08	45.5
1	0	0	100	6.98	2	70	25.4	180	105	211	51	160	14.8	6	75.3	1	1	1	0	1	1	1	166.8	0.07	6.79
1	0	0	100	6.75	2	73	24.1	105	71.5	173	51	122	14.3	6	73.8	2	1	0	0	0	1	1	9.7	0.03	28.9
1	0	0	100	3.24	2	79	24.7	131.5	70.5	188	51	137	14.1	5.7	67.2	1	1	1	0	1	1	1	30.8	0.04	17.4
1	0	0	100	6.84	2	74	20.6	156	88.5	218	51	167	12.4	5.8	69.6	1	1	1	0	1	1	1	18.6	0.04	10.4
1	0	0	100	5.37	2	73	22.3	130	75.5	199	51	148	11.3	6	70.1	1	1	1	0	1	1	1	12.2	0.01	16.5
1	0	0	100	5.37	2	69	20.9	153.5	81	184	51	133	12.2	6	80.5	2	1	1	0	0	1	1	23.9	0.01	30.8
1	0	0	100	6.45	2	72	29.7	109	68	186	51	135	12.9	6.1	74.3	1	1	1	0	1	1	1	24.9	0.03	8.89
1	0	0	100	5.04	2	73	25.9	151.5	92.5	193	51	142	13	5.7	78.3	2	1	1	0	1	1	1	5.1	0.09	0.69
1	1	0	100	7.08	2	71	29.1	120	66	224	51	173	12.7	5.5	85.5	2	1	1	1	0	1	1	109	0.36	16.7
1	1	0	100	7.2	2	76	28.9	128.5	66	219	51	168	12.3	5.2	68.6	1	1	1	0	1	1	1	111	0.05	36.7
1	1	0	100	2.38	2	73	21.2	153	74.5	213	51	162	12.6	5.5	73.8	1	1	1	0	1	1	1	13	0.01	26.9
1	0	0	100	4.16	1	80	22.1	136.5	71	199	51	148	15	5.6	72.5	1	1	0	1	1	1	1	6.4	0.03	3.43
1	0	0	100	5.05	2	83	25.1	108.5	62.5	205	51	154	13.1	5.5	65.3	1	1	1	0	1	1	1	8.6	0.02	100
1	0	1	0.2	0.2	1	71	21.9	129.5	81	146	51	95	13.8	5.1	44.6	2	0	0	1	1	1	0	52.6	0.05	1280
1	0	1	4.6	4.6	1	68	24.2	170.5	81.5	208	51	157	16.1	5.6	78.8	2	0	0	0	1	1	1	199.5	0.04	8.31
1	1	1	2.1	2.1	1	72	23.4	131.5	64.5	221	51	170	16.7	5.2	48.8	2	1	1	1	0	1	1	7.3	0.11	29.7
1	0	1	3.7	3.7	2	77	24.5	158	76	229	51	178	13.5	5.7	71.7	1	1	1	0	0	1	1	46.2	0.04	62.5
1	0	1	1.4	1.4	2	67	25.9	131.5	69	209	51	158	12.1	7	73.1	2	1	1	0	1	0	1	22.8	0.03	43.2
1	0	1	2.8	2.8	2	77	20.6	112.5	61	156	51	105	12.8	5.7	71.7	1	1	1	1	1	1	1	22.3	0.05	35.1
1	0	1	1.7	1.7	2	66	24.9	136.5	58.5	181	51	130	13.4	5.1	82.3	1	1	0	1	1	1	1	44.3	0.07	86.7
1	0	1	6.8	6.8	2	73	27	117	71.5	234	51	183	14.8	5.9	70.1	2	1	1	1	0	1	1	11.6	0.13	6.86
1	1	1	1.8	1.8	2	78	22.6	145	81.5	233	51	182	13.4	5	67.7	2	0	1	0	0	1	1	43.2	0.04	71.3
1	0	0	100	6.87	1	67	23	156.5	95	157	52	105	15.9	5.3	75.2	2	1	0	0	1	1	0	11.4	0.17	173
1	0	0	100	6.87	1	67	25.6	120.5	67	177	52	125	13.6	5.4	63.0	2	1	0	0	1	1	1	7	0.38	7.48
1	0	0	100	6.88	1	66	19.9	112.5	69.5	193	52	141	14.2	5.4	75.7	2	0	0	1	1	1	1	7.6	0.39	9.36
1	0	0	100	7.21	1	75	26	130.5	76.5	221	52	169	13.4	7.6	71.0	2	1	0	0	0	0	1	9.2	0.16	10.9
1	0	0	100	7.21	1	69	22.7	131.5	87.5	213	52	161	14.8	5.8	78.3	2	1	0	0	1	1	1	125	0.1	9.03
1	0	0	100	6.98	1	71	24.5	127	83.5	208	52	156	14.5	5.4	73.1	2	0	1	1	1	1	1	147.7	0.31	0.05
1	0	0	100	7	1	66	20.6	98	62	213	52	161	15.3	5.6	72.1	2	0	1	1	1	1	1	6.3	0.18	12.5
1	0	0	100	6.87	1	73	22.8	136	80	220	52	168	17.7	6.4	68.6	2	0	0	1	0	1	1	175.4	0.28	9.54
1	0	0	100	4.59	1	71	25.7	151	85.5	233	52	181	16.7	6.3	69.6	2	1	0	0	0	1	1	2.4	0.21	85.6
1	0	0	100	4.59	1	74	25.3	159	97.5	170	52	118	16.4	5.5	75.6	2	1	1	0	1	1	1	15.9	0.3	18.9
1	0	0	100	5.36	1	76	24.5	138.5	87.5	164	52	112	14.3	6.1	70.5	1	1	0	0	1	1	1	36.2	0.32	42.6
1	0	0	100	5.05	1	69	28.5	118	82	192	52	140	15.4	5.6	74.1	2	1	0	0	1	0	1	8.8	0.1	7.67
1	0	0	100	7	1	67	24.6	153.5	85	174	52	122	14	5.4	79.4	2	1	0	0	1	1	1	8.5	0.04	16.9
1	0	0	100	7.07	1	66	25.3	137.5	81.5	201	52	149	15	5	75.7	2	0	0	1	1	1	1	4.7	0.09	45.4
1	0	0	100	4.58	1	69	22.1	103	63	204	52	152	13.9	6.1	74.1	2	1	1	1	1	1	1	5.6	0.01	17.2
1	0	0	100	4.59	1	76	26.1	122	71	202	52	150	15.7	5.7	67.2	2	1	1	1	1	1	1	28	0.01	3.72
1	0	0	100	4.59	1	75	20.3	104.5	70.5	161	52	109	15.4	4.6	67.7	2	1	0	1	1	1	1	7.7	0.02	7.33
1	0	0	100	4.59	1	68	23.6	117	66.5	201	52	149	14.9	5.5	74.6	2	1	1	1	1	1	1	8.7	0.06	19.7
1	0	0	100	7.23	1	67	27.5	148.5	91	179	52	127	13.8	5.1	63.0	2	1	0	0	1	1	1	10.4	0.03	116
1	0	0	100	5.15	1	66	27	186	89.5	173	52	121	14.5	5.6	79.9	2	1	1	0	1	1	1	17.5	0.05	18.1
1	0	0	100	5.16	1	65	23.9	162	78	180	52	128	14.3	5.5	76.2	2	1	0	0	1	1	0	37.7	0.02	122
1	0	0	100	5.16	1	70	25.4	142.5	81	255	52	203	15.2	6.5	61.7	2	1	0	0	0	0	1	9.9	0.08	39.1
1	0	0	100	6.84	1	66	20.6	99.5	67	197	52	145	14.7	4.9	75.7	2	1	1	1	1	1	1	4.6	0.08	14.3
1	0	0	100	6.85	1	68	24.7	123	75.5	166	52	114	15.5	4.9	55.8	2	1	0	1	1	1	1	17.8	0.06	74.7
1	0	0	100	7.03	1	76	26.8	115.5	73.5	220	52	168	16.4	5.2	67.2	1	1	1	1	0	1	1	6.4	0.08	1.39
1	0	0	100	7.04	1	76	24.8	135	73	208	52	156	14.8	5.6	70.5	2	0	1	1	1	1	1	9.4	0.07	0.05
1	0	0	100	5.05	1	66	20.9	113	74	131	52	79	15.2	5.3	79.9	2	1	0	1	1	1	1	7.5	0.02	36.3

1	0	0	100	5.06	1	71	25.5	127.5	68	178	52	126	14	7	82.2	2	1	0	1	1	0	1	4.1	0.03	11.4
1	0	0	100	4.62	1	65	20.5	113	79	235	52	183	15.8	5.3	76.2	2	1	1	1	0	1	1	9.7	0.02	9.81
1	0	0	100	5.06	1	71	18.2	109.5	64	166	52	114	13.1	5.3	77.2	2	1	1	1	1	1	1	14.6	0.03	73.1
1	0	0	100	6.96	1	65	24.7	134	75.5	199	52	147	14.3	4.8	85.8	1	1	0	0	1	1	1	7.1	0.04	7.19
1	0	0	100	6.8	1	69	21.8	126.5	90	239	52	187	14.1	5.3	70.6	2	1	0	0	0	1	1	6.4	0.03	12.3
1	0	0	100	6.83	1	68	25.4	115	72.5	190	52	138	14	4.8	74.6	2	1	0	1	1	1	1	17.2	0.03	28.8
1	0	0	100	4.62	1	71	23	195	89.5	197	52	145	15.2	5.4	73.1	2	1	0	0	1	1	1	19.6	0.05	33.7
1	0	0	100	4.67	1	68	22.4	140	84	215	52	163	15.1	5.3	74.6	2	1	0	0	1	1	1	21	0.05	5.79
1	0	0	100	4.67	1	72	30.4	171	107.5	238	52	186	17.2	5.5	72.6	1	1	0	0	0	1	0	306.4	0.05	164
1	0	0	100	5.36	1	66	26.3	128	76	202	52	150	14.9	5.3	75.7	2	1	0	1	1	1	1	11.4	0.04	23.1
1	0	0	100	5.05	1	71	27.3	132	73	245	52	193	16.8	5.6	61.3	2	1	0	1	0	1	1	5.5	0.04	36.9
1	0	0	100	6.85	1	68	21.2	128	81.5	167	52	115	13.3	5.8	74.6	2	1	0	1	1	1	1	5.6	0.03	13.4
1	0	0	100	6.85	1	69	24.1	143	76	215	52	163	14	5.5	70.6	2	1	0	0	1	1	1	21.6	0.08	90.3
1	0	0	100	6.87	1	71	25.1	125	78.5	216	52	164	16.3	9.1	73.1	2	1	0	1	1	0	1	5.5	0.04	9.19
1	0	0	100	6.87	1	70	23.6	128	73.5	221	52	169	14.6	5.7	73.6	2	1	0	1	0	1	1	40.3	0.05	12.3
1	0	0	100	5.04	1	66	27.5	138.5	87	219	52	167	15.9	5.8	85.2	2	1	0	1	1	1	1	7.7	0.01	28.2
1	1	0	100	4.64	1	77	20.8	110.5	69	193	52	141	13.9	5.3	39.1	2	1	0	1	1	1	1	4.3	0.27	45.5
1	1	0	100	6.98	1	77	21.8	122.5	72.5	175	52	123	14.2	5.2	66.7	2	1	1	1	1	1	1	12.1	0.05	18.6
1	1	0	100	6.83	1	75	22.7	144	87.5	169	52	117	15.3	5.3	67.7	2	1	1	0	1	1	1	9.4	0.03	9.12
1	1	0	100	6.44	1	74	20.7	131	86.5	104	52	52	13.5	5.3	53.5	2	1	1	1	1	1	1	9.3	0.03	53.6
1	1	0	100	5.06	1	75	20.5	137.5	78.5	168	52	116	13.9	4.7	71.0	2	1	0	1	1	1	1	5.1	0.02	21.7
1	0	0	100	4.58	2	74	24.5	129	76	251	52	199	14.5	5.3	69.6	2	1	1	1	0	1	1	15.6	0.1	3.81
1	0	0	100	4.58	2	71	26.3	127.5	74	213	52	161	13.1	6.3	71.1	2	1	1	1	1	1	1	12.6	0.1	17.8
1	0	0	100	4.59	2	67	29.1	138	94.5	219	52	167	13.6	5.9	81.7	2	1	1	0	1	1	1	78.3	0.33	27.7
1	0	0	100	7.01	2	69	26.8	107	60.5	195	52	143	11.8	5.5	72.1	2	1	1	1	1	1	1	5.3	0.17	10.7
1	0	0	100	5.05	2	71	23.2	141.5	82	148	52	96	12.5	5.7	74.8	1	1	1	0	1	1	1	18.2	0.11	62.7
1	0	0	100	5.06	2	67	25.3	99	56.5	203	52	151	12.6	5.2	73.1	2	1	1	1	1	1	1	10.4	0.12	25.8
1	0	0	100	4.63	2	72	23.6	157	85	226	52	174	15.8	6.2	70.6	2	1	1	0	0	1	1	134.4	0.46	7.69
1	0	0	100	4.64	2	74	30.4	165.5	87.5	276	52	224	13.8	6.1	73.2	2	1	1	0	0	1	1	30	0.18	16.9
1	0	0	100	5.88	2	65	34.8	172.5	89.5	214	52	162	13.8	5.9	74.2	2	1	1	0	1	1	1	7.4	0.28	80.9
1	0	0	100	4.6	2	65	27.5	143.5	81	201	52	149	13.1	5.2	78.0	2	1	1	0	1	1	1	19.4	0.14	13.5
1	0	0	100	4.68	2	69	27.4	125.5	75	227	52	175	13.6	5	75.9	1	1	0	0	0	1	1	26.1	1.48	9.36
1	0	0	100	4.68	2	71	30.2	128.5	71.5	195	52	143	13.8	7.4	79.4	1	1	1	0	1	0	1	522.4	0.14	20.2
1	0	0	100	5.05	2	71	26.1	157	89	180	52	128	13.7	5.3	71.1	2	1	1	0	1	1	1	98.7	0.29	73
1	0	0	100	6.45	2	77	28.9	136	78	186	52	134	14.8	5.1	58.0	2	1	1	1	1	1	1	21	0.1	21
1	0	0	100	6.87	2	70	27.4	134.5	81	180	52	128	12.6	5.3	75.3	2	1	1	1	1	1	1	19	0.12	71.8
1	0	0	100	5.04	2	77	21.8	151.5	91.5	191	52	139	13	5	71.7	1	1	1	0	1	1	1	9.1	0.1	25.4
1	0	0	100	4.58	2	69	27.4	146	82.5	204	52	152	12.4	5.8	80.5	1	1	1	0	1	1	1	16.3	0.04	14
1	0	0	100	4.58	2	72	28.2	170	88.5	188	52	136	14.4	5.9	78.9	2	1	1	0	0	1	1	57.1	0.01	10.2
1	0	0	100	4.59	2	78	24.1	116.5	59	202	52	150	12.8	6.2	67.7	1	1	1	1	1	1	1	22.6	0.01	70.1
1	0	0	100	6.86	2	72	23.1	160.5	90.5	210	52	158	12.1	5	74.3	2	1	1	0	1	1	1	20.5	0.04	38.7
1	0	0	100	6.87	2	70	28.8	125	69	179	52	127	14.5	5.2	75.3	2	1	1	1	1	1	1	35.4	0.03	36.1
1	0	0	100	6.87	2	66	33.5	146.5	82.5	173	52	121	14.2	6	73.6	2	1	1	0	1	1	1	7.5	0.07	22.8
1	0	0	100	6.84	2	68	29.3	136	77	184	52	132	13.7	7.1	76.4	2	1	0	1	0	0	1	16.5	0.07	20.2
1	0	0	100	6.86	2	66	20.3	109.5	72.5	255	52	203	13	5.1	62.7	2	1	1	0	0	1	1	8.8	0.07	53.1
1	0	0	100	6.86	2	72	21	101.5	57	194	52	142	13.3	5.3	52.1	2	1	1	0	1	1	1	58.1	0.07	40.7
1	0	0	100	7.03	2	73	24.4	141.5	86.5	203	52	151	13.5	5.1	59.7	1	1	1	0	1	1	1	20.6	0.04	12.3
1	0	0	100	7.04	2	69	20.3	130.5	72	216	52	164	11.4	6	80.5	2	1	1	1	1	1	1	7.6	0.01	0.05
1	0	0	100	5.06	2	70	21.1	134.5	70	212	52	160	13.3	5.6	80.0	2	1	1	1	1	1	1	9.2	0.08	79.8
1	0	0	100	5.07	2	69	25.8	148	81.5	192	52	140	12.6	7.1	80.5	2	1	1	0	1	0	1	11.2	0.02	87.7
1	0	0	100	7	2	67	24.7	164	93	183	52	131	12.5	5.3	73.1	1	1	1	0	1	1	1	15.8	0.06	6.32

1	0	0	100	4.64	2	75	24.5	112.5	71.5	212	52	160	13.9	5.5	72.7	2	1	0	0	1	1	1	20.6	0.04	6.11
1	0	0	100	4.64	2	70	24.1	135	92	231	52	179	13.8	5.4	71.6	2	1	0	0	0	1	1	49.4	0.03	12.9
1	0	0	100	6.95	2	73	23	123.5	60.5	240	52	188	12.2	5.3	59.7	2	1	1	1	0	1	1	35.4	0.01	23
1	0	0	100	6.96	2	67	30.6	106.5	66	212	52	160	14.5	5.2	73.1	1	1	1	0	1	1	1	16.1	0.08	13
1	0	0	100	6.8	2	75	21.6	122	66.5	150	52	98	14.3	5.6	58.8	2	1	0	1	1	1	1	17.9	0.01	170
1	0	0	100	4.59	2	66	28.1	128	82.5	225	52	173	13.1	5.3	82.3	2	1	0	0	0	1	1	5.5	0.08	25.7
1	0	0	100	4.62	2	67	19.7	106.5	58	205	52	153	12.4	5.2	62.2	2	1	1	1	1	1	1	13.6	0.02	41.1
1	0	0	100	4.62	2	68	21.1	149	82	232	52	180	14.2	5.5	72.6	2	1	1	0	0	1	1	16.5	0.02	8.36
1	0	0	100	4.62	2	65	26.9	150	104	281	52	229	13.9	5.6	74.2	2	1	1	0	0	1	1	43.6	0.02	7.82
1	0	0	100	4.69	2	65	20.5	132	67.5	142	52	90	12.3	7	48.2	2	1	1	0	1	0	1	7.4	0.06	4.59
1	0	0	100	6.97	2	71	20.6	130.5	83	232	52	180	12.3	4.9	74.8	2	1	1	1	0	1	1	8.9	0.06	19.3
1	0	0	100	5.36	2	65	22.6	109.5	63	194	52	142	12.4	5.6	78.0	2	1	1	1	1	1	1	36	0.02	16.1
1	0	0	100	5.37	2	70	30.6	121.5	72.5	201	52	149	12.2	5.9	75.3	2	1	1	1	1	1	1	24.2	0.05	23.5
1	0	0	100	5.37	2	65	24.7	108	67	219	52	167	13	5.5	63.1	2	1	0	1	1	1	1	7.2	0.01	40.8
1	0	0	100	5.06	2	67	23.1	129	69.5	209	52	157	13.2	5.6	73.1	2	1	1	1	1	1	1	6.1	0.05	23.2
1	0	0	100	6.87	2	70	19.3	119	75.5	193	52	141	11.3	7	80.0	2	1	1	1	1	0	1	13.1	0.03	14.1
1	1	0	100	7.07	2	75	29.2	121.5	74	153	52	101	11.3	6.6	72.7	1	1	1	0	1	0	1	30.4	1.66	33.8
1	1	0	100	5.15	2	77	33.2	119.5	65	223	52	171	13.3	7.4	50.3	1	1	1	0	0	0	1	8.8	0.1	24.4
1	0	0	100	5.05	1	80	22.9	164.5	96	204	52	152	13.4	5.5	65.4	2	1	1	0	1	1	1	126.2	0.4	59.9
1	0	0	100	5.08	1	80	25	142.5	82.5	165	52	113	12.6	5.9	68.6	2	1	0	0	1	1	1	34	0.24	24.4
1	0	0	100	5.04	2	82	17.4	101	55.5	150	52	98	10.8	5.7	65.8	1	1	1	1	1	1	1	28.5	0.3	26.2
1	0	0	100	6.87	2	80	24.2	131	62.5	180	52	128	13.4	5.1	70.2	1	1	1	1	1	1	1	5.9	0.06	35.3
1	0	0	100	4.64	1	76	22.5	144.5	78	162	52	110	15.4	5.2	70.5	2	0	0	0	1	1	1	28.4	0.14	47.5
1	0	0	100	4.7	1	74	21.9	108	65.5	171	52	119	14.2	5.2	80.5	2	0	0	1	1	1	1	19.2	0.26	21.8
1	0	0	100	6.84	1	70	23.2	140	81	218	52	166	14.7	5.4	77.7	2	1	1	0	1	1	1	24.6	0.09	13.8
1	0	0	100	6.98	1	78	23.1	122.5	80	189	52	137	12.9	5.8	69.6	2	1	1	1	1	1	1	3	0.03	21.9
1	0	0	100	4.62	1	77	19.2	151	76	207	52	155	14.8	5.6	74.0	2	0	0	0	1	1	1	14.8	0.04	74.1
1	1	0	100	7	1	72	25.2	114	71.5	277	52	225	14.7	5.7	54.2	2	1	1	1	0	1	1	9.3	0.23	286
1	1	0	100	1.6	1	75	19.9	113	66	213	52	161	14.6	5.3	53.1	2	1	0	0	1	1	0	50.5	0.13	183
1	1	0	100	4.8	1	73	22.5	131.5	73	162	52	110	14.2	6.6	76.1	1	0	1	0	1	0	1	142.1	0.04	31.5
1	0	0	100	6.88	2	67	25.3	128.5	73	212	52	160	12.3	5.7	81.7	2	1	1	0	1	1	1	25.3	0.19	6.71
1	0	0	100	0	2	72	23.9	130.5	70.5	224	52	172	12.6	5.4	74.3	2	1	1	0	0	1	1	31.3	0.2	51
1	0	0	100	6.96	2	70	21.7	129.5	72	217	52	165	13.2	7.1	80.0	1	1	1	1	1	0	1	25	0.39	48
1	0	0	100	6.98	2	70	23	143.5	75.5	213	52	161	13	5.3	75.3	2	1	1	0	1	1	1	57.1	0.14	88.8
1	0	0	100	7.06	2	69	23.6	144.5	85.5	222	52	170	12.3	5.8	80.5	1	1	0	0	0	1	1	22.9	0.05	12.4
1	0	0	100	7.06	2	69	27.3	108.5	61.5	216	52	164	12.8	5.2	61.4	2	1	1	1	1	1	1	14.9	0.04	24
1	0	0	100	0.08	2	76	23.3	110.5	58.5	232	52	180	12.6	4.9	58.4	2	1	1	0	0	1	1	16.7	0.06	59
1	0	0	100	7.08	2	74	25.2	145.5	82	216	52	164	13.1	5.2	83.7	2	1	1	0	1	1	1	165.4	0.02	9.54
1	0	0	100	7.08	2	70	24.4	135	80.5	228	52	176	13.9	5.4	80.0	2	1	1	1	0	1	1	38.6	0.03	8.19
1	0	0	100	4.59	2	70	24.7	123.5	70	208	52	156	12.4	5.1	75.3	2	1	1	1	1	1	1	195	0.01	29.5
1	0	0	100	6.74	2	72	17.8	99	60	197	52	145	8.7	4.5	74.3	2	1	1	1	1	1	1	18.5	0.01	27.7
1	0	0	100	6.84	2	70	24.9	137.5	72.5	155	52	103	13.7	5.7	75.3	2	1	1	0	1	1	1	19.4	0.05	27.2
1	0	0	100	6.85	2	73	24.2	110	59	237	52	185	12.7	5.3	70.1	2	1	1	0	0	1	1	38.6	0.04	44.7
1	0	0	100	7	2	71	23.7	117.5	65.5	152	52	100	13.6	5.3	79.4	2	1	1	1	1	1	1	10.7	0.06	20.1
1	0	0	100	7.01	2	71	28.4	139.5	84.5	209	52	157	13.4	5.9	74.8	1	1	1	0	1	1	1	99.2	0.08	24.2
1	0	0	100	6.87	2	70	20.2	114.5	64.5	221	52	169	14.8	5.3	75.3	2	1	1	1	0	1	1	29	0.02	12.4
1	0	0	100	6.95	2	69	24.5	131.5	70	145	52	93	11.4	5.2	86.7	1	1	1	1	1	1	1	21	0.09	99.8
1	0	0	100	6.96	2	69	25.8	100	67	200	52	148	12.7	4.4	75.9	1	1	1	1	0	1	1	72.1	0.08	14.3
1	0	0	100	6.8	2	75	25.3	118.5	76	167	52	115	13.3	5.6	69.1	1	1	1	1	1	1	1	18.8	0.06	14.9
1	0	0	100	6.84	2	70	26	106.5	62	188	52	136	11.5	5.4	75.3	2	1	1	1	1	1	1	5.5	0.02	12.2
1	0	0	100	4.61	2	75	27.2	148.5	89.5	256	52	204	13.8	5.6	69.1	1	1	1	0	0	1	1	35.3	0.03	17.1

1	0	0	100	5.04	2	67	26.1	128	69	190	52	138	12.9	5.4	76.9	2	1	1	1	1	1	1	20.7	0.08	24.3
1	0	0	100	6.44	2	73	28.1	124.5	78	205	52	153	14	5.2	73.8	2	1	1	1	0	1	1	11.9	0.09	5.41
1	0	0	100	4.69	2	65	27.5	149.5	80	198	52	146	13.7	5.5	82.8	2	1	1	0	1	1	1	31.7	0.04	24.2
1	0	0	100	5.37	2	75	25.9	104	61.5	167	52	115	12.4	5.7	58.8	1	1	1	1	1	1	1	23.1	0.06	13.5
1	0	0	100	5.07	2	66	26.6	160	87	218	52	166	12.5	5.6	73.6	2	1	1	0	1	1	1	4.5	0.02	30.2
1	0	0	100	6.86	2	74	23.3	107.5	72.5	206	52	154	13.5	5.2	59.2	2	1	1	1	1	1	1	10.6	0.04	27.5
1	0	0	100	6.87	2	65	24.4	112.5	70	142	52	90	12	4.1	78.0	2	1	0	1	1	1	1	11.5	0.03	11.9
1	0	0	100	5.04	2	67	24	115	69	168	52	116	12.9	5.4	76.9	2	1	1	0	1	1	1	10.8	0.01	16.8
1	1	0	100	4.7	2	73	20.2	144	79	163	52	111	10.9	5.8	73.8	1	1	1	0	1	1	1	141.4	0.24	16.3
1	1	0	100	7.2	2	73	22.3	114	70	194	52	142	13	7.4	59.7	1	1	1	1	1	0	1	80.3	0.05	16.7
1	1	0	100	3.46	2	70	29	163.5	77.5	210	52	158	13	7	75.3	2	1	1	0	1	0	1	88.1	0.05	23.8
1	1	0	100	5.3	2	77	20.7	128	66.5	179	52	127	10.3	4.8	71.7	2	1	1	1	1	1	1	43.5	0.01	59.5
1	1	0	100	4.64	2	73	28.1	142.5	85	202	52	150	14.2	5.6	59.7	2	1	1	0	1	1	1	6.3	0.07	11.3
1	0	0	100	7.05	1	80	23.1	156.5	92.5	194	52	142	13.4	5.5	65.4	2	1	1	0	1	1	1	13.6	0.02	17
1	0	0	100	4.64	1	80	22.4	150.5	82	148	52	96	12.6	4.8	68.6	2	1	1	0	1	1	1	23.2	0.08	31.3
1	0	1	0.7	0.7	1	71	24.3	124.5	83	186	52	134	17.7	5.5	61.3	2	0	0	1	1	1	1	122.5	0.3	13.7
1	0	1	6.1	6.1	1	65	25.1	155	90.5	249	52	197	17.8	11.1	72.6	2	1	0	0	0	0	1	24.6	0.09	18.7
1	0	1	0.7	0.7	2	65	23.1	104.5	74.5	197	52	145	15.2	6.2	82.8	2	1	1	0	1	1	1	22.9	0.06	24.7
1	0	1	6.9	6.9	2	69	24	124.5	70.5	238	52	186	15.3	5.6	75.9	1	1	1	0	0	1	1	11.1	0.07	3.61
1	1	1	4	4	2	75	26.1	111	76	192	52	140	14.4	5.6	83.1	1	1	1	1	1	1	1	58.2	0.13	3.97
1	0	1	3	3	1	70	25.7	120.5	69.5	211	52	159	14.9	6.4	77.7	2	0	0	0	1	1	1	9	0.05	60.8
1	0	1	2.1	2.1	2	75	25.2	104.5	78.5	211	52	159	12	5.6	69.1	1	1	1	1	1	1	1	17.1	0.07	40.5
1	1	1	1.5	1.5	2	76	21.3	151.5	69.5	175	52	123	13.6	5.9	50.7	2	1	1	0	1	1	0	48.3	0.4	178
1	0	0	100	6.86	1	75	27	143	68.5	203	53	150	12.8	5.1	59.6	2	1	0	0	1	1	1	15.4	0.14	58.3
1	0	0	100	6.87	1	68	21.9	131.5	94.5	187	53	134	12.8	5.2	71.1	2	1	0	0	1	1	1	4.3	0.12	17.4
1	0	0	100	6.86	1	72	22	120	68.5	222	53	169	14.3	5.4	69.1	2	1	0	1	0	1	1	3.2	0.17	14.6
1	0	0	100	0.97	1	69	24.6	127.5	75	180	53	127	13.8	4.8	83.4	1	1	1	1	1	1	1	90.1	0.47	17.4
1	0	0	100	3.54	1	75	27.9	140	73.5	179	53	126	12.4	6.4	43.4	2	1	1	0	1	1	1	8.9	0.43	27.7
1	0	0	100	4.59	1	68	22.2	135	70	144	53	91	12.1	5.8	78.8	2	1	0	0	1	1	1	536.4	0.36	68
1	0	0	100	4.62	1	68	24.6	151.5	82.5	215	53	162	15.1	5.7	55.8	2	1	0	0	1	1	1	4.4	0.11	30.1
1	0	0	100	7.07	1	70	20.6	133	77	154	53	101	14.4	5.1	82.8	2	1	0	1	1	1	1	8.1	0.02	0.05
1	0	0	100	7.08	1	70	24.7	147	88.5	212	53	159	14.2	5.6	61.7	2	1	1	0	0	1	1	10.4	0.03	7.05
1	0	0	100	4.58	1	77	24.8	143.5	91	207	53	154	15.5	6.4	52.4	2	1	0	0	1	1	1	81.1	0.03	11.2
1	0	0	100	6.86	1	66	23.2	123	74	218	53	165	14.3	4.9	75.7	2	1	1	1	1	1	1	5.5	0.04	18.3
1	0	0	100	6.86	1	74	22.9	156	80.5	201	53	148	13.9	4.7	71.5	2	0	0	0	1	1	1	10.3	0.07	14.6
1	0	0	100	4.81	1	69	26.7	129.5	83	191	53	138	15.4	4.5	74.1	2	1	0	0	1	1	1	14.3	0.06	4.56
1	0	0	100	3.82	1	75	25.2	140.5	83	180	53	127	15.4	5.2	71.0	2	0	1	0	1	1	1	7.4	0.02	20.2
1	0	0	100	6.84	1	74	22.1	135.5	78	227	53	174	15	6.1	48.1	2	1	1	1	0	0	1	25.1	0.06	8.56
1	0	0	100	6.84	1	68	22.8	105.5	64	225	53	172	15.8	5.8	78.8	2	0	1	1	0	1	1	6.1	0.08	10.7
1	0	0	100	6.86	1	76	22.9	136	73.5	205	53	152	13.3	6.3	59.2	2	1	0	1	1	1	1	13.7	0.03	26.8
1	0	0	100	7	1	65	23.4	126.5	77.5	233	53	180	15	5.7	76.2	2	1	0	1	0	1	1	15.9	0.03	18.6
1	0	0	100	5.83	1	72	24.1	128	77	199	53	146	14	5.5	81.7	2	0	0	0	1	1	1	37.3	0.03	18.5
1	0	0	100	7.01	1	70	21.7	155	77.5	287	53	234	14.2	5.4	77.7	2	1	0	0	0	1	1	8.2	0.02	30.6
1	0	0	100	7.02	1	67	23.2	109	67	214	53	161	13.9	7	84.6	2	1	0	1	1	0	1	7.2	0.01	2.35
1	0	0	100	7.03	1	70	23.9	108	64.5	173	53	120	15	5.4	61.7	2	1	1	0	1	1	1	9	0.05	42.9
1	0	0	100	7.04	1	66	22.3	127	80	196	53	143	14.4	5.7	75.7	2	0	0	1	1	1	1	7.7	0.08	12
1	0	0	100	5.06	1	65	23.4	137	82	152	53	99	13.3	5.5	72.6	2	1	1	1	1	1	1	9.7	0.06	21.6
1	0	0	100	6.97	1	73	23.6	126.5	79.5	167	53	114	13.5	5.5	72.0	2	1	1	0	1	1	1	17	0.06	2.8
1	0	0	100	4.62	1	68	22.3	149.5	87	216	53	163	14.1	5.6	71.1	2	1	0	0	1	1	1	10.7	0.03	13.9
1	0	0	100	4.63	1	65	24.1	116.5	63.5	200	53	147	15.8	5.7	76.2	2	1	0	1	1	1	0	7.7	0.07	169
1	0	0	100	4.64	1	77	24	120	66	183	53	130	13.6	5.5	70.1	2	1	0	1	1	1	1	5.9	0.08	14.3

1	0	0	100	4.64	1	69	21.2	158.5	85	140	53	87	14.9	4.5	70.6	2	1	0	0	1	1	1	6.4	0.01	25.2
1	0	0	100	4.64	1	78	28.3	149	82	175	53	122	16.5	7	69.6	2	1	1	0	1	0	1	70.9	0.06	11.2
1	0	0	100	6.95	1	79	20.1	150	80	214	53	161	15.3	5.2	69.1	2	1	1	0	1	1	1	42.6	0.01	32.7
1	0	0	100	4.59	1	68	22.1	141	76	198	53	145	13.3	5	71.1	2	1	1	0	1	1	1	11.9	0.03	25.3
1	0	0	100	4.62	1	79	19.7	123.5	72	163	53	110	12.2	5.6	73.0	1	1	1	1	1	1	1	19	0.01	46.9
1	0	0	100	2.17	1	68	22.5	106	55.5	193	53	140	14.4	5.6	78.8	2	0	0	0	1	1	1	5.9	0.07	16.2
1	0	0	100	6.96	1	72	22.2	157.5	83	191	53	138	11.2	7.9	76.7	2	1	1	0	1	0	1	3	0.08	0.05
1	0	0	100	5.37	1	66	21.2	149.5	85	157	53	104	16.4	5.2	79.9	2	0	0	0	1	1	1	12.6	0.02	54
1	0	0	100	5.06	1	66	23.4	141.5	90.5	191	53	138	14.6	5.7	75.7	2	0	0	0	1	1	1	14.3	0.05	21.4
1	0	0	100	1.46	1	65	20.3	138	82.5	114	53	61	14.3	5.2	76.2	2	0	0	0	1	1	1	78.7	0.05	43.4
1	0	0	100	5.04	1	66	24.2	153.5	92.5	171	53	118	15.6	5.1	79.9	2	0	0	0	1	1	1	15.9	0.05	41.5
1	1	0	100	4.56	1	72	21.1	136.5	88	210	53	157	12.7	6	69.1	2	1	0	1	1	1	1	4.3	0.02	116
1	1	0	100	6.44	1	77	25.2	115	69.5	154	53	101	15.8	6.5	66.7	2	1	0	1	1	0	1	5	0.03	15.3
1	0	0	100	7.08	2	69	22	132	74.5	203	53	150	13.8	5.7	80.5	1	1	1	0	1	1	1	32.9	0.13	5.41
1	0	0	100	4.58	2	66	23.9	106	62.5	169	53	116	14.3	6.7	77.5	2	1	1	1	1	0	1	9.1	0.12	30.5
1	0	0	100	6.87	2	68	21.2	138	84.5	208	53	155	13.3	5.5	72.6	2	1	1	0	1	1	1	12.2	0.12	9.92
1	0	0	100	6.87	2	68	25.1	131	87	205	53	152	14.8	5.4	76.4	2	1	1	0	1	1	1	65.9	0.49	39.7
1	0	0	100	7.04	2	65	29.7	122	72	212	53	159	12.4	5.3	74.2	1	1	1	1	1	1	1	14.4	0.11	9.52
1	0	0	100	5.08	2	69	22.9	99	64.5	139	53	86	12.1	5.7	72.1	2	1	1	1	1	1	1	3.6	0.2	30.9
1	0	0	100	6.99	2	69	25.6	105	64.5	193	53	140	13.5	6.5	75.9	1	1	1	1	1	0	1	76.7	0.12	0.65
1	0	0	100	4.62	2	71	27.5	133	79.5	231	53	178	13.1	5.3	71.1	2	1	0	0	0	1	1	16.2	0.27	10.8
1	0	0	100	6.97	2	69	26.1	124.5	77.5	200	53	147	12.7	5.4	80.5	2	1	1	0	0	1	1	70.7	0.68	6.54
1	0	0	100	6.8	2	70	26.4	146	81	231	53	178	14	5.6	60.9	2	1	1	0	0	1	1	78	0.14	20.5
1	0	0	100	6.83	2	75	25.7	174.5	97	223	53	170	13	5.9	72.7	2	1	1	0	0	1	1	155.8	0.1	26.7
1	0	0	100	4.69	2	66	25.9	152.5	86	216	53	163	13.6	6	73.6	2	1	1	0	1	1	1	9.6	0.16	22.2
1	0	0	100	4.69	2	70	22.4	148.5	82.5	174	53	121	12.3	5.3	75.3	1	1	1	0	1	1	1	12.8	0.16	25.8
1	0	0	100	4.7	2	70	27.2	115.5	65	183	53	130	14.7	5.4	71.6	1	1	1	0	0	1	1	65.1	0.14	8.04
1	0	0	100	7	2	66	23.4	111	73.5	194	53	141	11	6	77.5	2	1	1	1	1	1	1	13.3	0.04	22
1	0	0	100	7	2	66	21.5	115.5	71.5	229	53	176	13.2	5.6	73.6	2	1	0	1	0	1	1	17.9	0.03	23.9
1	0	0	100	7.06	2	72	22.5	120.5	65.5	198	53	145	13.3	5.8	70.6	2	1	1	1	1	1	1	26.1	0.07	29.2
1	0	0	100	4.59	2	65	21.7	117.5	74	192	53	139	13.1	5.7	63.1	2	1	0	1	1	1	1	10.2	0.03	14.6
1	0	0	100	6.87	2	69	20.4	152.5	102.5	226	53	173	13.2	5.5	72.1	2	1	1	0	0	1	1	6.1	0.04	11.8
1	0	0	100	6.87	2	73	21.6	134	73	227	53	174	13.8	5.3	73.8	2	1	1	1	0	1	1	125.5	0.03	24.5
1	0	0	100	5.16	2	66	25.5	119	79.5	203	53	150	14.5	5.7	77.5	2	1	1	0	0	1	1	25.1	0.05	17.6
1	0	0	100	6.84	2	72	24.7	134.5	68.5	218	53	165	11.8	6.1	78.9	2	1	1	0	1	1	1	15.1	0.03	25.1
1	0	0	100	6.85	2	65	21.8	140.5	79	256	53	203	12.5	5.1	78.0	2	1	1	0	0	1	1	19.9	0.04	14.8
1	0	0	100	6.85	2	73	27.9	140	81	278	53	225	13.6	5.4	70.1	1	1	1	0	0	1	1	24.9	0.05	11.9
1	0	0	100	6.85	2	65	17.6	150.5	82.5	176	53	123	12.2	5.1	78.0	2	1	1	0	1	1	1	27.5	0.01	34.1
1	0	0	100	6.85	2	65	18.5	111	76	181	53	128	11.8	5.3	78.0	2	1	0	1	1	1	1	8.1	0.06	9.07
1	0	0	100	7.21	2	68	20.8	135.5	73	229	53	176	11.9	5.5	61.8	2	1	0	0	0	1	1	15.4	0.07	26.7
1	0	0	100	7.21	2	70	25.7	113.5	74.5	246	53	193	14.3	5.6	60.9	2	1	1	1	0	1	1	11.8	0.08	4.91
1	0	0	100	6.86	2	65	24.9	99.5	73	192	53	139	13.9	5.6	63.1	2	1	1	0	1	1	1	13.3	0.04	55.7
1	0	0	100	6.86	2	66	27.2	164.5	98.5	245	53	192	12.7	5.3	62.7	2	1	1	0	0	1	1	3.9	0.02	8.56
1	0	0	100	7.04	2	66	21.8	111	74	159	53	106	12	5.3	82.3	2	1	1	1	1	1	1	3.6	0.03	13.9
1	0	0	100	5.07	2	73	26.2	115	64	213	53	160	14.3	6.9	78.3	1	0	0	1	1	0	1	30.1	0.07	12.1
1	0	0	100	5.08	2	66	22.1	134.5	74	217	53	164	12.5	5.6	73.6	2	1	1	0	1	1	1	105	0.04	36.8
1	0	0	100	4.62	2	71	21.4	143	76	189	53	136	12.9	4.9	74.8	1	1	1	0	1	1	1	38.1	0.04	21.8
1	0	0	100	4.63	2	76	31.6	175	100	226	53	173	13.9	6.1	68.6	1	1	1	0	0	1	1	68.2	0.08	33.4
1	0	0	100	4.64	2	77	24	120.5	73	205	53	152	15.8	5.4	68.2	1	1	1	0	1	1	1	4.1	0.08	4.83
1	0	0	100	6.87	2	70	25.8	139	75.5	165	53	112	13.6	5	80.0	2	1	1	1	1	1	1	12.8	0.04	11.7
1	0	0	100	6.97	2	77	25.4	159	97	170	53	117	14.2	4.9	76.1	1	1	1	0	1	1	1	17.5	0.02	8.73

1	0	0	100	6.98	2	67	23.9	152	81.5	231	53	178	14.5	6.1	76.9	2	1	1	0	0	1	1	16.9	0.06	15.4
1	0	0	100	6.79	2	73	24.7	126	72	268	53	215	14.5	5.7	70.1	1	1	1	1	0	1	1	12.3	0.06	7.34
1	0	0	100	6.8	2	68	22.3	130	77	209	53	156	14.2	5.7	72.6	2	1	1	0	1	1	1	15.5	0.04	7.65
1	0	0	100	6.84	2	71	21.6	123.5	68.5	186	53	133	11.3	5.2	74.8	2	1	0	1	1	1	1	27.7	0.01	9.69
1	0	0	100	4.59	2	68	21	131.5	68	253	53	200	11	5.4	81.1	2	1	1	1	0	1	1	32.7	0.01	15.9
1	0	0	100	4.59	2	70	25	124	69.5	224	53	171	11.6	5.7	75.3	2	1	1	1	0	1	1	8.2	0.06	34.7
1	0	0	100	4.62	2	76	27.4	137.5	79	291	53	238	15	5.3	68.6	2	1	1	1	0	1	1	25	0.08	13.6
1	0	0	100	2.14	2	65	25.8	131.5	73	200	53	147	15.2	5.9	78.0	2	1	1	0	0	1	1	12.5	0.02	12.6
1	0	0	100	4.69	2	70	23.1	123.5	74.5	176	53	123	13	5.4	60.9	2	1	1	1	0	1	1	28.2	0.04	10.2
1	0	0	100	5.37	2	65	24.4	116.5	63.5	223	53	170	12.9	7.1	78.0	2	1	1	1	0	0	1	17.6	0.03	21
1	0	0	100	5.37	2	72	20.7	117	72.5	178	53	125	12.7	5.2	60.1	2	1	1	0	1	1	1	31.8	0.03	33.3
1	0	0	100	4.68	2	75	19.7	115	63.5	150	53	97	11.6	5.5	72.7	1	1	0	1	1	1	1	7.1	0.01	39.4
1	0	0	100	5.37	2	68	25.5	142.5	81.5	169	53	116	11.7	5.8	61.8	2	1	1	0	1	1	1	13.7	0.02	33.7
1	0	0	100	5.05	2	68	29.6	150	83	257	53	204	15	6.4	81.1	2	1	1	0	0	1	1	10.7	0.09	16.1
1	0	0	100	5.06	2	70	23.8	155	87.5	209	53	156	13.6	5.6	71.6	2	1	1	0	1	1	1	16.3	0.05	11.3
1	0	0	100	6.86	2	69	26.3	176.5	94.5	216	53	163	11.7	5.5	75.9	1	1	1	0	1	1	1	8.8	0.03	12.3
1	0	0	100	5.04	2	69	28.4	127	80.5	274	53	221	12.1	5.3	72.1	2	1	1	1	0	1	1	21.7	0.04	12.1
1	0	0	100	5.04	2	71	29.3	144.5	70	186	53	133	11.4	5.1	79.4	1	1	1	0	1	1	1	13	0.06	28.1
1	1	0	100	7.03	2	73	23.8	129.5	72.5	201	53	148	13	5.2	59.7	1	1	1	0	1	1	1	29.4	0.33	40
1	1	0	100	6.95	2	65	27.2	143.5	81.5	230	53	177	13.5	6.5	78.0	1	1	1	0	0	0	1	23.7	0.05	0.05
1	0	0	100	4.6	1	82	23.4	120	69	228	53	175	13.8	5.5	67.6	2	0	1	1	0	1	1	12	0.07	8.01
1	0	0	100	6.84	2	84	26	133.5	71	117	53	64	10.8	5.2	68.3	1	1	1	0	1	1	1	33.4	0.02	34.4
1	0	0	100	4.64	2	82	22.6	157	89	219	53	166	13.5	5.9	69.2	1	1	1	0	1	1	1	25.9	0.01	25.7
1	0	0	100	5.36	2	83	24.3	100	57	166	53	113	11.5	5.8	68.7	1	1	1	1	1	1	1	27	0.05	91.1
1	1	0	100	6.87	2	82	23.5	135	84.5	166	53	113	11.6	5.3	65.8	1	1	1	1	1	1	1	103.5	0.08	37.6
1	0	0	100	4.62	1	78	28.2	121.5	67	209	53	156	13.9	5.9	69.6	2	0	0	1	1	1	1	67.9	0.7	21.7
1	0	0	100	1.78	1	79	20.9	136	62	151	53	98	12.6	5.1	77.7	2	0	0	1	1	1	1	10.2	0.01	50.9
1	0	0	100	6.85	1	71	20.9	152	75.5	142	53	89	13.2	5.1	77.2	2	1	0	0	1	1	1	5.1	0.04	69.7
1	0	0	100	7.02	1	66	25.6	131.5	75.5	233	53	180	13.8	5.5	79.9	2	0	0	0	0	1	1	75.3	0.01	13.1
1	0	0	100	1.82	1	73	18.6	137.5	69	137	53	84	13.4	5.5	76.1	2	0	0	1	1	1	1	8.5	0.01	46.8
1	0	0	100	7.03	1	74	24.7	103.5	74.5	171	53	118	13.9	5.1	80.5	2	1	0	1	1	1	1	5.6	0.09	0.05
1	0	0	100	4.63	1	76	23.5	133	88	176	53	123	13.9	5.8	59.2	2	1	1	0	1	1	1	21.2	0.08	14.6
1	0	0	100	6.85	1	71	19.7	142.5	74	174	53	121	14.5	5.6	77.2	2	1	1	0	1	1	1	12.3	0.01	54.3
1	1	0	100	6.87	1	75	22.3	138.5	83.5	191	53	138	13.9	6	71.0	1	0	1	0	1	1	1	17	0.61	29.4
1	1	0	100	3.22	1	67	22.5	143	78	152	53	99	15	7.2	84.6	1	0	0	0	1	0	1	36	0.64	12.8
1	0	0	100	4.59	2	65	26.2	116	77	136	53	83	12.8	5.6	82.8	2	1	1	1	1	1	0	43.4	0.12	148
1	0	0	100	6.87	2	77	19.6	157.5	93.5	242	53	189	13.1	5.9	71.7	1	1	1	0	0	1	1	7.2	0.12	57.5
1	0	0	100	5.05	2	66	27	144	80	167	53	114	13.8	5.7	88.5	2	1	1	0	1	1	1	48.6	0.18	9.02
1	0	0	100	5.06	2	65	26.7	112.5	70	244	53	191	13.3	5.6	63.1	2	1	1	1	0	1	1	3.6	0.56	23.3
1	0	0	100	6.87	2	74	24.3	111	56	200	53	147	13.6	4.8	73.2	1	1	1	1	1	1	1	11.8	0.1	25
1	0	0	100	7	2	68	27.8	124.5	64.5	140	53	87	13.3	4.7	76.4	2	1	0	0	1	1	1	32.6	0.03	57.4
1	0	0	100	7.05	2	69	25.6	133.5	79.5	189	53	136	12.5	5.6	72.1	1	1	1	0	1	1	1	89.4	0.05	13.3
1	0	0	100	7.05	2	73	29.1	141.5	82	302	53	249	13.4	5.6	59.7	2	1	1	0	0	1	1	31	0.06	30.6
1	0	0	100	7.06	2	75	24.6	156	83	142	53	89	11.8	5.8	72.7	1	1	1	0	1	0	1	16.9	0.03	32.9
1	0	0	100	7.08	2	77	21.9	127.5	73	174	53	121	11.7	5.2	71.7	1	1	1	1	1	1	1	27.5	0.02	58.4
1	0	0	100	4.58	2	71	20.1	157.5	80	258	53	205	11.5	5.4	74.8	2	1	1	0	0	1	1	16.1	0.02	30.8
1	0	0	100	6.86	2	69	25.3	158	84.5	290	53	237	14.3	7	75.9	2	1	1	0	0	0	1	35.1	0.07	5.83
1	0	0	100	5.16	2	69	28.4	162	96.5	197	53	144	13.7	5.4	80.5	2	1	1	0	1	1	1	245.6	0.09	49.4
1	0	0	100	7.2	2	78	24.6	116	62.5	150	53	97	12.3	5.1	71.2	2	1	1	1	1	1	1	24.6	0.07	37.7
1	0	0	100	6.86	2	68	22.8	163.5	99	230	53	177	11.4	5.7	76.4	2	1	1	0	0	1	1	97.3	0.03	0.05
1	0	0	100	3.16	2	79	31.1	149.5	77	186	53	133	14	5.6	75.1	1	1	1	0	1	1	1	60.9	0.07	7.78

1	0	0	100	7.03	2	74	26.7	124	80	220	53	167	11.8	5.2	69.6	1	1	1	0	0	1	1	29.4	0.05	0.05
1	0	0	100	5.05	2	71	21.1	119	58.5	165	53	112	14.3	5.2	79.4	1	1	1	1	1	1	1	17.1	0.05	38.6
1	0	0	100	5.05	2	65	20.9	107.5	63	207	53	154	13.9	5.4	74.2	2	1	1	0	1	1	1	36.2	0.01	16.3
1	0	0	100	5.07	2	70	27.9	106	58	184	53	131	13.7	5.5	71.6	2	1	1	1	0	1	1	35.3	0.03	19.6
1	0	0	100	5.07	2	69	26.8	125	75	171	53	118	12.7	5.2	75.9	2	1	1	1	1	1	1	18.6	0.06	72.5
1	0	0	100	5.07	2	68	29	123.5	73.5	189	53	136	12.5	5.7	72.6	2	1	1	0	1	1	1	8.9	0.02	90.6
1	0	0	100	5.08	2	67	23.1	101	62	197	53	144	11.2	5.1	73.1	2	1	1	1	1	1	1	6.4	0.07	20.9
1	0	0	100	6.96	2	67	28.1	114	68.5	193	53	140	11.6	5.1	76.9	1	1	1	1	1	1	1	23.8	0.06	56.3
1	0	0	100	4.61	2	72	28.9	129.5	71.5	152	53	99	12.3	5.5	70.6	1	1	1	0	1	1	1	36.4	0.04	64.2
1	0	0	100	3.04	2	77	22.1	145	87	163	53	110	12.6	5.3	71.7	1	1	1	0	1	1	1	24.3	0.01	20.5
1	0	0	100	5.06	2	78	23.9	115	70	167	53	114	12.4	5.5	71.2	2	1	1	0	1	1	1	9	0.02	27.7
1	1	0	100	6.86	2	65	38	123	72.5	188	53	135	13.1	5.1	74.2	2	1	1	0	0	1	1	30	0.1	12.7
1	1	0	100	6.95	2	73	29.3	110.5	66	196	53	143	12.2	5.7	73.8	2	1	1	1	1	0	1	9.6	0.09	20.4
1	0	0	100	4.64	1	82	20.8	94.5	46	187	53	134	12.1	5.6	71.5	2	1	1	1	1	1	1	4.9	0.12	42.6
1	0	0	100	4.59	1	82	25.2	122.5	75.5	163	53	110	13.1	7.2	67.6	1	1	0	1	1	0	1	46.4	0.06	62
1	0	0	100	7	2	85	27.9	136.5	79.5	203	53	150	13.6	5.4	54.8	1	1	1	0	1	1	1	7.6	0.19	28.1
1	0	0	100	7.01	2	84	23.9	160.5	82.5	171	53	118	12.1	5.4	64.9	1	1	1	0	1	1	1	183.4	0.03	43.7
1	0	0	100	6.8	2	85	22.1	176	91.5	136	53	83	12.1	5.3	47.6	1	1	1	0	1	1	1	20.6	0.04	16.2
1	0	1	4.4	4.4	1	76	25.2	155	88	218	53	165	15.2	5.7	52.7	2	1	0	0	1	1	1	17.4	0.01	10.6
1	1	1	6.1	6.1	1	71	25.7	176.5	98	207	53	154	15.5	6.4	77.2	2	0	0	0	1	1	1	13.3	0.01	15.5
1	1	1	2.1	2.1	1	74	21.4	144.5	66.5	165	53	112	14.8	5.5	75.6	2	0	1	0	1	1	1	20.3	0.01	38.5
1	0	1	1.4	1.4	2	66	29.5	116	70	251	53	198	13.7	5.6	73.6	2	1	1	1	0	1	1	111.8	0.17	19.5
1	1	1	0.5	0.5	2	69	21.7	153	86	193	53	140	13.1	5	61.4	2	1	1	0	1	1	1	53.6	0.02	44.1
1	1	1	1	1	2	73	23.1	143.5	87	204	53	151	14.5	5.4	73.8	1	1	1	0	1	1	1	187.5	0.02	58.5
1	0	1	2.9	2.9	1	80	26.9	115	66	174	53	121	14.5	5.5	57.5	2	0	0	1	1	1	1	10.3	0.21	38.3
1	1	1	3.7	3.7	1	75	27.1	137	85.5	204	53	151	16.5	5.3	59.6	2	1	0	1	1	1	1	36.8	0.36	17.5
1	1	1	6.7	6.7	1	71	26.1	132.5	69	224	53	171	16.1	7	61.3	2	0	1	1	0	0	1	11.5	0.02	13.3
1	1	1	4.7	4.7	2	75	22.5	158.5	95	195	53	142	11.7	5	72.7	1	1	1	0	1	1	1	14.1	0.69	206
1	1	1	6.1	6.1	2	69	26.8	145.5	75.5	242	53	189	14.5	5	80.5	1	1	1	0	0	1	1	13.8	0.33	83.6
1	1	1	2.9	2.9	2	78	29.2	117	58.5	188	53	135	12.7	6.3	50.0	1	1	1	0	1	1	1	18.1	0.1	14.4
1	0	1	0.1	0.1	2	80	26.1	91.5	47	224	53	171	14.2	6.9	66.7	2	1	1	1	0	0	0	23.7	0.06	186
1	1	1	3.4	3.4	2	81	25.1	110	59.5	263	53	210	11.3	5.9	28.7	1	1	1	0	0	1	0	122.3	0.51	505
1	0	0	100	7.05	1	71	19.5	143.5	80	167	54	113	14.6	5.9	69.6	2	0	0	0	1	1	1	26.9	0.21	41.7
1	0	0	100	7.08	1	71	25.2	176.5	103	123	54	69	14.5	4.9	54.6	2	0	0	0	1	1	1	12.9	0.11	50.7
1	0	0	100	4.78	1	67	27.7	124	83	184	54	130	13.6	5.8	71.6	2	1	1	1	1	1	1	6.3	0.72	34.5
1	0	0	100	6.84	1	79	21.3	114.5	63	222	54	168	12.7	5.2	69.1	2	1	0	1	0	1	1	13.6	0.32	7.86
1	0	0	100	0.41	1	66	25.7	135.5	74.5	172	54	118	15.9	7.9	75.7	2	1	0	1	1	0	1	11.6	0.19	43.4
1	0	0	100	6.96	1	65	32.2	167	84	242	54	188	16.4	9.6	72.6	2	0	0	0	0	0	1	311.5	0.46	220
1	0	0	100	6.8	1	73	23	125	74	226	54	172	15.5	5.3	81.1	2	1	0	0	0	1	1	6.1	0.42	26
1	0	0	100	4.61	1	73	25.4	137.5	82.5	170	54	116	12.8	5.7	68.6	2	1	1	1	1	1	1	6.3	0.13	56.6
1	0	0	100	4.7	1	74	22.7	167.5	70	230	54	176	13.7	5.5	71.5	1	0	1	0	0	1	1	25.2	0.13	41.1
1	0	0	100	6.87	1	69	27	150.5	84	198	54	144	14.5	5.5	74.1	1	1	1	0	1	1	1	9.9	0.23	31
1	0	0	100	7	1	65	20.3	136.5	81	172	54	118	13.9	5.3	76.2	2	1	0	1	1	1	1	8.4	0.01	16.9
1	0	0	100	2.98	1	72	21.9	148.5	80.5	271	54	217	15	5.5	76.7	2	1	1	0	0	1	1	111.2	0.08	9.33
1	0	0	100	4.58	1	76	20.9	137	65	179	54	125	12.5	5.6	47.5	2	0	0	1	1	1	1	5.2	0.05	61.4
1	0	0	100	6.86	1	76	32.6	143	92.5	152	54	98	14.2	4.5	47.5	2	1	0	0	1	1	1	10.5	0.06	30.5
1	0	0	100	6.87	1	69	25.1	129.5	88.5	177	54	123	13.6	5	35.2	2	1	0	1	1	1	1	144.5	0.03	25.9
1	0	0	100	6.87	1	77	19.9	105	73	207	54	153	13.8	5	52.4	2	1	0	0	1	1	1	40.7	0.04	82.6
1	0	0	100	6.87	1	69	22	132.5	87.5	211	54	157	14.7	5.2	49.9	2	1	0	0	1	1	1	108.7	0.04	110
1	0	0	100	6.88	1	76	24.8	123.5	61.5	205	54	151	14.5	4.9	59.2	2	1	0	0	1	1	1	59.2	0.06	68.9
1	0	0	100	5.15	1	75	22.9	108.5	67	164	54	110	15.2	5.5	59.6	2	1	1	1	1	1	1	5.9	0.01	35.9

1	0	0	100	5.16	1	67	18	93	58	124	54	70	12.9	5.2	75.2	2	1	0	1	1	1	1	8.8	0.02	76.9
1	0	0	100	3.52	1	74	21.7	129.5	84.5	156	54	102	14.5	5.4	71.5	2	0	0	1	1	1	0	17.1	0.03	57.5
1	0	0	100	6.84	1	71	22.3	143.5	87.5	159	54	105	12.1	5.4	61.3	2	0	1	0	1	1	1	47	0.09	40.7
1	0	0	100	7	1	77	27.4	163	95.5	174	54	120	13.9	5.4	52.4	2	1	1	0	1	1	1	16.4	0.04	28.2
1	0	0	100	3.49	1	75	21.2	121.5	76.5	204	54	150	13.2	5.7	71.0	2	1	1	1	1	1	1	5.2	0.09	19.8
1	0	0	100	7.03	1	67	22.6	108.5	69	221	54	167	14.7	5.2	71.6	2	1	1	1	0	1	1	15	0.03	8.35
1	0	0	100	7.03	1	74	27.9	165.5	82	204	54	150	14.5	5.5	60.0	2	1	0	0	1	1	1	122	0.09	56.2
1	0	0	100	5.08	1	71	22.4	156	81.5	208	54	154	13.4	5.6	77.2	1	1	0	0	1	1	1	6.6	0.07	18.2
1	0	0	100	6.98	1	69	23.7	142.5	84	205	54	151	13.6	6	70.6	2	1	0	0	1	1	1	7.9	0.09	0.92
1	0	0	100	6.99	1	72	25.7	109	67.5	170	54	116	13.4	6.1	69.1	2	1	0	1	1	1	1	7	0.07	15.8
1	0	0	100	4.62	1	75	24.8	171	81.5	160	54	106	16	5.2	71.0	2	1	0	0	1	1	1	10.3	0.06	24.7
1	0	0	100	4.62	1	65	27.2	115.5	73	219	54	165	14.1	5.2	63.9	2	0	0	1	1	1	1	5.4	0.03	43.1
1	0	0	100	4.62	1	78	23.5	112	64	151	54	97	13.7	5.2	78.3	2	1	1	1	1	1	1	11.6	0.04	20
1	0	0	100	4.63	1	69	26.3	141.5	91	194	54	140	15.1	5	70.6	2	1	0	0	1	1	1	20.8	0.09	29.3
1	0	0	100	4.64	1	76	24.6	137	83.5	196	54	142	14.4	4.9	67.2	2	1	0	1	1	1	1	51.1	0.04	32.4
1	0	0	100	6.83	1	65	25.5	158.5	90.5	200	54	146	15.5	7	80.5	2	0	0	0	1	0	1	51.9	0.05	10.4
1	0	0	100	4.85	1	72	22.7	112	63.5	224	54	170	14.7	5.4	76.7	2	0	0	1	0	1	1	26.3	0.05	8.44
1	0	0	100	7.15	1	71	19.7	112	75	168	54	114	10.9	5.6	73.1	1	1	1	1	1	1	1	18.4	0.02	28.5
1	0	0	100	6.97	1	65	29.5	140	73	195	54	141	17.6	6.3	72.6	2	1	0	0	1	1	0	52.8	0.08	0.05
1	0	0	100	3.63	1	74	21.7	112.5	71.5	204	54	150	13.1	7	53.5	2	1	1	1	0	0	1	63.5	0.03	15.3
1	0	0	100	5.06	1	74	23.2	128.5	80	177	54	123	13.7	5.1	60.0	2	1	0	0	1	1	1	16.8	0.03	45.3
1	0	0	100	5.06	1	70	23.6	134.5	84.5	192	54	138	14.4	5.1	70.1	2	0	0	1	1	1	1	4.3	0.07	1.29
1	0	0	100	5.07	1	66	23.7	140.5	82	203	54	149	16.5	5.5	72.1	2	1	0	0	1	1	1	4.8	0.02	38.4
1	1	0	100	5.36	1	73	28.6	127.5	75	179	54	125	14.8	10.8	72.0	2	1	1	1	1	0	1	41.3	0.09	59.9
1	0	0	100	7	2	66	23.8	152	92	177	54	123	13.3	5.1	77.5	2	1	1	0	1	1	1	8.7	1.74	28.6
1	0	0	100	6.86	2	78	26.2	157	84	224	54	170	10.7	5.2	44.0	1	1	1	0	0	1	1	36.2	0.35	121
1	0	0	100	6.87	2	71	27.2	129	70	223	54	169	13.8	5.9	52.5	2	1	1	1	0	1	1	14.4	0.16	33.3
1	0	0	100	6.87	2	65	25.6	121.5	78	178	54	124	14	5.6	82.8	2	1	1	1	1	1	1	32	0.19	17.2
1	0	0	100	6.88	2	65	18.5	133	75.5	260	54	206	13.6	5.3	74.2	2	1	1	1	0	1	1	8.6	0.15	55.6
1	0	0	100	6.84	2	78	16.5	170.5	93.5	211	54	157	12	5.6	71.2	1	1	1	0	1	1	1	18.7	5.26	8.05
1	0	0	100	6.86	2	67	24.2	128.5	77.5	244	54	190	13.8	6.3	73.1	2	1	1	0	0	1	1	12.3	0.13	42.4
1	0	0	100	6.86	2	74	21.7	101	45.5	189	54	135	12.7	6	59.2	2	1	1	0	1	1	1	12.3	0.12	40.9
1	0	0	100	6.86	2	74	26.4	137	74	219	54	165	12.1	5.6	73.2	2	1	1	0	1	1	1	16.6	0.11	47.3
1	0	0	100	5.05	2	73	28.2	141	92	195	54	141	13.7	5	59.7	2	1	1	0	1	1	1	37.4	0.14	52.3
1	0	0	100	6.6	2	68	24.1	163.5	87.5	223	54	169	14.1	5.3	76.4	1	1	1	0	0	1	1	47.6	0.21	8.08
1	0	0	100	7	2	70	21.9	136	80	178	54	124	12.9	5.4	75.3	2	1	1	1	1	1	1	11.1	0.17	7.91
1	0	0	100	6.96	2	71	31.3	136.5	90.5	175	54	121	12.7	5.3	71.1	1	1	1	0	1	1	1	16.3	0.11	21.9
1	0	0	100	4.59	2	71	25.4	127	78.5	223	54	169	14.3	5.7	60.5	2	1	1	1	0	1	1	6.9	0.28	16.4
1	0	0	100	4.6	2	74	30.7	148.5	92	247	54	193	13.2	5.5	69.6	2	1	1	0	0	1	1	10.6	0.16	85.5
1	0	0	100	5.37	2	74	25.7	159	100.5	194	54	140	13.9	5.5	73.2	1	1	1	0	1	1	1	36.6	0.31	28.1
1	0	0	100	5.06	2	65	27.7	129	66	195	54	141	14.5	5.6	63.1	2	1	1	0	1	1	0	84.7	0.12	144
1	0	0	100	4.58	2	77	28.6	110.5	65.5	209	54	155	12.9	5.9	68.2	2	1	1	1	0	1	1	9.9	0.08	27.3
1	0	0	100	4.58	2	70	26.2	135	76	218	54	164	13.1	5.2	75.3	2	1	1	1	1	1	1	70.7	0.01	24.9
1	0	0	100	4.59	2	69	25.4	135	73	206	54	152	12.3	6	75.9	2	1	1	0	1	1	1	19.1	0.05	22.9
1	0	0	100	6.88	2	66	22	112.5	65.5	197	54	143	14.2	5.4	62.7	2	1	1	1	1	1	1	8.4	0.01	23.7
1	0	0	100	5.15	2	74	27.3	114.5	60	235	54	181	13.5	5.7	77.8	2	1	1	1	0	1	1	12.7	0.02	72.4
1	0	0	100	5.16	2	71	26.3	104.5	64.5	190	54	136	13.4	5.6	79.4	2	1	1	0	1	1	1	4.7	0.01	27.4
1	0	0	100	5.16	2	67	23.1	132	74	190	54	136	11.3	5.4	73.1	2	1	1	0	1	1	1	12.3	0.06	23.1
1	0	0	100	6.84	2	66	25.1	98	54.5	274	54	220	12	5.5	77.5	2	1	1	1	0	1	1	15.9	0.08	5.38
1	0	0	100	7.21	2	71	31.3	131.5	78.5	250	54	196	14.8	5.4	74.8	2	1	1	1	0	1	1	14.1	0.08	26.8
1	0	0	100	6.86	2	73	20.7	126.5	74	209	54	155	11.2	5.2	59.7	2	1	1	0	1	1	1	87	0.03	21.2

1	0	0	100	6.86	2	65	20.8	130.5	69.5	199	54	145	11.9	5.3	74.2	2	1	1	1	1	1	1	29	0.04	32
1	0	0	100	6.86	2	65	24.8	172	86.5	226	54	172	13.4	6.4	82.8	2	1	1	0	0	1	1	17	0.06	25.4
1	0	0	100	7.01	2	65	21.9	113.5	75	162	54	108	11.2	5.3	78.0	2	1	1	1	1	1	1	15.1	0.06	15.5
1	0	0	100	7.02	2	72	23.1	89.5	49	231	54	177	12.5	5.8	74.3	1	1	1	1	0	1	1	10.1	0.05	8.13
1	0	0	100	5.05	2	70	27.3	121.5	71.5	274	54	220	13.2	6.2	75.3	2	1	1	1	0	1	1	32.7	0.09	8.32
1	0	0	100	5.06	2	70	23.1	139	73	217	54	163	10.9	5.5	75.3	1	1	1	0	1	1	1	47.8	0.03	10.4
1	0	0	100	5.07	2	68	23.1	143	85.5	179	54	125	13	5.6	72.6	2	1	1	0	1	1	1	22.1	0.09	43.6
1	0	0	100	5.22	2	71	20.9	111.5	66.5	186	54	132	13	5.5	79.4	2	1	1	1	0	1	1	14.6	0.03	3.26
1	0	0	100	6.98	2	70	27.2	115.5	68	188	54	134	13.6	5.3	75.3	1	1	1	0	1	1	1	11.3	0.09	22.3
1	0	0	100	4.62	2	73	25.2	133	71	207	54	153	13	6.2	73.8	2	1	1	1	0	1	1	20	0.03	10.1
1	0	0	100	4.62	2	69	21.3	90.5	58	177	54	123	12.6	5.9	75.9	2	1	1	1	0	1	1	5.7	0.04	15.7
1	0	0	100	4.63	2	66	23.4	134	74.5	218	54	164	9.3	5.9	62.7	2	1	1	1	1	1	1	22.5	0.04	19.7
1	0	0	100	6.97	2	73	28.8	132	92	193	54	139	12	5.6	70.1	1	1	1	0	0	1	1	10	0.07	13.6
1	0	0	100	6.8	2	79	22.9	129.5	75	203	54	149	14.2	4.8	67.2	1	1	0	0	1	1	1	6.5	0.03	31
1	0	0	100	6.8	2	69	21	137	83.5	206	54	152	12.5	5.7	75.9	2	1	1	1	1	1	1	18.4	0.03	29.4
1	0	0	100	6.84	2	69	23.5	125.5	73.5	156	54	102	12.8	5.3	80.5	1	1	1	1	1	1	1	4.6	0.02	11.4
1	0	0	100	6.84	2	74	21.7	121	64.5	226	54	172	11.8	5.2	73.2	1	1	1	1	0	1	1	24.2	0.04	7.06
1	0	0	100	4.6	2	68	15.3	110	67	199	54	145	13.6	5	72.6	2	1	0	1	1	1	1	18.4	0.06	30.1
1	0	0	100	4.62	2	66	24.8	134.5	76.5	223	54	169	13.4	5.7	77.5	2	1	1	0	0	1	1	8.4	0.05	35
1	0	0	100	6.79	2	70	31.3	120	62.5	173	54	119	12.3	6.1	60.9	2	1	1	1	1	1	1	46.1	0.03	81.9
1	0	0	100	4.67	2	73	23.9	157	81	197	54	143	13.9	5.2	78.3	1	1	1	0	1	1	1	71.4	0.02	8.35
1	0	0	100	4.7	2	67	24.1	159	83.5	149	54	95	13.3	6.3	73.1	2	1	1	0	1	1	1	17.1	0.03	18.4
1	0	0	100	0.52	2	70	25.3	166.5	88	198	54	144	13.3	5.1	75.3	2	1	1	0	1	1	1	15.1	0.03	0.05
1	0	0	100	5.37	2	73	25	114	59.5	168	54	114	13.7	5.5	73.8	2	1	1	1	1	1	1	162.8	0.04	21.9
1	0	0	100	5.05	2	72	20.8	114	79	220	54	166	11.8	5.2	74.3	2	1	1	0	0	1	1	40.5	0.01	90.4
1	0	0	100	6.85	2	77	27.2	172	88	206	54	152	14.3	5.6	68.2	1	1	1	0	1	1	1	10.2	0.05	23.1
1	0	0	100	6.86	2	69	22.9	135.5	79	212	54	158	14.5	5.3	75.9	2	1	1	0	0	1	1	29.9	0.03	51.3
1	0	0	100	6.87	2	70	24.1	152.5	89.5	215	54	161	14.4	4.9	75.3	1	1	1	0	1	1	1	27.4	0.05	17.9
1	1	0	100	6.87	2	68	29.2	135	67	261	54	207	13.4	5.7	76.4	2	1	1	1	0	1	1	12.9	0.11	16.8
1	1	0	100	6.86	2	72	28	122.5	80.5	200	54	146	12.6	5.2	78.9	1	1	1	1	1	1	1	22.6	0.05	30.2
1	1	0	100	6.87	2	70	25.6	140.5	93	206	54	152	12.9	5.5	60.9	1	1	1	0	1	1	1	45.1	0.02	45.2
1	1	0	100	6.97	2	74	26.3	138.5	77.5	205	54	151	14.8	6.1	77.8	2	1	1	1	1	1	1	24.3	0.02	4.22
1	1	0	100	4.54	2	79	23.2	168	72.5	178	54	124	13.5	7.4	38.9	1	1	1	0	1	0	1	1697.7	0.08	54.1
1	0	0	100	4.59	1	81	24.9	139	75	199	54	145	13.9	6.5	68.1	2	1	0	1	1	0	1	30	0.11	35.1
1	0	0	100	5.06	1	84	24.1	123	69.5	223	54	169	14	6.9	66.7	2	0	0	1	0	0	1	3.6	0.14	33.9
1	0	0	100	4.59	1	80	22.1	136.5	72.5	232	54	178	14.4	8	65.4	2	1	0	1	0	0	1	18.8	0.03	71.7
1	0	0	100	4.62	1	83	17.1	96	52	146	54	92	11.9	4.8	56.3	2	1	1	1	1	1	1	12.7	0.06	44.5
1	1	0	100	4.58	1	89	25.3	164	88.5	197	54	143	14.8	6	61.3	2	1	0	0	1	1	1	46.8	2.19	41.4
1	1	0	100	4.52	1	87	24	142	80.5	185	54	131	13.8	5.1	33.5	2	1	1	0	1	1	1	533.1	0.03	15.9
1	0	0	100	5.15	1	70	28.3	111.5	70.5	187	54	133	14.5	5.6	73.6	2	1	0	0	1	1	0	273.7	0.13	195
1	0	0	100	4.64	1	68	17.2	126.5	88.5	151	54	97	15.5	5.2	90.5	2	1	0	1	1	1	1	28.5	0.01	15.4
1	1	0	100	4	1	73	27.5	138	74	159	54	105	14.6	7.1	60.4	2	1	0	0	1	0	1	9.8	0.29	4.34
1	1	0	100	5.04	1	72	20.8	139	84.5	165	54	111	13.8	5.1	81.7	2	1	0	1	1	1	1	43.4	0.02	41.7
1	0	0	100	6.86	2	75	23.4	141.5	81	216	54	162	11.7	5.5	58.8	1	1	1	0	1	1	1	7.7	0.1	50.7
1	0	0	100	6.84	2	75	29.7	121	71	288	54	234	14.1	5.5	72.7	2	1	1	0	0	1	1	14.8	0.22	14.1
1	0	0	100	5.05	2	73	24.9	120	71	167	54	113	13.9	6.4	73.8	2	1	1	0	0	1	1	23.3	0.12	53.1
1	0	0	100	4.62	2	78	25.3	150.5	84.5	195	54	141	13.7	5.3	57.6	2	1	1	0	1	1	1	11.5	0.13	63.4
1	0	0	100	6.8	2	74	28.4	133	72.5	204	54	150	14.1	5.2	73.2	2	1	1	1	1	1	1	6	0.1	26.8
1	0	0	100	6.87	2	66	23.9	130.5	90.5	198	54	144	13.6	5.2	82.3	1	1	1	0	1	1	1	26.6	0.1	19.9
1	0	0	100	5.04	2	66	24.4	129	72.5	261	54	207	13.2	5.2	77.5	2	1	1	1	0	1	1	560	0.2	17.6
1	0	0	100	7.08	2	69	23.8	116.5	74.5	206	54	152	12	5.3	72.1	2	1	1	1	1	1	1	29	0.08	4.35

1	0	0	100	4.58	2	74	25.3	131	83.5	199	54	145	13.5	5.6	77.8	2	1	1	1	1	1	1	45.1	0.01	36.1
1	0	0	100	6.88	2	74	25.2	127	81	214	54	160	12.9	4.9	77.8	1	1	1	1	1	1	1	37.3	0.06	24
1	0	0	100	6.84	2	72	27	121	73	247	54	193	14	5.7	78.9	2	1	1	1	0	1	1	16.6	0.04	13.1
1	0	0	100	7.02	2	70	22.7	154.5	82.5	202	54	148	11.5	5.1	71.6	2	1	1	0	1	1	1	5.1	0.02	3.53
1	0	0	100	7.04	2	69	20.4	125.5	73	199	54	145	11.8	5.5	80.5	2	1	1	1	1	1	1	5.5	0.01	10.1
1	0	0	100	5.06	2	76	22.2	126.5	59	172	54	118	12.4	5.2	68.6	2	1	1	0	1	1	1	4.7	0.03	33.5
1	0	0	100	6.97	2	66	28.1	140	76.5	183	54	129	13.1	5.1	77.5	2	1	1	0	1	1	1	23.6	0.08	11.6
1	0	0	100	6.87	2	67	26.9	127	58.5	172	54	118	12.5	6.7	81.7	2	1	1	1	1	0	1	16.1	0.06	5.24
1	0	0	100	6.87	2	69	21.6	116.5	76.5	156	54	102	12	5.5	72.1	2	1	1	0	1	1	1	10	0.02	17
1	0	0	100	6.96	2	72	27.5	141	83	195	54	141	12.4	5	74.3	1	1	0	0	1	1	1	75.4	0.03	13.4
1	0	0	100	5.36	2	70	24	113.5	63.5	175	54	121	12.2	5.3	71.6	2	1	1	1	1	1	1	13.7	0.03	23.8
1	0	0	100	5.37	2	69	21.3	120	81	176	54	122	12.4	4.9	61.4	2	1	1	0	1	1	1	5.7	0.01	42.1
1	0	0	100	5.05	2	74	22.4	128.5	68	209	54	155	12.4	5.7	59.2	1	1	1	1	1	1	1	13.2	0.01	44.1
1	0	0	100	5.06	2	75	24.7	147	81.5	196	54	142	12.2	5.4	72.7	1	1	1	0	1	1	1	28.7	0.09	85.3
1	0	0	100	6.86	2	73	25.2	112	65	206	54	152	13.2	5.2	73.8	1	1	1	0	1	1	1	45.9	0.05	21.9
1	1	0	100	7.04	2	74	22.6	144.5	57.5	239	54	185	11.5	5.5	69.6	1	1	1	0	0	1	1	43.1	0.56	32.9
1	1	0	100	4.62	2	79	25.7	135.5	85	211	54	157	11	5.6	67.2	1	1	1	1	1	1	1	17.1	0.26	23.2
1	1	0	100	6.87	2	73	29.9	168	85	186	54	132	11	7.4	51.7	2	1	1	0	1	0	1	66.4	3.19	18.2
1	1	0	100	5.87	2	76	27.4	142.5	89.5	256	54	202	11	5.3	27.3	2	1	1	0	0	1	1	750	0.08	63.1
1	1	0	100	6.04	2	74	24	119	72.5	212	54	158	11.8	5.2	73.2	1	1	1	1	1	1	1	15.7	0.02	9.89
1	1	0	100	6.4	2	69	24.9	121.5	66.5	189	54	135	12.9	5.1	80.5	2	1	1	0	1	1	1	20.3	0.02	12.2
1	1	0	100	5.06	2	68	27.7	124.5	72.5	244	54	190	13.6	5.7	76.4	2	1	1	1	0	1	1	34	0.04	10.7
1	1	0	100	4.63	2	75	21.9	103.5	60	158	54	104	12.1	5.1	72.7	2	1	1	1	1	1	1	23.7	0.01	37.3
1	1	0	100	5.37	2	73	30.7	151.5	84.5	194	54	140	13.8	5.9	70.1	2	1	1	0	1	1	1	20.9	0.03	14.8
1	1	0	100	6.86	2	74	24.6	116.5	65	209	54	155	15.2	5.7	73.2	2	1	1	1	0	1	1	26.1	0.05	20
1	1	0	100	6.48	1	81	16.6	93.5	59	146	54	92	13.5	6.3	64.9	1	0	0	1	1	1	1	11.9	0.03	70.7
1	0	0	100	4.62	2	80	26.6	135.5	69.5	211	54	157	13.5	6.2	49.3	2	1	0	0	1	1	1	17	0.13	34.1
1	0	1	2.6	2.6	2	70	25.4	118	64.5	213	54	159	14.5	5	60.9	1	1	1	0	1	1	1	22.5	0.21	24.2
1	1	1	5.6	5.6	2	70	17.7	108.5	66.5	187	54	133	14	4.8	75.3	2	1	1	1	1	1	1	9.5	0.03	15.6
1	1	1	3.3	3.3	1	80	20.1	155.5	82	165	54	111	14.6	4.8	72.5	1	1	0	0	1	1	1	1137.8	0.37	122
1	0	1	5.4	5.4	1	71	18.8	125.5	51.5	155	54	101	14.2	5.8	82.2	2	0	1	1	1	1	1	42.8	0.22	252
1	0	1	4.1	4.1	2	69	26.4	159	102	289	54	235	14.7	5.9	80.5	2	1	0	0	0	1	1	175	0.1	10.7
1	1	1	4.7	4.7	2	70	21	140.5	98	187	54	133	12.8	5.4	71.6	2	1	1	0	1	1	1	16.4	0.03	5.97
1	0	0	100	5.16	1	79	30.3	128.5	80	205	55	150	13.6	6.4	69.1	2	1	0	0	1	1	1	3.1	0.1	32.7
1	0	0	100	5.16	1	69	31	110	82	186	55	131	15.1	5.7	62.2	2	1	1	1	1	1	1	7.1	0.17	12.4
1	0	0	100	5.07	1	69	23.2	143.5	73.5	181	55	126	15.7	9.2	78.3	2	1	0	0	1	0	1	64.7	0.14	29.2
1	0	0	100	6.95	1	65	20.1	139.5	75	194	55	139	14.9	5.3	85.8	2	1	0	0	1	1	1	6.6	0.21	44.3
1	0	0	100	4.59	1	77	21.9	128.5	66.5	163	55	108	13.9	5.2	78.8	1	1	0	0	1	1	1	38.7	0.42	14.7
1	0	0	100	5.37	1	66	23.2	141.5	83.5	229	55	174	16.4	5.3	72.1	2	1	1	0	0	1	1	16.9	0.16	26.5
1	0	0	100	6.45	1	76	25.7	108	63	178	55	123	14.5	5.3	70.5	2	0	0	1	1	1	1	9.2	0.19	21.7
1	0	0	100	6.45	1	75	18.7	112.5	65.5	155	55	100	13.8	4.9	75.1	2	1	0	1	1	1	1	28.5	0.27	3.28
1	0	0	100	6.87	1	76	23.3	161	90	192	55	137	15.6	5.2	67.2	2	0	0	0	1	1	1	32.8	0.11	14
1	0	0	100	5.04	1	67	28.2	112.5	73.5	180	55	125	14.9	5.7	84.6	2	1	0	0	1	1	1	12	2.76	7.62
1	0	0	100	7	1	72	26.7	124.5	73.5	210	55	155	14.9	5.5	69.1	2	1	1	1	1	1	1	8.1	0.07	0.05
1	0	0	100	7.07	1	70	19.1	102	58.5	177	55	122	14.2	4.7	77.7	1	0	1	1	1	1	1	8.2	0.02	12.7
1	0	0	100	7.08	1	75	24.7	153.5	84.5	205	55	150	13.7	5	75.1	1	1	0	0	1	1	1	20.2	0.09	16.3
1	0	0	100	7.08	1	75	26.1	152.5	84	202	55	147	16	5.5	67.7	2	1	1	0	1	1	1	15.7	0.05	46.2
1	0	0	100	4.59	1	77	27.5	138	85	235	55	180	15.2	5.7	58.8	2	1	0	1	0	1	1	7.8	0.03	40.7
1	0	0	100	6.87	1	67	18.2	134.5	85	180	55	125	12.6	5.3	63.0	2	1	1	1	1	1	1	17.8	0.05	45.1
1	0	0	100	6.88	1	73	23.7	125.5	64	229	55	174	15.5	4.9	60.4	2	1	1	1	0	1	1	7.8	0.06	11
1	0	0	100	5.16	1	66	21.9	117.5	69	191	55	136	13.4	5.1	75.7	2	1	0	0	1	1	1	6.6	0.08	42.9

1	0	0	100	6.84	1	71	17.8	94	65	183	55	128	14.3	4.9	69.6	2	1	1	1	1	1	1	6.8	0.01	22.1
1	0	0	100	6.86	1	74	19.3	142.5	86.5	167	55	112	13.4	5.4	71.5	2	0	0	0	1	1	1	10	0.01	28.3
1	0	0	100	6.86	1	68	20.3	116.5	65	142	55	87	15	5.2	74.6	2	0	0	0	1	1	1	2.9	0.01	44.4
1	0	0	100	7.01	1	70	23.2	91.5	57.5	205	55	150	13.9	5.6	77.7	2	1	0	1	1	1	1	7.4	0.05	7.13
1	0	0	100	0.72	1	72	24.7	138.5	73.5	216	55	161	13.2	5.2	72.6	1	1	0	1	1	1	1	5.7	0.06	0.98
1	0	0	100	5.05	1	65	20.1	127	78.5	180	55	125	13.1	5.8	76.2	2	1	0	1	1	1	1	4.6	0.02	14.9
1	0	0	100	5.06	1	77	23.2	127.5	75.5	183	55	128	12.6	5.5	74.0	2	1	0	0	1	1	1	4.8	0.05	32.5
1	0	0	100	5.06	1	71	19.3	134.5	84.5	152	55	97	13.3	5.3	73.1	2	1	0	1	1	1	1	218.1	0.01	17.2
1	0	0	100	5.08	1	71	24.8	103.5	69.5	214	55	159	13.5	6.6	82.2	1	1	0	1	1	0	1	13.1	0.02	29.6
1	0	0	100	6.98	1	72	24.2	178.5	98.5	164	55	109	12.4	5.3	81.7	2	1	0	0	1	1	1	35	0.01	26.4
1	0	0	100	4.63	1	66	29.1	125	81.5	165	55	110	13.7	5.5	72.1	2	1	0	1	1	1	1	5.5	0.05	13.3
1	0	0	100	4.64	1	69	21.8	129	80.5	196	55	141	14.1	5.5	70.6	2	1	1	1	1	1	1	12.7	0.04	9.71
1	0	0	100	6.95	1	67	22.1	115.5	63.5	179	55	124	14.1	5.7	79.4	2	0	1	1	1	1	1	20.6	0.02	28.8
1	0	0	100	5.71	1	76	22.7	141	82.5	225	55	170	16.4	5.4	70.5	1	1	0	0	0	1	1	14.1	0.03	6.79
1	0	0	100	6.8	1	70	20.6	109.5	61	234	55	179	15.1	5.5	70.1	2	0	0	1	0	1	1	7.2	0.06	17.7
1	0	0	100	4.6	1	70	24	124	72.5	189	55	134	15.2	5.2	82.8	2	1	0	1	1	1	1	7.4	0.01	43.6
1	0	0	100	4.61	1	73	21	150	90	234	55	179	14.1	5.3	76.1	2	1	0	0	0	1	1	45.8	0.01	14.4
1	0	0	100	6.44	1	68	22.8	128	73	203	55	148	15.1	5.1	74.6	2	1	1	1	1	1	1	7.4	0.03	24
1	0	0	100	6.44	1	66	25	122.5	70.5	136	55	81	14	5.4	72.1	2	1	0	1	1	1	1	5.8	0.03	4.82
1	0	0	100	4.67	1	70	18.2	152	82	182	55	127	14.2	5.1	70.1	2	1	1	0	1	1	1	44.9	0.08	19.2
1	0	0	100	6.96	1	65	26.3	111	69.5	208	55	153	12.8	5.3	76.2	2	1	0	1	1	1	1	85.9	0.06	12
1	0	0	100	5.36	1	67	20	89	53.5	134	55	79	10.7	6.3	75.2	2	0	1	1	1	1	1	5.6	0.01	42
1	0	0	100	6.87	1	75	21.3	127	80.5	144	55	89	12.1	5.5	67.7	2	1	1	1	1	1	1	14.5	0.01	38.6
1	0	0	100	6.87	1	71	25	134.5	73.5	186	55	131	14.6	5.2	77.2	2	1	0	0	1	1	1	14.6	0.08	14.1
1	0	0	100	5.04	1	72	23.4	117	65	152	55	97	13	7.4	76.7	1	1	1	0	1	0	1	12.9	0.02	17.5
1	1	0	100	7	1	70	21.2	114.5	63.5	166	55	111	13.7	6.2	77.7	2	1	0	0	1	1	1	12.3	1.51	29.1
1	1	0	100	2.48	1	76	23.4	126.5	72.5	191	55	136	14.5	6.1	74.5	1	1	1	1	1	1	1	12.5	0.18	21.7
1	0	0	100	7.22	2	71	27.2	135	69.5	183	55	128	11.2	5.5	52.5	1	1	1	1	0	1	1	19.7	1.73	14.7
1	0	0	100	6.87	2	73	27	132	65	195	55	140	13.2	5.3	70.1	2	1	1	1	1	1	1	7.2	0.63	35.8
1	0	0	100	6.86	2	72	25.3	124	70	232	55	177	12.1	5.6	74.3	2	1	1	0	0	1	1	8	0.15	20.4
1	0	0	100	6.98	2	70	22.3	111.5	64.5	216	55	161	14.3	5.6	80.0	2	1	1	1	1	1	1	9.2	0.12	5.78
1	0	0	100	4.64	2	69	23.1	138	84	203	55	148	13.2	5.6	75.9	2	1	1	1	1	1	1	33.3	0.66	13.8
1	0	0	100	6.87	2	75	23.4	112	66	189	55	134	11.5	5.3	69.1	1	1	0	0	1	1	1	19.6	0.13	18.9
1	0	0	100	6.8	2	65	27.2	135	79.5	230	55	175	12.7	5	78.0	2	1	1	0	0	1	1	216.8	3.25	34.4
1	0	0	100	4.62	2	79	23.6	125.5	76	229	55	174	12.9	5.8	70.7	1	1	1	0	0	1	1	61.4	0.13	33
1	0	0	100	6.79	2	69	18.2	145	82	224	55	169	12.1	6.1	75.9	2	1	1	0	0	1	1	4.8	0.64	16.8
1	0	0	100	6.44	2	66	24.8	144	75	196	55	141	13.3	5.4	62.7	2	1	1	0	1	1	1	43.2	0.67	30.4
1	0	0	100	4.67	2	67	24.8	139	78	231	55	176	13.1	5.5	73.1	2	1	1	1	0	1	1	36.8	1.34	8.17
1	0	0	100	6.87	2	76	29	127	67	257	55	202	13.9	6.8	72.2	2	1	1	1	0	0	1	7.9	0.19	30.8
1	0	0	100	5.04	2	65	26.1	178	104.5	305	55	250	14.1	8.2	63.1	2	1	1	0	0	0	1	112.9	0.25	2.14
1	0	0	100	7.05	2	73	30.9	168.5	94.5	215	55	160	14.6	5.2	59.7	2	1	1	0	1	1	1	106.2	0.06	36.2
1	0	0	100	7.05	2	73	25.6	151	79	175	55	120	11.2	5.5	59.7	2	1	1	0	0	1	1	123.2	0.01	46.3
1	0	0	100	7.06	2	67	18.7	162.5	91	232	55	177	12.8	4.8	76.9	1	1	1	0	0	1	1	100.4	0.05	30.9
1	0	0	100	7.08	2	78	28	152	69.5	208	55	153	10.4	5.6	75.6	1	1	1	0	1	1	1	191.2	0.07	41.1
1	0	0	100	4.58	2	68	24.1	125	70.5	260	55	205	13.5	5.8	81.1	2	1	0	1	0	1	1	17.4	0.07	29.7
1	0	0	100	4.58	2	66	21	113	67	248	55	193	14.8	5.4	77.5	2	1	1	1	0	1	1	7.7	0.04	10.3
1	0	0	100	6.86	2	72	27.2	138.5	87.5	200	55	145	13.6	5.7	74.3	1	1	1	0	1	1	1	8.8	0.04	24.3
1	0	0	100	5.16	2	69	21.8	105	62.5	204	55	149	11.3	5.3	61.4	2	1	1	1	1	1	1	8.2	0.03	89.7
1	0	0	100	5.16	2	73	24.6	147.5	74.5	152	55	97	14.2	5.5	73.8	2	1	1	0	0	1	1	45.2	0.02	49.9
1	0	0	100	6.84	2	65	19.7	122.5	70	190	55	135	12.7	5.7	74.2	2	1	0	1	1	1	1	31.2	0.02	28.3
1	0	0	100	6.85	2	73	24.9	117.5	82	245	55	190	13.2	5	73.8	2	1	1	1	0	1	1	9.3	0.03	29.5

1	0	0	100	7.01	2	71	22.2	108.5	59	197	55	142	14.2	5.1	71.1	2	1	1	1	1	1	1	51.1	0.01	18.6
1	0	0	100	7.01	2	66	21.9	114	57	193	55	138	12.2	5.4	77.5	2	0	1	1	1	1	1	16.4	0.04	28.8
1	0	0	100	5.06	2	70	29.9	105.5	56.5	185	55	130	13.8	7.2	75.3	1	1	0	1	0	0	1	73.1	0.09	18.3
1	0	0	100	5.06	2	69	24.8	118	73.5	184	55	129	12.5	6.1	61.4	2	1	1	1	1	1	1	11	0.05	11.7
1	0	0	100	5.06	2	67	24.9	107.5	65	242	55	187	12.8	5.7	81.7	2	1	1	1	0	1	1	21	0.04	3.22
1	0	0	100	6.97	2	76	26.8	141	80	194	55	139	13	5.7	76.7	2	1	1	0	1	1	1	9.7	0.06	15
1	0	0	100	6.87	2	66	29	127.5	73	219	55	164	13.5	6.6	82.3	2	0	1	0	1	0	1	16.3	0.08	3.27
1	0	0	100	6.95	2	69	21	98.5	62	171	55	116	11.1	5.2	80.5	1	1	1	1	1	1	1	41	0.04	19.2
1	0	0	100	6.95	2	69	25	146	80	244	55	189	14.9	5.2	72.1	2	1	1	0	0	1	1	9.2	0.06	20.4
1	0	0	100	6.95	2	65	25.5	130	68.5	207	55	152	15.4	5.5	78.0	2	1	1	1	1	1	1	85.3	0.03	45.4
1	0	0	100	6.96	2	72	25.3	166.5	88.5	184	55	129	13.9	4.8	70.6	2	1	0	0	1	1	1	13	0.05	31.1
1	0	0	100	6.96	2	65	24.4	155	89	243	55	188	14	5.4	78.0	2	1	1	0	0	1	1	17	0.02	39.2
1	0	0	100	6.98	2	65	23.1	120	74	243	55	188	14.1	5.3	63.1	2	1	1	1	0	1	1	18	0.03	3.56
1	0	0	100	6.8	2	65	28.7	211	104	251	55	196	13.2	6.1	78.0	1	1	0	0	0	1	1	6.4	0.07	25.2
1	0	0	100	6.8	2	67	23	128.5	79	229	55	174	14.2	5.5	54.0	2	1	1	1	0	1	1	15.4	0.04	9.12
1	0	0	100	6.8	2	65	16.8	146.5	77.5	209	55	154	14.1	5.1	74.2	2	1	1	0	1	1	1	28	0.07	56.3
1	0	0	100	6.84	2	65	18.6	135.5	79	199	55	144	12.2	4.6	63.1	2	1	1	1	1	1	1	16.2	0.03	54.7
1	0	0	100	6.79	2	69	28.2	159	94	312	55	257	14	5.2	72.1	1	1	1	0	0	1	1	13	0.04	4.08
1	0	0	100	4.68	2	73	29.3	124.5	67.5	186	55	131	14.3	5.4	78.3	1	1	1	1	1	1	1	6	0.02	55.9
1	0	0	100	5.11	2	70	23.6	134	75	212	55	157	13.5	5.6	75.3	2	1	1	1	1	1	1	8.4	0.02	19.7
1	0	0	100	4.69	2	70	26	147.5	83	176	55	121	14.7	6.8	80.0	1	1	1	0	1	0	1	41.5	0.04	15.3
1	0	0	100	5.36	2	69	22.5	108.5	62.5	174	55	119	12.8	5.5	75.9	2	1	1	1	1	1	1	28.7	0.02	9.26
1	0	0	100	5.37	2	70	27.3	136	75	224	55	169	13.5	5.6	80.0	1	1	1	1	0	1	1	14.5	0.02	26.3
1	0	0	100	5.37	2	71	26	108.5	57.5	202	55	147	13.1	6.3	74.8	1	1	1	0	1	1	1	32.1	0.09	91.4
1	0	0	100	5.06	2	72	23	113.5	66	181	55	126	12.2	5.8	74.3	2	1	1	1	1	1	1	12.6	0.02	17.5
1	0	0	100	5.06	2	70	22.1	95.5	55	153	55	98	10.6	5.6	75.3	2	1	1	1	1	1	1	36	0.01	36.2
1	0	0	100	6.85	2	69	20.9	102.5	57.5	232	55	177	13.6	5.7	72.1	2	1	1	1	0	1	1	20.7	0.02	24.8
1	0	0	100	6.87	2	69	23.6	161	89	211	55	156	13.6	5.1	80.5	2	1	1	0	1	1	1	23.1	0.06	8.05
1	0	0	100	6.87	2	72	23.6	120.5	69.5	207	55	152	11.2	5.6	78.9	2	1	1	1	0	1	1	41.7	0.03	34
1	0	0	100	6.87	2	65	26.3	108	62.5	198	55	143	12.1	6.1	78.0	2	1	1	1	1	1	1	12.8	0.02	27.6
1	0	0	100	5.04	2	66	23.8	141	77.5	170	55	115	13.1	5.3	77.5	2	1	1	0	1	1	1	53.6	0.02	20.4
1	0	0	100	5.04	2	79	20.8	141	72.5	200	55	145	12.1	5	57.2	2	1	1	0	1	1	1	21.9	0.08	37.3
1	0	0	100	5.04	2	66	24.7	118.5	72.5	161	55	106	13.3	5.4	82.3	2	1	0	1	1	1	1	10.8	0.04	28.5
1	1	0	100	4.58	2	78	18.6	111.5	63	280	55	225	12.7	5.3	71.2	1	1	1	1	0	1	1	27.7	0.02	18.8
1	1	0	100	6.87	2	74	24.3	145.5	75	219	55	164	12.6	5.7	73.2	1	1	1	0	1	1	1	27.4	0.08	97.8
1	1	0	100	6.87	2	70	23.9	146.5	75.5	225	55	170	14.9	5	80.0	2	1	1	0	0	1	1	12.6	0.01	23
1	1	0	100	6.87	2	68	24.2	140.5	79	170	55	115	11.7	4.9	72.6	1	1	1	0	1	1	1	6.9	0.04	35.9
1	1	0	100	4.63	2	71	21.3	106.5	64.5	212	55	157	11.1	5.2	74.8	2	1	1	1	1	1	1	16.2	0.01	30.3
1	1	0	100	4.75	2	75	23	93.5	52.5	213	55	158	11.6	5.7	69.1	1	1	1	1	1	1	1	9.8	0.01	54.5
1	0	0	100	1	1	80	24.3	157.5	78.5	207	55	152	14.3	5	72.5	2	1	0	0	1	1	1	21.3	0.1	103
1	0	0	100	7	1	82	20.8	137.5	83	156	55	101	12.1	5.5	71.5	1	1	0	1	1	1	1	6.9	0.15	58.9
1	1	0	100	3.49	1	82	23.1	131	81	201	55	146	14.9	5.2	67.6	2	1	1	1	1	1	1	89.7	0.12	96.7
1	1	0	100	6.77	1	82	20.9	145.5	64.5	183	55	128	12.6	6.7	76.1	2	0	1	0	1	0	1	8.7	0.06	33.6
1	0	0	100	1.64	2	82	20.3	141	69	151	55	96	11.9	5.7	73.5	1	1	1	0	1	1	1	33.9	0.14	62.7
1	0	0	100	1.49	2	81	23.3	124	62	238	55	183	13	5.4	69.7	2	1	1	0	0	1	1	11.2	0.05	61
1	0	0	100	5.06	1	76	24.3	111.5	70	138	55	83	13.1	5.5	79.4	2	1	0	1	1	1	1	6.2	0.11	11.3
1	0	0	100	4.64	1	77	19.3	118	64	170	55	115	13.4	5.9	70.1	2	1	0	1	1	1	0	109.6	8.28	173
1	0	0	100	7.21	1	74	25.6	116.5	67.5	171	55	116	14	4.9	75.6	2	0	0	0	1	1	1	4.9	0.03	9.44
1	0	0	100	4.64	1	65	28.1	151	80	201	55	146	15	5.6	76.2	2	1	1	0	1	1	1	16.2	0.04	16.8
1	0	0	100	6.96	1	65	20.7	105.5	68	169	55	114	13.2	5.5	80.5	2	1	0	1	1	1	1	14.2	0.02	17.5
1	0	0	100	4.62	1	70	21.5	147.5	88.5	195	55	140	15.3	5.4	77.7	2	0	1	0	1	1	1	23.1	0.07	13.7

1	0	0	100	5.04	1	71	30.4	130.5	72.5	205	55	150	14.3	5.5	77.2	2	1	1	1	1	1	1	5.8	0.04	6.35
1	1	0	100	4.59	1	73	22.1	123.5	75.5	214	55	159	13.4	5.1	72.0	2	1	0	1	1	1	1	7.4	0.02	35.6
1	1	0	100	5.07	1	78	17.9	151	83	130	55	75	15.8	5.6	73.5	2	0	0	0	1	1	1	1645.1	0.04	15.5
1	0	0	100	7.07	2	65	21.3	104	63.5	224	55	169	12.5	5.4	74.2	2	1	1	1	0	1	1	14.4	0.1	5.91
1	0	0	100	6.86	2	65	34.6	159.5	83.5	208	55	153	12.7	7.1	78.0	2	1	1	0	1	0	1	12.2	0.13	18.7
1	0	0	100	7.04	2	67	25.4	142	65.5	203	55	148	13.4	5.5	76.9	2	1	1	0	1	1	1	18.9	0.26	75.6
1	0	0	100	5.08	2	72	26	119.5	73.5	175	55	120	12.5	5.7	78.9	2	1	1	0	1	1	1	72.6	0.11	152
1	0	0	100	7	2	74	28	118.5	80	207	55	152	14.1	5.4	73.2	1	1	1	0	1	1	1	6.8	0.14	23.5
1	0	0	100	4.62	2	70	28.7	162	91	206	55	151	14.1	5.5	71.6	2	1	1	0	1	1	1	106.7	0.19	36.7
1	0	0	100	6.8	2	66	20	109.5	69	206	55	151	14.9	5.2	82.3	2	1	1	1	1	1	1	9.4	0.15	0.96
1	0	0	100	4.62	2	67	26.2	133.5	85.5	203	55	148	11.9	5.7	76.9	2	1	1	1	1	1	1	72.4	1.56	59.4
1	0	0	100	4.69	2	68	25.4	163	79	213	55	158	11.5	5.1	72.6	2	1	1	0	1	1	1	18	0.2	40.6
1	0	0	100	6.85	2	67	20.7	144	84.5	208	55	153	12.3	5.4	76.9	2	1	1	0	1	1	1	77.2	0.14	10
1	0	0	100	7.07	2	69	27.9	150.5	79	187	55	132	12.5	5.5	72.1	2	1	0	0	0	1	1	12.7	0.03	24
1	0	0	100	4.58	2	74	29	126.5	77	163	55	108	12.1	5.5	77.8	2	1	1	1	0	1	1	19.6	0.01	24.7
1	0	0	100	6.86	2	65	25.7	155.5	93	219	55	164	13.2	5	63.1	2	1	1	0	1	1	1	83.4	0.03	25.5
1	0	0	100	5.15	2	65	25.2	131.5	85	218	55	163	13.2	5.6	74.2	2	1	1	0	1	1	1	33.7	0.02	51.7
1	0	0	100	5.16	2	71	26.3	140.5	79.5	207	55	152	12.4	5.5	74.8	1	1	1	0	1	1	1	42.9	0.05	102
1	0	0	100	6.85	2	75	22.4	140	76.5	254	55	199	15	4.9	69.1	1	1	1	0	0	1	1	15	0.07	21
1	0	0	100	7.02	2	71	20.7	130.5	73	271	55	216	12	5.3	71.1	1	1	1	1	0	1	1	14.2	0.03	22.5
1	0	0	100	0.18	2	69	21.2	158	88	193	55	138	12.4	5.3	75.9	2	1	1	0	1	1	1	14.8	0.01	12.9
1	0	0	100	7.04	2	65	28.8	142	82	200	55	145	12.2	5.1	82.8	2	1	1	0	1	1	1	16.6	0.02	20.6
1	0	0	100	6.98	2	76	20.4	133	68.5	206	55	151	12.8	5.4	76.7	1	1	1	1	1	1	1	14.3	0.03	0.84
1	0	0	100	7	2	77	18.3	123.5	75	227	55	172	11.9	4.9	68.2	1	1	1	1	0	1	1	13.7	0.01	23.6
1	0	0	100	7	2	72	26.4	112.5	65	192	55	137	12.6	5.6	60.1	1	1	1	1	1	1	1	9.9	0.05	22.8
1	0	0	100	7	2	72	23.3	139	74	193	55	138	12.7	6.4	60.1	1	1	1	1	1	1	1	11.3	0.02	9.64
1	0	0	100	6.95	2	77	24.7	108.5	64.5	177	55	122	11.6	5	76.1	2	1	1	1	1	1	1	16.4	0.05	9.72
1	0	0	100	6.83	2	70	22.6	113	69	156	55	101	12	4.9	80.0	1	1	1	1	1	1	1	18.9	0.01	49.8
1	0	0	100	4.59	2	76	19.4	100	64	166	55	111	12.7	6.5	76.7	1	1	1	1	1	0	1	28.9	0.01	28.8
1	0	0	100	4.62	2	68	21.9	113.5	66	178	55	123	12.6	5.4	72.6	2	1	1	1	1	1	1	14.2	0.01	9.37
1	0	0	100	4.62	2	79	22.9	95	53.5	164	55	109	11.9	6	75.1	1	1	1	1	1	1	1	12.2	0.02	12
1	0	0	100	5.05	2	72	22.4	136	81.5	161	55	106	13.3	5.5	60.1	2	1	1	0	1	1	1	2.9	0.08	16.7
1	0	0	100	6.86	2	70	24.6	126	77.5	190	55	135	11.3	5.9	75.3	2	1	1	1	1	1	1	71.3	0.02	13.9
1	0	0	100	6.87	2	72	25.1	158	78.5	167	55	112	13	5.4	74.3	2	1	1	0	1	1	1	22.5	0.03	24.2
1	0	0	100	5.04	2	70	23.9	137.5	67	180	55	125	11.3	5.5	80.0	2	1	1	1	1	1	1	5.4	0.01	34.9
1	1	0	100	6.86	2	78	24.2	115.5	79	211	55	156	13.8	5.4	67.7	1	1	1	0	1	1	1	9.9	0.13	24.1
1	1	0	100	7	2	78	22.6	134	69	199	55	144	13.2	4.9	71.2	1	1	1	0	1	1	1	37.4	0.07	5.01
1	1	0	100	7.07	2	79	26.5	106.5	67	289	55	234	13.9	5.6	67.2	1	1	1	1	0	1	1	11.1	0.04	39.1
1	1	0	100	4.58	2	77	29.7	122	67.5	206	55	151	13.7	6.6	58.0	1	1	0	0	1	0	1	250	0.03	16.9
1	1	0	100	7.01	2	70	28.3	152.5	86.5	197	55	142	12.7	6	80.0	2	1	0	0	1	1	1	58.9	0.03	27.2
1	1	0	100	6.86	2	71	23.1	152	79	218	55	163	10.1	5	60.5	1	1	1	0	1	1	1	61.1	0.01	59.3
1	0	0	100	4.63	1	81	23.2	150	89	222	55	167	16	5.6	50.9	2	1	1	0	0	1	1	8.3	0.04	6.29
1	1	0	100	4.63	1	81	22.2	134	73	168	55	113	12.8	5.4	57.1	2	1	1	1	1	1	1	20.2	0.07	38.8
1	0	0	100	6.86	2	81	25.7	132.5	67	167	55	112	14.8	5	66.3	2	1	1	1	0	1	1	75.4	0.26	16.8
1	0	0	100	2.34	2	83	24.2	150.5	85.5	178	55	123	14.2	5.3	55.6	1	1	1	0	1	1	1	11.8	0.27	362
1	0	0	100	6.85	2	80	21.1	147.5	77.5	171	55	116	12.9	5.3	56.8	2	1	1	0	1	1	1	10.7	0.03	37.8
1	1	0	100	2.57	2	83	24.2	122.5	74	196	55	141	13.8	5.4	68.7	2	1	1	0	1	1	1	88.6	0.02	82
1	0	1	5.2	5.2	1	76	21.9	126.5	82	213	55	158	14.7	5.9	67.2	2	0	0	1	1	1	1	11.4	0.28	84.7
1	0	1	2	2	2	71	24.5	152.5	89	208	55	153	14.1	5.5	74.8	2	1	1	0	1	1	1	11.6	0.02	2.5
1	1	1	3.6	3.6	2	66	20.6	148.5	76	205	55	150	12.9	5.4	77.5	2	1	1	0	1	1	1	5.6	0.28	58.2
1	1	1	4.1	4.1	2	69	20.4	113.5	63.5	207	55	152	11.3	5.4	72.1	1	1	1	1	1	1	1	4	0.01	53.6

1	0	1	4.3	4.3	2	74	24.3	131.5	81.5	246	55	191	12.1	5.5	73.2	2	1	1	1	0	1	1	14.1	0.13	5.77
1	0	0	100	4.59	1	76	27.5	120.5	77.5	172	56	116	13.8	5.5	59.2	2	1	0	0	1	1	1	11.9	0.2	11.8
1	0	0	100	6.87	1	65	26.6	135	85	198	56	142	15.2	5.6	72.6	2	0	0	0	1	1	1	4.7	0.12	30.9
1	0	0	100	6.88	1	75	23.5	141	72	168	56	112	12.3	5.2	80.0	2	1	0	0	1	1	1	592.3	0.22	104
1	0	0	100	5.05	1	69	27.5	145	89.5	183	56	127	14.3	5.2	78.3	2	1	0	0	1	1	1	78.1	0.27	26.2
1	0	0	100	5.05	1	68	27.1	137	79	147	56	91	14.8	5.2	78.8	2	1	0	1	1	1	1	20.2	0.12	7.41
1	0	0	100	4.64	1	71	33.3	132	74.5	183	56	127	15.2	5.4	82.2	1	1	0	0	1	1	1	87.6	0.3	65.8
1	0	0	100	6.96	1	68	27.7	133.5	81.5	248	56	192	15.7	5.5	78.8	2	1	0	1	0	1	1	11.7	0.16	4.78
1	0	0	100	6.44	1	67	26.2	131	83	194	56	138	15.9	5.1	79.4	2	0	0	1	1	1	1	68.9	1.14	17.4
1	0	0	100	4.67	1	69	26.5	132	80	209	56	153	12.6	8.4	70.6	2	1	1	1	1	0	1	18.8	0.17	8.72
1	0	0	100	5.05	1	69	18.4	170.5	110	134	56	78	14.2	5.2	70.6	2	0	0	0	1	1	1	13.7	0.16	117
1	0	0	100	5.04	1	78	24.9	127.5	71.5	195	56	139	14.9	6.2	66.3	2	1	0	0	1	1	1	32.9	0.71	39.4
1	0	0	100	7.07	1	73	20.8	131.5	69	145	56	89	15.2	5.4	72.0	1	0	1	1	1	1	1	22.6	0.03	0.05
1	0	0	100	2.71	1	78	20.7	135	83.5	164	56	108	13.1	5.8	69.6	2	1	1	1	1	1	1	12.3	0.04	84.5
1	0	0	100	7.08	1	66	22.3	111.5	67	144	56	88	14.9	4.4	79.9	2	1	0	0	1	1	1	12.6	0.01	5.3
1	0	0	100	4.58	1	72	19	112	65.5	181	56	125	15.1	5.2	76.7	2	1	0	1	1	1	1	15.5	0.02	17.1
1	0	0	100	6.87	1	76	19.6	142	81	197	56	141	13.6	5	74.5	2	1	0	0	1	1	1	8.9	0.04	64
1	0	0	100	7.2	1	70	24.1	100.5	65	186	56	130	14.4	5.5	73.6	2	0	1	1	1	1	1	20.6	0.03	8.62
1	0	0	100	1.71	1	66	22.5	134	80.5	211	56	155	12.5	5.4	72.1	2	1	0	1	1	1	1	4	0.01	23.3
1	0	0	100	5.05	1	71	27.1	118.5	68	201	56	145	13.9	5	77.2	2	1	0	1	1	1	1	15.1	0.06	195
1	0	0	100	5.06	1	68	23.2	126	69	229	56	173	13.5	5.3	74.6	2	1	1	1	0	1	1	17	0.08	20.3
1	0	0	100	5.08	1	75	25.3	149	82	186	56	130	14	5.6	75.1	2	1	0	0	1	1	1	8	0.01	22.3
1	0	0	100	6.98	1	71	19.7	131	73	189	56	133	14.5	5.5	82.2	2	0	1	1	1	1	1	10.9	0.05	22.6
1	0	0	100	4.63	1	74	21.7	111.5	69.5	173	56	117	13	5.4	71.5	2	0	0	1	1	1	1	10.5	0.03	15.7
1	0	0	100	4.63	1	76	21.3	112	63.5	171	56	115	11.3	5.3	67.2	2	1	0	1	1	1	1	10.9	0.02	70.3
1	0	0	100	4.63	1	74	19	101.5	55.5	167	56	111	13.3	5.5	68.2	2	0	0	1	1	1	1	6.6	0.07	24.6
1	0	0	100	3.78	1	65	23.3	122	72.5	186	56	130	14.3	4.6	76.2	2	0	0	1	1	1	1	7.9	0.04	9.54
1	0	0	100	6.95	1	70	22.3	144.5	73.5	132	56	76	13.5	4.6	82.8	1	1	0	0	1	1	1	24.1	0.09	54.2
1	0	0	100	6.79	1	76	25.7	112	68.5	152	56	96	15.9	5.9	59.2	2	1	1	0	1	1	1	4.1	0.07	13.9
1	0	0	100	6.8	1	69	26.6	102	58.5	184	56	128	15.5	5.9	78.3	2	1	0	1	1	1	1	63.5	0.04	70.9
1	0	0	100	4.67	1	66	24	147.5	90.5	219	56	163	15.5	6	91.8	2	1	0	0	1	1	1	294	0.06	12.8
1	0	0	100	4.69	1	67	21.7	112.5	73.5	203	56	147	15.9	4.9	75.2	2	0	0	1	1	1	1	6.1	0.07	7.19
1	0	0	100	6.97	1	66	23.9	125	83.5	211	56	155	14.2	5.8	75.7	2	1	1	1	1	1	1	16.4	0.04	16.9
1	0	0	100	5.37	1	71	28.7	123.5	73	181	56	125	12.1	5.6	73.1	1	1	0	0	1	1	1	8.6	0.05	50.7
1	0	0	100	2.22	1	68	20.5	135.5	74	168	56	112	14.7	5.5	62.6	1	0	0	1	1	1	1	10	0.02	63
1	0	0	100	6.87	1	73	24	114.5	70	242	56	186	13.9	5.4	53.9	2	1	0	0	0	1	1	5.3	0.03	41.7
1	1	0	100	1.86	1	71	29	116	75.5	204	56	148	15.1	5.4	82.2	2	1	1	1	1	1	1	3.5	0.11	10.2
1	0	0	100	1.9	2	71	30.7	143.5	83	256	56	200	13.3	5.3	74.8	2	1	1	0	0	1	1	401.2	0.36	7.47
1	0	0	100	6.86	2	75	21	111	64	222	56	166	11	5.2	72.7	2	1	1	1	0	1	1	163.8	1.6	28.6
1	0	0	100	6.87	2	68	21.3	110	62	192	56	136	13.4	5.4	81.1	2	1	1	1	1	1	1	5.8	0.31	74
1	0	0	100	6.87	2	65	22.8	119	70	217	56	161	12	5.2	78.0	2	1	1	1	1	1	1	26.6	0.13	16.7
1	0	0	100	6.87	2	72	29.7	171.5	89.5	208	56	152	11.5	5.9	78.9	2	1	1	0	1	1	1	8.8	1.72	39
1	0	0	100	7.2	2	66	19.3	105.5	65	167	56	111	12.2	9.8	73.6	2	1	1	1	1	0	1	14.1	1.6	5.57
1	0	0	100	6.85	2	69	18.6	99.5	64	159	56	103	12	5.5	75.9	2	1	1	1	1	1	1	21.6	0.19	22.3
1	0	0	100	7.04	2	65	22.7	121	75	258	56	202	11.1	5.5	78.0	2	1	1	1	0	1	1	5.4	0.17	11.2
1	0	0	100	6.84	2	70	18.4	125	83.5	179	56	123	12.6	5.5	75.3	2	1	1	1	1	1	1	23.1	0.12	6.55
1	0	0	100	4.62	2	65	25.4	176	89	290	56	234	14.1	5.4	78.0	2	1	1	0	0	1	1	15.7	0.17	18.1
1	0	0	100	4.7	2	78	23.2	127.5	74	189	56	133	11.8	5.4	75.6	1	1	1	1	1	1	1	31.7	1.16	38.9
1	0	0	100	6.87	2	65	24.1	110	61	256	56	200	13.4	5.4	82.8	2	1	1	1	0	1	1	17.3	0.11	7.41
1	0	0	100	5.04	2	66	24	134.5	81.5	217	56	161	12.8	5.1	77.5	2	1	1	0	1	1	1	142.7	0.45	49.3
1	0	0	100	7	2	72	24.1	131	66	162	56	106	11.1	4.9	74.3	1	1	0	1	1	1	1	29.7	0.03	174

1	0	0	100	7.07	2	67	18.6	135.5	77	178	56	122	9.8	5.6	73.1	2	1	1	1	1	1	1	93.9	0.06	22.6
1	0	0	100	7.08	2	66	31.6	148	81.5	212	56	156	13.6	6.9	77.5	2	1	1	0	1	0	1	45.2	0.08	2.66
1	0	0	100	7.08	2	72	25.7	123	75.5	179	56	123	14	4.9	78.9	2	1	1	0	1	1	1	19.7	0.06	27.3
1	0	0	100	4.59	2	67	26.2	151.5	80	263	56	207	13.7	5.5	76.9	2	1	1	0	0	1	1	24.5	0.08	14.3
1	0	0	100	6.86	2	74	24	137	77	225	56	169	12	5.2	59.2	2	1	1	1	0	1	1	28.1	0.04	26.9
1	0	0	100	6.87	2	70	22.4	126	71	219	56	163	14	5.7	71.6	1	1	1	1	1	1	1	18.7	0.04	11.2
1	0	0	100	6.87	2	72	20.2	150.5	89.5	241	56	185	13.2	5.9	74.3	2	0	1	0	0	1	1	11.3	0.03	51.2
1	0	0	100	6.87	2	75	19.1	168.5	93.5	247	56	191	12.1	9.8	77.2	2	1	1	0	0	0	1	209.1	0.02	29.1
1	0	0	100	6.87	2	69	37.4	150	89	228	56	172	16	5	80.5	1	1	1	0	0	1	1	73.4	0.05	7.06
1	0	0	100	6.88	2	77	20.1	142.5	77	201	56	145	10.8	5.2	71.7	2	1	1	0	1	1	1	19.1	0.05	28.2
1	0	0	100	5.16	2	70	19.4	130.5	73	200	56	144	12.3	5.4	75.3	2	1	1	0	1	1	1	119.3	0.09	18.4
1	0	0	100	5.16	2	65	27	148	87	208	56	152	14.6	5.1	78.0	2	1	1	0	1	1	1	84	0.03	19.1
1	0	0	100	5.16	2	65	32.1	121	70.5	212	56	156	13.3	6.4	54.7	2	1	1	0	1	1	1	639.9	0.03	13
1	0	0	100	6.84	2	69	23.1	137.5	74	193	56	137	14.4	5.5	80.5	2	1	1	1	1	1	1	13.1	0.05	10.5
1	0	0	100	6.85	2	65	24.8	135.5	84.5	250	56	194	13.1	5.8	82.8	2	1	1	1	0	1	1	13.2	0.07	28.1
1	0	0	100	6.86	2	65	27.9	156.5	89.5	243	56	187	13.3	5.3	89.1	2	1	1	0	0	1	1	29.6	0.01	43.5
1	0	0	100	7	2	78	18.1	120	82.5	154	56	98	12.3	5	67.7	1	1	1	1	1	1	1	12.8	0.01	36.7
1	0	0	100	7.01	2	67	24.3	113	63.5	219	56	163	12	5.5	76.9	1	1	1	1	1	1	1	5.8	0.05	17.3
1	0	0	100	7.03	2	65	25.6	136.5	85.5	200	56	144	13.1	5	74.2	2	1	0	1	1	1	1	7.9	0.07	13.3
1	0	0	100	7.03	2	69	25	122	63.5	158	56	102	11.2	5.3	80.5	1	1	1	1	1	1	1	42.1	0.09	40.4
1	0	0	100	5.05	2	71	26.2	136	81	221	56	165	12.3	5.9	79.4	1	1	1	1	0	1	1	6.8	0.08	31.3
1	0	0	100	5.05	2	67	23.9	115	63	148	56	92	14.4	4.6	76.9	2	1	1	0	1	1	1	27.6	0.05	5.58
1	0	0	100	5.06	2	72	29	121.5	63.5	186	56	130	12.2	5.4	74.3	2	1	1	0	1	1	1	21.1	0.03	100
1	0	0	100	6.97	2	67	25.4	110.5	61.5	234	56	178	12.3	5.3	73.1	2	1	1	1	0	1	1	193.8	0.06	67.7
1	0	0	100	6.97	2	67	20.9	104.5	64	166	56	110	13.5	5.5	73.1	2	1	1	1	1	1	1	148.2	0.06	14.4
1	0	0	100	4.63	2	73	19.6	104.5	55	222	56	166	12.2	5.6	70.1	2	1	1	1	0	1	1	11.2	0.04	62.8
1	0	0	100	4.63	2	69	23.8	124	68.5	188	56	132	12.1	5.4	75.9	2	1	1	1	0	1	1	10.3	0.01	15.9
1	0	0	100	4.64	2	69	25.6	106	69	238	56	182	13.3	5.1	75.9	1	1	1	1	0	1	1	77.4	0.03	14.4
1	0	0	100	6.95	2	72	20.2	157	73.5	163	56	107	11.6	5.4	78.9	2	1	1	0	1	1	1	22.4	0.01	108
1	0	0	100	6.96	2	75	20.9	105	60	222	56	166	10	5	77.2	1	1	1	1	0	1	1	24.8	0.01	0.05
1	0	0	100	6.98	2	67	23.8	111	69.5	229	56	173	13.1	5.2	73.1	1	1	0	1	0	1	1	16.5	0.04	12.1
1	0	0	100	6.98	2	67	29.5	129.5	79	189	56	133	13.4	5.1	81.7	2	1	1	1	1	1	1	45.6	0.08	65.7
1	0	0	100	6.8	2	73	25.6	145	76	177	56	121	13.4	5.7	73.8	2	1	1	0	1	1	1	10.6	0.04	13.8
1	0	0	100	6.83	2	69	27.9	128	74.5	207	56	151	10.8	5.4	72.1	2	1	1	0	1	1	1	31.8	0.06	136
1	0	0	100	6.83	2	69	23.9	103	73	216	56	160	13.1	5.9	72.1	2	1	1	1	1	1	1	17.3	0.04	20.1
1	0	0	100	6.83	2	70	22.8	101	66	173	56	117	13.2	5.4	71.6	2	1	1	1	1	1	1	11	0.03	11.2
1	0	0	100	6.84	2	67	21.5	119	70	196	56	140	13.2	5.5	73.1	2	1	1	1	1	1	1	13.3	0.03	9.05
1	0	0	100	4.59	2	76	22.8	117.5	68.5	181	56	125	12.1	5.3	68.6	1	1	1	0	1	1	1	8	0.03	74.1
1	0	0	100	4.59	2	66	24.3	128.5	70.5	238	56	182	12.8	5.8	73.6	2	1	1	1	0	1	1	46.2	0.04	7.81
1	0	0	100	4.6	2	78	30.3	130.5	59.5	211	56	155	12.5	6.5	71.2	1	1	1	1	0	0	1	8.2	0.03	80.1
1	0	0	100	4.61	2	69	30.5	143.5	91	262	56	206	14.5	5.7	72.1	2	1	1	0	0	1	1	24.1	0.09	47.1
1	0	0	100	4.61	2	69	24.9	140.5	69.5	241	56	185	14.6	5	75.9	1	1	1	0	0	1	1	20.2	0.02	104
1	0	0	100	4.61	2	79	23.4	128	72	202	56	146	12.4	6.1	70.7	1	1	1	1	1	1	1	35.3	0.07	15.6
1	0	0	100	4.62	2	66	31.8	115	61	209	56	153	14	5.2	73.6	2	1	1	0	1	1	1	5.3	0.05	26.4
1	0	0	100	4.62	2	69	24.4	136.5	70	200	56	144	14.5	5.5	75.9	2	1	1	0	0	1	1	3.5	0.06	42.3
1	0	0	100	4.67	2	66	25.9	107.5	72	218	56	162	12.6	5.4	73.6	2	1	1	1	0	1	1	9.4	0.02	13.2
1	0	0	100	4.69	2	68	22.2	121.5	83.5	215	56	159	13	5.6	76.4	2	1	0	0	1	1	1	16	0.03	3.07
1	0	0	100	4.7	2	66	23.2	131.5	72	199	56	143	13	5.4	77.5	2	1	1	1	1	1	1	11.7	0.03	27.2
1	0	0	100	6.97	2	74	23.9	100	64.5	199	56	143	12.8	5.4	73.2	1	1	1	1	1	1	1	8.8	0.03	0.05
1	0	0	100	5.36	2	71	26.4	138	76	180	56	124	12.3	5	79.4	1	1	1	1	1	1	1	24.3	0.04	57.5
1	0	0	100	6.87	2	66	18.8	105	65.5	208	56	152	12.6	4.9	47.8	2	1	0	1	1	1	1	5.7	0.01	21

1	0	0	100	5.04	2	66	19.3	123.5	85	170	56	114	14.9	5.2	54.3	2	1	1	0	1	1	1	12.6	0.08	39.2
1	0	0	100	5.04	2	68	21.2	139.5	78.5	184	56	128	14	5.1	72.6	2	1	1	0	1	1	1	31.3	0.07	8.12
1	1	0	100	5.04	2	76	21.8	125.5	71.5	264	56	208	12.3	5.6	72.2	1	1	1	1	0	1	1	14.5	0.32	58.4
1	1	0	100	5.06	2	75	25.3	129	73	203	56	147	13.7	7.1	58.8	2	1	1	0	1	0	1	22.6	0.11	120
1	1	0	100	6.87	2	72	28.2	140	82	243	56	187	13	6.2	78.9	2	1	1	0	0	1	1	87.3	0.33	7.46
1	1	0	100	6.86	2	76	24.9	144.5	83.5	259	56	203	14.2	5.3	68.6	2	1	1	0	0	1	1	23.3	0.03	3.92
1	1	0	100	7.21	2	70	20.8	122.5	84	199	56	143	12.3	6	75.3	2	1	1	1	1	1	1	12	0.04	15.3
1	1	0	100	7.04	2	65	28.4	145	81	224	56	168	14.3	6.1	78.0	1	1	1	0	0	1	1	17.3	0.09	4.83
1	1	0	100	4.59	2	79	22.2	150	85.5	188	56	132	12	5.3	75.1	1	1	1	0	1	1	1	52.4	0.02	18
1	0	0	100	4.59	1	84	24.3	111.5	75	199	56	143	15.9	6	63.5	2	1	1	1	1	1	1	13.2	0.07	16.9
1	0	0	100	7.08	2	81	26.2	138	78	220	56	164	13.6	5.4	74.0	1	1	1	1	0	1	1	30.8	0.1	23.7
1	0	0	100	4.59	2	80	27.4	145	89.5	259	56	203	14.8	6	74.6	2	1	1	0	0	1	1	127.9	0.07	46.3
1	1	0	100	6.87	2	87	19.4	141	67.5	178	56	122	10.3	5.5	46.9	2	1	1	0	1	1	1	9.3	0.1	69.8
1	0	0	100	6.95	1	77	25	142	78.5	200	56	144	14.7	7.6	70.1	2	1	0	0	1	0	1	1040.2	0.28	45.4
1	0	0	100	6.8	1	78	23.6	171.5	87.5	245	56	189	14.7	5.7	58.3	2	1	0	0	0	1	1	14.7	0.24	21
1	0	0	100	6.44	1	65	21.4	167.5	106.5	186	56	130	16.9	5.3	72.6	2	1	1	0	1	1	1	17.1	0.27	3.4
1	0	0	100	6.86	1	75	23.4	121.5	68	198	56	142	14	5.2	47.8	2	1	0	0	1	1	1	16.6	0.06	28.8
1	0	0	100	6.97	1	65	23.3	125	85	150	56	94	14.6	5.1	80.5	2	0	0	0	1	1	1	11.6	0.01	2.13
1	0	0	100	5.04	1	70	23.1	152.5	84.5	158	56	102	14.5	4.9	77.7	2	1	0	0	1	1	1	8.9	0.06	26
1	1	0	100	4.64	1	71	23.1	131	82	140	56	84	12.9	6.2	54.6	2	1	1	0	1	0	0	72.2	0.01	182
1	0	0	100	7	2	69	29.2	129	78	219	56	163	14.3	7.2	75.9	1	1	1	0	1	0	1	138.3	0.24	13.7
1	0	0	100	6.85	2	74	22.9	136.5	74	204	56	148	13.7	4.9	51.4	2	1	1	0	0	1	1	7.9	0.2	22.7
1	0	0	100	4.63	2	69	29.4	160	83.5	226	56	170	14.1	5.8	80.5	2	1	1	0	0	1	1	36.2	0.16	25.1
1	0	0	100	6.87	2	77	31.1	157	80.5	202	56	146	12	5.4	58.0	1	1	1	0	1	1	1	55.8	0.12	1.19
1	0	0	100	6.86	2	66	23.9	115	64	203	56	147	13	5.5	82.3	1	1	1	1	1	1	1	84.1	0.52	10.6
1	0	0	100	5.04	2	72	30	126	69	185	56	129	14	6.7	78.9	2	1	1	0	1	0	1	11.7	0.12	19.9
1	0	0	100	5.04	2	77	28.1	113	59	220	56	164	12.1	6.9	58.0	2	1	1	0	0	0	1	10.3	0.24	65
1	0	0	100	5.04	2	72	21.8	154	78.5	196	56	140	11.7	5.7	78.9	2	1	1	0	1	1	1	8.8	0.15	16.6
1	0	0	100	7.06	2	68	30.4	153.5	85	284	56	228	12.1	5.3	72.6	2	1	1	0	0	1	1	12.9	0.04	32.1
1	0	0	100	7.06	2	69	26	148.5	81	224	56	168	14	5.8	75.9	1	1	1	0	0	1	1	222.7	0.02	19.6
1	0	0	100	4.58	2	70	23.9	100.5	61	205	56	149	13.3	5.6	75.3	2	1	1	1	1	1	1	64.5	0.02	0.82
1	0	0	100	4.58	2	66	25.5	110.5	61	185	56	129	12.5	5.3	77.5	1	1	1	0	0	1	1	11.2	0.08	7.82
1	0	0	100	6.86	2	73	20	109.5	59	212	56	156	11.3	5.4	78.3	2	1	1	1	1	1	1	29.6	0.04	16.8
1	0	0	100	5.15	2	75	32	119.5	61	170	56	114	11.5	5.7	69.1	2	1	1	0	1	1	1	63.8	0.05	36.4
1	0	0	100	5.16	2	67	29.1	127.5	70	237	56	181	13.6	6	73.1	2	1	1	1	0	1	1	13.9	0.04	35.5
1	0	0	100	5.16	2	66	28.4	122	68.5	185	56	129	11.6	5.7	73.6	2	1	1	1	1	1	1	86.5	0.04	29
1	0	0	100	5.16	2	73	22.7	95.5	62	227	56	171	13.5	5.6	59.7	2	1	1	0	0	1	1	3.6	0.03	31.2
1	0	0	100	6.84	2	69	25.1	152.5	88	238	56	182	14.9	5.3	75.9	2	1	1	0	0	1	1	9.4	0.08	14.3
1	0	0	100	6.84	2	76	28.1	121	85	170	56	114	14.2	5.8	44.6	1	1	1	1	1	1	1	109.7	0.08	12.2
1	0	0	100	6.85	2	70	23.2	131.5	73.5	219	56	163	12.1	5.6	52.8	2	1	1	1	1	1	1	29.2	0.02	13.2
1	0	0	100	6.85	2	77	23	171	94.5	209	56	153	12.5	5	71.7	2	1	1	0	1	1	1	142.3	0.03	53.4
1	0	0	100	5.07	2	67	24	118	65.5	225	56	169	14.5	5.5	73.1	2	1	1	0	0	1	1	7.1	0.05	43.4
1	0	0	100	6.99	2	73	20.3	95	50	207	56	151	12	5.7	73.8	2	1	1	1	1	1	1	9.9	0.06	33.7
1	0	0	100	4.64	2	65	25.1	136.5	72	184	56	128	13.8	5.5	74.2	2	1	1	0	1	1	1	4.6	0.08	90.9
1	0	0	100	6.87	2	70	23.9	140.5	80.5	193	56	137	13.6	5	80.0	2	1	0	0	1	1	1	34.2	0.06	2.47
1	0	0	100	6.24	2	75	24.9	149	74.5	206	56	150	11.8	5.7	69.1	1	1	1	0	1	1	1	84.9	0.04	71.1
1	0	0	100	6.98	2	65	30.5	143	75.5	201	56	145	14.4	5.9	89.1	1	1	0	0	1	0	1	33.4	0.02	6.53
1	0	0	100	6.98	2	74	27.2	140.5	75.5	211	56	155	12.5	5	69.6	1	1	1	0	1	1	1	35.2	0.03	23.4
1	0	0	100	6.79	2	74	19.6	112	67	237	56	181	12.8	5.2	73.2	1	1	1	1	0	1	1	25.6	0.05	16.8
1	0	0	100	4.59	2	70	31.3	135.5	77.5	203	56	147	13.8	5.4	60.9	2	1	1	1	1	1	1	18.2	0.04	68.7
1	0	0	100	4.62	2	76	26.4	140	78.5	194	56	138	14.5	6.1	72.2	2	1	1	0	1	1	1	35.9	0.03	44.5

1	0	0	100	5.05	2	74	21.5	150	80	164	56	108	12.9	5.4	73.2	2	1	1	0	1	1	1	14.1	0.01	178
1	0	0	100	5.06	2	73	21.4	143	83	213	56	157	13	5.4	73.8	2	1	1	0	1	1	1	11.4	0.04	59.3
1	0	0	100	6.87	2	72	25	135	66	194	56	138	12.8	5.4	70.6	1	1	1	1	1	1	1	18.4	0.05	39.1
1	0	0	100	5.04	2	75	28.5	137	81.5	195	56	139	12.3	5	72.7	1	1	1	0	1	1	1	21	0.06	2.26
1	1	0	100	7.07	2	70	30.6	121	83	225	56	169	15	5.1	71.6	2	1	1	0	0	1	1	16	0.12	4.08
1	1	0	100	6.84	2	72	23.1	107.5	59	231	56	175	12.3	5.7	74.3	2	1	1	0	0	1	1	12.5	0.11	16.5
1	1	0	100	6.86	2	79	25.1	132.5	68.5	207	56	151	13.9	5.1	75.1	1	1	1	1	0	1	1	22.4	1.17	7.12
1	1	0	100	5.4	2	77	22.4	157	88.5	201	56	145	11.2	5.4	71.7	1	1	1	0	1	1	1	25.3	0.09	27
1	1	0	100	7	2	70	22.3	97.5	64	211	56	155	12.8	6.3	75.3	1	1	1	1	1	1	1	16.5	0.05	14.4
1	1	0	100	6.8	2	65	23.4	96	58.5	197	56	141	11.8	4.8	54.7	2	1	1	1	1	1	1	5.7	0.04	32.1
1	1	0	100	4.59	1	80	21.5	152	78	179	56	123	12.8	5.4	46.2	1	1	1	0	1	1	1	18.5	0.15	5.74
1	0	0	100	6.97	2	85	19	143	66	226	56	170	10.6	5.6	67.8	1	1	1	0	0	1	1	20.4	0.02	34.9
1	0	1	4.4	4.4	1	74	18.4	114	61.5	189	56	133	14.9	5.2	68.2	1	1	1	1	1	1	1	7.6	0.01	126
1	1	1	4.8	4.8	1	73	25	146.5	88	197	56	141	14.6	5.3	53.9	2	1	0	0	1	1	1	6.4	0.03	9.7
1	1	1	5.7	5.7	1	75	20.7	180	86.5	208	56	152	13.3	6.1	71.0	1	0	1	0	1	1	1	401	0.09	327
1	0	1	4.6	4.6	2	68	20.9	129	71.5	201	56	145	13.1	6.1	76.4	2	1	1	0	1	1	1	45.1	0.06	36.2
1	1	1	1.4	1.4	2	72	26.5	130.5	101	229	56	173	14.4	4.8	52.1	2	1	1	0	0	1	0	22	0.07	139
1	1	1	0.7	0.7	2	67	22.4	92	55	205	56	149	13	5.4	87.9	2	1	1	1	0	1	1	15	0.01	24.8
1	0	1	2.1	2.1	1	69	21.6	134.5	83	222	56	166	14.7	5.9	78.3	2	0	1	1	0	1	1	20.1	0.09	59.2
1	0	1	6.8	6.8	2	66	24.8	135.5	70.5	197	56	141	11.7	5	77.5	2	1	1	1	1	1	1	19.1	0.01	25.6
1	0	0	100	6.87	1	67	23.7	130.5	76.5	143	57	86	12.9	5.3	63.0	2	1	0	1	1	1	1	11.7	0.1	28.4
1	0	0	100	7.04	1	70	19.6	111	77.5	179	57	122	14.3	5	73.6	2	0	1	1	1	1	1	6.3	0.1	6.71
1	0	0	100	6.09	1	76	24	161	88.5	215	57	158	16.3	6.1	74.5	2	1	1	0	1	1	1	24.6	0.1	5.44
1	0	0	100	6.97	1	69	23.9	105.5	62	222	57	165	11.2	5.2	78.3	1	1	1	1	0	1	1	5.4	0.16	0.05
1	0	0	100	5.37	1	66	29.5	130.5	74.5	199	57	142	16	5.7	72.1	2	1	0	1	1	1	1	13.7	0.11	0.53
1	0	0	100	7	1	76	26.4	140	79	164	57	107	14.8	5.4	59.2	2	1	1	0	1	1	1	55.1	0.06	23.8
1	0	0	100	7.07	1	74	27.2	131.5	71.5	183	57	126	14	7.3	60.0	2	1	0	0	0	0	1	4.9	0.07	10.2
1	0	0	100	4.58	1	71	26	133	86	235	57	178	16.9	6.9	73.1	2	1	0	0	0	0	1	23.8	0.04	3.41
1	0	0	100	4.59	1	66	21.9	126.5	86.5	249	57	192	14.9	5.3	56.6	2	1	1	1	0	1	1	7.3	0.04	6.2
1	0	0	100	5.83	1	66	21	148.5	90	180	57	123	16.2	5.6	75.7	2	1	1	0	0	1	1	7.3	0.04	6.64
1	0	0	100	6.87	1	70	19.6	130.5	65	181	57	124	13.7	6.2	77.7	2	1	1	1	1	1	1	17	0.01	29.3
1	0	0	100	6.87	1	77	25.1	188.5	96	199	57	142	14.2	5.6	70.1	2	1	0	0	1	1	1	8.7	0.03	49.1
1	0	0	100	6.88	1	68	24.4	114	71.5	170	57	113	13.5	5.3	74.6	2	1	0	0	1	1	1	162.7	0.07	32.2
1	0	0	100	5.15	1	65	20.1	131.5	87.5	196	57	139	14.5	5.8	76.2	2	0	0	1	1	1	1	7	0.08	35.1
1	0	0	100	6.84	1	72	22.2	155	87	225	57	168	15.2	5.6	69.1	2	0	0	0	0	1	1	13.1	0.04	27.8
1	0	0	100	6.85	1	76	28.2	141	75	192	57	135	13.6	6	70.5	2	1	1	0	1	1	1	41.4	0.04	14.6
1	0	0	100	6.85	1	65	22.5	95.5	57	173	57	116	13.6	5.9	72.6	2	0	0	1	1	0	1	7.4	0.03	17.3
1	0	0	100	5.05	1	66	24.8	121.5	79.5	188	57	131	14.9	5.2	75.7	2	1	0	0	1	1	1	3.9	0.01	39.9
1	0	0	100	5.07	1	73	24.5	128.5	84.5	197	57	140	13.5	5.4	72.0	2	1	0	1	1	1	1	64.7	0.03	11
1	0	0	100	6.98	1	65	27.2	123	84.5	165	57	108	14.7	5.4	57.0	2	1	1	1	1	1	1	4.2	0.09	12.4
1	0	0	100	6.98	1	69	24	105	66	176	57	119	13.1	5.5	74.1	2	1	1	1	1	1	1	12.6	0.01	12.8
1	0	0	100	6.9	1	76	20.1	120.5	59	164	57	107	12.6	4.9	74.5	1	1	1	1	1	1	1	8	0.07	52.9
1	0	0	100	7	1	70	20.3	118.5	71.5	158	57	101	12.4	5.4	77.7	2	0	1	1	1	1	1	13.1	0.01	22.5
1	0	0	100	4.63	1	76	26.8	137	73.5	184	57	127	14.9	5	70.5	2	1	0	0	1	1	1	17.2	0.01	44.9
1	0	0	100	4.63	1	77	24.9	124.5	66	160	57	103	16.1	5.5	70.1	2	1	0	0	1	1	1	11.8	0.01	48.9
1	0	0	100	4.64	1	70	22.5	116	70.5	229	57	172	15.6	5.5	77.7	2	1	0	1	0	1	1	5.6	0.04	43
1	0	0	100	6.83	1	67	27.2	133.5	89	209	57	152	16	5.2	71.6	2	1	0	1	1	1	1	5.1	0.01	2.06
1	0	0	100	6.84	1	74	22.9	130.5	71	166	57	109	13.7	5	53.5	2	1	0	1	1	1	1	215.9	0.08	19.3
1	0	0	100	4.62	1	74	21.2	123	74.5	193	57	136	15.1	5.5	71.5	2	1	0	0	1	1	1	13.5	0.04	21.2
1	0	0	100	4.68	1	71	21.6	94.5	52.5	161	57	104	13.2	5.4	73.1	1	1	0	1	1	1	1	28.8	0.07	27
1	0	0	100	5.06	1	75	24.7	100	65.5	248	57	191	13.6	5.6	71.0	2	1	1	0	0	1	1	11.5	0.07	39.1

1	0	0	100	6.86	1	75	20.5	167	85	180	57	123	15.6	5.4	80.0	2	0	0	0	1	1	1	20.2	0.04	5.03
1	0	0	100	6.86	1	68	24.2	113	75	210	57	153	15.2	5.4	71.1	2	1	1	1	0	1	1	33.6	0.05	10.7
1	0	0	100	6.87	1	65	18.7	125	67.5	174	57	117	13	5.4	72.6	2	0	1	1	1	1	1	7.9	0.06	21.9
1	0	0	100	1.57	1	70	23.2	120	71	262	57	205	14.4	5.3	73.6	2	1	0	1	0	1	1	14	0.05	1.56
1	0	0	100	6.06	1	75	19.1	102.5	67.5	207	57	150	12	4.4	67.7	2	1	0	1	1	1	1	10.6	0.09	25.4
1	1	0	100	4.6	1	73	25.8	128	69.5	254	57	197	14.8	5.3	72.0	2	1	0	1	0	1	1	10.3	0.03	10.2
1	0	0	100	7	2	71	29.7	135.5	75	203	57	146	14.1	5.7	71.1	2	1	1	0	1	1	0	119.4	0.23	143
1	0	0	100	6.86	2	72	27.5	144	69	249	57	192	12.2	6.9	70.6	2	1	0	0	0	0	1	27.1	0.44	60.6
1	0	0	100	7.23	2	67	22.1	158.5	96.5	236	57	179	13.5	5.3	62.2	2	1	1	0	0	1	1	12.3	0.12	29.8
1	0	0	100	7.03	2	70	21.7	97.5	54.5	220	57	163	12.8	5.2	75.3	1	1	0	1	0	1	1	8	0.1	3.82
1	0	0	100	5.07	2	76	31	148.5	78.5	256	57	199	13.1	6.7	76.7	1	1	1	0	0	0	1	15.4	0.1	4.34
1	0	0	100	6.99	2	70	21	123.5	85	167	57	110	13	5.3	75.3	1	1	1	1	1	1	1	28.8	0.12	17.2
1	0	0	100	4.63	2	75	22.3	125	73.5	200	57	143	11.2	5.6	83.1	2	1	1	1	1	1	1	30.8	0.12	86.5
1	0	0	100	6.79	2	76	26.2	115	72	271	57	214	13.7	5.7	58.4	2	1	1	0	0	1	1	24	0.25	18.1
1	0	0	100	4.67	2	67	24.9	201.5	100	281	57	224	14.3	4.8	76.9	2	1	1	0	0	1	1	57.5	0.18	9.15
1	0	0	100	4.69	2	71	26.5	139	83.5	179	57	122	12.7	5.3	74.8	2	1	0	1	1	1	1	15.4	0.25	38.1
1	0	0	100	5.04	2	71	30.7	124.5	81.5	195	57	138	13.5	5.2	71.1	1	1	1	0	1	1	1	50.1	0.25	52
1	0	0	100	7	2	71	22.2	115.5	63	182	57	125	12.5	5.6	71.1	2	1	1	1	1	1	1	13	0.05	11.7
1	0	0	100	7.05	2	76	22.5	160.5	88.5	194	57	137	12.8	5.3	72.2	1	1	1	0	1	1	1	46.7	0.01	59.5
1	0	0	100	4.58	2	67	25.1	140.5	85	256	57	199	12.4	5.9	73.1	2	1	1	0	0	1	1	21.6	0.04	5.31
1	0	0	100	4.58	2	72	24.2	135.5	78.5	184	57	127	12.7	5.6	74.3	2	1	1	1	0	1	1	9.4	0.03	26.2
1	0	0	100	4.59	2	76	25.1	150.5	80	209	57	152	12.2	5.3	68.6	2	1	1	0	1	1	1	41.6	0.04	41.5
1	0	0	100	4.59	2	79	22.3	138	76	206	57	149	12.5	5.2	70.7	2	1	0	1	1	1	1	4.5	0.01	28.5
1	0	0	100	6.86	2	66	29.3	116.5	75.5	159	57	102	11.2	4.9	82.3	2	1	1	1	1	1	1	11.6	0.05	19.3
1	0	0	100	5.16	2	67	24.5	115.5	69	201	57	144	12.5	5.8	76.9	2	1	1	0	1	1	1	73.4	0.07	27.4
1	0	0	100	5.16	2	70	26.2	155.5	83.5	209	57	152	13.3	5.9	75.3	2	1	1	0	1	1	1	27.2	0.03	31.8
1	0	0	100	5.16	2	72	25.2	152.5	82	218	57	161	13.8	5.4	60.1	2	1	1	0	1	1	1	30.2	0.06	22.4
1	0	0	100	6.84	2	69	29.2	136	76	234	57	177	14.1	5.3	61.4	2	1	1	0	0	1	1	7	0.04	22.6
1	0	0	100	6.85	2	67	24.3	102.5	66	248	57	191	14.7	5.3	76.9	2	1	0	1	0	1	1	10.3	0.09	30.9
1	0	0	100	6.85	2	78	30.4	166.5	88	270	57	213	15.5	5.4	67.7	2	1	1	0	0	1	1	6.8	0.03	11.1
1	0	0	100	6.85	2	68	21.9	112.5	67	244	57	187	13.3	5.6	72.6	2	1	1	1	0	1	1	10.6	0.02	11.9
1	0	0	100	7.01	2	69	24.8	128	67	257	57	200	13.3	6	75.9	1	1	1	1	0	1	1	12.4	0.04	6.77
1	0	0	100	5.08	2	68	19.7	134	70.5	220	57	163	11.8	5.1	72.6	2	1	1	1	0	1	1	16.2	0.04	56.3
1	0	0	100	7	2	66	22.1	112.5	68	215	57	158	12.3	5.5	77.5	2	1	1	1	1	1	1	12.9	0.05	9.27
1	0	0	100	4.62	2	67	21.1	114	75.5	230	57	173	14	5	73.1	2	1	1	1	0	1	1	26.5	0.02	16.5
1	0	0	100	6.97	2	67	19	131	78	236	57	179	13	4.9	73.1	2	1	1	1	0	1	1	14.2	0.01	30.9
1	0	0	100	6.97	2	70	21.5	131.5	75	220	57	163	13.4	5.5	80.0	1	1	1	1	0	1	1	13.6	0.02	5.84
1	0	0	100	6.98	2	68	25.1	148.5	96	192	57	135	13.4	4.7	76.4	2	1	1	0	1	1	1	14.6	0.05	4.5
1	0	0	100	6.8	2	72	21.7	136.5	75	198	57	141	13.6	5.2	70.6	2	1	1	1	1	1	1	4.7	0.01	23.7
1	0	0	100	6.8	2	67	19.6	150	90.5	159	57	102	12.7	5	73.1	2	1	0	0	1	1	1	9.9	0.03	53.8
1	0	0	100	6.8	2	66	23.1	113	69	219	57	162	13.3	5.5	77.5	2	1	0	1	1	1	1	7.4	0.01	10.7
1	0	0	100	6.83	2	70	29.2	129.5	83.5	220	57	163	15.1	6.8	75.3	1	1	1	0	0	0	1	161.6	0.09	22.9
1	0	0	100	6.84	2	70	24.5	129	67.5	177	57	120	12.4	5.8	71.6	2	1	1	1	1	1	1	9.6	0.02	32
1	0	0	100	4.59	2	65	23.8	134	75	232	57	175	13.7	5.6	74.2	2	1	1	1	0	1	1	5.2	0.02	17.9
1	0	0	100	4.6	2	68	22.3	119.5	63	216	57	159	12.9	5.2	76.4	2	1	1	0	1	1	1	28.4	0.02	27
1	0	0	100	4.62	2	79	22.9	158	76	209	57	152	14.7	5	67.2	1	1	1	0	1	1	1	18.9	0.05	44.3
1	0	0	100	6.44	2	68	27.3	131.5	80.5	224	57	167	14.8	5.8	72.6	2	1	1	1	0	1	1	46.8	0.06	8.31
1	0	0	100	6.96	2	76	19.1	160.5	81.5	215	57	158	12.2	5.4	72.2	1	1	1	0	1	1	1	15.1	0.03	0.05
1	0	0	100	6.86	2	66	25.4	141.5	82.5	195	57	138	13.3	5.4	77.5	2	1	1	0	1	1	1	11.4	0.09	34.6
1	0	0	100	6.87	2	69	20.1	117.5	70	213	57	156	13.7	6	80.5	1	1	1	1	1	1	1	29.4	0.04	8.54
1	0	0	100	6.87	2	68	22.8	107	64.5	222	57	165	11.9	5.5	61.8	2	1	1	1	0	1	1	12.3	0.08	16.4

1	0	0	100	6.87	2	74	22	137.5	76.5	178	57	121	13.1	6.8	69.6	1	1	1	0	1	0	1	12.4	0.05	32.6
1	0	0	100	6.87	2	69	22.9	97	60.5	192	57	135	13.8	5.3	80.5	2	1	1	0	1	1	1	14.6	0.02	4.27
1	0	0	100	5.04	2	67	25.7	123	73	213	57	156	12.1	6.2	81.7	2	1	1	1	1	1	1	34.8	0.03	1.74
1	1	0	100	5.08	2	74	33.9	132	87.5	212	57	155	13.5	5.5	73.2	2	1	1	1	1	1	1	11	0.22	10.4
1	1	0	100	6.87	2	73	20.1	150	67	222	57	165	13.2	5.3	73.8	2	1	1	0	0	1	1	8.5	0.02	42.1
1	1	0	100	7.03	2	73	22.6	100	56	153	57	96	13.7	5.2	59.7	1	1	1	1	1	1	1	6.9	0.03	19.5
1	1	0	100	6.96	2	69	23.1	108	67.5	222	57	165	12	5	80.5	1	1	1	1	0	1	1	17.2	0.01	9.67
1	1	0	100	6.98	2	70	25.8	159.5	96.5	221	57	164	13.1	5.1	75.3	1	1	0	0	0	1	1	24.4	0.02	51.2
1	0	0	100	7.07	1	80	20.8	117	69.5	173	57	116	14.3	5.1	57.5	2	1	1	1	1	1	1	8	0.03	54.7
1	1	0	100	5.63	1	88	19.6	156.5	75	195	57	138	9.3	5.2	17.1	1	1	0	0	1	0	1	3004.3	0.08	256
1	0	0	100	6.79	2	80	28.6	127	77.5	187	57	130	14	5.3	70.2	1	1	1	0	1	1	1	10.3	0.21	8.37
1	0	0	100	6.86	2	83	19.3	99	60	192	57	135	11.9	5.6	65.3	2	1	1	1	1	1	1	7.4	0.03	34.1
1	1	0	100	6.82	2	82	23	133	75	166	57	109	13.5	5.4	56.0	1	1	1	0	1	1	1	18.6	0.07	48.2
1	0	0	100	5.02	1	75	22.8	155.5	72	218	57	161	16.2	8.1	67.7	2	0	0	0	1	0	1	54.7	0.26	33.7
1	0	0	100	6.87	1	68	18.9	131.5	69.5	172	57	115	14.8	5.6	84.0	1	1	1	0	0	1	1	29.5	0.33	45.3
1	0	0	100	5.15	1	66	18.9	113.5	69	162	57	105	15.8	5.1	72.1	2	1	1	1	1	1	1	7.9	0.57	12.6
1	0	0	100	6.87	1	76	32.5	148	85.5	198	57	141	13.7	6.2	70.5	2	1	0	0	0	1	1	25	0.2	13.5
1	0	0	100	4.63	1	70	24.7	126	80.5	165	57	108	13.1	4.9	70.1	1	1	0	1	1	1	1	4	0.04	13
1	0	0	100	6.83	1	67	24.1	128	76	175	57	118	14.5	5.7	79.4	2	1	0	1	1	1	1	9.9	0.04	9.9
1	0	0	100	4.59	1	66	22.6	120	69	197	57	140	16	6	75.7	2	0	0	1	1	1	1	9.2	0.09	16.1
1	0	0	100	5.37	1	72	21.5	143	82.5	139	57	82	13.3	5.3	76.7	1	0	0	0	1	1	1	10	0.03	19.5
1	0	0	100	5.04	1	74	24.5	131.5	76	137	57	80	15.5	5.3	71.5	2	1	0	1	1	1	1	5.7	0.05	19.2
1	0	0	100	7	2	66	24.4	143	75.5	275	57	218	12.8	5.2	73.6	2	1	1	0	0	1	1	15.4	0.17	27.6
1	0	0	100	7.05	2	68	30.2	152.5	82	197	57	140	13.1	5.2	72.6	1	1	1	0	1	1	1	34.7	0.12	25.3
1	0	0	100	4.59	2	69	27.1	110	76	268	57	211	10.9	5.6	20.3	2	1	1	0	0	1	1	2428	0.21	15.3
1	0	0	100	6.87	2	72	25.9	121	67.5	206	57	149	12.8	5.8	70.6	2	1	1	0	1	1	1	36.8	0.1	42.2
1	0	0	100	5.16	2	78	25.1	101	55.5	212	57	155	12.4	5.6	50.0	2	1	1	0	1	1	1	4.5	0.14	23.4
1	0	0	100	5.06	2	72	26.9	107	57	221	57	164	12.2	5.9	74.3	1	1	1	1	0	1	1	18.3	0.12	43.6
1	0	0	100	2.42	2	71	26.9	117	71.5	181	57	124	10.5	4.4	46.2	1	1	1	0	1	1	1	201.3	0.34	31.2
1	0	0	100	7	2	79	22.5	120	59	250	57	193	12.8	5.5	57.2	1	1	0	0	0	1	1	12.1	0.58	25
1	0	0	100	4.62	2	70	13.6	99.5	56	155	57	98	11.3	5.1	75.3	2	1	1	1	1	1	1	20.1	0.17	41.2
1	0	0	100	4.61	2	77	31.2	147	83.5	168	57	111	12.8	5.8	71.7	1	1	1	0	1	1	1	138.7	0.1	16.3
1	0	0	100	4.7	2	74	28.8	150.5	78	219	57	162	12.2	5.7	73.2	1	1	0	0	1	1	1	8.1	0.12	68.3
1	0	0	100	6.86	2	71	31.9	136.5	82	231	57	174	12.9	5	74.8	2	1	1	0	0	1	1	32.6	0.45	15.4
1	0	0	100	4.05	2	74	22.8	123.5	70	204	57	147	13.6	7.6	73.2	2	1	1	0	1	0	1	42.9	0.13	15.4
1	0	0	100	5.04	2	73	32.8	132.5	71	173	57	116	12.8	5.1	78.3	1	1	1	1	1	1	1	6.2	0.39	1.46
1	0	0	100	7.05	2	74	21.5	136	69	222	57	165	13	5.1	51.4	1	1	1	1	0	1	1	19.5	0.02	41.5
1	0	0	100	7.08	2	73	24.3	153.5	80.5	200	57	143	11.8	5.2	73.8	1	1	1	0	1	1	1	27.8	0.04	45.6
1	0	0	100	6.86	2	79	23.9	147	83	176	57	119	13.7	5.4	70.7	1	1	0	0	1	1	1	16	0.03	29.5
1	0	0	100	6.86	2	73	25	141	77	195	57	138	14.3	5.5	59.7	2	1	1	0	0	1	1	26.4	0.03	0.05
1	0	0	100	6.87	2	65	30.2	108.5	72	194	57	137	11.9	5.2	74.2	1	1	1	1	1	1	1	12.2	0.03	21.6
1	0	0	100	5.16	2	73	27.8	105.5	70	207	57	150	13.1	5.7	70.1	2	1	1	0	1	1	1	13.4	0.04	24.7
1	0	0	100	5.16	2	74	23.3	130.5	74	207	57	150	13.3	6.3	69.6	2	1	1	0	0	1	1	22	0.03	6.36
1	0	0	100	6.85	2	70	26.2	162.5	87	172	57	115	15.6	5.6	75.3	2	1	1	0	1	1	1	17.7	0.09	21.3
1	0	0	100	7.21	2	65	24.2	147	91.5	186	57	129	11.8	5.3	78.0	2	1	1	0	1	1	1	9.2	0.04	46.1
1	0	0	100	5.05	2	71	26.6	166	82	176	57	119	12.6	5.4	74.8	1	1	1	0	1	1	1	4.5	0.01	22.8
1	0	0	100	5.07	2	71	21	127.5	57.5	172	57	115	12.5	5.6	79.4	1	1	1	1	1	1	1	6.1	0.01	25.8
1	0	0	100	5.08	2	76	26	156	80	208	57	151	12.2	5.7	58.4	1	1	1	0	1	1	1	3.6	0.08	57.3
1	0	0	100	6.97	2	67	26.7	126	68.5	202	57	145	13.3	5.3	81.7	2	1	0	0	0	1	1	5.5	0.04	0.05
1	0	0	100	4.62	2	72	29.6	123.5	72	184	57	127	13.2	5.2	74.3	2	1	1	1	1	1	1	5.6	0.06	20.1
1	0	0	100	4.63	2	68	24.8	116.5	58.5	225	57	168	10.6	5.2	76.4	2	1	1	1	0	1	1	8	0.02	25.8

1	0	0	100	4.63	2	72	25.3	137.5	82.5	207	57	150	14.5	5.8	78.9	2	1	1	1	1	1	1	5.1	0.09	50.4
1	0	0	100	6.97	2	72	29.5	146.5	85.5	270	57	213	14.1	5.4	70.6	2	1	1	0	0	1	1	66.2	0.06	23
1	0	0	100	6.98	2	71	23.4	116	64	197	57	140	12.8	5.3	71.1	1	1	1	1	1	1	1	8.1	0.02	18.8
1	0	0	100	6.82	2	66	19.7	134	76.5	207	57	150	12.8	5.3	77.5	2	1	1	1	1	1	1	12	0.01	38.9
1	0	0	100	4.59	2	67	26.7	132.5	67	204	57	147	14	5.6	76.9	2	1	0	0	1	1	1	4.4	0.03	24.1
1	0	0	100	4.62	2	77	27.2	113.5	57.5	183	57	126	13.2	5.3	71.7	1	1	1	0	0	1	1	12.7	0.07	58.5
1	0	0	100	5.36	2	68	24.5	158.5	95	187	57	130	13.2	5.7	72.6	2	1	1	0	1	1	1	6.7	0.02	41.9
1	0	0	100	5.36	2	71	19	131.5	54.5	173	57	116	10.6	5.8	74.8	2	1	1	1	1	1	1	24.4	0.05	192
1	0	0	100	6.86	2	77	23.1	142	80	223	57	166	13	6	71.7	1	1	1	0	0	1	1	21	0.09	7.83
1	1	0	100	7.05	2	74	27.5	121	69.5	206	57	149	12.4	5.3	40.3	1	1	1	0	1	1	1	70.1	0.41	43.1
1	1	0	100	3.55	2	75	24.6	129	67	208	57	151	14.3	6.7	72.7	2	1	1	0	1	0	1	23.2	0.18	14.5
1	1	0	100	7.07	2	70	29.5	149.5	78.5	200	57	143	14.4	5	80.0	2	1	1	0	1	1	1	35.7	0.04	25.6
1	1	0	100	6.77	2	78	28.4	143	75.5	252	57	195	13	5.2	71.2	2	1	1	0	0	1	1	146.9	0.05	20.7
1	1	0	100	6.18	2	73	21	155.5	80.5	199	57	142	14.4	5.5	59.7	2	0	1	0	1	1	1	16.7	0.01	0.05
1	1	0	100	6.84	2	78	21.7	95	54	183	57	126	10	5.1	29.3	2	1	1	1	1	1	1	14.6	0.03	34.7
1	1	0	100	5.06	2	76	20	138.5	87.5	143	57	86	11.7	5.4	72.2	2	1	1	1	1	1	0	40.5	0.02	267
1	1	0	100	5.04	2	78	31.2	109.5	60	202	57	145	12.9	5.3	57.6	2	1	1	1	1	1	1	3	0.03	2.94
1	1	0	100	6.98	1	82	19.7	119.5	66.5	154	57	97	14.9	5.2	56.7	1	1	0	0	1	1	0	4.5	0.03	110
1	0	0	100	6.86	2	81	21.1	145	70	231	57	174	13.1	5	56.4	2	1	1	0	0	1	1	24.9	0.05	129
1	1	0	100	7.07	2	80	19.3	133.5	77	251	57	194	12.1	5.2	66.7	2	1	1	1	0	1	0	45.1	0.01	187
1	1	0	100	4.64	2	82	21.7	83.5	47.5	202	57	145	11.8	4.9	65.8	1	1	1	1	1	1	1	14.2	0.01	31
1	0	1	2.7	2.7	1	66	28	122	76.5	165	57	108	13.3	5.5	79.9	2	1	1	1	1	1	1	2.7	0.02	20
1	0	1	2.9	2.9	2	67	25.8	122	69.5	186	57	129	14.8	6.1	54.0	1	1	1	1	0	1	1	17.5	0.07	10.9
1	1	1	3.5	3.5	2	78	22.3	109.5	54.5	202	57	145	13.3	5	67.7	2	1	1	0	1	1	1	25.6	0.04	92.9
1	1	1	2.3	2.3	2	66	24.5	102	55.5	151	57	94	13.6	4.7	73.6	2	1	1	1	1	1	1	11.1	0.03	5.84
1	0	1	5.8	5.8	1	73	24.1	128	73	246	57	189	13.3	6.5	76.1	2	1	1	1	0	0	1	16.3	1.83	17.7
1	1	1	1	1	1	74	19.7	129.5	69	200	57	143	13	5.6	71.5	1	0	1	1	1	1	1	29.4	0.03	11.8
1	1	1	2.1	2.1	1	72	28.2	149.5	85	196	57	139	15.7	5.1	76.7	2	1	1	0	1	1	1	110.6	0.01	12
1	1	1	3.9	3.9	2	70	28.9	116.5	68	255	57	198	12.9	5.3	80.0	2	1	1	1	0	1	0	17.7	0.14	59.5
1	1	1	3.1	3.1	2	69	25.3	120	71.5	218	57	161	13.3	5.5	80.5	2	1	1	0	0	1	1	18.6	0.02	1.97
1	1	1	5.8	5.8	2	71	20	173	87	190	57	133	11.9	5.3	79.4	2	1	1	0	1	1	1	26.9	0.01	7.41
1	1	1	0.6	0.6	2	75	27.8	167	91.5	176	57	119	13.6	5.9	69.1	1	1	1	0	1	1	1	17.2	0.05	37.7
1	0	0	100	6.88	1	78	21	148.5	82.5	200	58	142	11.7	5.3	69.6	2	1	1	0	1	1	1	6.7	0.14	51.5
1	0	0	100	7.04	1	68	23.7	154.5	86.5	135	58	77	15.3	4.8	84.0	1	1	0	0	1	1	1	7.8	0.1	0.05
1	0	0	100	4.63	1	73	22.1	151.5	64.5	179	58	121	12.8	5.5	72.0	2	1	0	0	1	1	1	16.8	0.13	30.7
1	0	0	100	4.64	1	73	26.1	134	78.5	249	58	191	15.8	5.7	60.4	2	1	1	1	0	1	1	6.8	0.1	12.3
1	0	0	100	6.87	1	67	30.9	133.5	74.5	202	58	144	12.6	6.3	71.6	2	1	0	0	1	1	1	292.5	0.23	6.03
1	0	0	100	6.95	1	73	25.4	159	86	192	58	134	16.3	5.1	68.6	2	1	1	0	1	1	1	48.4	0.12	15
1	0	0	100	4.62	1	67	27.4	152.5	97.5	244	58	186	17.6	8.7	63.0	2	1	0	0	0	0	1	714.3	0.11	14.9
1	0	0	100	6.86	1	71	22.2	149.5	83	182	58	124	13.8	5	77.2	2	0	0	0	1	1	1	9.4	0.17	25.9
1	0	0	100	1.22	1	68	20.5	128.5	70.5	208	58	150	14.4	4.8	84.0	1	0	0	1	1	1	1	386.7	0.12	50.8
1	0	0	100	6.87	1	66	24.6	122	65.5	185	58	127	15.3	5.6	79.9	2	1	1	1	1	1	1	34.6	0.11	1.78
1	0	0	100	7	1	66	27.2	139	75	180	58	122	15.1	5.3	79.9	2	1	0	0	1	1	1	29.4	0.01	27.6
1	0	0	100	7.06	1	70	27.3	135.5	75	175	58	117	15.3	5.7	73.6	2	1	1	1	1	1	1	7.9	0.01	5.85
1	0	0	100	4.59	1	72	18.2	129	68	244	58	186	13	5.3	72.6	2	1	0	1	0	1	1	6.1	0.06	28.5
1	0	0	100	6.86	1	69	23.7	111	66	188	58	130	15.4	4.7	74.1	2	0	0	1	1	1	1	5.9	0.01	8.93
1	0	0	100	6.87	1	71	24.8	125.5	74	161	58	103	13.8	5.7	73.1	2	1	0	1	1	1	1	2.7	0.03	6.42
1	0	0	100	6.87	1	66	22.3	105.5	60.5	169	58	111	14.7	5.5	72.1	2	1	0	1	1	1	1	3.1	0.01	6.78
1	0	0	100	5.16	1	66	26.9	130.5	75	197	58	139	16.4	5.9	75.7	2	1	0	0	1	1	1	21.5	0.08	81.8
1	0	0	100	6.85	1	66	21.1	112	63.5	155	58	97	13.1	5.2	63.5	2	1	0	1	1	1	1	7.6	0.02	5.06
1	0	0	100	6.85	1	71	22.4	129	77.5	199	58	141	13.6	5.4	77.2	2	1	0	0	1	1	1	22.9	0.03	36.2

1	0	0	100	6.85	1	71	20.7	139	86.5	232	58	174	14.4	5.2	73.1	2	1	1	1	0	1	1	11.7	0.03	9.51
1	0	0	100	7.01	1	74	24.2	115.5	62.5	169	58	111	16.5	5.7	71.5	1	1	1	1	1	1	1	16.8	0.03	3.76
1	0	0	100	5.05	1	71	24.3	96	66.5	178	58	120	13.7	5.2	69.6	1	1	1	1	1	1	1	4.1	0.06	24.2
1	0	0	100	5.08	1	71	20.6	150.5	93.5	177	58	119	14.2	5.2	73.1	2	1	0	0	1	1	1	10.9	0.02	6.35
1	0	0	100	5.08	1	65	24.1	107	73	203	58	145	14	5.4	76.2	2	1	0	1	1	1	1	4.6	0.05	20.8
1	0	0	100	7	1	67	24.4	112.5	71	224	58	166	14.7	5.4	79.4	2	1	0	1	0	1	1	4.7	0.04	6.24
1	0	0	100	7	1	70	26.3	123	81.5	195	58	137	12.8	5.5	70.1	2	1	0	0	1	1	1	13.4	0.03	16.4
1	0	0	100	4.62	1	67	22.8	125.5	76.5	204	58	146	15.7	7.5	75.2	2	1	0	0	1	0	1	11.4	0.02	6.13
1	0	0	100	4.63	1	68	20.2	114	65.5	176	58	118	14.9	5.5	78.8	2	0	1	1	1	1	1	4.2	0.01	39.6
1	0	0	100	4.63	1	69	24.3	118	77	182	58	124	13.7	6.6	83.4	2	1	1	1	1	0	1	36.4	0.09	11.5
1	0	0	100	4.64	1	72	26.1	148	82.5	153	58	95	14.5	5.4	54.2	2	1	0	0	1	1	1	8.9	0.05	10.2
1	0	0	100	6.97	1	72	20.7	106.5	67.5	167	58	109	12.2	5.2	76.7	1	0	0	1	1	1	1	13.4	0.04	26.1
1	0	0	100	6.8	1	66	20.2	112	77	205	58	147	15.4	4.7	79.9	1	1	0	1	1	1	1	21.4	0.02	6.56
1	0	0	100	4.59	1	69	25.2	141.5	86	173	58	115	15.8	6	70.6	2	1	0	0	1	1	1	53.2	0.03	45.2
1	0	0	100	6.45	1	69	20.8	159	82.5	212	58	154	15.6	6.5	78.3	2	1	1	0	1	0	1	137.2	0.02	16.3
1	0	0	100	6.45	1	73	21.1	117	80	205	58	147	13.4	5.9	81.1	1	1	1	1	1	1	1	69.6	0.05	29.1
1	0	0	100	6.45	1	73	21.3	106	66.5	193	58	135	16	5.7	72.0	2	1	0	1	1	1	1	15.1	0.04	21.3
1	0	0	100	6.86	1	65	21.5	128.5	71	179	58	121	14	7.1	76.2	2	0	0	1	1	0	1	27.1	0.07	11.1
1	0	0	100	6.86	1	69	26	163	91	181	58	123	13.6	5.7	74.1	2	1	0	0	1	1	1	13.2	0.08	6.95
1	0	0	100	6.87	1	69	26.2	112.5	60	182	58	124	15	4.9	78.3	2	0	1	1	1	1	1	45.1	0.01	61.7
1	0	0	100	7.06	2	76	28.9	172.5	84	227	58	169	15.4	5.3	76.7	1	1	0	0	0	1	1	35.1	0.12	45.1
1	0	0	100	7.07	2	71	24.3	143	88.5	177	58	119	12.8	5.1	46.2	1	1	1	0	1	1	1	473.5	0.38	29.7
1	0	0	100	4.59	2	67	31.8	131.5	83.5	259	58	201	14	5.6	73.1	2	1	0	1	0	1	1	9.3	0.18	9.23
1	0	0	100	7.21	2	69	21	124.5	69.5	183	58	125	11.9	5.2	53.2	2	1	1	0	1	1	1	4.4	0.34	14.9
1	0	0	100	7.23	2	72	24.6	156	84	258	58	200	14.8	5.6	70.6	2	1	1	0	0	1	1	10.1	0.24	4.64
1	0	0	100	6.84	2	76	21.8	135	66.5	213	58	155	12	5.8	58.4	1	1	1	1	1	1	1	24.6	0.12	13.1
1	0	0	100	6.85	2	67	26.3	123	73.5	197	58	139	12.1	5.5	76.9	2	1	0	1	1	1	1	5.2	0.1	35
1	0	0	100	6.86	2	76	24.5	136	64	221	58	163	12.9	5.4	72.2	2	1	1	0	0	1	1	22.6	0.16	30.3
1	0	0	100	6.86	2	65	26.3	125.5	75.5	238	58	180	12.1	5.5	78.0	2	1	0	1	0	1	1	14.5	0.1	7.4
1	0	0	100	5.06	2	66	29.5	109.5	55	186	58	128	12.8	5.3	82.3	2	1	1	1	1	1	1	34.2	0.2	17.4
1	0	0	100	6.97	2	65	20	134	71.5	217	58	159	11.9	5.6	82.8	2	1	1	0	1	1	1	8.1	0.23	31.6
1	0	0	100	4.64	2	69	23.5	127	77.5	183	58	125	13.9	5.4	75.9	2	1	1	1	1	1	1	17.5	0.16	12.2
1	0	0	100	6.84	2	73	24.4	144	87	219	58	161	13.1	5.8	51.7	2	1	1	0	1	1	1	22.2	0.12	15.9
1	0	0	100	4.61	2	73	20.4	111	55.5	192	58	134	11.6	5.2	59.7	2	1	1	1	1	1	1	53.7	0.16	31.6
1	0	0	100	4.62	2	73	21.2	111.5	70.5	244	58	186	14.3	4.9	73.8	2	1	0	1	0	1	1	17.9	0.1	18.3
1	0	0	100	4.69	2	66	22.8	115.5	69	202	58	144	14.1	4.4	77.5	2	1	1	1	1	1	1	11.6	0.1	27.2
1	0	0	100	7.06	2	69	23.9	125	77	217	58	159	14	5.5	75.9	2	1	1	1	1	1	1	10.3	0.09	51.6
1	0	0	100	6.86	2	77	26.7	114.5	73.5	218	58	160	10.9	5.4	68.2	2	1	1	0	1	1	1	16.6	0.01	32.9
1	0	0	100	6.88	2	67	18.8	124.5	86	205	58	147	12.2	5.2	76.9	2	1	1	0	1	1	1	14.4	0.03	70.5
1	0	0	100	5.16	2	69	22.8	133.5	82	176	58	118	12.5	5.8	75.9	2	1	1	1	1	1	1	46.6	0.04	13.2
1	0	0	100	5.16	2	73	25.2	125	79	216	58	158	13	4.2	73.8	1	1	1	1	1	1	1	11.4	0.03	61.3
1	0	0	100	6.85	2	67	21.8	105.5	57	225	58	167	12.7	5	76.9	2	1	1	1	0	1	1	4.5	0.03	27.9
1	0	0	100	7.01	2	66	21.1	141.5	72	195	58	137	12.6	5.1	77.5	2	1	1	0	0	1	1	16.8	0.03	52.8
1	0	0	100	7.02	2	68	20.5	111.5	69.5	225	58	167	15.8	5.7	81.1	1	1	0	1	0	1	1	30.1	0.03	0.05
1	0	0	100	7.04	2	67	27.1	118.5	73	227	58	169	11.7	5.7	81.7	2	1	1	1	0	1	1	10	0.06	0.05
1	0	0	100	5.07	2	68	22.7	119	66.5	165	58	107	11.7	5.2	76.4	2	1	1	1	1	1	1	14.1	0.09	23
1	0	0	100	5.08	2	65	18.8	99	64.5	203	58	145	11.4	5.6	78.0	2	1	1	1	1	1	1	13.4	0.01	58.7
1	0	0	100	7	2	67	19.9	102.5	56.5	173	58	115	11.8	5.4	76.9	2	1	1	1	1	1	1	3.6	0.06	4.93
1	0	0	100	4.63	2	71	25.3	123.5	73	216	58	158	13.6	6.1	71.1	2	1	1	1	1	1	1	16.3	0.02	18.2
1	0	0	100	4.63	2	69	22.7	120.5	72	183	58	125	13.1	5.2	72.1	2	1	0	1	0	1	1	11.9	0.03	43.7
1	0	0	100	4.64	2	71	24.7	124.5	73.5	209	58	151	12.6	7.2	74.8	2	1	0	1	1	0	1	9.3	0.03	31

1	0	0	100	6.95	2	65	24.6	128.5	76.5	171	58	113	12.3	5.3	74.2	2	1	1	0	1	1	1	61.9	0.09	19.8
1	0	0	100	6.96	2	69	21.8	126.5	77	276	58	218	13.8	6	75.9	1	1	1	1	0	1	1	22	0.01	3.51
1	0	0	100	6.79	2	71	27.8	142	76.5	197	58	139	14.4	5.5	74.8	2	1	1	0	1	1	1	5.3	0.02	19.5
1	0	0	100	6.8	2	72	24.1	114	66	239	58	181	12.8	5.5	74.3	2	1	1	1	0	1	1	60.2	0.07	46.7
1	0	0	100	6.8	2	74	23.6	140	78.5	181	58	123	13.8	5.5	77.8	1	1	1	0	1	1	1	20.1	0.03	9.95
1	0	0	100	6.82	2	67	26.4	129.5	85.5	222	58	164	15.5	5.4	81.7	2	1	1	0	0	1	1	10.3	0.05	5.9
1	0	0	100	4.62	2	70	26	121	79	284	58	226	13.6	6.2	60.9	2	1	1	1	0	1	1	10.7	0.02	9.76
1	0	0	100	6.44	2	67	22.3	124	71.5	272	58	214	14	5.2	81.7	2	1	0	1	0	1	1	19	0.03	25
1	0	0	100	5.37	2	66	29.9	129.5	76.5	163	58	105	13.6	6.9	82.3	2	1	0	0	1	0	1	29	0.06	3.95
1	0	0	100	6.87	2	67	22.7	125.5	66.5	241	58	183	13.7	6.6	76.9	2	1	1	1	0	0	1	4.9	0.06	13.3
1	0	0	100	5.04	2	67	25.2	127.5	80	189	58	131	13.4	5.3	73.1	2	1	1	1	1	1	1	9.2	0.02	24
1	1	0	100	4.59	2	73	19.2	113.5	64	193	58	135	12.3	6.1	51.7	2	1	0	0	1	0	0	15	0.23	91.7
1	1	0	100	6.86	2	68	30.6	184.5	91.5	292	58	234	15.6	11.5	76.4	2	1	1	0	0	0	1	550.7	0.31	11.1
1	1	0	100	7.01	2	78	24.4	136.5	65.5	260	58	202	12	6.2	75.6	1	1	1	0	0	1	1	77.8	0.06	71.9
1	1	0	100	6.87	2	72	20.2	146	76.5	221	58	163	12.8	5.6	74.3	2	1	1	0	0	1	1	19.2	0.06	35.2
1	1	0	100	4.6	2	71	26.9	131	66.5	185	58	127	13.6	6.9	74.8	2	1	1	0	1	0	0	20.8	0.08	177
1	1	0	100	6.71	2	74	27.2	128	84	250	58	192	13.8	5.5	59.2	2	1	1	1	0	1	1	151.9	0.08	22.1
1	0	0	100	4.59	1	80	23.9	116	62.5	202	58	144	13.3	5.7	65.4	2	1	1	1	1	1	1	10	0.19	35.5
1	0	0	100	6.85	1	82	21.4	134	72	185	58	127	13.6	5.3	67.6	2	0	0	1	1	1	1	17.7	0.04	18.5
1	0	0	100	7.03	1	81	22.1	123	71.5	229	58	171	12.5	5.2	72.0	1	1	1	1	0	1	1	10	0.04	12.5
1	0	0	100	5.06	1	85	23	129	66	215	58	157	14.6	6.4	49.5	2	1	0	1	1	1	1	4.1	0.04	123
1	0	0	100	4.63	1	81	27.2	107	60	206	58	148	14.6	5.1	68.1	1	1	1	1	1	1	1	34.3	0.08	42.4
1	1	0	100	6.8	2	84	23.4	178.5	69	195	58	137	12.7	5.6	55.2	2	1	1	0	0	1	1	30.4	0.11	51.1
1	0	0	100	4.63	1	72	19.4	100.5	59.5	189	58	131	13.2	4.9	72.6	1	1	0	1	1	1	1	4.8	0.11	73.9
1	0	0	100	6.98	1	79	27.7	171.5	100	230	58	172	16.5	5.3	51.6	2	1	0	0	0	1	1	281.9	0.34	34.4
1	0	0	100	6.86	1	75	25	155	85.5	180	58	122	13.9	5.5	71.0	1	1	1	0	1	1	1	17.9	0.52	31.8
1	0	0	100	6.86	1	73	17.1	137.5	87.5	166	58	108	14	5.4	72.0	2	1	0	1	1	1	1	18	0.01	3.06
1	0	0	100	6.87	1	65	27.5	106	72	176	58	118	14.2	7.4	76.2	2	1	0	1	0	0	1	22.8	0.05	5.14
1	0	0	100	5.16	1	72	19.7	145	82.5	185	58	127	12.5	6.1	48.8	2	1	1	0	0	1	1	147.5	0.01	75.3
1	0	0	100	6.95	1	65	30.7	146	90.5	255	58	197	14.2	5.3	76.2	2	1	0	0	0	1	1	660.9	0.04	14.9
1	0	0	100	6.82	1	69	18.8	107.5	60	177	58	119	13	5.1	78.3	1	1	1	1	1	1	1	6.4	0.03	48.4
1	0	0	100	6.44	1	79	20.2	111	62	193	58	135	13.2	5.1	73.0	2	1	1	1	1	1	1	8.6	0.02	61.1
1	1	0	100	6.8	1	78	22.2	143.5	80.5	229	58	171	14.8	4.7	73.5	2	0	0	0	0	1	1	8.6	0.06	28.6
1	1	0	100	7	1	75	26.4	121.5	64	183	58	125	12.2	5.1	75.1	1	1	0	1	1	1	1	9	0.02	22.5
1	0	0	100	7.05	2	75	22	133.5	75	196	58	138	11.2	5.8	72.7	2	1	1	1	1	1	1	90.2	0.15	54.2
1	0	0	100	4.58	2	68	32.7	149.5	91.5	185	58	127	13.5	5.1	61.8	2	1	1	0	1	1	1	751.5	0.46	31.4
1	0	0	100	6.86	2	76	21.6	191	99	210	58	152	13.8	5.2	58.4	2	1	1	0	1	1	1	241.1	0.1	44.2
1	0	0	100	6.87	2	65	17.8	102	62	220	58	162	10.7	5.8	78.0	2	1	1	1	0	1	1	16.5	0.12	17.8
1	0	0	100	6.87	2	78	27.1	156.5	82	221	58	163	12.7	7	67.7	2	1	1	0	0	0	1	8.8	0.55	1.46
1	0	0	100	6.8	2	73	26.8	159	89.5	275	58	217	14.2	5	70.1	1	1	1	0	0	1	1	50.4	0.19	16.6
1	0	0	100	6.84	2	72	19.5	100	56	164	58	106	11.3	5.5	74.3	1	1	1	1	1	1	1	83.8	0.37	52.9
1	0	0	100	4.59	2	69	26.3	149	79	225	58	167	13.4	5.4	53.2	2	0	1	0	0	1	1	66.6	0.17	67.8
1	0	0	100	4.62	2	72	26.8	143.5	76	244	58	186	12.8	5.5	70.6	1	1	1	0	0	1	1	21.6	0.17	69.4
1	0	0	100	5.06	2	65	25.4	139.5	85	224	58	166	13.6	5.4	78.0	2	1	1	0	0	1	1	8.7	0.22	2.51
1	0	0	100	5.04	2	66	23.6	100.5	59.5	158	58	100	14.3	5.6	77.5	2	1	1	1	1	1	1	10.4	1.59	16.2
1	0	0	100	7.05	2	67	22.3	139	86	235	58	177	13.4	5.5	76.9	2	1	1	1	0	1	1	20.2	0.01	25.5
1	0	0	100	7.4	2	72	29.2	172.5	105.5	198	58	140	13.2	5.6	70.6	2	1	1	0	1	1	1	63.2	0.04	42.9
1	0	0	100	4.58	2	75	26	179	108.5	213	58	155	14.9	5.3	77.2	2	1	1	0	1	1	1	338.1	0.02	23.5
1	0	0	100	4.58	2	69	22.6	104.5	64.5	219	58	161	12.3	5.4	75.9	2	1	1	1	1	1	1	14	0.02	10.1
1	0	0	100	6.88	2	68	24.9	122	77	232	58	174	12.2	5.9	72.6	2	1	1	0	0	1	1	3.2	0.08	44.8
1	0	0	100	6.84	2	74	24.1	127	74.5	219	58	161	13.1	5.6	73.2	1	1	1	0	1	1	1	4.8	0.01	39.7

1	0	0	100	6.85	2	69	20.8	141.5	78.5	226	58	168	13.8	5.3	72.1	1	1	1	0	0	1	1	4.5	0.06	23.1
1	0	0	100	7.02	2	67	22.1	126	71.5	150	58	92	12.5	5.7	76.9	2	1	1	1	1	1	1	4.5	0.05	27.1
1	0	0	100	7.03	2	76	23.1	119	83.5	171	58	113	12.6	5.3	68.6	1	1	1	1	1	1	1	36.7	0.04	44.7
1	0	0	100	5.05	2	73	25.2	153.5	89.5	169	58	111	13.1	5.1	73.8	2	1	0	0	1	1	1	95	0.03	53.7
1	0	0	100	7	2	70	28.7	116.5	69	204	58	146	14.3	5	75.3	1	1	1	0	1	1	1	47	0.04	27.5
1	0	0	100	7	2	66	18.3	105.5	70	147	58	89	15.2	5.1	82.3	2	1	1	1	1	1	1	29	0.01	20
1	0	0	100	4.63	2	67	25	105.5	74	161	58	103	12.1	5.9	73.1	2	1	0	0	1	1	1	222.5	0.05	14.7
1	0	0	100	4.64	2	75	25.2	146	88	193	58	135	13.2	4.9	72.7	2	1	1	0	1	1	1	16	0.04	29.3
1	0	0	100	6.95	2	72	21.5	114	68.5	229	58	171	12	5.4	70.6	2	1	1	1	0	1	1	20.2	0.01	19
1	0	0	100	6.8	2	65	25.3	129.5	78.5	200	58	142	12.1	5.2	74.2	2	1	1	1	1	1	1	10.6	0.08	14.9
1	0	0	100	6.84	2	65	23.8	149.5	84	175	58	117	11.2	5	82.8	2	1	1	0	1	1	1	17	0.01	54.5
1	0	0	100	4.6	2	75	26.5	124.5	74	200	58	142	13.1	6.2	77.2	2	1	1	1	1	0	1	58.1	0.02	34.5
1	0	0	100	4.61	2	68	29.9	123	70.5	233	58	175	14.5	5.4	76.4	2	1	1	1	0	1	1	37.7	0.05	12.1
1	0	0	100	4.62	2	67	28.7	104	59	182	58	124	14.4	5	81.7	2	1	1	0	1	1	1	48.5	0.07	8.67
1	0	0	100	4.69	2	78	23.2	121	62.5	176	58	118	13.8	5.9	71.2	2	1	1	0	1	1	1	13	0.06	2.86
1	0	0	100	4.69	2	69	25.4	125.5	73	164	58	106	12.1	5.4	75.9	1	1	1	0	1	1	1	54.3	0.03	1.98
1	0	0	100	6.97	2	76	29.9	177.5	93	228	58	170	14.4	6.1	72.2	2	1	1	0	0	1	1	14.2	0.06	0.05
1	0	0	100	6.97	2	73	22.3	115.5	68.5	181	58	123	12.7	4.8	78.3	1	1	1	1	1	1	1	22.5	0.01	16.8
1	0	0	100	5.05	2	76	25.6	151	88	210	58	152	12.6	5.6	72.2	1	1	1	0	1	1	1	24.8	0.01	31.9
1	0	0	100	5.06	2	71	20.7	89	60	199	58	141	11.5	5.5	60.5	1	1	1	1	1	1	1	20.4	0.01	81.6
1	0	0	100	5.06	2	68	22.7	132	80.5	202	58	144	13.2	5.7	76.4	2	1	1	1	0	1	1	4.8	0.03	15.5
1	0	0	100	5.04	2	70	26.6	135	86	197	58	139	13.5	5.7	75.3	1	1	1	0	1	1	1	19.3	0.02	66.7
1	0	0	100	5.04	2	69	30.3	134	66.5	189	58	131	13.7	5.8	80.5	2	1	1	1	1	1	1	5.7	0.06	69
1	1	0	100	6.98	2	70	28.4	134.5	69	199	58	141	13.1	6.1	75.3	1	1	1	0	0	1	1	857.1	0.18	80.1
1	1	0	100	6.44	2	74	28.8	129	74.5	199	58	141	13.1	5.5	73.2	2	1	1	1	1	1	1	23.9	0.15	32.4
1	1	0	100	6.87	2	79	26.6	113.5	72	263	58	205	15.3	5.5	70.7	1	1	1	1	0	1	1	16.5	0.15	15.4
1	1	0	100	7.07	2	77	23.8	140.5	71	194	58	136	12.4	5.5	76.1	1	1	1	0	1	1	1	154	0.05	259
1	1	0	100	4.67	2	77	19.6	117	81.5	221	58	163	11.1	5.3	58.0	2	1	1	1	0	1	1	6.2	0.03	38
1	1	0	100	2.99	2	71	20.5	122.5	73.5	216	58	158	12.5	5.2	79.4	2	1	1	1	1	1	1	11	0.05	28.3
1	1	0	100	5.05	2	79	21	127.5	67.5	155	58	97	11.5	4.3	70.7	2	1	1	0	1	1	1	21	0.05	73.5
1	1	0	100	5.06	2	77	24.1	100.5	53	176	58	118	11.3	7	50.3	1	1	1	0	1	0	1	75	0.08	97.9
1	0	0	100	3.03	1	82	19.4	162	90	212	58	154	15	5.9	50.6	2	1	0	0	1	1	1	56.2	0.31	56.6
1	0	0	100	5.07	1	80	22.7	101.5	58.5	188	58	130	11.8	5.1	57.5	2	1	1	0	1	1	1	2.3	0.05	26.5
1	0	0	100	4.61	1	80	22.9	172	93.5	183	58	125	12.9	6.1	65.4	2	1	0	0	1	1	1	72.1	0.04	37.5
1	0	1	1	1	1	65	27.5	123	77	188	58	130	13.9	4.8	57.0	2	0	0	1	1	1	1	14.6	0.14	61.1
1	0	1	2.2	2.2	1	71	22.5	116.5	66.5	155	58	97	14.9	5.6	73.1	2	1	1	1	1	1	1	25	0.06	78.9
1	0	1	2.7	2.7	1	71	20.5	151.5	75	145	58	87	14.2	5.4	77.2	2	0	0	0	1	1	1	47.5	0.02	36
1	1	1	6.1	6.1	1	77	20	106	61	193	58	135	13.5	5.4	74.0	1	0	0	1	1	1	1	23	0.04	1.8
1	0	1	3.5	3.5	2	72	25.8	139.5	74.5	219	58	161	13.5	5.3	74.3	2	1	1	0	1	1	1	9.2	0.04	12.3
1	0	1	2.9	2.9	2	71	24	132.5	75	179	58	121	12.1	5.4	71.1	2	1	1	0	1	1	1	6.3	0.09	41.3
1	1	1	3.1	3.1	2	71	21.2	156.5	79.5	162	58	104	14	5.2	71.1	1	1	1	0	1	1	1	91.8	0.58	311
1	1	1	4.3	4.3	2	67	19.9	136.5	76	169	58	111	12.7	5.3	76.9	2	1	1	1	1	1	1	20.1	0.01	14.1
1	0	0	100	7.08	1	69	25.2	137	82.5	155	59	96	15.6	5.1	55.4	2	0	0	1	1	1	1	40.1	0.27	47
1	0	0	100	4.63	1	67	22.8	152	88	133	59	74	15.1	5.8	75.2	2	1	0	0	1	1	1	3.8	0.11	54.4
1	0	0	100	6.83	1	76	22.9	162.5	113	201	59	142	14.6	5	47.5	2	1	1	0	1	1	1	1134.3	0.27	25.1
1	0	0	100	4.62	1	75	24	160	82	237	59	178	16.4	5.7	71.0	2	0	0	0	0	1	1	45.1	0.32	33.5
1	0	0	100	6.44	1	70	27.6	120.5	80.5	215	59	156	15	5.3	77.7	2	1	0	1	1	1	1	25.3	0.12	13.6
1	0	0	100	4.69	1	68	23.7	139.5	83	166	59	107	15.1	5.3	84.0	2	1	0	0	1	1	1	13.4	0.37	9.45
1	0	0	100	5.07	1	75	29	169.5	93	245	59	186	14.4	6.5	71.0	2	1	0	0	0	0	1	95.9	0.21	23.2
1	0	0	100	6.87	1	66	23.4	141.5	56	170	59	111	14.1	5.1	79.9	2	1	0	0	1	1	1	315.4	0.56	65.3
1	0	0	100	4.26	1	70	26.6	127	72.5	115	59	56	11.5	4.5	73.6	2	1	0	1	1	1	1	4.2	0.38	2.38

1	0	0	100	7	1	65	24.1	126	88.5	227	59	168	16.4	5.4	76.2	2	1	1	1	0	1	1	35.2	0.03	0.05
1	0	0	100	7.05	1	69	23.3	115.5	73	184	59	125	14.5	5.1	78.3	2	1	0	1	1	1	1	147	0.07	26.5
1	0	0	100	4.58	1	70	24	117	71.5	224	59	165	15.5	5.4	73.6	2	1	0	0	0	1	1	13.2	0.02	25.2
1	0	0	100	6.86	1	73	21.5	159.5	77.5	124	59	65	13.6	5	76.1	2	1	0	0	1	1	1	18.1	0.06	4.7
1	0	0	100	6.87	1	67	26.2	126.5	84	230	59	171	13.4	5.5	75.2	2	1	1	0	0	1	1	77.8	0.01	15.9
1	0	0	100	6.87	1	71	25.9	148	88	192	59	133	16.3	5.5	82.2	2	1	0	0	1	1	1	41.8	0.04	25
1	0	0	100	6.88	1	71	24.9	130	82	253	59	194	14.6	5.2	69.6	2	1	0	1	0	1	1	10.8	0.03	54.8
1	0	0	100	5.16	1	77	26.1	151.5	89.5	205	59	146	14.5	6.8	70.1	2	1	1	0	1	0	1	22.3	0.09	34.1
1	0	0	100	6.85	1	70	19.8	98	67.5	196	59	137	14.5	5	73.6	2	1	0	1	1	1	1	5	0.01	9.07
1	0	0	100	6.86	1	66	19.5	101.5	62.5	204	59	145	14.3	5.2	79.9	2	1	0	1	1	1	1	9.5	0.03	18.6
1	0	0	100	6.86	1	72	21.5	111.5	73	169	59	110	13.7	5.1	69.1	2	1	0	0	1	1	1	7.4	0.03	29.2
1	0	0	100	7.01	1	65	21.8	158	90.5	186	59	127	14.9	5.2	76.2	2	1	0	0	1	1	1	5	0.02	5.64
1	0	0	100	7.02	1	73	26.5	157	84.5	190	59	131	10.2	5.7	72.0	2	1	0	0	1	1	0	5.9	0.04	211
1	0	0	100	7.03	1	70	21.4	101	59.5	200	59	141	15.3	5.5	73.6	2	1	0	1	1	1	1	16.4	0.04	7.66
1	0	0	100	7.03	1	71	21.6	106.5	70	226	59	167	15.5	5.8	77.2	2	1	1	1	0	1	1	5.5	0.02	0.21
1	0	0	100	7.04	1	69	19.8	122	71	143	59	84	12.9	5.9	89.9	2	0	1	1	1	0	1	18.6	0.01	33.4
1	0	0	100	5.07	1	65	24.2	115	65.5	180	59	121	13.7	5.3	80.5	2	0	0	1	1	1	1	11.6	0.04	29.5
1	0	0	100	5.08	1	70	23.6	123.5	77.5	209	59	150	14	6.1	73.6	1	1	1	1	1	1	1	3.7	0.05	4.89
1	0	0	100	4.63	1	66	26.2	133.5	85	168	59	109	14.1	5.7	75.7	2	1	0	0	1	1	1	9.9	0.01	10.3
1	0	0	100	1.62	1	78	21.1	132.5	85	144	59	85	12.2	4.8	66.3	2	1	1	0	1	1	1	19.1	0.05	135
1	0	0	100	4.63	1	66	23.3	162	94.5	175	59	116	16	5.6	75.7	2	1	0	0	1	1	1	29.2	0.01	12.1
1	0	0	100	4.64	1	69	27	146	81	225	59	166	15.5	5.9	70.6	2	1	0	0	0	1	1	4.4	0.03	25
1	0	0	100	4.64	1	70	24	161	82.5	140	59	81	12.1	5.7	70.1	2	0	0	0	1	1	1	7.5	0.08	41.4
1	0	0	100	6.96	1	67	23.2	127.5	72.5	209	59	150	14	5.1	71.6	2	1	0	1	1	1	1	6	0.04	9.97
1	0	0	100	6.98	1	69	21.6	106	65.5	158	59	99	12.6	5.4	70.6	1	1	0	1	1	1	1	32	0.02	30.4
1	0	0	100	6.84	1	67	21.9	133.5	75	169	59	110	13.2	5.3	75.2	2	1	0	1	1	1	1	6.7	0.03	18.4
1	0	0	100	4.69	1	73	21.5	106	69	172	59	113	12.7	5.3	81.1	2	1	1	1	1	1	1	3.3	0.05	32.7
1	0	0	100	5.06	1	66	26.6	143	82	209	59	150	15	6	75.7	2	1	0	0	1	1	1	20.3	0.04	12.2
1	0	0	100	6.86	1	68	22.6	171.5	95	210	59	151	15.1	5.7	74.6	2	1	1	0	1	1	1	13.5	0.03	25.3
1	0	0	100	6.87	1	76	24.9	116.5	78	229	59	170	16	4.8	67.2	2	1	0	1	0	1	1	10.3	0.06	8.7
1	0	0	100	5.57	1	77	23.8	113.5	64	152	59	93	13.2	4.4	52.4	2	1	1	1	1	1	1	32.4	0.04	64.1
1	0	0	100	6.87	1	66	24.9	145.5	95.5	187	59	128	16.4	5.4	75.7	2	1	0	0	1	1	1	7.2	0.04	17.1
1	0	0	100	6.87	1	71	23.9	118	74.5	185	59	126	15.2	5.5	69.6	2	1	0	1	0	1	1	11.4	0.01	6.33
1	0	0	100	5.04	1	76	20.4	120.5	78	122	59	63	10	5.2	67.2	2	1	1	1	1	1	1	4.2	0.06	82.4
1	1	0	100	6.84	1	77	22.5	108.5	62	193	59	134	13.7	5.2	52.4	2	1	1	1	1	1	1	41.4	0.02	22.6
1	1	0	100	6.8	1	73	22.9	128.5	78	156	59	97	15.5	5.2	81.1	2	1	0	1	1	1	1	6.7	0.07	25.2
1	1	0	100	6.84	1	77	24.4	145	80.5	136	59	77	14.5	5.4	66.7	2	1	1	0	1	1	1	12.7	0.02	20.1
1	0	0	100	7.07	2	67	25.8	151	79.5	195	59	136	13.6	6	73.1	2	1	1	0	0	1	1	24.1	0.72	15.5
1	0	0	100	6.84	2	74	27.7	93	61.5	191	59	132	12.3	5.3	69.6	2	1	1	1	0	1	1	24.1	0.13	51.2
1	0	0	100	4.63	2	74	31	144	86.5	198	59	139	12.9	6	69.6	1	1	1	0	1	1	1	27.6	0.11	48.2
1	0	0	100	6.95	2	71	24.2	118	60.5	171	59	112	13.3	7	79.4	2	1	1	1	0	0	1	25.8	0.72	28.3
1	0	0	100	6.8	2	65	23	128	69.5	200	59	141	13.1	5.3	74.2	2	1	0	1	1	1	1	14.2	0.12	43.1
1	0	0	100	6.79	2	69	28	183	99.5	187	59	128	12.7	5.7	72.1	2	1	1	0	1	1	1	7.1	0.11	10.5
1	0	0	100	5.04	2	77	25.8	146	78	175	59	116	13	5.8	71.7	2	1	1	0	1	1	1	14.5	0.12	26.9
1	0	0	100	7.07	2	77	19.8	138	71	197	59	138	11.8	4.9	68.2	1	1	1	1	1	1	1	15.7	0.03	54.9
1	0	0	100	7.07	2	66	20.7	106	66	220	59	161	12.1	5.6	77.5	2	1	1	1	0	1	1	17	0.01	2.55
1	0	0	100	7.08	2	76	19	110.5	62	231	59	172	13.5	5.5	72.2	1	1	1	1	0	1	0	21.3	0.02	207
1	0	0	100	4.58	2	77	19.8	109	56	188	59	129	14.1	5.3	71.7	2	1	1	0	1	1	1	19.8	0.02	121
1	0	0	100	4.59	2	69	20.1	116	82	243	59	184	12.7	5.4	75.9	2	1	1	1	0	1	1	13.6	0.03	14.5
1	0	0	100	6.86	2	70	23.2	130	70	251	59	192	13.8	5	60.9	2	1	1	1	0	1	1	7.4	0.05	25.5
1	0	0	100	6.86	2	72	24	122	66	180	59	121	12.1	4.9	60.1	2	1	1	0	1	1	1	15.6	0.02	59.9

1	0	0	100	6.87	2	66	19.6	109	63.5	205	59	146	15	7.4	73.6	2	1	1	1	1	0	1	16.8	0.02	39.5
1	0	0	100	5.15	2	73	23.1	124.5	74.5	231	59	172	13.2	5.7	70.1	2	1	1	1	0	1	1	20.6	0.01	93.1
1	0	0	100	5.15	2	69	20.6	127	71.5	162	59	103	11.8	5.3	75.9	1	1	1	0	1	1	1	10.5	0.03	32.3
1	0	0	100	5.16	2	66	25.1	111.5	65.5	225	59	166	14.7	5.6	62.7	2	1	1	0	0	1	1	21.3	0.04	51.7
1	0	0	100	6.84	2	75	26.9	117	66	184	59	125	13.9	5.8	69.1	2	1	1	1	1	1	1	26.8	0.05	37.7
1	0	0	100	6.84	2	68	25.6	160	87	218	59	159	15.4	5.3	76.4	2	1	1	0	1	1	1	10	0.04	36.3
1	0	0	100	7.21	2	74	18	136	80	173	59	114	12.9	5	73.2	2	1	1	1	1	1	1	18	0.01	21.6
1	0	0	100	7.03	2	70	31.2	132.5	72.5	227	59	168	13.8	6.4	71.6	2	1	1	1	0	1	1	13.2	0.07	23.4
1	0	0	100	5.05	2	66	25.3	117	69.5	216	59	157	12.7	5.5	73.6	2	1	1	1	1	1	1	397.3	0.04	13.8
1	0	0	100	5.05	2	65	24.9	113	70.5	186	59	127	12.5	5.3	74.2	2	1	1	1	1	1	1	4.6	0.03	27.9
1	0	0	100	5.06	2	71	24.3	105	67	192	59	133	12.8	5.6	71.1	2	1	1	1	1	1	1	8.6	0.05	15.1
1	0	0	100	5.06	2	70	20.9	155.5	86	234	59	175	12.9	5.4	80.0	1	1	1	0	0	1	1	4.7	0.01	37.2
1	0	0	100	5.08	2	70	24.5	147.5	77.5	163	59	104	11.8	5.3	75.3	2	1	1	0	1	1	1	11.3	0.03	31.7
1	0	0	100	6.98	2	67	26	115	70.5	161	59	102	12.4	5.6	62.2	2	1	1	0	0	1	1	4.9	0.07	0.05
1	0	0	100	7	2	70	25.5	106	63	187	59	128	12.2	5.1	71.6	2	1	1	1	1	1	1	5.3	0.07	13
1	0	0	100	7	2	74	20.6	142	85.5	235	59	176	12.5	5.5	77.8	2	1	1	0	0	1	1	120.2	0.04	35.8
1	0	0	100	4.63	2	71	32	144.5	70.5	177	59	118	12.9	5.7	71.1	1	1	1	0	1	1	1	30.6	0.03	67.7
1	0	0	100	0.94	2	76	21.6	106.5	57	186	59	127	11.9	5.4	68.6	1	1	1	1	1	1	1	11	0.02	112
1	0	0	100	4.64	2	76	21.8	143.5	73.5	174	59	115	12.8	5.1	72.2	1	1	1	0	1	1	1	4.7	0.02	79
1	0	0	100	4.64	2	70	22.8	107	59.5	201	59	142	12	6.1	75.3	2	1	1	1	1	1	1	14.8	0.04	60.8
1	0	0	100	6.95	2	74	27.2	151	79	264	59	205	13.7	6.3	77.8	2	1	1	0	0	1	1	98.5	0.05	3.67
1	0	0	100	6.8	2	68	22.2	107	60	157	59	98	11.8	7.7	76.4	2	1	1	1	1	0	1	13.4	0.04	21.5
1	0	0	100	6.8	2	69	24.8	112	70	225	59	166	13.9	5.9	61.4	1	1	1	1	0	1	1	8.8	0.02	9.62
1	0	0	100	6.84	2	65	19.6	112.5	65.5	152	59	93	12	5.4	78.0	2	1	1	0	1	1	1	6.2	0.01	23.7
1	0	0	100	4.59	2	69	23.2	152	93	155	59	96	13.2	5.4	80.5	1	1	1	0	0	1	1	28.9	0.06	24.7
1	0	0	100	0	2	71	26.2	125	76	197	59	138	12.9	5.5	79.4	2	1	1	1	1	1	1	15.8	0.05	36
1	0	0	100	4.62	2	67	23.8	122	67	206	59	147	12.5	5.1	73.1	2	1	1	1	1	1	1	10.4	0.02	18.2
1	0	0	100	6.96	2	72	23.3	152	72.5	193	59	134	11.9	5.3	70.6	2	1	1	0	1	1	1	24.8	0.05	0.05
1	0	0	100	5.36	2	75	27.7	134.5	72.5	168	59	109	14.5	5.8	69.1	1	1	1	0	0	1	1	16.5	0.05	24.3
1	0	0	100	5.37	2	65	22.4	132.5	87.5	207	59	148	13.4	4.9	82.8	2	1	1	1	1	1	1	112.6	0.02	22.7
1	0	0	100	5.06	2	67	22.2	144	83	259	59	200	12.9	5.4	81.7	2	1	1	0	0	1	1	25.9	0.03	13.9
1	0	0	100	6.87	2	70	25.9	136.5	77.5	201	59	142	13	4.9	80.0	2	1	1	1	1	1	1	40.1	0.01	10.5
1	0	0	100	5.04	2	67	23	166	92	201	59	142	12.4	5.3	81.7	2	1	1	0	1	1	1	112.1	0.08	31.5
1	0	0	100	5.04	2	79	27.6	145.5	74.5	245	59	186	11.8	5.8	75.1	2	1	1	0	0	1	1	43.1	0.09	38.7
1	0	0	100	5.04	2	72	25	103.5	66	214	59	155	13.2	5.7	74.3	1	1	1	1	1	1	1	8.8	0.04	55.7
1	1	0	100	6.87	2	69	22.7	149	71.5	238	59	179	13.8	5.5	61.4	2	1	1	0	0	1	1	9.8	0.01	55.6
1	1	0	100	5.37	2	76	25.2	153	92.5	224	59	165	13.8	5.4	58.4	1	1	1	0	0	1	1	19.9	0.02	18.1
1	0	0	100	6.71	1	83	26	143	63	209	59	150	14.2	5.1	71.0	2	1	1	0	1	1	1	6.3	0.03	48.3
1	1	0	100	6.95	1	83	24.1	164	89	182	59	123	14.3	4.9	25.9	2	0	0	0	1	1	1	391.1	0.04	107
1	0	0	100	5.07	2	82	26.5	100	63	175	59	116	13.5	5.4	56.0	1	1	1	1	1	1	1	4.1	0.07	76.7
1	0	0	100	5.07	1	78	25	125	75	204	59	145	13.9	5.3	52.0	2	1	0	1	1	1	1	2	0.06	16.5
1	0	0	100	6.98	1	68	21.2	102.5	69	159	59	100	14.7	5.3	78.8	2	0	0	1	1	1	1	11.6	0.07	6.86
1	0	0	100	4.61	1	71	23.4	120	73	149	59	90	14.6	7.2	69.6	1	1	0	1	1	0	1	23.5	0.02	38.3
1	0	0	100	4.78	1	73	18.8	101	70	149	59	90	13	4.8	81.1	2	1	0	1	1	1	1	31.5	0.03	25.2
1	1	0	100	6.87	1	73	23.6	151	87	219	59	160	14	5.1	72.0	2	1	1	0	1	1	1	12.2	0.4	20.5
1	1	0	100	6.98	1	73	18.8	130	80	137	59	78	12.7	4.9	68.6	2	1	1	1	1	1	1	291.3	0.01	48.8
1	0	0	100	7.08	2	67	28.9	137	83.5	236	59	177	13.5	5.7	76.9	2	1	1	1	0	1	1	619.8	0.1	0.05
1	0	0	100	4.58	2	70	32.5	118	67.5	236	59	177	14.2	6.1	71.6	2	1	1	0	0	1	1	81.1	0.37	7.82
1	0	0	100	6.87	2	69	25.9	148.5	85	245	59	186	14.4	4.9	72.1	2	1	1	0	0	1	1	17.9	0.12	20.1
1	0	0	100	7.04	2	65	18.6	116	73.5	148	59	89	11	5.4	89.1	2	1	1	0	1	1	1	25.9	1.37	10.5
1	0	0	100	4.67	2	71	23.2	139.5	72.5	192	59	133	11.8	5.3	74.8	2	1	0	0	1	1	1	26	0.16	63.5

1	0	0	100	5.06	2	71	28.8	151	82	162	59	103	12.2	6.5	74.8	2	1	1	0	1	0	1	35.2	0.27	26.7
1	0	0	100	5.06	2	70	25.5	144	74.5	164	59	105	12.1	6.2	71.6	2	1	1	0	1	1	1	42	1.47	25
1	0	0	100	7.08	2	75	20.1	106.5	61	134	59	75	11.3	5.6	72.7	2	1	1	1	1	1	1	20.1	0.04	14.9
1	0	0	100	4.59	2	76	21.8	108	64	193	59	134	12.8	5.5	76.7	1	1	1	0	1	1	1	13.5	0.03	15.4
1	0	0	100	4.59	2	79	19.3	113.5	65	148	59	89	12.3	5.4	43.7	2	1	1	1	1	1	1	12	0.04	43.2
1	0	0	100	4.59	2	65	39	129	85	187	59	128	13.7	5.5	78.0	2	1	1	0	0	1	1	25.4	0.05	44.1
1	0	0	100	6.86	2	65	21.6	128.5	85.5	249	59	190	13.5	5.5	78.0	2	1	0	1	0	1	0	24.5	0.02	116
1	0	0	100	5.15	2	71	30.6	148.5	84	222	59	163	13.6	5.7	71.1	1	1	1	0	0	1	1	45.9	0.06	41.2
1	0	0	100	5.16	2	70	27.7	138.5	71	217	59	158	12.9	5.8	71.6	2	1	1	0	1	1	1	14.4	0.07	59.6
1	0	0	100	5.16	2	65	25	101.5	67.5	202	59	143	13.7	5.1	74.2	2	1	1	1	1	1	1	22.3	0.07	2.11
1	0	0	100	5.16	2	69	21.7	151	80.5	225	59	166	13.9	5.5	80.5	2	1	1	0	0	1	1	61.3	0.01	39.9
1	0	0	100	4.44	2	76	19.1	130.5	70	206	59	147	11.8	4.9	68.6	2	1	1	0	1	1	1	136.1	0.01	59.4
1	0	0	100	6.86	2	73	21.3	125	68	199	59	140	10.9	5.6	73.8	2	1	1	1	1	1	1	18	0.01	36.1
1	0	0	100	7.02	2	78	22.5	111	71	221	59	162	12.8	5.6	75.6	2	1	1	1	0	0	1	48.8	0.04	16.6
1	0	0	100	7.02	2	65	25.7	114.5	72	179	59	120	12.8	5	63.1	2	1	1	1	1	1	1	5.7	0.03	16.4
1	0	0	100	5.06	2	69	24.8	122.5	71.5	195	59	136	13.1	6	61.4	2	1	1	1	1	1	1	9	0.08	8.28
1	0	0	100	5.08	2	65	30.5	104	60.5	156	59	97	11.4	5.4	82.8	2	1	1	0	1	1	1	19.1	0.06	49.9
1	0	0	100	6.1	2	67	22.7	115	67.5	195	59	136	12.2	5.6	76.9	2	1	1	1	1	1	1	9.3	0.01	10.2
1	0	0	100	4.62	2	72	23.5	111	74.5	161	59	102	14.9	5.6	74.3	2	1	1	1	1	1	1	5.5	0.03	7.29
1	0	0	100	4.64	2	71	25.5	113.5	60	166	59	107	13.9	4.9	79.4	1	1	1	0	1	1	1	14.4	0.04	11.4
1	0	0	100	6.87	2	67	28.3	147	99	213	59	154	13.8	4.7	76.9	2	1	0	0	1	1	1	16.9	0.04	5.93
1	0	0	100	6.83	2	76	20.7	157.5	75.5	218	59	159	13.6	4.9	76.7	1	1	1	0	1	1	1	27.7	0.02	35.6
1	0	0	100	6.84	2	65	24.5	170	101	209	59	150	11	5.8	78.0	2	1	1	0	1	1	1	93.8	0.04	10.2
1	0	0	100	4.62	2	74	23.6	94	67	202	59	143	13	5.1	59.2	1	1	1	1	1	1	1	18.2	0.02	11.7
1	0	0	100	6.79	2	75	24.6	134	77.5	168	59	109	14.2	5.9	69.1	2	1	0	0	1	1	1	9.8	0.06	32.8
1	0	0	100	4.68	2	76	33.8	164	84.5	226	59	167	13.1	9	76.7	1	1	1	0	0	0	1	176.4	0.04	26.5
1	0	0	100	6.96	2	77	21.2	161.5	90.5	162	59	103	11.8	5.6	71.7	1	1	1	0	1	1	1	9.2	0.04	0.84
1	0	0	100	5.37	2	71	25	130	69.5	158	59	99	11.5	5.4	74.8	1	1	0	0	1	1	1	107.7	0.03	71.7
1	0	0	100	6.86	2	66	28.1	143.5	91.5	231	59	172	13.9	5.2	77.5	2	1	1	0	0	1	1	8	0.04	11.3
1	0	0	100	6.87	2	76	23.3	123.5	60	204	59	145	11.4	3.7	68.6	2	1	1	1	1	1	1	53	0.09	108
1	0	0	100	5.04	2	68	29.1	154.5	91	192	59	133	13.2	8	76.4	2	1	0	0	1	0	1	16	0.07	25.5
1	0	0	100	5.04	2	65	23.9	105	61	183	59	124	12.9	5.2	82.8	2	1	1	1	1	1	1	7.1	0.02	19
1	1	0	100	6.84	2	71	28.7	184	98	247	59	188	10.2	5.5	26.2	2	1	1	0	0	1	1	1112.3	0.34	60
1	1	0	100	7	2	76	20	141.5	79.5	219	59	160	11.5	5.2	68.6	1	1	1	0	1	1	1	306.2	0.03	52.3
1	1	0	100	6.86	2	78	25.8	137.5	75	185	59	126	13.5	6.9	71.2	2	1	0	0	1	0	1	54.4	0.07	75.2
1	1	0	100	6.87	2	75	26.6	135.5	78.5	185	59	126	12.6	5.3	77.2	1	1	1	0	1	1	1	219.1	0.06	45.9
1	1	0	100	6.8	2	78	17.4	109.5	66.5	178	59	119	12.9	5.6	67.7	1	1	1	1	1	1	1	20.9	0.05	39.3
1	1	0	100	5.37	2	77	23.2	110	69.5	197	59	138	12.6	5	50.3	1	1	1	1	1	1	1	10.4	0.01	27.4
1	1	0	100	6.87	2	74	27.6	149.5	95	191	59	132	12.2	5.7	77.8	2	1	1	0	1	1	1	27.7	0.04	8.43
1	1	0	100	6.87	2	69	22.3	128.5	83	191	59	132	14.8	6.3	75.9	1	1	1	1	0	1	1	14.7	0.06	58.8
1	0	1	1.7	1.7	1	70	21.9	108.5	68.5	173	59	114	13.6	5.5	55.0	2	0	1	1	1	1	1	4.1	1.21	49.2
1	0	1	0.7	0.7	1	74	21	134	77.5	181	59	122	12.1	7.3	71.5	1	1	0	0	1	0	1	8.2	0.09	98.9
1	0	1	1.1	1.1	1	68	25.1	138.5	74	175	59	116	15.1	6.6	71.1	2	1	0	0	1	0	0	66.8	0.03	486
1	1	1	6.3	6.3	1	69	24	129	82	174	59	115	16.8	5.8	70.6	2	0	0	0	1	1	0	21.6	0.2	82.3
1	1	1	1.8	1.8	1	72	27.2	155	89	228	59	169	14.3	5.5	72.6	2	1	0	0	0	1	1	27.9	0.52	35.3
1	0	1	1.2	1.2	2	68	22.6	152.5	81.5	240	59	181	12.2	5.2	72.6	1	1	1	0	0	1	1	47.3	0.02	179
1	1	1	4.9	4.9	2	83	17.3	174	89.5	221	59	162	15.1	5.6	68.7	2	1	1	0	0	0	1	342.4	0.01	14.3
1	0	0	100	7.06	1	73	22.1	136	78.5	136	60	76	13.8	5.3	68.6	1	1	0	0	1	1	1	97.7	0.6	22
1	0	0	100	7.08	1	69	25.3	162	85	206	60	146	14.5	6.1	78.3	2	1	0	0	1	1	1	19.2	0.4	10
1	0	0	100	7.08	1	77	26.2	116	76.5	210	60	150	14.8	5.3	66.7	2	1	0	0	1	1	1	15.5	0.12	4.91
1	0	0	100	6.87	1	76	23.2	156.5	73.5	212	60	152	14.9	5.6	70.5	2	1	0	0	0	1	1	6.3	0.14	29

1	0	0	100	5.26	1	74	18.3	151.5	84.5	195	60	135	12.1	5.4	53.5	2	1	1	0	1	1	1	95.1	0.11	26.2
1	0	0	100	6.97	1	71	24.9	108	62.5	188	60	128	13.6	5	73.1	2	1	1	0	1	1	1	10.6	0.12	0.05
1	0	0	100	6.8	1	72	22.7	136.5	78.5	223	60	163	15.3	5.2	69.1	2	1	1	1	0	1	1	8.4	0.56	2.21
1	0	0	100	6.83	1	70	22.9	128	73.5	214	60	154	16.7	5.9	61.7	2	1	0	1	1	1	1	236.6	0.42	234
1	0	0	100	4.6	1	67	21.2	161	69.5	187	60	127	15.3	5.4	75.2	2	0	0	0	1	1	1	27.8	0.39	40.1
1	0	0	100	3.08	1	73	19.1	117	53.5	221	60	161	12.8	5.5	68.6	1	1	0	1	0	1	1	21.4	0.11	16.8
1	0	0	100	6.44	1	65	21.8	96	70.5	177	60	117	14.9	4.6	72.6	2	1	1	1	1	1	1	8.7	0.11	14.5
1	0	0	100	5.36	1	71	23.3	125	78	198	60	138	14.3	5.4	77.2	2	1	0	0	1	1	1	23.4	0.34	18.5
1	0	0	100	5.04	1	68	28.6	122	83	188	60	128	13.7	5.1	90.5	2	1	0	0	1	1	1	25.1	0.13	7.48
1	0	0	100	5.04	1	74	21	155.5	80	159	60	99	14.5	4.9	60.0	2	0	0	0	1	1	1	40.6	0.14	68.3
1	0	0	100	7.07	1	73	26.8	134	78	189	60	129	14.4	5.3	60.4	2	1	0	1	1	1	1	7.3	0.05	0.05
1	0	0	100	7.07	1	74	26.8	116	75.5	219	60	159	16.6	6.6	71.5	2	1	1	1	1	0	1	21.2	0.08	2.66
1	0	0	100	4.59	1	74	20	120.5	68	175	60	115	13.6	4.9	53.5	2	1	0	0	1	1	1	60.8	0.01	13.4
1	0	0	100	6.86	1	66	26.5	99	57	212	60	152	11.6	5.4	79.9	2	1	0	1	0	1	1	7.7	0.02	31.5
1	0	0	100	6.86	1	71	20.5	133.5	84	186	60	126	14	4.7	73.1	2	1	0	1	1	1	1	58.6	0.09	32.2
1	0	0	100	6.88	1	78	24.8	144.5	77	197	60	137	15.3	5	66.3	1	1	1	0	1	1	1	15.3	0.08	31
1	0	0	100	5.16	1	69	19.3	129.5	83.5	164	60	104	14	5.2	70.6	2	1	0	0	1	1	1	14.5	0.01	14.1
1	0	0	100	5.16	1	73	24.4	117	69	184	60	124	12.8	6.4	53.9	2	1	1	1	1	1	1	10.8	0.04	2.54
1	0	0	100	6.84	1	70	20.3	130.5	64	234	60	174	15.8	5.7	77.7	2	1	1	1	0	1	1	17.8	0.03	7.93
1	0	0	100	6.84	1	77	21.2	107.5	72	165	60	105	13.5	5.6	52.4	2	1	0	1	1	1	1	19.8	0.06	14.3
1	0	0	100	6.85	1	71	21.5	132.5	74.5	182	60	122	14.1	5.3	73.1	2	1	0	1	1	1	1	14.9	0.05	75.7
1	0	0	100	6.85	1	69	23.4	132.5	74	192	60	132	13.6	5.4	74.1	2	0	0	1	1	1	1	7.2	0.09	26.9
1	0	0	100	5.6	1	71	23	136	80	189	60	129	15	11	73.1	2	1	1	1	1	0	0	203.4	0.02	235
1	0	0	100	6.86	1	69	19.6	92.5	55.5	186	60	126	13.4	4.8	78.3	2	1	0	1	1	1	1	39.8	0.05	10.8
1	0	0	100	6.86	1	69	19.6	117	64.5	186	60	126	14.4	5.1	70.6	2	1	0	0	1	1	1	166.5	0.05	48.5
1	0	0	100	7.01	1	78	28.1	116	74.5	190	60	130	15.4	5.1	73.5	2	1	0	0	1	1	1	8.8	0.02	8.73
1	0	0	100	5.05	1	72	26.4	109.5	64	143	60	83	12.2	5.2	72.6	2	1	1	1	1	1	1	9.3	0.04	27.7
1	0	0	100	5.07	1	74	24.7	124	88	267	60	207	15.7	5.2	60.0	2	1	0	1	0	1	1	7.4	0.05	13.3
1	0	0	100	4.64	1	67	26.6	144	82	190	60	130	15.9	4.6	71.6	2	1	1	0	1	1	1	13	0.02	26
1	0	0	100	6.87	1	74	23.5	159.5	93	223	60	163	15.3	6.5	68.2	2	1	0	0	0	0	1	172.7	0.04	0.6
1	0	0	100	6.95	1	73	30.9	158	85	204	60	144	15.3	8	72.0	2	1	0	0	1	0	1	141.2	0.07	5.18
1	0	0	100	2.5	1	68	24.8	128.5	67.5	129	60	69	12.4	5	78.8	2	1	1	1	1	0	1	12.6	0.04	68.1
1	0	0	100	2.05	1	70	23.4	126	77.5	185	60	125	14.4	5.2	77.7	1	1	1	1	1	1	1	11.5	0.03	16.7
1	0	0	100	2.37	1	74	21	138.5	87.5	167	60	107	9.8	4.8	71.5	2	0	1	1	1	1	1	7.3	0.02	36.6
1	0	0	100	4.61	1	72	22.9	111.5	73.5	183	60	123	15.9	5.5	76.7	2	0	0	1	1	1	1	39.2	0.02	6.28
1	0	0	100	4.62	1	70	24.6	153	100	249	60	189	17	5.1	70.1	2	1	1	0	0	1	1	84.1	0.09	36.2
1	0	0	100	4.67	1	66	24	124	73.5	164	60	104	14.2	6	75.7	2	1	0	1	1	1	1	14.9	0.06	29
1	0	0	100	6.86	1	67	23.2	134.5	66	215	60	155	14	5.3	71.6	2	0	1	1	0	1	1	14.5	0.01	19
1	0	0	100	6.87	1	73	20.8	122	65.5	223	60	163	13.8	5.6	72.0	2	1	0	1	0	1	1	8.9	0.04	3.55
1	0	0	100	6.87	1	66	23.9	132.5	82.5	167	60	107	14.5	5	79.9	2	1	0	1	1	1	1	18.3	0.03	21.6
1	0	0	100	6.87	1	75	19.1	127	79	222	60	162	14	5	67.7	2	0	1	1	0	1	1	23.9	0.09	20.2
1	1	0	100	4.89	1	76	26	110	64	226	60	166	13.3	5.5	67.2	2	1	1	1	0	1	1	6.9	0.01	4.96
1	0	0	100	7	2	78	24.9	154	71	179	60	119	14.2	8.4	71.2	1	1	1	0	1	0	1	34.2	0.13	23.3
1	0	0	100	7.01	2	70	21.5	142	88	181	60	121	12.8	5.6	71.6	1	1	1	0	1	1	1	36.3	0.14	42.5
1	0	0	100	5.05	2	69	29.8	146	80	135	60	75	14.2	4.8	72.1	2	1	1	0	1	1	1	4.9	0.16	11.3
1	0	0	100	5.06	2	74	26.3	114	71	190	60	130	12.6	4.9	59.2	1	1	1	1	1	1	1	16.7	0.1	31.8
1	0	0	100	6.98	2	66	27.1	145	80	218	60	158	12.3	5.3	77.5	2	1	0	0	1	1	1	12.7	0.17	70.6
1	0	0	100	6.87	2	72	23.5	143.5	83.5	200	60	140	12.7	5.3	70.6	2	1	1	0	1	1	1	21.1	0.22	45.5
1	0	0	100	6.87	2	71	23.2	117	78	190	60	130	15.1	5.5	74.8	2	1	1	0	0	1	1	33.7	0.51	15.5
1	0	0	100	6.95	2	70	23.7	133.5	65.5	205	60	145	11.9	5.6	80.0	2	1	1	1	1	1	1	26.5	0.2	94
1	0	0	100	6.95	2	67	22.8	116	69	203	60	143	13.4	5.2	76.9	2	1	1	1	0	1	1	10.2	0.4	22.8

1	0	0	100	6.95	2	77	28.4	135	71.5	219	60	159	14.6	5.7	76.1	1	1	1	0	1	1	1	14.9	0.2	72.5
1	0	0	100	6.8	2	71	29.1	152.5	73.5	206	60	146	12.6	5.2	79.4	2	1	1	0	1	1	1	27.1	0.13	39.6
1	0	0	100	4.59	2	66	25.4	158	94	160	60	100	13.1	7.1	73.6	2	1	1	0	1	0	1	10.2	0.11	36.6
1	0	0	100	6.44	2	66	24.9	107	73	221	60	161	14.1	5.5	77.5	2	1	1	1	0	1	1	30.3	0.19	9.45
1	0	0	100	4.69	2	67	26.4	135.5	90	188	60	128	13.4	5.1	73.1	2	1	0	0	0	1	1	22.3	0.7	23.1
1	0	0	100	6.85	2	70	26.3	151	85.5	175	60	115	13.9	5.7	75.3	1	1	1	0	0	1	1	20.6	0.19	23.1
1	0	0	100	6.86	2	65	29.5	115.5	68	153	60	93	13.6	9.4	74.2	2	1	0	1	1	0	1	34.9	0.39	51.6
1	0	0	100	7	2	71	31.3	179	97	185	60	125	13.9	5.2	52.5	1	1	1	0	1	1	1	20.6	0.05	7.69
1	0	0	100	4.58	2	65	26.1	158.5	98.5	156	60	96	15.2	5.6	74.2	2	1	1	0	1	1	1	7.8	0.02	0.69
1	0	0	100	4.58	2	70	26.6	131.5	76	211	60	151	11.6	5.4	60.9	2	1	1	0	1	1	1	5.1	0.03	37
1	0	0	100	6.86	2	70	22.6	162	89	215	60	155	12.5	4.9	75.3	2	1	1	0	1	1	1	12.6	0.05	17.6
1	0	0	100	6.86	2	78	18.4	130.5	78	162	60	102	12.8	4.6	71.2	2	1	1	1	1	1	1	27.3	0.02	24.8
1	0	0	100	6.87	2	68	26.2	114	73.5	199	60	139	12.5	5.3	72.6	2	1	0	1	1	1	1	11.2	0.03	27.4
1	0	0	100	6.87	2	67	23.5	115	70.5	255	60	195	12.9	5.6	62.2	2	1	0	1	0	1	1	5.7	0.01	22.7
1	0	0	100	7.23	2	71	23	120.5	68.5	232	60	172	13.7	5.4	71.1	2	1	1	1	0	1	1	24.3	0.02	18.2
1	0	0	100	5.15	2	65	21.2	91	62	206	60	146	12.6	5.8	74.2	2	1	1	1	1	1	1	9.4	0.07	17.5
1	0	0	100	5.16	2	65	25.6	145.5	93	268	60	208	14.3	6.3	78.0	2	1	1	0	0	1	1	31.7	0.04	5.24
1	0	0	100	6.86	2	65	25.2	110	67.5	284	60	224	12.9	5.6	78.0	2	1	1	1	0	1	1	20.1	0.08	12.1
1	0	0	100	7.02	2	65	23.1	132.5	74	213	60	153	13.6	6.3	82.8	1	1	1	1	1	1	1	10.1	0.03	3.32
1	0	0	100	5.06	2	67	18.6	149	70	172	60	112	12.4	5.5	81.7	2	1	1	0	1	1	1	9.8	0.01	51.5
1	0	0	100	4.63	2	66	23	109	59.5	212	60	152	13.2	5.6	77.5	2	1	1	1	1	1	1	9.4	0.09	21.9
1	0	0	100	4.63	2	65	28.8	108	58.5	240	60	180	13.2	5.8	74.2	2	1	1	1	0	1	1	7.6	0.03	8.75
1	0	0	100	4.63	2	69	27.9	134.5	78.5	266	60	206	13.3	5.7	75.9	2	1	1	1	0	1	1	16	0.06	14.3
1	0	0	100	4.64	2	67	23	144	92.5	186	60	126	13.6	5	73.1	2	1	1	0	1	1	1	33.7	0.01	21.8
1	0	0	100	4.64	2	69	28.1	129	72	231	60	171	13.4	5.5	72.1	2	1	1	1	0	1	1	16.6	0.07	20.3
1	0	0	100	4.64	2	67	24.8	133.5	73.5	235	60	175	13.8	5.2	76.9	1	1	1	1	0	1	1	31.4	0.03	26.4
1	0	0	100	4.64	2	65	19.7	129.5	86	216	60	156	12.6	5.4	74.2	2	1	0	0	1	1	1	20.1	0.01	13.5
1	0	0	100	6.87	2	65	22.4	98.5	70	148	60	88	13.4	4.9	78.0	2	1	1	1	1	1	1	33.8	0.01	5.24
1	0	0	100	6.95	2	68	24.9	125.5	61.5	230	60	170	13.8	6	81.1	2	1	1	1	0	1	1	19.8	0.02	8.34
1	0	0	100	6.98	2	65	28.8	138	75.5	201	60	141	13.2	5.1	82.8	1	1	1	0	1	1	1	24.7	0.02	8.73
1	0	0	100	6.98	2	73	26.5	138.5	75	192	60	132	13.8	6	73.8	2	1	1	1	1	1	1	22.5	0.08	12.8
1	0	0	100	6.8	2	73	24.4	114	68.5	221	60	161	12.8	6.2	59.7	2	1	1	1	0	1	1	23.5	0.08	116
1	0	0	100	4.59	2	69	23.9	113.5	68.5	223	60	163	14.1	5.8	75.9	2	1	0	1	0	1	1	29.2	0.06	15.2
1	0	0	100	4.6	2	70	22.3	176.5	98	231	60	171	13.8	5.1	71.6	2	1	1	0	0	1	1	41	0.04	28.2
1	0	0	100	4.61	2	69	23.3	119	65.5	164	60	104	11.2	5.7	61.4	2	1	1	1	1	1	1	17	0.03	73.2
1	0	0	100	4.61	2	74	22.4	155.5	83	215	60	155	14.1	5.3	73.2	1	1	1	0	1	1	1	49	0.02	35.8
1	0	0	100	4.62	2	66	28.9	131	84	220	60	160	14	5.7	47.8	2	1	1	1	0	1	1	3.5	0.09	34
1	0	0	100	1.44	2	70	20.3	117.5	80	200	60	140	12.6	5.5	80.0	2	1	1	1	1	1	1	20	0.03	41.3
1	0	0	100	4.67	2	68	24.9	150	87	209	60	149	13.7	5.3	72.6	2	1	1	0	1	1	1	45.7	0.03	18.5
1	0	0	100	5.37	2	69	24.6	153.5	92.5	232	60	172	12.3	5.4	72.1	2	1	1	0	0	1	1	18.5	0.04	25.1
1	0	0	100	5.37	2	67	24.7	110.5	67.5	192	60	132	13.3	5.4	73.1	2	1	1	1	1	1	1	10	0.03	9.85
1	0	0	100	5.37	2	69	24.4	102.5	61.5	183	60	123	12.7	5.1	75.9	2	1	1	1	1	1	1	16.5	0.01	15.1
1	0	0	100	5.37	2	67	22.7	95.5	58	163	60	103	12.5	5.7	76.9	2	1	1	1	1	1	1	10.9	0.01	16.8
1	0	0	100	5.06	2	67	24.2	112	69	198	60	138	12.9	6.3	76.9	2	1	1	1	1	1	1	9	0.03	14
1	0	0	100	6.86	2	79	25.8	153	88	244	60	184	13.2	5.6	70.7	1	1	1	0	0	1	1	29.8	0.03	22.7
1	0	0	100	6.87	2	73	27.1	133.5	73.5	238	60	178	13	5.6	73.8	2	1	1	0	0	1	1	14.9	0.03	20.5
1	0	0	100	6.87	2	65	25.6	134.5	76.5	199	60	139	11.6	5.6	78.0	2	1	1	1	0	1	1	9.8	0.02	36.7
1	1	0	100	6.87	2	66	25.7	119	82	197	60	137	14	6.1	73.6	2	1	0	1	1	1	1	16.7	0.12	21.3
1	1	0	100	6.86	2	69	19.1	155.5	86	227	60	167	13.2	5	72.1	2	1	1	0	0	1	1	8.4	0.03	5.95
1	0	0	100	4.64	1	81	24.2	140.5	83	185	60	125	14.8	5.5	45.8	2	0	1	0	1	1	1	7.7	0.14	9.9
1	1	0	100	3.84	1	83	23	122.5	68.5	226	60	166	14.7	5.5	67.2	1	1	0	1	0	1	1	15.7	0.02	49.7

1	0	0	100	4.59	2	81	23.2	115.5	70	176	60	116	13.3	6.3	66.3	2	1	1	0	0	1	1	10.7	0.11	6.2
1	0	0	100	6.8	2	82	18.7	103	63.5	234	60	174	12	6.3	48.6	1	1	1	1	0	1	1	22.8	0.05	9.77
1	1	0	100	7.04	2	82	22.5	156.5	69.5	208	60	148	12.5	5.2	56.0	1	1	1	0	1	1	1	593.9	0.04	42
1	0	0	100	6.88	1	78	21.4	155	82.5	201	60	141	13.1	5.5	42.5	1	0	0	0	1	1	1	141	0.11	117
1	0	0	100	4.63	1	71	29.9	149	88	225	60	165	13.3	5	49.2	1	1	0	0	0	1	1	9.7	0.24	46.9
1	0	0	100	7.06	1	72	20.5	125	78.5	152	60	92	15.4	4.6	72.6	1	1	0	1	1	1	1	6.9	0.01	5.91
1	0	0	100	5.08	1	66	25.8	114.5	63.5	168	60	108	13.8	5.7	72.1	2	1	0	1	1	1	1	13	0.01	0.51
1	0	0	100	1.75	1	65	26.7	163.5	99	205	60	145	16.1	5.1	80.5	2	1	0	0	1	1	1	6.9	0.07	8.93
1	0	0	100	5.05	1	73	21.7	95	61.5	129	60	69	12.7	5.6	68.6	2	0	0	1	1	1	1	2.4	0.01	68.9
1	0	0	100	5.06	2	70	31	119	80	167	60	107	13	5.5	75.3	2	1	0	0	1	1	1	6.9	3.47	14.7
1	0	0	100	6.84	2	71	30.1	163.5	88.5	231	60	171	13.6	6.2	71.1	2	1	1	0	0	1	1	16.5	0.11	0.05
1	0	0	100	6.84	2	73	22.8	141	80	213	60	153	12.9	5.3	70.1	2	1	0	0	1	1	1	76.2	0.12	63.8
1	0	0	100	4.69	2	65	27.9	132.5	77.5	195	60	135	11.7	5.9	74.2	2	1	1	0	0	1	1	12	0.37	28.8
1	0	0	100	5.37	2	72	28.9	112	68.5	237	60	177	12.8	5.9	70.6	1	1	1	1	0	1	1	11.5	0.1	0.75
1	0	0	100	5.04	2	75	28.2	134.5	70.5	189	60	129	12.3	5.8	77.2	1	1	1	0	1	1	1	30.8	0.65	28.6
1	0	0	100	6.86	2	78	23.8	118	68	171	60	111	11.2	5.4	67.7	2	1	1	1	1	1	1	15.8	0.01	14.4
1	0	0	100	6.88	2	68	24.9	112	67	217	60	157	12.7	5	76.4	2	1	1	1	1	1	1	9	0.03	31.5
1	0	0	100	5.15	2	66	22.8	117	74.5	203	60	143	14	5.5	73.6	2	1	1	0	1	1	1	23.3	0.03	22.1
1	0	0	100	5.16	2	71	23.7	150.5	81	217	60	157	12.7	5.2	74.8	2	1	1	0	1	1	1	9.4	0.02	22.1
1	0	0	100	5.16	2	73	27.6	120.5	64	200	60	140	14.7	5.4	51.7	2	1	1	0	1	1	1	33.9	0.05	71.7
1	0	0	100	6.86	2	76	24	144	82.5	222	60	162	13.4	4.9	72.2	1	1	1	0	0	1	1	27.7	0.02	21.4
1	0	0	100	5.05	2	67	21.7	134	68	206	60	146	12.4	4.8	81.7	2	1	1	1	0	1	1	22.9	0.08	8.78
1	0	0	100	5.06	2	70	21.3	126.5	74.5	162	60	102	12.7	5.5	60.9	2	1	0	0	1	1	1	36.6	0.03	22.5
1	0	0	100	5.08	2	68	22.2	125.5	82.5	176	60	116	14.3	6	76.4	2	1	1	0	0	1	1	26.5	0.01	20.1
1	0	0	100	6.99	2	73	27.7	116.5	64	171	60	111	12.9	4.6	78.3	2	1	1	1	1	1	1	12.7	0.08	54.3
1	0	0	100	7	2	66	24.3	139	73	215	60	155	14.4	5.2	88.5	2	1	1	1	1	1	1	13.2	0.02	28.8
1	0	0	100	7	2	65	23.2	141.5	78	142	60	82	11.7	5.2	78.0	2	1	1	0	1	1	1	9.8	0.06	33.5
1	0	0	100	6.95	2	76	19.3	121	72.5	173	60	113	11.8	5.1	68.6	2	1	1	1	1	1	1	12	0.01	49.9
1	0	0	100	6.95	2	65	23.6	132.5	74.5	216	60	156	13.4	6.2	82.8	2	1	1	0	1	1	1	61.1	0.03	3.51
1	0	0	100	6.84	2	68	28	115.5	58.5	205	60	145	12.7	5.4	81.1	1	1	1	1	1	1	1	23.2	0.03	0.05
1	0	0	100	6.97	2	66	30.6	127.5	70.5	227	60	167	12.8	5.5	77.5	2	1	1	1	0	1	1	23.9	0.04	0.05
1	0	0	100	5.37	2	71	31.5	125	74.5	177	60	117	13.8	6	71.1	1	1	1	0	1	1	1	5.6	0.09	72.4
1	0	0	100	5.06	2	75	21.6	120.5	73	210	60	150	12.6	5.4	58.8	1	1	1	1	1	1	1	9	0.02	19.6
1	0	0	100	4.1	2	76	24.2	136.5	74.5	259	60	199	14.7	6	58.4	2	1	1	0	0	1	1	33	0.08	11.6
1	0	0	100	3.32	2	69	29.8	125.5	73.5	191	60	131	13.1	5.2	75.9	2	1	1	0	1	1	1	95.1	0.06	12.3
1	1	0	100	4.58	2	78	25.8	147.5	80.5	233	60	173	13.7	5.4	71.2	2	1	1	0	0	1	1	15.7	0.05	12.6
1	1	0	100	7.02	2	71	24.7	155.5	81.5	202	60	142	13.9	5.8	60.5	2	1	1	0	1	1	1	26.9	0.06	17.5
1	1	0	100	5.07	2	75	27.9	100.5	61	235	60	175	13.1	5.5	69.1	1	1	1	1	0	1	1	14.7	0.06	39.8
1	1	0	100	5.73	2	66	21.3	103.5	60.5	255	60	195	13.7	5.5	73.6	2	1	1	1	0	1	1	11.2	0.09	9.89
1	0	0	100	6.82	1	86	24.4	134	70	215	60	155	14.9	5.6	65.8	2	0	0	1	1	1	1	45.1	0.12	33.7
1	0	0	100	6.87	1	83	23.3	153.5	88	222	60	162	15.2	5.6	67.2	2	1	0	0	0	1	1	56.4	0.01	16.2
1	0	0	100	7.23	2	85	26	165.5	100.5	229	60	169	12.9	5.7	64.4	2	1	1	0	0	1	1	11.1	0.02	50.8
1	0	0	100	4.8	2	83	18.7	112	67.5	204	60	144	10	5	65.3	1	1	1	1	1	1	1	19.3	0.02	41.7
1	0	1	2.3	2.3	1	74	22.2	172.5	92	183	60	123	12.9	5.2	71.5	2	1	1	0	1	1	0	51.9	0.03	99.2
1	1	1	1.8	1.8	1	67	22.8	128	74.5	145	60	85	13.8	4.9	71.6	2	1	0	1	1	1	0	101.9	0.57	241
1	1	1	2.7	2.7	1	65	22	139	85.5	175	60	115	13.5	4.7	80.5	2	1	0	1	1	1	1	8.3	0.01	35.3
1	0	1	3.7	3.7	2	70	22.5	165.5	72.5	200	60	140	13.3	5.1	60.9	2	1	1	0	1	1	1	25.4	0.37	10.9
1	0	1	3.3	3.3	2	75	22.6	123.5	61.5	189	60	129	11	6.2	72.7	2	1	1	1	1	0	1	29.7	0.05	22.1
1	0	1	5.1	5.1	2	74	16.9	104.5	69	171	60	111	14.1	5.5	77.8	2	1	1	1	1	1	1	15.7	0.02	29.5
1	0	1	2.9	2.9	1	75	20.6	141	66	210	60	150	13.8	5.7	71.0	2	1	0	0	0	1	1	16.6	0.08	81.7
1	0	1	6.6	6.6	2	72	27.5	131	70.5	200	60	140	12.6	5.1	60.1	2	1	0	0	0	1	1	19.4	0.02	63.2

1	0	0	100	7.07	1	70	24	140.5	82	235	61	174	15.4	5.5	70.1	2	1	0	0	0	1	1	27.5	0.17	1.14
1	0	0	100	7.08	1	69	21.4	102.5	64	216	61	155	14.5	5.8	83.4	1	1	0	1	1	1	1	106	0.1	0.05
1	0	0	100	4.59	1	70	22.9	124.5	82	196	61	135	11.8	5	77.7	2	1	0	1	1	1	1	54.7	0.1	55.4
1	0	0	100	4.69	1	66	22.2	142.5	92	169	61	108	15.1	6.1	79.9	2	0	0	0	1	0	1	94	0.52	3.76
1	0	0	100	5.04	1	68	22.8	143	76	189	61	128	16.7	5	78.8	2	1	0	0	1	1	1	10.4	0.99	21.8
1	0	0	100	4.58	1	69	22.9	185	107	156	61	95	16.4	5.2	78.3	2	1	0	0	1	1	1	10.6	0.05	14.7
1	0	0	100	4.59	1	71	22.5	103	68	177	61	116	13.5	4.8	73.1	2	1	0	1	1	1	1	8.1	0.04	45.9
1	0	0	100	5.62	1	67	21.2	112	71.5	179	61	118	13.3	5.6	79.4	2	0	0	1	1	1	1	9.7	0.06	65.1
1	0	0	100	5.16	1	76	27.9	156.5	79.5	196	61	135	15.1	5.3	59.2	2	1	0	0	1	1	1	6.7	0.06	37
1	0	0	100	5.16	1	66	26.9	126.5	75	177	61	116	15	5.7	75.7	2	1	0	1	1	1	1	7.9	0.02	38.2
1	0	0	100	6.84	1	70	25.4	118.5	73	216	61	155	14.5	5.1	82.8	2	0	0	0	1	1	1	19.3	0.06	3.27
1	0	0	100	7.04	1	73	23.7	125.5	63	228	61	167	15.4	5.5	76.1	2	0	0	1	0	1	1	19.8	0.01	4.16
1	0	0	100	5.06	1	76	22.7	106	63	171	61	110	12.5	5.7	67.2	2	1	1	1	1	1	1	4	0.02	73.1
1	0	0	100	5.06	1	67	24.5	129	71	145	61	84	14.2	5.1	75.2	2	1	0	1	1	1	0	11.7	0.05	75.3
1	0	0	100	6.98	1	69	23.6	125	80.5	211	61	150	13.9	5.2	62.2	2	1	1	0	1	1	1	35.6	0.03	0.05
1	0	0	100	4.63	1	69	24.6	115.5	72	206	61	145	14.5	5.4	70.6	2	1	1	0	1	1	1	12.1	0.05	20.4
1	0	0	100	4.64	1	69	20.8	131.5	73.5	254	61	193	14.1	6	74.1	2	1	0	1	0	1	1	5.9	0.02	3.61
1	0	0	100	6.95	1	71	22.7	124.5	73	198	61	137	16	5.4	77.2	2	1	1	1	1	1	1	28.6	0.04	14.8
1	0	0	100	6.8	1	65	21	148	91	148	61	87	15	5.2	72.6	2	1	0	0	1	1	1	5.6	0.04	28.9
1	0	0	100	6.44	1	71	28.5	154	78	181	61	120	15.8	5.2	73.1	2	1	0	0	1	1	0	11.9	0.04	27.5
1	0	0	100	4.7	1	74	20.4	113	73.5	158	61	97	9.7	5.3	75.6	1	0	0	1	1	1	1	3.2	0.05	40.3
1	0	0	100	4.7	1	66	21.8	127	68.5	167	61	106	15.5	5.1	79.9	2	1	1	1	1	1	1	15	0.03	10.2
1	0	0	100	5.06	1	65	18	114.5	69.5	182	61	121	14.5	5.8	80.5	2	1	1	1	1	1	1	5.6	0.02	11.8
1	0	0	100	6.85	1	74	25.7	138.5	77	196	61	135	15.3	6.1	60.0	2	1	1	0	1	1	1	604.5	0.01	9.48
1	0	0	100	6.87	1	68	23.9	111	64	251	61	190	15.2	5.7	71.1	2	1	1	1	0	1	1	9.5	0.03	0.74
1	0	0	100	4.59	2	69	26.7	138.5	71.5	213	61	152	14	5.3	53.2	2	1	1	0	1	1	1	17.5	0.24	6.15
1	0	0	100	6.86	2	72	24	129.5	71.5	226	61	165	12.6	7.4	74.3	1	1	1	1	0	0	1	33.1	0.2	14
1	0	0	100	6.84	2	70	24	106.5	62.5	152	61	91	11.5	5.5	75.3	2	1	1	1	1	1	1	10.3	0.11	13.4
1	0	0	100	6.85	2	72	33.4	140.5	70.5	273	61	212	12.7	4.7	60.1	2	1	1	0	0	1	1	6.2	0.37	20.4
1	0	0	100	5.08	2	70	23.7	118	64	159	61	98	12.8	5.6	75.3	2	1	1	1	0	1	1	47.4	3.34	13.9
1	0	0	100	6.98	2	67	23.9	114	63	217	61	156	14.3	7.4	81.7	2	1	1	1	1	0	1	5.8	0.19	0.05
1	0	0	100	4.63	2	72	24.7	109.5	73.5	218	61	157	13.9	5.7	74.3	2	1	1	1	1	1	1	24.4	0.1	36
1	0	0	100	4.63	2	69	23.4	108.5	68.5	217	61	156	12.9	6.4	72.1	2	1	0	1	1	1	1	7	0.22	11.7
1	0	0	100	4.63	2	72	19.7	93.5	51.5	178	61	117	11.3	5.8	70.6	2	1	1	1	1	1	1	13.1	0.61	16.3
1	0	0	100	6.96	2	66	25.5	114.5	77	200	61	139	13.7	5.5	82.3	2	1	0	1	1	1	1	21.5	0.16	5.44
1	0	0	100	4.62	2	68	27.1	141.5	92.5	171	61	110	13.1	5.4	76.4	2	1	1	0	1	1	1	54.1	0.63	28.1
1	0	0	100	4.68	2	71	21.5	100.5	57.5	175	61	114	12.9	5	71.1	1	1	1	1	1	1	1	22.8	1.16	11.3
1	0	0	100	6.86	2	70	24.7	100.5	60	211	61	150	12.9	5.4	80.0	2	1	1	1	1	1	1	21.4	0.13	46.9
1	0	0	100	7.07	2	79	26.4	146.5	71	208	61	147	11.8	6.5	57.2	2	1	0	0	1	0	1	545.9	0.07	0.05
1	0	0	100	7.07	2	66	23.4	134.5	83.5	202	61	141	13.6	5.9	73.6	2	1	1	0	1	1	1	37.4	0.05	45.9
1	0	0	100	7.07	2	69	18.7	105	55	237	61	176	12.1	5.3	53.2	1	1	1	1	0	1	1	16.7	0.03	49.2
1	0	0	100	4.59	2	67	21.3	104.5	72.5	208	61	147	11.6	5.2	62.2	2	1	0	1	1	1	1	11.9	0.05	17.3
1	0	0	100	6.86	2	74	21.7	125	73.5	215	61	154	13.3	5.5	73.2	2	1	1	0	1	1	1	11	0.04	11.9
1	0	0	100	6.87	2	66	20.8	125	91	237	61	176	13.7	5.6	62.7	2	1	0	0	0	1	1	10	0.06	22.2
1	0	0	100	6.84	2	69	22.7	111.5	64	239	61	178	13.9	5.7	61.4	2	1	1	1	0	1	1	8.2	0.01	4.17
1	0	0	100	6.85	2	74	24.4	143.5	75.5	194	61	133	12.5	4.9	69.6	1	1	1	0	1	1	1	51.4	0.08	73.3
1	0	0	100	7.01	2	70	18.2	110.5	62.5	157	61	96	12.5	5.2	75.3	2	1	1	1	1	1	1	17.3	0.01	16.2
1	0	0	100	7.04	2	69	29.4	145.5	78	208	61	147	14	5.8	61.4	1	1	1	0	1	1	1	31.2	0.09	12.1
1	0	0	100	5.06	2	70	21.8	134.5	77	230	61	169	14.1	5.4	71.6	1	1	1	0	0	1	1	22.7	0.06	82.2
1	0	0	100	5.07	2	66	24.4	112	66.5	172	61	111	12.6	5.7	77.5	2	1	0	1	1	1	1	16.2	0.01	16.6
1	0	0	100	6.97	2	70	27.6	136.5	92.5	175	61	114	12	5.1	80.0	1	1	1	0	0	1	1	18	0.03	19

1	0	0	100	4.62	2	72	23.1	117.5	68.5	210	61	149	14.2	5.1	74.3	2	1	1	1	1	1	1	15.4	0.04	73.8
1	0	0	100	4.62	2	72	22.4	97	55	201	61	140	13.4	5.5	70.6	1	1	1	1	0	1	1	15.3	0.08	29.2
1	0	0	100	4.63	2	68	23.4	117.5	66	200	61	139	13	5.3	81.1	2	1	0	1	1	1	1	24	0.04	42.8
1	0	0	100	4.63	2	66	23.6	126	72	229	61	168	13.2	5.4	82.3	2	1	1	1	0	1	1	46.4	0.04	18.9
1	0	0	100	4.63	2	68	27.9	117.5	63	175	61	114	12.7	5.2	72.6	2	1	1	1	1	1	1	12	0.03	28.3
1	0	0	100	6.97	2	67	19.7	92	53.5	220	61	159	11.4	5.5	81.7	2	1	1	1	0	1	1	20.3	0.01	12.2
1	0	0	100	1.86	2	68	27.1	131.5	71	200	61	139	14	5.5	76.4	1	1	1	0	1	1	1	23.3	0.05	54.3
1	0	0	100	6.8	2	70	22.9	161.5	92	208	61	147	14.3	4.8	75.3	2	1	1	0	1	1	1	26.6	0.03	20.6
1	0	0	100	6.84	2	65	25.3	135	88.5	173	61	112	13.7	5.6	78.0	2	1	1	1	1	1	1	13.4	0.04	1.26
1	0	0	100	4.59	2	71	26.2	114.5	63.5	272	61	211	13.4	5.5	74.8	1	1	0	1	0	1	1	17	0.06	24.2
1	0	0	100	4.59	2	73	24	114	76.5	213	61	152	13.1	5.4	70.1	2	1	1	0	1	1	1	12.4	0.06	16.6
1	0	0	100	4.6	2	71	27.3	117.5	73.5	202	61	141	15.2	5.7	79.4	2	1	0	1	1	1	1	18.8	0.03	11.5
1	0	0	100	4.62	2	72	23.1	150.5	75	181	61	120	13.6	5.3	78.9	2	1	1	0	1	1	1	9.4	0.04	30.2
1	0	0	100	4.62	2	65	23.9	123	80.5	203	61	142	13	5.6	82.8	2	1	1	1	1	1	1	56.7	0.02	21.9
1	0	0	100	4.62	2	72	18.7	169.5	102	161	61	100	13.8	5.1	52.1	2	1	1	0	1	1	1	73.2	0.01	112
1	0	0	100	6.79	2	67	25.8	107	66	273	61	212	12.3	5.2	73.1	2	1	1	1	0	1	1	11.2	0.02	17.3
1	0	0	100	5.36	2	69	27.1	138	77	166	61	105	13.7	5.3	72.1	2	1	1	1	1	1	1	20.1	0.03	31.5
1	0	0	100	5.37	2	72	24.2	121.5	70	212	61	151	14	5.4	70.6	1	1	1	0	1	1	1	5.8	0.01	15.8
1	0	0	100	5.37	2	73	17.8	108	59.5	216	61	155	13	6.2	73.8	2	1	1	1	0	1	1	10	0.02	8.9
1	0	0	100	5.06	2	72	27.7	109.5	71.5	183	61	122	14.2	6.3	74.3	1	1	0	1	1	1	1	20.4	0.08	43.5
1	0	0	100	5.06	2	71	20.8	96.5	61	218	61	157	12	5.2	74.8	2	1	1	1	1	1	1	6.6	0.05	23.5
1	0	0	100	6.87	2	74	20.4	106.5	62	177	61	116	12.9	5.7	51.4	2	1	1	0	0	1	1	7.4	0.04	19.2
1	1	0	100	7.07	2	77	21.8	122	71.5	203	61	142	13.1	4.6	76.1	1	1	1	1	1	1	1	20.3	0.09	8.25
1	1	0	100	5.62	1	82	23.2	118.5	67.5	231	61	170	14.2	5.1	50.6	2	0	1	1	0	1	1	20.7	0.12	28.6
1	1	0	100	6.97	1	81	27.8	166.5	96.5	236	61	175	18.4	8.8	72.0	1	1	0	0	0	0	1	61.4	1.28	12.3
1	0	0	100	6.86	2	81	23	143	76.5	192	61	131	11.5	5.1	66.3	2	1	1	0	1	1	1	11.6	0.18	28.4
1	0	0	100	6.45	2	83	25.3	117.5	67.5	189	61	128	11.2	5.2	65.3	2	1	1	0	0	1	1	20.7	0.14	51
1	0	0	100	4.63	2	85	24.3	96	62.5	170	61	109	13.2	5.1	64.4	1	1	0	1	1	1	1	23.8	0.03	89.3
1	0	0	100	7.08	1	66	28.4	116.5	76	249	61	188	14.4	7	79.9	2	1	0	1	0	0	1	14.2	0.12	23.9
1	0	0	100	0.68	1	71	24.4	136	67	141	61	80	11.5	6.2	77.2	2	0	1	1	1	1	1	75.1	1.01	238
1	0	0	100	5.16	1	66	25.5	113	71	198	61	137	14.6	5.2	79.9	2	1	0	1	1	1	1	12.5	0.02	75.6
1	0	0	100	7.03	1	65	24.1	112.5	75.5	220	61	159	13.4	5.6	76.2	2	1	1	1	0	1	1	7.5	0.02	3.61
1	1	0	100	6.85	1	65	20	152.5	84	182	61	121	15.9	6.8	101.3	2	0	0	0	1	0	1	34.1	0.05	2.5
1	1	0	100	2.86	1	76	24.8	136	75.5	153	61	92	14.1	5.2	67.2	1	1	0	0	1	1	1	14.3	0.06	67.2
1	0	0	100	6.86	2	73	24.7	121.5	59	197	61	136	12.2	5.2	70.1	1	1	1	1	0	1	1	9.4	0.47	60.8
1	0	0	100	5.05	2	76	27.2	122	69.5	182	61	121	12.2	6.1	76.7	2	1	1	0	1	1	1	20.4	0.38	70.1
1	0	0	100	5.08	2	69	31.6	151.5	86	304	61	243	13.9	5.7	80.5	2	1	1	0	0	1	1	32.3	0.1	32.3
1	0	0	100	6.87	2	79	23.7	125	83	218	61	157	13	5.6	70.7	2	1	1	1	1	1	1	52.1	0.26	26.3
1	0	0	100	7	2	68	18.9	174	86.5	163	61	102	13.7	4.9	81.1	1	1	1	0	1	1	1	135.7	0.04	82.6
1	0	0	100	7.06	2	78	24.5	210.5	97.5	233	61	172	14.7	5.7	67.7	1	1	1	0	0	1	1	32.1	0.06	114
1	0	0	100	4.58	2	76	24.9	132.5	81.5	178	61	117	12.8	5.4	72.2	2	1	1	0	1	1	1	4.8	0.07	12.3
1	0	0	100	6.86	2	70	25.5	142	75.5	171	61	110	13.6	5.1	71.6	2	1	1	0	0	1	1	15.6	0.03	25.6
1	0	0	100	6.87	2	73	24.6	130.5	78.5	209	61	148	12.1	5.7	70.1	2	1	1	0	1	1	1	12.3	0.01	25.7
1	0	0	100	5.16	2	79	21.6	130	74.5	174	61	113	13.1	5.2	75.1	1	1	1	1	1	1	1	55.9	0.01	41.1
1	0	0	100	5.16	2	67	29	108	71.5	165	61	104	12.4	5.5	81.7	2	1	1	1	1	1	1	16.9	0.06	17.3
1	0	0	100	5.08	2	66	22.7	156.5	92	192	61	131	13.7	5.4	73.6	2	1	1	0	1	1	1	10.5	0.03	53.8
1	0	0	100	5.08	2	66	22.8	118	72.5	245	61	184	13	5.5	82.3	2	1	1	1	0	1	1	11.3	0.02	27.1
1	0	0	100	6.99	2	73	24.8	110	65.5	173	61	112	13.3	5	78.3	1	1	1	1	1	1	1	17	0.03	21.4
1	0	0	100	4.63	2	66	25.6	151.5	85	218	61	157	12.2	5.4	77.5	2	1	1	0	1	1	1	12.4	0.06	21.2
1	0	0	100	4.64	2	66	24.3	125	70	205	61	144	12.5	5.6	82.3	2	1	1	1	1	1	1	9.5	0.04	20
1	0	0	100	4.61	2	70	27.2	134.5	87.5	246	61	185	12.9	5.9	71.6	2	1	1	1	0	1	1	12.1	0.03	6.32

1	0	0	100	4.68	2	70	24.2	138	82	258	61	197	14.1	5.9	71.6	1	1	1	0	0	1	1	21.6	0.06	29.5
1	0	0	100	4.69	2	65	31.1	143.5	82.5	199	61	138	14.2	5.4	78.0	2	1	1	0	1	1	1	21.3	0.04	23
1	0	0	100	4.7	2	67	25.5	151	75	200	61	139	15.2	4.8	81.7	2	1	0	0	0	1	1	12.3	0.09	24.2
1	0	0	100	6.65	2	76	22.1	132.5	80	210	61	149	13	6.2	72.2	2	1	1	1	1	1	1	18.3	0.02	0.05
1	0	0	100	6.97	2	68	27.4	156.5	97	183	61	122	13.4	5	72.6	2	1	0	0	1	1	1	18.1	0.08	20.6
1	0	0	100	5.37	2	68	19.5	129.5	81.5	198	61	137	12	5.9	81.1	2	1	1	1	1	1	1	14.7	0.02	34.6
1	0	0	100	6.85	2	76	25.5	154.5	75.5	207	61	146	13.3	5.7	68.6	1	1	1	0	0	1	1	12.1	0.03	19.4
1	0	0	100	6.86	2	69	18.8	96	60.5	208	61	147	12.6	5.2	80.5	2	1	1	1	1	1	1	31.9	0.02	10.5
1	0	0	100	6.87	2	79	22.6	139	78.5	224	61	163	10.9	5.3	70.7	1	1	1	1	0	1	1	151.6	0.01	61.9
1	0	0	100	5.04	2	76	25.7	108.5	77.5	282	61	221	13	5.6	76.7	2	1	1	0	0	1	1	23.3	0.04	20.2
1	1	0	100	7.08	2	73	28.7	164	94.5	239	61	178	14.5	5.2	40.6	2	1	1	0	0	1	1	31.8	0.04	9.33
1	1	0	100	6.87	2	65	24.2	142	85	226	61	165	14.3	5.1	74.2	2	1	0	0	0	1	1	64.4	0.08	31.1
1	1	0	100	7.01	2	75	17.1	137	77.5	159	61	98	12.1	4.7	69.1	1	1	1	0	1	1	1	463.8	0.03	90.1
1	1	0	100	6.95	2	76	27.9	150	88.5	195	61	134	12.3	5.8	50.7	1	1	1	0	1	1	0	32.6	0.03	49.4
1	0	0	100	5.37	1	81	19.6	126.5	77.5	146	61	85	12.2	5.5	68.1	1	1	0	1	1	1	1	9.4	0.04	98.2
1	0	0	100	6.98	2	80	22.9	123	70	200	61	139	12.7	5.6	70.2	1	1	1	0	1	1	1	35.9	0.12	0.05
1	0	0	100	5.36	2	84	23.3	145.5	76	195	61	134	12.5	5.5	68.3	1	1	1	0	1	1	1	57.8	0.13	61.8
1	0	0	100	4.63	2	80	24.3	123	67.5	185	61	124	11.8	5.9	49.3	1	1	1	0	1	1	1	37.1	0.03	58.7
1	0	1	1.2	1.2	1	71	21.1	168.5	76	134	61	73	14.5	5.8	73.1	1	0	0	0	1	1	1	22.9	0.77	72.1
1	0	1	1.8	1.8	1	69	25.2	146.5	83	186	61	125	14.4	5.7	62.2	2	0	0	0	1	1	1	8.7	0.08	18.1
1	0	1	2.5	2.5	1	77	19.7	143.5	74	169	61	108	16.1	5.7	66.7	2	0	0	0	1	1	0	21.8	0.07	24.5
1	0	1	1.5	1.5	2	67	28.8	125	87	149	61	88	14.3	5.3	73.1	2	1	0	1	1	1	1	19.4	0.04	32
1	1	1	4.2	4.2	2	70	22.3	109.5	66	201	61	140	13.6	5.4	80.0	2	1	1	1	1	1	1	14.6	0.03	24.9
1	1	1	0.4	0.4	1	76	24.3	155.5	81.5	171	61	110	15.2	5.9	74.5	2	0	0	0	1	1	1	20.8	0.14	86.3
1	0	0	100	4.6	1	69	21.7	147.5	84	199	62	137	15	11	74.1	2	1	1	0	1	0	1	1191.3	0.11	2.13
1	0	0	100	6.87	1	74	28.2	164	99	236	62	174	14.5	5.1	71.5	2	1	0	0	0	1	1	15.7	0.17	52.9
1	0	0	100	5.04	1	74	22.4	135	74	176	62	114	17.2	6.3	71.5	2	1	0	0	1	1	1	102.3	0.19	76.4
1	0	0	100	7	1	68	26.8	178.5	89.5	234	62	172	14.7	5.1	78.8	2	1	1	0	0	1	1	23.5	0.03	17.6
1	0	0	100	7.07	1	66	21.2	136	73.5	188	62	126	15.7	5.1	72.1	2	1	0	1	1	1	1	15	0.01	17
1	0	0	100	7.07	1	71	26.8	118	67.5	194	62	132	14.5	5.7	77.2	2	1	1	1	1	1	1	4.6	0.02	0.05
1	0	0	100	4.58	1	77	22.7	114	77.5	182	62	120	14.8	5.7	58.8	2	0	1	1	1	1	1	5.1	0.02	17.8
1	0	0	100	7.21	1	67	24.8	99	65	202	62	140	14.3	5.4	71.6	2	1	0	1	1	1	1	12.5	0.03	62.6
1	0	0	100	6.87	1	66	26.9	106	71.5	194	62	132	15.2	5.7	79.9	2	1	0	1	1	1	1	5.2	0.03	26.3
1	0	0	100	6.88	1	74	23.7	114	71.5	247	62	185	16.3	5.5	71.5	2	1	0	1	0	1	1	8.2	0.05	41.2
1	0	0	100	5.15	1	74	20.8	184.5	110.5	211	62	149	15	5.9	71.5	2	1	0	0	1	1	1	19.1	0.01	18.3
1	0	0	100	6.85	1	66	25.3	110.5	71	172	62	110	14.3	5.3	75.7	2	1	0	1	1	1	1	6	0.02	15.9
1	0	0	100	6.85	1	72	23.9	163.5	96.5	182	62	120	14.9	5.3	76.7	2	1	0	0	1	1	1	14.3	0.02	62.9
1	0	0	100	7.21	1	69	19.8	106	65.5	208	62	146	14.4	5	62.2	2	1	1	1	1	1	1	15.3	0.02	30.2
1	0	0	100	7.04	1	69	26	146.5	86	179	62	117	14.8	5.3	78.3	1	1	1	0	1	1	1	7.5	0.01	8.26
1	0	0	100	5.08	1	75	23.1	130	71	197	62	135	15.4	5.2	67.7	2	1	0	1	1	1	1	8.6	0.03	20.1
1	0	0	100	6.98	1	69	20.9	126	80	164	62	102	14.3	4.9	74.1	2	1	0	1	1	1	1	2	0.03	0.05
1	0	0	100	6.98	1	73	23.1	131	75.5	194	62	132	14.7	5	68.6	2	1	0	1	1	1	1	11.2	0.03	12.4
1	0	0	100	4.63	1	65	25.9	154	93.5	190	62	128	17.9	5.6	63.9	2	0	0	0	1	0	1	20.3	0.03	17.3
1	0	0	100	4.64	1	73	21.7	126.5	78.5	196	62	134	14.1	5.9	76.1	2	1	0	1	1	1	1	4.2	0.05	14.5
1	0	0	100	4.62	1	72	23.1	135.5	85	237	62	175	13.6	5.2	72.6	1	1	1	1	0	1	1	7.7	0.04	12.4
1	0	0	100	4.62	1	69	23.4	114.5	66	200	62	138	14.9	6.1	78.3	2	1	0	1	1	1	1	6.2	0.03	12.7
1	0	0	100	4.67	1	68	21.3	109	68.5	167	62	105	14.7	5.5	78.8	2	1	0	1	1	1	1	21.7	0.01	13.6
1	0	0	100	5.36	1	65	17.2	110	62.5	150	62	88	13.9	5.1	80.5	2	1	1	1	1	1	1	14.1	0.01	64.5
1	1	0	100	4.11	1	65	22.5	103.5	65.5	170	62	108	11.4	5.2	80.5	2	1	0	1	1	1	1	15.9	0.14	69.7
1	1	0	100	4.35	1	69	26.7	120.5	75.5	218	62	156	15.1	5.3	70.6	2	1	0	0	1	1	1	110.5	0.06	47.4
1	1	0	100	4.61	1	75	20.9	153.5	71	249	62	187	14.5	4.7	71.0	2	1	0	0	0	1	1	6.7	0.08	30.9

1	1	0	100	6.86	1	79	27.2	140	79	217	62	155	14.2	5.1	69.1	2	1	0	0	1	1	1	367.9	0.04	5.32
1	0	0	100	7.07	2	67	24.3	126.5	74.5	203	62	141	13.5	5	73.1	2	1	1	1	1	1	1	12.1	0.3	28.1
1	0	0	100	6.86	2	68	21.8	153	82.5	207	62	145	13.6	6.3	72.6	2	1	1	0	1	1	1	18.7	0.16	14.7
1	0	0	100	6.87	2	70	26.5	116	72.5	225	62	163	13.3	5.9	60.9	2	1	1	1	0	1	1	12.6	0.1	21.2
1	0	0	100	6.84	2	72	23.2	106.5	59.5	192	62	130	12.8	4.9	70.6	2	1	1	1	0	1	1	10.7	0.11	30.7
1	0	0	100	7.02	2	66	22.9	157	88.5	211	62	149	12.8	4.6	73.6	2	1	1	0	1	1	1	18	0.39	53.4
1	0	0	100	7.04	2	65	19.9	135.5	68	187	62	125	11.1	5.2	63.1	2	1	1	1	0	1	1	6.1	0.17	1.35
1	0	0	100	5.05	2	72	21.8	138	79	171	62	109	13.8	5.7	74.3	2	1	1	0	0	1	1	39.8	0.43	5.95
1	0	0	100	6.97	2	65	28.8	116.5	62.5	227	62	165	12.6	5.4	78.0	2	1	1	1	0	1	1	19.4	0.12	0.25
1	0	0	100	6.8	2	69	19.6	131.5	74.5	239	62	177	14.2	6.3	41.7	2	1	1	1	0	1	1	30.9	0.13	35.6
1	0	0	100	6.8	2	70	26	128	83.5	183	62	121	13	6	60.9	2	1	1	0	0	1	1	7.2	0.16	2.34
1	0	0	100	4.6	2	77	25.7	140	103	308	62	246	12.6	5.5	68.2	2	1	1	0	0	1	1	28.4	0.23	29.3
1	0	0	100	4.69	2	66	21.1	130	75	198	62	136	14.4	6.1	77.5	2	1	0	1	1	1	1	6.5	0.52	40.6
1	0	0	100	4.7	2	74	24.4	128.5	78	218	62	156	13.7	5.8	69.6	1	1	1	1	1	1	1	6	0.12	18.1
1	0	0	100	7	2	76	22.2	149.5	77	210	62	148	12.4	5.1	72.2	2	1	1	0	1	1	1	19.9	0.03	41.2
1	0	0	100	7.06	2	65	21.8	136.5	80	230	62	168	12.5	5.1	74.2	2	1	1	1	0	1	1	36.9	0.03	24.4
1	0	0	100	7.07	2	69	19.3	140	75.5	209	62	147	11	5.3	95.3	2	1	1	0	1	1	1	300	0.01	0.05
1	0	0	100	7.07	2	67	25.5	122	72	221	62	159	12.4	5.2	81.7	2	1	1	1	0	1	1	98.4	0.02	8.36
1	0	0	100	7.08	2	65	26.4	125.5	75	202	62	140	14.8	5.3	63.1	2	1	1	1	0	1	1	26.8	0.03	0.05
1	0	0	100	4.58	2	68	25.9	111.5	66.5	188	62	126	13.2	5.5	72.6	2	1	0	1	1	1	1	19.6	0.07	15
1	0	0	100	6.88	2	66	28.1	130.5	70	212	62	150	12.3	5.4	82.3	2	1	1	1	1	1	1	11	0.06	20
1	0	0	100	6.88	2	65	18.8	111.5	65.5	245	62	183	13.6	5.2	74.2	2	1	1	1	0	1	1	4.6	0.07	50
1	0	0	100	6.84	2	70	22.6	113	69.5	221	62	159	12.3	5.6	60.9	2	1	0	1	0	1	1	5.6	0.03	11.7
1	0	0	100	0.76	2	72	21.1	165	85	240	62	178	14.2	5.2	74.3	2	1	1	0	0	1	1	54.3	0.05	45.4
1	0	0	100	6.86	2	79	20.7	129	68	250	62	188	12.9	5.5	67.2	2	1	1	1	0	1	1	12	0.04	29.3
1	0	0	100	7.02	2	74	27.2	130.5	71.5	178	62	116	12.4	5	73.2	1	1	0	0	1	1	1	18.3	0.02	39.4
1	0	0	100	7.03	2	69	29.5	111.5	71.5	192	62	130	12.9	5.4	86.7	1	1	1	1	1	1	1	4.8	0.08	4.88
1	0	0	100	5.05	2	71	26.6	131	75	168	62	106	14	5.3	74.8	1	1	1	0	1	1	1	3.7	0.02	38.9
1	0	0	100	5.06	2	65	30.1	116.5	71.5	217	62	155	14.7	5.9	78.0	2	1	0	1	1	1	1	25.2	0.06	11.3
1	0	0	100	5.06	2	72	23.4	123	73.5	186	62	124	13	6	74.3	1	1	1	1	1	1	1	33.7	0.06	21
1	0	0	100	5.07	2	70	23	145	82	207	62	145	10.2	5.6	75.3	1	1	1	0	1	1	1	28.6	0.02	46.4
1	0	0	100	5.07	2	65	20.8	102.5	61.5	179	62	117	12.9	5.9	82.8	2	1	1	1	0	1	1	11.1	0.01	34.3
1	0	0	100	5.08	2	70	24.3	128	70.5	217	62	155	11.7	5.7	80.0	2	1	1	0	1	1	1	8.3	0.06	65.2
1	0	0	100	5.08	2	72	28.2	133	82.5	209	62	147	14.1	6.2	70.6	1	1	1	0	1	1	1	13.9	0.09	53.2
1	0	0	100	6.97	2	77	24	130.5	67	218	62	156	12.9	5.2	71.7	1	1	1	1	1	1	1	15.2	0.04	1.73
1	0	0	100	6.98	2	73	23.8	132	78.5	233	62	171	12.9	5.3	70.1	2	1	1	0	0	1	1	19.9	0.06	1.12
1	0	0	100	7	2	66	23.6	137	84.5	200	62	138	13.1	4.9	82.3	2	1	0	1	1	1	1	6.8	0.05	36.2
1	0	0	100	7	2	65	28.4	121	80	159	62	97	12	5.2	74.2	2	1	1	1	1	1	1	3.8	0.03	39
1	0	0	100	4.63	2	72	22.7	109.5	65	230	62	168	12.9	5.3	70.6	2	1	1	0	0	1	1	24.2	0.04	28.5
1	0	0	100	4.63	2	71	23	158	83	215	62	153	13.9	6	79.4	2	1	1	0	1	1	1	10.5	0.01	12.2
1	0	0	100	4.63	2	68	25	136	75.5	225	62	163	14	5.4	72.6	2	1	0	1	0	1	1	39.2	0.03	6.42
1	0	0	100	4.64	2	65	25.1	111.5	69.5	210	62	148	13.7	5.6	78.0	2	1	1	1	1	1	1	7.5	0.02	14.1
1	0	0	100	6.95	2	75	23.1	134	64.5	181	62	119	13.3	6.7	69.1	1	1	1	1	1	0	1	41.6	0.02	32.1
1	0	0	100	6.98	2	66	28.5	132.5	76.5	182	62	120	12.4	5	77.5	2	1	1	1	1	1	1	23.6	0.09	57.9
1	0	0	100	6.98	2	71	25.6	135.5	76	159	62	97	13.8	5.7	74.8	2	1	1	0	1	1	1	106	0.01	78.2
1	0	0	100	6.83	2	77	19	143	79	215	62	153	7.7	5.5	71.7	2	1	1	0	1	1	1	17.3	0.01	20.1
1	0	0	100	6.84	2	67	23.1	114.5	61.5	267	62	205	14.2	5.4	76.9	2	1	1	1	0	1	1	11.7	0.01	0.05
1	0	0	100	4.59	2	65	19.2	113	64	178	62	116	13.3	5.9	74.2	2	1	1	1	1	1	1	15.5	0.01	50.1
1	0	0	100	4.6	2	72	21.4	132	65.5	186	62	124	13.6	5.2	45.9	2	1	1	1	1	1	1	19	0.01	46.4
1	0	0	100	4.61	2	72	24	119	75.5	233	62	171	13.6	5.5	70.6	2	1	1	1	0	1	1	6.3	0.08	7.26
1	0	0	100	2.8	2	67	21.6	117	66.5	207	62	145	13.6	5.6	76.9	2	1	1	0	1	1	1	6.9	0.02	14.1

1	0	0	100	6.79	2	71	18.6	105	53.5	154	62	92	12.3	5.2	60.5	2	1	1	1	1	1	1	18.5	0.01	50
1	0	0	100	6.44	2	70	22.1	115.5	66.5	186	62	124	12.5	5.6	71.6	2	1	1	0	1	1	1	5.3	0.06	8.4
1	0	0	100	4.67	2	70	35.8	196	94	179	62	117	14.3	5.9	75.3	1	1	1	0	1	0	1	65.3	0.08	8.12
1	0	0	100	4.67	2	68	23.7	176.5	87	190	62	128	13.3	7.2	81.1	1	1	1	0	1	0	1	103.3	0.05	35.2
1	0	0	100	4.69	2	71	24.4	135	86	191	62	129	13.4	5.7	85.5	2	1	0	1	1	1	1	69.1	0.02	90.9
1	0	0	100	4.69	2	73	24	139	86.5	173	62	111	13.7	4.4	70.1	1	1	1	1	1	1	1	81.7	0.05	15.2
1	0	0	100	5.06	2	75	22.3	134	70	213	62	151	13.8	5.3	69.1	2	1	1	0	1	1	1	9.1	0.03	27.6
1	0	0	100	5.07	2	70	20.5	114	61.5	205	62	143	12.8	5.5	75.3	2	1	1	0	0	1	1	38.7	0.01	28.1
1	0	0	100	5.07	2	69	25.8	109.5	63.5	221	62	159	12.1	5.5	75.9	2	1	1	1	0	1	1	12.5	0.03	35.1
1	0	0	100	6.86	2	69	21.6	158	88	223	62	161	13.6	4.8	75.9	1	1	0	0	0	1	1	42.4	0.04	6.71
1	0	0	100	6.87	2	72	24.6	147.5	82.5	226	62	164	14.1	5.3	78.9	2	1	1	0	0	1	1	39.8	0.06	14.7
1	0	0	100	5.04	2	68	24.5	109.5	66	221	62	159	11.9	5.4	72.6	2	1	1	0	0	1	1	27.1	0.01	17.9
1	0	0	100	5.04	2	69	23.8	110	64.5	181	62	119	11.2	5.1	80.5	2	1	1	1	1	1	1	5.7	0.02	22.5
1	1	0	100	6.84	2	79	20.7	129.5	73	214	62	152	14.4	5.2	43.7	1	1	1	1	1	1	1	36.1	0.15	39.3
1	1	0	100	6.87	2	75	22.7	132	76.5	208	62	146	13.2	5.3	72.7	1	1	0	1	1	1	1	5.8	0.12	32.8
1	1	0	100	6.44	2	74	20.5	132	70.5	187	62	125	12.2	5.3	59.2	2	1	1	1	1	1	1	21.3	0.05	15
1	0	0	100	4.58	1	80	24.4	107	76	188	62	126	15.3	5.9	57.5	2	1	0	1	1	1	1	23.9	0.03	43.1
1	0	0	100	4.62	1	83	17.3	119	76	135	62	73	13.5	5.4	71.0	2	1	0	0	1	1	1	32.8	0.01	65.9
1	1	0	100	7.05	1	80	21.6	158.5	82	165	62	103	14.9	4.7	68.6	1	1	0	0	1	1	1	78.2	0.02	81.5
1	0	0	100	4.6	2	81	20.6	134.5	71	173	62	111	11.8	5.7	66.3	1	1	1	1	1	1	1	12.8	0.01	14.6
1	0	0	100	6.95	1	69	24.6	112	67.5	219	62	157	14.6	5.4	74.1	2	1	1	1	1	1	0	13.1	0.38	57.2
1	0	0	100	6.95	1	73	27	126.5	67	197	62	135	13.8	5.8	68.6	2	1	1	1	0	1	1	21.2	0.24	20.9
1	0	0	100	6.86	1	73	22.4	136.5	71	173	62	111	14.3	7.8	76.1	2	1	0	1	1	0	1	11.3	0.04	40.7
1	0	0	100	5.05	1	71	33.3	142	88	194	62	132	15.1	6.5	69.6	2	1	1	0	1	0	1	28.5	0.01	28.7
1	0	0	100	4.64	1	78	24.8	133	76.5	174	62	112	14.8	5	78.3	2	1	0	1	1	1	1	22.7	0.03	75.7
1	0	0	100	7.08	2	79	22.8	116.5	64	193	62	131	11.7	7.2	70.7	1	1	1	1	1	0	1	100.8	1.87	104
1	0	0	100	5.08	2	71	20.9	135	72.5	218	62	156	12.9	6.1	60.5	1	1	1	1	1	1	1	31.8	0.22	11.3
1	0	0	100	5.04	2	65	26.3	125.5	73.5	152	62	90	12	5.5	78.0	2	1	1	1	1	1	1	18.8	1.64	16.9
1	0	0	100	7.07	2	67	23.6	158.5	82	236	62	174	14.1	5.3	81.7	2	1	1	0	0	1	1	69.4	0.03	22.7
1	0	0	100	6.87	2	77	26	135	66	235	62	173	12.2	6.3	68.2	1	1	1	0	0	1	1	22.2	0.03	172
1	0	0	100	6.87	2	78	17.8	125	60	196	62	134	11.6	5.2	75.6	1	1	1	1	1	1	1	62.2	0.06	21.6
1	0	0	100	6.84	2	74	23.5	150.5	71	207	62	145	12.6	4.6	69.6	2	1	1	0	1	1	1	13.3	0.03	95.2
1	0	0	100	6.84	2	75	25.5	140.5	83	242	62	180	12.6	5.1	29.9	1	1	1	0	0	1	1	84.5	0.07	11.1
1	0	0	100	6.86	2	76	25.1	180.5	99.5	181	62	119	12.7	5.3	58.4	1	1	1	0	1	1	1	18.4	0.02	57.2
1	0	0	100	5.08	2	69	25.7	141.5	74.5	205	62	143	11.4	5.9	75.9	2	1	1	0	0	1	1	230.6	0.03	11.5
1	0	0	100	6.97	2	70	23.9	115	71.5	179	62	117	11	5.4	80.0	1	1	0	1	1	1	1	10.9	0.02	14.5
1	0	0	100	4.64	2	74	27.6	130	74	218	62	156	15.2	5.3	59.2	2	1	1	0	1	1	1	201.6	0.05	106
1	0	0	100	4.64	2	77	23.6	163	93	189	62	127	12.4	4.9	76.1	1	1	1	0	1	1	1	63.7	0.03	40
1	0	0	100	6.87	2	75	24.2	147	86.5	222	62	160	12	5.8	72.7	2	1	1	0	0	1	1	48.8	0.05	22.6
1	0	0	100	6.95	2	74	24.6	157	100.5	209	62	147	13.5	5.2	73.2	1	1	1	0	1	1	1	15.6	0.04	25.7
1	0	0	100	6.95	2	71	25.5	173.5	96	196	62	134	13.7	5.3	71.1	2	1	1	0	1	1	1	132.6	0.03	42.4
1	0	0	100	6.95	2	69	27.2	138	80	231	62	169	14	5.6	80.5	2	1	0	1	0	1	1	9.1	0.07	11.2
1	0	0	100	6.96	2	69	24	111.5	67	193	62	131	13.8	5.3	80.5	1	1	0	0	1	1	1	24.5	0.01	36
1	0	0	100	6.98	2	70	19.1	139	86	201	62	139	13.9	5.1	75.3	2	1	1	1	1	1	1	633	0.05	41.1
1	0	0	100	4.6	2	73	22.1	144.5	73.5	198	62	136	11.6	6.4	78.3	2	1	1	0	1	1	1	15.7	0.03	30.8
1	0	0	100	5.37	2	69	28.2	132	81.5	256	62	194	12.9	5.7	72.1	2	1	1	0	0	1	1	24.9	0.03	5.51
1	0	0	100	6.87	2	70	26.6	108	61.5	191	62	129	13.4	5.1	71.6	2	1	1	1	1	1	1	8.1	0.05	9.19
1	0	0	100	6.87	2	79	20.8	124.5	59.5	202	62	140	12	5.2	57.2	1	1	1	1	1	1	1	66.6	0.06	44.1
1	1	0	100	4.63	2	75	27.8	128	67.5	199	62	137	14.5	6.6	58.8	2	1	1	1	1	0	1	119	0.13	110
1	1	0	100	4.63	2	77	26.2	125	86.5	146	62	84	12.2	5.1	76.1	2	1	1	0	1	1	1	22.4	0.07	22.2
1	1	0	100	6.8	2	79	17.5	128	72	200	62	138	12.4	5.4	70.7	1	1	1	0	1	1	1	15.9	0.04	23.4

1	1	0	100	4.25	1	88	19.5	149.5	72	137	62	75	14.9	5.5	64.8	2	0	0	0	1	1	1	174.9	0.02	190
1	0	0	100	4.62	2	85	24.4	138	80.5	285	62	223	14.2	5.3	54.8	1	1	1	0	0	1	1	14.3	0.02	34.3
1	1	0	100	7.08	2	84	33.6	129	74	195	62	133	13.1	5.7	64.9	1	1	1	0	1	1	1	21.9	0.05	49.9
1	1	0	100	6.44	2	82	21.5	132	75.5	149	62	87	14.6	5.5	69.2	2	1	1	1	1	1	1	6.9	0.02	3.98
1	0	1	6.5	6.5	1	69	23.5	188.5	91.5	218	62	156	15.1	4.5	78.3	2	1	0	0	1	1	1	71	0.05	82.8
1	0	1	2.7	2.7	1	70	30.4	128	84.5	190	62	128	13.9	5.8	77.7	2	1	1	0	1	1	1	10.6	0.06	24.4
1	1	1	4.3	4.3	2	74	25.8	140	78	192	62	130	13.5	5.8	73.2	2	1	1	0	1	1	1	50.7	0.1	12
1	1	1	1.8	1.8	2	77	21.3	149.5	76.5	182	62	120	12.8	5.4	68.2	1	1	1	0	1	1	1	43.5	0.04	75.4
1	0	1	3.8	3.8	1	74	21.3	144.5	87.5	213	62	151	14.2	4.8	60.0	2	1	1	0	1	1	1	12.5	0.09	29.7
1	1	1	3.7	3.7	2	77	20.3	110	61.5	259	62	197	13.3	5.1	71.7	1	1	1	1	0	1	1	24.1	0.02	5.01
1	0	0	100	6.87	1	70	25.4	144	81	230	63	167	13.2	5.1	70.1	1	1	1	0	0	1	0	215.5	1.39	100
1	0	0	100	6.84	1	72	25.3	109	67.5	182	63	119	15.2	5.4	72.6	2	1	0	1	1	1	1	7.8	0.1	18.2
1	0	0	100	6.86	1	66	21.2	102	64	183	63	120	12.5	4.9	72.1	2	1	0	0	1	1	1	15.6	0.21	39.5
1	0	0	100	4.62	1	70	25.6	123	72	164	63	101	14.4	6.5	77.7	2	1	0	1	1	0	1	457.5	0.14	7.07
1	0	0	100	6.98	1	70	30.4	146	85	180	63	117	15.8	6	77.7	1	1	0	0	1	0	1	36.9	0.27	10.9
1	0	0	100	4.59	1	74	22.5	151.5	89	217	63	154	16.2	5.1	68.2	2	1	1	0	1	1	1	10.8	0.42	16.5
1	0	0	100	6.86	1	66	19.6	210.5	85.5	178	63	115	15.1	5.2	79.9	2	0	0	0	1	1	1	36.7	0.12	67.5
1	0	0	100	6.87	1	70	26.7	131	76.5	185	63	122	15.3	8.1	70.1	2	1	0	0	1	0	1	374.3	0.1	10.9
1	0	0	100	7	1	67	26	139	93	191	63	128	14.1	5.6	63.0	2	0	0	0	1	1	1	20.1	0.04	33.2
1	0	0	100	7.07	1	68	25.6	166.5	98.5	210	63	147	15	5.3	62.6	2	1	0	0	1	1	1	11.6	0.08	12.8
1	0	0	100	7.08	1	70	23.9	113.5	70	204	63	141	14.9	5	73.6	1	1	1	1	1	1	1	21.8	0.01	9.27
1	0	0	100	4.58	1	65	22.5	124	88.5	194	63	131	15.2	5.7	76.2	2	1	0	1	1	1	1	13.1	0.08	9.33
1	0	0	100	5.16	1	72	23.6	102.5	73	202	63	139	17.3	6.2	44.3	2	0	1	1	1	1	0	6.9	0.07	55
1	0	0	100	6.84	1	76	29.8	106	67.5	178	63	115	13.9	6.1	59.2	2	1	1	1	0	1	1	14.2	0.02	13.8
1	0	0	100	6.84	1	70	23.5	127	78	200	63	137	15.3	5.4	70.1	1	1	1	1	1	1	1	14.1	0.01	30
1	0	0	100	6.84	1	76	27.1	161	77.5	217	63	154	15.1	5.6	59.2	2	1	0	0	1	1	1	10.1	0.05	45.6
1	0	0	100	6.85	1	73	22.5	125.5	76	215	63	152	13.3	5.2	76.1	2	1	1	1	1	1	1	11.6	0.03	32.9
1	0	0	100	7	1	65	23.6	94.5	62	185	63	122	14.2	5.4	72.6	2	1	0	1	1	1	1	8.4	0.04	9.43
1	0	0	100	7	1	73	21.2	151	91	137	63	74	15	5.7	68.6	2	1	0	0	1	1	1	7.9	0.03	8.19
1	0	0	100	7	1	67	23.6	103.5	60	206	63	143	14.9	5.3	71.6	2	1	0	1	1	1	1	6.5	0.02	13.4
1	0	0	100	7	1	71	19.9	127.5	75.5	159	63	96	12	5.1	88.6	2	0	0	1	1	1	1	29.3	0.09	13.2
1	0	0	100	4.63	1	76	22.9	167.5	85	189	63	126	14.6	4.7	67.2	2	1	0	0	1	1	1	4.6	0.02	22.2
1	0	0	100	6.87	1	72	20.8	135	76.5	160	63	97	13.4	5.1	76.7	2	1	0	1	1	1	1	4.1	0.01	24.6
1	0	0	100	3.42	1	71	25.4	157	86	218	63	155	16.7	6.4	73.1	2	1	0	0	1	1	1	47.7	0.08	3.97
1	0	0	100	6.97	1	73	21	157	86	178	63	115	14.7	5	81.1	2	1	0	0	1	1	1	32.8	0.03	36.7
1	0	0	100	7	1	67	21.5	147.5	76	189	63	126	15.8	6.3	79.4	2	0	0	0	1	1	1	40.8	0.02	35.5
1	0	0	100	6.8	1	67	23.1	172	104	147	63	84	16.8	5.7	71.6	2	0	0	0	1	1	1	57.5	0.02	28.5
1	0	0	100	6.83	1	69	21.3	145.5	85	224	63	161	14.1	6.1	78.3	2	1	0	0	0	1	1	6.7	0.09	113
1	0	0	100	6.84	1	72	20.9	125.5	74	185	63	122	13.3	5.5	76.7	2	0	0	1	1	1	1	9.4	0.07	24.7
1	0	0	100	4.59	1	69	21.6	105	58.5	190	63	127	15.7	3.8	74.1	2	1	0	1	1	1	1	13.2	0.02	19.5
1	0	0	100	6.44	1	65	22.4	144	96	174	63	111	14.3	4.8	80.5	2	1	0	0	1	1	1	57.5	0.02	0.25
1	0	0	100	6.87	1	73	26.6	114	75	171	63	108	13.6	5.9	68.6	2	1	0	1	1	1	1	5	0.03	70.3
1	0	0	100	5.04	1	77	21	128	66	154	63	91	12.8	5.5	58.8	2	1	1	0	1	1	1	6.9	0.02	57
1	1	0	100	6.41	1	73	22.3	138.5	81.5	171	63	108	16.8	6.4	72.0	2	1	1	1	1	0	1	91.4	0.05	8.98
1	0	0	100	7	2	74	27.9	144	80	231	63	168	14.8	5.9	73.2	1	1	1	0	0	0	1	21.7	0.1	13
1	0	0	100	6.86	2	70	25.7	135.5	75.5	194	63	131	10.7	5.8	46.5	1	1	1	0	1	0	1	94	0.15	39.2
1	0	0	100	6.86	2	67	25.1	143	83	228	63	165	13	5.3	73.1	2	1	0	0	0	1	1	6.3	0.11	40.8
1	0	0	100	6.87	2	65	21	125	75	221	63	158	13.8	5.2	54.7	2	1	1	1	0	1	1	8.3	0.17	13.8
1	0	0	100	6.87	2	70	23.1	120.5	72	216	63	153	12.2	5.9	75.3	2	1	1	1	1	1	1	23.8	0.45	8.53
1	0	0	100	6.87	2	69	29.9	130.5	79	372	63	309	12.5	6	80.5	2	1	1	1	0	1	1	16	0.11	11.9
1	0	0	100	7.04	2	68	29.3	143.5	77.5	234	63	171	14.4	6.7	76.4	2	1	1	0	0	0	1	6.9	0.12	0.05

1	0	0	100	5.06	2	67	22.5	120	63.5	158	63	95	10.2	5.5	76.9	2	1	1	1	1	1	1	15.7	0.25	26.9
1	0	0	100	6.96	2	70	24.1	152.5	87	180	63	117	11.6	5.5	60.9	2	1	1	0	1	1	1	13.1	0.15	160
1	0	0	100	6.98	2	72	24.4	126.5	79	202	63	139	13.6	5.1	74.3	1	1	0	1	1	1	1	211	0.44	20
1	0	0	100	6.98	2	65	22.6	137.5	77.5	194	63	131	12.5	5.2	78.0	2	1	1	0	1	1	1	15.3	0.11	45.4
1	0	0	100	6.8	2	66	23.6	119	65	206	63	143	11.8	5.9	82.3	2	1	1	1	1	1	1	22.7	0.16	10.7
1	0	0	100	6.83	2	68	27.3	120	69	219	63	156	13.2	5.3	72.6	2	1	1	1	0	1	1	458.9	0.15	13.5
1	0	0	100	6.85	2	66	29.3	149	83.5	278	63	215	12.9	5.2	77.5	2	1	1	0	0	1	1	14.2	0.11	20.1
1	0	0	100	7	2	70	23.6	129.5	69.5	221	63	158	12.8	5.2	75.3	2	1	1	1	0	1	1	14.8	0.05	18.6
1	0	0	100	7.05	2	74	23.2	135	78.5	178	63	115	13.1	5.5	69.6	1	1	1	1	1	1	1	10.6	0.04	69.6
1	0	0	100	7.07	2	66	26.6	156.5	81	206	63	143	14.5	5.3	82.3	2	1	1	0	1	1	1	177.2	0.08	14.9
1	0	0	100	7.08	2	66	24	94	60.5	209	63	146	12.8	5.1	77.5	2	1	1	1	1	1	1	6.4	0.02	20.3
1	0	0	100	4.36	2	67	26.2	133	75.5	184	63	121	13.1	6.1	76.9	2	1	1	1	1	1	1	16.8	0.04	6.15
1	0	0	100	4.58	2	66	24.4	142	73	222	63	159	13.1	5.2	73.6	2	1	1	0	0	1	1	19.6	0.04	10.9
1	0	0	100	6.86	2	69	28.2	148	70.5	188	63	125	13.1	5.2	75.9	2	1	1	0	1	1	1	30.4	0.08	25.8
1	0	0	100	6.87	2	69	22.4	123	74.5	204	63	141	12.8	5.4	72.1	2	1	1	1	1	1	1	7.6	0.02	49
1	0	0	100	1.43	2	66	28.2	125	75	198	63	135	16	4.9	54.3	2	1	1	0	1	1	1	22.5	0.08	62.2
1	0	0	100	6.84	2	75	23.6	154	97.5	256	63	193	13.3	5.3	69.1	1	1	1	0	0	1	1	114.9	0.09	34.3
1	0	0	100	6.84	2	67	23.9	131	85.5	215	63	152	12	4.9	76.9	2	1	1	1	1	1	1	16.9	0.02	40.9
1	0	0	100	7	2	69	24.5	129	70.5	218	63	155	13	5.3	75.9	2	1	1	1	1	1	1	8	0.02	16.2
1	0	0	100	7.01	2	69	23.1	113.5	67.5	203	63	140	12	5.4	72.1	2	1	1	1	1	1	1	5.5	0.04	29.4
1	0	0	100	7.03	2	66	26.1	118.5	74	238	63	175	13.6	9.7	82.3	2	1	1	1	0	0	1	6.6	0.07	12.7
1	0	0	100	7.03	2	74	21.2	163	94	209	63	146	13.5	5.2	73.2	2	1	1	0	1	1	1	9.4	0.05	19.5
1	0	0	100	5.06	2	68	25.7	106.5	63	219	63	156	14.7	7.6	81.1	2	1	1	1	1	0	1	8	0.04	14.4
1	0	0	100	5.06	2	70	26.1	114.5	73	200	63	137	12.4	5.1	60.9	1	1	1	1	1	1	1	11.4	0.05	46.3
1	0	0	100	6.97	2	73	25.9	128.5	68.5	188	63	125	12.5	5	78.3	1	1	0	0	1	1	1	23.9	0.04	14
1	0	0	100	6.98	2	65	22.4	117	66	204	63	141	12.1	5.5	63.1	1	1	1	1	1	1	1	26.2	0.05	66.1
1	0	0	100	6.98	2	69	28.4	128.5	72	191	63	128	13.7	6	72.1	1	1	1	1	1	1	1	13	0.05	20.8
1	0	0	100	4.64	2	78	26.7	142.5	76	229	63	166	13.5	6.4	67.7	1	1	1	0	0	1	1	17.2	0.09	27.8
1	0	0	100	6.97	2	67	23.7	146.5	74	269	63	206	13.1	5.3	73.1	2	1	1	0	0	1	1	11	0.04	1.82
1	0	0	100	6.98	2	70	24.4	131.5	70	204	63	141	13.4	5.8	71.6	2	1	1	1	1	1	1	42	0.04	22.7
1	0	0	100	6.98	2	70	22.2	111	71	201	63	138	12	5.7	80.0	1	1	1	1	1	1	1	98.6	0.07	43.4
1	0	0	100	6.8	2	65	20.7	115	63	178	63	115	13.3	5	78.0	2	1	1	1	1	1	1	13	0.05	23
1	0	0	100	6.8	2	68	23.3	114.5	72	213	63	150	14.8	7.4	72.6	2	1	1	1	1	0	1	19	0.08	8.27
1	0	0	100	4.59	2	69	23.4	147.5	81	203	63	140	13	5.9	75.9	2	1	1	0	1	1	1	179.4	0.04	72.9
1	0	0	100	3.13	2	73	19.8	117.5	86.5	210	63	147	12.7	5.4	59.7	2	1	1	1	1	1	1	14.8	0.06	47.3
1	0	0	100	4.69	2	65	19.1	165	100.5	175	63	112	12.9	5.4	78.0	2	1	1	0	1	1	1	71.6	0.03	68
1	0	0	100	5.37	2	65	24.4	120.5	73.5	213	63	150	11.9	5.9	74.2	2	1	1	1	1	1	1	8.6	0.04	0.56
1	0	0	100	5.37	2	75	22.5	135.5	84.5	198	63	135	12.5	5.5	72.7	1	1	1	1	1	1	1	12.5	0.01	23.7
1	0	0	100	6.44	2	66	22.2	86	48	192	63	129	11.4	5.4	82.3	2	1	1	1	1	1	1	14.2	0.02	66.6
1	0	0	100	6.87	2	74	20.8	106.5	62	251	63	188	13.4	5.6	73.2	1	1	1	0	0	1	1	14.3	0.09	12.7
1	0	0	100	6.87	2	66	21	113	77.5	219	63	156	13.3	5.4	62.7	2	1	1	0	1	1	1	10.2	0.08	6.76
1	0	0	100	5.04	2	65	17.8	176.5	94.5	195	63	132	12.5	5.5	74.2	2	1	1	0	1	1	1	18	0.01	69.4
1	1	0	100	5.06	2	78	23.6	96.5	50	199	63	136	13.2	5.3	57.6	2	1	1	1	1	1	1	7.2	0.05	40
1	1	0	100	7.15	2	75	23.8	144.5	84	241	63	178	13.8	6.6	72.7	2	1	1	0	0	0	1	15	0.03	7.34
1	1	0	100	5.04	2	70	30.4	107	73	243	63	180	11.3	5.5	75.3	2	1	1	0	0	1	1	25.2	0.03	1.54
1	0	0	100	5.06	1	81	26.4	135.5	70	163	63	100	10.2	5.8	38.0	1	1	0	0	1	1	1	9.3	0.07	37.6
1	0	0	100	4.59	1	81	20.2	137	71.5	147	63	84	13.4	5.2	64.9	2	1	1	0	1	1	1	3.4	0.03	24.9
1	0	0	100	4.58	2	84	17.9	142	79	206	63	143	11.6	5.2	68.3	1	1	1	0	1	1	1	293.6	1.38	112
1	0	0	100	4.59	2	81	28.6	123	67	201	63	138	13.6	6.1	66.3	2	1	1	1	1	1	1	17.5	0.1	14.4
1	0	0	100	7.06	2	83	25.8	157.5	83	293	63	230	12.6	5.5	65.3	1	1	1	0	0	1	1	140.3	0.05	10.8
1	0	0	100	6.86	1	74	19.1	102	67.5	172	63	109	14.2	5.5	71.5	2	1	0	0	1	1	1	7.2	0.14	8.93

1	0	0	100	5.16	1	67	22.6	121.5	70.5	149	63	86	14.2	5.1	79.4	2	1	0	1	1	1	1	4	0.06	30.9
1	0	0	100	6.85	1	68	22.2	146.5	81.5	219	63	156	14	4.8	71.1	2	1	1	0	1	1	1	4.9	0.05	19.1
1	0	0	100	5.05	1	70	26.9	121	81.5	182	63	119	15.9	5.1	73.6	2	1	1	0	1	1	1	6.7	0.04	14.7
1	0	0	100	5.08	1	66	25.9	132.5	88.5	244	63	181	14.6	6.6	72.1	2	0	0	1	0	0	1	18.3	0.06	5.34
1	0	0	100	4.61	1	71	18.9	146.5	89	178	63	115	16.1	5.4	77.2	1	0	0	0	1	1	1	20.7	0.02	10.7
1	0	0	100	5.04	1	68	23.6	123.5	66.5	220	63	157	14.5	5.7	78.8	2	1	0	0	0	1	1	12.1	0.08	15.5
1	1	0	100	5.37	1	76	22.3	106	69.5	166	63	103	12.1	5.6	74.5	1	1	0	1	1	1	1	13	0.34	42.7
1	0	0	100	6.88	2	68	28.8	115	76	211	63	148	13.8	5.5	81.1	2	1	1	1	1	1	1	13.1	0.32	20.4
1	0	0	100	6.84	2	71	31.2	136	82.5	259	63	196	15.2	6.6	74.8	2	1	1	1	0	0	1	39.6	0.15	23.5
1	0	0	100	7.02	2	70	27.4	120.5	68.5	186	63	123	13.6	5.8	75.3	1	1	1	1	1	1	1	11.7	0.2	17.1
1	0	0	100	7.04	2	66	25.6	129	73.5	207	63	144	13.1	6.4	62.7	2	1	1	0	1	1	1	4.7	0.24	22.5
1	0	0	100	4.63	2	72	35.9	109	65.5	183	63	120	11.7	5.9	78.9	2	1	1	0	0	1	1	47.9	0.11	20.8
1	0	0	100	7.07	2	74	25	122	69	212	63	149	13.9	5.3	73.2	2	1	1	0	1	1	1	18.3	0.05	50.7
1	0	0	100	7.08	2	67	26.6	125	83.5	201	63	138	14	5.9	76.9	2	1	1	1	1	1	1	27.1	0.04	9
1	0	0	100	6.87	2	71	21.4	123	73	240	63	177	13.3	5	71.1	2	1	1	1	0	1	1	17.6	0.07	3.56
1	0	0	100	7.21	2	73	22.2	126.5	77	194	63	131	14.7	5	73.8	2	1	1	1	1	1	1	6.9	0.08	57.7
1	0	0	100	5.06	2	73	25	95.5	50	210	63	147	12.7	5.4	73.8	2	1	1	1	1	1	1	5.9	0.05	17.1
1	0	0	100	5.08	2	69	21	149.5	75.5	160	63	97	12.4	5.3	80.5	2	1	1	0	1	1	1	36.8	0.01	14.9
1	0	0	100	6.98	2	66	20.3	182	90	209	63	146	13.4	5.2	77.5	2	1	1	0	0	1	1	17.3	0.04	0.05
1	0	0	100	6.98	2	72	33.2	115.5	61.5	264	63	201	12.2	7.4	70.6	2	1	1	0	0	0	1	88.7	0.05	28.9
1	0	0	100	6.99	2	66	29.4	132.5	72	201	63	138	11	7.9	62.7	2	1	1	0	1	0	1	8.3	0.08	19.8
1	0	0	100	6.83	2	72	24.1	122	78	212	63	149	13.8	5.5	60.1	1	1	1	0	1	1	1	14.8	0.03	0.81
1	0	0	100	4.59	2	75	24.9	101	61	218	63	155	12.9	6	51.0	2	1	1	0	0	1	1	11.9	0.01	82.5
1	0	0	100	4.69	2	72	28.1	158.5	81.5	193	63	130	12.7	6.4	52.1	1	1	1	0	1	0	1	655.7	0.04	110
1	0	0	100	6.96	2	76	25.6	112.5	66	188	63	125	12.2	6.4	72.2	2	1	0	1	1	1	1	8.8	0.07	0.05
1	0	0	100	5.06	2	78	19.1	131.5	80	143	63	80	12.5	5.7	67.7	2	1	1	1	1	1	1	29.7	0.01	14.8
1	0	0	100	6.45	2	71	27.9	103.5	58.5	167	63	104	13.4	5.5	74.8	2	1	1	1	1	1	1	20.6	0.02	7.89
1	1	0	100	7.2	2	75	30.9	172.5	96	245	63	182	14	5.5	58.8	2	1	1	0	0	1	1	13.2	0.33	24.7
1	1	0	100	4.91	2	65	21.3	98.5	60	223	63	160	11.2	6.5	82.8	2	1	1	1	0	0	1	7.7	0.01	24.9
1	1	0	100	4.63	2	76	29.1	136.5	78.5	215	63	152	12.7	8	68.6	2	1	1	1	1	0	1	89.6	0.04	15
1	1	0	100	6.86	1	86	21.5	161.5	71.5	183	63	120	13.1	5.4	65.8	1	1	1	0	1	1	1	18.2	0.04	113
1	1	0	100	6.99	2	89	28.1	130.5	73.5	194	63	131	14.2	5.3	65.9	2	1	1	0	1	1	1	6.6	0.05	32.8
1	1	1	2	2	1	75	21.9	163	86.5	167	63	104	14.6	5.3	59.6	2	0	0	0	1	1	1	458.3	0.02	112
1	0	1	5.1	5.1	2	70	25.1	153.5	78	209	63	146	14	5.5	75.3	1	1	1	0	1	1	1	4.2	0.03	42.4
1	1	1	3.7	3.7	2	73	23	113	53.5	156	63	93	11.2	5.4	73.8	1	1	1	1	1	1	1	14.2	0.01	43.4
1	0	1	6.3	6.3	1	78	19.3	156.5	82.5	154	63	91	10.4	6	69.6	2	1	1	0	1	1	1	23.3	0.15	0.05
1	1	1	0.8	0.8	1	77	25.4	142	79	178	63	115	15.3	4.5	66.7	2	1	0	0	1	1	0	51.5	0.04	119
1	1	1	0.9	0.9	1	72	19.9	128	73	135	63	72	12	5.3	76.7	1	1	1	0	1	1	1	53.4	0.01	38.3
1	0	0	100	7.07	1	67	30.3	142	80.5	211	64	147	16.8	5	75.2	2	1	0	0	1	1	1	33.8	0.31	0.05
1	0	0	100	5.15	1	70	24.3	144	83	174	64	110	14.7	5.3	70.1	2	1	0	0	1	1	1	43	0.1	19.2
1	0	0	100	6.88	1	74	22.4	156	89.5	176	64	112	13.5	5.7	75.6	2	0	0	0	1	1	1	20.1	0.12	15.4
1	0	0	100	6.87	1	67	23.6	117.5	73	232	64	168	13.8	5.2	71.6	2	1	0	1	0	1	1	15.2	0.11	5.72
1	0	0	100	6.98	1	68	24.1	127.5	84	164	64	100	12	5.3	71.1	2	1	0	0	1	1	1	6.6	0.13	5.57
1	0	0	100	4.6	1	69	29.8	162.5	106.5	153	64	89	16.2	5.1	78.3	2	0	0	0	1	1	1	27.3	0.11	19.1
1	0	0	100	7	1	68	21.3	131	76	146	64	82	12.4	5.5	71.1	2	1	0	0	1	1	1	69.9	0.04	14.4
1	0	0	100	7.07	1	66	24.8	129	81.5	215	64	151	15.1	5.3	72.1	2	1	0	0	1	1	1	17	0.06	2.08
1	0	0	100	6.97	1	67	25.5	149	90	233	64	169	14.8	5.7	84.6	2	1	0	0	0	1	1	74.4	0.02	4.92
1	0	0	100	6.97	1	73	24.5	131	67.5	211	64	147	13.4	5.1	68.6	1	1	1	0	1	1	1	27.4	0.05	14.9
1	0	0	100	6.98	1	65	22.4	147.5	82	177	64	113	13.2	5.9	76.2	2	0	1	0	1	1	1	3.2	0.03	4.7
1	0	0	100	6.99	1	69	19.3	113.5	75	191	64	127	12.9	4.6	78.3	2	1	0	1	1	1	1	3.1	0.01	33.9
1	0	0	100	7	1	65	26.2	118.5	80.5	205	64	141	15	4.9	80.5	2	0	0	1	1	1	1	7.1	0.04	10.1

1	0	0	100	6.95	1	73	22.2	116.5	72.5	187	64	123	13.8	5.4	76.1	2	1	0	1	1	1	1	36.1	0.01	13.8
1	0	0	100	6.8	1	71	18.6	131.5	86.5	181	64	117	12.9	5.4	54.6	2	1	1	1	1	1	1	7.2	0.02	14.4
1	0	0	100	6.8	1	66	22.9	177	92	258	64	194	14.6	5.4	75.7	2	1	1	0	0	1	1	13.7	0.04	39.4
1	0	0	100	6.8	1	71	23	127.5	69.5	215	64	151	13.7	5.3	61.3	2	1	0	1	1	1	1	5.2	0.07	33.4
1	0	0	100	6.8	1	68	25.1	121	76	214	64	150	14.8	5.4	71.1	1	1	0	1	1	1	1	8.7	0.06	14.2
1	0	0	100	6.83	1	72	20.6	133.5	77	163	64	99	12	6.3	81.7	1	1	0	1	1	1	1	124.2	0.02	18.7
1	0	0	100	2.54	1	75	21.3	150.5	93.5	234	64	170	13	5.2	71.0	2	1	1	0	0	1	1	5.6	0.01	22.9
1	0	0	100	4.62	1	69	25.9	144.5	80.5	238	64	174	15.5	5.7	70.6	2	1	0	0	0	1	1	26.5	0.03	4.96
1	0	0	100	6.79	1	72	18.7	141.5	90	203	64	139	13.2	7.1	69.1	2	0	1	0	1	0	1	20.2	0.03	0.9
1	0	0	100	5.37	1	66	20.6	123.5	81.5	176	64	112	16	5.5	85.2	2	1	0	0	1	1	1	12.7	0.09	9.61
1	0	0	100	6.86	1	69	21.8	141	85.5	197	64	133	16.8	5.1	74.1	2	1	0	0	1	1	1	6.9	0.01	9.66
1	0	0	100	5.04	1	69	25	135	75.5	189	64	125	12.7	5.9	74.1	2	1	0	0	1	1	1	7.1	0.03	24.4
1	1	0	100	6.96	1	78	22	140	74	200	64	136	14.4	8.6	78.3	2	0	0	0	1	0	1	96.5	0.05	23.5
1	0	0	100	7	2	70	26	99.5	56.5	253	64	189	12.9	5.8	71.6	1	1	1	1	0	1	1	6.6	0.12	28.2
1	0	0	100	6.86	2	71	26.1	112.5	62	220	64	156	13.4	5.2	71.1	1	1	1	1	0	1	1	6.4	0.19	21.3
1	0	0	100	7.03	2	67	25.1	115	71.5	228	64	164	14.9	5.7	76.9	1	1	1	1	0	1	1	14.6	0.13	6.45
1	0	0	100	5.05	2	71	24.3	133	74	175	64	111	13.7	5.4	74.8	1	1	1	0	1	1	1	10.1	0.59	16.1
1	0	0	100	6.98	2	67	25.9	148.5	84.5	208	64	144	13.1	5.6	73.1	2	1	1	0	1	1	1	79.9	0.21	3.73
1	0	0	100	4.6	2	68	19.9	120	77	193	64	129	13.6	5.3	76.4	2	1	1	1	1	1	1	32.4	0.71	33.1
1	0	0	100	1.68	2	65	24.3	127	66.5	221	64	157	11.8	5.5	74.2	2	1	1	1	0	1	1	6.2	0.03	27.1
1	0	0	100	7	2	66	23.9	127	71	216	64	152	14.7	4.9	82.3	1	1	1	1	1	1	1	25.2	0.01	26.8
1	0	0	100	7	2	67	25.1	144	83.5	198	64	134	13.2	6.6	81.7	2	1	1	0	1	0	1	28.3	0.05	20.3
1	0	0	100	7.06	2	66	24.5	116.5	69.5	212	64	148	13.1	5.2	77.5	2	1	1	1	1	1	1	39.6	0.03	53.5
1	0	0	100	7.07	2	71	23	131.5	68	206	64	142	13.3	5.2	71.1	2	1	1	0	1	1	1	9	0.07	10.3
1	0	0	100	7.08	2	67	25.4	119	75.5	211	64	147	14.2	5.2	81.7	2	1	1	1	1	1	1	13	0.02	0.05
1	0	0	100	4.58	2	75	23.8	139.5	75.5	202	64	138	13.9	5.6	77.2	1	1	1	0	1	1	1	11.5	0.02	1.43
1	0	0	100	4.58	2	69	21.9	166.5	96	230	64	166	13.8	5.5	80.5	2	1	1	0	0	1	1	30.7	0.03	21.5
1	0	0	100	6.87	2	68	29	144	75	251	64	187	13.9	5.8	81.1	2	1	1	0	0	1	1	15	0.04	33.1
1	0	0	100	5.16	2	65	25.3	116.5	65	233	64	169	13	5.4	74.2	2	1	1	1	0	1	1	21.9	0.01	9.49
1	0	0	100	6.85	2	70	23.8	125.5	76.5	226	64	162	13.3	4.8	75.3	2	1	1	0	0	1	1	66.5	0.03	7.36
1	0	0	100	6.85	2	69	22.9	93	53	197	64	133	12.7	5.8	86.7	2	1	1	1	1	1	1	13.3	0.03	8.76
1	0	0	100	2.39	2	66	18.9	109.5	59	211	64	147	12.6	5.1	73.6	2	1	1	1	1	1	1	8.2	0.01	61.9
1	0	0	100	7.01	2	66	29.5	125.5	77	191	64	127	13	5.5	82.3	2	1	1	1	1	1	1	7.7	0.04	7.22
1	0	0	100	7.04	2	72	21.5	108	70.5	214	64	150	11.6	5.3	70.6	2	1	1	1	1	1	1	3.8	0.03	32.1
1	0	0	100	5.07	2	71	24.4	129	78	218	64	154	15.4	5.1	71.1	2	1	1	1	1	1	1	35.9	0.03	18.3
1	0	0	100	6.95	2	71	25.9	159.5	84.5	187	64	123	12.8	5.3	74.8	1	1	1	0	0	1	1	27.6	0.04	88.7
1	0	0	100	6.96	2	79	23	152.5	84	213	64	149	13.9	5.3	70.7	1	1	1	0	1	1	1	38.3	0.08	21.9
1	0	0	100	2.09	2	71	28.4	150	85.5	220	64	156	13.4	5.3	71.1	1	1	1	0	0	1	1	37.1	0.04	20.1
1	0	0	100	4.59	2	66	24.9	132	79	202	64	138	13.9	5.3	73.6	2	1	1	0	1	1	1	6.3	0.05	25.4
1	0	0	100	4.59	2	76	20.9	149.5	74	185	64	121	12.9	5.4	58.4	2	1	1	0	0	1	1	63.5	0.06	46.6
1	0	0	100	4.6	2	65	25.1	147	81	210	64	146	13.9	5.2	74.2	2	1	0	0	1	1	1	17.2	0.04	20.1
1	0	0	100	4.62	2	78	22.5	136.5	77	216	64	152	11.1	5.6	71.2	2	1	1	0	1	1	1	6.1	0.06	31.2
1	0	0	100	6.44	2	69	22.6	136.5	86.5	171	64	107	13.1	5.1	80.5	2	1	1	0	1	1	1	91.7	0.02	49
1	0	0	100	4.67	2	69	26.1	125.5	67.5	218	64	154	14.5	5.3	75.9	2	1	1	1	1	1	1	30.4	0.03	56.3
1	0	0	100	4.69	2	69	23.6	109	69	176	64	112	12.3	5	80.5	2	1	1	1	1	1	1	21	0.02	34.2
1	0	0	100	5.37	2	67	24.2	116.5	74	183	64	119	13.7	5.6	76.9	2	1	0	0	1	1	1	20.7	0.01	11.7
1	0	0	100	5.37	2	66	25.6	131	75.5	194	64	130	12.1	5.5	62.7	2	1	1	0	1	1	1	15.6	0.02	64.4
1	0	0	100	5.06	2	66	27	155.5	84.5	218	64	154	12.8	5.8	62.7	2	1	1	0	1	1	1	26.2	0.03	3.59
1	1	0	100	4.63	2	74	27.9	136.5	73	218	64	154	13.3	6.6	69.6	2	1	1	0	1	0	1	25.8	0.18	39.9
1	1	0	100	6.84	2	77	20.7	155.5	85	183	64	119	11.6	5.6	71.7	1	1	1	0	1	1	1	19.8	0.05	17.6
1	0	0	100	6.82	1	83	22.2	105	62	162	64	98	13.2	5.9	67.2	1	1	0	1	1	1	1	10	0.01	26.6

1	0	0	100	6.83	1	81	26.2	122	69	158	64	94	12.3	5.2	64.9	2	1	1	1	1	1	1	83.4	0.08	35.6
1	0	0	100	6.84	1	81	23.2	168	85.5	165	64	101	14.6	5.1	64.9	1	1	0	0	1	1	1	7	0.03	82.6
1	0	0	100	6.87	2	85	28.8	140	75.5	232	64	168	13.5	5.9	47.6	1	1	1	0	0	1	0	1527.7	0.05	169
1	1	0	100	4.1	2	81	25.5	127.5	68	196	64	132	11.6	6.2	48.9	1	1	1	0	1	1	1	14.1	0.02	237
1	0	0	100	7	1	66	22.5	91	53.5	188	64	124	15.9	6.4	79.9	2	1	1	1	1	1	1	18.5	0.15	30.7
1	0	0	100	6.98	1	73	18.7	129.5	71	148	64	84	13	5.5	76.1	2	1	0	0	1	0	1	18.6	0.11	0.05
1	0	0	100	6.97	1	68	24.7	158	85.5	205	64	141	14.4	5.4	78.8	2	1	0	0	1	1	1	35.4	0.16	32.4
1	0	0	100	6.84	1	76	20.5	142	84.5	172	64	108	14.7	5.6	70.5	2	1	1	0	1	1	1	12	0.08	48.6
1	0	0	100	6.44	1	68	26.4	146.5	91.5	203	64	139	14.5	5.6	78.8	2	0	0	0	1	1	1	25.1	0.02	1.03
1	0	0	100	5.04	1	66	26.1	112.5	66.5	173	64	109	17	5.2	85.2	2	1	1	0	1	1	1	22.1	0.04	10.3
1	1	0	100	6.98	1	74	19.2	153	72.5	180	64	116	13.8	6.1	75.6	2	1	0	0	1	1	1	14.9	0.04	1.28
1	0	0	100	7.23	2	71	20.9	142	71.5	201	64	137	12.2	5.3	74.8	2	1	1	0	1	1	1	8.7	0.11	204
1	0	0	100	6.97	2	74	22.7	112.5	57.5	198	64	134	11.8	5.6	69.6	1	1	1	0	1	1	1	8.3	0.17	3.64
1	0	0	100	6.98	2	75	26.6	124	68.5	218	64	154	13.6	8.2	72.7	1	1	1	1	1	0	1	11.3	0.15	30.7
1	0	0	100	6.98	2	67	22.8	151.5	90.5	187	64	123	12.5	4.8	73.1	2	1	1	0	1	1	1	37.8	0.38	75
1	0	0	100	5.06	2	65	24.1	112	69	205	64	141	13.8	5.5	78.0	2	1	0	1	0	1	1	9.4	0.21	6.37
1	0	0	100	6.85	2	77	25.5	158.5	89.5	180	64	116	14.6	5.4	68.2	2	1	1	0	1	1	1	103.9	0.13	49.5
1	0	0	100	5.04	2	75	21.7	119	65.5	175	64	111	11.6	6.3	77.2	1	1	1	1	1	1	1	16.5	0.54	33.7
1	0	0	100	7.05	2	68	25.7	136	87.5	146	64	82	14.6	4	76.4	1	1	0	1	1	1	1	4.8	0.04	13.5
1	0	0	100	7.05	2	65	23	153.5	85	236	64	172	13.1	5.3	78.0	2	1	1	0	0	1	1	10.6	0.04	23.2
1	0	0	100	7.07	2	78	23.6	124.5	74	200	64	136	12.9	5.6	71.2	1	1	1	1	1	1	1	16.2	0.07	5.52
1	0	0	100	6.86	2	71	24.5	106	64.5	216	64	152	13.4	5.1	74.8	2	1	1	1	1	1	1	16.1	0.03	31.3
1	0	0	100	6.88	2	71	19.2	120.5	69	249	64	185	12.9	5.8	71.1	2	1	1	1	0	1	1	16.7	0.02	39.4
1	0	0	100	6.88	2	70	27.9	111	66.5	204	64	140	14.5	5.9	71.6	2	1	1	0	1	1	1	6.7	0.03	17.3
1	0	0	100	5.16	2	69	25.2	145	82.5	217	64	153	14.4	5.7	72.1	2	1	1	0	1	1	1	50.6	0.07	6.47
1	0	0	100	6.84	2	75	19.8	107.5	60.5	192	64	128	12.5	5.2	72.7	2	1	1	1	1	1	1	10.2	0.01	70.7
1	0	0	100	7.21	2	71	28.8	178	89.5	191	64	127	12	5.4	79.4	2	1	0	0	1	1	1	46.9	0.08	9.03
1	0	0	100	6.86	2	70	19.7	125	76.5	242	64	178	13.3	5.4	71.6	2	1	1	0	0	1	1	5.4	0.02	17.1
1	0	0	100	5.05	2	69	21.8	139	83	169	64	105	13.4	5.7	75.9	2	1	0	1	1	1	0	32.2	0.02	8.94
1	0	0	100	5.05	2	68	21.7	116.5	62	212	64	148	12	5.7	81.1	2	1	1	1	1	1	1	32.2	0.02	22.5
1	0	0	100	6.99	2	67	24.6	121.5	75.5	185	64	121	13.6	4.9	81.7	2	1	0	1	1	1	1	15.3	0.05	54
1	0	0	100	4.63	2	76	26.3	134.5	76.5	242	64	178	13.6	5.7	68.6	2	1	1	0	0	1	1	15.4	0.07	43.3
1	0	0	100	7	2	66	25	149	91.5	186	64	122	15.9	5.1	77.5	2	1	1	0	1	1	1	19.7	0.02	0.05
1	0	0	100	6.8	2	67	25.5	131	87	253	64	189	13.3	5.6	76.9	1	1	1	1	0	1	1	13.8	0.03	32.8
1	0	0	100	4.61	2	76	29.6	104	60	180	64	116	11.9	5.4	68.6	2	1	1	1	1	1	1	20.3	0.01	21.2
1	0	0	100	6.96	2	70	28.1	103.5	62.5	236	64	172	14.1	5.5	75.3	2	1	0	0	0	1	1	16.8	0.07	15.2
1	0	0	100	5.05	2	68	27.1	109	64	234	64	170	12.5	4.9	81.1	2	1	1	0	0	1	1	46.7	0.03	21.8
1	0	0	100	4.18	2	74	28.3	143	86.5	176	64	112	14.5	5.1	69.6	2	1	1	0	1	1	1	20.9	0.07	22.1
1	0	0	100	5.04	2	75	22	129	70	229	64	165	13.4	5.3	77.2	2	1	1	0	0	1	1	80.7	0.08	79.4
1	1	0	100	7.08	2	70	31.1	132	82	190	64	126	13.8	5.8	75.3	1	1	1	0	1	1	1	13.9	0.15	5.91
1	1	0	100	3.34	2	77	22.9	123.5	73	186	64	122	14.4	5.2	58.0	1	1	1	0	1	1	1	37.7	0.08	18.1
1	1	0	100	7.07	2	68	27	133	73	203	64	139	13.4	5.5	61.8	2	1	1	0	1	1	1	44.7	0.05	50.8
1	1	0	100	5.15	2	77	18.5	105.5	65.5	236	64	172	12.5	6.7	76.1	1	1	1	0	0	0	1	53.6	0.02	43.4
1	1	0	100	6.85	2	78	22.9	144	78	229	64	165	13.6	5.4	75.6	2	1	1	0	0	1	1	15.7	0.03	15.6
1	1	0	100	6.95	2	71	20.2	103.5	56.5	219	64	155	11.6	5.8	85.5	1	1	1	1	1	1	1	16.7	0.01	13.4
1	1	0	100	6.95	2	77	20	152.5	88	190	64	126	11.7	5.4	71.7	1	1	1	0	1	1	1	17.4	0.01	4.17
1	1	0	100	4.62	2	75	28.1	144	86.5	170	64	106	14.2	5.3	69.1	1	1	1	0	1	1	1	181.3	0.08	45.8
1	1	0	100	6.86	2	77	18.8	122	63.5	209	64	145	11.4	5.8	76.1	2	1	1	0	1	1	1	9.8	0.01	84.2
1	1	0	100	4.62	1	82	23.2	167.5	79.5	203	64	139	13.6	4.9	71.5	2	1	1	0	1	1	1	3.8	1.9	75.7
1	1	0	100	6.96	1	80	24.7	108.5	68.5	211	64	147	14.7	5.2	68.6	2	1	0	0	1	1	1	86.4	0.09	34.1
1	0	1	3.6	3.6	1	70	23.2	175.5	90.5	200	64	136	14.3	5.5	77.7	1	0	0	0	1	1	1	104	0.1	62.8

1	0	1	4.9	4.9	1	73	19.5	111.5	65	188	64	124	12.7	5.9	72.0	2	0	1	0	1	1	1	73.6	0.08	48
1	1	1	2.9	2.9	1	75	20.6	103	55.5	150	64	86	13.1	6.2	80.0	2	0	0	1	1	1	1	66.9	0.03	68.1
1	0	1	5.5	5.5	1	85	22.5	110.5	67.5	177	64	113	11.4	5.6	63.1	1	1	1	1	1	1	1	5.2	0.02	36.8
1	0	1	5.5	5.5	1	79	25.2	131	72.5	230	64	166	15	6.1	65.8	2	0	0	0	0	1	1	63.9	0.16	2.58
1	1	1	3.8	3.8	1	74	20	160.5	88.5	189	64	125	15.3	5	71.5	2	0	0	0	1	1	1	118.2	0.08	46.8
1	0	1	2.1	2.1	2	68	26.9	124.5	79	223	64	159	13.2	5.3	72.6	2	1	1	1	0	1	1	8.8	0.06	47.6
1	0	1	1.2	1.2	2	70	31.5	158.5	85	218	64	154	11.3	5.2	75.3	2	1	1	0	1	1	1	1593.9	0.03	15.7
1	0	0	100	2.91	1	73	25.7	123	74.5	175	65	110	13	5.4	60.4	2	1	1	1	1	1	0	22.5	0.14	220
1	0	0	100	6.98	1	66	26	137.5	81	223	65	158	14.5	5.6	75.7	2	1	0	1	0	1	1	81.5	0.1	23.2
1	0	0	100	2.8	1	74	23.9	120.5	78.5	255	65	190	14.3	5.2	60.0	2	1	0	0	0	1	1	2.7	0.27	46.4
1	0	0	100	4.59	1	71	20.6	108	50	192	65	127	14.6	6.2	37.5	2	1	0	1	1	1	1	77.2	0.18	28.9
1	0	0	100	6.36	1	65	21.4	110.5	70	203	65	138	16.3	12.4	76.2	2	1	0	1	1	0	1	86.8	0.45	8.06
1	0	0	100	5.37	1	65	24.5	123	72	197	65	132	15.3	5.4	76.2	2	1	0	0	1	1	1	9.6	0.11	46.2
1	0	0	100	5.04	1	67	21.1	107	60.5	187	65	122	14.9	5.5	79.4	2	1	0	1	1	1	1	14	0.98	4.59
1	0	0	100	4.58	1	69	22.3	132.5	85.5	220	65	155	12.9	5.6	70.6	2	1	0	1	0	1	1	6.7	0.03	17.2
1	0	0	100	4.58	1	75	24.7	166.5	86.5	197	65	132	14.8	5.7	75.1	2	1	0	0	1	1	1	6.7	0.03	18.4
1	0	0	100	6.88	1	66	21.2	126	82	190	65	125	15.2	8.2	85.2	2	0	0	1	1	0	1	25.4	0.03	15.9
1	0	0	100	6.88	1	69	23.5	122	74.5	200	65	135	15	5.4	70.6	2	1	0	1	1	1	1	16.3	0.05	19.1
1	0	0	100	5.16	1	69	25.3	146	99.5	236	65	171	15.4	5.3	62.2	2	1	1	0	0	1	1	15.7	0.04	4.19
1	0	0	100	7.2	1	68	20.3	106	67	148	65	83	12.5	5.8	71.1	2	1	1	1	1	1	1	10.3	0.01	22.7
1	0	0	100	7.04	1	71	21.4	165	92	185	65	120	16.8	5.4	61.3	2	1	0	0	1	1	0	52.7	0.08	126
1	0	0	100	5.08	1	65	21.8	147	90.5	210	65	145	14.3	6.2	72.6	2	1	0	0	1	0	1	19.1	0.02	26.7
1	0	0	100	6.99	1	74	21.1	116.5	70	198	65	133	11.5	5.1	68.2	1	0	0	1	1	1	1	12	0.02	29.6
1	0	0	100	3.97	1	65	25.8	97	63	218	65	153	15.2	5.1	63.9	2	1	0	1	1	1	1	4.6	0.04	14
1	0	0	100	4.63	1	77	24.2	119	67.5	200	65	135	14.5	5.4	58.8	2	0	1	1	1	1	1	8.9	0.08	11.2
1	0	0	100	6.95	1	67	21.7	126.5	76	216	65	151	12	5	79.4	2	1	1	1	1	1	1	8.7	0.03	26.7
1	0	0	100	6.96	1	65	24.9	130	78.5	213	65	148	15.1	4.8	76.2	2	1	0	1	1	1	1	36.9	0.04	29.4
1	0	0	100	6.98	1	67	21.2	138.5	74.5	192	65	127	14	4.9	75.2	2	1	0	1	1	1	1	5	0.01	6.41
1	0	0	100	6.79	1	67	24.6	122.5	85.5	223	65	158	14.5	6.9	75.2	2	1	0	1	0	0	1	8.5	0.02	11.5
1	0	0	100	5.36	1	66	25.6	148.5	95.5	229	65	164	14.7	5	79.9	2	1	0	0	0	1	1	32.4	0.04	53.6
1	1	0	100	7.06	1	77	22.3	138	65	187	65	122	13	5	66.7	1	1	1	1	1	1	1	16.3	0.09	11.1
1	0	0	100	7	2	76	22	111.5	67	180	65	115	12	5.3	76.7	1	1	1	1	1	1	1	12.5	0.14	23.1
1	0	0	100	4.59	2	71	25.6	157	87.5	209	65	144	12.3	5	74.8	2	1	1	0	1	1	1	41.3	0.36	30.3
1	0	0	100	6.87	2	71	22.8	161	85	259	65	194	13.6	5.6	74.8	2	1	1	0	0	1	1	6.8	0.1	14.2
1	0	0	100	6.87	2	77	19.5	98	53.5	213	65	148	11.4	6.8	58.0	1	1	1	1	1	0	1	12.7	0.17	18.4
1	0	0	100	6.87	2	75	17.6	124	62.5	227	65	162	12.1	5.2	72.7	1	1	1	1	0	1	1	9.2	0.18	27.5
1	0	0	100	7.02	2	71	25.7	118.5	73.5	221	65	156	13.2	5.6	74.8	2	1	1	1	0	1	1	4.7	0.28	40.8
1	0	0	100	4.61	2	69	24	134.5	76.5	210	65	145	13.3	5.3	75.9	2	1	1	1	1	1	1	9.9	0.13	15
1	0	0	100	5.06	2	72	27	117.5	70	220	65	155	13	5.6	74.3	2	1	1	1	0	1	1	23.3	0.13	11.9
1	0	0	100	5.06	2	65	25.1	106	55	182	65	117	13.2	4.9	78.0	2	1	1	0	1	1	1	22.5	0.46	56.1
1	0	0	100	6.86	2	68	22.2	133	73.5	228	65	163	13.3	5.2	81.1	2	1	1	0	0	1	1	15.2	0.23	26.5
1	0	0	100	6.87	2	70	24.5	152	82.5	195	65	130	12.4	5.6	80.0	1	1	1	0	1	1	1	169.6	0.28	72.2
1	0	0	100	7.07	2	72	27.8	149.5	81.5	273	65	208	14.2	5.2	52.1	2	1	1	0	0	1	1	28.2	0.04	61.4
1	0	0	100	7.08	2	70	24.6	138	81	233	65	168	12.6	5.3	71.6	2	1	0	1	0	1	1	9.7	0.06	31.9
1	0	0	100	7.08	2	72	24	130	82	255	65	190	14.7	5.5	74.3	2	1	1	1	0	1	1	15.6	0.02	19.6
1	0	0	100	6.86	2	65	23	128.5	81	215	65	150	12.6	5.8	78.0	2	1	1	1	1	1	1	27.3	0.05	23.1
1	0	0	100	5.16	2	69	29.7	135	80	232	65	167	13.7	5.8	61.4	2	1	1	0	0	1	1	19.9	0.04	75.9
1	0	0	100	6.84	2	69	25.5	121	78.5	231	65	166	15.2	5	80.5	2	1	1	1	0	1	1	9.4	0.04	6.91
1	0	0	100	6.85	2	74	22.2	137	79	209	65	144	12.4	5.4	59.2	1	1	1	1	1	1	1	6.6	0.05	16.7
1	0	0	100	6.85	2	68	18.2	126	76	197	65	132	12.1	6.2	72.6	2	1	1	0	1	1	1	8.8	0.03	42.8
1	0	0	100	6.85	2	70	30.7	126	73.5	247	65	182	15.3	7.2	41.5	2	1	1	0	0	0	1	16	0.03	29.3

1	0	0	100	6.85	2	68	20.6	114.5	66	233	65	168	14.8	5.5	76.4	1	1	1	1	0	1	1	9	0.01	12.5
1	0	0	100	7.01	2	75	23	91	61	230	65	165	13.3	5.7	72.7	1	1	1	1	0	1	1	14.9	0.03	22.2
1	0	0	100	5.05	2	65	22.1	141.5	81.5	164	65	99	12	5.6	78.0	2	1	0	0	1	1	1	26.3	0.01	16
1	0	0	100	5.06	2	75	24.9	121.5	68.5	198	65	133	13.7	5.4	72.7	2	1	1	1	1	1	1	5.2	0.04	9.68
1	0	0	100	6.97	2	73	22.2	129.5	81.5	223	65	158	12.1	5.4	78.3	1	1	1	1	0	1	1	77.6	0.07	9.04
1	0	0	100	6.98	2	70	21.9	93.5	56.5	208	65	143	12	5.4	60.9	1	1	1	1	1	1	1	4.4	0.07	17.4
1	0	0	100	6.98	2	65	24.2	107.5	66.5	253	65	188	11.8	5.6	82.8	2	1	0	1	0	1	1	4.4	0.05	28.4
1	0	0	100	6.98	2	73	19	91.5	60.5	171	65	106	11.4	5.1	73.8	1	1	1	1	1	1	1	17.3	0.07	9.72
1	0	0	100	4.62	2	72	24.3	139.5	73	221	65	156	13.4	5.8	70.6	2	1	1	0	0	1	1	13.6	0.04	26
1	0	0	100	4.63	2	68	19.6	181	101	218	65	153	12.5	5.4	81.1	2	1	1	0	1	1	1	21.8	0.03	65
1	0	0	100	4.64	2	71	23.7	123.5	76.5	216	65	151	13	5.3	74.8	2	1	1	0	1	1	1	21.1	0.02	35.6
1	0	0	100	2.04	2	66	21.5	118.5	66.5	227	65	162	13.3	5.8	77.5	2	1	1	1	0	1	1	29.4	0.04	19.1
1	0	0	100	6.96	2	70	16.9	105	59.5	177	65	112	12.7	5.4	75.3	2	1	1	1	1	1	1	41.5	0.02	67.4
1	0	0	100	7.15	2	67	31.5	114.5	71.5	204	65	139	13.4	6	76.9	2	1	1	0	1	1	1	8.1	0.03	12
1	0	0	100	6.8	2	70	21.6	94.5	61	200	65	135	13.4	5.7	75.3	2	1	1	1	1	1	1	6.4	0.05	7.84
1	0	0	100	6.8	2	77	26	164.5	92	239	65	174	13.8	5.6	71.7	1	1	1	0	0	1	1	74.4	0.03	134
1	0	0	100	6.83	2	66	23.4	95	57	218	65	153	11.9	5.5	77.5	2	1	1	1	1	1	1	12.2	0.05	25.4
1	0	0	100	4.59	2	74	23.9	141.5	74	175	65	110	12.5	5.5	73.2	2	1	1	0	1	1	1	41	0.06	38.6
1	0	0	100	5.02	2	68	23	101.5	52	191	65	126	13.2	5.3	72.6	2	1	1	1	0	1	1	7.6	0.01	24.6
1	0	0	100	4.6	2	68	24.5	146	79	233	65	168	10.7	6.4	81.1	2	1	1	0	0	1	1	39.9	0.02	48.6
1	0	0	100	4.61	2	68	23.4	113.5	62.5	235	65	170	15.2	5.3	72.6	2	1	1	0	0	1	1	5.4	0.01	9.8
1	0	0	100	4.61	2	71	24.2	121	69	161	65	96	13.9	5.8	74.8	2	1	0	0	1	1	1	52.8	0.03	58.1
1	0	0	100	4.69	2	65	25.2	149.5	82	170	65	105	13.2	4.7	89.1	2	1	0	0	1	1	1	46.2	0.08	86.5
1	0	0	100	6.85	2	66	23.7	116	76	219	65	154	12.1	5.3	73.6	2	1	1	1	1	1	1	18.1	0.01	9.48
1	0	0	100	6.85	2	68	21.1	133.5	74	198	65	133	12.6	5.4	76.4	1	1	1	1	0	1	1	24.6	0.02	22.1
1	0	0	100	6.86	2	75	19.8	113	62.5	255	65	190	12	5.3	69.1	2	1	1	1	0	1	1	54.1	0.01	13.3
1	0	0	100	6.87	2	66	21.9	112	71.5	153	65	88	14.8	5.4	77.5	2	1	0	1	1	1	1	60.8	0.04	280
1	1	0	100	6.87	2	71	27.1	126	68.5	188	65	123	15.2	5.2	71.1	2	1	1	0	1	1	1	11.2	0.02	25.7
1	1	0	100	7.21	2	73	22.9	126	65.5	235	65	170	13.8	5.4	70.1	2	1	1	0	0	1	1	14.8	0.03	70.7
1	1	0	100	4.62	2	71	23.8	125	78	214	65	149	11.8	5.6	71.1	2	1	1	0	1	1	1	9.4	0.04	19.9
1	1	0	100	6.87	2	74	17.2	133	86.5	144	65	79	12.7	5.8	77.8	2	1	1	1	1	1	1	33.7	0.01	26
1	1	0	100	6.85	1	82	22.6	129	77.5	181	65	116	14.4	4.9	71.5	1	1	0	1	1	1	1	10.4	0.02	3.94
1	0	0	100	5.49	2	80	28.6	156	86	252	65	187	14.7	5.4	66.7	1	1	1	0	0	1	1	26	0.58	67
1	0	0	100	4.64	2	81	24.1	143.5	81	211	65	146	11.9	6.1	56.4	1	1	1	0	1	1	1	50.8	0.01	34.2
1	0	0	100	6.86	1	79	19.7	105	59.5	200	65	135	13.8	5.4	46.5	2	0	0	1	1	1	1	8.1	0.03	28.4
1	0	0	100	4.81	1	68	27.7	126	81.5	212	65	147	13.7	5.3	84.0	2	1	0	0	1	1	1	18.1	0.07	17.5
1	0	0	100	6.84	1	78	24.8	111	68.5	180	65	115	15.9	5.7	66.3	2	1	0	0	1	1	1	7.9	0.02	34.3
1	0	0	100	6.8	1	66	27.8	167.5	103	220	65	155	14.8	5.2	72.1	2	1	0	0	0	1	1	15	0.02	41.3
1	1	0	100	6.96	1	68	23.8	158	86	194	65	129	16.6	4.6	74.6	1	0	0	0	1	1	1	18.8	0.07	18.1
1	0	0	100	7	2	69	27.8	132.5	74.5	222	65	157	13.2	5.3	75.9	2	1	1	1	0	1	1	10.5	0.17	2.46
1	0	0	100	6.88	2	71	27.1	141.5	79.5	175	65	110	12.2	5.3	71.1	2	1	1	0	1	1	1	17.7	0.1	67.3
1	0	0	100	6.86	2	76	27.6	109	65	149	65	84	12.9	5.1	58.4	2	1	1	1	1	1	1	9.6	0.1	56.6
1	0	0	100	4.64	2	72	29	126.5	75	189	65	124	13.4	4.5	60.1	1	1	0	1	1	1	1	9.4	0.32	9.9
1	0	0	100	6.87	2	74	30.6	145	77.5	191	65	126	13.2	6.4	59.2	2	1	1	0	1	1	1	244.1	0.12	23.2
1	0	0	100	6.8	2	68	20.7	165.5	90.5	237	65	172	14.7	5.4	81.1	2	1	1	0	0	1	1	238.1	0.1	9.83
1	0	0	100	5.05	2	75	25.9	165	101	223	65	158	14.6	5.7	51.0	2	1	1	0	0	1	1	39.6	0.29	15.6
1	0	0	100	5.05	2	68	29.3	133.5	68	221	65	156	14.7	5.2	76.4	2	1	1	1	0	1	1	41	0.1	5.33
1	0	0	100	6.87	2	76	18.7	104.5	59.5	190	65	125	14	5.6	72.2	1	1	1	1	1	1	1	19.1	0.24	18.9
1	0	0	100	5.04	2	69	26	123.5	63.5	215	65	150	12.2	6.6	75.9	2	1	1	0	1	0	1	31	0.44	38.8
1	0	0	100	7	2	67	18.9	112	66	184	65	119	12.7	5.3	81.7	2	1	1	1	1	1	1	16.6	0.01	42.9
1	0	0	100	7	2	74	22.1	142	78	239	65	174	13.5	5	69.6	2	1	1	0	0	1	1	20	0.02	9.01

1	0	0	100	7.07	2	70	22.3	142	67	220	65	155	14.4	6.1	71.6	2	1	0	0	0	1	1	48.4	0.04	95.3
1	0	0	100	7.23	2	72	21.5	129.5	82	255	65	190	13.5	5.2	52.1	1	1	1	1	0	1	1	9.2	0.01	32.2
1	0	0	100	5.16	2	65	23.6	124.5	74	235	65	170	13.2	5.9	54.7	2	1	0	1	0	1	1	31.4	0.01	40.4
1	0	0	100	7.02	2	68	19.1	112.5	66	219	65	154	12.2	5.5	81.1	2	1	1	1	1	1	1	20.5	0.04	13.2
1	0	0	100	7.04	2	69	18.2	110	69	205	65	140	11.2	5.1	72.1	2	1	1	1	1	1	1	7.1	0.04	0.05
1	0	0	100	5.06	2	71	28.9	118	72.5	197	65	132	13.8	5.3	74.8	1	1	1	0	0	1	1	8.2	0.03	28.9
1	0	0	100	5.06	2	69	24	130.5	76	172	65	107	12.4	6.1	75.9	2	1	1	0	1	1	1	37.3	0.06	12.3
1	0	0	100	5.08	2	73	23.8	121	63	179	65	114	11.9	5.5	70.1	1	1	1	1	1	1	1	17.3	0.01	49.5
1	0	0	100	4.63	2	79	20.6	96.5	57.5	164	65	99	11.9	5.4	67.2	1	1	1	1	1	1	1	29	0.03	32.5
1	0	0	100	4.64	2	71	24.4	131.5	77	225	65	160	13	6.3	71.1	1	1	1	1	0	1	1	38.2	0.03	16.4
1	0	0	100	6.97	2	69	25.2	136.5	87	205	65	140	14.4	5.4	72.1	2	1	1	1	0	1	1	43.1	0.02	0.49
1	0	0	100	6.8	2	75	18.7	152	83	193	65	128	15.3	5.2	72.7	2	1	1	0	1	1	1	15.5	0.02	9.34
1	0	0	100	6.82	2	70	24.9	113	59	217	65	152	12.5	6	75.3	2	1	1	1	1	1	1	8.2	0.03	20.1
1	0	0	100	6.84	2	74	20.2	116.5	74.5	234	65	169	12.9	4.8	73.2	2	1	1	1	0	1	1	14.3	0.01	31.6
1	0	0	100	5.04	2	70	22.5	107.5	66.5	165	65	100	12.3	6.1	71.6	2	1	1	1	1	1	1	8.5	0.01	20
1	0	0	100	5.04	2	72	30.8	141	79	203	65	138	13.7	5.5	70.6	1	1	1	0	1	1	1	4.8	0.03	31.3
1	1	0	100	6.85	2	75	26	120.5	74	223	65	158	12.1	5.6	58.8	2	1	1	1	0	1	1	31.6	0.1	50.1
1	1	0	100	5.37	2	69	21.8	112.5	73.5	174	65	109	13.2	5.8	75.9	2	1	0	1	1	1	1	11.8	0.02	23.6
1	0	0	100	5.36	2	80	21.5	106.5	59	173	65	108	12.3	5.8	70.2	1	1	1	1	1	1	1	48.8	0.12	37.3
1	0	0	100	5.07	2	80	24.6	118.5	72.5	178	65	113	13.6	5.5	66.7	1	1	1	0	1	1	1	5.1	0.03	22
1	1	0	100	4.64	2	80	19.9	136	83	201	65	136	13	5.5	74.6	1	1	1	0	1	1	1	55.1	0.05	15.5
1	0	1	3.4	3.4	1	75	17.4	102	63	191	65	126	12.1	4.9	53.1	2	1	0	0	1	1	1	111.2	0.03	8.01
1	0	1	3.7	3.7	1	69	23.1	156.5	87	139	65	74	13.8	5.6	78.3	1	1	0	0	1	1	1	11.1	0.07	38.7
1	1	1	4.7	4.7	1	70	21	131	70	209	65	144	14.1	5.6	77.7	2	0	0	1	1	1	1	10	0.11	5.69
1	1	1	1.9	1.9	1	68	19.9	128.5	77.5	188	65	123	15.5	5.5	74.6	2	1	0	1	1	1	1	8.8	0.26	88.9
1	1	1	6.3	6.3	1	70	24.4	124.5	73.5	209	65	144	15.4	5.6	61.7	2	0	0	1	1	1	1	11	0.06	13.1
1	0	1	1.6	1.6	1	84	19.8	121	72	172	65	107	14.7	6	66.7	2	1	1	1	1	0	1	11.6	0.2	71
1	1	1	0.4	0.4	2	71	20.1	120	72	189	65	124	12.5	5.4	74.8	1	1	1	1	1	1	1	115.8	0.03	175
1	0	0	100	7	1	65	22.6	132.5	82.5	208	66	142	14.1	5.9	76.2	2	0	0	1	1	1	1	30.5	0.16	11.4
1	0	0	100	4.58	1	73	20.5	153.5	92	184	66	118	16.3	5.5	68.6	2	1	0	0	1	1	1	6.6	0.1	66.1
1	0	0	100	6.87	1	67	23.9	136	75.5	161	66	95	13.3	5.3	63.0	2	1	1	0	1	1	1	3.9	0.14	25.2
1	0	0	100	6.98	1	71	20.2	149	81	175	66	109	14.9	6.1	73.1	2	1	0	0	1	1	1	4.3	0.13	60.4
1	0	0	100	6.98	1	71	26.6	125	74	172	66	106	13.4	5.3	73.1	2	1	0	0	1	1	1	2.9	0.14	18.4
1	0	0	100	6.96	1	65	28.3	163.5	92.5	198	66	132	16.2	10.2	80.5	2	1	0	0	1	0	1	1169.7	0.27	13.3
1	0	0	100	4.67	1	66	26.4	156.5	83.5	183	66	117	16.2	5.1	79.9	2	0	0	0	1	1	1	20	0.1	18.8
1	0	0	100	6.88	1	72	21.7	133	75	223	66	157	14.9	4.9	76.7	2	1	0	1	0	1	1	21	0.08	24.2
1	0	0	100	6.85	1	76	22.3	116	75.5	167	66	101	13.8	5.4	59.2	2	1	0	1	1	1	1	5.4	0.02	21.6
1	0	0	100	7.03	1	75	22.8	109.5	68.5	241	66	175	13.8	5.3	75.1	2	1	1	1	0	1	1	15.4	0.05	0.9
1	0	0	100	5.05	1	69	18.3	118.5	80.5	176	66	110	13.6	5.1	74.1	2	1	0	1	1	1	1	31.6	0.01	14
1	0	0	100	5.06	1	66	27.6	129	89	180	66	114	14.3	5.8	79.9	2	1	1	1	1	1	1	34.8	0.09	15.2
1	0	0	100	7	1	72	23.6	123.5	74.5	179	66	113	14.1	5.1	72.6	2	1	1	1	1	1	1	8.1	0.03	30.7
1	0	0	100	7	1	71	23.6	163.5	93.5	232	66	166	13.3	5	69.6	2	1	0	0	0	1	1	9.4	0.02	54.3
1	0	0	100	6.87	1	75	22.1	110.5	67	194	66	128	14.7	5.9	71.0	2	1	1	1	1	1	1	94.2	0.03	16
1	0	0	100	6.87	1	70	23.7	115	68.5	212	66	146	14.6	5.2	73.6	2	1	0	1	1	1	1	6.7	0.03	18.6
1	0	0	100	6.87	1	66	25.9	107.5	56	213	66	147	14.9	5.6	75.7	2	0	0	1	1	1	1	12.8	0.07	10.4
1	0	0	100	6.95	1	71	22.1	101.5	67.5	232	66	166	10.5	5	73.1	2	1	0	1	0	1	1	28.2	0.04	66.6
1	0	0	100	6.97	1	72	22.3	136	70.5	195	66	129	14.9	5.1	69.1	2	1	0	1	1	1	1	4.8	0.05	145
1	0	0	100	6.83	1	72	20.8	161.5	93	222	66	156	14.6	5.5	72.6	2	1	1	0	0	1	1	11.6	0.03	42.8
1	0	0	100	4.69	1	66	24.9	153.5	89	249	66	183	17.7	5.3	79.9	2	1	1	0	0	1	1	33.3	0.02	5.67
1	0	0	100	5.37	1	65	20.2	131	81.5	192	66	126	15.5	5.4	76.2	2	1	0	0	1	1	1	11.7	0.02	2.46
1	0	0	100	2.12	1	71	22.2	118.5	73.5	157	66	91	12.8	5.4	82.2	2	1	0	1	1	1	1	4.1	0.02	27.5

1	0	0	100	5.06	1	72	28.5	129.5	75	190	66	124	14.8	5.1	72.6	2	1	0	0	1	1	1	3.4	0.06	0.9
1	0	0	100	6.85	1	72	21.7	123.5	73	173	66	107	13.9	5.1	69.1	2	1	0	1	1	1	1	16.5	0.01	31.7
1	1	0	100	0.86	1	73	20.1	123	64.5	213	66	147	12	5.6	60.4	2	1	1	1	1	1	1	3.9	0.2	91.3
1	1	0	100	1.77	1	79	20.3	121.5	71	167	66	101	13.2	5	83.8	2	0	0	1	1	1	1	18.2	0.02	36.5
1	1	0	100	4.62	1	70	21.4	159	80.5	238	66	172	15.2	5.3	77.7	2	0	0	0	0	0	1	6.2	0.03	9.25
1	0	0	100	7.01	2	78	23.6	127	74.5	245	66	179	13.4	5.1	71.2	1	1	1	0	0	1	1	49.8	0.1	19.8
1	0	0	100	6.81	2	65	19	146	81	147	66	81	11.5	5.4	82.8	2	1	1	0	1	1	1	45.2	0.28	67.2
1	0	0	100	5.06	2	70	26.1	135	69	162	66	96	12.8	5.2	80.0	1	1	1	1	1	1	1	20.4	0.11	30.8
1	0	0	100	4.64	2	66	27.8	125	70.5	244	66	178	13.1	5.8	73.6	2	1	0	1	0	1	1	10.8	0.15	35.5
1	0	0	100	6.8	2	70	19.6	122.5	68	186	66	120	13.8	5.7	60.9	2	1	1	1	0	1	1	10.1	0.34	11.1
1	0	0	100	6.8	2	75	26.2	154.5	81.5	171	66	105	13.5	5.9	72.7	2	1	0	0	1	1	1	15.4	0.16	32.8
1	0	0	100	4.67	2	65	26.6	155	85.5	196	66	130	12.7	5.1	78.0	2	1	1	0	1	1	1	209	0.19	62.8
1	0	0	100	5.36	2	66	24	154	92.5	197	66	131	13.3	5.4	73.6	2	1	1	0	1	1	1	19.3	0.29	26.9
1	0	0	100	5.04	2	77	23.5	149	76.5	171	66	105	13.8	5.2	76.1	1	1	1	0	1	1	1	33.1	0.18	115
1	0	0	100	7.06	2	65	25.4	156	80	226	66	160	13.1	5	78.0	2	1	0	0	0	1	1	11.1	0.02	44.5
1	0	0	100	7.07	2	74	25.7	160.5	86.5	188	66	122	12.3	5.5	77.8	1	1	1	0	1	1	1	16.6	0.05	0.05
1	0	0	100	7.08	2	67	26.7	129.5	84	227	66	161	12.9	5.4	76.9	2	1	1	1	0	1	1	23	0.01	44.4
1	0	0	100	4.58	2	71	21.2	128	66	239	66	173	12.4	5.4	60.5	1	1	1	1	0	1	1	27.7	0.07	18
1	0	0	100	4.58	2	67	24.8	102	61.5	256	66	190	12.4	5.7	76.9	2	1	1	1	0	1	1	8.1	0.02	9.03
1	0	0	100	6.86	2	69	24.5	120	66	225	66	159	13.3	5.2	72.1	2	1	1	1	0	1	1	5.5	0.05	43.8
1	0	0	100	6.88	2	69	20.3	137	82.5	183	66	117	13.1	5.4	75.9	2	1	1	0	1	1	1	31.3	0.01	24.3
1	0	0	100	5.16	2	67	23.4	123	88	232	66	166	13.5	5.7	73.1	2	1	1	1	0	1	1	12.5	0.02	8.27
1	0	0	100	6.84	2	72	24.1	92.5	53.5	213	66	147	12.1	6.1	78.9	2	1	1	1	1	1	1	26	0.03	15.1
1	0	0	100	6.84	2	71	28.4	101	69	208	66	142	13.6	5.7	74.8	2	1	1	1	0	1	1	4.1	0.02	30.2
1	0	0	100	6.85	2	68	26.5	128	75	213	66	147	12.5	5.6	81.1	2	1	1	0	1	1	1	155.6	0.05	22.5
1	0	0	100	6.86	2	67	28.6	124	69.5	177	66	111	13.6	5.4	81.7	1	0	1	1	1	1	1	10.2	0.09	10.1
1	0	0	100	7.04	2	71	24.5	163.5	82	198	66	132	14.1	5.5	74.8	2	1	1	0	1	1	1	13.8	0.08	13.8
1	0	0	100	6.99	2	68	25.1	112.5	70.5	219	66	153	14	5.2	76.4	2	1	1	1	1	1	1	15.5	0.04	4.66
1	0	0	100	4.64	2	68	21.5	140.5	66	166	66	100	13.1	5.6	81.1	2	1	1	0	1	1	1	76.6	0.04	33.9
1	0	0	100	6.96	2	72	26.1	133.5	77.5	214	66	148	13.5	5	74.3	1	1	0	0	1	1	1	55.1	0.04	46.6
1	0	0	100	6.97	2	69	20.9	120	72	242	66	176	13.2	5.3	75.9	1	1	1	1	0	1	1	80.5	0.03	46.8
1	0	0	100	6.97	2	65	21.1	102.5	59	206	66	140	13.3	5	74.2	2	1	1	1	1	1	1	38.2	0.01	2.44
1	0	0	100	6.98	2	69	22.9	147.5	72	248	66	182	14.2	5.2	75.9	1	1	1	0	0	1	1	18.1	0.02	23.3
1	0	0	100	6.8	2	68	22	150	79.5	251	66	185	14.1	5.5	87.3	1	1	1	0	0	1	1	28.2	0.07	21.8
1	0	0	100	6.8	2	68	19.6	111	71.5	235	66	169	12.7	5.5	61.8	2	1	1	1	0	1	1	8.2	0.03	6.26
1	0	0	100	6.8	2	75	24	123	78.5	189	66	123	12.1	5.8	69.1	2	1	1	1	1	1	1	17	0.01	57.4
1	0	0	100	6.83	2	70	19.7	138	82.5	188	66	122	13.8	5.1	80.0	2	1	1	1	1	1	1	11.8	0.01	21.3
1	0	0	100	4.59	2	67	23.4	137	80.5	214	66	148	14.7	5.5	81.7	2	1	1	1	1	1	1	95.8	0.08	16.4
1	0	0	100	4.6	2	67	25.3	144	86.5	191	66	125	13.7	5.3	62.2	1	1	1	0	1	1	1	12.1	0.05	19.2
1	0	0	100	1.88	2	66	23.3	125	82	210	66	144	12.6	5.4	77.5	2	1	1	1	1	1	1	44.1	0.01	21.9
1	0	0	100	4.62	2	70	25.8	124	77	223	66	157	12	5.8	80.0	1	1	1	0	0	1	1	4	0.07	26.6
1	0	0	100	4.62	2	70	20.8	133.5	77.5	225	66	159	12.8	5.5	71.6	2	1	1	1	0	1	1	16.8	0.06	60.4
1	0	0	100	4.67	2	71	20.3	121.5	79	184	66	118	14.6	5.7	74.8	1	1	1	1	1	1	1	44	0.03	37.1
1	0	0	100	5.37	2	72	22.8	117	62.5	246	66	180	12.7	5.5	52.1	1	1	1	1	0	1	1	3.6	0.05	13
1	0	0	100	6.86	2	67	20.2	127.5	64	229	66	163	13.3	5.4	73.1	2	1	1	1	0	1	1	285.9	0.02	26.1
1	0	0	100	6.86	2	70	27.4	119.5	69.5	182	66	116	13.2	5	80.0	2	1	0	0	1	1	1	12.5	0.07	21.6
1	0	0	100	6.87	2	75	24.3	104.5	65.5	206	66	140	12.4	5.4	72.7	1	1	0	0	0	1	1	29.9	0.05	26.9
1	0	0	100	6.87	2	78	24.3	116	67	183	66	117	13.9	6.1	75.6	2	1	1	1	1	1	1	10.2	0.06	45
1	0	0	100	5.04	2	79	30.4	109.5	54	233	66	167	14.6	5.6	70.7	1	1	1	1	0	1	1	15.9	0.02	31.2
1	1	0	100	7.02	2	71	20	117	73	184	66	118	13.3	5.2	85.5	1	1	1	1	1	1	1	32.1	1.9	28.1
1	1	0	100	5.16	2	79	22.6	110	69	241	66	175	13.1	5.6	57.2	2	1	1	1	0	1	1	22.1	0.08	25.9

1	1	0	100	6.7	2	72	25.1	151.5	102.5	201	66	135	14.4	5.3	78.9	2	1	1	0	1	1	1	34.2	0.02	30.6
1	1	0	100	6.98	2	78	20.7	145	84	229	66	163	13.7	6.5	71.2	1	1	1	0	0	0	1	24	0.02	9.02
1	1	0	100	5.08	2	73	21.4	120.5	70	189	66	123	13.3	5.5	78.3	2	1	0	1	1	1	1	6.2	0.01	10.7
1	0	0	100	6.87	1	68	23.1	111	70.5	173	66	107	14	6.1	74.6	2	0	0	0	1	1	1	7.7	0.56	31.6
1	0	0	100	6.84	1	79	30.7	115.5	66.5	140	66	74	13.2	6.8	69.1	2	1	0	0	1	0	1	55.9	0.1	33.6
1	0	0	100	4.69	1	74	23.2	129	76.5	169	66	103	13.6	6.3	71.5	2	1	0	0	1	0	1	10.7	0.17	22.7
1	0	0	100	7	1	72	18.1	105	55.5	152	66	86	13.2	5.2	76.7	2	1	1	0	1	1	1	14.9	0.04	61.8
1	0	0	100	5.04	1	79	20.6	118.5	74.5	170	66	104	13.4	6	73.0	2	1	0	0	1	1	1	26.5	0.04	46.7
1	0	0	100	6.98	2	72	26.7	142.5	88	179	66	113	13.1	7	74.3	1	1	1	0	1	0	1	15.2	0.1	0.05
1	0	0	100	6.98	2	65	31.5	117	67	186	66	120	14	5.7	78.0	1	1	1	1	1	1	1	9.2	0.11	20.1
1	0	0	100	6.95	2	71	23.1	122.5	78	196	66	130	12.3	5.4	52.5	2	1	1	1	1	1	1	8.6	0.1	151
1	0	0	100	7.06	2	75	22	172.5	91	197	66	131	13.9	5.2	69.1	2	1	1	0	1	1	1	9.2	0.04	33.2
1	0	0	100	7.07	2	69	23.5	127.5	71	201	66	135	13.7	6	72.1	2	1	1	1	0	1	1	15.1	0.07	16.2
1	0	0	100	4.58	2	73	22.3	145	86.5	192	66	126	13.4	5.5	73.8	2	1	1	0	1	1	1	58.5	0.04	38.1
1	0	0	100	5.16	2	73	21	138	79	243	66	177	12.9	5.8	78.3	2	1	1	0	0	1	1	37.4	0.01	36.7
1	0	0	100	6.85	2	66	26.4	125	78.5	186	66	120	14.4	5.2	73.6	2	1	1	0	1	1	1	15.9	0.03	6.3
1	0	0	100	6.85	2	76	24.5	133	72	218	66	152	12.9	8.3	76.7	2	1	1	1	1	0	1	32.6	0.06	29.2
1	0	0	100	7.01	2	74	28.6	116	70.5	191	66	125	12.6	5.4	77.8	1	1	1	0	1	1	1	36.7	0.09	31.2
1	0	0	100	5.05	2	65	23.1	126	71.5	200	66	134	12.8	5.4	82.8	2	1	1	1	1	1	1	8.2	0.01	5.3
1	0	0	100	2.45	2	70	19.8	130	73	227	66	161	11.4	6.4	80.0	2	1	1	1	0	0	1	24	0.01	0.14
1	0	0	100	4.63	2	74	24	130	87	155	66	89	11.7	5	59.2	2	1	1	0	1	1	1	68.4	0.07	48.7
1	0	0	100	6.95	2	67	24.4	138	78	171	66	105	13.8	5.9	54.0	1	1	1	1	0	1	1	10.7	0.06	11.9
1	0	0	100	6.96	2	68	23.9	129.5	67.5	155	66	89	13.3	4.2	72.6	2	1	1	0	1	1	1	51	0.03	0.05
1	0	0	100	6.84	2	78	25.1	161.5	83	230	66	164	10.4	5.4	44.0	1	1	1	0	0	1	1	1138.6	0.01	52.6
1	0	0	100	4.6	2	74	20.2	136.5	68	208	66	142	13.9	4.9	77.8	2	1	1	0	0	1	1	10.3	0.01	49.2
1	0	0	100	4.62	2	66	30	153.5	83	218	66	152	13.4	6	77.5	2	1	1	0	1	1	1	30.2	0.02	53.2
1	0	0	100	6.79	2	70	19.4	119	75.5	235	66	169	13.6	5.6	60.9	2	1	1	1	0	1	1	6.8	0.01	13.6
1	0	0	100	4.69	2	78	28.2	133.5	85	187	66	121	14.1	5.7	71.2	1	1	1	0	1	1	1	97	0.05	54.1
1	0	0	100	5.05	2	70	27	125	70.5	268	66	202	14.9	6.5	71.6	2	1	1	1	0	0	1	14	0.02	8.17
1	0	0	100	5.04	2	76	24.1	103.5	52	245	66	179	12	5.5	44.6	1	1	1	1	0	1	1	14.7	0.05	50.9
1	0	0	100	5.04	2	68	28	105	67	191	66	125	12.6	5.4	61.8	2	1	1	1	0	1	1	12	0.03	6.15
1	1	0	100	7.07	2	74	26.2	115.5	64	171	66	105	13.8	5.5	69.6	1	1	1	1	1	1	1	10.1	0.03	76.7
1	1	0	100	5.15	2	70	26	95.5	56	221	66	155	12.7	5.4	71.6	2	1	1	0	0	1	1	20.2	0.02	8.15
1	0	1	2.5	2.5	1	72	22.3	134.5	49	166	66	100	15.1	5.3	76.7	2	0	0	0	1	1	1	640.1	0.04	430
1	1	1	4.3	4.3	1	66	25.6	123	82.5	192	66	126	14	5.4	75.7	2	1	0	0	1	1	1	43.3	0.43	37
1	1	1	5.5	5.5	1	70	21.5	134.5	76.5	146	66	80	12.6	5.2	77.7	1	1	0	1	1	1	0	15.9	0.02	153
1	1	1	3.9	3.9	2	67	22.6	114	72	207	66	141	15.1	5.2	73.1	2	1	1	1	1	1	1	2.8	0.04	51.5
1	1	1	0.8	0.8	2	80	23.7	148	84	203	66	137	13.6	5.6	74.6	1	1	0	0	1	1	1	16.7	0.06	81.3
1	1	1	1.3	1.3	1	74	21.5	106.5	60.5	171	66	105	13.2	5.4	71.5	2	1	1	1	1	1	1	13.2	0.04	297
1	1	1	6.3	6.3	2	74	23.5	137.5	65.5	204	66	138	11.8	5.4	73.2	1	1	1	0	1	1	1	42.9	0.01	97.7
1	0	1	3.2	3.2	2	81	24.2	135.5	72.5	211	66	145	13.5	8.2	69.7	2	1	1	0	1	0	1	30.9	0.02	90.5
1	1	1	1.2	1.2	2	84	20	165.5	90	189	66	123	13	4.5	68.3	1	1	1	0	1	1	1	540.5	0.02	141
1	0	0	100	6.87	1	69	24.2	126	82	244	67	177	13.3	5.1	74.1	2	1	1	0	0	1	1	3.9	0.1	49
1	0	0	100	5.15	1	72	25.3	142.5	76	188	67	121	13.1	6	72.6	1	1	0	0	1	1	1	4.4	0.14	0.29
1	0	0	100	5.05	1	73	23.1	143.5	73	199	67	132	15.4	7.6	72.0	2	1	0	0	1	0	1	26.8	0.1	4.5
1	0	0	100	6.97	1	70	22.9	117.5	65	170	67	103	15.3	8.3	77.7	1	1	1	1	1	0	0	8.5	0.24	22.6
1	0	0	100	6.98	1	69	25.6	121.5	65	199	67	132	15.7	6.1	74.1	1	0	0	1	1	1	1	8.4	0.2	11.4
1	0	0	100	3.7	1	72	22.6	117.5	74.5	199	67	132	14.1	5	72.6	2	1	0	1	1	1	1	18.3	0.15	43.4
1	0	0	100	6.87	1	70	29.9	129	72	174	67	107	14.2	5.2	73.6	1	1	0	0	1	1	0	24.5	0.1	237
1	0	0	100	4.6	1	76	25.9	167.5	82	199	67	132	15	5.1	52.7	2	1	1	0	1	1	1	31.6	0.26	29.5
1	0	0	100	1.28	1	73	19.2	117	70.5	143	67	76	15.4	5.1	60.4	2	0	0	0	1	1	1	4.3	0.57	65.3

1	0	0	100	2.68	1	68	23.5	109.5	64	151	67	84	14.2	5.5	74.6	2	1	0	1	1	1	1	16.1	0.13	64.3
1	0	0	100	0.97	1	70	24.6	134.5	86	171	67	104	15.2	5.5	77.7	2	1	1	0	1	1	1	7.2	0.14	14.6
1	0	0	100	6.87	1	67	25.8	145	87	208	67	141	15	5.4	79.4	2	1	0	0	1	1	1	7.3	0.48	17.7
1	0	0	100	7.06	1	66	21.5	130.5	75.5	202	67	135	14.3	5.3	79.9	2	1	0	1	1	1	1	10.9	0.02	23.3
1	0	0	100	7.08	1	67	27.1	130.5	89.5	165	67	98	16.9	6.2	91.2	2	1	0	0	0	1	1	30.4	0.02	8.95
1	0	0	100	4.58	1	71	23.3	141	89.5	198	67	131	13.3	5.5	69.6	2	1	1	0	1	1	1	17.9	0.02	32.1
1	0	0	100	6.86	1	67	27.1	165	93	194	67	127	14.9	5.5	71.6	2	1	0	0	1	1	1	365.3	0.02	14
1	0	0	100	6.86	1	68	21.7	163.5	93	160	67	93	13.1	4.7	84.0	2	0	0	0	1	1	1	5.6	0.08	23.9
1	0	0	100	7.04	1	69	22.8	163	89	231	67	164	15.4	7.1	70.6	2	1	0	0	0	0	0	8.3	0.05	47.2
1	0	0	100	7.04	1	77	22.5	111.5	70.5	247	67	180	14.3	4.8	70.1	2	1	1	1	0	1	1	5.7	0.03	0.05
1	0	0	100	5.06	1	73	22.5	128.5	75.5	196	67	129	16.3	6.6	72.0	1	1	0	1	1	0	1	14.6	0.07	16.6
1	0	0	100	6.99	1	70	23.1	114	66.5	175	67	108	13.2	5.4	82.8	2	1	0	1	1	1	1	56.2	0.06	42
1	0	0	100	7	1	67	21.8	110	63	203	67	136	15.2	4.5	84.6	2	1	0	1	1	1	1	18.8	0.02	17.9
1	0	0	100	4.63	1	68	20.6	144	83.5	200	67	133	13.8	6.2	78.8	2	1	0	0	1	1	1	14	0.04	13.9
1	0	0	100	6.87	1	66	21.9	131	78	170	67	103	15.1	5.2	79.9	2	1	0	1	1	1	1	27.3	0.03	16.8
1	0	0	100	6.79	1	65	24.2	158	90	247	67	180	14.9	6.4	72.6	2	1	0	0	0	0	0	10.6	0.03	92.7
1	0	0	100	6.8	1	68	22.3	138.5	78	192	67	125	14.6	6.7	84.0	2	1	0	1	1	0	1	32.2	0.03	22.3
1	0	0	100	6.83	1	70	19.2	131.5	75	194	67	127	13.9	5.8	73.6	2	1	0	0	1	1	0	17.3	0.07	129
1	0	0	100	4.59	1	69	25.3	150	83	231	67	164	14	5.6	74.1	2	1	0	0	0	1	1	3	0.04	6.82
1	0	0	100	4.61	1	70	23.6	115.5	74	206	67	139	14.4	5.1	73.6	2	1	0	1	1	1	1	5	0.03	22.4
1	0	0	100	4.62	1	73	24.1	159.5	77.5	244	67	177	14.9	8	81.1	2	1	0	0	0	0	1	83	0.03	19.8
1	0	0	100	4.62	1	66	29.5	160	83.5	196	67	129	15.4	5.8	63.5	2	1	0	0	1	1	1	1476.6	0.06	17.8
1	0	0	100	4.62	1	65	20.6	159.5	90.5	238	67	171	15	5.5	76.2	2	1	1	0	0	1	1	28.2	0.01	9.56
1	0	0	100	6.44	1	72	26.3	146	88	147	67	80	15.3	6.3	60.9	2	1	0	0	1	1	1	40.1	0.05	10.5
1	0	0	100	5.06	1	73	22.9	120	72.5	198	67	131	13.7	5.9	72.0	2	1	0	0	1	1	1	89.3	0.06	23.4
1	0	0	100	6.85	1	73	18.3	103	58	175	67	108	13.3	5.4	68.6	2	1	0	1	1	1	0	8.1	0.01	112
1	0	0	100	6.86	1	65	20.5	97	53	170	67	103	14.3	4.8	72.6	2	0	0	1	1	1	1	5.5	0.01	19
1	0	0	100	5.04	1	75	29.3	141.5	76.5	176	67	109	14.6	5.7	67.7	2	1	0	0	1	1	1	16.8	0.06	7.41
1	1	0	100	4.79	1	72	17.8	91	56.5	181	67	114	13.4	5.1	69.1	2	1	0	1	1	1	1	7.6	0.03	17.6
1	0	0	100	4.59	2	69	23.3	146	82	176	67	109	13.9	5.8	61.4	2	1	1	0	1	0	1	44.5	0.21	28.9
1	0	0	100	7.23	2	66	23.1	107.5	62	215	67	148	11.9	5.5	77.5	2	1	1	1	1	1	1	11.1	0.18	21.4
1	0	0	100	5.15	2	66	26	133.5	78.5	236	67	169	11.8	5.4	73.6	2	1	1	0	0	1	1	10.2	0.11	0.81
1	0	0	100	7.21	2	69	23.9	103	60	251	67	184	13	5.2	75.9	1	1	1	1	0	1	1	22.3	0.19	10.8
1	0	0	100	6.86	2	73	26.7	117	76.5	202	67	135	12.7	5	59.7	2	1	1	1	1	1	1	9.5	0.55	18.1
1	0	0	100	6.87	2	66	21.3	126	84	280	67	213	13.3	5.6	73.6	2	1	1	1	0	1	1	52	0.1	8.65
1	0	0	100	7	2	68	21.7	114	68	238	67	171	12	5.7	72.6	1	1	1	1	0	1	1	11.4	0.01	25.2
1	0	0	100	7	2	65	26.2	141	82.5	257	67	190	14.9	5.2	82.8	2	1	1	0	0	1	1	113.5	0.04	9.66
1	0	0	100	7.08	2	65	22.6	120	66.5	219	67	152	13.5	5.6	78.0	2	1	1	1	0	1	1	8.8	0.03	9
1	0	0	100	6.86	2	65	22.1	126	71.5	201	67	134	12.8	4.9	74.2	2	1	0	1	1	1	1	11.2	0.04	16.1
1	0	0	100	6.87	2	78	25.8	128	66.5	221	67	154	11.9	5.3	67.7	1	1	0	1	0	1	1	17.7	0.06	8.11
1	0	0	100	7.23	2	76	27.4	114	57.5	201	67	134	12.5	5	44.6	1	1	1	0	1	1	1	10.5	0.02	24.4
1	0	0	100	5.15	2	68	27.6	106	63.5	272	67	205	12.3	5.5	72.6	2	1	1	1	0	1	1	24.7	0.05	1.38
1	0	0	100	6.85	2	77	17.3	93	52	156	67	89	12.1	5	81.9	2	1	1	1	1	1	1	15.6	0.01	17.1
1	0	0	100	6.85	2	76	24.3	101	61	205	67	138	13	8.1	72.2	1	1	1	0	1	0	1	20	0.06	36.5
1	0	0	100	6.86	2	66	22.1	103	58.5	202	67	135	12.6	5.1	77.5	2	1	0	1	1	1	1	9.9	0.05	16.4
1	0	0	100	7.01	2	67	26.4	114	66.5	214	67	147	12.6	5.3	76.9	2	1	1	1	1	1	1	28.2	0.01	16.8
1	0	0	100	7.02	2	69	22.1	155.5	82	200	67	133	11.5	5.5	86.7	1	1	1	0	1	1	1	31.5	0.02	16.3
1	0	0	100	7.04	2	72	25.9	147.5	79.5	228	67	161	13.5	5.7	74.3	2	1	0	0	0	1	1	6.2	0.06	1.06
1	0	0	100	5.05	2	68	20.3	86	42	151	67	84	12.6	5.3	76.4	2	1	1	1	1	1	1	56	0.01	49.7
1	0	0	100	5.06	2	67	24.5	129.5	67.5	207	67	140	11.9	5.8	73.1	2	1	1	1	1	1	1	6.8	0.05	32.1
1	0	0	100	5.07	2	72	26.3	111.5	66	194	67	127	12.1	5.1	70.6	2	1	1	0	1	1	1	3.9	0.01	21.6

1	0	0	100	6.98	2	66	24.6	141	82	249	67	182	13.4	6.1	82.3	1	1	1	0	0	1	1	21.6	0.06	17.7
1	0	0	100	1.79	2	71	18.5	131.5	78	175	67	108	11.7	5.2	85.5	2	1	1	1	1	1	1	42.9	0.03	47.9
1	0	0	100	7	2	66	23.7	150.5	83	186	67	119	12.3	5.1	82.3	2	1	1	0	1	1	1	34.5	0.01	28.3
1	0	0	100	4.63	2	71	18.1	107.5	69	203	67	136	9.5	5.7	74.8	2	1	1	1	1	1	1	18.6	0.01	10.9
1	0	0	100	4.63	2	69	23	130	61.5	208	67	141	14.1	5.4	75.9	2	1	1	0	1	1	1	34.5	0.09	13.9
1	0	0	100	4.64	2	71	20.3	102.5	53.5	194	67	127	13	5.3	60.5	2	1	1	1	1	1	1	17.8	0.03	8.91
1	0	0	100	6.87	2	67	20.1	117	75.5	239	67	172	13.2	5.6	81.7	2	1	0	1	0	1	1	9.9	0.02	9.33
1	0	0	100	6.95	2	65	27.1	134	75	194	67	127	13.7	5.1	82.8	2	1	1	0	1	1	1	26	0.06	24.5
1	0	0	100	6.96	2	68	27	123.5	78.5	172	67	105	12.4	5	76.4	1	1	0	0	1	1	1	6.1	0.02	5.39
1	0	0	100	4.59	2	65	25	132	73	196	67	129	12.6	5.7	82.8	1	1	1	0	1	1	1	70.2	0.03	9.25
1	0	0	100	4.59	2	69	23.1	88.5	50.5	207	67	140	13.2	5.4	80.5	2	1	1	1	1	1	1	6.9	0.01	21.4
1	0	0	100	4.62	2	69	22.6	106.5	66	206	67	139	14	5.6	61.4	2	1	0	1	1	1	1	8.3	0.03	78
1	0	0	100	2.84	2	67	19.8	114.5	67	216	67	149	13.5	6	81.7	2	1	1	1	1	1	1	13.9	0.03	19.1
1	0	0	100	5.37	2	66	21.4	99.5	55	194	67	127	11.1	5.6	73.6	2	1	1	1	1	1	1	18.2	0.04	35.3
1	0	0	100	5.37	2	65	22.5	100	60.5	192	67	125	12.8	5.5	78.0	2	1	0	1	1	1	1	4.3	0.01	37.2
1	0	0	100	5.37	2	72	28.5	123.5	80.5	217	67	150	13.6	6	70.6	1	1	0	0	1	1	1	7.7	0.03	13.8
1	0	0	100	5.05	2	69	21.6	134	77	212	67	145	14.4	5.3	61.4	2	1	1	1	1	1	0	82.7	0.04	127
1	0	0	100	5.06	2	65	27.5	92	47.5	206	67	139	11.4	5.7	78.0	2	1	1	1	1	1	1	3.6	0.03	28.6
1	0	0	100	6.85	2	73	17.6	134	64	202	67	135	10.6	7.4	73.8	2	1	1	1	1	0	1	71.3	0.04	64.3
1	0	0	100	6.86	2	65	19.9	97	57.5	188	67	121	11.1	5.1	78.0	2	1	1	1	1	1	1	32.8	0.01	111
1	0	0	100	6.86	2	70	25.5	119	80	185	67	118	13.7	4.9	75.3	2	1	1	1	1	1	1	16.6	0.07	45.9
1	0	0	100	6.87	2	76	21	129.5	70	226	67	159	13.6	5	72.2	2	1	1	1	0	1	1	25.2	0.02	21.5
1	0	0	100	6.87	2	65	26.8	134.5	73	247	67	180	12.2	5.4	82.8	2	1	1	1	0	1	1	27.2	0.06	23.2
1	0	0	100	6.87	2	67	21.9	130.5	81	166	67	99	13.6	8.2	76.9	2	1	1	1	1	0	1	22.6	0.07	91.9
1	0	0	100	5.04	2	71	25.5	125.5	78	205	67	138	14.1	5.3	71.1	1	1	1	0	1	1	1	4.1	0.06	15.2
1	1	0	100	4.63	2	72	28.3	122.5	71.5	225	67	158	14.9	5.4	78.9	2	1	1	0	0	1	1	21.4	0.21	18.5
1	1	0	100	7.21	2	73	19.5	136	75.5	208	67	141	12.2	5.2	59.7	1	1	1	1	1	1	1	23	0.03	58
1	1	0	100	6.98	2	75	24.7	126.5	74	190	67	123	13.3	5	77.2	1	1	0	0	1	1	1	27.7	0.04	49.7
1	1	0	100	6.87	2	75	20.3	119.5	63	209	67	142	12.5	5.8	72.7	2	1	1	0	1	1	1	11.7	0.03	16.9
1	0	0	100	2.93	1	80	23.7	173.5	84	136	67	69	14.5	4.6	68.6	2	1	0	0	1	1	1	19.9	0.37	83.9
1	0	0	100	4.6	1	83	19.9	128.5	86.5	169	67	102	14.7	5.1	71.0	1	1	0	1	1	1	1	6.7	0.19	35.4
1	0	0	100	7.01	2	81	27.2	142	67	184	67	117	14.4	5.3	66.3	1	1	1	0	1	1	1	9.8	0.02	15.4
1	1	0	100	6.82	2	82	19.2	114.5	59	212	67	145	12.4	5.3	69.2	1	1	1	1	1	1	1	21.8	0.03	22.9
1	0	0	100	4.64	1	73	25	151	83	159	67	92	14.4	5.8	76.1	2	1	0	0	1	1	1	8.3	0.25	12.2
1	0	0	100	6.98	1	76	24.8	163.5	84	176	67	109	14.1	5	70.5	2	1	1	0	1	1	1	323.2	0.46	42
1	0	0	100	6.82	1	67	24.4	140.5	87	175	67	108	14	5	75.2	2	1	0	0	1	1	1	9	0.14	56
1	0	0	100	2.54	1	71	19.2	115	70.5	205	67	138	14.1	5.1	82.2	2	1	0	1	1	1	1	38.5	0.12	10.5
1	0	0	100	6.86	2	65	25.6	113.5	68.5	220	67	153	13.6	5.4	78.0	2	1	1	0	0	1	0	26.7	0.27	98.3
1	0	0	100	7.01	2	72	23.1	131	93	217	67	150	14.2	5.5	70.6	2	1	1	0	1	1	1	7.8	0.11	14.8
1	0	0	100	5.08	2	76	27.3	155.5	81	213	67	146	12	6.2	72.2	1	1	1	0	1	0	1	117.2	0.11	16.8
1	0	0	100	6.8	2	69	28	130.5	78.5	270	67	203	15.2	5.2	72.1	1	1	1	0	0	1	1	11.8	0.22	19.3
1	0	0	100	6.84	2	70	23.6	147.5	80	223	67	156	11.8	5.1	71.6	2	1	1	0	0	1	1	15.1	0.11	66.3
1	0	0	100	6.87	2	78	20.9	95	55.5	156	67	89	10.7	5.2	39.2	1	1	1	0	1	1	1	21.1	3.6	5.25
1	0	0	100	7.07	2	76	28.9	156	78	356	67	289	14.4	6.3	68.6	2	1	1	0	0	1	1	203.6	0.06	6.78
1	0	0	100	4.58	2	70	26.5	120.5	69.5	219	67	152	14.6	5.1	75.3	2	1	1	0	1	1	1	40.7	0.08	31.2
1	0	0	100	6.87	2	71	25.2	132.5	70	230	67	163	14	5.3	60.5	2	1	1	0	0	1	1	10.4	0.04	42.3
1	0	0	100	5.16	2	73	25.5	95	64	249	67	182	14.1	5.7	70.1	1	1	1	0	0	1	1	4.8	0.05	22.2
1	0	0	100	6.85	2	75	23.5	145	88	244	67	177	12.9	5.8	69.1	2	1	1	0	0	1	1	11.8	0.05	40.1
1	0	0	100	6.86	2	66	15.2	111.5	65	169	67	102	12.2	5.4	62.7	2	1	1	1	1	1	1	10.6	0.01	31.2
1	0	0	100	7.01	2	72	19.4	120.5	68.5	174	67	107	11.4	5.3	74.3	2	1	1	1	1	1	1	9.2	0.04	21.5
1	0	0	100	7.04	2	75	21.6	123	69.5	228	67	161	10	5.4	77.2	2	1	1	1	0	1	1	5.2	0.09	0.05

1	0	0	100	5.06	2	67	28.8	121	70	202	67	135	14	5.4	76.9	2	1	1	0	1	1	1	14.2	0.02	30.6
1	0	0	100	5.08	2	74	27.5	151.5	76.5	195	67	128	12.3	5.7	77.8	1	1	1	0	1	1	1	13.8	0.05	20
1	0	0	100	5.08	2	78	25.6	134.5	86.5	204	67	137	13.5	5.8	67.7	2	1	1	0	1	1	1	10.4	0.02	22.5
1	0	0	100	6.98	2	74	30	138.5	80	168	67	101	13.3	5.8	73.2	1	1	1	0	1	1	1	21.8	0.02	23.2
1	0	0	100	6.95	2	75	25.9	132	75	223	67	156	12.6	5.3	83.1	1	1	1	0	0	1	1	60.8	0.04	43.9
1	0	0	100	2.24	2	74	26.7	136	76	243	67	176	13.1	7	73.2	2	1	1	0	0	0	1	5	0.03	87.4
1	0	0	100	6.83	2	74	15.7	148	78	172	67	105	13.4	6.8	73.2	1	1	1	0	1	0	1	36.1	0.01	22.6
1	0	0	100	4.69	2	71	21.8	125.5	71.5	218	67	151	14.1	5.4	79.4	1	1	0	1	1	1	1	10	0.05	25.3
1	0	0	100	6.97	2	65	29.5	134.5	79	219	67	152	13.1	5.3	74.2	2	1	0	0	1	1	1	4.5	0.03	16.5
1	0	0	100	5.37	2	76	21.9	126	71.5	162	67	95	12.1	5	76.7	1	1	1	1	1	1	1	58.9	0.02	20.3
1	0	0	100	5.06	2	69	22.6	107	69.5	192	67	125	12.4	5	75.9	2	1	1	1	1	1	1	15	0.04	38.9
1	0	0	100	6.86	2	77	25.3	118.5	79	190	67	123	13.9	5.6	68.2	1	1	1	1	0	1	1	7.3	0.06	11.4
1	1	0	100	7	2	77	23.6	122.5	74	190	67	123	13.4	5.2	58.0	1	1	1	1	1	1	1	56.1	0.03	53.2
1	1	0	100	6.84	2	74	22.2	126.5	75.5	222	67	155	14	5.2	59.2	1	1	1	1	0	1	1	22.6	0.01	54
1	1	0	100	4.21	2	76	24.3	113	75.5	196	67	129	11	5.5	58.4	2	1	1	0	1	1	1	32.8	0.01	12.5
1	1	0	100	7.01	2	71	22.5	156	88	204	67	137	11.2	5	74.8	2	1	1	0	1	1	1	27.6	0.01	34.4
1	1	0	100	6.59	2	79	21.7	146	75	231	67	164	13	5.1	70.7	1	1	1	0	0	1	1	136.2	0.01	54.4
1	1	0	100	3.78	2	71	30.8	154.5	93.5	198	67	131	13.5	5.8	74.8	1	1	1	0	1	1	1	57.6	0.03	10.4
1	1	0	100	2.96	2	69	23.4	123	68.5	226	67	159	12.5	5.4	72.1	2	1	1	1	0	1	1	12	0.02	21.1
1	0	0	100	7.23	2	81	23.7	185	96.5	215	67	148	12.3	5.7	66.3	2	1	1	0	1	1	1	28.3	0.06	63.7
1	1	0	100	4.49	2	82	18.5	115.5	70.5	200	67	133	12.6	5.2	65.8	2	1	1	1	1	1	1	13.8	0.01	68.2
1	1	0	100	6.8	2	84	24.9	149.5	84	174	67	107	13.6	5.7	64.9	2	1	1	0	1	1	1	41.7	0.04	16.2
1	1	1	6	6	1	79	24.7	143.5	83	237	67	170	15.7	5.4	57.9	2	0	0	0	0	1	1	28.5	0.62	8.95
1	0	1	5	5	2	70	25.4	147.5	79.5	167	67	100	14.9	9.2	80.0	1	1	1	0	1	0	1	2005.8	0.02	25.4
1	0	1	2.6	2.6	2	70	23.2	170	90.5	208	67	141	14.4	5.8	75.3	2	1	1	0	1	1	1	40.8	0.06	25.6
1	0	0	100	4.58	1	71	26	162.5	99	210	68	142	13.4	5.2	77.2	2	0	0	0	1	1	1	36.9	0.14	14.6
1	0	0	100	5.16	1	69	25	135	84	206	68	138	13.9	5.4	70.6	2	1	1	0	1	1	1	9.6	0.13	16.8
1	0	0	100	4.69	1	68	20.1	167.5	89.5	119	68	51	12.5	5.2	84.0	2	0	0	0	1	0	1	55.4	1	116
1	0	0	100	5.04	1	77	26.8	144	82.5	175	68	107	14.1	5.5	74.0	2	1	0	0	1	1	1	27.9	0.2	34.1
1	0	0	100	7	1	66	21.3	145	72	180	68	112	13.4	5.5	75.7	2	1	0	0	1	1	1	14.3	0.03	4.55
1	0	0	100	7.07	1	65	23.5	163.5	84	177	68	109	9.7	5.7	76.2	2	1	0	0	1	1	1	26	0.02	81.1
1	0	0	100	5.15	1	69	19.3	147.5	82	187	68	119	14.3	5.5	74.1	2	1	0	0	1	1	1	6.9	0.01	62.2
1	0	0	100	7.21	1	73	27.1	143.5	74.5	229	68	161	15	5.5	72.0	2	1	0	0	0	1	1	46.8	0.09	28.1
1	0	0	100	6.85	1	70	24.8	138	81	200	68	132	16.2	5.6	55.0	2	1	0	0	1	1	1	2.4	0.06	16.9
1	0	0	100	7	1	65	23.9	108	61.5	147	68	79	13.1	5.7	85.8	2	0	1	1	1	1	1	14.1	0.02	6.53
1	0	0	100	7	1	70	22.2	130.5	76	146	68	78	15.8	5.4	77.7	2	1	0	1	1	1	1	15.3	0.03	46
1	0	0	100	6.97	1	66	20.2	126	72.5	232	68	164	15.6	5.7	85.2	2	0	0	1	0	1	1	44.2	0.04	3.89
1	0	0	100	7	1	75	21.6	107.5	57	183	68	115	13.5	5.6	67.7	2	1	1	1	1	1	1	9.2	0.02	24.9
1	0	0	100	4.63	1	79	23.3	121	66.5	195	68	127	14.7	5.6	57.9	2	1	1	1	1	1	1	9.1	0.03	83.1
1	0	0	100	6.87	1	79	22.9	124.5	72.5	165	68	97	14	5.4	73.0	2	1	1	1	1	1	1	7.4	0.01	10.1
1	0	0	100	6.98	1	72	21.6	171	87	203	68	135	13.8	5.3	76.7	2	0	0	0	1	1	1	55.5	0.05	50
1	0	0	100	6.98	1	71	25.7	150.5	78.5	137	68	69	14.4	5.3	77.2	2	0	0	0	1	1	1	56.2	0.03	46.6
1	0	0	100	7.15	1	70	24.9	135.5	92.5	191	68	123	14.4	5.4	70.1	2	1	0	0	1	1	1	2.6	0.05	20.7
1	0	0	100	6.82	1	73	23.5	142	83.5	170	68	102	14.4	5.3	76.1	2	1	0	0	1	1	1	11.3	0.03	23.1
1	0	0	100	4.59	1	67	28.3	164.5	83.5	206	68	138	12.5	5.7	75.2	2	1	0	0	1	1	1	439.2	0.04	19.5
1	0	0	100	4.59	1	79	21.5	137.5	84	179	68	111	13	4.5	57.9	1	1	0	0	1	1	1	24.6	0.01	46.8
1	0	0	100	4.62	1	68	25.8	138.5	84	189	68	121	15.5	5.4	71.1	2	1	0	0	1	1	0	226.3	0.04	140
1	0	0	100	4.62	1	69	20.7	158	85	183	68	115	14	5.2	78.3	2	0	0	0	1	1	1	17.3	0.02	24
1	0	0	100	4.67	1	72	27.8	172	86	180	68	112	16	5.1	60.9	2	1	0	0	1	1	1	54.4	0.03	84
1	0	0	100	5.37	1	67	18.3	107	69	205	68	137	13.1	5.4	79.4	2	1	0	1	1	1	1	20.9	0.01	8.54
1	0	0	100	5.06	1	69	23.9	127.5	82	185	68	117	14	5.4	74.1	2	1	0	0	1	1	1	59	0.05	23

1	0	0	100	5.02	1	65	22.8	160	89.5	234	68	166	15.4	10.6	76.2	2	1	1	0	0	0	1	69.8	0.04	5.34
1	1	0	100	6.87	1	74	21.8	123	72	148	68	80	12.2	4.7	71.5	2	1	0	1	1	1	1	31.3	0.64	53.5
1	0	0	100	4.59	2	65	24.3	124	86.5	224	68	156	13.6	5.4	78.0	2	1	1	1	0	1	1	71	0.12	9.53
1	0	0	100	6.84	2	68	28.4	108.5	61.5	224	68	156	12.9	6.1	61.8	2	1	1	1	0	1	1	7.3	0.13	0.89
1	0	0	100	6.95	2	69	25.9	101.5	64	262	68	194	14.3	8.4	80.5	2	1	1	1	0	0	1	17	0.1	9.43
1	0	0	100	6.95	2	73	25.8	144	73.5	217	68	149	12.7	5.9	70.1	2	1	1	0	0	1	1	20.7	0.12	44.4
1	0	0	100	7.15	2	70	21.9	117	69	253	68	185	13.1	5.9	71.6	2	1	1	1	0	1	1	18.3	0.32	22.9
1	0	0	100	4.67	2	76	23.5	160.5	80	202	68	134	13.5	6.2	76.7	1	1	1	0	1	1	1	60.4	0.17	19.1
1	0	0	100	4.67	2	66	26.1	176.5	101.5	203	68	135	15.6	5.5	77.5	2	1	1	0	1	1	1	60.7	0.2	11.7
1	0	0	100	5.04	2	75	24.8	111.5	47	213	68	145	11.5	6.1	69.1	2	1	1	0	1	0	1	5	0.17	75.9
1	0	0	100	7	2	71	18.8	129.5	58.5	260	68	192	12.8	4.7	74.8	1	1	1	1	0	1	1	15.7	0.01	1.86
1	0	0	100	7.06	2	73	23.9	134.5	70	172	68	104	12.4	5.4	59.7	1	1	1	0	1	1	1	30.9	0.07	14
1	0	0	100	7.08	2	68	25	128.5	69	253	68	185	13.5	5.6	76.4	2	1	1	0	0	1	1	31.6	0.04	6.23
1	0	0	100	4.58	2	71	22.3	110	77	171	68	103	13.5	5.1	74.8	1	1	1	0	1	1	1	25.3	0.03	6.99
1	0	0	100	6.86	2	70	21.8	166.5	84.5	191	68	123	12.7	5.1	60.9	2	1	1	0	1	1	1	10.4	0.07	33.2
1	0	0	100	6.87	2	68	17.7	132	88	262	68	194	14.3	5	72.6	2	1	1	0	0	1	1	48.3	0.03	15.7
1	0	0	100	6.87	2	65	22.7	126	76.5	162	68	94	12.7	5.2	63.1	2	1	0	0	1	1	1	20.8	0.03	32.1
1	0	0	100	6.84	2	69	31.4	95.5	60	185	68	117	13.9	5.2	72.1	1	1	1	1	1	1	1	4.7	0.07	56.9
1	0	0	100	6.85	2	69	23.2	108	70.5	311	68	243	13.1	5.6	75.9	2	1	1	1	0	1	1	18.6	0.03	0.05
1	0	0	100	6.85	2	65	24	148	87	138	68	70	12.7	5	78.0	2	1	1	0	1	1	1	18.7	0.03	20.1
1	0	0	100	7.21	2	70	22.9	116.5	72	234	68	166	10.8	6.3	71.6	2	1	1	1	0	1	1	7.6	0.04	1.98
1	0	0	100	7.02	2	68	24.8	176.5	86.5	220	68	152	13.6	5.2	61.8	2	1	1	0	0	1	1	17.8	0.01	50.2
1	0	0	100	5.08	2	72	23.7	143	73.5	226	68	158	12.6	5.4	60.1	1	1	1	0	0	1	1	94.4	0.05	18.2
1	0	0	100	4.62	2	70	20.7	145.5	83.5	233	68	165	13.9	5.5	75.3	2	1	1	0	0	1	1	15.1	0.05	28.4
1	0	0	100	6.97	2	65	20	129.5	80.5	184	68	116	11.5	5.3	54.7	2	1	1	1	1	1	1	161.9	0.01	26.6
1	0	0	100	4.59	2	70	22.3	134	78	225	68	157	13.8	5.5	75.3	2	1	1	1	0	1	1	17.1	0.01	23.4
1	0	0	100	4.62	2	66	25.1	132	75.5	193	68	125	13.5	5.3	77.5	2	1	1	0	1	1	1	15.6	0.02	28.4
1	0	0	100	5.36	2	66	25.4	138	74	208	68	140	13.8	5.6	62.7	2	1	1	1	1	1	1	22.9	0.02	21.6
1	0	0	100	5.37	2	73	23.1	97.5	62	248	68	180	12.5	6.1	73.8	2	1	1	1	0	1	1	8	0.04	18.1
1	0	0	100	5.37	2	74	23.4	112.5	71.5	218	68	150	13.7	5.1	45.2	2	1	1	0	1	1	1	4.5	0.02	56.9
1	0	0	100	6.85	2	69	21.2	118.5	71.5	183	68	115	13.6	5.1	72.1	2	1	0	1	1	1	1	16	0.01	20.6
1	0	0	100	6.86	2	75	21.7	143	85	197	68	129	13.6	5.2	72.7	1	1	1	0	1	1	1	33.1	0.04	18.9
1	0	0	100	6.87	2	68	26.3	122	76	218	68	150	14.8	6.3	72.6	2	1	1	1	0	1	1	18	0.09	3.89
1	0	0	100	5.04	2	69	22.7	184	91.5	155	68	87	13.6	6.2	75.9	2	1	1	0	1	0	1	138.1	0.01	34.5
1	1	0	100	7.05	2	72	27.7	145	83.5	247	68	179	13.9	5	70.6	2	1	1	0	0	1	1	156	0.05	25.3
1	1	0	100	7.07	2	76	20.5	121.5	78	222	68	154	14.5	5.3	50.7	2	1	1	0	0	1	1	526.8	0.07	91.7
1	1	0	100	4.58	2	73	17.5	158	82.5	234	68	166	12.6	5.8	73.8	1	1	1	0	0	1	1	80.5	0.01	24.9
1	0	0	100	6.87	1	84	19.7	133.5	66.5	182	68	114	11.3	5.7	63.5	1	0	0	0	1	1	1	13.2	0.01	57
1	0	0	100	6.97	1	66	26.1	131.5	78.5	206	68	138	14.3	6.1	75.7	2	1	0	1	1	1	1	3.1	0.15	1.44
1	0	0	100	5.16	1	70	25.1	160	81	212	68	144	14.2	5.3	61.7	2	1	0	0	1	1	1	10.7	0.02	52.9
1	1	0	100	5.05	1	73	20.5	130	70	137	68	69	13.8	7.5	81.1	2	1	0	1	1	0	1	858.4	0.06	159
1	1	0	100	6.86	1	72	22.6	134.5	77	180	68	112	14.6	6.9	72.6	2	1	1	1	1	0	1	25	0.08	30.7
1	0	0	100	6.85	2	71	23.6	178	94	225	68	157	13.8	6.5	74.8	2	1	1	0	0	0	1	35.3	0.69	28.7
1	0	0	100	4.62	2	70	23	144.5	76.5	181	68	113	10.7	6.3	80.0	2	1	1	0	1	1	1	9.2	0.55	35.2
1	0	0	100	6.87	2	75	25.6	118	74	225	68	157	12.5	5.2	69.1	2	1	1	1	0	1	1	16.6	0.19	9.3
1	0	0	100	7.05	2	68	25	169	81	197	68	129	13.1	5.5	72.6	1	1	1	0	1	1	1	75.8	0.05	18.9
1	0	0	100	7.08	2	74	22.1	142	77.5	220	68	152	12.3	6.6	77.8	2	1	1	0	0	0	1	14.3	0.05	13.5
1	0	0	100	6.86	2	78	22.8	134.5	66.5	244	68	176	14.2	5.1	75.6	2	1	1	1	0	1	1	14.5	0.02	28
1	0	0	100	5.16	2	75	35.4	154.5	83.5	184	68	116	14.7	6	72.7	2	1	1	0	1	1	1	29.6	0.01	36.3
1	0	0	100	6.86	2	69	25.1	114	75	236	68	168	12.6	5.3	80.5	2	1	1	0	0	1	1	7.5	0.04	45.9
1	0	0	100	7.04	2	72	25.1	152.5	79.5	227	68	159	13.4	5.9	74.3	2	1	1	0	0	1	1	197.1	0.02	23.7

1	0	0	100	5.05	2	70	24.4	153	83.5	173	68	105	12.4	4.9	46.5	2	1	1	0	1	1	1	18.9	0.05	21.3
1	0	0	100	6.98	2	73	23.3	131.5	77.5	232	68	164	11.8	5.6	70.1	2	1	1	1	0	1	1	28.6	0.03	0.05
1	0	0	100	7	2	66	29.9	137	84.5	191	68	123	12.2	5.7	73.6	2	1	1	0	1	1	1	29.8	0.05	8.52
1	0	0	100	4.64	2	72	23.8	198	89	224	68	156	14	5.2	78.9	2	1	1	0	0	1	1	12.8	0.02	26.5
1	0	0	100	6.96	2	68	28.6	129.5	72	222	68	154	14.3	5.4	61.8	1	1	1	0	0	1	1	25	0.06	22.7
1	0	0	100	2.99	2	77	22.2	158	83	202	68	134	11.3	5.3	71.7	2	1	1	0	1	1	1	82.2	0.09	31.8
1	0	0	100	4.62	2	72	24.2	114.5	65.5	237	68	169	12.6	5.2	70.6	2	1	1	1	0	1	1	11.3	0.05	27.6
1	0	0	100	4.67	2	65	21.1	149.5	81.5	238	68	170	13.2	5.4	78.0	2	1	1	0	0	1	1	37.7	0.05	33.1
1	0	0	100	6.97	2	66	19.8	154.5	87	282	68	214	12.4	5.5	77.5	2	1	1	0	0	1	1	763.6	0.03	20.9
1	0	0	100	6.97	2	67	23.6	148.5	77	209	68	141	10	5.5	76.9	2	1	1	0	0	1	1	19.4	0.01	29.5
1	1	0	100	6.86	2	75	30.5	137	79.5	188	68	120	12.8	5.9	58.8	1	1	1	0	1	1	1	22.9	0.66	12.9
1	1	0	100	6.95	2	70	16.1	146.5	81	175	68	107	11.9	5.8	80.0	2	1	1	0	1	1	1	58.8	0.81	27.9
1	1	0	100	7.06	2	73	22.5	163.5	100.5	178	68	110	14	5.5	59.7	1	1	1	0	1	1	1	46.1	0.06	89.2
1	1	0	100	6.87	2	66	30.4	135.5	75	226	68	158	12.2	5.5	73.6	2	1	1	1	0	1	1	13.7	0.06	20.6
1	1	0	100	6.8	2	71	27.9	140	77.5	225	68	157	10	6	74.8	2	1	1	0	0	1	1	8.6	0.02	23.1
1	1	0	100	5.07	2	79	24.6	138.5	77	187	68	119	13.8	5.6	43.7	2	1	0	0	1	1	1	22.3	0.05	84.1
1	0	0	100	7.03	2	81	23.2	140	70.5	239	68	171	13	7	56.4	1	1	1	0	0	0	1	1449.4	0.09	0.55
1	0	0	100	6.84	2	85	20.1	124	59	228	68	160	11.1	5.8	64.4	1	1	1	1	0	1	1	49.5	0.03	28.5
1	1	0	100	7.08	2	82	22.2	139	73.5	171	68	103	7.8	5.1	48.6	1	1	1	0	1	1	1	25.1	0.01	125
1	0	1	2.4	2.4	1	67	28.1	158.5	94.5	228	68	160	16.1	5.6	75.2	2	1	0	0	0	1	1	105.3	0.02	26.9
1	1	1	1.9	1.9	1	76	20.3	129	72.5	219	68	151	13.1	5.7	79.4	1	0	0	0	1	1	1	44.8	0.31	18.4
1	1	1	2.9	2.9	2	76	24.7	148.5	90	214	68	146	14.8	5.2	72.2	2	1	1	0	1	1	1	13.8	0.11	42.7
1	1	1	3	3	1	71	18.7	151	80.5	150	68	82	14.6	5.1	73.1	2	0	0	0	1	1	1	4.7	0.05	53
1	1	1	4.9	4.9	2	75	25.8	117	62	185	68	117	9.7	5.7	69.1	1	1	1	0	1	1	1	22.1	0.01	19.6
1	0	0	100	7.03	1	77	18.2	145.5	81	228	69	159	14.2	5.6	66.7	2	1	0	0	0	1	1	6	0.68	0.05
1	0	0	100	6.82	1	68	20	100.5	64	180	69	111	15.2	5.1	84.0	2	1	0	1	1	1	1	8	0.15	33.3
1	0	0	100	4.61	1	66	24.4	157.5	84.5	204	69	135	15.3	6.1	85.2	2	0	0	0	1	1	1	16.8	0.46	43.1
1	0	0	100	4.62	1	72	24	146.5	80	199	69	130	12.1	5	69.1	1	0	0	0	1	1	1	68.7	0.18	22.8
1	0	0	100	4.62	1	65	26	169	102	211	69	142	11.9	5.3	57.0	2	1	0	0	1	1	1	8.1	0.14	56.2
1	0	0	100	4.62	1	66	21.1	155.5	75	188	69	119	13.9	4.9	72.1	2	1	0	0	1	1	1	19.2	0.11	94.9
1	0	0	100	6.87	1	69	23.9	131	86.5	210	69	141	13.3	5.2	74.1	2	1	0	0	1	1	1	13.8	0.13	4.81
1	0	0	100	7.07	1	67	24.8	155	96.5	195	69	126	15.2	5.8	71.6	2	1	0	0	0	1	1	50.3	0.03	6.45
1	0	0	100	5.16	1	66	23	104	63.5	218	69	149	14.2	5.4	72.1	2	0	0	1	1	1	1	10.6	0.01	2.35
1	0	0	100	6.85	1	79	24.9	119	78.5	203	69	134	14.1	5.5	65.8	2	1	0	0	1	1	1	9.4	0.08	13.1
1	0	0	100	7.03	1	71	20.2	145	80	167	69	98	13.5	4.8	61.3	2	1	0	0	1	1	1	7	0.03	8.65
1	0	0	100	5.36	1	79	20.1	170.5	98.5	189	69	120	16.5	5	69.1	1	0	0	0	1	1	1	109.7	0.01	46.9
1	0	0	100	5.06	1	67	24.6	156.5	89	205	69	136	15.8	5.1	75.2	2	1	0	0	0	1	1	103.9	0.02	12.4
1	0	0	100	6.98	1	68	23.3	155	73	220	69	151	14.8	5.8	74.6	2	1	0	0	0	1	1	7.1	0.03	12
1	0	0	100	6.95	1	71	26.2	154.5	86.5	245	69	176	13.9	5.6	37.5	2	1	0	0	0	1	1	1924.4	0.04	33
1	0	0	100	6.82	1	67	23.1	134	88.5	243	69	174	14.8	5.6	71.6	2	1	1	1	0	1	1	4.3	0.07	17.3
1	0	0	100	6.86	1	67	23.9	123	79.5	223	69	154	13.8	7.6	79.4	2	1	0	0	0	0	1	43.8	0.04	12.9
1	0	0	100	6.87	1	68	21.9	110	66.5	209	69	140	15.6	5.4	74.6	2	1	1	1	1	1	1	9.6	0.02	3.89
1	1	0	100	6.84	1	76	22.2	127	61.5	189	69	120	14.4	5.2	52.7	2	1	0	0	1	1	1	6.5	0.6	39.3
1	1	0	100	5.04	1	72	22.8	145.5	75	184	69	115	14.3	5.4	72.6	2	0	0	0	1	1	1	165.1	2.51	16.8
1	1	0	100	6.86	1	77	21.5	104.5	66	184	69	115	13.9	5.5	70.1	2	0	0	1	1	1	1	13.4	0.04	28.2
1	1	0	100	6.86	1	77	24.6	144.5	81	206	69	137	13.9	5.9	70.1	1	1	0	0	1	1	1	17.7	0.05	33.2
1	0	0	100	4.58	2	69	20.4	145.5	81.5	189	69	120	12.9	5.2	72.1	2	1	1	0	1	1	1	100.5	0.1	88.1
1	0	0	100	6.88	2	65	25.2	117.5	68.5	272	69	203	13.4	6	54.7	2	1	0	1	0	1	1	9.9	0.15	0.3
1	0	0	100	6.88	2	66	26.3	120	66.5	204	69	135	12.2	5.9	73.6	2	1	1	1	1	1	1	9.2	0.11	52.9
1	0	0	100	5.05	2	74	22.4	139.5	86	154	69	85	13.3	5.3	73.2	2	1	0	0	1	1	1	29.4	0.58	42
1	0	0	100	6.87	2	68	21.4	117	63.5	186	69	117	12	5.2	72.6	2	1	1	0	1	1	1	365.9	0.13	21.8

1	0	0	100	2.05	2	67	29.8	128	81.5	226	69	157	12.8	5.7	76.9	2	1	1	1	0	1	1	12.2	0.11	2.29
1	0	0	100	4.62	2	66	24.3	128.5	76.5	143	69	74	12.2	5.9	77.5	2	1	1	1	1	1	1	41.2	0.94	44.8
1	0	0	100	6.44	2	66	22.3	122.5	61.5	203	69	134	12	4.9	62.7	2	1	0	1	1	1	1	11.6	0.68	6.46
1	0	0	100	6.87	2	79	27.3	147	80	224	69	155	12.3	5.4	57.2	1	1	1	0	0	1	1	1298.9	0.25	18
1	0	0	100	7	2	66	23.7	122.5	60.5	242	69	173	12.7	5.4	77.5	2	1	1	1	0	1	1	12.5	0.01	57.7
1	0	0	100	7	2	74	26	210.5	99.5	221	69	152	13.6	7	77.8	2	1	1	0	0	0	1	163.6	0.05	25
1	0	0	100	7.06	2	71	25.2	131.5	78	213	69	144	12.2	5.3	71.1	1	1	1	1	1	1	1	15.6	0.04	40.6
1	0	0	100	7.07	2	65	26.4	117.5	81	220	69	151	10.7	5.8	78.0	2	1	1	1	0	1	1	21.3	0.02	28.3
1	0	0	100	4.58	2	68	17.6	111	71	197	69	128	13.6	4.9	61.8	1	1	1	1	1	1	1	18.2	0.01	29.1
1	0	0	100	4.58	2	67	25.8	126	69.5	219	69	150	14.6	6	81.7	2	1	0	0	1	1	1	17.4	0.04	10.3
1	0	0	100	4.58	2	70	22.1	114	78.5	195	69	126	13.2	5.7	52.8	2	1	1	1	1	1	1	65.1	0.03	58
1	0	0	100	6.86	2	73	21.9	94	52	185	69	116	12.6	5	70.1	2	1	1	1	1	1	1	3.8	0.06	35.5
1	0	0	100	6.87	2	73	21.9	108.5	65	254	69	185	12.2	5.1	59.7	1	1	1	1	0	1	1	13.1	0.01	25.5
1	0	0	100	6.87	2	70	25.1	139	75.5	180	69	111	14.3	5.2	80.0	2	1	1	1	1	1	1	6.9	0.03	39.8
1	0	0	100	5.16	2	68	24.5	147.5	81	258	69	189	12.6	5.9	47.2	2	1	1	0	0	1	1	132.5	0.03	44.4
1	0	0	100	5.16	2	76	21.9	149	87	146	69	77	13.6	5	72.2	1	1	1	0	1	1	1	9.1	0.02	39.8
1	0	0	100	5.16	2	66	20.7	135.5	80.5	249	69	180	15.3	5.2	82.3	2	1	0	0	0	1	1	12.2	0.03	7.17
1	0	0	100	6.84	2	66	20.8	122	66	198	69	129	13.7	5.1	73.6	2	1	0	1	1	1	1	7.7	0.06	29.8
1	0	0	100	6.84	2	70	27.3	139.5	87	224	69	155	13.4	5.5	75.3	2	1	1	0	0	1	1	10.8	0.05	20.6
1	0	0	100	7.2	2	70	28.7	130	67	187	69	118	14.1	5.7	71.6	2	1	1	1	0	1	1	38.4	0.03	27.9
1	0	0	100	5.05	2	67	21.1	120	71	189	69	120	12.9	5.5	62.2	2	1	1	1	1	1	1	9.2	0.01	53.3
1	0	0	100	5.06	2	69	27.6	161	94	198	69	129	14.2	5.2	75.9	2	1	0	0	1	1	1	17.1	0.04	31.2
1	0	0	100	5.06	2	69	22.1	121	64	182	69	113	11	5.1	72.1	2	1	1	1	1	1	1	8	0.04	26.8
1	0	0	100	5.08	2	72	24.4	128	78	209	69	140	12.3	5.3	70.6	1	1	1	0	1	1	1	117.7	0.02	10.7
1	0	0	100	6.98	2	65	19.8	133.5	75	197	69	128	12.4	5.5	78.0	2	1	1	0	1	1	1	7.2	0.03	7.13
1	0	0	100	4.63	2	74	23.2	154.5	68.5	202	69	133	11.9	7.2	73.2	1	1	0	0	1	0	1	14	0.09	50.1
1	0	0	100	4.64	2	70	20.1	116	71.5	184	69	115	12.1	5.5	75.3	2	1	1	1	1	1	1	11.9	0.07	21.5
1	0	0	100	6.95	2	68	23.5	145	84	266	69	197	13.4	5.2	61.8	2	1	1	0	0	1	1	50.5	0.05	36.6
1	0	0	100	6.95	2	78	28.8	123.5	81.5	220	69	151	13.2	5.4	71.2	2	1	1	1	0	1	1	13.9	0.04	11.3
1	0	0	100	6.97	2	67	21.5	92	57.5	208	69	139	12.8	5.6	96.6	2	1	1	1	1	1	1	18.8	0.07	8.63
1	0	0	100	6.97	2	77	22.7	153.5	84	183	69	114	14.1	5.1	68.2	1	1	1	0	1	1	1	17.9	0.01	29.8
1	0	0	100	6.98	2	68	24.4	140	90.5	210	69	141	13.5	5.5	72.6	2	1	1	0	1	1	1	33.4	0.01	30.4
1	0	0	100	6.79	2	66	17.1	100	62.5	253	69	184	13.4	5.1	73.6	2	1	1	1	0	1	1	10.1	0.01	9.47
1	0	0	100	4.6	2	70	23.7	135.5	84.5	162	69	93	14.1	6.4	80.0	2	1	1	0	1	1	1	12.3	0.02	3.52
1	0	0	100	4.61	2	70	26.8	154	80.5	188	69	119	14.4	5.4	75.3	2	1	0	0	1	1	1	29	0.04	18.7
1	0	0	100	4.62	2	65	23.2	185	98.5	251	69	182	13.3	5	82.8	2	1	1	0	0	1	1	126.9	0.04	17.2
1	0	0	100	5.37	2	65	24.4	128.5	69	200	69	131	12.3	7.4	74.2	2	1	1	1	0	0	1	5.5	0.03	36.7
1	0	0	100	5.37	2	66	21.1	131	74.5	208	69	139	14.3	5.8	77.5	2	1	0	0	1	1	1	41.8	0.01	46.5
1	0	0	100	5.37	2	68	22.6	118	69.5	219	69	150	13.1	5.4	76.4	2	1	1	0	1	1	1	50.2	0.02	31
1	0	0	100	6.44	2	69	22.9	168	95	278	69	209	12.6	5.8	75.9	2	1	1	0	0	1	1	818.8	0.03	9.85
1	0	0	100	6.87	2	73	26	141	85.5	188	69	119	13	5.3	70.1	2	1	1	0	1	1	1	43.1	0.03	19.4
1	1	0	100	6.87	2	76	20.9	134	73.5	225	69	156	11.5	5.4	68.6	2	1	1	1	0	1	1	36.1	0.04	53.2
1	1	0	100	3.62	2	76	21.7	125	75.5	227	69	158	13.2	5.6	76.7	1	1	0	0	0	1	1	70.7	0.03	49.7
1	1	0	100	4.61	2	76	27.6	122.5	63	199	69	130	13.3	5.7	44.6	1	1	1	1	1	1	1	9.5	0.01	67.1
1	0	0	100	6.97	1	80	18.2	113	78.5	172	69	103	13.1	5.4	68.6	1	1	0	1	1	1	1	8.7	1.42	12.4
1	1	0	100	6.86	1	81	23.1	130	68	203	69	134	11.6	5.2	64.9	2	1	1	1	1	1	1	16.3	0.05	33.1
1	0	0	100	6.86	2	69	25.6	120	84	193	69	124	11.3	5	75.9	2	1	1	0	1	1	1	26.1	0.16	12.9
1	0	0	100	5.06	2	67	28.6	125.5	66	219	69	150	13.3	5.2	76.9	2	1	1	0	1	1	1	34.6	0.1	18.7
1	0	0	100	4.69	2	69	27.6	125	74	175	69	106	13.7	5.3	80.5	2	1	1	0	1	1	1	47.8	0.35	16.8
1	0	0	100	5.39	2	65	28	135.5	75.5	187	69	118	12.6	5.4	78.0	2	1	1	0	0	1	1	9.4	0.19	37.9
1	0	0	100	7.07	2	65	18.7	128.5	64.5	192	69	123	12.8	4.8	74.2	2	1	1	1	1	1	1	22.5	0.06	72.7

1	0	0	100	6.86	2	72	23.9	147	77	213	69	144	12.1	6.8	74.3	2	1	1	0	1	0	1	48.8	0.09	32.6
1	0	0	100	6.86	2	68	23.7	144.5	70	181	69	112	10.5	4.9	81.1	2	1	0	0	1	1	1	16.7	0.02	48.9
1	0	0	100	7.23	2	76	32.4	191.5	103	245	69	176	14.3	5.4	68.6	2	1	1	0	0	1	1	8	0.05	19.8
1	0	0	100	6.87	2	77	19.4	153	75.5	249	69	180	12.5	5.8	76.1	2	1	1	0	0	1	1	17.8	0.02	39
1	0	0	100	5.16	2	72	21.4	121.5	61	210	69	141	13.3	5.8	60.1	2	1	1	0	1	1	1	9.1	0.01	9.76
1	0	0	100	7	2	66	21.9	110.5	71	192	69	123	12.3	5	82.3	2	1	1	0	1	1	1	5.5	0.01	10.8
1	0	0	100	6.87	2	69	20.1	118	59	173	69	104	13.4	5.4	72.1	2	1	1	1	1	1	0	7.5	0.05	114
1	0	0	100	6.44	2	72	24.8	149	81.5	208	69	139	12.2	5.3	70.6	2	1	1	0	1	1	1	170.3	0.03	12.2
1	0	0	100	5.37	2	67	21.8	135.5	78.5	194	69	125	12.8	5.7	62.2	2	1	1	0	1	1	1	8.4	0.06	52.4
1	0	0	100	6.87	2	66	19.2	107.5	69	168	69	99	11.7	5.5	82.3	2	1	1	1	1	1	1	15.9	0.01	9.3
1	0	0	100	5.04	2	65	33.1	129.5	73.5	200	69	131	11.7	5.7	78.0	2	1	0	0	1	1	1	56.6	0.05	26.4
1	1	0	100	7.07	2	73	26	147	83	256	69	187	14.6	5.5	70.1	2	1	1	0	0	1	1	10.6	0.12	24.2
1	1	0	100	3.66	2	70	29.2	120.5	75.5	276	69	207	14	5.2	80.0	2	1	1	0	0	1	1	18.4	0.01	19.5
1	1	0	100	6.98	2	68	21.9	108.5	65.5	175	69	106	10.1	5.5	76.4	1	1	1	1	1	1	1	7.9	0.01	12.3
1	1	0	100	6.97	2	76	21.7	114.5	58.5	226	69	157	13.7	5.6	72.2	2	1	1	1	0	1	1	144.5	0.02	8.93
1	1	0	100	6.83	2	66	20.2	109	66	180	69	111	12.7	5	77.5	2	1	1	1	1	1	1	17.6	0.04	9.12
1	1	0	100	6.87	2	77	31.9	166.5	101.5	220	69	151	14	5.2	50.3	1	1	1	0	0	1	1	1121.1	0.02	60.1
1	1	0	100	7	1	84	20.1	141.5	64	213	69	144	14.3	5.5	66.7	2	1	1	0	1	1	1	41.7	0.02	24.7
1	1	1	6.4	6.4	1	72	21	117	73	161	69	92	15.3	5.7	76.7	2	0	0	1	1	1	1	23.9	0.02	28.8
1	0	1	1	1	2	66	19	131	78	213	69	144	12.1	5.6	77.5	2	1	1	0	1	1	1	14.8	0.02	33.1
1	1	1	6.7	6.7	2	72	24.2	131	66.5	188	69	119	11.7	5.3	70.6	2	1	1	1	1	1	1	27.6	0.04	48
1	0	0	100	5.73	1	70	23.6	163	88	204	70	134	13	5.2	73.6	2	0	0	0	1	1	1	163	0.1	14.7
1	0	0	100	4.63	1	65	20	157.5	92.5	185	70	115	15.9	7.6	80.5	2	0	0	0	1	0	1	8.6	0.17	13.9
1	0	0	100	4.64	1	71	27.4	135	77	193	70	123	14.8	5.8	73.1	1	1	0	1	1	1	1	8.3	0.29	11.9
1	0	0	100	6.97	1	70	24.8	132.5	70.5	138	70	68	15.1	4.9	77.7	2	1	0	0	1	1	1	39.8	2.21	24.1
1	0	0	100	6.8	1	74	24.2	110	68.5	214	70	144	14.7	5.4	71.5	2	1	0	1	1	1	1	5.2	0.43	27.6
1	0	0	100	6.87	1	72	21.3	154.5	82.5	180	70	110	13.4	5.1	88.0	2	0	1	0	1	1	1	21	0.2	11.1
1	0	0	100	7.05	1	69	22.6	133	72.5	175	70	105	14.3	5.7	70.6	2	0	1	1	1	1	1	12.7	0.01	22.5
1	0	0	100	7.06	1	70	22.2	164	102.5	198	70	128	15.5	5.3	61.7	2	0	1	0	0	1	1	8.1	0.03	36.1
1	0	0	100	7.06	1	74	22.6	144	82.5	222	70	152	11.3	5.6	68.2	2	1	0	0	0	1	1	7	0.01	58.7
1	0	0	100	7.07	1	71	25.8	120	75	202	70	132	16.3	4.9	82.2	1	1	0	0	1	1	1	12.1	0.06	15
1	0	0	100	7.08	1	75	21.8	116.5	66.5	213	70	143	12.8	6.3	75.1	2	0	1	1	1	1	1	13.6	0.04	0.05
1	0	0	100	3.53	1	77	16.7	142.5	85.5	194	70	124	12.6	5.5	66.7	2	0	0	0	1	1	1	37	0.09	61.2
1	0	0	100	6.85	1	75	24.3	146	97.5	212	70	142	15.1	5.5	71.0	2	1	0	0	1	1	1	18.5	0.07	113
1	0	0	100	7.02	1	73	19.6	153	84.5	211	70	141	13	5.4	68.6	2	1	0	0	1	1	1	155.8	0.07	93.7
1	0	0	100	5.08	1	75	21.6	128	76.5	235	70	165	14.5	5.4	67.7	2	0	0	1	0	1	1	25.7	0.04	12.1
1	0	0	100	4.63	1	66	23.7	126.5	72.5	177	70	107	13.4	5.3	72.1	2	1	0	0	1	1	1	8.8	0.03	43.7
1	0	0	100	4.64	1	71	24.7	150	87	184	70	114	14.6	6.8	61.3	2	1	0	0	1	0	1	6.2	0.06	8.35
1	0	0	100	6.98	1	69	24.6	143.5	81	211	70	141	14.9	5.3	83.4	2	1	0	0	1	0	1	21.4	0.04	16.1
1	0	0	100	4.62	1	73	21.4	123.5	67.5	246	70	176	15.1	5.3	76.1	2	0	0	1	0	1	1	10.7	0.03	8.74
1	0	0	100	4.62	1	67	19.2	121.5	67.5	205	70	135	13.4	5.3	71.6	2	1	0	1	1	1	1	11.6	0.02	20.6
1	0	0	100	5.06	1	66	20.3	130	87	156	70	86	14.7	5.1	79.9	2	0	0	1	1	1	1	6.6	0.03	7.77
1	0	0	100	6.87	1	69	19.7	147.5	79.5	210	70	140	14	5.8	78.3	2	1	0	0	1	1	1	30.7	0.04	13.2
1	0	0	100	5.04	1	67	27.9	130.5	77.5	170	70	100	14.4	5.2	75.2	2	0	0	0	1	1	1	30.7	0.02	50.7
1	1	0	100	6.38	1	67	22.3	129	83	261	70	191	13.5	6.8	71.6	2	1	0	1	0	0	1	1132.7	0.38	25.1
1	1	0	100	6.48	1	71	25.7	128	85	244	70	174	16.1	5.6	77.2	1	1	0	0	0	1	1	10.7	0.05	0.05
1	0	0	100	7.07	2	75	19	114.5	63.5	155	70	85	12.3	5.2	72.7	2	1	1	1	1	1	1	22.4	0.32	0.05
1	0	0	100	6.87	2	68	25.5	165	90	269	70	199	14.2	8.2	76.4	2	1	1	0	0	0	1	117.6	0.13	6.78
1	0	0	100	6.87	2	72	19.4	129	74.5	231	70	161	11.6	5.8	78.9	2	1	0	1	0	1	1	16	0.15	39.5
1	0	0	100	6.85	2	67	24.8	141.5	73.5	224	70	154	12.7	5.1	62.2	2	1	1	0	0	1	1	5.3	0.14	27.2
1	0	0	100	7.04	2	74	22.3	114.5	69	204	70	134	12.6	5.1	59.2	2	1	1	1	1	1	1	9.4	0.57	27.7

1	0	0	100	6.83	2	73	19	138.5	85.5	227	70	157	13.4	5.4	70.1	2	1	0	1	0	1	1	86.6	0.15	17.9
1	0	0	100	6.44	2	70	25.9	110	68.5	197	70	127	14.7	5.8	75.3	2	1	1	1	1	1	1	54.6	0.45	16.6
1	0	0	100	7	2	66	24.3	114	75	218	70	148	12.3	5.6	62.7	2	1	1	1	0	1	1	18.8	0.03	11.8
1	0	0	100	4.58	2	73	24.2	105	58	211	70	141	12.7	5.6	73.8	2	1	1	1	1	1	1	55.8	0.02	33.6
1	0	0	100	4.58	2	74	22.8	155.5	99.5	194	70	124	12.8	5.4	69.6	2	1	1	0	1	1	1	45.8	0.04	19.6
1	0	0	100	6.86	2	78	21.5	156.5	91	191	70	121	14.2	4.9	67.7	2	1	1	0	1	1	1	36	0.02	76
1	0	0	100	6.86	2	72	23.4	144	76	192	70	122	12.5	5.4	74.3	2	1	1	0	0	1	1	16.5	0.02	75.8
1	0	0	100	6.87	2	67	20.3	127	80.5	212	70	142	13.5	5.8	73.1	2	1	1	1	1	1	1	9.5	0.02	39.8
1	0	0	100	6.87	2	77	18.4	130	72	246	70	176	13.3	5.4	58.0	2	1	1	1	0	1	1	33.3	0.08	21.6
1	0	0	100	6.87	2	73	21.6	117.5	74	198	70	128	12.6	5.1	73.8	2	1	1	1	1	1	1	39.2	0.05	27.3
1	0	0	100	6.88	2	65	20.3	135.5	75	207	70	137	14.5	5.2	54.7	2	1	0	1	1	1	1	9.1	0.03	29.6
1	0	0	100	5.16	2	70	25.4	138	76.5	218	70	148	11.3	5.7	60.9	1	1	1	0	1	1	1	9.5	0.03	15.6
1	0	0	100	6.85	2	66	20	131	64.5	203	70	133	11.8	5.7	77.5	2	1	1	0	1	1	1	10.6	0.01	23.9
1	0	0	100	6.98	2	66	21.1	146.5	79.5	245	70	175	10.6	5.5	77.5	2	1	1	0	0	1	1	25.9	0.01	16.8
1	0	0	100	4.62	2	72	19.4	117	64.5	218	70	148	11.7	5.7	78.9	2	1	1	1	1	1	1	17.3	0.04	149
1	0	0	100	4.63	2	70	22.2	127	77	198	70	128	13.1	5.4	80.0	2	1	1	0	1	1	1	19.7	0.01	34.9
1	0	0	100	4.63	2	67	25.3	137	79	259	70	189	15.5	5.8	73.1	2	1	0	1	0	1	1	7.3	0.04	21.3
1	0	0	100	6.96	2	73	23.6	109	70	208	70	138	12.9	5.7	70.1	1	1	1	0	0	1	1	28	0.03	32.1
1	0	0	100	6.8	2	70	25.9	101	58	179	70	109	12.6	5	80.0	2	1	1	1	1	1	1	13.8	0.02	69.4
1	0	0	100	6.83	2	68	20.7	130	76.5	191	70	121	13	5.2	72.6	2	1	1	1	1	1	1	15.4	0.01	10.6
1	0	0	100	4.6	2	70	21.2	148.5	81.5	158	70	88	10.8	5	75.3	2	1	1	0	1	1	1	34.5	0.02	51.4
1	0	0	100	4.6	2	69	22.6	125.5	76.5	175	70	105	13.7	5.2	75.9	2	1	1	1	1	1	1	12.6	0.09	12.9
1	0	0	100	4.62	2	72	25.5	139	66	229	70	159	14.9	5.8	70.6	2	1	1	0	0	1	1	174.4	0.04	47
1	0	0	100	4.62	2	70	25.3	135	76.5	173	70	103	12.5	5.2	75.3	2	1	1	0	1	1	1	23.1	0.03	37.7
1	0	0	100	5.05	2	71	20.3	136	74	214	70	144	13	5.6	74.8	1	1	1	0	0	1	1	81.3	0.04	35.7
1	0	0	100	5.07	2	65	23.1	137.5	84	235	70	165	12.7	5.8	74.2	2	1	1	0	0	1	1	25.1	0.05	41.9
1	0	0	100	5.07	2	66	18.2	97	60	178	70	108	12.7	5.3	82.3	2	1	1	1	1	1	1	11.8	0.02	5.53
1	0	0	100	6.87	2	65	24.2	124.5	70	285	70	215	12.9	8	82.8	2	1	1	1	0	0	1	18.6	0.06	5.03
1	0	0	100	5.04	2	69	23	104	60	172	70	102	11.9	5.6	75.9	2	1	1	1	1	1	1	25.8	0.01	17.2
1	0	0	100	5.04	2	74	19.9	80	46.5	166	70	96	12.5	5.5	73.2	2	1	1	1	1	1	1	14.8	0.06	18
1	1	0	100	7	2	73	20.5	114	65.5	228	70	158	11.3	5.4	78.3	2	1	1	0	0	1	1	6.5	2.26	29.9
1	1	0	100	7.04	2	75	22.8	137.5	79.5	172	70	102	10.8	5.2	69.1	1	1	0	0	1	1	1	49	0.05	25.1
1	1	0	100	6.95	2	73	26	132.5	63.5	222	70	152	11.7	5.9	73.8	2	1	1	1	0	1	1	23.7	0.06	83.4
1	1	0	100	6.84	2	72	21.9	157.5	89.5	206	70	136	11.4	5.2	60.1	2	1	1	0	1	1	1	131.5	0.02	41.2
1	0	0	100	4.63	2	80	23.8	141	83.5	256	70	186	12.8	5.4	66.7	1	1	1	0	0	1	1	14.8	0.27	39.8
1	0	0	100	6.86	1	67	27.5	121	75.5	235	70	165	16.8	8.7	79.4	1	1	1	0	0	0	1	5.2	0.22	28.3
1	0	0	100	7.04	1	72	19.9	109.5	63.5	169	70	99	14.1	4.8	76.7	2	1	1	1	1	1	1	5.4	0.35	10.3
1	0	0	100	6.88	1	67	27.5	141.5	83	187	70	117	17.2	5.9	71.6	2	0	0	0	1	1	1	18.4	0.06	28.2
1	0	0	100	5.06	1	77	24.1	136	87.5	198	70	128	15.2	5.8	66.7	2	0	0	1	1	1	1	36.5	0.01	56.3
1	0	0	100	7	1	69	23.9	145	81.5	218	70	148	16.2	5.6	78.3	2	0	0	0	1	1	1	12.5	0.07	4.27
1	0	0	100	7.08	2	69	29.3	150	87	170	70	100	12.5	5.7	75.9	2	1	0	0	1	1	1	370.2	0.4	30.8
1	0	0	100	6.87	2	68	31.9	159.5	77	218	70	148	15.4	6.3	72.6	1	1	1	0	1	0	1	81.8	0.15	12.8
1	0	0	100	5.04	2	76	24.2	110.5	71	159	70	89	13.6	5.4	72.2	1	1	1	1	1	1	1	7.6	0.14	26.1
1	0	0	100	7	2	70	31.3	148.5	84.5	218	70	148	12.3	5.4	75.3	2	1	1	0	0	1	1	85.5	0.05	28.9
1	0	0	100	4.59	2	66	22.8	129	79	211	70	141	13	5.5	73.6	2	1	1	1	1	1	1	25.3	0.02	35.8
1	0	0	100	6.87	2	77	21.7	118.5	59.5	234	70	164	10.9	5.6	68.2	1	1	1	1	0	1	1	11.1	0.01	50.7
1	0	0	100	5.15	2	66	29.6	110.5	66.5	200	70	130	13.1	6.2	77.5	1	1	1	1	1	1	1	2.9	0.07	17.2
1	0	0	100	5.16	2	65	22.5	109	65.5	172	70	102	12.6	5.5	74.2	2	1	1	0	1	1	1	9.4	0.01	48.5
1	0	0	100	6.85	2	66	18.3	111	60	207	70	137	12.9	5	77.5	2	1	1	1	1	1	1	13.9	0.03	55.7
1	0	0	100	5.05	2	67	21.4	132	74	196	70	126	12.8	5.4	81.7	2	1	1	0	1	1	1	200.7	0.01	14.1
1	0	0	100	6.98	2	72	25	145	78.5	244	70	174	12.4	5.7	74.3	2	1	1	0	0	1	0	41.5	0.09	46.1

1	0	0	100	4.64	2	66	27.7	103	64	178	70	108	11.8	5.9	82.3	2	1	1	1	1	1	1	34.2	0.05	12.5
1	0	0	100	6.95	2	71	28.5	115	69	189	70	119	15.1	5.8	71.1	1	1	1	0	1	1	1	36.6	0.06	52
1	0	0	100	6.95	2	65	17.3	98	56.5	171	70	101	11.3	7.7	82.8	2	1	1	1	1	0	1	7.4	0.04	26.5
1	0	0	100	6.98	2	67	32.9	146	77.5	258	70	188	13.7	5.3	81.7	2	1	1	0	0	1	1	70.2	0.06	25.3
1	0	0	100	6.98	2	79	21.1	136.5	73	183	70	113	12.6	5.5	70.7	2	1	1	1	1	1	1	48.8	0.02	8.79
1	0	0	100	3.58	2	69	19.3	120	68.5	196	70	126	12.9	4.8	72.1	2	1	1	1	1	1	1	18.3	0.01	74.6
1	0	0	100	4.59	2	70	21.6	162.5	84	211	70	141	13.6	5.3	80.0	2	1	1	0	1	1	1	20.5	0.03	31.9
1	0	0	100	4.62	2	76	18	121	63	177	70	107	11.4	5.2	68.6	2	1	1	0	1	1	1	65.5	0.04	106
1	0	0	100	4.62	2	69	17.4	143	81.5	228	70	158	9.1	5.9	86.7	2	1	1	0	0	1	1	54.1	0.03	80.5
1	0	0	100	4.62	2	70	19.9	137	74.5	177	70	107	12.6	5.8	71.6	2	1	1	1	1	1	1	8.2	0.01	131
1	0	0	100	6.45	2	69	29.7	112	58.5	182	70	112	13.3	5.6	75.9	1	1	1	1	0	1	1	15.8	0.06	84.2
1	0	0	100	6.85	2	73	21.6	158	86.5	227	70	157	13.8	5.8	70.1	2	1	1	0	0	1	1	86.7	0.03	28.7
1	0	0	100	0.83	2	68	26.4	143.5	86.5	207	70	137	12.6	5.4	76.4	1	1	1	0	1	1	1	14.8	0.07	17
1	1	0	100	5.37	2	67	33.9	137.5	92.5	178	70	108	13.1	6.2	73.1	2	1	0	0	1	1	1	196.5	0.14	11.5
1	1	0	100	5.16	2	72	20.3	113	73.5	172	70	102	13.7	6	74.3	2	1	1	0	0	1	1	21.8	0.02	25.3
1	1	0	100	6.84	2	75	24.4	125.5	64.5	204	70	134	13.2	5.4	72.7	2	1	1	0	1	1	1	10.8	0.05	6.4
1	1	0	100	6.86	2	70	30.9	160	93.5	214	70	144	13.7	5.4	75.3	2	1	1	0	1	1	1	204.1	0.09	9.37
1	1	0	100	4.64	2	74	23.1	126	72.5	194	70	124	11.5	5.3	73.2	2	1	1	1	0	1	1	15.8	0.05	12.4
1	0	0	100	6.95	2	81	27.2	141.5	89	221	70	151	13.2	5.5	74.0	1	1	1	0	0	1	1	29.6	0.04	15.2
1	1	0	100	5.04	2	85	29.7	110.5	68.5	209	70	139	15.4	6.9	47.6	1	1	0	0	1	0	1	21.4	0.25	66.7
1	1	0	100	4.62	2	81	25.8	115	65	231	70	161	12.8	6.8	69.7	2	1	1	1	0	0	1	46.8	0.05	35.6
1	0	1	0.6	0.6	1	75	26.5	136	81	207	70	137	15.8	6.5	75.1	2	0	0	0	1	0	1	33.1	0.02	7.11
1	0	1	3.7	3.7	2	72	23.4	142	79.5	229	70	159	12.7	6.3	60.1	2	1	1	0	0	1	1	26.5	0.17	33.8
1	0	1	0.5	0.5	2	71	28.2	154.5	79	231	70	161	12.9	5.2	74.8	1	1	1	0	0	1	1	45.6	0.08	98.8
1	1	1	4.2	4.2	2	76	18.2	122.5	81	195	70	125	13	4.9	72.2	1	1	1	0	1	1	1	63.3	0.03	43.7
1	1	1	2.5	2.5	2	74	26.7	168.5	84.5	245	70	175	14.7	8.9	59.2	1	1	1	0	0	0	1	721.9	0.04	114
1	1	1	2.9	2.9	2	71	20	117.5	75	190	70	120	12.4	5.4	71.1	2	1	1	0	1	1	1	10.7	0.03	11.4
1	0	0	100	7.04	1	66	24.3	139	86.5	165	71	94	14.8	6.2	79.9	1	0	0	1	1	1	1	6.7	0.16	6.84
1	0	0	100	5.05	1	68	26	147.5	90	207	71	136	13	5.1	50.2	2	1	1	0	1	1	1	11.4	0.69	8.36
1	0	0	100	5.08	1	71	21.9	109.5	66.5	192	71	121	14.9	5.5	82.2	1	0	1	1	1	1	1	11.8	0.16	12.8
1	0	0	100	6.87	1	67	27.3	109.5	82.5	300	71	229	16.2	4.6	56.2	2	1	0	1	0	1	1	4.4	2.2	11.2
1	0	0	100	7	1	69	23.2	123.5	73	207	71	136	14.6	5.6	74.1	2	1	0	1	0	1	1	9	0.02	4.38
1	0	0	100	5.07	1	66	22.3	96.5	61	228	71	157	13.7	5.2	79.9	2	1	0	1	0	1	1	4.6	0.01	13.6
1	0	0	100	5.07	1	74	28.1	125	67	186	71	115	15.5	5.3	75.6	1	0	0	0	1	1	1	5.9	0.05	60.3
1	0	0	100	4.63	1	71	23.3	142.5	71.5	186	71	115	14.5	5.8	88.6	2	0	0	0	1	1	1	36	0.02	15
1	0	0	100	6.87	1	68	22.2	123	78	196	71	125	14.7	5.2	74.6	2	1	0	0	1	1	1	4.5	0.05	47.2
1	0	0	100	6.95	1	74	26	145	79.5	259	71	188	16.7	5.8	75.6	2	1	0	0	0	1	1	24.3	0.02	7.59
1	0	0	100	6.95	1	67	32.5	135	78.5	196	71	125	16.7	5	63.0	2	1	1	1	1	1	1	55.8	0.02	12.3
1	0	0	100	6.97	1	68	23.5	142	82.5	171	71	100	15.5	5	84.0	2	1	0	0	1	1	1	15.9	0.07	38.2
1	0	0	100	6.98	1	65	23.8	120	68	239	71	168	14.2	5.5	80.5	2	1	0	0	0	1	1	9.5	0.04	28.5
1	0	0	100	4.68	1	73	18.4	96	54	218	71	147	12.8	5.5	76.1	2	1	1	1	1	1	1	22.4	0.01	30.5
1	0	0	100	4.69	1	69	20.6	126	70.5	200	71	129	15	5.6	74.1	2	1	1	1	1	1	1	20.1	0.04	13.3
1	1	0	100	3.42	1	74	23.7	134.5	72.5	163	71	92	15	5.4	68.2	2	1	1	0	1	1	1	35.3	0.1	20.4
1	0	0	100	7.07	2	69	26.3	101	58	236	71	165	12.8	5.5	72.1	2	1	1	1	0	1	1	33.9	0.13	26.8
1	0	0	100	7.08	2	66	22.4	146.5	91.5	239	71	168	13.8	6.2	88.5	2	1	1	0	0	1	1	46.1	0.13	25.7
1	0	0	100	6.86	2	73	31.1	129	78	190	71	119	12.5	4.9	59.7	2	1	1	1	1	1	1	31.3	0.17	59.9
1	0	0	100	7.21	2	73	25.8	129.5	80.5	201	71	130	12.8	5.1	70.1	2	1	1	0	0	1	1	13.6	0.11	25.4
1	0	0	100	6.86	2	65	24	104.5	63	260	71	189	13.2	5.1	74.2	2	1	0	1	0	1	1	22.7	0.15	17.6
1	0	0	100	7.04	2	71	23.9	170	83.5	229	71	158	14.6	5.3	74.8	1	1	1	0	0	1	1	5	0.11	1.02
1	0	0	100	6.96	2	69	27.1	124	75	224	71	153	14	5.1	80.5	1	1	1	0	0	1	1	31.5	0.23	30.5
1	0	0	100	4.59	2	67	20.8	109	66.5	202	71	131	13.5	5.4	76.9	2	1	1	1	1	1	1	27.9	0.16	48.4

1	0	0	100	4.62	2	78	23.9	174.5	97	268	71	197	13.3	5.3	71.2	1	1	1	0	0	1	1	415.1	0.23	54.4
1	0	0	100	5.05	2	69	31.1	149.5	83	193	71	122	14.4	5	75.9	2	1	0	0	1	1	1	21.3	0.23	70.9
1	0	0	100	6.87	2	70	20.9	152.5	96	183	71	112	13.7	6	75.3	2	1	1	0	1	1	1	11.7	0.11	18.6
1	0	0	100	7.06	2	66	22.9	150	71.5	179	71	108	11.2	4.9	62.7	2	1	1	0	1	1	1	13.6	0.01	28.2
1	0	0	100	7.08	2	68	18.8	146	77	204	71	133	14.1	5.1	81.1	2	1	1	0	1	1	1	39.9	0.02	32
1	0	0	100	6.86	2	75	23.3	157	75	186	71	115	12.1	5.2	69.1	2	1	1	0	1	1	1	19.9	0.04	108
1	0	0	100	6.87	2	65	20.6	133	78	265	71	194	13.9	5.5	74.2	2	1	1	1	0	1	1	25	0.04	17.4
1	0	0	100	5.16	2	78	18.4	113.5	61	218	71	147	14.5	5.3	71.2	1	1	1	1	0	1	1	28.3	0.03	15.8
1	0	0	100	6.84	2	66	24.6	95.5	67	251	71	180	13.9	6.4	77.5	2	1	1	1	0	1	1	13.2	0.07	5.51
1	0	0	100	6.84	2	69	22.7	139	79	240	71	169	14.6	5.6	80.5	2	1	1	0	0	1	1	10	0.03	34.6
1	0	0	100	6.85	2	66	23.6	130	80.5	162	71	91	12.4	5.9	82.3	2	1	0	1	1	1	1	8.4	0.04	74.6
1	0	0	100	7.02	2	67	21.2	107	63.5	187	71	116	12.5	5.3	81.7	2	1	1	1	1	1	1	17.3	0.01	22.9
1	0	0	100	5.06	2	71	22.3	129	73.5	194	71	123	11.5	5.7	74.8	1	1	1	0	1	1	1	134.6	0.03	57.8
1	0	0	100	5.06	2	65	25.9	130	77	168	71	97	13.2	5.2	82.8	2	1	1	1	1	1	1	45.1	0.03	27.2
1	0	0	100	5.07	2	72	28.8	136	73	229	71	158	14	5.1	74.3	2	1	1	0	0	1	1	55.3	0.04	17.1
1	0	0	100	5.08	2	65	22.4	117.5	70.5	204	71	133	13.1	5.4	78.0	2	1	1	1	1	1	1	10.3	0.05	36.8
1	0	0	100	4.62	2	65	20.6	97.5	53.5	210	71	139	12.4	5.7	78.0	2	1	1	1	1	1	1	12.2	0.04	17.7
1	0	0	100	4.63	2	66	21.3	107	60.5	204	71	133	13.4	5.6	77.5	2	1	1	1	1	1	1	17.1	0.02	21.5
1	0	0	100	6.98	2	74	24.2	127.5	67	216	71	145	12.8	5.5	73.2	1	1	1	1	1	1	1	26.2	0.03	51.4
1	0	0	100	6.8	2	69	21.1	106	62.5	186	71	115	12.4	5.1	75.9	2	1	1	1	1	1	1	25.1	0.06	11.2
1	0	0	100	6.8	2	76	21.4	105.5	66	212	71	141	11.4	5.5	68.6	2	1	1	1	1	1	1	22.6	0.06	70
1	0	0	100	4.6	2	65	22.2	130.5	77	219	71	148	12.5	5.8	63.1	2	1	1	0	1	1	1	15.2	0.06	47.8
1	0	0	100	4.62	2	77	25.1	155	97.5	201	71	130	15.6	5.5	58.0	2	1	1	0	1	1	1	43.8	0.02	18.8
1	0	0	100	4.67	2	71	30.1	164.5	107	243	71	172	12.6	5.1	71.1	2	1	1	0	0	1	1	93	0.07	47.4
1	0	0	100	5.05	2	73	21.4	121.5	78	198	71	127	13.6	5.3	78.3	1	1	1	0	1	1	1	5.5	0.04	16
1	0	0	100	6.87	2	68	21.7	97	63	221	71	150	14.1	5.1	72.6	1	1	1	1	0	1	1	24.2	0.01	20.1
1	0	0	100	5.04	2	66	26.3	133	83	202	71	131	12.9	5.3	82.3	2	1	1	0	1	1	1	46.6	0.06	15.5
1	0	0	100	5.04	2	68	23.1	93	62	173	71	102	12.7	5.6	72.6	2	1	1	1	0	1	1	3.1	0.05	10.4
1	1	0	100	4.41	2	67	24	107	63.5	189	71	118	13	4.9	81.7	2	1	0	1	1	1	1	23.2	0.37	13.8
1	1	0	100	7.08	2	73	18	115	69	216	71	145	14.4	4.8	73.8	2	1	1	1	1	1	1	12.7	0.01	33.9
1	1	0	100	6.79	2	75	20.5	114.5	66	210	71	139	13	5.6	72.7	2	1	1	1	1	1	1	22.7	0.03	7.16
1	1	0	100	5.05	2	65	20.7	139.5	97	231	71	160	13.5	5.6	78.0	2	1	1	0	0	1	1	8	0.01	18.3
1	1	0	100	6.17	1	87	21.4	131	68.5	174	71	103	13.5	4.9	69.0	1	1	0	1	1	1	0	13.4	0.03	149
1	1	0	100	7	2	80	23.5	133.5	66	219	71	148	12.5	5.2	66.7	2	1	1	1	1	1	1	8.5	0.03	9.39
1	0	0	100	5.04	1	76	22.8	121	70	176	71	105	12.5	5.2	70.5	2	1	1	1	1	1	1	16.4	0.04	26.6
1	1	0	100	4.69	1	78	22.9	145	78.5	176	71	105	14.1	5	69.6	2	1	0	0	1	1	1	7	0.07	58.1
1	0	0	100	6.86	2	74	29.1	114	63.5	230	71	159	13.5	6.2	73.2	1	1	0	0	0	1	1	19.2	0.22	6.16
1	0	0	100	5.04	2	78	26.4	138.5	68	226	71	155	11.8	5.6	67.7	2	1	1	1	0	1	1	8.8	0.1	96
1	0	0	100	7	2	66	27.7	144.5	80.5	254	71	183	13.3	5.1	77.5	2	1	1	0	0	1	1	19.7	0.04	25.7
1	0	0	100	7.06	2	70	24.6	138	77	230	71	159	12.8	5.4	75.3	2	1	1	1	0	1	1	28.3	0.03	30.7
1	0	0	100	7.07	2	72	23.9	107.5	64.5	209	71	138	11.8	5.6	70.6	2	1	1	1	1	1	1	20.7	0.03	23.6
1	0	0	100	7.07	2	73	20.6	106.5	66.5	227	71	156	11.9	5.2	78.3	2	1	1	1	0	1	1	21.1	0.01	0.05
1	0	0	100	4.58	2	65	23.7	114.5	65.5	268	71	197	15.7	5.3	78.0	2	1	1	1	0	1	1	10.2	0.03	19.7
1	0	0	100	5.02	2	78	21.8	106	60	216	71	145	12.6	6	57.6	1	1	1	1	1	1	1	14.8	0.05	24.3
1	0	0	100	4.59	2	77	22.6	146.5	77.5	213	71	142	13	5.1	71.7	1	1	1	0	1	1	1	19.5	0.03	217
1	0	0	100	6.87	2	70	21.5	146	81.5	229	71	158	13.7	5.5	80.0	2	1	1	0	0	1	1	4.5	0.01	48.3
1	0	0	100	5.15	2	66	27.7	130	72.5	209	71	138	14.4	5.9	82.3	2	1	1	1	1	1	1	17.4	0.04	11.8
1	0	0	100	7.21	2	75	18.5	99	63.5	222	71	151	13.6	5.6	77.2	2	1	1	1	0	1	1	26.5	0.07	43
1	0	0	100	7.01	2	69	19.2	142.5	74.5	217	71	146	12.6	5.1	80.5	2	1	1	0	1	1	1	7.1	0.01	10.7
1	0	0	100	7.02	2	70	26.6	144	78.5	201	71	130	12.5	5.7	60.9	1	1	1	0	1	1	1	5.2	0.03	39.2
1	0	0	100	5.05	2	66	18.2	97	52	195	71	124	11.7	5.1	73.6	2	1	1	1	1	1	1	3.2	0.01	71.2

1	0	0	100	6.62	2	72	25.2	131	71	276	71	205	14.9	5.6	70.6	2	1	1	1	0	1	1	6.1	0.04	27.9
1	0	0	100	4.59	2	74	21.6	132	70.5	183	71	112	12.4	5.4	77.8	1	1	1	0	1	1	1	35	0.03	75.3
1	0	0	100	2.48	2	79	23.2	138	91	226	71	155	13.4	5.3	67.2	1	1	1	0	0	1	1	12.6	0.02	37.4
1	0	0	100	6.44	2	76	28.1	131.5	74.5	257	71	186	12.6	5.4	68.6	2	1	1	1	0	1	1	27.5	0.09	14.8
1	0	0	100	5.36	2	68	24	139	83	259	71	188	12.5	5.7	76.4	2	1	1	1	0	1	1	9.7	0.06	79.9
1	0	0	100	5.06	2	72	24.5	144.5	84.5	186	71	115	11.8	5.8	70.6	1	1	1	0	1	1	1	24.1	0.01	1.69
1	1	0	100	4.64	2	79	22.4	109.5	61.5	206	71	135	12.3	6.8	70.7	2	1	1	1	0	0	1	11.5	0.03	25.3
1	1	0	100	6.98	1	90	19.6	131	53.5	174	71	103	13.3	5.1	43.0	1	1	1	0	1	1	1	958.9	0.01	101
1	0	1	2.2	2.2	2	65	23.9	152	89.5	205	71	134	14.2	4.6	78.0	2	1	1	0	1	1	1	17	0.24	0.05
1	0	0	100	7.07	1	68	23.9	126.5	72	183	72	111	14.6	7.6	50.2	2	1	1	0	1	0	1	13.5	0.36	16.2
1	0	0	100	6.85	1	68	25.1	121	74	185	72	113	15.6	6.3	62.6	2	0	0	0	1	1	0	91.5	0.18	405
1	0	0	100	5.04	1	71	25.3	169	93	182	72	110	12.2	6	88.6	2	1	0	0	1	1	1	21.8	0.13	34.2
1	0	0	100	2.7	1	79	26.8	127	84.5	265	72	193	13.9	6.2	65.8	2	1	0	1	0	1	1	14	0.05	16.7
1	0	0	100	7.08	1	69	22.4	110	68.5	238	72	166	12	5.5	70.6	2	0	0	1	0	1	1	8.9	0.04	24.7
1	0	0	100	7.02	1	69	21.4	122.5	77	165	72	93	13	5.6	78.3	2	0	0	0	1	1	1	5.6	0.02	31.2
1	0	0	100	7.02	1	71	20.1	111.5	60	210	72	138	10.9	5.8	61.3	2	1	0	1	1	1	1	5.5	0.01	11.7
1	0	0	100	7.04	1	66	23.5	167.5	93	203	72	131	15.1	5.7	79.9	2	0	1	0	1	1	1	5.8	0.01	0.05
1	0	0	100	5.06	1	70	19	159.5	85	222	72	150	14.9	5.3	61.7	2	1	1	0	0	1	1	16.5	0.01	13.4
1	0	0	100	4.64	1	72	28.8	132.5	78	192	72	120	15	6.1	72.6	2	1	0	1	1	1	1	9.3	0.05	32.5
1	0	0	100	6.83	1	66	24.3	163.5	101	212	72	140	16.5	5.1	75.7	2	1	0	0	1	1	1	10.2	0.03	22.5
1	0	0	100	6.96	1	66	20.6	93.5	63	184	72	112	11.9	5.3	85.2	2	1	0	1	1	1	1	12.6	0.01	0.72
1	0	0	100	5.37	1	70	19.7	146	82	167	72	95	13.7	5.4	70.1	1	1	0	0	1	1	1	18.1	0.03	15.3
1	0	0	100	6.86	1	74	25.6	141	78.5	161	72	89	15.6	5.5	71.5	2	1	0	0	1	1	1	11.7	0.03	84
1	0	0	100	7.05	2	69	22	143.5	72.5	231	72	159	13.9	5.7	72.1	2	1	1	0	0	1	1	59.8	0.15	4.67
1	0	0	100	4.58	2	67	24.4	155.5	88.5	230	72	158	12.8	5.4	62.2	2	1	1	0	0	1	1	18.3	0.34	27.5
1	0	0	100	6.87	2	70	27.3	106.5	65.5	227	72	155	13.2	6.3	60.9	2	1	0	1	0	1	1	16.9	0.11	53.3
1	0	0	100	6.84	2	70	28.7	158	107	257	72	185	15.3	5.4	75.3	2	1	1	0	0	1	1	899.8	0.15	31.3
1	0	0	100	4.68	2	72	21.4	123	64.5	196	72	124	14.5	5.3	74.3	1	1	0	1	1	1	1	17.3	0.12	28.8
1	0	0	100	7	2	72	22.2	160.5	81	231	72	159	14	5.4	78.9	1	1	1	0	0	1	1	10.7	0.01	24.2
1	0	0	100	7	2	65	24.1	138	91	155	72	83	13.8	5.1	78.0	2	1	1	0	1	1	1	18.6	0.04	33.4
1	0	0	100	7.07	2	70	25.3	133.5	76	202	72	130	12.8	5.6	75.3	1	1	1	1	1	1	1	14.9	0.06	20.2
1	0	0	100	7.07	2	70	18.6	121	79	201	72	129	11.8	5.3	75.3	2	1	1	1	1	1	1	36.3	0.01	47.3
1	0	0	100	7.07	2	67	25.9	111.5	74.5	253	72	181	14	5.6	73.1	2	1	0	1	0	1	1	15.2	0.08	24.7
1	0	0	100	6.86	2	70	21.2	104	54	183	72	111	12.2	5.1	71.6	2	1	1	0	1	1	1	43.4	0.03	7.38
1	0	0	100	6.87	2	67	21.3	101	62	223	72	151	13.8	5.3	76.9	2	1	1	1	0	1	1	12.9	0.04	7.62
1	0	0	100	6.87	2	66	21	152	83	184	72	112	11.8	5.5	77.5	2	1	1	0	1	1	1	22.3	0.02	25.1
1	0	0	100	6.88	2	69	24.3	165.5	87.5	198	72	126	12.5	5.4	75.9	2	1	1	0	1	1	1	42.1	0.03	72.9
1	0	0	100	5.15	2	75	23.1	154	95	258	72	186	14.1	5.7	72.7	2	1	0	0	0	1	1	29.2	0.03	6.2
1	0	0	100	6.84	2	72	22.6	122	72.5	242	72	170	14.3	5.3	74.3	2	1	1	1	0	1	1	16.9	0.04	25.1
1	0	0	100	6.86	2	66	26.9	133.5	76.5	254	72	182	13.5	5.2	73.6	2	1	1	1	0	1	1	5.6	0.05	73.8
1	0	0	100	7.01	2	71	21.8	124.5	71.5	188	72	116	13.1	4.9	74.8	1	1	1	1	1	1	1	17.6	0.01	43.7
1	0	0	100	7.01	2	69	23.4	106.5	69	227	72	155	12.9	5.1	75.9	2	1	1	0	0	1	1	26.9	0.04	35.9
1	0	0	100	7.02	2	74	18.1	131.5	74.5	214	72	142	12	6.2	77.8	1	1	1	1	1	1	1	66.2	0.01	21.8
1	0	0	100	5.06	2	69	24.3	136.5	74	207	72	135	13.2	6	80.5	2	1	1	1	1	1	1	48.9	0.09	8.32
1	0	0	100	5.07	2	72	28	109.5	63.5	238	72	166	12	5.6	60.1	1	1	0	1	0	1	1	7	0.02	46.3
1	0	0	100	4.64	2	71	20	118.5	72	183	72	111	12.9	6.3	60.5	1	1	1	0	1	1	1	37.5	0.07	34.3
1	0	0	100	6.87	2	71	21	102.5	61.5	203	72	131	13.2	5.3	71.1	2	1	1	1	1	1	1	6.6	0.03	3.48
1	0	0	100	6.96	2	67	24.5	92	59	250	72	178	13	5.1	76.9	1	1	0	1	0	1	1	12.1	0.01	8.93
1	0	0	100	6.98	2	66	19.8	151	82.5	194	72	122	11.7	5.2	77.5	2	1	1	0	1	1	1	12.9	0.01	158
1	0	0	100	6.98	2	65	26.3	162.5	86	231	72	159	12.9	5.3	82.8	2	1	1	0	0	1	1	65.3	0.03	24.8
1	0	0	100	6.8	2	66	20.3	98.5	68	285	72	213	13.2	6.1	73.6	2	1	1	1	0	1	1	22.2	0.04	4.02

1	0	0	100	6.84	2	73	23.7	121	65.5	213	72	141	12.2	5.4	70.1	2	1	1	1	1	1	0	13.6	0.06	76.5
1	0	0	100	4.59	2	74	21.7	105	61	225	72	153	12.8	5.2	73.2	2	1	1	1	0	1	1	25.9	0.05	31.2
1	0	0	100	4.59	2	65	25.5	96	62.5	176	72	104	13.5	5.2	74.2	2	1	1	1	1	1	1	15.9	0.02	18.2
1	0	0	100	4.67	2	65	24.3	138	90	216	72	144	14.4	5.4	82.8	2	1	1	0	1	1	1	265.1	0.04	43.9
1	0	0	100	4.67	2	65	23.7	127	64.5	207	72	135	13.2	5.4	74.2	2	1	1	1	1	1	1	10.8	0.08	56.5
1	0	0	100	4.69	2	66	20.2	143.5	82.5	194	72	122	13.5	5	77.5	2	1	1	0	1	1	1	15.9	0.09	18.3
1	0	0	100	4.7	2	70	23.3	137.5	77.5	200	72	128	14.4	5.5	71.6	2	1	1	1	1	1	1	6.3	0.01	27.3
1	0	0	100	5.37	2	73	21.8	118	62.5	221	72	149	13.2	5.6	73.8	2	1	1	1	0	1	1	12.8	0.02	28.5
1	0	0	100	5.06	2	73	19.4	95.5	55.5	184	72	112	11.3	6.1	73.8	2	1	1	1	1	1	1	5.3	0.01	26.5
1	0	0	100	6.45	2	72	26.5	147	80	205	72	133	14.5	4.8	70.6	2	1	1	0	1	1	1	18.5	0.05	52.1
1	0	0	100	6.86	2	77	25.6	122	71	211	72	139	12.2	5.5	68.2	1	1	1	1	1	1	1	11.8	0.05	36.7
1	0	0	100	6.86	2	65	23	92.5	58	272	72	200	14.3	5.1	74.2	2	1	1	1	0	1	1	8.5	0.04	5.2
1	0	0	100	6.86	2	67	18.2	121.5	66.5	251	72	179	14	5.5	81.7	2	1	1	1	0	1	1	33.5	0.02	35.9
1	0	0	100	6.87	2	65	25.7	129	87.5	220	72	148	12	5.4	74.2	2	1	0	1	0	1	1	24.8	0.09	13.7
1	0	0	100	5.04	2	65	24.5	147.5	83	186	72	114	13.1	5	82.8	2	1	1	0	1	1	1	32.1	0.06	1.5
1	1	0	100	5.06	2	73	23.7	117.5	77	203	72	131	13.2	5.3	73.8	2	1	0	0	1	1	1	21.5	0.12	23.2
1	1	0	100	6.87	2	75	22.9	88.5	52.5	198	72	126	13.4	5.5	69.1	2	1	0	1	0	1	1	6.2	0.33	19.4
1	1	0	100	5.42	2	76	25.3	111.5	66	208	72	136	13.7	5.4	68.6	2	1	1	1	1	1	1	8.1	0.02	0.36
1	1	0	100	6.87	2	75	27	109	59	228	72	156	13	5.7	77.2	1	1	1	0	0	1	1	112.6	0.09	32.4
1	0	0	100	6.03	1	81	22.7	158	96	185	72	113	15.7	4.6	68.1	2	1	0	0	1	1	1	25.3	0.06	63.8
1	1	0	100	6.44	1	82	20.8	166	83.5	159	72	87	12.3	5.2	50.6	2	1	0	0	1	1	1	2.4	0.1	79.1
1	1	0	100	7.07	1	82	24.7	171	87	172	72	100	15.2	5.5	71.5	2	1	0	0	1	1	1	107.9	0.08	79.4
1	1	0	100	5.74	1	82	20	209.5	101.5	189	72	117	13.6	4.8	71.5	1	0	0	0	1	1	1	6.5	0.03	71.4
1	0	0	100	6.85	1	73	23.7	113.5	66.5	155	72	83	14.1	5.2	76.1	2	1	0	1	1	1	1	51.4	0.08	9.13
1	0	0	100	6.98	1	77	19.2	133.5	70.5	166	72	94	12.2	5.1	70.1	1	1	1	0	1	1	1	6.6	0.03	11
1	1	0	100	5.37	1	77	25.9	140	77	196	72	124	15.4	6.1	70.1	1	1	0	0	1	1	1	118.5	0.04	26.8
1	0	0	100	6.86	2	73	21.6	162	81.5	218	72	146	12.2	5.2	73.8	2	1	1	0	1	1	1	25.4	0.63	89.9
1	0	0	100	6.88	2	71	25.1	122.5	77	213	72	141	13.7	7.3	71.1	2	1	1	0	1	0	1	28.1	0.34	92.4
1	0	0	100	4.58	2	72	24.4	90.5	60	214	72	142	12.7	5.5	74.3	2	1	1	1	1	1	1	12.6	0.02	63.2
1	0	0	100	4.58	2	75	21	158	84	178	72	106	12.9	5.3	58.8	2	1	1	0	1	1	1	13.3	0.02	23.3
1	0	0	100	6.87	2	70	24.3	107.5	61.5	260	72	188	11.6	5.4	75.3	1	1	1	1	0	1	1	8.5	0.08	48.6
1	0	0	100	5.16	2	75	25	123.5	70	219	72	147	13.6	5.9	72.7	2	1	1	0	1	1	1	26.6	0.03	29.4
1	0	0	100	6.84	2	66	21.5	109	63.5	223	72	151	13.2	5.3	62.7	2	1	1	1	0	1	1	10.3	0.02	22.4
1	0	0	100	6.84	2	68	30	123	73.5	219	72	147	12.7	5.5	76.4	2	1	1	0	1	1	1	29.1	0.03	59.1
1	0	0	100	6.97	2	65	25.4	120	73	228	72	156	13.9	8.1	82.8	2	1	1	0	0	0	1	16.5	0.03	19.4
1	0	0	100	4.64	2	67	22.2	121	70.5	262	72	190	14.6	5.1	87.9	2	1	1	1	0	1	1	28.6	0.04	25
1	0	0	100	6.98	2	73	26.4	103.5	61	211	72	139	13.5	5	70.1	2	1	0	1	1	1	1	68.2	0.02	13.8
1	0	0	100	6.79	2	75	18.5	161	93.5	174	72	102	12.9	5.5	69.1	2	1	0	0	1	1	1	31.5	0.01	40.2
1	0	0	100	5.37	2	65	25.2	152	78	179	72	107	12.4	5.1	82.8	2	1	1	0	1	1	1	28.7	0.02	21.1
1	0	0	100	5.06	2	65	21.9	133	76.5	186	72	114	13.4	5.3	74.2	2	1	1	1	1	1	1	13.6	0.01	25.6
1	0	0	100	6.86	2	69	23.5	147.5	95.5	212	72	140	10.5	5.4	80.5	1	1	1	0	1	1	1	19.2	0.01	47
1	0	0	100	6.87	2	76	20.6	135.5	76	236	72	164	12	5.2	72.2	2	1	1	1	0	1	1	60.6	0.07	19
1	0	0	100	5.04	2	67	24.9	122	71	158	72	86	11.7	5.5	73.1	2	1	1	1	1	1	1	6.9	0.02	6.05
1	1	0	100	4.64	2	77	25.4	152.5	78	217	72	145	14.6	8	68.2	2	1	1	0	1	0	1	49.6	0.11	26.6
1	1	0	100	6.66	2	69	27.8	145	82	258	72	186	13	5.9	72.1	2	1	1	0	0	1	1	75	0.07	34.5
1	1	0	100	7.07	1	80	25.5	139.5	75.5	208	72	136	13.9	5.6	72.5	1	1	0	0	1	1	1	122.5	0.05	37.7
1	0	0	100	6.88	2	82	25	127	65.5	250	72	178	13.7	5.3	69.2	1	1	1	0	0	1	1	26.9	0.11	28.3
1	0	0	100	3.06	2	82	19.9	136	76	171	72	99	13.6	5.4	69.2	2	1	1	1	1	1	1	39.8	0.08	23.8
1	1	0	100	6.84	2	85	23.9	120.5	63.5	239	72	167	12.2	5.5	64.4	2	1	1	1	0	1	1	33.5	0.02	14.5
1	1	0	100	7.2	2	81	22.3	103.5	60.5	222	72	150	10.9	5.4	66.3	2	1	1	1	0	1	1	10.4	0.06	42.2
1	1	0	100	4.62	2	80	20.6	109.5	58.5	156	72	84	12.6	5.4	70.2	1	1	1	1	1	1	1	21.7	0.01	62.4

1	0	1	0.6	0.6	1	74	18.5	116.5	70	156	72	84	14	5.2	75.6	2	1	0	1	1	1	1	14.4	0.7	84
1	0	1	6.7	6.7	2	66	27	102	65	215	72	143	11.4	5.9	73.6	2	1	1	1	1	1	1	21.7	0.53	10.1
1	0	1	1.7	1.7	2	76	26.5	135.5	79	236	72	164	13.2	5.5	68.6	2	1	1	0	0	1	1	11.2	0.01	13.4
1	0	0	100	6.8	1	67	22.1	91.5	48.5	225	73	152	15.7	5.4	75.2	2	0	1	1	0	1	1	5.1	0.12	16.4
1	0	0	100	7	1	76	20.4	128.5	67.5	185	73	112	12.9	5.2	67.2	2	1	1	1	1	1	1	6	0.04	29.4
1	0	0	100	4.58	1	65	26.9	124	81	202	73	129	15	4.8	80.5	2	1	0	1	1	1	1	42.6	0.09	30
1	0	0	100	6.85	1	75	23.5	134	74.5	174	73	101	14.4	5.3	67.7	2	1	0	0	1	1	1	3.9	0.04	27.4
1	0	0	100	7.01	1	76	25.9	155	82	229	73	156	14.6	5.2	52.7	2	1	0	0	0	1	1	23.9	0.06	23.5
1	0	0	100	4.63	1	69	26	149.5	81	158	73	85	12.8	5.7	74.1	2	1	0	0	1	1	1	4.1	0.04	78.2
1	0	0	100	4.64	1	65	24.2	146	85	175	73	102	14	5.2	72.6	2	0	0	0	1	1	1	2.7	0.03	16.7
1	0	0	100	6.87	1	78	21.5	91	55	220	73	147	11.9	5.2	69.6	2	1	0	1	0	1	1	5.4	0.03	25.8
1	0	0	100	6.95	1	75	24.1	128.5	67.5	185	73	112	13.8	5.8	75.1	2	1	0	1	1	1	1	90.2	0.02	77.6
1	0	0	100	6.82	1	70	22.4	127	71.5	166	73	93	13.5	5.4	77.7	2	1	0	1	1	1	1	20	0.02	12.4
1	0	0	100	4.59	1	70	18.5	113	77	184	73	111	12.8	5.8	55.0	2	1	0	1	1	0	1	8.8	0.02	15.9
1	0	0	100	4.62	1	74	25.2	152	89	204	73	131	14.9	5.2	53.5	2	1	0	0	1	1	1	27.3	0.04	3.16
1	1	0	100	6.98	1	75	26.6	135.5	72.5	205	73	132	15.5	5.5	67.7	2	0	0	1	1	1	1	10.1	0.05	0.05
1	0	0	100	7.07	2	73	27	135	96	243	73	170	13.1	5.5	73.8	2	1	1	0	0	1	1	30.4	0.11	19.5
1	0	0	100	6.85	2	73	25.3	109	62.5	232	73	159	12.9	5.9	73.8	2	1	1	1	0	1	1	10.2	0.29	67.3
1	0	0	100	7.04	2	68	26.5	127	72	261	73	188	14.9	5.9	76.4	2	1	1	1	0	1	1	63.9	0.12	14.8
1	0	0	100	6.95	2	74	31.2	167	99	220	73	147	15.1	7.9	73.2	1	1	1	0	0	0	1	175.7	0.43	9.43
1	0	0	100	7	2	72	24.9	112	60	275	73	202	15.2	5.3	74.3	2	1	1	1	0	1	1	10.6	0.03	7.36
1	0	0	100	7.06	2	73	21.9	118.5	70	178	73	105	12.2	4.9	70.1	1	1	1	0	1	1	1	165	0.01	53.2
1	0	0	100	7.07	2	72	24.5	107	75	207	73	134	13.8	5.4	74.3	2	1	1	1	1	1	1	20.1	0.07	29.4
1	0	0	100	6.86	2	67	23.7	123	75	209	73	136	13.2	5.3	81.7	1	1	1	1	1	1	1	38.2	0.01	25.7
1	0	0	100	6.86	2	65	26.7	108	63	213	73	140	12.2	5	74.2	2	1	1	1	0	1	1	16	0.03	21.1
1	0	0	100	6.87	2	73	19.7	139	85.5	191	73	118	11.9	5.6	70.1	1	1	1	0	1	0	1	74.6	0.03	6.64
1	0	0	100	6.87	2	69	22.1	131	64.5	200	73	127	13.7	5.4	72.1	2	1	0	1	1	1	1	5.1	0.05	28
1	0	0	100	6.88	2	79	17.2	98	56	266	73	193	14.1	5.2	57.2	2	1	0	1	0	1	1	6.4	0.01	32.7
1	0	0	100	6.88	2	71	19.9	122	60	250	73	177	12.4	5.2	71.1	2	1	1	1	0	1	1	28.7	0.02	27.8
1	0	0	100	5.15	2	70	26.7	144.5	79	192	73	119	14	6.4	60.9	2	1	0	0	0	1	1	32.2	0.02	52.4
1	0	0	100	5.16	2	65	26	159.5	94.5	231	73	158	13.5	5.8	74.2	2	1	1	0	0	1	1	38.5	0.03	29.3
1	0	0	100	5.16	2	68	21.8	129	66	236	73	163	13.8	5.6	76.4	2	1	1	1	0	1	1	5.2	0.08	15.3
1	0	0	100	6.85	2	66	19.7	123	77.5	225	73	152	13.1	5.1	77.5	2	1	1	0	0	1	1	10.2	0.01	4.49
1	0	0	100	6.98	2	65	22	135.5	72	176	73	103	12.8	5.5	74.2	2	1	1	1	1	1	1	3.7	0.03	0.05
1	0	0	100	6.98	2	73	27.1	131	58.5	231	73	158	13.9	5.7	73.8	2	1	1	1	0	1	1	56.6	0.05	0.05
1	0	0	100	4.62	2	74	21.7	133	74.5	212	73	139	12.7	5.1	77.8	2	1	1	1	1	1	1	8.1	0.02	42.1
1	0	0	100	4.63	2	65	21.4	143.5	78.5	214	73	141	13.7	5.9	74.2	2	1	1	0	1	1	1	26.8	0.03	40.8
1	0	0	100	4.63	2	71	24	151	89.5	232	73	159	13.5	5.5	71.1	2	1	0	0	0	1	1	24.6	0.02	49.3
1	0	0	100	4.64	2	69	23	112.5	75.5	220	73	147	12.5	5.8	75.9	2	1	1	1	0	1	1	164.5	0.02	5.75
1	0	0	100	4.06	2	67	24.5	156.5	82.5	211	73	138	14.4	5.1	73.1	2	1	1	0	1	1	1	11.3	0.09	18.7
1	0	0	100	6.97	2	70	21.8	162.5	78.5	194	73	121	11.4	5.1	75.3	1	1	1	0	1	1	1	91.4	0.08	155
1	0	0	100	6.98	2	68	25.6	130	70	274	73	201	13.3	7.3	72.6	1	1	1	1	0	0	1	28.8	0.04	5.57
1	0	0	100	6.8	2	70	25.2	147	93	203	73	130	15.2	10.4	86.1	1	1	1	0	1	0	1	35.6	0.07	30.4
1	0	0	100	6.83	2	73	20.8	135.5	73	206	73	133	15.6	5.2	73.8	1	1	1	1	1	1	1	16.5	0.04	23.4
1	0	0	100	4.59	2	72	23.2	136.5	89	197	73	124	13.1	5.5	60.1	2	1	1	0	0	1	1	82.9	0.08	146
1	0	0	100	5.37	2	71	27	121.5	65.5	176	73	103	13.1	5.4	74.8	1	1	1	1	1	1	1	18.4	0.06	10.6
1	0	0	100	5.06	2	67	29.1	127.5	70	200	73	127	14.7	5.2	76.9	2	1	1	0	1	1	1	7	0.02	30.2
1	0	0	100	5.06	2	69	24.5	118	70	226	73	153	13.4	5.3	72.1	2	1	1	1	0	1	1	6.2	0.04	16.1
1	0	0	100	5.07	2	70	19.4	105.5	60	197	73	124	12.1	5.4	75.3	2	1	1	0	0	1	1	15.6	0.01	67.4
1	0	0	100	6.87	2	69	28.5	139	84.5	205	73	132	13.5	6.1	86.7	2	1	0	1	1	1	1	41.4	0.02	35.8
1	0	0	100	5.04	2	78	17.3	150	90	180	73	107	13.5	6	57.6	2	1	1	0	1	1	1	31.1	0.01	2.06

1	1	0	100	6.98	2	74	19.7	128.5	73	213	73	140	11.3	4.3	73.2	1	1	1	1	1	1	1	44.3	0.01	25.9
1	1	0	100	4.12	2	75	18.5	122.5	69.5	158	73	85	11.4	7	69.1	2	1	1	1	1	0	1	9.5	0.03	71.8
1	1	0	100	3.37	2	72	23.6	122	74.5	170	73	97	12.5	5.3	74.3	2	1	1	1	1	1	1	45.9	0.04	21.3
1	0	0	100	5.08	1	80	21.6	140.5	86	250	73	177	11.9	5.5	68.6	2	1	0	0	0	1	1	7.6	0.02	24.1
1	0	0	100	5.37	2	85	24.9	121.5	70.5	198	73	125	13	5.5	64.4	1	1	1	1	1	1	1	9.1	0.08	95.3
1	0	0	100	6.87	2	80	24.1	102.5	64.5	236	73	163	12.1	5.3	70.2	2	1	1	1	0	1	1	11	0.06	27.2
1	0	0	100	6.85	1	70	24.9	133.5	77	245	73	172	15.3	6.3	77.7	2	0	0	1	0	1	1	7.1	0.33	24.7
1	0	0	100	5.06	1	70	21.9	105.5	65.5	197	73	124	14.3	5.7	77.7	2	0	1	1	1	1	1	6.5	0.05	15.9
1	0	0	100	7	1	74	20.5	137.5	80.5	178	73	105	13.4	6	68.2	1	1	0	1	1	1	1	6.3	0.01	83.1
1	1	0	100	6.86	1	77	19.9	153.5	81.5	208	73	135	13.8	5.3	74.0	2	1	1	0	1	1	1	23.7	0.01	20.7
1	0	0	100	7.07	2	72	30.4	126	57.5	184	73	111	11.5	5.7	74.3	2	1	1	1	1	1	1	44.9	0.13	29.6
1	0	0	100	7.03	2	69	24.6	128	77	220	73	147	13.7	5.1	72.1	1	1	1	0	0	1	1	34.3	0.16	39.9
1	0	0	100	7	2	69	21.7	116.5	69.5	228	73	155	12.1	5.8	61.4	2	1	1	1	0	1	1	17.4	0.12	8.89
1	0	0	100	4.64	2	79	26.1	138.5	74.5	196	73	123	14.1	5.9	67.2	2	1	1	1	1	1	1	7.4	0.26	19.1
1	0	0	100	6.83	2	68	17	96.5	55.5	172	73	99	11.8	5.2	76.4	2	1	1	1	1	1	1	19	0.26	37.7
1	0	0	100	5.04	2	71	22.4	93	64.5	186	73	113	13	5.3	74.8	2	1	0	0	1	1	0	51.7	0.54	190
1	0	0	100	7.08	2	73	24.9	104.5	65	207	73	134	12.6	5	84.3	1	1	0	1	1	1	1	24.6	0.01	59.1
1	0	0	100	7.08	2	75	29.9	134	85.5	228	73	155	12.3	7.8	77.2	2	1	0	0	0	0	1	65.1	0.05	27.8
1	0	0	100	6.86	2	67	23.7	127	85.5	209	73	136	13.6	5.6	76.9	2	1	1	0	1	1	1	9.7	0.03	26.6
1	0	0	100	6.87	2	68	23.2	116	68.5	228	73	155	14.3	5.4	72.6	2	1	1	1	0	1	1	11.6	0.03	0.05
1	0	0	100	6.87	2	75	26.6	178.5	96	198	73	125	10.1	5.8	72.7	1	1	1	0	1	1	1	77.3	0.01	50.3
1	0	0	100	5.16	2	77	26.7	133.5	78	193	73	120	12.2	5.6	76.1	1	1	1	0	1	1	1	68.1	0.04	32
1	0	0	100	6.84	2	65	17.5	103	55.5	231	73	158	12	5.4	74.2	2	1	1	1	0	1	1	28.2	0.02	6.11
1	0	0	100	1.85	2	66	23.3	128	76	180	73	107	12.8	4.1	73.6	2	1	1	1	1	1	1	14.9	0.03	48
1	0	0	100	6.86	2	71	18	136.5	74	211	73	138	13.4	5.4	74.8	2	1	1	1	1	1	1	4.7	0.01	17.2
1	0	0	100	6.98	2	69	25.1	141.5	79.5	225	73	152	12.5	5.1	53.2	2	1	1	0	0	1	1	5.6	0.06	40
1	0	0	100	4.6	2	74	17.8	122	68	199	73	126	12	5.3	73.2	1	1	0	1	1	1	1	14.2	0.02	58.8
1	0	0	100	1.3	2	66	26.7	135.5	77.5	166	73	93	12.5	5.7	62.7	2	1	1	0	1	1	1	4.2	0.01	29.2
1	0	0	100	5.37	2	68	29.6	142	78.5	203	73	130	12.3	5.5	76.4	2	1	0	0	1	1	1	39.9	0.02	43.5
1	0	0	100	6.87	2	70	22.9	125.5	68.5	210	73	137	12.7	5.1	71.6	1	1	1	0	0	1	1	74.6	0.04	25
1	0	0	100	6.87	2	69	21.7	125	77	210	73	137	12.6	4.7	75.9	2	1	1	1	1	1	1	19.2	0.04	27.1
1	1	0	100	4.59	2	71	26.8	154.5	80	220	73	147	12.3	6.3	79.4	1	1	1	0	0	1	1	466.7	1.11	45.3
1	1	0	100	6.87	2	79	24.4	150.5	84.5	194	73	121	14.5	5.8	70.7	1	1	1	0	1	1	1	6.6	0.16	8.42
1	1	0	100	7	2	78	30.6	127.5	69.5	265	73	192	13.9	5.9	71.2	1	1	1	0	0	1	1	51.9	0.05	50.3
1	1	0	100	6.87	2	73	28.4	146	84	185	73	112	13.9	5	51.7	1	1	1	0	1	1	1	229	0.07	16.7
1	0	0	100	7	2	80	24	141.5	72.5	246	73	173	12.1	5.1	70.2	1	1	1	0	0	1	1	34.8	0.16	58.6
1	0	0	100	5.08	2	81	20.7	124.5	73	194	73	121	12.8	5.7	66.3	1	1	1	1	1	1	1	18	0.03	54.1
1	0	1	0.4	0.4	1	74	23.3	159.5	90.5	229	73	156	14.4	5.6	71.5	1	1	0	0	0	1	1	21.9	0.01	31.4
1	0	1	2.8	2.8	1	69	22.4	140	75	133	73	60	13.6	5.6	78.3	2	1	0	0	1	1	1	164.7	0.03	60.4
1	1	1	2.2	2.2	1	69	27.3	123.5	68	215	73	142	15.3	6.7	78.3	1	1	0	0	1	0	0	119.6	0.11	113
1	1	1	5.7	5.7	2	69	22.3	124.5	78	228	73	155	14.6	5.3	75.9	2	1	1	1	0	1	1	13.3	0.02	9.33
1	0	1	1.7	1.7	2	75	22.8	126.5	61.5	159	73	86	7.1	4.3	72.7	1	1	1	0	1	1	1	16	0.01	26.2
1	0	0	100	7.07	1	70	20.2	131	69.5	146	74	72	12.6	5.1	61.7	2	0	1	1	1	1	1	12.4	0.11	40
1	0	0	100	4.64	1	66	23.3	134.5	85	192	74	118	13.8	5.6	79.9	2	1	0	1	1	1	1	7.1	0.11	6.6
1	0	0	100	5.01	1	73	24.9	95.5	58.5	230	74	156	14.5	4.7	76.1	2	1	1	1	0	1	1	7.3	0.49	16.7
1	0	0	100	6.96	1	65	21.9	124	75.5	187	74	113	12.9	5	72.6	2	1	1	1	1	1	1	3	0.21	4.15
1	0	0	100	6.96	1	69	18.5	123.5	67	162	74	88	12.7	5.7	74.1	2	0	0	1	1	1	1	10.7	0.8	0.05
1	0	0	100	5.36	1	68	23.7	148	82	189	74	115	14.6	5.5	71.1	2	1	0	0	1	1	1	7.8	0.12	103
1	0	0	100	6.87	1	68	23.8	125	77.5	150	74	76	16.2	7	74.6	2	1	0	1	1	0	1	11.6	0.04	34.3
1	0	0	100	7.04	1	71	21.3	166	97	218	74	144	14.9	5.4	73.1	2	1	1	0	1	1	1	6.2	0.02	0.05
1	0	0	100	1.82	1	73	29.6	136.5	86	209	74	135	16.2	6	72.0	1	1	0	1	1	1	0	7.3	0.06	75.4

1	0	0	100	4.64	1	68	19.8	122.5	74.5	175	74	101	13.5	5.5	84.0	2	1	0	1	1	1	1	32.6	0.03	64.7
1	0	0	100	6.96	1	74	28.5	169.5	88	195	74	121	14.6	5.3	68.2	2	1	0	0	1	1	1	22.2	0.02	56.5
1	0	0	100	6.8	1	71	20.3	117.5	79	224	74	150	15	5.2	73.1	2	0	0	1	0	1	1	6.5	0.05	12.9
1	0	0	100	6.84	1	69	21.1	111.5	69.5	152	74	78	13.5	5.7	70.6	2	1	0	1	1	1	1	13.5	0.04	37.3
1	0	0	100	4.6	1	69	23.1	151	79	170	74	96	13.1	5.2	74.1	2	1	0	0	1	1	1	9	0.01	28.2
1	1	0	100	6.86	1	73	21	162.5	84	147	74	73	12.7	4.9	76.1	2	1	0	0	1	1	1	12.3	0.12	66.8
1	0	0	100	5.91	2	71	26.3	188.5	104	236	74	162	13.6	5.4	74.8	1	1	0	0	0	1	1	118.5	0.61	75.4
1	0	0	100	6.86	2	65	29.8	100	68.5	187	74	113	13.2	5.4	74.2	2	1	1	1	1	1	1	24.6	0.11	6.26
1	0	0	100	6.84	2	67	23.9	106.5	52	235	74	161	14.7	5.6	81.7	2	1	1	1	0	1	1	18.1	0.15	17.5
1	0	0	100	5.08	2	76	23.2	112.5	63.5	185	74	111	12.8	6.4	68.6	1	1	1	0	1	1	1	18	1.26	10.4
1	0	0	100	6.82	2	65	22.9	114.5	65.5	245	74	171	12.5	5.9	78.0	2	1	1	1	0	1	1	11.9	0.11	143
1	0	0	100	6.79	2	78	26.8	155.5	89.5	240	74	166	13.6	6.1	67.7	2	1	1	0	0	1	1	23.6	0.42	24.3
1	0	0	100	6.44	2	67	25.7	123	84	231	74	157	12.6	5.8	76.9	2	1	1	1	0	1	1	31.3	0.37	42.4
1	0	0	100	7	2	69	24.8	137.5	73	213	74	139	12.1	5.5	75.9	2	1	1	1	1	1	1	16.5	0.01	3.52
1	0	0	100	7.06	2	74	25.4	139	83	209	74	135	13.1	4.9	69.6	1	1	1	0	1	1	1	8	0.02	42
1	0	0	100	7.07	2	68	22.5	185	99.5	224	74	150	14.6	5.6	76.4	1	1	1	0	0	1	1	51.7	0.02	50.6
1	0	0	100	5.16	2	69	20.5	101.5	64	177	74	103	11.5	5.5	72.1	2	1	1	1	1	1	1	16.3	0.02	42.2
1	0	0	100	7.2	2	71	23.4	126.5	73	243	74	169	10.9	5.5	60.5	2	1	1	1	0	1	1	11.8	0.01	40.4
1	0	0	100	7.02	2	70	23	120.5	71.5	211	74	137	12.7	5.2	60.9	1	1	0	1	1	1	1	19.8	0.06	17.6
1	0	0	100	6.98	2	73	18	108.5	65.5	203	74	129	12.8	5.4	78.3	2	1	1	1	1	1	1	6.3	0.01	10.4
1	0	0	100	7	2	66	28.9	145.5	86	255	74	181	13.4	5	82.3	2	1	1	0	0	1	1	4.9	0.04	4.66
1	0	0	100	4.63	2	67	19.3	95	61	187	74	113	12.1	5.8	81.7	2	1	1	1	1	1	1	24.8	0.03	21.5
1	0	0	100	4.64	2	66	27	135	92	245	74	171	13.8	5.7	73.6	2	1	1	0	0	1	1	24.4	0.02	24.6
1	0	0	100	4.64	2	68	23	129.5	74.5	248	74	174	13.4	5.9	81.1	2	1	1	1	0	1	1	24.2	0.01	29.8
1	0	0	100	6.87	2	68	26.7	113	67.5	225	74	151	13.8	7.8	72.6	2	1	1	0	0	0	1	27.7	0.02	10.4
1	0	0	100	6.95	2	70	25.5	143	76	202	74	128	11.2	5.2	80.0	2	1	1	0	1	1	1	16.9	0.06	16.7
1	0	0	100	6.97	2	66	27.6	138	83.5	216	74	142	12.6	5.5	62.7	2	1	1	0	1	1	1	12.9	0.03	23.3
1	0	0	100	6.98	2	74	24.4	112.5	61.5	218	74	144	13.2	4.3	77.8	2	1	1	1	1	1	1	18.3	0.08	50.7
1	0	0	100	6.79	2	74	19.7	141	82	186	74	112	12.2	6	77.8	2	1	1	0	1	1	1	27.3	0.02	14.6
1	0	0	100	6.8	2	71	23	131.5	80	198	74	124	14.1	5.3	71.1	2	1	1	0	0	1	1	36.5	0.02	23.9
1	0	0	100	6.8	2	73	21.6	95.5	66	195	74	121	12.1	5.5	78.3	1	1	1	1	1	1	1	12.6	0.03	52.5
1	0	0	100	4.59	2	65	19.2	133	85.5	208	74	134	12	5.4	78.0	2	1	1	1	1	1	1	14.5	0.02	40.8
1	0	0	100	4.59	2	70	21.1	110	64.5	206	74	132	12	5.5	75.3	2	1	1	0	1	1	1	28.2	0.04	31.7
1	0	0	100	4.59	2	73	20.2	134	77.5	236	74	162	13.9	5.2	73.8	2	1	1	1	0	1	1	28.4	0.03	50.9
1	0	0	100	4.62	2	76	22.2	139	77.5	242	74	168	15.1	5.5	58.4	2	1	1	1	0	1	1	19.5	0.07	17.2
1	0	0	100	6.97	2	65	25.4	142	84	209	74	135	13.7	5.7	78.0	2	1	0	0	1	1	1	8	0.03	6.99
1	0	0	100	6.87	2	66	25.7	109	72	233	74	159	14.2	5.7	77.5	2	1	1	1	0	1	1	10.4	0.02	8.11
1	0	0	100	5.04	2	68	22.9	117.5	65.5	201	74	127	14.4	6	72.6	2	1	1	1	1	1	1	6	0.03	15.4
1	1	0	100	4.89	2	79	16.4	126.5	69.5	155	74	81	10.8	5.5	67.2	2	1	1	1	1	1	1	12.7	0.01	103
1	1	0	100	6.55	2	74	25.2	141.5	73	207	74	133	12.4	5.4	73.2	1	1	1	0	1	1	1	17.6	0.05	14.7
1	1	0	100	6.97	2	67	22.1	112	71	227	74	153	8.6	5.3	81.7	2	1	1	1	0	1	1	19.1	0.01	8.25
1	0	0	100	5.47	1	85	24.2	151	89.5	184	74	110	11.8	5.3	70.0	2	1	1	0	1	1	1	18.2	0.12	28.3
1	0	0	100	7.01	1	81	18.7	97	48	194	74	120	10.2	5.6	50.9	1	1	0	1	1	1	1	8.1	0.05	219
1	1	0	100	5.08	1	80	17.8	105.5	63	219	74	145	12.8	5.9	57.5	2	1	1	1	1	1	1	9.3	0.03	65.8
1	0	0	100	7.08	2	81	23.5	114	56.5	221	74	147	11.7	5.5	69.7	1	1	1	1	0	1	1	18.4	0.03	0.05
1	0	0	100	6.87	1	67	24.1	119.5	77.5	218	74	144	15.6	5.4	75.2	2	0	0	0	1	1	1	12.5	0.04	30.4
1	0	0	100	4.7	1	68	23	145	94.5	199	74	125	11	5.9	78.8	2	1	0	0	1	1	1	17.1	0.01	70.5
1	1	0	100	6.87	1	71	19.9	146.5	81.5	175	74	101	12.2	5.4	73.1	2	0	0	0	1	1	1	334.8	0.27	51.6
1	0	0	100	6.84	2	71	30	131.5	76.5	250	74	176	14	5.8	60.5	2	1	1	1	0	1	1	12.1	0.1	27.1
1	0	0	100	4.64	2	70	29.7	125.5	70.5	184	74	110	13.1	6	71.6	2	1	1	0	1	1	1	12	0.11	9.33
1	0	0	100	6.44	2	67	26.5	147.5	87	243	74	169	14.8	8.6	62.2	2	1	1	0	0	0	1	695.2	0.16	53

1	0	0	100	7	2	75	28.9	178.5	84	218	74	144	13	5.7	77.2	1	1	1	0	0	1	1	348.8	0.05	21.3
1	0	0	100	6.86	2	74	21.9	149	74.5	186	74	112	12.1	5.2	73.2	1	1	1	0	1	1	1	33.2	0.04	54.6
1	0	0	100	5.08	2	73	18.6	130.5	55	216	74	142	12.5	5.4	78.3	2	1	1	0	1	1	1	6.1	0.09	127
1	0	0	100	4.64	2	71	24.8	125.5	79	188	74	114	12.2	4.9	71.1	1	1	0	1	1	1	1	19.1	0.03	84.2
1	0	0	100	6.97	2	69	27.2	137	81.5	241	74	167	15.1	4.8	80.5	1	1	1	1	0	1	1	285.7	0.02	12.9
1	0	0	100	6.98	2	67	28.3	157	91.5	228	74	154	13.5	5.7	76.9	1	1	1	0	0	1	1	177.3	0.03	72.5
1	0	0	100	6.98	2	66	21	109.5	70.5	224	74	150	15.2	5.2	82.3	2	1	0	1	0	1	1	96.1	0.04	0.81
1	0	0	100	6.98	2	69	26.7	131	80	233	74	159	13.5	5.5	75.9	1	1	1	0	0	0	1	29.8	0.04	52.1
1	0	0	100	6.96	2	66	23.1	99.5	67	170	74	96	11.1	4.9	77.5	2	1	1	1	1	1	1	12.4	0.04	0.05
1	0	0	100	6.97	2	69	24.5	118	69	204	74	130	12.6	5.9	75.9	2	1	1	1	0	1	1	26.3	0.07	19
1	0	0	100	5.37	2	70	21	104.5	61.5	223	74	149	13.7	5.5	71.6	1	1	1	1	0	1	1	9.4	0.01	23.7
1	1	0	100	7.08	2	75	27.2	104.5	67	213	74	139	10.7	5	69.1	1	1	1	0	0	1	1	19.1	0.05	18.3
1	1	0	100	7.23	2	72	25	130.5	71.5	196	74	122	13.9	5.4	74.3	2	1	1	1	1	1	1	19.8	0.09	71.3
1	1	0	100	6.87	2	75	23.8	159.5	88	234	74	160	15	6.3	58.8	1	1	1	0	0	1	1	7.4	0.05	9.71
1	1	0	100	5.04	2	76	25.5	113.5	60	211	74	137	11.8	5.5	72.2	2	1	1	0	1	1	1	140.9	0.05	101
1	1	0	100	6.8	2	83	22.6	121.5	63.5	205	74	131	12.3	6.8	42.5	2	1	1	0	1	0	1	55.1	0.06	152
1	0	1	3.1	3.1	2	70	27.2	126.5	78.5	182	74	108	12.8	5.8	80.0	1	1	1	1	1	1	1	76.9	0.02	76.6
1	0	0	100	5.37	1	70	20.4	153	82	151	75	76	13.3	6.5	82.8	2	1	0	0	1	0	1	20.4	0.34	42.6
1	0	0	100	7.06	1	76	22.4	124.5	75.5	243	75	168	14.1	5.4	67.2	2	0	1	1	0	1	1	7.4	0.06	33.3
1	0	0	100	7.08	1	67	20.7	110	68.5	168	75	93	12.6	4.7	79.4	2	1	0	1	1	1	1	7.4	0.02	40
1	0	0	100	4.58	1	74	19.8	130.5	73.5	242	75	167	12.7	6	75.6	2	1	0	0	0	1	1	60	0.03	6.57
1	0	0	100	4.59	1	69	21.9	125	75	165	75	90	14.2	5.2	74.1	2	1	0	0	1	1	1	7.2	0.01	4.44
1	0	0	100	5.16	1	67	27.6	110.5	75	203	75	128	15	5.5	79.4	2	1	1	0	0	1	1	13.9	0.02	16.9
1	0	0	100	6.85	1	71	20.3	113	57.5	197	75	122	12.8	6.8	69.6	2	0	0	0	1	0	1	24.4	0.03	19.9
1	0	0	100	5.05	1	69	23	142.5	77	184	75	109	14.8	5.3	78.3	2	1	0	0	1	1	1	17.8	0.03	42.6
1	0	0	100	5.08	1	71	26.5	137	65.5	180	75	105	16.1	5.2	49.2	2	1	0	1	1	1	1	14.6	0.04	0.1
1	0	0	100	6.98	1	67	22.2	126.5	82.5	161	75	86	12.5	5	84.6	2	1	0	0	1	1	1	26.9	0.03	12.1
1	0	0	100	4.63	1	70	20	151.5	90	177	75	102	14.3	4.7	77.7	2	1	0	0	1	1	1	10.7	0.04	12.6
1	0	0	100	6.87	1	77	22.8	137.5	80	187	75	112	14.5	4.8	70.1	1	1	0	1	1	1	1	15.4	0.05	14.9
1	0	0	100	6.84	1	71	22.4	135	72	134	75	59	16	4.5	77.2	2	1	0	0	1	1	1	5	0.04	41.7
1	0	0	100	4.62	1	65	16.6	93	61.5	212	75	137	13.7	6.1	76.2	2	1	1	1	1	1	1	25.8	0.08	26.9
1	0	0	100	6.44	1	67	22.1	152.5	82.5	182	75	107	15.2	6.1	84.6	2	1	0	0	1	0	1	12.2	0.06	3.1
1	0	0	100	5.04	1	66	28.3	151	82	183	75	108	14.1	5.3	79.9	2	1	0	0	1	1	1	112.7	0.07	32
1	0	0	100	7	2	65	25.6	149	64	229	75	154	13.9	5.1	63.1	2	1	1	0	0	1	1	12.8	0.13	77.2
1	0	0	100	4.63	2	73	25.9	146.5	87	230	75	155	13	5.7	73.8	1	1	0	0	0	1	1	29.4	0.18	53.7
1	0	0	100	4.59	2	67	23.2	212	101.5	232	75	157	14.9	5.7	73.1	2	1	1	0	0	1	1	29.1	0.28	189
1	0	0	100	4.7	2	74	27.1	135.5	73.5	231	75	156	14.2	5.1	69.6	1	1	1	1	0	1	1	23.3	0.12	11
1	0	0	100	5.37	2	67	20.2	103.5	58	213	75	138	11.8	5.8	73.1	2	1	0	1	1	1	1	13.1	2.81	55.8
1	0	0	100	7.07	2	72	22.9	129.5	74	213	75	138	12.1	5.1	74.3	1	1	1	1	1	1	1	19.5	0.02	6.84
1	0	0	100	7.07	2	74	27.5	153.5	84.5	239	75	164	13.4	5.3	77.8	2	1	1	0	0	1	1	19.4	0.08	29.3
1	0	0	100	4.73	2	69	22.4	133	85	291	75	216	13	5.1	61.4	2	1	1	1	0	1	1	36.3	0.02	3.12
1	0	0	100	4.58	2	65	25.6	133.5	76.5	232	75	157	13.4	5.7	78.0	2	1	1	1	0	1	1	27.3	0.08	16.8
1	0	0	100	5.16	2	77	18.7	124.5	76.5	181	75	106	12.7	5.3	68.2	1	1	1	1	1	1	1	6.1	0.01	19.2
1	0	0	100	6.86	2	74	20.5	124	72.5	235	75	160	10.5	5.4	69.6	2	1	1	1	0	1	1	30.5	0.01	36.1
1	0	0	100	5.05	2	73	25.8	115.5	62.5	205	75	130	12.2	6.1	78.3	2	1	1	1	1	1	1	13.3	0.05	11.3
1	0	0	100	5.05	2	66	20.8	130	78.5	214	75	139	13.7	5.2	77.5	2	1	1	0	1	1	1	57	0.01	29.7
1	0	0	100	7	2	67	18	94	56.5	223	75	148	12	5.3	76.9	2	1	1	1	0	1	1	10.5	0.01	14.5
1	0	0	100	4.62	2	66	20.9	119	75	247	75	172	14.2	5	77.5	2	1	1	0	0	1	1	7.6	0.05	38.5
1	0	0	100	4.63	2	71	21.7	147	78	239	75	164	13	5.6	74.8	2	1	1	0	0	1	1	141.5	0.06	36
1	0	0	100	4.63	2	65	22.6	92.5	66	207	75	132	12.9	5.2	78.0	2	1	1	1	1	1	1	18.7	0.01	7.28
1	0	0	100	6.87	2	74	25.7	151.5	82.5	251	75	176	13.9	5.1	73.2	2	1	1	0	0	1	1	25.3	0.08	13.3

1	0	0	100	4.6	2	78	21.3	125	74.5	217	75	142	12.9	5.4	57.6	2	1	1	1	1	1	1	15.2	0.02	21.5
1	0	0	100	4.67	2	65	22.7	122.5	76	240	75	165	13.1	5.2	78.0	2	1	1	1	0	1	1	33	0.07	6.29
1	0	0	100	4.69	2	68	19.5	129	76.5	222	75	147	13.3	5.2	76.4	2	1	1	1	0	1	1	18.1	0.09	11.7
1	0	0	100	4.69	2	69	20.8	132	70.5	197	75	122	12.2	5.2	80.5	2	1	0	1	1	1	1	21.9	0.03	29.7
1	0	0	100	6.96	2	75	24.7	133.5	81	240	75	165	13.9	5.3	51.0	1	1	0	0	0	1	1	6.2	0.07	0.05
1	0	0	100	5.36	2	70	21.3	147	80.5	186	75	111	12.9	5.4	60.9	2	1	1	0	1	1	1	20.2	0.02	40.4
1	0	0	100	5.06	2	71	20.8	119	65	242	75	167	11.5	5.6	71.1	2	1	1	1	0	1	1	4.8	0.01	45.7
1	0	0	100	6.85	2	68	22.4	141	71.5	203	75	128	12.8	4.8	76.4	2	1	1	0	1	1	1	30.7	0.03	43
1	0	0	100	6.86	2	76	23.3	160.5	89	271	75	196	13	5.5	68.6	1	1	1	0	0	1	1	15.3	0.04	56.9
1	0	0	100	1.23	1	81	20.8	104.5	65	191	75	116	12.8	5.2	30.1	1	1	1	1	0	1	1	48.6	0.13	39.2
1	0	0	100	6.87	1	81	22.1	140	70	186	75	111	12.5	5.1	68.1	2	1	0	0	1	1	1	68.7	0.03	61.2
1	1	0	100	7.07	1	83	23.6	130.5	61.5	221	75	146	14.9	5.7	64.0	1	1	1	0	0	1	1	27.2	0.09	10
1	1	0	100	6.3	1	80	21.3	164	84.5	166	75	91	13.9	4.9	68.6	1	1	1	0	1	1	1	27.9	0.05	40
1	0	0	100	6.8	2	80	24.8	111.5	59	211	75	136	13.5	5.5	43.4	2	1	0	1	1	1	1	8.6	0.14	61.9
1	0	0	100	3.06	2	87	23.7	115.5	68	206	75	131	9	6.6	66.8	2	1	1	1	1	0	1	42.4	0.04	8.31
1	0	0	100	4.7	1	71	19.2	126	84	213	75	138	14.9	5.3	82.2	1	0	0	1	1	1	1	12.7	0.13	1.46
1	0	0	100	6.87	1	72	22.3	156.5	98.5	253	75	178	16.3	5.7	60.9	2	1	1	0	0	1	1	12	0.08	0.05
1	0	0	100	6.85	1	66	20.8	122.5	81	207	75	132	13.9	5.5	79.9	2	1	0	1	1	1	1	8.7	0.01	15.9
1	0	0	100	5.06	1	67	23.1	123.5	75.5	173	75	98	15.4	5.3	84.6	2	1	1	1	1	1	1	105.5	0.02	39.3
1	1	0	100	7	1	73	19.7	142.5	86.5	141	75	66	12.5	5.9	48.5	2	0	0	0	1	1	1	14.4	0.01	138
1	0	0	100	6.86	2	67	31.4	143	82.5	287	75	212	14	7.8	76.9	2	1	1	0	0	0	1	10.5	0.18	0.05
1	0	0	100	7.08	2	66	27.2	162.5	93.5	201	75	126	14.7	7.4	82.3	2	1	1	0	0	0	1	97.2	0.05	5.74
1	0	0	100	7.04	2	73	21.2	120	55	194	75	119	12.2	5	78.3	2	1	1	0	0	1	1	90.1	0.01	0.05
1	0	0	100	5.06	2	72	20.9	149.5	82.5	172	75	97	13.7	5.2	60.1	1	1	1	0	1	1	1	7	0.01	48.2
1	0	0	100	6.97	2	72	27.7	133.5	71.5	202	75	127	12	5.1	74.3	1	1	1	0	0	1	1	12.2	0.01	0.05
1	0	0	100	4.63	2	72	27.7	127.5	85	201	75	126	13.3	5.3	78.9	2	1	1	1	1	1	1	12.6	0.04	11.9
1	0	0	100	6.98	2	71	22	123	75.5	182	75	107	12.2	6.7	74.8	2	1	1	0	1	0	1	14.4	0.01	58.7
1	0	0	100	6.8	2	65	22.9	107	71	197	75	122	12.2	5.3	82.8	2	1	1	1	1	1	1	6.4	0.03	21
1	0	0	100	6.83	2	65	29.8	105.5	67	192	75	117	13.8	6.1	78.0	2	1	1	1	1	1	1	31.8	0.05	12.7
1	0	0	100	5.04	2	66	27.2	127.5	86	209	75	134	12.7	6.2	77.5	2	1	1	1	1	1	1	15.9	0.09	4.78
1	0	1	5.6	5.6	1	73	21	101.5	58.5	164	75	89	13.2	4.9	76.1	2	0	0	1	1	1	1	27.2	1.78	215
1	0	0	100	4.58	1	77	18.4	115	69	160	76	84	11.1	6	70.1	2	1	0	1	1	1	1	48.1	0.26	14.8
1	0	0	100	4.64	1	76	21	169.5	79	207	76	131	14.9	6.6	59.2	2	1	1	0	0	0	1	9.8	0.45	20.4
1	0	0	100	3.35	1	75	23	165	89	186	76	110	15.8	5.1	80.0	2	1	0	0	1	1	1	27.2	0.05	20.1
1	0	0	100	6.86	1	72	21	117.5	64.5	217	76	141	14.2	4.3	76.7	2	1	0	1	1	1	1	73.9	0.04	71.5
1	0	0	100	6.87	1	75	24	125.5	85	272	76	196	15	5.7	67.7	2	1	1	1	0	1	1	24	0.05	17.8
1	0	0	100	5.16	1	66	19.5	118.5	80	147	76	71	13.7	5.4	75.7	1	1	0	1	1	1	1	21.9	0.01	50.5
1	0	0	100	6.84	1	67	21.6	128	79	227	76	151	14.6	5.2	75.2	2	0	1	1	0	1	1	12	0.01	15
1	0	0	100	6.84	1	73	20.3	158	86	203	76	127	14.4	5.4	72.0	2	1	0	0	1	1	1	22.5	0.02	11.7
1	0	0	100	7.03	1	65	22.3	132	83.5	188	76	112	12.9	5.4	80.5	2	1	1	1	1	1	1	8.5	0.03	13.1
1	0	0	100	6.97	1	72	18.4	155	76	189	76	113	13.8	5.1	76.7	1	1	1	0	1	1	1	29.5	0.04	2.51
1	0	0	100	7	1	79	18.4	107	75.5	167	76	91	12.9	5.3	77.7	1	1	0	0	1	1	1	36	0.04	25.5
1	0	0	100	4.64	1	71	26.3	132.5	74	184	76	108	15.3	5.6	77.2	2	1	0	0	1	1	1	12.6	0.06	25.9
1	0	0	100	6.8	1	66	21.9	109.5	70.5	156	76	80	15.6	5.2	63.5	2	0	0	0	1	1	1	21	0.02	61.4
1	0	0	100	6.83	1	71	23.4	146	80.5	184	76	108	13.9	5.4	73.1	2	1	1	0	1	1	1	13	0.05	19.5
1	0	0	100	4.7	1	69	22.4	153	73.5	213	76	137	14.6	5.6	74.1	2	1	1	0	1	1	1	132.4	0.05	22
1	0	0	100	5.06	1	67	22.1	139	84	173	76	97	13.7	4.7	79.4	2	1	0	0	1	1	1	7.7	0.01	32.8
1	0	0	100	6.87	1	66	19.3	144.5	89.5	240	76	164	14.5	5.3	75.7	2	1	0	0	0	1	1	41	0.01	19.3
1	1	0	100	6.87	1	67	23.7	125	71.5	177	76	101	14.2	5.3	79.4	2	1	0	0	0	1	1	10.5	0.03	25.5
1	1	0	100	5.83	1	75	20.6	147.5	78	214	76	138	13.1	5.5	71.0	2	1	1	0	1	1	1	11.5	0.05	36.8
1	0	0	100	6.86	2	75	22.1	99.5	61.5	258	76	182	12.3	5.2	72.7	1	1	1	1	0	1	1	5.5	0.1	55.3

1	0	0	100	5.05	2	69	23.9	152	85	194	76	118	11	5.7	72.1	2	1	1	0	1	1	1	266.8	0.18	29
1	0	0	100	4.59	2	66	22	102.5	65	178	76	102	11.7	5	77.5	2	1	0	1	1	1	1	14.5	5.15	15.3
1	0	0	100	5.37	2	66	23	93	60	201	76	125	13.9	5.6	62.7	2	1	1	1	1	1	1	5.8	0.81	11.8
1	0	0	100	6.87	2	72	26.1	121	68.5	201	76	125	12.3	5.2	70.6	2	1	1	1	1	1	1	9.6	0.11	32.9
1	0	0	100	5.04	2	66	24.4	120	65	176	76	100	10.8	5.4	77.5	2	1	1	1	1	1	1	22.1	0.1	28.4
1	0	0	100	7.07	2	66	22.6	113.5	68.5	216	76	140	13.7	5.6	62.7	2	1	0	0	0	1	1	5.5	0.02	29.9
1	0	0	100	4.58	2	70	25.5	122	69	234	76	158	13.1	5.3	75.3	2	1	1	0	0	1	1	38.7	0.03	15.2
1	0	0	100	4.59	2	72	19.9	100	56.5	206	76	130	12.3	5.6	70.6	2	1	0	1	1	1	1	7.8	0.01	31.6
1	0	0	100	4.39	2	71	24.3	106.5	66	268	76	192	14.2	6	74.8	2	1	1	1	0	1	1	9.5	0.05	6.82
1	0	0	100	5.16	2	65	21	147.5	83.5	249	76	173	13.9	5.3	78.0	2	1	0	0	0	1	1	10.7	0.02	17.1
1	0	0	100	6.86	2	78	24.5	128	73.5	258	76	182	12.7	5.3	57.6	1	1	1	0	0	1	1	6.8	0.02	11.3
1	0	0	100	6.86	2	66	22.1	102	58	214	76	138	11.1	5.3	73.6	2	1	1	1	1	1	1	7.9	0.02	14.1
1	0	0	100	7.01	2	65	24.6	117	61.5	214	76	138	13.9	9.3	82.8	2	1	0	1	1	0	1	126.3	0.08	6.05
1	0	0	100	6.98	2	73	28.7	154.5	83.5	210	76	134	12.6	6	73.8	1	1	1	0	1	1	1	14.9	0.08	0.05
1	0	0	100	4.63	2	70	24.2	113.5	70.5	189	76	113	12.7	7.5	75.3	2	1	0	1	1	0	1	29.4	0.07	34.1
1	0	0	100	4.64	2	68	23.1	130	80	194	76	118	13.7	5.1	76.4	2	1	1	1	1	1	1	20.1	0.03	7.19
1	0	0	100	4.64	2	67	24.5	100	63	232	76	156	13.9	5.4	76.9	2	1	1	1	0	1	1	9.4	0.02	28.4
1	0	0	100	6.95	2	78	27.1	119	70	261	76	185	15	5.2	71.2	2	1	0	0	0	1	1	15.1	0.06	26.5
1	0	0	100	6.95	2	65	26.4	121	71.5	242	76	166	12.5	5.4	74.2	2	1	1	1	0	1	1	5.2	0.01	111
1	0	0	100	6.98	2	67	27.2	189	95	211	76	135	13.5	5.6	81.7	2	1	0	0	0	1	1	264	0.03	105
1	0	0	100	4.59	2	72	25.9	141	83.5	227	76	151	13.5	4.9	74.3	2	1	1	0	0	1	1	20.9	0.08	34.8
1	0	0	100	4.62	2	67	25.6	133	76	201	76	125	14.2	5.3	76.9	1	1	1	1	1	1	1	9.5	0.02	38.4
1	1	0	100	7.01	1	83	21.3	125.5	55	165	76	89	12.2	5.5	71.0	2	1	0	1	1	1	1	22.8	0.01	108
1	0	0	100	5.06	1	76	24	153	68	205	76	129	12.5	5.7	74.5	2	1	0	0	0	1	1	88.1	0.03	61
1	0	0	100	6.8	1	65	21.4	164	100	179	76	103	14.4	5.9	80.5	2	0	0	0	1	1	1	9.6	0.04	19.9
1	0	0	100	7.07	2	71	25.4	141.5	80.5	282	76	206	14.6	5.3	60.5	2	1	1	0	0	1	1	137.1	0.13	14.2
1	0	0	100	6.86	2	74	26.7	133	69.5	200	76	124	13.1	6.1	69.6	1	1	1	0	1	1	1	10.8	1.82	48.4
1	0	0	100	5.07	2	72	21.5	96.5	56.5	202	76	126	12.8	5.6	70.6	1	1	1	1	1	1	1	13.1	0.22	25.7
1	0	0	100	6.87	2	75	30.4	128.5	71.5	189	76	113	14.3	5.1	69.1	2	1	1	1	1	1	1	357.2	0.12	63
1	0	0	100	7.08	2	68	29.7	148.5	93	214	76	138	13.4	5.5	81.1	1	1	1	0	1	1	1	22.5	0.03	16.3
1	0	0	100	6.88	2	67	31.6	125	70.5	231	76	155	12.1	5.1	62.2	2	1	1	1	0	1	1	103.5	0.05	83.9
1	0	0	100	6.84	2	70	19.4	141	85	283	76	207	13.5	5.5	75.3	2	1	1	0	0	1	1	12	0.01	44.3
1	0	0	100	2.19	2	79	22	105	50.5	216	76	140	14.4	5.6	70.7	1	1	1	1	1	1	0	10.7	0.06	152
1	0	0	100	7.02	2	75	27	97.5	64.5	275	76	199	12.8	5.7	69.1	1	1	0	1	0	1	1	10.7	0.04	8.96
1	0	0	100	6.87	2	75	22.8	143.5	76	162	76	86	13.9	5.1	72.7	1	1	1	0	1	1	1	23.1	0.04	60.6
1	0	0	100	6.96	2	67	24	165.5	105.5	192	76	116	13.6	5.9	81.7	2	1	1	0	1	1	1	87.9	0.04	0.05
1	0	0	100	6.96	2	66	22.4	122.5	67.5	233	76	157	11.4	5.1	88.5	2	1	1	1	0	1	1	34.9	0.01	0.05
1	0	0	100	5.04	2	71	22.9	139	79	181	76	105	11.4	5.4	79.4	1	1	1	1	1	1	1	14.2	0.04	53.5
1	1	0	100	7.08	2	74	26.6	130	80.5	218	76	142	12.2	5.5	69.6	1	1	1	1	1	1	1	44.1	0.06	21.1
1	1	0	100	4.62	2	76	25.8	126.5	60	240	76	164	12.4	5.7	72.2	2	1	1	1	0	1	1	24.2	0.07	41.4
1	0	1	2.7	2.7	1	69	21.1	101.5	63.5	181	76	105	13.7	5.5	74.1	2	1	0	1	1	1	1	34.2	0.02	72
1	0	1	1.7	1.7	1	72	22.8	131	83.5	160	76	84	14.2	5.5	72.6	1	1	0	1	1	1	0	22	0.09	129
1	1	1	2.6	2.6	1	68	22.6	161	93	195	76	119	13.1	4.9	74.6	1	1	0	0	1	1	1	60.2	0.02	132
1	1	1	1.5	1.5	2	76	29.5	134.5	74.5	181	76	105	14.8	7.5	58.4	1	1	1	0	1	0	1	176.6	0.05	51.7
1	0	0	100	5.08	1	70	21.5	107.5	55	176	77	99	13	6.3	77.7	1	1	1	0	1	1	1	17.8	5.8	8.95
1	0	0	100	6.98	1	66	20.8	133.5	76	241	77	164	15.4	5.6	79.9	2	1	0	0	0	1	1	18.5	0.15	1.56
1	0	0	100	4.69	1	76	23.7	109	68.5	175	77	98	14.3	5.5	74.5	2	0	0	1	1	1	1	11.3	0.4	24.6
1	0	0	100	4.81	1	78	23	102.5	60.5	175	77	98	13.3	5	78.3	2	1	0	1	1	1	1	16.5	0.01	17
1	0	0	100	7.03	1	65	25.4	133	86.5	212	77	135	15.8	5.3	80.5	2	1	0	1	1	1	1	18.9	0.02	22.7
1	0	0	100	5.05	1	66	19.9	132	81	152	77	75	14.2	5	72.1	2	1	0	1	1	1	1	3.7	0.03	87.5
1	0	0	100	5.36	1	65	22	157	90	186	77	109	16	5.3	80.5	2	1	0	0	1	1	1	24.2	0.03	14.5

1	0	0	100	6.85	1	65	20.8	108	58	204	77	127	16.2	4.9	76.2	2	0	1	1	1	1	1	21.3	0.02	20
1	0	0	100	6.87	1	72	23	145.5	84.5	191	77	114	15.4	6	72.6	2	1	0	0	1	1	1	6.3	0.02	17.2
1	1	0	100	6.97	1	76	21.8	165.5	90.5	155	77	78	12.5	5.5	79.4	2	1	0	0	1	1	1	55.8	0.19	57.9
1	1	0	100	6.29	1	75	21.1	145	84.5	174	77	97	15.1	4.9	59.6	1	1	0	0	1	1	1	7.5	0.29	27.5
1	1	0	100	7.02	1	74	23.1	142.5	84	199	77	122	14.7	5.1	71.5	2	0	0	0	1	1	1	10.9	0.04	111
1	1	0	100	2.81	1	76	17.7	123	66	186	77	109	12.3	6.4	70.5	2	1	1	1	1	1	1	9.1	0.06	27.4
1	0	0	100	6.87	2	78	17.3	120	64.5	198	77	121	12.4	5.7	75.6	2	1	1	1	1	1	1	31.4	0.41	30.8
1	0	0	100	4.68	2	68	22.9	103	67	216	77	139	13.8	4.8	72.6	2	1	1	1	1	1	1	9.7	0.52	56.7
1	0	0	100	3.46	2	65	21.5	171	104.5	177	77	100	14.9	5	78.0	2	1	1	0	1	1	1	17.6	1.09	45.7
1	0	0	100	7	2	65	25.1	162	84.5	212	77	135	13.4	5.5	74.2	2	1	1	0	0	1	1	37.4	0.04	49.2
1	0	0	100	7	2	79	22.5	132	71.5	216	77	139	12.4	5.2	67.2	1	1	1	0	0	1	1	20	0.01	15.7
1	0	0	100	7.06	2	67	21.3	177	112.5	194	77	117	13.9	5.7	81.7	2	1	1	0	1	1	1	91.1	0.02	16.3
1	0	0	100	7.08	2	66	26	126.5	77	189	77	112	13.5	5	82.3	1	1	1	1	1	1	1	14.2	0.02	18.4
1	0	0	100	5.16	2	66	23.1	121.5	76	212	77	135	13.5	5.1	73.6	2	1	1	1	1	1	1	24.7	0.02	37.7
1	0	0	100	6.84	2	73	27.9	133.5	73	274	77	197	15.1	5.5	73.8	2	1	1	1	0	1	1	33.1	0.09	9.13
1	0	0	100	7.01	2	66	23.6	144.5	77	220	77	143	13.1	6	77.5	2	1	1	0	0	0	1	11.9	0.02	40.7
1	0	0	100	7.01	2	73	20.6	157	90	205	77	128	13.2	5	73.8	1	1	1	0	1	1	1	60.2	0.01	14.7
1	0	0	100	4.62	2	66	25	101	62.5	227	77	150	11.9	5.4	62.7	2	1	1	1	0	1	1	10.5	0.02	97.4
1	0	0	100	4.63	2	70	24.3	123.5	70.5	243	77	166	13.9	5.4	75.3	2	1	1	1	0	1	1	139.1	0.05	15.5
1	0	0	100	6.95	2	67	20	125	70	186	77	109	14	5	73.1	2	1	0	0	1	1	1	30	0.02	25.2
1	0	0	100	6.98	2	74	28.5	132	78	232	77	155	13.8	5.1	73.2	2	1	0	0	0	1	1	16.1	0.04	15.6
1	0	0	100	3.79	2	69	19.3	168	101	282	77	205	13.3	5.4	72.1	1	1	1	0	0	1	1	17.1	0.03	18.6
1	0	0	100	6.83	2	74	24.2	132.5	70.5	218	77	141	14	6.9	73.2	1	1	1	0	1	0	1	48.5	0.05	3.41
1	0	0	100	4.59	2	75	22	131	69.5	192	77	115	11	5.7	77.2	1	1	1	1	1	1	1	11.4	0.01	51.5
1	0	0	100	4.61	2	77	24.9	163	81.5	189	77	112	13.9	5.5	71.7	1	1	1	0	1	1	1	22	0.05	10.5
1	0	0	100	4.69	2	67	21.1	143	79	190	77	113	13.4	5.5	81.7	2	1	1	0	1	1	1	33.3	0.05	25.6
1	0	0	100	5.37	2	71	17.1	126	65	210	77	133	12.4	6.4	79.4	1	1	1	0	1	1	1	9.1	0.01	24
1	0	0	100	5.37	2	73	21.9	125.5	76	251	77	174	12.5	5.4	51.7	1	1	1	1	0	1	1	16.7	0.05	15.2
1	0	0	100	5.06	2	70	18.4	159.5	83.5	224	77	147	14	5.2	75.3	2	1	1	0	0	1	1	13	0.01	57.5
1	0	0	100	6.86	2	73	18	138.5	81	209	77	132	11.6	5.2	73.8	2	1	1	1	1	1	1	42.4	0.02	36.5
1	0	0	100	6.87	2	66	19.3	112	69	204	77	127	11.3	5.7	73.6	2	1	1	1	1	1	1	20.9	0.01	15.4
1	0	0	100	6.87	2	66	25.3	194	108.5	224	77	147	14.1	5.6	77.5	2	1	1	0	0	1	1	55.3	0.08	26.5
1	1	0	100	6.87	2	77	18.1	98	57.5	203	77	126	12.7	5.4	68.2	2	1	1	1	0	1	1	7.7	0.01	11.8
1	1	0	100	6.98	2	68	27.2	114	72.5	186	77	109	12.9	5.4	76.4	2	1	1	0	0	1	1	9.3	0.01	5.52
1	0	0	100	5.37	2	83	21.8	137	62.5	185	77	108	11.2	5.6	65.3	1	1	1	1	1	1	1	21.3	0.02	33.2
1	0	0	100	6.97	1	66	21.5	141.5	85	177	77	100	13.9	5.7	63.5	2	1	0	0	1	0	1	226.7	0.08	1
1	0	0	100	4.1	1	79	24.6	146	82	222	77	145	15.1	4.9	69.1	1	1	1	0	0	0	1	13.5	0.01	7.58
1	0	0	100	6.8	1	68	19.9	121	75.5	157	77	80	15	4.9	78.8	2	0	0	1	1	1	1	27.1	0.03	131
1	0	0	100	5.06	1	66	22.1	151	93	257	77	180	13.2	5.4	79.9	2	1	0	0	0	1	1	7.2	0.01	3
1	0	0	100	4.64	2	68	27.4	130.5	72.5	187	77	110	13.8	5.5	76.4	2	1	0	1	0	1	1	9	0.18	21.3
1	0	0	100	6.8	2	69	21.8	152.5	74	182	77	105	10	5.6	75.9	2	1	1	0	1	1	1	10.4	0.15	10.3
1	0	0	100	4.62	2	70	28.2	154.5	89.5	203	77	126	13.3	5.9	75.3	2	1	1	0	1	1	1	25.4	0.1	33
1	0	0	100	5.04	2	72	26.4	156.5	82	167	77	90	12.3	6.7	78.9	2	1	1	0	1	0	1	13.1	0.19	20.9
1	0	0	100	7.08	2	70	21.5	123.5	80.5	239	77	162	13.7	5	80.0	1	1	1	1	0	1	1	26.3	0.01	33.2
1	0	0	100	6.85	2	72	20.8	121	64	242	77	165	11.3	5.1	60.1	1	1	1	0	0	1	1	7.9	0.04	16.6
1	0	0	100	6.86	2	65	26	134.5	77	189	77	112	10.7	5.4	78.0	2	1	1	1	0	1	1	64.2	0.07	48.4
1	0	0	100	6.99	2	69	23.1	111	67	213	77	136	8.9	5.5	72.1	2	1	0	1	1	1	1	38.5	0.01	50.2
1	0	0	100	4.63	2	69	34.2	123.5	82	231	77	154	14.3	5.6	72.1	2	1	0	0	0	1	1	7.2	0.06	39.6
1	0	0	100	4.64	2	75	18.4	135	74.5	242	77	165	11.9	5.4	72.7	2	1	1	0	0	1	1	35.7	0.03	19.6
1	0	0	100	6.96	2	72	21.2	120	77.5	194	77	117	13.4	5.3	74.3	1	1	1	1	1	0	1	60.8	0.01	40.4
1	0	0	100	7.15	2	79	23.3	126	77	241	77	164	13.2	5.2	70.7	2	1	1	1	0	1	1	7.1	0.04	34.1

1	1	0	100	6.95	2	67	20.1	154.5	74	212	77	135	12.6	6.5	73.1	2	1	1	0	1	0	1	7.3	0.02	26.2
1	1	1	0.3	0.3	1	77	17.9	103	63.5	182	77	105	13.9	5.1	66.7	2	0	0	0	1	1	1	55.9	0.02	40.1
1	0	0	100	5.06	1	71	25.6	177.5	93.5	204	78	126	14.7	5.5	73.1	1	1	0	0	1	1	1	6.3	0.15	42.3
1	0	0	100	6.87	1	69	18.9	130	69.5	202	78	124	14.7	5.4	83.4	2	0	0	1	1	1	1	21.9	0.27	22.2
1	0	0	100	7	1	69	22.4	145	82.5	192	78	114	15.6	4.6	78.3	2	1	0	0	1	1	1	8.8	0.04	38.1
1	0	0	100	7.07	1	71	23.2	127	79	261	78	183	14.3	6	77.2	2	1	1	1	0	1	1	57.7	0.01	6.95
1	0	0	100	4.58	1	68	22.4	138.5	81.5	240	78	162	15	5.4	55.8	2	1	0	1	0	1	1	3.3	0.06	20.3
1	0	0	100	4.59	1	66	21.1	118	80.5	231	78	153	13.6	5.7	75.7	2	1	0	1	0	1	1	94.4	0.02	38.8
1	0	0	100	4.62	1	66	23	153	90.5	182	78	104	14.3	5.3	63.5	2	0	0	0	1	1	1	7.4	0.03	14.5
1	0	0	100	4.63	1	66	26	145	83	231	78	153	15.3	5.4	79.9	2	1	0	0	0	1	1	11.2	0.02	13.4
1	0	0	100	4.59	1	71	20.4	109.5	70.5	206	78	128	13.2	5.7	69.6	2	1	1	1	1	1	1	5.9	0.01	13.3
1	0	0	100	4.67	1	67	21.6	155.5	80	194	78	116	16.7	5.1	79.4	2	1	0	0	1	1	1	72.9	0.02	24.4
1	0	0	100	6.96	1	73	19	112.5	75	198	78	120	11.8	5.9	72.0	2	1	1	1	1	1	1	43.4	0.03	7.51
1	0	0	100	6.87	1	67	23.2	123	83	180	78	102	16.1	5.8	75.2	1	0	1	1	1	1	1	56.1	0.03	6.81
1	0	0	100	5.04	1	66	24.3	130.5	66.5	186	78	108	13.8	6.7	72.1	2	1	0	1	1	0	1	174	0.04	29.3
1	0	0	100	7	2	70	25.2	142.5	74	224	78	146	13.9	5.8	80.0	1	1	1	0	0	1	1	206.9	0.28	17.8
1	0	0	100	5.08	2	68	23.6	105	67	161	78	83	13.4	5.7	76.4	2	1	1	0	1	1	1	25.2	0.14	9.89
1	0	0	100	7.05	2	73	30.3	132.5	74	216	78	138	14.2	5.5	73.8	1	1	1	1	1	1	1	28.1	0.06	4.15
1	0	0	100	7.07	2	74	23.5	97	58.5	209	78	131	12.2	4.8	77.8	2	1	1	1	1	1	1	43.4	0.02	16.1
1	0	0	100	7.08	2	71	24	132	79	214	78	136	14.2	5.3	60.5	2	1	1	0	1	1	1	17.6	0.02	9.92
1	0	0	100	7.08	2	75	21.5	124.5	65	264	78	186	14.4	5.2	77.2	1	1	1	1	0	1	1	45.5	0.03	9.76
1	0	0	100	6.87	2	71	22.9	103.5	60.5	204	78	126	13.6	5.4	74.8	2	1	0	1	1	1	1	3.6	0.01	29.2
1	0	0	100	6.84	2	67	25.9	116	65.5	228	78	150	12.8	5.3	81.7	2	1	0	1	0	1	1	6.1	0.04	3.72
1	0	0	100	6.85	2	72	20.6	170.5	87.5	186	78	108	12.4	5.5	70.6	2	1	1	0	1	1	1	5.4	0.01	78.8
1	0	0	100	6.85	2	65	22.1	115.5	83	187	78	109	13.4	5.3	63.1	2	1	0	1	1	1	1	4.7	0.04	34.1
1	0	0	100	7.02	2	68	22.2	117.5	73	212	78	134	12.4	5.7	72.6	1	1	1	1	1	1	1	8	0.01	12.7
1	0	0	100	5.07	2	70	23.1	101.5	65.5	239	78	161	11.8	5.7	75.3	1	1	1	1	0	1	1	16.4	0.05	17.9
1	0	0	100	6.99	2	68	21.5	111	66.5	223	78	145	12.3	5.2	53.6	2	1	0	0	0	1	1	27.2	0.01	15.7
1	0	0	100	7	2	65	25.3	113	72	224	78	146	13.1	5.2	74.2	2	1	1	0	0	1	1	13.8	0.06	28.7
1	0	0	100	4.64	2	68	22.5	113	69	238	78	160	9.4	5	61.8	2	1	1	1	0	1	1	11.9	0.01	31.2
1	0	0	100	5.51	2	69	26	140	78.5	208	78	130	13.9	7.7	80.5	1	1	1	0	1	0	1	24.1	0.04	13.8
1	0	0	100	6.96	2	72	24.2	106	70.5	196	78	118	12.9	5.5	70.6	1	1	1	1	0	1	1	22.6	0.09	24.9
1	0	0	100	6.84	2	67	24.4	125.5	76	204	78	126	14.4	5.3	76.9	2	1	1	1	1	1	1	4.4	0.02	5.9
1	0	0	100	4.59	2	71	18	118	79	179	78	101	11.3	4.7	74.8	2	1	1	1	1	1	1	5.8	0.01	49
1	0	0	100	4.62	2	65	24.5	122.5	68.5	229	78	151	13.3	5.2	78.0	2	1	1	1	0	1	1	19.2	0.07	23.6
1	0	0	100	6.96	1	82	22.3	103.5	58.5	189	78	111	13	5.3	67.6	1	1	0	1	1	1	1	5.4	0.01	13.3
1	0	0	100	7.01	2	80	22	125	67	200	78	122	12.7	5.8	74.6	1	1	1	0	0	1	1	13.3	0.05	5.6
1	1	0	100	6.87	2	84	21.7	153.5	93	186	78	108	13.5	5	72.5	1	1	0	0	1	1	1	24.9	0.02	211
1	0	0	100	7.04	1	67	21.1	143.5	82.5	236	78	158	13.7	5	84.6	2	1	0	0	0	1	1	9.3	0.4	15.5
1	0	0	100	7.04	1	71	23.2	108.5	66.5	231	78	153	14.1	5.8	61.3	2	1	0	1	0	1	1	3.7	0.03	4.92
1	0	0	100	5.06	1	69	26	141	78.5	192	78	114	15.7	6	78.3	2	1	0	0	1	1	1	26.3	0.06	16.5
1	0	0	100	4.67	2	70	23.1	141	79.5	216	78	138	13.6	6.5	75.3	1	1	0	0	1	0	1	92.4	0.2	30.8
1	0	0	100	5.37	2	65	31.3	129.5	79	193	78	115	12.4	7.7	78.0	2	1	1	0	0	0	1	107.3	1.67	16.4
1	0	0	100	6.87	2	74	22.4	134	79.5	245	78	167	13.6	5.1	73.2	2	1	1	0	0	1	1	23.7	0.13	21.1
1	0	0	100	6.87	2	74	17.9	135.5	66	194	78	116	12	5.6	69.6	2	1	1	1	0	1	1	5.5	0.01	91.3
1	0	0	100	6.87	2	78	18.3	109.5	68	223	78	145	12.5	5.6	67.7	1	1	0	1	0	1	1	6.3	0.02	40.2
1	0	0	100	6.85	2	75	15.9	106	64	177	78	99	13.3	4.9	69.1	2	1	1	1	1	1	1	9.7	0.01	48.9
1	0	0	100	4.62	2	68	23.3	176.5	87.5	217	78	139	12.8	5.8	81.1	2	1	1	0	1	1	1	22.1	0.02	28.8
1	0	0	100	6.44	2	69	21.5	131.5	72	196	78	118	12.3	8.6	72.1	2	1	1	1	1	0	1	26.5	0.02	9.58
1	0	0	100	5.06	2	69	22.6	164.5	91	201	78	123	12.5	5.3	72.1	2	1	1	0	1	1	1	45.7	0.09	12.8
1	1	0	100	6.97	2	77	21.4	108	70	229	78	151	13	5.1	71.7	1	1	1	1	0	1	1	12.7	0.18	0.05

1	1	0	100	5.08	2	71	21.5	111	65	236	78	158	13.4	5	79.4	2	1	1	1	0	1	1	18.2	0.02	32.4
1	1	0	100	4.63	2	75	26.3	136	83	231	78	153	13.8	5.5	69.1	2	1	1	0	0	1	1	176.9	0.04	4.2
1	1	0	100	6.45	2	68	23.8	131	74	225	78	147	10.6	5.4	81.1	2	1	1	1	0	1	1	254	0.01	23.1
1	1	0	100	4.58	2	83	23.6	122	64.5	198	78	120	13.3	5.1	65.3	1	1	1	1	1	1	1	33.3	0.24	31
1	1	0	100	4.59	2	82	26.7	120.5	72.5	204	78	126	13.3	5.8	65.8	2	1	1	1	1	1	1	5.3	0.04	57.9
1	0	0	100	6.8	1	70	21.9	121.5	73	220	79	141	15.8	7.5	70.1	2	1	1	0	0	0	0	5.3	0.17	98.5
1	0	0	100	7.07	1	75	21.8	123.5	65	206	79	127	13.3	4.9	75.1	2	0	0	0	1	1	1	14.6	0.01	38.3
1	0	0	100	7.21	1	67	21.6	130.5	66.5	234	79	155	14.5	5.1	79.4	2	1	0	1	0	1	1	24.3	0.04	12.1
1	0	0	100	6.15	1	69	18.3	164	89	179	79	100	12.9	5	74.1	1	0	0	0	1	1	1	22.9	0.05	34.9
1	0	0	100	6.97	1	74	22.1	141.5	79.5	244	79	165	14	5.6	60.0	1	1	0	0	0	1	1	23.8	0.02	13.7
1	0	0	100	6.98	1	66	19.6	103	63.5	214	79	135	13	5.5	85.2	2	0	0	1	1	1	1	12.2	0.01	8.4
1	0	0	100	4.63	1	66	23.2	137	83.5	238	79	159	15.5	5.5	79.9	2	1	0	0	0	1	1	7.4	0.03	8.2
1	0	0	100	4.64	1	69	24.2	123.5	76.5	199	79	120	13.6	5.1	83.4	2	0	0	1	1	1	1	6	0.06	37.2
1	0	0	100	6.97	1	68	22.8	123.5	76	222	79	143	15.2	5.3	71.1	2	0	0	1	0	1	1	11.8	0.04	66.4
1	0	0	100	6.82	1	67	23.7	123	77	191	79	112	16.3	5.8	71.6	2	1	0	1	1	1	0	11.7	0.05	143
1	0	0	100	6.83	1	65	22.9	103.5	74	195	79	116	14.2	5.4	80.5	2	1	0	1	1	1	1	3.1	0.03	38.3
1	0	0	100	6.84	1	65	19.9	130	74.5	157	79	78	14	5.2	92.5	2	1	0	1	1	1	1	23.2	0.01	32.3
1	0	0	100	6.79	1	67	26.2	120.5	73.5	161	79	82	12.4	5.1	79.4	2	1	0	1	1	1	1	5.3	0.02	7.47
1	0	0	100	5.04	1	75	23.6	115	72	186	79	107	13.7	5.5	71.0	2	1	0	1	1	1	1	6.4	0.03	21.4
1	0	0	100	6.98	2	68	20.6	136.5	75.5	190	79	111	13.3	5.4	72.6	2	1	1	0	1	1	1	5.3	0.14	4.15
1	0	0	100	1.28	2	66	25.4	150	89.5	187	79	108	12.8	5.5	77.5	2	1	1	0	1	1	1	9.3	0.1	14.7
1	0	0	100	6.87	2	66	24.7	141.5	76.5	220	79	141	14.2	4.9	77.5	2	1	1	0	0	1	1	8.4	0.66	18
1	0	0	100	4.58	2	65	21.7	153	86	172	79	93	13.4	5.5	78.0	2	1	1	0	1	1	1	47.2	0.06	24.7
1	0	0	100	4.58	2	75	23.1	136	75	191	79	112	13.5	5.4	58.8	2	1	1	0	1	1	1	5.7	0.03	55.2
1	0	0	100	6.86	2	71	21.3	93	61	186	79	107	12.1	5.8	71.1	2	1	1	1	1	1	1	7.2	0.01	33.9
1	0	0	100	7.01	2	66	18.3	111.5	63.5	243	79	164	11.9	5.5	73.6	2	1	1	1	0	1	1	4	0.03	23.1
1	0	0	100	5.08	2	65	19.9	119	74.5	198	79	119	12.8	5.2	78.0	2	1	1	1	1	1	1	5.1	0.02	17.8
1	0	0	100	4.64	2	72	26.2	134	70.5	152	79	73	13.5	6.6	74.3	1	1	1	0	1	0	1	18.3	0.04	15.2
1	0	0	100	4.68	2	69	23.4	155.5	82	222	79	143	14.4	9.1	80.5	1	1	1	0	0	0	1	56.2	0.03	5.75
1	0	0	100	4.69	2	67	21	94	61	215	79	136	12.9	5.8	81.7	2	1	1	1	1	1	1	13.4	0.02	5.29
1	0	0	100	6.85	2	70	17.6	140.5	70	213	79	134	11.2	6	71.6	2	1	1	0	1	1	1	30.2	0.01	7.41
1	0	0	100	6.87	2	73	20.7	99	62	220	79	141	12.7	5.2	73.8	2	1	0	1	0	1	1	7.4	0.04	15.4
1	1	0	100	6.87	2	78	22.9	105	65	223	79	144	12.4	6.4	71.2	2	1	1	1	0	1	1	14.8	0.03	5.56
1	1	0	100	6.8	2	66	23.4	110.5	68	179	79	100	11.5	6	77.5	2	1	1	1	1	1	1	24.1	0.03	28.8
1	0	0	100	6.96	1	80	18	157.5	75.5	163	79	84	13.1	5	68.6	2	0	0	0	1	1	1	2.6	0.05	0.05
1	0	0	100	6.85	1	79	27	103	62	222	79	143	15.6	5.7	65.8	2	1	0	1	0	1	0	21.8	0.16	60.5
1	0	0	100	6.97	1	73	22.9	110	62.5	202	79	123	14.3	4.4	76.1	2	1	0	0	1	0	0	18.7	0.16	25.8
1	0	0	100	7.03	1	69	20	116	68	235	79	156	13.8	5.7	83.4	2	1	0	1	0	1	1	35.9	0.01	4.28
1	0	0	100	6.97	1	73	19.2	133	71	205	79	126	15.1	6.2	72.0	2	1	0	1	1	1	1	5.8	0.08	26.7
1	0	0	100	6.98	1	68	18.7	115	77.5	209	79	130	10.7	6.1	55.8	2	1	1	1	1	1	1	4.8	0.06	7.25
1	0	0	100	6.97	1	74	20.7	161.5	81.5	177	79	98	14	5.1	71.5	2	1	0	0	1	1	1	19.3	0.01	40.9
1	1	0	100	4.61	1	79	24.2	130	71	242	79	163	13.7	5.4	69.1	2	1	0	0	0	1	1	11.2	0.03	90.8
1	0	0	100	7	2	69	20	134	69	214	79	135	12.7	4.7	34.1	2	1	1	0	1	1	1	23.7	0.01	41.4
1	0	0	100	6.86	2	67	24.8	117.5	63	229	79	150	13.6	5.4	62.2	2	1	0	0	0	1	1	10.4	0.02	12.8
1	0	0	100	7.23	2	67	26.5	164.5	93.5	261	79	182	12.7	6.2	76.9	2	1	1	0	0	1	1	187.9	0.08	21.6
1	0	0	100	5.06	2	76	19	119	69	188	79	109	12.7	5	76.7	1	1	1	0	1	1	1	50.5	0.01	34.3
1	0	0	100	6.98	2	68	27.8	131.5	65.5	193	79	114	12.7	5.4	76.4	1	1	1	0	0	1	1	10.5	0.01	6.2
1	0	0	100	6.8	2	70	24.3	131.5	68.5	248	79	169	12.6	7	75.3	2	1	1	1	0	0	1	6.9	0.05	33.9
1	0	0	100	4.61	2	71	28.9	112.5	67	210	79	131	12.4	5.3	74.8	1	1	1	1	1	1	1	27	0.01	51.9
1	0	0	100	4.62	2	76	22.2	122	70.5	184	79	105	13.2	8.1	58.4	2	1	1	0	1	0	1	16.9	0.08	15.5
1	1	0	100	4.64	2	77	24.8	140.5	62.5	204	79	125	12.7	5.5	71.7	2	1	1	0	1	1	1	449.2	0.06	131

1	0	0	100	6.87	2	80	19.4	128	65.5	199	79	120	11.8	5.8	70.2	1	1	1	0	1	1	1	59	0.04	78.6
1	0	1	6.6	6.6	1	65	26.6	135	82.5	250	79	171	16.4	5.2	51.3	2	1	0	0	0	1	1	75.1	0.08	34.3
1	0	0	100	7.07	1	75	28.7	157.5	90	200	80	120	14.1	4.9	71.0	2	1	0	0	1	1	0	101.7	0.27	119
1	0	0	100	6.86	1	69	18.5	162	101	210	80	130	13.4	5.4	83.4	2	1	0	0	1	1	1	10.1	0.23	18.8
1	0	0	100	6.86	1	66	25.1	135	80.5	233	80	153	15.3	5.4	79.9	2	1	0	1	0	1	1	22.7	0.05	159
1	0	0	100	5.16	1	69	19.4	121.5	75.5	227	80	147	14.5	5.6	70.6	2	1	1	1	0	1	1	23.7	0.02	2.21
1	0	0	100	6.87	1	71	22.7	108	61	170	80	90	11.9	5	69.6	2	1	0	1	1	1	1	9.4	0.03	20.5
1	0	0	100	6.97	1	72	21.9	125.5	68	216	80	136	15.7	11	69.1	1	1	0	1	1	0	1	188.4	0.03	2.18
1	0	0	100	4.6	1	69	21.4	134	84	158	80	78	13.1	5.2	78.3	2	1	0	0	1	1	1	5.9	0.04	111
1	0	0	100	5.37	1	75	23.2	149	83.5	195	80	115	15.6	5.2	67.7	2	1	0	0	1	1	1	13.9	0.02	8.15
1	0	0	100	6.86	1	73	24.6	162.5	97.5	202	80	122	13.6	4.7	72.0	2	0	0	0	1	1	1	165.1	0.01	12.3
1	0	0	100	5.16	2	69	26.6	106	61	221	80	141	13.1	5.7	46.8	1	1	1	0	0	1	1	25.1	1.02	6.52
1	0	0	100	7	2	71	27.8	143.5	78.5	231	80	151	14.7	5.7	74.8	2	1	0	0	0	1	1	71.2	0.17	3.61
1	0	0	100	4.64	2	65	23.9	99	62.5	169	80	89	11.7	5.9	82.8	2	1	1	1	1	1	1	13	0.34	13.2
1	0	0	100	6.44	2	67	21.6	97.5	55	176	80	96	11.1	5.4	81.7	2	1	1	1	0	1	1	15.2	0.25	20.6
1	0	0	100	7.07	2	68	23.1	146.5	79.5	241	80	161	14.7	4.9	76.4	2	1	1	0	0	1	1	48.1	0.03	2.72
1	0	0	100	7.08	2	68	17.7	123.5	70.5	212	80	132	12.6	5.6	87.3	1	1	1	1	1	1	1	23	0.01	21.5
1	0	0	100	6.86	2	72	22.4	136.5	81	189	80	109	12.6	5.5	60.1	2	1	1	0	1	1	1	23.9	0.02	47.8
1	0	0	100	7.23	2	67	22.9	110.5	67	273	80	193	12.6	5.5	76.9	2	1	1	1	0	1	1	58.9	0.01	7.27
1	0	0	100	6.84	2	70	21.4	117	63.5	244	80	164	12	5.4	75.3	1	1	1	1	0	1	1	11	0.03	57.1
1	0	0	100	2.66	2	73	20.3	94	46.5	178	80	98	11.4	5.9	78.3	2	1	1	1	1	1	1	16.4	0.03	19.4
1	0	0	100	7.21	2	70	19	87.5	65.5	215	80	135	12.8	5.5	75.3	2	1	1	1	1	1	1	26.1	0.01	17.8
1	0	0	100	7	2	74	21.5	143.5	94	209	80	129	13.6	4.8	59.2	1	1	1	0	1	1	1	10.7	0.02	23.7
1	0	0	100	4.64	2	67	21.9	125.5	76.5	217	80	137	12.2	5.3	73.1	2	1	1	1	1	1	1	20.4	0.03	17.2
1	0	0	100	6.95	2	72	22.8	121	68.5	239	80	159	12.9	5.7	84.9	2	1	1	0	0	1	1	6.3	0.05	0.05
1	0	0	100	6.97	2	72	21.6	133.5	66.5	227	80	147	13.2	5.4	78.9	2	1	1	1	0	1	1	22.6	0.07	17.8
1	0	0	100	6.84	2	66	27.4	113.5	65.5	220	80	140	12.4	5.7	54.3	2	1	1	1	0	1	1	8.2	0.08	0.05
1	0	0	100	5.37	2	72	22	128.5	66.5	276	80	196	11.9	5.8	74.3	1	1	1	1	0	1	1	82	0.02	15.5
1	0	0	100	6.86	2	70	20.4	128	83	189	80	109	12.8	5	71.6	2	1	1	1	0	1	1	19.2	0.02	23.2
1	0	0	100	6.87	2	70	23.1	151.5	88	242	80	162	13	5	71.6	2	1	1	0	0	1	1	96.1	0.04	32.4
1	1	0	100	6.96	2	66	19.8	173	75.5	187	80	107	13.8	5.1	62.7	2	1	1	0	1	1	1	32.1	0.02	64.8
1	0	0	100	1.89	2	81	18.5	130	69	216	80	136	13.4	5.5	69.7	1	1	1	1	0	1	1	192.6	0.03	40.4
1	0	0	100	6.85	2	83	19.9	159.5	82.5	211	80	131	10.5	5.6	68.7	1	1	1	0	0	1	1	62.1	0.01	70.9
1	0	0	100	5.06	1	79	22.1	157.5	76.5	195	80	115	14.1	5.4	69.1	2	1	0	0	1	1	1	13.3	0.13	71.7
1	0	0	100	6.85	2	76	25.6	100	61.5	244	80	164	13.5	5.3	72.2	2	1	1	1	0	1	1	12.4	0.25	29.5
1	0	0	100	6.84	2	72	23.9	107.5	64.5	200	80	120	12.3	5.6	60.1	2	1	1	0	1	1	1	7.9	0.8	7.8
1	0	0	100	6.86	2	71	27	142	77	244	80	164	12.4	5.4	79.4	2	1	1	0	0	1	1	33.2	0.03	76.2
1	0	0	100	7.04	2	71	23.5	114.5	64.5	191	80	111	10.3	5	74.8	2	1	1	1	1	1	1	10	0.01	4.71
1	0	0	100	6.95	2	75	21.5	122.5	67	227	80	147	13.3	4.7	77.2	1	1	1	0	0	1	1	25.9	0.01	103
1	1	0	100	6.87	2	79	23.4	147	71	236	80	156	14.4	5.8	70.7	1	1	1	0	0	1	1	21.4	0.37	46.2
1	1	0	100	4.69	2	78	25.8	179.5	89	198	80	118	12.3	5.6	67.7	2	1	1	0	1	1	1	36.5	0.02	145
1	1	0	100	7.03	2	69	22.1	129.5	72.5	250	80	170	13.4	5.3	80.5	2	1	1	0	0	1	1	20.7	0.01	8.65
1	1	0	100	6.84	2	87	20.4	125	71	247	80	167	12.7	5.3	63.5	2	1	1	0	0	1	1	71.5	0.07	95
1	0	1	1.2	1.2	2	69	23	132	78	245	80	165	13.6	6	75.9	2	1	1	0	0	1	1	23.7	0.01	24.9
1	0	1	6.7	6.7	2	69	22.1	111.5	64.5	218	80	138	11.7	5	75.9	2	1	1	1	1	1	1	19.5	0.02	48.7
1	0	1	4.6	4.6	2	73	29	160.5	80	219	80	139	14.3	6.4	70.1	2	1	1	0	1	1	1	121.3	0.8	14.3
1	0	1	4.5	4.5	2	75	23.1	140.5	80	229	80	149	13.6	4.9	72.7	1	1	1	0	0	1	1	9.7	0.02	55
1	0	0	100	5.16	1	71	19.2	149.5	91.5	254	81	173	15.1	5.7	73.1	2	0	0	0	0	0	1	45.5	0.23	15.8
1	0	0	100	7	1	69	25.3	155.5	98	202	81	121	14	5.1	74.1	1	1	0	0	1	1	1	20.3	0.02	42.2
1	0	0	100	5.15	1	68	24.1	121	83	160	81	79	15.5	5.6	62.6	2	0	0	1	1	1	1	6.3	0.02	12.7
1	0	0	100	6.86	1	74	20.2	130.5	84.5	215	81	134	13.8	5.8	75.6	2	1	0	1	1	1	1	15.9	0.04	30.1

1	0	0	100	5.06	1	66	20.3	99.5	62.5	181	81	100	13.8	5.4	75.7	2	1	0	1	1	1	1	3.5	0.01	63.4
1	0	0	100	6.79	1	72	22.5	141.5	77	232	81	151	12.6	5.7	69.1	2	1	0	0	0	0	1	94.5	0.07	45.3
1	1	0	100	5.06	1	78	21.5	155	86.5	165	81	84	15.4	5.4	66.3	2	1	0	0	1	1	1	23.4	0.09	15.4
1	0	0	100	6.87	2	67	23.8	120	75.5	191	81	110	14.6	5.1	76.9	2	1	1	0	1	1	1	14.3	0.52	6.92
1	0	0	100	7.21	2	67	20.1	118.5	72.5	246	81	165	13.8	5.6	76.9	2	1	1	1	0	1	1	8.4	0.04	28.1
1	0	0	100	6.87	2	69	22.2	100	55	243	81	162	12.3	5.9	75.9	2	1	1	1	0	1	1	6.3	0.02	28.6
1	0	0	100	6.88	2	70	22.1	157	75	358	81	277	13.1	5.4	52.8	2	1	1	0	0	1	1	15.3	0.06	19.9
1	0	0	100	4.07	2	76	24.3	100	65.5	197	81	116	12.5	5.9	68.6	1	1	1	1	1	1	1	3.4	0.03	33.1
1	0	0	100	5.07	2	65	21.8	138	84.5	234	81	153	12.7	6	78.0	2	1	1	1	0	1	1	14.5	0.02	11.6
1	0	0	100	6.97	2	67	21.7	189	103	248	81	167	10.8	5.5	13.7	2	1	1	0	0	1	1	1017.1	0.06	80.9
1	0	0	100	6.97	2	69	24.6	126	66.5	190	81	109	11.7	5.1	80.5	1	1	1	0	1	1	1	7.2	0.02	38.4
1	0	0	100	6.98	2	67	24.1	128.5	71	211	81	130	13.9	5.5	73.1	2	1	1	0	1	1	1	12.6	0.04	10.7
1	0	0	100	7	2	65	21	121	67.5	257	81	176	12.5	5.3	78.0	2	1	0	1	0	1	1	15.6	0.02	55.8
1	0	0	100	6.95	2	70	25.1	150	86.5	204	81	123	14.7	5.6	71.6	2	1	1	0	0	1	1	32	0.03	23.9
1	0	0	100	6.95	2	73	23.5	107	60.5	254	81	173	11.5	5.2	73.8	2	1	1	1	0	1	1	7.9	0.04	24.6
1	0	0	100	6.96	2	70	21.6	145.5	83	293	81	212	13.3	5.7	80.0	1	1	1	0	0	1	1	40	0.04	6.34
1	0	0	100	6.98	2	65	23.1	124.5	75.5	231	81	150	12.3	5.1	82.8	2	1	0	0	0	1	1	18.5	0.04	11.7
1	0	0	100	6.8	2	67	27.9	116	66	233	81	152	14.3	6.1	54.0	2	1	1	1	0	1	1	8.7	0.07	8.92
1	0	0	100	4.6	2	66	21.7	91	42	205	81	124	11.9	5.7	77.5	2	1	0	1	0	1	1	5.8	0.03	26.8
1	0	0	100	4.6	2	79	23.2	181.5	80	224	81	143	14.5	5.1	67.2	2	1	1	0	0	1	1	4.9	0.08	23
1	0	0	100	4.62	2	67	16.9	116	63.5	223	81	142	11.3	5.7	62.2	2	1	1	1	0	1	1	18.4	0.02	93.5
1	0	0	100	6.87	2	65	19.4	96	53	186	81	105	12.5	5.1	78.0	2	1	1	1	1	1	1	20.8	0.03	99.6
1	1	0	100	6.8	2	75	20.8	118	55	216	81	135	14.6	5.9	58.8	2	1	1	0	1	0	1	8.6	0.15	31.5
1	1	0	100	7.16	2	75	25.8	165.5	83.5	255	81	174	14.4	8.4	69.1	1	1	1	0	0	0	1	35.5	0.12	21.7
1	0	0	100	6.98	1	83	23.9	135.5	80.5	206	81	125	14.4	5.3	67.2	2	1	1	1	1	1	1	74.2	0.11	22.3
1	1	0	100	4.63	1	80	23.2	118	66.5	188	81	107	12.9	4.9	51.3	2	1	0	0	1	1	1	47.2	0.02	20.8
1	0	0	100	6.86	2	83	25	149	85.5	211	81	130	13	5.3	73.0	1	1	1	0	1	1	1	152.6	0.32	38.9
1	0	0	100	5.07	1	71	19.4	126.5	73.5	236	81	155	16.8	5.8	73.1	1	0	0	1	0	1	1	106.8	2.82	12.2
1	0	0	100	7.05	1	68	26.6	131	77.5	171	81	90	13.8	5.2	71.1	1	1	0	1	1	1	1	8.4	0.03	22.2
1	0	0	100	7.07	2	66	24.2	142.5	81.5	227	81	146	11.4	5.4	73.6	2	1	1	0	0	1	1	95.6	0.08	16.1
1	0	0	100	6.87	2	72	23.6	137.5	76	192	81	111	10.9	5.2	74.3	2	1	1	1	1	1	1	19	0.01	19.6
1	0	0	100	6.88	2	65	18.2	110.5	62	224	81	143	13.4	5.1	54.7	2	1	1	1	0	1	1	10	0.04	63.5
1	0	0	100	5.15	2	65	30.1	125	85.5	166	81	85	12.8	5.6	74.2	2	1	1	1	1	1	1	15.4	0.03	19.4
1	0	0	100	7.01	2	67	19.7	108.5	71	175	81	94	11.7	5.2	81.7	2	1	1	1	1	1	1	35.5	0.03	11.2
1	0	0	100	5.08	2	74	16.5	121.5	65	233	81	152	12.1	6.2	73.2	2	1	1	1	0	1	1	16.9	0.02	1.71
1	0	0	100	6.97	2	72	24.9	152	82.5	223	81	142	12.1	5.3	84.9	1	1	0	0	0	1	1	52	0.07	11.2
1	0	0	100	6.06	2	65	26.3	138	82	243	81	162	14.4	5.8	78.0	2	1	1	1	0	1	1	12.1	0.07	5.39
1	0	0	100	5.37	2	68	22.5	118	73.5	218	81	137	10	6	76.4	2	1	1	0	1	1	1	134.6	0.03	54.5
1	0	0	100	2.89	2	71	17.4	114	74.5	190	81	109	12.5	5.2	74.8	2	1	1	0	1	1	1	50.4	0.02	1.66
1	1	0	100	5.15	2	67	23.1	109.5	65.5	208	81	127	11.8	5.3	76.9	2	1	0	1	1	1	1	9.4	0.02	60.8
1	0	1	0.4	0.4	1	65	19.3	139	70.5	149	81	68	13.8	4.6	63.9	2	1	0	1	1	0	1	22.8	0.03	84.4
1	0	1	3.6	3.6	1	67	20.1	112	74	174	81	93	13.8	5.1	79.4	2	0	0	1	1	1	1	13.3	0.03	58.5
1	1	1	5.4	5.4	2	70	24	152	84	200	81	119	15.7	6.3	75.3	2	1	1	0	1	0	1	10.7	0.04	4.2
1	1	1	1.6	1.6	1	86	20.5	92.5	52.5	137	81	56	15.4	5.5	49.2	2	1	1	0	1	1	0	21.7	0.03	165
1	0	1	2.1	2.1	2	72	22.4	150.5	76	192	81	111	12.1	5.3	78.9	1	1	1	0	1	1	1	337.4	0.02	131
1	0	0	100	6.98	1	66	24.2	130.5	74	161	82	79	12.5	5.2	85.2	2	1	0	1	1	1	1	67.9	1.81	13.5
1	0	0	100	7.06	1	65	21	122	61.5	208	82	126	14	5.9	80.5	2	1	1	1	1	1	1	14.2	0.02	18.7
1	0	0	100	7.07	1	66	21.9	128	76.5	273	82	191	14	5.4	79.9	2	1	0	1	0	1	1	35.7	0.04	0.05
1	0	0	100	7.01	1	65	23.6	145	75	275	82	193	15.1	5.2	72.6	2	0	1	0	0	1	1	102.8	0.08	4.77
1	0	0	100	7	1	66	26.4	140	102	260	82	178	15.5	5.2	75.7	2	1	0	0	0	1	1	7.6	0.03	9.14
1	1	0	100	5.04	1	73	20.5	115.5	64.5	188	82	106	13.7	5.6	53.9	1	1	0	0	1	1	1	30.7	0.04	33.9

1	0	0	100	6.98	2	66	28.6	120.5	75.5	285	82	203	13.1	5	82.3	2	1	1	1	0	1	1	39.7	0.13	11.1
1	0	0	100	7.08	2	66	22.2	96.5	51	180	82	98	12.4	6.6	73.6	2	1	1	1	1	0	1	23.6	0.01	36.3
1	0	0	100	4.58	2	65	24.9	107	63.5	227	82	145	13.1	5.9	78.0	2	1	1	1	0	1	1	10.2	0.04	20.9
1	0	0	100	6.86	2	68	30.5	146	75.5	191	82	109	12.3	6.4	81.1	2	1	0	0	1	1	1	114.9	0.08	12.9
1	0	0	100	6.88	2	65	21.3	112	71	249	82	167	14.8	4.9	74.2	2	1	1	1	0	1	1	9.4	0.02	18.2
1	0	0	100	6.88	2	69	17.6	141	71.5	254	82	172	12.5	4.8	46.8	1	1	1	0	0	1	1	5.5	0.03	60
1	0	0	100	5.05	2	65	24.8	115	59	226	82	144	13.6	5.2	74.2	2	1	1	1	0	1	1	7.2	0.05	15.5
1	0	0	100	5.08	2	66	20	103.5	60.5	221	82	139	12.4	5.3	62.7	2	1	1	1	0	1	1	10.9	0.02	32.8
1	0	0	100	4.62	2	78	26.7	150	79	212	82	130	13.4	5.5	71.2	2	1	1	0	1	1	1	16.1	0.05	87.2
1	0	0	100	4.64	2	72	28.1	105	58	270	82	188	13.6	5.7	60.1	1	1	1	1	0	1	1	13.8	0.06	21.3
1	0	0	100	6.98	2	66	21.5	101.5	57	212	82	130	12.3	5.2	77.5	2	1	1	1	1	1	1	5	0.04	28.7
1	0	0	100	6.6	2	76	17.5	180.5	100.5	227	82	145	10.8	5.2	29.7	1	1	1	0	0	1	1	774.3	0.04	98
1	0	0	100	4.59	2	68	28.2	125.5	79	173	82	91	13.2	5.2	76.4	2	1	1	0	1	1	1	27.4	0.02	13.5
1	0	0	100	4.61	2	70	19.3	140.5	76.5	249	82	167	13.5	5.8	71.6	1	1	1	0	0	1	1	12.5	0.02	13.5
1	0	0	100	5.37	2	72	25.4	107	63	221	82	139	12.7	5.4	60.1	1	1	1	1	0	1	1	6.4	0.03	54.1
1	0	0	100	6.87	2	70	25.4	103.5	62.5	200	82	118	12.7	5.4	75.3	2	1	1	1	1	1	1	10.4	0.02	18.5
1	0	0	100	5.04	2	73	22.6	110	58	219	82	137	11.9	5.9	70.1	2	1	1	1	1	1	1	5.8	0.03	27
1	1	0	100	6.87	2	76	23.2	115	68	185	82	103	12.4	5.4	72.2	1	1	0	0	0	1	1	113.6	0.09	11
1	0	0	100	4.69	1	73	19.5	139	84.5	161	82	79	12.3	4.6	72.0	2	1	0	0	1	1	1	8	0.45	38.7
1	0	0	100	7	1	65	22	84	56.5	130	82	48	10.9	6	80.5	2	1	1	1	1	1	1	11.9	0.01	29.2
1	0	0	100	5.9	1	69	24.9	114	67	202	82	120	14.7	4.9	74.1	2	0	0	0	1	1	1	148.9	0.01	6.7
1	0	0	100	5.16	2	74	25.2	145	74	232	82	150	11.9	6.6	77.8	1	1	1	0	0	0	1	62.7	0.17	30.1
1	0	0	100	6.86	2	72	21.6	151	85	211	82	129	12	5.8	74.3	2	1	1	0	0	1	1	150.8	0.11	6.57
1	0	0	100	6.95	2	68	22.5	128	71.5	193	82	111	12.5	5.5	76.4	2	1	1	1	1	1	1	9.8	0.67	39.7
1	0	0	100	6.8	2	67	24	104.5	59.5	240	82	158	14.2	5.5	76.9	1	1	1	1	0	1	1	25.1	0.64	4.38
1	0	0	100	4.7	2	67	23.1	123.5	83.5	240	82	158	13.1	5.4	87.9	2	1	1	0	0	1	1	4.8	0.14	21.6
1	0	0	100	6.85	2	71	25.1	140	71.5	213	82	131	12.6	5.1	71.1	2	1	1	0	1	1	1	10.3	0.06	103
1	0	0	100	6.86	2	70	22.6	127.5	65.5	222	82	140	13.6	5.5	75.3	2	1	1	0	0	1	1	4.6	0.09	51.7
1	0	0	100	6.86	2	67	21.2	117	63.5	181	82	99	13.4	5.5	73.1	2	1	1	1	1	1	1	10.3	0.02	6.57
1	0	0	100	5.06	2	72	19.3	106	57	185	82	103	11.9	5.3	74.3	2	1	1	1	1	1	1	11.5	0.01	72.7
1	0	0	100	6.99	2	65	24.9	137.5	76	265	82	183	12.4	5.3	74.2	2	1	1	1	0	1	1	14.9	0.07	26
1	0	0	100	6.95	2	69	29.4	145.5	79.5	266	82	184	13	5.2	80.5	1	1	1	0	0	1	1	64.8	0.03	16.7
1	0	0	100	6.87	2	74	23.3	123	72.5	204	82	122	14.4	5.4	73.2	2	1	0	0	1	1	1	12.2	0.03	50.2
1	0	0	100	6.87	2	68	19.7	93.5	54	188	82	106	13.4	5.5	61.8	1	1	1	1	0	1	1	18.6	0.01	12.1
1	1	0	100	4.6	2	71	24.6	102	57	223	82	141	12.3	5.2	71.1	1	1	1	0	0	0	1	11.7	1.5	24.2
1	1	0	100	6.87	2	74	19.3	138	79	204	82	122	13.4	5.1	69.6	1	1	1	1	1	1	1	8.2	0.01	39.9
1	1	0	100	6.44	2	80	27.3	122	68	273	82	191	13.1	7	70.2	2	1	1	1	0	0	1	25.7	0.08	10.7
1	1	0	100	6.87	2	83	29.4	138.5	80.5	213	82	131	14.1	6.4	68.7	2	1	1	1	1	1	1	32	0.09	28.4
1	1	1	3	3	2	75	26.7	133.5	68.5	262	82	180	12.9	5.9	36.0	1	1	1	0	0	1	1	93.6	0.05	116
1	0	0	100	5.08	1	66	23.3	132.5	77.5	160	83	77	13.2	5.3	79.9	2	0	0	0	1	1	1	14.5	0.42	41.2
1	0	0	100	7	1	75	24	172.5	93	187	83	104	15.2	5.4	71.0	2	1	0	0	1	1	1	8.6	0.07	40.1
1	0	0	100	6.87	1	67	21.1	137	86	206	83	123	13.2	5.1	71.6	2	1	0	1	1	1	1	62	0.02	144
1	0	0	100	6.88	1	65	24.9	119	65.5	181	83	98	14.9	5.3	76.2	2	0	0	1	1	1	1	148.4	0.06	126
1	0	0	100	6.86	1	68	19.8	119	75	228	83	145	13.7	5.3	71.1	2	1	0	1	0	1	1	14	0.01	9.98
1	0	0	100	5.04	1	65	27.8	131	80.5	151	83	68	13.4	5.2	80.5	2	1	0	0	1	1	1	24.7	0.06	49.8
1	1	0	100	6.83	1	75	18.8	139.5	88	166	83	83	13.6	5	67.7	2	1	1	0	1	1	1	5.5	0.04	56
1	0	0	100	6.97	2	71	25.3	102	62	209	83	126	11.6	5.5	79.4	2	1	1	1	1	1	1	6.3	0.2	8.25
1	0	0	100	7.07	2	77	21.1	124.5	71	199	83	116	12.3	5.2	71.7	1	1	1	1	1	1	1	67.3	0.01	9.06
1	0	0	100	6.86	2	70	19	115	72.5	254	83	171	12.9	5.1	75.3	2	1	1	1	0	1	1	7.7	0.07	1.19
1	0	0	100	6.86	2	72	21.2	150	82.5	207	83	124	12.7	6.3	78.9	1	1	1	0	1	1	1	12	0.02	13.5
1	0	0	100	6.86	2	65	22.1	111	69	250	83	167	13.4	4.9	74.2	2	1	1	1	0	1	1	10.6	0.05	26.5

1	0	0	100	6.87	2	71	23.5	133.5	77.5	231	83	148	12.9	5.5	74.8	2	1	1	1	0	1	1	12.4	0.05	20.2
1	0	0	100	5.15	2	69	22.1	124.5	66.5	227	83	144	13.3	5.3	72.1	2	1	1	1	0	1	1	28.7	0.02	1.06
1	0	0	100	5.16	2	70	20.4	114	72.5	236	83	153	14.1	5.8	75.3	1	1	1	0	0	1	1	77.1	0.03	9.71
1	0	0	100	6.84	2	66	19.3	104.5	68.5	223	83	140	12.4	5.1	77.5	2	1	1	1	0	1	1	6.6	0.01	12.4
1	0	0	100	7.02	2	68	21.9	108.5	62	200	83	117	13.2	5.6	81.1	2	1	1	1	1	1	1	22.7	0.03	8.13
1	0	0	100	5.06	2	66	19.9	137.5	76.5	192	83	109	13.6	5.3	77.5	2	1	1	1	1	1	1	15.3	0.01	58.4
1	0	0	100	6.98	2	65	26.1	126.5	80.5	234	83	151	13.2	5.8	82.8	2	1	1	1	0	1	1	11.5	0.02	0.05
1	0	0	100	6.87	2	71	21.8	124	79	244	83	161	11.6	5.2	71.1	2	1	1	1	0	1	1	32.4	0.02	59.9
1	0	0	100	6.8	2	71	25.7	120	73	212	83	129	13.8	6.4	79.4	1	1	1	1	1	1	1	53.9	0.05	6.33
1	0	0	100	4.62	2	69	24.6	118.5	67	220	83	137	14.5	6.4	72.1	2	1	1	1	0	1	1	10	0.01	12.9
1	0	0	100	4.62	2	75	17.9	140	78.5	171	83	88	11.5	5.2	72.7	2	1	1	0	0	1	1	76.1	0.01	34.9
1	0	0	100	4.62	2	76	22.9	130.5	70.5	218	83	135	14.5	6.2	72.2	2	1	1	1	1	1	1	41.1	0.09	9.92
1	0	0	100	5.37	2	68	21.6	108.5	72	183	83	100	12.8	5.9	61.8	2	1	0	1	1	1	1	7.1	0.04	24
1	1	0	100	6.87	2	79	22.6	135	79	297	83	214	12.2	5.9	70.7	1	1	1	1	0	1	1	8.1	0.07	50.8
1	1	0	100	6.84	2	77	21.6	108	69	198	83	115	12.4	5.4	71.7	2	1	1	1	1	1	1	10.9	0.03	10.4
1	1	0	100	5.67	1	80	22.5	171	87	212	83	129	13.9	5.5	68.6	2	1	0	0	1	0	1	44.2	0.14	102
1	1	0	100	6.38	1	80	22	123	72.5	208	83	125	15.6	5	65.4	2	0	0	0	1	1	1	17.3	0.03	19
1	0	0	100	5.04	2	68	25.8	105	55	216	83	133	12.9	6.4	81.1	2	1	1	0	1	1	1	7.9	0.13	45.4
1	0	0	100	5.04	2	69	21.2	112.5	66.5	163	83	80	13.9	4.8	80.5	2	1	1	0	1	1	1	25.9	0.89	17.6
1	0	0	100	7	2	74	24.1	85.5	45.5	241	83	158	12.1	5	73.2	2	1	1	1	0	1	1	19.3	0.01	26.9
1	0	0	100	7.07	2	68	27.6	115	75.5	228	83	145	13.2	5.5	72.6	2	1	1	1	0	1	1	13.1	0.02	13.1
1	0	0	100	7.03	2	68	24.9	131.5	83.5	289	83	206	12.8	5	81.1	2	1	1	1	0	1	1	13.3	0.02	18.6
1	0	0	100	6.84	2	65	28.7	146	81.5	217	83	134	12	5.5	82.8	2	1	1	0	1	1	1	26.9	0.08	7.91
1	1	0	100	7.21	2	74	28.5	142.5	81	185	83	102	12.2	4.9	69.6	2	1	1	0	1	1	1	25.9	0.09	64
1	1	0	100	6.95	2	79	29.2	131	75	247	83	164	14.1	5.4	67.2	1	1	1	0	0	1	1	20.9	0.04	25.3
1	1	0	100	5.05	2	70	29.5	121	79	227	83	144	14.8	5.5	75.3	1	1	1	0	0	1	1	12.7	0.05	33.8
1	1	0	100	6.86	2	78	25	118.5	71	236	83	153	12.6	5.3	71.2	1	1	1	0	0	1	1	9.2	0.06	73.4
1	0	0	100	6.98	2	80	25.9	141.5	79	218	83	135	14.3	6.2	70.2	2	1	1	0	0	0	1	20	0.06	12.9
1	0	1	1.4	1.4	1	73	22.3	134	75	200	83	117	14.5	5.8	68.6	2	1	0	1	1	1	1	6.3	0.02	22.1
1	0	1	3.8	3.8	2	68	23.2	115	69	239	83	156	12	5.5	53.6	2	1	1	1	0	1	1	11.9	0.03	30.8
1	0	0	100	4.59	1	74	20.9	132	91.5	259	84	175	16.3	5.1	60.0	2	1	0	0	0	1	1	10.7	0.02	77.1
1	0	0	100	6.95	1	69	20.5	136	70	172	84	88	16.9	5.2	83.4	2	0	0	0	1	1	1	7.2	0.02	7.29
1	0	0	100	5.07	1	73	22.7	119	69	231	84	147	14	5.2	81.1	2	1	0	1	0	1	1	18.2	0.03	19.5
1	0	0	100	4.6	1	73	24.3	121.5	81.5	200	84	116	14	6	81.1	2	1	0	1	1	1	1	12	0.03	176
1	0	0	100	4.61	1	79	19.4	112.5	64	209	84	125	13.3	5.6	69.1	1	1	1	1	1	1	1	7.3	0.01	18.9
1	1	0	100	2.43	1	74	21.5	137	72	214	84	130	14.1	5.2	80.5	2	1	0	1	1	1	1	50.7	0.03	68.7
1	1	0	100	4.68	1	66	22.4	166.5	94.5	165	84	81	14.9	5	79.9	2	0	0	0	1	1	1	2.6	0.01	14.5
1	0	0	100	5.37	2	68	23.5	137	86.5	224	84	140	12.7	5.2	76.4	2	1	1	0	0	1	1	16.7	0.33	6.01
1	0	0	100	6.84	2	65	22.6	127	70.5	203	84	119	13.6	5.1	78.0	2	1	1	1	1	1	1	7.6	0.04	23.5
1	0	0	100	5.05	2	72	22.7	152.5	78	213	84	129	13.5	5.4	74.3	1	1	1	0	1	1	1	16.7	0.03	18.8
1	0	0	100	5.08	2	66	19.6	114	61	177	84	93	12.9	6	73.6	2	1	1	1	1	1	1	7.8	0.01	46.8
1	0	0	100	6.98	2	66	22.1	108.5	61	205	84	121	12.1	5.5	73.6	2	1	1	1	1	1	1	20.9	0.02	0.05
1	0	0	100	6.99	2	72	18.8	113.5	57.5	194	84	110	11.4	8	74.3	2	1	1	1	0	0	1	4.8	0.03	50.2
1	0	0	100	6.95	2	75	22.9	132	77	209	84	125	13.5	5.1	72.7	2	1	1	1	1	1	1	33.5	0.04	15.3
1	0	0	100	6.98	2	68	17.9	121.5	70	217	84	133	12.7	4.8	81.1	1	1	0	1	1	1	1	35.7	0.01	13.7
1	0	0	100	6.98	2	68	22.8	140	75	216	84	132	12.6	11.4	81.1	2	1	1	0	1	0	1	103.2	0.02	4.55
1	0	0	100	6.98	2	72	21.6	103.5	56	219	84	135	12.1	6.1	78.9	1	1	0	1	1	1	1	9.1	0.02	52.5
1	0	0	100	4.59	2	70	21.5	134.5	78	236	84	152	13.9	5.8	60.9	2	1	1	1	0	1	1	9.9	0.02	31
1	0	0	100	4.6	2	67	22.1	132.5	86.5	210	84	126	13.2	5.2	73.1	2	1	1	1	0	1	1	3.6	0.05	37.7
1	0	0	100	4.68	2	69	28.1	148.5	84.5	209	84	125	13	6	72.1	1	1	1	0	1	1	1	20.5	0.01	29.3
1	0	0	100	3.15	2	74	16.9	135	71.5	230	84	146	13.1	4.9	77.8	2	1	1	1	0	1	1	43.2	0.02	23.2

1	0	0	100	6.8	2	80	23.7	163	89	315	84	231	15	5.9	66.7	1	1	1	0	0	1	1	7.1	0.15	6.5
1	0	0	100	4.62	1	74	22.9	126.5	66.5	192	84	108	14.9	6.7	68.2	2	1	1	0	1	0	1	5.1	0.03	10.7
1	0	0	100	5.16	2	65	24.6	137	74.5	167	84	83	12.8	4.9	82.8	2	1	1	1	1	1	1	76.6	0.05	35
1	0	0	100	6.84	2	79	22.7	141	80.5	325	84	241	12.3	5.7	43.7	1	1	1	0	0	1	1	7.6	0.04	23.3
1	0	0	100	6.85	2	75	23.9	132	74.5	236	84	152	12.6	5.6	77.2	2	1	1	0	0	1	1	20.1	0.07	22.2
1	0	0	100	6.86	2	76	22.4	138.5	80.5	211	84	127	12.9	5.7	68.6	1	1	1	1	1	1	1	10.7	0.03	155
1	0	0	100	6.98	2	69	20.9	136	75	204	84	120	13.8	5.7	80.5	1	1	1	1	1	1	1	19.3	0.04	14.1
1	0	0	100	6.95	2	67	28.7	126	66	185	84	101	13.6	6	73.1	2	1	1	1	1	1	1	7.5	0.03	13.3
1	0	0	100	4.61	2	67	24.5	113.5	74	261	84	177	11.1	5.7	73.1	2	1	1	1	0	1	1	1317.3	0.06	33.9
1	0	1	2.3	2.3	2	71	22.3	154	92	191	84	107	13.8	4.8	74.8	1	1	1	0	1	1	1	32.5	0.02	30
1	0	0	100	2.91	1	71	24.3	135	98.5	186	85	101	13.8	5	73.1	2	1	0	0	1	1	0	11.7	0.65	70.8
1	0	0	100	6.87	1	75	18.9	105.5	74	215	85	130	14.7	5.6	67.7	2	0	0	1	1	1	1	15.4	0.02	6.35
1	0	0	100	6.97	1	71	23.6	166	89.5	180	85	95	15.3	4.8	82.2	1	1	0	0	1	1	1	30.8	0.01	32.1
1	0	0	100	2.77	1	77	17.5	148	82.5	228	85	143	12.1	5.2	78.8	2	1	0	0	0	1	1	23.8	0.03	96.8
1	0	0	100	6.85	1	76	21.8	162.5	90	206	85	121	14.9	5.3	70.5	1	1	0	0	1	1	0	40.2	0.04	200
1	0	0	100	7.21	1	68	20.7	105.5	62.5	191	85	106	15.2	5.5	71.1	2	1	0	1	1	1	1	4	0.02	6.76
1	0	0	100	5.15	2	67	22.2	136.5	82	191	85	106	14.2	5.6	73.1	2	1	1	1	1	1	1	44.2	0.04	19.6
1	0	0	100	6.84	2	78	19	110.5	66.5	231	85	146	12.3	5.4	44.0	1	1	1	1	0	1	1	56.5	0.04	36.5
1	0	0	100	4.64	2	72	21.6	129.5	78.5	230	85	145	12.7	5.8	70.6	2	1	1	1	0	1	1	11.6	0.02	18.6
1	0	0	100	6.8	2	68	21.6	112.5	75	189	85	104	14.5	5.2	72.6	2	1	0	1	0	1	1	5.7	0.04	10.7
1	0	0	100	6.8	2	68	22.1	97	64.5	268	85	183	12.7	5.7	61.8	2	1	1	1	0	1	1	8	0.01	8.84
1	0	0	100	0.7	2	68	21.3	112	64	227	85	142	12.4	5.5	81.1	2	1	1	1	0	1	1	6.8	0.01	15.9
1	0	0	100	4.69	2	70	16.8	117.5	77.5	193	85	108	12.4	8.9	75.3	2	1	1	0	1	0	1	16.6	0.04	31
1	0	0	100	4.7	2	74	20.5	123.5	63.5	210	85	125	14.1	5.6	73.2	2	1	1	1	1	1	1	31.6	0.03	75.4
1	0	0	100	4.58	2	70	23.7	120.5	73	230	85	145	14.1	5.5	86.1	2	1	1	0	0	1	1	26.3	0.02	23.4
1	0	0	100	7	2	70	21.7	123.5	86	212	85	127	12.9	5.4	80.0	1	1	1	0	1	1	1	9.1	0.01	10.9
1	0	0	100	6.87	2	76	29.6	112.5	73.5	224	85	139	12.6	5	68.6	2	1	1	1	0	1	1	4.9	0.04	45.2
1	0	0	100	6.98	2	75	21.3	159.5	99.5	160	85	75	13.5	5.1	69.1	1	1	1	0	1	1	1	36.3	0.01	130
1	0	0	100	6.85	2	72	24.3	146	83	220	85	135	14.4	5.9	70.6	2	1	1	0	0	1	1	24.8	0.06	7.06
1	1	0	100	2.74	2	76	26.3	119	73	175	85	90	11.8	4.5	76.7	2	1	1	1	1	1	1	38.2	0.55	172
1	1	0	100	5.15	2	69	19	106	62.5	235	85	150	12.6	5.7	75.9	2	1	1	0	0	1	1	4.4	0.01	7.17
1	1	0	100	6.86	1	84	24.6	114.5	67	199	85	114	13.6	4.9	55.9	2	1	0	1	1	1	1	88.5	0.03	79.8
1	0	1	6	6	2	70	27.2	122	72	255	85	170	12.9	5.5	75.3	2	1	1	0	0	1	1	57.2	0.08	0.05
1	1	1	2.6	2.6	2	74	22.9	145.5	81	213	85	128	12.9	5.5	77.8	2	1	0	0	1	1	1	14.2	0.06	12.9
1	1	1	0.9	0.9	2	69	23.4	135.5	88.5	208	85	123	12.1	5.1	75.9	1	1	0	1	1	1	1	88.9	0.02	25.3
1	0	1	4.1	4.1	2	72	26.8	160.5	88	263	85	178	15.3	5.4	70.6	2	1	1	0	0	1	1	138.5	0.12	126
1	0	0	100	4.62	1	67	23.2	164.5	90.5	232	86	146	13.9	6.8	75.2	2	1	0	0	0	0	1	23.1	0.14	74.6
1	0	0	100	4.96	1	77	23.4	130.5	74	212	86	126	13.9	5.4	47.1	2	1	1	1	1	1	1	76.9	0.03	0.05
1	0	0	100	5.37	1	71	20.3	142.5	84	205	86	119	14.7	5.1	73.1	1	0	0	0	1	1	1	9.4	0.03	14.1
1	0	0	100	6.87	2	69	21	125.5	68.5	219	86	133	12.7	5.4	61.4	2	1	0	1	1	1	1	6.1	0.12	66.4
1	0	0	100	7.06	2	70	23.8	166.5	87	289	86	203	13.8	5.4	60.9	2	1	1	0	0	1	1	14.6	0.05	14.1
1	0	0	100	7.07	2	65	19.9	124	66	270	86	184	12	5.5	74.2	2	1	0	1	0	1	1	9.2	0.01	12
1	0	0	100	6.87	2	75	19	137.5	84.5	253	86	167	13.2	5.4	72.7	2	1	1	0	0	1	1	59.4	0.03	4.43
1	0	0	100	6.95	2	75	20.8	137	74.5	235	86	149	13.1	6.1	83.1	2	1	0	1	0	0	1	27.7	0.05	14.2
1	0	0	100	6.79	2	71	23.4	125.5	82.5	213	86	127	13.7	5.7	71.1	1	1	1	1	1	1	1	24.8	0.04	57
1	0	0	100	4.7	2	69	21.9	127.5	79	242	86	156	13.5	5.5	72.1	1	1	0	1	0	1	1	18.6	0.05	38.7
1	0	0	100	4.7	2	67	21.9	124.5	69	191	86	105	13.8	5.7	76.9	2	1	1	0	1	1	1	3.4	0.02	12.9
1	1	0	100	7.07	2	71	20.8	128.5	78	250	86	164	12.4	5.3	71.1	2	1	1	1	0	1	1	13.2	0.07	23.1
1	1	0	100	7.08	2	73	17.9	124	73.5	240	86	154	12.8	5.2	78.3	2	1	1	1	0	1	1	29	0.01	22
1	1	0	100	6.84	2	73	21.7	129.5	74.5	210	86	124	12.1	5.3	70.1	2	1	1	0	0	1	1	24.6	0.02	17.7
1	0	0	100	6.98	1	77	24	128	66	168	86	82	14.1	4.7	66.7	2	1	0	0	1	1	1	33	0.16	24.8

1	0	0	100	7.07	1	76	30.4	156	86	238	86	152	14.1	4.9	67.2	2	1	0	0	0	1	1	10.5	0.06	71.5
1	0	0	100	4.63	1	70	24	146	83.5	192	86	106	15.2	5.1	61.7	2	1	0	0	1	1	1	27.8	0.06	39
1	0	0	100	4.63	1	65	23.7	156.5	79.5	203	86	117	13.9	5.1	80.5	2	0	0	0	1	1	1	11.9	0.01	8.66
1	0	0	100	4.59	1	74	17.9	143.5	76	209	86	123	14.8	5.4	80.5	2	0	0	0	1	1	1	16.3	0.06	39.9
1	1	0	100	7	2	75	25.6	133	82.5	236	86	150	13.9	5.3	72.7	2	1	1	1	0	1	1	12.7	0.04	8.32
1	1	0	100	6.96	2	71	26.7	114	79	227	86	141	12.1	5.5	79.4	1	1	0	0	0	1	1	37.9	0.03	0.05
1	1	0	100	4.68	2	74	24.9	140	87.5	208	86	122	14.1	5	77.8	2	1	1	0	1	1	1	17.8	0.05	56.4
1	0	1	4.7	4.7	2	75	19.7	122.5	73.5	186	86	100	11.6	5.1	77.2	1	1	1	1	0	1	1	24.8	0.06	59.5
1	0	0	100	7.08	1	70	21.9	118	78.5	187	87	100	15.3	5.3	73.6	2	1	0	1	1	1	1	4.3	0.02	17.8
1	0	0	100	4.67	1	66	23.7	150.5	96.5	259	87	172	17.5	5.2	75.7	1	1	0	0	0	1	1	192.4	0.05	19.9
1	1	0	100	3.19	1	70	19.2	106.5	61	182	87	95	12.2	5.9	77.7	2	1	0	1	1	0	1	15	0.06	20.8
1	0	0	100	4.58	2	74	22.6	161.5	80	212	87	125	13.2	5.5	59.2	2	1	1	0	1	1	1	27.7	0.02	44.4
1	0	0	100	4.59	2	72	20.6	129	70.5	199	87	112	12.7	5.6	70.6	2	1	1	0	0	1	1	21.4	0.03	16.9
1	0	0	100	4.59	2	67	19	110.5	70	246	87	159	14	5.6	54.0	2	1	0	1	0	1	1	8.1	0.01	20.4
1	0	0	100	6.85	2	70	23.2	109.5	61	207	87	120	13	5.4	60.9	2	1	1	1	0	1	1	6.1	0.03	27.4
1	0	0	100	5.08	2	69	27	121.5	68	202	87	115	13.5	5.5	72.1	2	1	1	1	1	1	1	6.1	0.08	19.9
1	0	0	100	6.87	2	65	16.9	121	72.5	238	87	151	13.5	5.3	82.8	2	1	0	1	0	1	1	70	0.03	35.3
1	0	0	100	5.04	2	66	23.6	122.5	82	205	87	118	12.6	5.7	77.5	2	1	0	1	1	1	1	21.7	0.04	44.4
1	1	0	100	7.07	2	75	26.6	154	83	241	87	154	13.8	5	58.8	2	1	1	0	0	1	1	40	0.02	64.5
1	1	0	100	7	2	82	18.1	164	90.5	231	87	144	13.4	4.9	69.2	2	1	1	0	0	1	1	54.4	0.02	68.6
1	0	0	100	7	1	72	21	134.5	74	213	87	126	15.6	5.6	60.9	2	1	0	1	1	1	1	11.7	0.06	5.91
1	0	0	100	7	2	77	24.1	139	69	215	87	128	13.3	5.2	50.3	1	1	1	0	1	1	1	8.8	0.17	13.8
1	0	0	100	0.68	2	70	15.7	148.5	100	211	87	124	13.8	5.6	80.0	1	1	0	0	1	1	1	152.4	0.14	18.2
1	0	0	100	4.59	2	71	23.6	134.5	86	220	87	133	14.4	5	71.1	2	1	1	0	0	1	1	12.5	0.01	15
1	0	0	100	6.87	2	79	24.4	144.5	90.5	216	87	129	14.2	5.3	67.2	2	1	1	0	1	1	1	11.8	0.06	40.9
1	1	0	100	7	2	75	26.1	165.5	81.5	312	87	225	13.3	5.2	69.1	2	1	1	0	0	1	1	32.1	0.03	51.4
1	0	1	4	4	2	70	28.7	112.5	67	260	87	173	12.9	5.1	75.3	2	1	1	1	0	1	1	42.2	0.07	26.5
1	0	1	0.7	0.7	2	68	18.8	145	97.5	174	87	87	14.3	5.1	81.1	2	1	0	0	1	1	1	78.7	0.33	32.9
1	0	1	6.8	6.8	2	74	26.8	123.5	89.5	206	87	119	14	5.3	69.6	2	1	1	0	1	1	1	36.8	0.02	201
1	0	0	100	6.97	1	72	24.2	113.5	68	185	88	97	15	5	69.1	1	1	0	0	1	1	0	11	0.14	82.5
1	0	0	100	6.82	1	67	25	126.5	84	215	88	127	15.9	5.2	71.6	1	1	1	1	1	1	1	136.4	0.06	12.1
1	0	0	100	6.96	2	74	31.4	128	74	211	88	123	13.5	4.9	77.8	1	1	0	1	1	1	1	17	0.17	14.7
1	0	0	100	7.08	2	65	21.9	118	72	222	88	134	14.4	5.2	89.1	2	1	1	1	0	1	1	66.9	0.04	14.2
1	0	0	100	6.87	2	69	24.9	140.5	78	188	88	100	13.4	6.8	75.9	2	1	1	0	1	0	1	38.1	0.05	28.5
1	0	0	100	6.87	2	71	21.9	115	63.5	244	88	156	12.9	5.2	79.4	2	1	1	1	0	1	1	19.5	0.02	39.3
1	0	0	100	6.87	2	67	25.7	107.5	59.5	266	88	178	13.3	5.5	76.9	2	1	1	1	0	1	1	5.3	0.04	8.18
1	0	0	100	5.16	2	68	20.7	130	85	243	88	155	12	5.5	72.6	2	1	1	1	0	1	1	19.8	0.07	10.6
1	0	0	100	6.85	2	68	27.9	127	73	281	88	193	12.9	6.5	76.4	2	1	1	0	0	0	1	26.8	0.02	15.8
1	0	0	100	6.85	2	74	18.8	147.5	87.5	204	88	116	12.8	5.8	59.2	2	1	1	0	1	1	1	30.2	0.03	35.3
1	0	0	100	6.85	2	74	20	127	68	233	88	145	11.1	5.5	73.2	2	1	1	1	0	1	1	11.1	0.01	18.9
1	0	0	100	7.21	2	69	20.9	121.5	73	248	88	160	13.6	5.1	80.5	2	1	1	1	0	1	1	17.9	0.03	7.74
1	0	0	100	6.86	2	73	22.2	139	75.5	176	88	88	12	5.1	78.3	2	1	1	1	0	1	1	21.9	0.03	32.2
1	0	0	100	5.06	2	76	23.5	137.5	89	217	88	129	13.5	5.4	72.2	2	1	0	1	1	1	1	23.5	0.01	45.9
1	0	0	100	6.8	2	73	22.2	141.5	82	230	88	142	13.8	5.5	73.8	2	1	1	0	0	1	1	7	0.05	16.8
1	0	0	100	6.96	2	77	18.3	115.5	63.5	234	88	146	13.6	5.6	68.2	1	1	1	1	0	1	0	13	0.01	0.05
1	0	0	100	6.87	2	70	29.3	109.5	73	229	88	141	14.3	4.8	80.0	2	1	1	1	0	1	1	26.6	0.02	11.3
1	1	0	100	7.01	2	79	20.8	129	72.5	216	88	128	13.6	5.1	70.7	2	1	1	1	0	1	1	6.8	0.02	11.5
1	0	0	100	6.84	2	65	18.3	149	85.5	209	88	121	10.5	6.5	54.7	2	1	1	0	1	0	1	11.2	1.46	57.7
1	0	0	100	6.87	2	65	22.8	120.5	66	207	88	119	13.5	5.5	82.8	2	1	1	1	1	1	1	21.2	0.07	2.95
1	1	0	100	5.16	2	77	26	121.5	69.5	209	88	121	14.1	4.8	68.2	1	1	1	0	1	1	1	61.2	0.02	18.1
1	1	1	5.9	5.9	2	66	23.7	162.5	97.5	219	88	131	14	5.5	73.6	2	1	0	0	1	1	1	17.8	0.03	11.4

1	0	0	100	5.04	1	66	29.2	133.5	78	152	89	63	13.8	5.8	79.9	2	1	0	0	1	1	1	11.5	2.9	20.4
1	0	0	100	7.05	1	69	21	141	80	196	89	107	11.7	4.7	78.3	2	1	0	0	1	1	1	22.8	0.03	25.3
1	0	0	100	4.59	1	70	20.1	124.5	74	193	89	104	13.6	4.9	61.7	2	0	0	1	1	1	1	3.7	0.02	20
1	0	0	100	6.86	1	67	18.7	107.5	77	227	89	138	15.8	5.9	75.2	2	1	0	1	0	1	1	4.8	0.02	31.8
1	0	0	100	7.01	1	75	19.4	157.5	88	168	89	79	13.8	5.5	75.1	2	1	0	0	1	1	1	14.9	0.01	27.2
1	0	0	100	7.01	1	71	23.1	152.5	81.5	231	89	142	14.4	7.2	82.2	1	1	0	0	0	0	1	20	0.06	28.2
1	0	0	100	4.69	1	66	24.6	136	84.5	194	89	105	15.4	5	75.7	2	1	0	0	1	1	1	6.5	0.02	23.7
1	0	0	100	5.05	1	68	24.6	142.5	83.5	170	89	81	14.2	5.3	78.8	2	1	0	0	1	1	1	10	0.03	32.5
1	0	0	100	5	2	66	16.9	113	63	245	89	156	13	5.4	77.5	2	1	1	1	0	1	1	21.6	0.09	21.3
1	0	0	100	6.86	2	65	20.2	92	65.5	193	89	104	11.5	5.8	78.0	2	1	1	1	0	1	1	7.6	0.02	24.6
1	0	0	100	5.16	2	73	21.7	145.5	79.5	206	89	117	12.9	5.5	70.1	2	1	1	0	1	1	1	11.8	0.03	9.82
1	0	0	100	4.67	2	66	24.5	133	71	224	89	135	13.4	5.5	62.7	2	1	1	0	0	1	1	6.7	0.04	31.4
1	0	0	100	7	2	68	26.6	137.5	86	253	89	164	15.9	4.8	76.4	1	1	1	1	0	1	1	12.5	0.06	13.2
1	0	0	100	5.04	2	70	26.8	145	80	206	89	117	13.1	5.2	80.0	2	1	1	0	1	1	1	1000	0.03	10.2
1	0	0	100	6.8	1	65	20.9	187.5	101.5	188	90	98	14.4	5.4	76.2	2	1	1	0	1	1	1	11.2	0.16	91
1	0	0	100	5.08	1	72	18.8	104.5	59.5	204	90	114	12.8	5.1	69.1	1	1	0	1	1	1	1	20.8	0.07	94.9
1	0	0	100	6.96	1	65	22	138.5	81	251	90	161	13	5	80.5	2	1	0	0	0	1	1	12.7	0.04	15.5
1	0	0	100	6.45	2	70	24.2	137	75.5	223	90	133	13.2	5.2	80.0	2	1	1	0	0	1	1	49.8	0.11	13.1
1	0	0	100	7	2	78	19	144.5	74	174	90	84	11.3	6.6	71.2	1	1	1	0	1	0	1	96.4	0.05	155
1	0	0	100	6.85	2	66	20	96.5	62	249	90	159	12.6	5.3	62.7	2	1	1	1	0	1	1	12.8	0.02	3.46
1	0	0	100	4.59	2	72	23.7	146.5	75	221	90	131	13.3	5.3	74.3	2	1	1	0	0	1	1	19.9	0.04	28.1
1	1	0	100	7.07	2	83	21.8	122	65	212	90	122	12	5.6	55.6	2	1	1	1	1	1	1	160.4	0.01	51.6
1	0	0	100	4.63	2	71	26.7	159.5	83.5	260	90	170	13.6	5.2	79.4	2	1	1	0	0	1	1	90.7	0.04	21.1
1	0	0	100	5.37	2	72	21.3	94	57	220	90	130	13.8	5.6	74.3	2	1	1	1	0	1	1	18.3	0.02	11.4
1	0	0	100	4.61	2	80	25.7	152.5	82.5	221	90	131	13.3	5.2	66.7	2	1	1	0	0	1	1	60	0.09	23.7
1	1	1	3.5	3.5	1	67	19.7	155.5	84	161	90	71	14.7	4.4	75.2	2	0	0	0	1	0	1	20.9	0.04	23.6
1	1	1	5.5	5.5	2	74	24.2	146	77.5	238	90	148	14.2	6.4	73.2	1	1	1	0	0	1	1	6	0.14	19.6
1	0	0	100	6.98	1	71	21.3	184.5	100.5	202	91	111	14.3	5.4	88.6	2	1	0	0	1	1	1	11.1	0.26	0.25
1	0	0	100	6.87	1	78	20.6	117.5	71	190	91	99	12.6	4.9	73.5	1	1	0	0	1	1	1	19.7	0.23	52.9
1	0	0	100	7	1	67	18.5	141.5	72	189	91	98	13.4	5.5	75.2	2	1	0	0	1	1	1	72.7	0.01	28.3
1	0	0	100	6.85	1	76	22.4	121.5	69.5	234	91	143	14.4	5.7	70.5	2	1	0	0	0	1	1	12.7	0.01	9.32
1	0	0	100	7.02	1	68	22.1	126	72	269	91	178	13.8	5.2	78.8	2	0	0	1	0	1	1	25	0.02	7.61
1	0	0	100	5.07	1	69	24.2	131.5	81.5	201	91	110	13.7	5.8	49.9	2	1	1	1	1	1	1	107.8	0.03	30.2
1	0	0	100	6.95	1	69	23	124	80	196	91	105	14.6	4.9	74.1	2	1	0	1	1	1	1	8.3	0.03	4.33
1	0	0	100	6.98	1	72	23	126	75.5	215	91	124	15.3	5.7	69.1	1	0	0	1	1	1	1	10.1	0.07	14.4
1	0	0	100	4.68	1	69	21.2	140	86.5	224	91	133	9.7	6.3	74.1	2	1	0	0	0	1	1	8.4	0.03	28.9
1	0	0	100	4.58	2	65	22.6	125	69.5	198	91	107	14.1	5.3	82.8	2	1	0	1	1	1	1	18.4	0.02	53.2
1	0	0	100	5.16	2	77	24.3	163.5	83.5	198	91	107	13.8	5.5	71.7	1	1	1	0	1	1	1	6	0.05	28.9
1	0	0	100	6.85	2	66	20.4	118	60.5	271	91	180	12.8	5.2	62.7	2	1	0	1	0	1	1	3.3	0.04	24.9
1	0	0	100	5.07	2	70	19.1	131.5	73.5	216	91	125	11.8	5.4	75.3	1	1	1	0	1	1	1	46.7	0.02	107
1	0	0	100	4.63	2	68	25.8	142.5	82.5	201	91	110	14.1	5.6	76.4	2	1	1	0	1	1	1	10.8	0.06	12.8
1	0	0	100	6.8	2	69	26.4	131	83.5	194	91	103	12.5	5.4	37.6	2	1	0	1	1	1	1	12.1	0.02	68.5
1	0	0	100	6.86	2	66	21.2	124.5	70.5	231	91	140	15.3	5.2	73.6	2	1	1	1	0	1	1	30.5	0.03	132
1	0	0	100	2.24	2	80	21.3	121.5	71	248	91	157	12.1	6.1	66.7	1	1	0	1	0	1	1	12.7	0.02	46.8
1	0	0	100	5.15	1	70	26.6	141.5	81	196	91	105	14.9	5.4	70.1	2	0	0	0	1	1	1	91.9	0.05	31.8
1	0	0	100	6.86	1	72	21.3	134	66	224	91	133	11.9	4.8	69.1	2	0	1	1	0	1	1	8.3	0.02	43
1	0	0	100	7	2	67	20.5	107	61	223	91	132	11.2	7.6	76.9	2	1	1	1	0	0	1	18	0.01	4.11
1	0	0	100	6.86	2	65	18	141.5	74	200	91	109	12.7	5.5	78.0	2	1	1	0	1	1	1	12.8	0.01	18
1	0	1	0.8	0.8	1	78	27.7	186.5	98.5	233	91	142	14.1	5.2	69.6	1	1	0	0	0	1	1	5.4	0.32	39.2
1	0	0	100	7.05	1	77	21.4	152.5	82.5	205	92	113	14.2	4.8	74.0	1	0	0	0	0	1	1	44.5	0.12	14.7
1	0	0	100	6.96	1	75	25.1	160.5	94	253	92	161	14.2	4.9	75.1	1	1	1	0	0	1	1	13.2	0.15	45

1	0	0	100	5.06	1	69	23.9	118.5	67	170	92	78	12.5	5.4	78.3	2	1	1	0	1	1	1	3.9	0.02	39.9
1	0	0	100	4.6	1	65	21.2	124	75.5	217	92	125	13.8	5.4	80.5	2	1	0	1	1	1	1	31.8	0.01	4.59
1	0	0	100	6.85	2	71	18.3	159.5	81	233	92	141	15	4.7	74.8	2	1	1	0	0	1	1	10.9	0.01	14.8
1	0	0	100	4.64	2	69	26.5	175	97.5	229	92	137	14.3	5.7	72.1	2	1	0	0	0	1	1	11.3	0.02	39.8
1	0	0	100	6.95	2	69	28.3	131.5	78	214	92	122	13.6	5.3	75.9	2	1	1	1	1	1	1	15.3	0.09	26.6
1	1	0	100	7.2	2	82	26.6	153	86	239	92	147	14.5	5.5	65.8	1	1	1	0	0	1	1	238.9	0.07	59
1	0	0	100	6.98	2	67	28.4	146	86	226	92	134	13.2	5.5	76.9	1	1	1	0	0	1	1	16.7	0.06	11.9
1	0	0	100	6.8	2	77	18.9	155	75	236	92	144	14.7	5.4	50.3	2	1	1	0	0	1	1	624.3	0.06	70
1	0	0	100	5.05	1	69	21.2	120.5	70	201	93	108	12.9	5.4	74.1	2	0	0	1	1	1	1	3.9	0.02	22.2
1	0	0	100	4.64	1	75	21.6	146	83.5	223	93	130	15	5.5	75.1	2	1	0	0	0	1	1	35.4	0.02	66.8
1	0	0	100	1.04	1	76	18.4	150.5	78	173	93	80	14.2	6	67.2	2	0	0	0	1	1	1	60.5	0.04	86.5
1	0	0	100	5.04	1	66	26	151	85	162	93	69	14	5.9	75.7	2	1	0	0	1	1	1	8.2	0.02	12.2
1	0	0	100	5.07	2	68	22.3	141	85.5	279	93	186	12.7	5.9	72.6	2	1	1	0	0	1	1	44.2	0.49	32
1	0	0	100	7	2	68	25	144.5	87	221	93	128	13.5	5.2	72.6	1	1	1	0	0	1	1	21.6	0.04	35.4
1	0	0	100	6.86	2	70	23.6	127	60	216	93	123	11.9	6.6	75.3	2	1	1	1	1	0	1	12.4	0.06	42
1	0	0	100	6.86	2	71	20.3	109	58.5	216	93	123	12.5	5	79.4	2	1	1	1	1	1	1	16.2	0.03	130
1	0	0	100	6.97	2	67	26.7	122	80.5	215	93	122	12.5	5.6	62.2	2	1	1	1	0	1	1	10	0.04	8.47
1	0	0	100	6.8	2	73	25.1	117.5	63	232	93	139	13.7	5.2	73.8	2	1	1	1	0	1	1	14.4	0.06	60.3
1	0	0	100	6.85	2	65	17.1	145	71.5	219	93	126	12	5.9	74.2	2	1	1	0	1	1	1	29.4	0.01	20.8
1	0	0	100	5.04	2	72	21.9	124	73.5	203	93	110	14.2	5.8	74.3	2	1	1	0	0	1	1	44.9	0.07	35.8
1	1	0	100	7.07	2	67	25.5	119	66.5	238	93	145	12.2	5.3	73.1	2	1	1	1	0	1	1	43.3	0.01	16.5
1	0	0	100	7	1	73	16.2	128	71.5	186	93	93	15.2	4.9	68.6	2	0	0	1	1	1	1	43.4	0.03	18.8
1	0	0	100	6.96	1	66	22.6	152.5	89	228	93	135	14.8	5.1	79.9	2	1	0	0	0	1	1	45.9	0.01	19
1	0	0	100	5.04	2	77	25.9	105	65	216	93	123	12.1	5.4	68.2	2	1	1	1	1	1	1	6.2	0.19	57.3
1	0	0	100	7	2	67	23.2	129.5	72	269	93	176	13.1	5.4	73.1	1	1	1	1	0	1	1	10.7	0.03	11.1
1	0	0	100	7.07	2	74	25.8	129.5	76	219	93	126	13	5.6	59.2	1	1	1	0	0	1	1	19.2	0.04	0.05
1	0	0	100	6.86	2	71	30.1	136	74	227	93	134	12.5	5.7	74.8	1	1	1	0	0	1	1	25.4	0.01	23.5
1	0	0	100	6.84	2	71	15.9	117.5	68.5	208	93	115	12.7	5.5	71.1	2	1	1	1	1	1	1	5	0.01	14.6
1	0	0	100	6.87	1	69	22.8	137	78.5	215	94	121	14.3	5.1	74.1	2	1	0	1	1	1	1	14.7	0.05	23.2
1	0	0	100	6.97	1	70	19	118.5	75.5	220	94	126	13.2	5.5	73.6	2	1	0	1	0	1	1	9	0.03	3.85
1	0	0	100	7.02	2	68	29.8	145.5	82	215	94	121	12.8	5.4	81.1	2	1	0	0	1	1	1	109.3	0.16	42.7
1	0	0	100	6.86	2	73	20.4	133	69.5	236	94	142	13.8	6.9	78.3	2	1	1	1	0	0	1	9.5	0.03	11.9
1	0	0	100	6.87	2	66	25.2	116.5	65	253	94	159	13.3	5.6	62.7	2	1	1	1	0	1	1	15.5	0.04	4.14
1	0	0	100	4.6	2	68	21.6	128	66	237	94	143	13.4	5.5	76.4	2	1	0	0	0	1	1	6	0.08	30.6
1	0	0	100	6.97	2	70	19	116	65.5	251	94	157	12.8	5.5	71.6	2	1	1	1	0	1	1	5.9	0.01	14.6
1	0	0	100	4.63	2	72	24.7	127	79	202	94	108	13.6	6	74.3	2	1	1	0	0	1	1	28.6	0.07	11.9
1	0	0	100	6.97	2	66	20.6	97.5	58.5	240	94	146	12.1	5.4	82.3	2	1	1	1	0	1	1	7	0.01	22.8
1	0	0	100	5.05	1	69	21	178.5	108.5	175	95	80	16.2	5.2	83.4	2	1	0	0	1	1	1	130.5	0.03	28.5
1	0	0	100	6.98	1	77	21.9	147.5	80.5	264	95	169	14.4	5.1	78.8	1	1	0	0	0	1	1	52.4	0.01	31.8
1	0	0	100	1	1	83	24	149.5	80	243	95	148	13.6	5	67.2	2	1	0	0	0	1	1	22.6	0.26	52.8
1	0	0	100	7.07	2	73	29	135.5	89	205	95	110	12.1	5.2	73.8	2	1	1	0	0	1	1	10.7	0.03	58.5
1	0	0	100	5.16	2	73	26.4	123	77	183	95	88	12.8	5.5	73.8	2	1	1	0	0	1	1	23.5	0.02	6.52
1	0	0	100	6.86	2	71	20.6	109.5	64.5	237	95	142	12	5.3	74.8	2	1	1	1	0	1	1	10.3	0.04	56.4
1	1	0	100	7.04	2	73	22	139	84	236	95	141	11.3	5.2	70.1	2	1	0	1	0	1	1	11.1	0.02	79.6
1	0	0	100	5.06	1	79	20.8	132	69	183	96	87	15.5	5.6	73.0	2	0	0	1	1	1	1	7.1	0.18	67.2
1	0	0	100	6.87	1	65	19.7	161	80.5	183	96	87	14.1	5.5	72.6	2	1	1	0	1	1	1	7.9	0.05	31.7
1	0	0	100	7.04	1	70	21.5	106	57	255	96	159	14.5	5.3	77.7	2	0	1	1	0	1	1	4.5	0.01	19.8
1	0	0	100	7.04	1	74	23.2	120.5	75.5	213	96	117	12.5	5.5	68.2	2	1	0	0	0	1	1	16.5	0.03	61.5
1	0	0	100	7	2	71	18.7	143	85	281	96	185	13.9	4.8	71.1	2	1	1	0	0	1	1	62	0.03	0.05
1	0	0	100	7.05	2	73	20.4	147.5	70	223	96	127	12	5.1	59.7	1	1	0	0	0	1	1	69.4	0.02	67.7
1	0	0	100	6.87	2	70	16.7	143.5	82	300	96	204	14.1	5.7	52.8	2	1	1	0	0	1	1	10.8	0.03	28.8

1	0	0	100	6.85	2	70	24.5	121.5	77.5	258	96	162	12.6	5.2	60.9	2	1	1	1	0	1	1	9.8	0.03	11.3
1	0	0	100	6.98	2	66	23.2	131.5	82	230	96	134	13.4	5.6	77.5	2	1	0	1	0	1	1	59.5	0.05	7.97
1	0	0	100	6.98	2	70	26	151.5	73.5	247	96	151	10.2	6.6	41.5	1	1	1	0	0	0	1	6198.9	0.1	61.9
1	0	0	100	7	2	70	22.2	141	82.5	235	96	139	14.9	5.4	71.6	1	1	0	0	0	1	1	25.9	0.02	3.23
1	0	0	100	6.96	1	76	21.3	167.5	78.5	210	97	113	14	4.5	74.5	1	1	0	0	1	1	1	86.9	0.03	44
1	0	0	100	6.8	1	71	22.2	132	89	175	97	78	16	5.4	69.6	2	0	0	1	1	1	1	7.1	0.03	11.2
1	0	0	100	7.08	2	75	25.1	157	93.5	194	97	97	15.1	5.8	58.8	2	1	1	0	1	1	1	87.3	0.02	71.8
1	0	0	100	4.6	2	65	24.9	133.5	81	234	97	137	13.4	5.4	78.0	2	1	1	1	0	1	1	83.9	0.05	24.6
1	0	0	100	6.98	2	66	19.6	150.5	80.5	244	97	147	11.7	5.2	82.3	2	1	1	0	0	1	1	31.5	0.03	5.82
1	1	0	100	5.37	2	70	30.8	146	72	355	97	258	13.5	5.3	60.9	2	1	0	0	0	1	1	92.7	0.05	51.1
1	1	0	100	6.86	2	81	19.3	156.5	63	245	97	148	12.4	5.7	69.7	2	1	1	0	0	1	1	20.9	0.04	226
1	1	1	2	2	1	75	22.5	127	74.5	175	97	78	13.9	5.6	75.1	2	1	0	1	1	1	1	32.1	0.04	13.5
1	0	0	100	7.07	1	72	22.8	138.5	80.5	224	98	126	14.7	4.9	72.6	2	0	0	1	0	1	1	173.9	0.09	86
1	0	0	100	7.07	2	73	22.2	142.5	77	253	98	155	13.7	5.2	70.1	2	1	1	0	0	1	1	8.3	0.03	11
1	0	0	100	6.87	2	67	26.1	141	72	247	98	149	13.4	5.3	73.1	2	1	1	0	0	1	1	8.1	0.04	51.7
1	0	0	100	6.84	2	68	20.2	120	67.5	217	98	119	11.7	5.6	72.6	2	1	1	1	1	1	1	14.4	0.02	28.9
1	1	0	100	7.21	2	70	24.2	112	63	265	98	167	13.9	10.2	75.3	2	1	1	0	0	0	1	10.1	0.05	54.5
1	0	0	100	7	1	67	34.3	147.5	80	210	99	111	15.9	6.5	79.4	2	1	0	0	1	0	1	164.7	0.01	23.8
1	0	0	100	6.87	1	66	22.6	120	85.5	239	99	140	16.6	6.9	72.1	2	1	0	1	0	0	1	47.2	0.02	6.99
1	0	0	100	4.62	1	70	23.2	166	88.5	285	99	186	15	5.3	73.6	2	1	0	0	0	0	1	15.5	0.03	12.9
1	0	0	100	5.06	1	69	25.3	139	84	186	99	87	13.7	5.6	62.2	2	0	0	1	1	1	1	5.8	0.06	72.1
1	0	0	100	5.07	2	68	22	108.5	55	211	99	112	13.9	5.4	76.4	2	1	1	1	1	1	1	5.1	0.53	12.4
1	0	0	100	6.87	2	74	20	126	70.5	250	99	151	12.9	4.6	69.6	2	1	1	1	0	1	1	15	0.08	5.7
1	0	0	100	5.16	2	73	25.9	115	62	212	99	113	12.4	5.7	70.1	2	1	1	0	0	1	1	12.6	0.01	12.7
1	0	0	100	6.85	2	77	26.9	111.5	63.5	208	99	109	13.7	6.5	71.7	2	1	1	1	0	0	1	4.5	0.05	8.01
1	0	0	100	6.98	2	70	21	191.5	106	213	99	114	12.3	5.5	71.6	1	1	1	0	1	1	1	126.9	0.02	7.55
1	0	0	100	6.98	2	65	20.4	117	59	248	99	149	13.5	4.8	82.8	2	1	1	1	0	1	1	11.9	0.01	0.05
1	0	0	100	6.87	1	75	22.8	131.5	77	174	99	75	12.3	4.4	75.1	2	1	0	0	1	0	1	67.1	0.24	50.7
1	0	0	100	5.04	2	71	16.5	108.5	59.5	235	99	136	11.7	5.4	79.4	1	1	0	0	0	1	1	26.8	0.01	31.6
1	0	0	100	2.68	1	73	21.8	122	87	179	100	79	16.3	6	76.1	1	0	0	1	1	1	1	16.9	0.06	41.2
1	0	0	100	4.62	2	76	22.7	128.5	79.5	266	100	166	13.2	5.5	68.6	2	1	1	0	0	1	1	15.9	0.15	38.2
1	0	0	100	6.88	2	65	21.2	117	64.5	208	100	108	13.6	4.9	63.1	2	1	1	1	1	1	1	8	0.04	41.5
1	0	0	100	5.16	2	76	19.7	145	75.5	234	100	134	13.7	6.3	58.4	1	1	1	0	0	0	1	14.4	0.08	40.5
1	0	0	100	7	2	68	20.5	108	69.5	221	100	121	12.2	5	76.4	1	1	1	1	0	1	1	34.1	0.02	12.3
1	0	0	100	6.87	2	70	17.4	102	60	208	100	108	12.4	5.5	75.3	2	1	1	1	1	1	1	76.3	0.04	1.83
1	0	0	100	4.62	1	80	24.8	156.5	78.5	209	100	109	14.9	5.8	72.5	2	0	0	0	1	1	1	15.7	0.04	26.6
1	0	0	100	6.97	1	76	16.2	113.5	58	222	100	122	15.7	5.4	74.5	2	1	1	1	0	1	1	38.5	0.03	30.9
1	0	0	100	5.37	2	79	18.4	170	95	210	100	110	12.1	5.6	57.2	1	1	1	0	1	1	1	30.4	0.09	162
1	0	0	100	6.84	2	66	23.2	124.5	80.5	246	101	145	13.9	5.5	73.6	2	1	0	1	0	1	1	10.6	0.08	34.3
1	0	0	100	7	2	66	21.8	95.5	54.5	227	101	126	11.3	5.5	77.5	1	1	1	1	0	1	1	13.6	0.03	64.7
1	0	0	100	6.95	2	69	30.7	153	86.5	228	101	127	15.9	7	72.1	2	1	1	0	0	0	1	20.7	0.02	13.1
1	0	0	100	6.98	2	71	22.2	150.5	90	241	101	140	13.8	5.8	79.4	1	1	1	0	0	1	1	20.3	0.05	22.9
1	0	0	100	6.97	1	70	20.9	126.5	68.5	210	102	108	13.4	4.9	77.7	2	1	0	0	1	1	1	16.1	0.01	48.1
1	0	0	100	5.08	2	72	24.8	131	78	183	102	81	14.2	6.1	74.3	2	1	1	0	1	1	1	47.6	0.1	19.5
1	0	0	100	5.15	2	70	28.1	142.5	88.5	205	102	103	13.3	5.4	71.6	1	1	1	0	0	1	1	15.6	0.01	17
1	0	0	100	0.68	1	65	23.5	155.5	98.5	210	103	107	13.8	5.7	76.2	2	1	0	0	1	1	1	33.4	0.02	39.4
1	0	0	100	6.95	1	71	20.8	117.5	64.5	210	103	107	14	5.7	77.2	2	1	1	1	1	1	1	10.9	0.01	9.33
1	0	0	100	6.82	2	66	18.5	111	68	215	103	112	12	5.9	77.5	2	1	0	0	1	1	1	11.6	0.05	41.1
1	0	0	100	4.58	2	67	23.1	109	64.5	253	103	150	12.9	5.5	76.9	2	1	1	1	0	1	1	11.3	0.02	4.19
1	0	0	100	6.88	2	74	31.1	179	93.5	262	103	159	14.5	5.3	69.6	2	1	1	0	0	1	1	16.4	0.04	57
1	0	0	100	6.97	2	66	20.8	103.5	62	206	103	103	11.7	5	77.5	2	1	1	1	0	1	1	68.9	0.01	7.42

1	0	0	100	6.84	2	76	18.2	131.5	77.5	207	103	104	12.3	5.6	68.6	2	1	1	1	0	1	1	13.8	0.03	25.7
1	0	1	1	1	2	83	20.1	125.5	79	240	103	137	11.7	5.5	55.6	1	1	1	0	0	1	1	7.8	0.02	63.2
1	0	0	100	6.95	1	67	24	150	89.5	203	104	99	14.2	5.1	79.4	2	0	0	0	1	1	1	8.9	0.05	11.3
1	0	0	100	6.98	1	67	21.3	140	74	191	104	87	13.4	5	71.6	2	1	0	0	1	1	1	7.4	0.03	59.6
1	0	0	100	6.87	1	70	21.7	138.5	92.5	217	104	113	12.6	5.2	82.8	2	1	0	0	1	1	1	18.8	0.01	8.16
1	0	0	100	6.88	2	67	17.1	96	50.5	203	104	99	13.1	5.3	73.1	2	1	1	1	1	1	1	9.2	0.01	34.1
1	0	0	100	4.63	2	65	16.6	107.5	73.5	227	104	123	12	5	78.0	2	1	1	1	0	1	1	3.4	0.01	73.9
1	0	0	100	6.95	2	68	22.5	122	64	215	105	110	13.5	5.2	76.4	2	1	1	0	1	1	1	59.5	0.01	14.2
1	0	0	100	4.7	2	66	27.1	155	82	204	105	99	15	5.6	77.5	2	1	0	0	1	1	1	40	0.06	4.05
1	0	0	100	4.62	2	65	20.9	137.5	87.5	190	106	84	13.3	5.3	78.0	2	1	1	1	1	1	1	29.2	0.02	15.1
1	1	0	100	5.05	2	69	20	114	67	191	106	85	13.4	6.1	75.9	2	1	1	1	1	1	1	12.2	0.01	22.8
1	1	0	100	6.97	2	73	23.2	140.5	77.5	248	106	142	14.6	4.9	73.8	1	1	1	0	0	1	1	21.9	0.01	11.2
1	0	0	100	6.85	1	68	24.9	128.5	82.5	253	106	147	13.9	5.2	71.1	2	1	0	1	0	1	1	8.1	0.03	28.4
1	0	0	100	5.48	1	75	17.7	121	68	206	108	98	13.5	5.2	71.0	2	1	0	1	1	1	1	12.3	0.25	27.7
1	0	0	100	7.04	1	70	18.4	89.5	57.5	196	108	88	12.5	5.1	77.7	1	0	0	1	1	1	1	7.9	0.01	25
1	1	0	100	5.15	1	74	24.1	90	52	189	108	81	13.1	5.6	71.5	2	1	1	1	1	1	0	12.4	0.03	94.4
1	0	0	100	4.69	2	72	24.1	162.5	92.5	216	108	108	13.6	7.7	78.9	1	1	1	0	1	0	1	21.6	0.16	6.22
1	0	0	100	4.58	2	65	31.8	134.5	88.5	255	108	147	13.2	5.9	74.2	2	1	1	1	0	1	1	14.9	0.02	21.8
1	0	0	100	7.02	2	72	21	117.5	74	259	108	151	13.2	5.5	74.3	1	1	1	0	0	1	1	19.4	0.02	47.8
1	0	0	100	5.04	2	72	20	104	59.5	264	108	156	13.2	5.5	78.9	2	1	1	1	0	1	1	29.2	0.01	93.8
1	0	0	100	6.86	2	66	28.8	154	94	248	109	139	13.2	5.4	77.5	2	1	1	0	0	1	1	7.2	0.7	33.4
1	0	0	100	6.79	1	74	19.5	124	76.5	219	110	109	14.4	5.9	68.2	2	1	1	1	1	1	1	14.1	0.05	22.9
1	0	0	100	5.37	1	75	19.8	111	73	198	111	87	14.3	5.5	67.7	2	1	0	1	1	0	1	7.6	0.03	19.7
1	1	0	100	2.7	1	74	15.1	112.5	63.5	253	111	142	14.6	12.4	80.5	2	1	1	1	0	0	1	113.3	0.01	6.03
1	0	0	100	6.85	2	70	17.7	141	76	247	111	136	12.6	5.3	80.0	2	1	1	0	0	1	1	18	0.01	26.7
1	0	0	100	6.97	1	68	19.5	118	71	199	112	87	13.7	5.6	78.8	2	1	0	1	1	1	1	13.7	0.14	17
1	0	0	100	4.67	1	66	22.2	148.5	81	271	112	159	15.5	5.1	79.9	2	1	0	0	0	1	1	215.3	0.03	12.3
1	0	0	100	6.95	1	74	22.7	114	65	229	113	116	14.4	5.4	80.5	2	1	0	1	0	0	1	21.9	0.01	8.39
1	0	0	100	4.69	1	66	21.1	153	90.5	220	113	107	13.1	5.7	79.9	2	1	0	0	0	1	1	9.6	0.01	26.4
1	0	0	100	5.05	2	68	20.8	106	60.5	219	113	106	12.8	5.4	76.4	2	1	1	1	1	1	1	6.6	0.01	27.9
1	1	0	100	6.44	2	78	19.1	132	72	238	113	125	13.7	5.4	50.0	2	1	0	0	0	1	1	12	0.01	56.9
1	0	0	100	6.95	1	77	21.1	136	68.5	236	114	122	13.9	5.2	70.1	2	1	0	1	0	1	1	20.8	0.01	6.34
1	0	0	100	5.06	2	75	25.5	136.5	72.5	202	114	88	14.4	5.3	69.1	2	1	0	1	1	1	1	41.2	0.04	32.2
1	0	0	100	4.64	2	67	25.1	124	71.5	262	116	146	14	5.7	76.9	2	1	0	1	0	1	1	9.5	0.02	6.78
1	0	0	100	6.87	2	65	22.7	104	68	219	118	101	13.4	5	89.1	2	1	1	1	1	1	1	14.3	0.03	37
1	0	0	100	6.87	2	65	16.5	99.5	58.5	227	119	108	12.3	5.5	82.8	2	1	1	1	0	1	1	14.6	0.1	23.1
1	0	0	100	4.63	2	75	18.4	146	74.5	253	119	134	11.9	5.5	69.1	2	1	1	0	0	1	1	103.8	0.01	43.8
1	0	0	100	6.98	1	76	19	96.5	50.5	235	120	115	14.3	5.8	70.5	1	0	0	0	0	1	1	65.8	0.35	71.3
1	0	0	100	4.69	1	74	21.2	123	80.5	235	120	115	15.5	5.5	75.6	2	0	0	0	0	1	1	56.1	0.03	43.6
1	0	0	100	6.84	1	72	15.9	115.5	68.5	255	122	133	13.5	5.8	72.6	2	1	0	1	0	1	1	8.3	0.01	19.4
1	0	0	100	6.98	1	66	22.4	127	78.5	216	123	93	15.7	4.8	79.9	2	0	0	1	1	1	1	10.2	0.03	21.9
1	0	0	100	5.12	1	78	17.9	161.5	88	258	126	132	11.7	6.8	66.3	2	1	0	0	0	0	1	33.2	0.01	75.1
1	0	0	100	6.87	1	71	22.3	143	77.5	216	130	86	13.9	5	69.6	2	1	0	0	1	1	1	5.4	0.01	60.3
1	0	0	100	4.61	1	65	27.8	140	75.5	257	166	91	15	5	80.5	2	1	1	0	0	1	1	22.8	1.18	18.5
1	0	0	100	4.59	2	69	23.1	113.5	69.5	328	168	160	13.9	4.8	75.9	2	1	1	0	0	1	1	38.4	0.02	22.7
